# Genome-wide identification and functional analysis of Dof transcription factor family in *Camelina sativa*

**DOI:** 10.1186/s12864-022-09056-9

**Published:** 2022-12-08

**Authors:** Tao Luo, Yanan Song, Huiling Gao, Meng Wang, Hongli Cui, Chunli Ji, Jiping Wang, Lixia Yuan, Runzhi Li

**Affiliations:** 1 Institute of Molecular Agriculture and Bioenergy, College of Agriculture, Shanxi Agricultural University, Taigu, 030801 China; 2 College of Biological Science and Technology, Jinzhong University, Jinzhong, 030600 Shanxi China

**Keywords:** Oil crop, Dof transcriptional factors, Genome-wide characterization, Expression profiling, Lipid metabolism, Stress responses

## Abstract

**Background:**

Dof transcription factors (TFs) containing C_2_-C_2_ zinc finger domains are plant-specific regulatory proteins, playing crucial roles in a variety of biological processes. However, little is known about Dof in *Camelina sativa,* an important oil crop worldwide, with high stress tolerance. In this study, a genome-wide characterization of Dof proteins is performed to examine their basic structural characteristics, phylogenetics, expression patterns, and functions to identify the regulatory mechanism underlying lipid/oil accumulation and the candidate Dofs mediating stress resistance regulation in *C. sativa*.

**Results:**

Total of 103 *CsDof* genes unevenly distributed on 20 chromosomes were identified from the *C. sativa* genome, and they were classified into four groups (A, B, C and D) based on the classification of Arabidopsis *Dof* gene family. All of the CsDof proteins contained the highly-conserved typic CX_2_C-X_21_-CX_2_C structure. Segmental duplication and purifying selection were detected for *CsDof* genes. 61 *CsDof* genes were expressed in multiple tissues, and 20 of them showed tissue-specific expression patterns, suggesting that *CsDof* genes functioned differentially in different tissues of *C. sativa.* Remarkably, a set of *CsDof* members were detected to be possible involved in regulation of oil/lipid biosynthesis in *C. sativa*. Six *CsDof* genes exhibited significant expression changes in seedlings under salt stress treatment.

**Conclusions:**

The present data reveals that segmental duplication is the key force responsible for the expansion of *CsDof* gene family, and a strong purifying pressure plays a crucial role in *CsDofs’* evolution. Several CsDof TFs may mediate lipid metabolism and stress responses in *C. sativa.* Several CsDof TFs may mediate lipid metabolism and stress responses in *C. sativa.* Collectively, our findings provide a foundation for deep understanding the roles of CsDofs and genetic improvements of oil yield and salt stress tolerance in this species and the related crops.

**Supplementary Information:**

The online version contains supplementary material available at 10.1186/s12864-022-09056-9.

## Introduction

Lipids are major and essential components of all plant cells, not only providing structural integrity and energy for various metabolic processes, but functioning in intra- and inter-cellular signal transduction [1]. It has been found that lipids/fatty acids and their oxidation products are involved in plant responses against diverse stresses, including salt, drought, heavy metal, pathogen infection and other adversities [2–6]. Importantly, vegetable oils are necessary for human health and animal nutrition [7–9], also increasing to be as sources of a new generation of environmentally friendly, biodegradable, and renewable industrial products such as biopolymers, biodiesel and high-grade lubricants [10, 11]. To meet a growing market demand for vegetable oils globally, it is so much imperative to largely increase vegetable oil yields, which needs to decipher the mechanism responsible for lipid/oil biosynthesis and regulation in oil-rich plants.

Camelina (*Camelina sativa* (L.) Crantz), a “low-input and environment-friendly” crop with a high seed oil content (36–47%) [12], has become a widely-grown oil crop in the world. *C. sativa* seed oil is extremely high in unsaturated fatty acids (UFAs) (up to 90%), particularly 40% of health-promoting ɑ-linolenic acids (ω-3 fatty acids) [12–14]. Servicing as the sustainable feedstock for the commercial production of food, feed, biofuel (e.g., aviation fuel and biodiesel), and other high-value industrial products. In addition, compared to traditional oil crops (e.g., soybean and oilseed rape), *C. sativa* has excellent agronomic traits such as a short growth cycle, tolerance to abiotic stresses, and resistance to common pests and disease [15]. Given such high-value applications of the seed oils and plant strong stress tolerance, increasing efforts are focused on elucidation the regulatory mechanism mediating oil biosynthesis and stress responses in *C. sativa*.

Plant lipid metabolism and stress responses are modulated by diverse regulatory pathways. One of important players involving in such complicated regulatory networks is transcription factor (TF), which can mediate coordinately multiple biological processes by regulating expressions of the related target genes [16]. TFs can interact with other regulatory factors or directly bind with cis-elements to regulate transcriptional expression of the downstream genes. To date, a number of TFs have been identified to regulate plant lipid biosynthesis and stress responses, including LEC1 [17], WRI1 [18], FUS3 [19], MYB [20], bZIP [21], Dof [22, 23], NAC [24], and WRKY [25]. The Dof (DNA binding with one finger) TF is a member of the Zinc finger superfamily, characterized by a C_2_-C_2_ type zinc finger domain containing 50–52 amino acids [26]. Since the isolation of *ZmDof 1* encoding a Dof protein from maize, Dof TF family has been successively detected in various plants. For instance, 36, 30, 37,58, 55, 89 and 110 Dof members were identified, respectively, in *Arabidopsis* [27, 28], rice (*Oryza sativa*) [27, 29], tomato [30], *Gossypium arboretum, Gossypium raimondii* (diploid cotton), *Gossypium hirsutum* (tetraploid cotton) and *Gossypium barbadense* (tetraploid cotton) [31]. The known studies showed that Dof TFs functioned diversely in plants. In *Arabidopsis*, *Dof* genes are expressed in vascular tissues, influencing vascular tissue development and formation [32], and a similar function was later found in tomato [33]. *Arabidopsis* CDF1 (Cycling Dof Factor 1) is involved in photoperiodic regulation and flowering inhibition [34]. *Arabidopsis* CDF4 increases ABA levels to positively regulate leaf senescence, and also promotes floral organ abscission through activation of the *PGAZAT* gene [35]. Moreover, OsDof 3 regulates GA reaction through interaction with GAMYB [36]. AtDof 3.2, as a negative regulator of seed germination, interacts with TCP14, a positive regulator of seed germination [37]. Barley Dof-encoding gene (scutellum and aleurone-expressed DOF, *SAD*) can activate endosperm specific gene transcription through interaction with R2R3MYB protein [38]. Maize *ZmDof 36* up-regulated the expression levels of genes related to starch synthesis while significantly increasing starch content [23]. For *Chlamydomonas reinhardtii*, overexpression of endogenous *CrDof* significantly increased the lipid content in algae cells, and genes related to fatty acid synthesis and elongation were significantly up-regulated [22, 39]. Among the soybean Dof family members, overexpression of *GmDof 4* and *GmDof 11* also significantly increased lipid content in transgenic *Arabidopsis* seeds [40]. Similarly, *GmDof 4* overexpression led to enhancement of oil level in cells of *Chlorella vulgaris* by 46.4 to 52.9% [41]. In addition, Dof proteins were identified to plays a role in stress response. Wheat TaDof showed distinct preference or specificity in a variety of abiotic stress responses [42]. AtDof 5.8 TF directly regulates ANAC069 which is a plasma membrane-bound NAC protein, integrating auxin and salt signals to regulate seeds germination in *Arabidopsis*. Interestingly, these two genes showed similar response patterns to salt, drought and ABA treatment in *Arabidopsis* [43]. Salt stress can significantly affect the photosynthesis and growth and development of *C. sativa*, leading to stunted plant growth [44]. During this stress response, a large number of genes related to lipid, amino acid and nucleotide synthesis are up-regulated or down-regulated in roots and stems [44]. Whether any member of Dof TF family also functions importantly in regulation of salt tolerance remains to be investigated in Camelina.

Collectively, these previous studies indicate that the regulatory functions of Dof TFs are quite comprehensive, including mediating oil metabolism pathway, abiotic stress response, plant growth and development, and other life activities. Based on these findings, we hypothesized that Dof TFs might mediate various life processes in *C. sativa*, including lipid metabolism and stress responses. However, no detailed characterization of the Dof TF family members has been reported so far in this important oilseed crop with a highly undifferentiated hexaploid genome structure. Therefore, we performed a genome-wide characterization of CsDof TFs in *C. sativa*, aiming to elucidate their biological functions, particularly in mediating lipid biosynthesis and stress responses. To understand the evolutionary relationships of CsDofs, we constructed the phylogenetic tree of CsDofs with *Arabidopsis* AtDof proteins, and also employed the inter-genomic synteny analysis between *C. sativa* and other Cruciferae plants. Moreover, we analyzed the spatio-temporal expression patterns of *CsDof* genes. Real-time RT-PCR (qRT-PCR) was also conducted to examine a number of *CsDof* genes in responses to salt stress. The current study will generate new scientific data to further revealing the regulation network mediated by CsDof TFs, providing valuable insights into the genetic improvement of *C. sativa* and other crops, especially plant resistance to various stresses and lipid biosynthesis.

## Results

### 103 CsDof proteins were identified in *C. sativa*

To identify the Dof proteins encoded in the Camelina genome, all 36 AtDof proteins from *Arabidopsis* were used as query sequences to perform genome-wide detection by BLASTp (Basic Local Alignment Search Tool) in the publicly available genome database of *C. sativa*. All candidate sequences were then validated using the HMM (Hidden Markov Model) with the Dof domain (PF02701). Finally, a total of 103 CsDof proteins were obtained (Additional file 1). These CsDof proteins were renamed from CsDof 1 to CsDof 103, and their characteristics were further examined, including the gene locus ID, gene starting and ending positions on chromosomes, protein sequence length (SL), molecular weight (MW), isoelectric point (pI), and member classification (Additional file 1). The length of CsDof protein sequence ranged from 174 (CsDof 36 and CsDof 86) to 587 amino acids (CsDof 56), with an average of 380 amino acid residues. The CsDof MWs ranged from 19.24 kDa (CsDof 36) to 63.06 kDa (CsDof 56), with an average of 41.15 kDa. In addition, the pIs of CsDof 1 and CsDof 11 were the smallest (4.47), and CsDof 55 was the largest (10.06), with an average of 8.37, indicating that most of them were basic proteins. Notably, compared with the numbers of Dof family members reported in other plant species, the CsDof family with 103 members is one of the larger Dof families tested demonstrates that this TF family was extensively expanded in *C. sativa* genome during its evolutionary process.

### Multiple sequence alignment and phylogenetic analysis of CsDof proteins

**Fig. 1:**
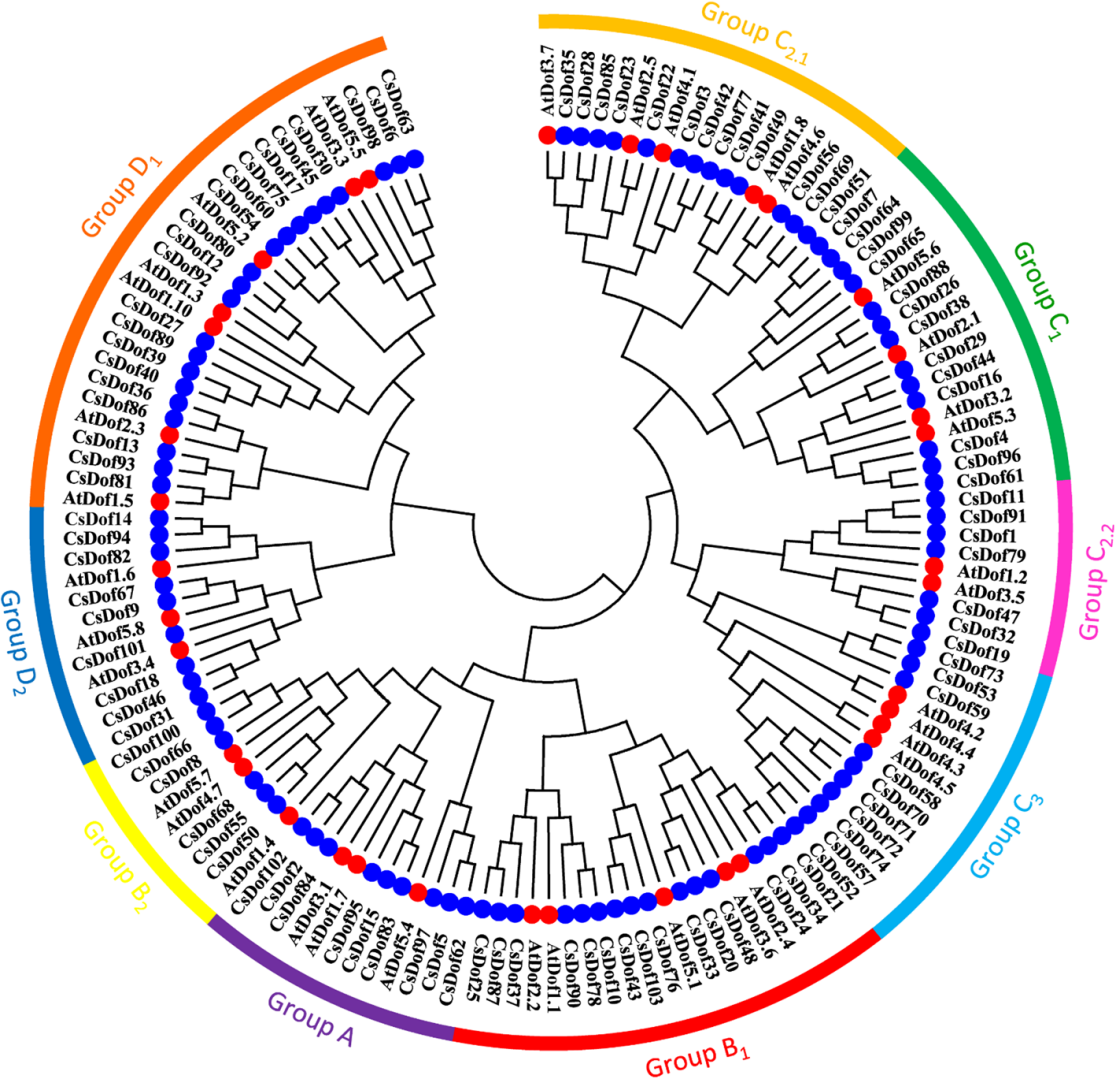
Comparative phylogenetic tree showing the domains relationship of CsDofs and AtDofs. The identified CsDofs (blue solid circle) and AtDofs (red solid circle) were clustered to the different groups in phylogenetic tree. The various colors on the periphery represent the different groups (or subgroups) of CsDof domains

**Fig. 2:**
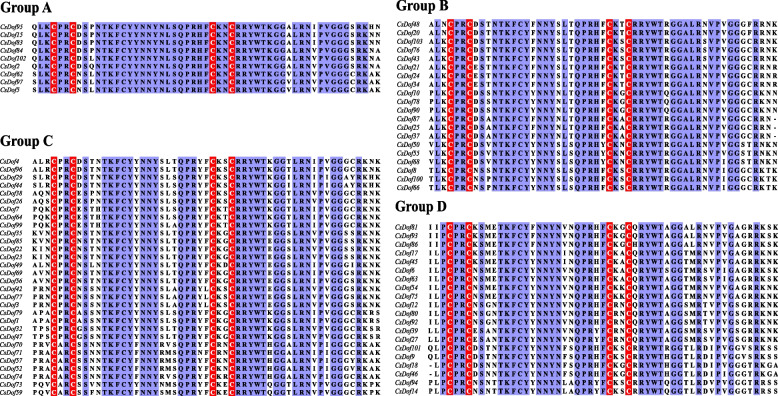
Multiple sequence alignment of the Dof domain from CsDofs. Red box indicated the C_2_C_2_-tepy zinc-finger domain

In order to ascertain the evolutionary relationship among CsDof proteins, a phylogenetic tree containing 36 AtDofs and 103 CsDofs was constructed with MEGA7.0 software using the neighbor-joining method (Fig. 1). Eventually, CsDof was classified into four groups (group A, B, C and D) [27], with 9 CsDofs in group A, 21 CsDofs in group B, 43 CsDofs in group C, and 30 CsDofs in group D. Furthermore, group B, C, and D were divided into several subgroups (B_1_, B_2_, C_1_, C_2.1_, C_2.2_, C_3_, D_1_ and D_2_) [27]. Then, the CsDof sequences randomly selected from group A, B, C and D were used for multiple sequences alignment. As shown in the Fig. 2, all these sequences contained a CX_2_C-X_21_-CX_2_C type zinc finger domain, the typical nature of Dof proteins. The conserved domain consisted of 52 amino acid residues, of which four cysteine residues coordinated with zinc in the N-terminal region.

### Conserved motif and gene structure analysis of CsDof proteins

**Fig. 3:**
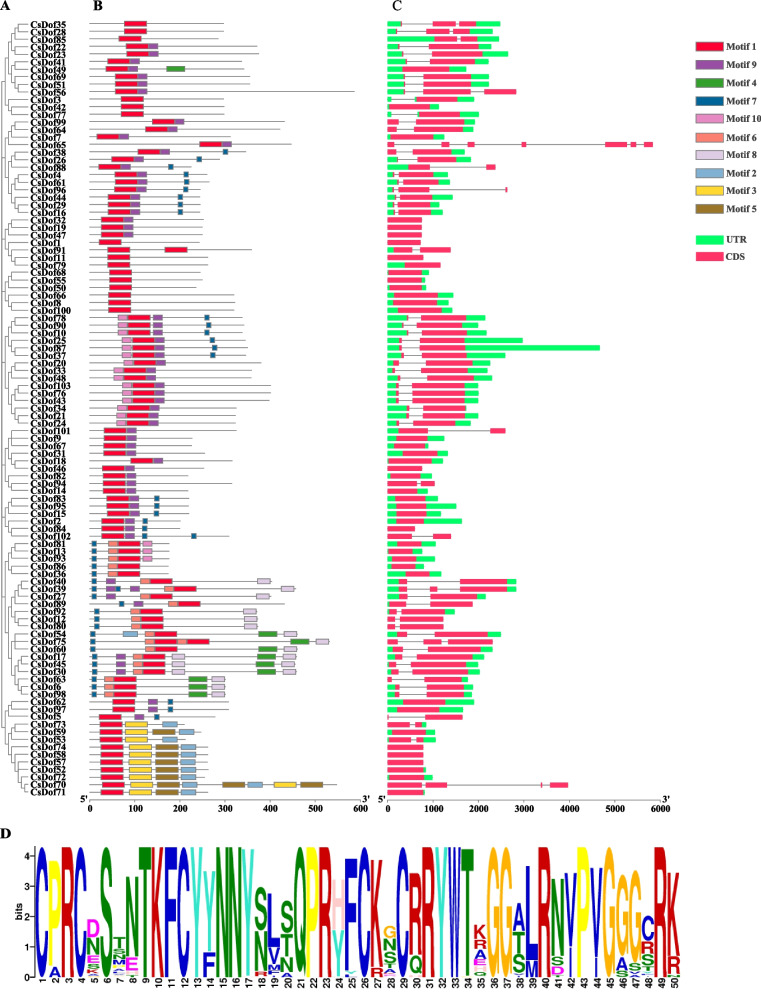
Analysis of conserved motif and gene structure of *C. sativa* Dof family. **A** The evolutionary tree of CsDof family. **B** Motif analysis of CsDof family. **C** The gene structure of *CsDof*. **D** Dof conservative motif (motif1) of *C. sativa*. The various color boxes represent different motifs, or gene structures

To reveal the functional regions of CsDof proteins, conserved motifs were predicted by the MEME (Multiple Em for Motif Elicitation) program, and a total of 10 conserved motifs (denoted as Motif 1 to Motif 10) were detected in all the 103 Dof proteins (Fig. 3A, B and Additional file 2). Among these conserved motifs, Motif 1 (Fig. 3D and Additional file 2) is a conserved Dof domain found in all CsDof proteins. In accordance with the results of conservative motif analysis, two Motifs 1 were detected in CsDof 91 and CsDof 75, respectively (Fig. 3B). Moreover, Motif 9 was predicted in most subgroups except for B_2_ and C_3_. For example, the D_2_ subgroup contained both Motif 9 and Motif 1. Motifs 6 and 8 were detected only in the D_1_ subgroup, while Motifs 3 and 5 were found only in the C_3_ subgroup. In addition, Motifs 2 and 4 were mainly found in C_3_ and D_1_ subgroups, respectively.

Analysis of *CsDof* gene structure (Fig. 3C) showed that most *CsDof* genes contained coding regions (CDS) and untranslated regions (UTR), while 17 *CsDof* genes were absent of UTRs, such as *CsDof 65*, *CsDof 32* and *CsDof 58*. The number of exons of *CsDof* genes ranged from one to seven, while the number of introns was from one to six. Total of 41 *CsDof* genes had only one exon. Introns occurred in 41 *CsDof* genes, accounting for 39.8% of the all *CsDof* genes, such as *CsDof 8*, *CsDof 66* and*CsDof 49* (Fig. 3C). Furthermore, several *CsDof* genes including *CsDof 39*, *CsDof 65*, *CsDof 70* and *CsDof 75* showed the characteristics of split genes (Fig. 3C).

### Uneven distribution of *CsDof* genes on chromosomes

To determine the distribution of *CsDof* genes on chromosomes, all identified *CsDof* genes were mapped onto chromosomes by TBtools software [45]. A total of 102 *CsDofs* ORFs were located on 20 chromosomes of Camelina (Additional file 3), including two alternatively spliced variants (*CsDof 39* and *CsDof 40*) produced by one *CsDof* gene locus (*Csa07g036310*). A single *Dof* gene (*CsDof 1*) did not map to any chromosome, but appeared in the scaffold region (Scaffold00734). 13 *CsDof* genes were located in Chromosome 11 while only one gene mapped to Chromosome 1, 15, 19 and 20, respectively. The number of *CsDof* genes located on the rest chromosomes ranged from two to eight. This indicates that *CsDof* genes are unevenly distributed on chromosomes.

### Segmental duplication and comparative syntenic maps

**Fig. 4:**
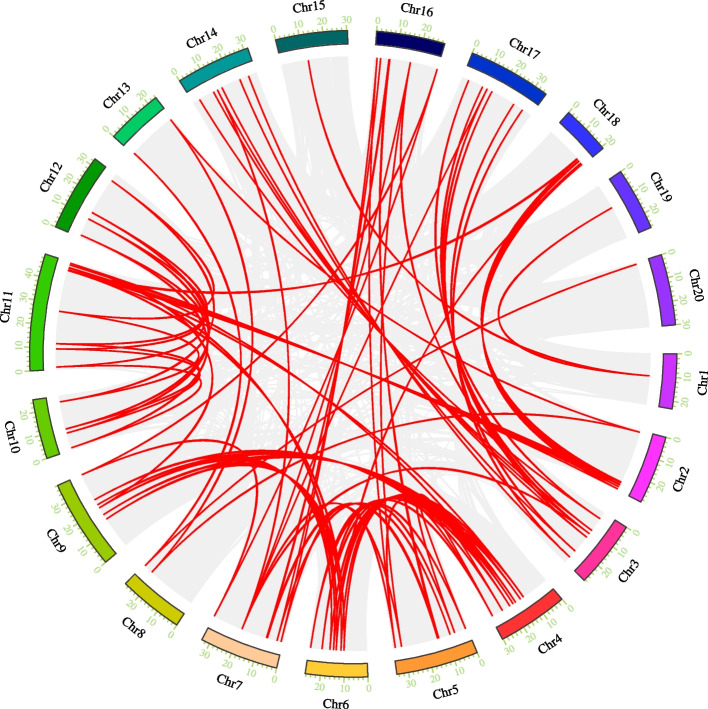
Synteny analysis of interchromosomal relationships of *CsDof* genes. All gene pairs and *CsDof* gene pairs in the *C. sativa* genome were indicated by gray lines and red lines respectively

It is recognized that expansion of a gene family is due to duplication events that occur within the entire family or between some members. Compared with other species including model plants *Arabidopsis* and rice, *C. sativa* contains more *Dof* genes, showing that *CsDof* family expands greatly. To elucidate the mechanism responsible for such expansion, TB tools and MCScanX [45] were used to examine the duplication pattern of *CsDof* genes, including tandem and segmental duplication. A total of 82 pair segmental duplication genes (Fig. 4 and Additional file 4) were identified in the *CsDof* gene family, but no tandem duplication was detected in any *CsDof* gene. These results revealed that segmental replication might be the core driving force for *CsDof* gene family evolution.

To investigate whether selection pressure (purifying and positive selection) acts on *CsDof* gene family formation, the synonymous substitution rates (Ks) and non-synonymous substitution rates (Ka) as well as the Ka/Ks of the identified orthologous *CsDof* gene pairs were calculated using KaKs Calculator 2.0 (Additional file 4). Only a duplicate gene pair of Csa10g022470/Csa12g037530 had the value of Ka/Ks (1.061) > 1, whereas the rest of the duplicate *CsDof* gene pairs exhibited the Ka/Ks value less than 0.8, indicating that the *CsDof* gene family may be mainly affected by purifying selection or negative selection in their evolution. Duplication events of these 82 *CsDof* gene pairs were calculated to be occurred between 0.91 and 70.56 million years ago (Additional file 4).

**Fig. 5:**
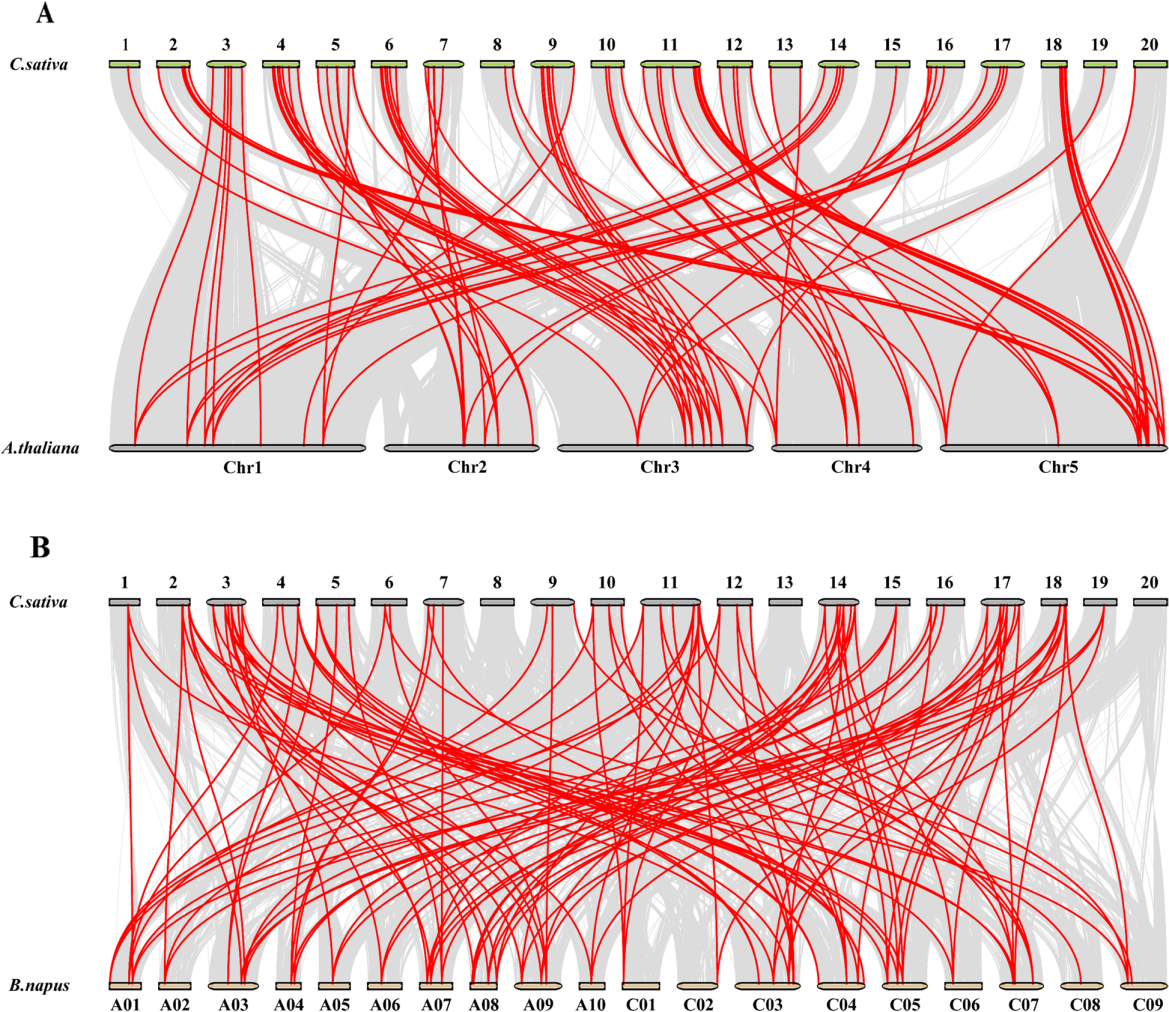
Synteny analysis of *CsDof* genes between *C. sativa* and two other related plant species (*A. thaliana* and *B. napus*). **A** *C. sativa* and *A. thaliana*. **B** *C. sativa* and *B. napus*. The collinear blocks between *C. sativa* and other species were showed gray lines. The syntenic *Dof* gene pairs between *C. sativa* and *A. thaliana* or *B. napus* were highlighted with red. The chromosome number was indicated at the top or bottom of every chromosome

To further explore the origin and evolution of *CsDof* genes, the comparative synteny analysis was performed between *C. sativa* and the two closely-related plant species *Arabidopsis thaliana* and *Brassica napus*. As shown in the comparative synteny maps (*C. sativa* VS *A. thaliana* and *C. sativa* VS *B. napus*), Orthologous relationships were detected between 84 *CsDof* genes and 31 *AtDof* genes, and then 84 orthologous *Dof* gene pairs were identified accordingly (Fig. 5A and Additional file 5), with all of them located on the syntenic locus in *Arabidopsis* and *C. sativa* chromosomes. Remarkably, multiple *CsDof* genes were identified as putative orthologs of a single *AtDof* gene. 24 *A. thaliana Dof* three syntenic copies were detected in *C. sativa*. For example, *Csa14g009010* (*CsDof 78*), *Csa17g011020* (*CsDof 90*) and *Csa03g011080* (*CsDof 10*) were identified as the ortholog of *AT1G07640* (*AtDof 1.1*) (Additional file 5). In addition, 71 *BnaDof* genes, located on chromosomes A01-A10 and C01-C09 in *B. napus* (Additional file 6), were selected for collinearity analysis with *C. sativa*. The orthologous relationships were also detected between 59 *CsDof* genes and 44 *BnaDof* genes, and the corresponding 115 orthologous *Dof* gene pairs were built between these two species (Fig. 5B and Additional file 7).

### Multiple response elements exist in the *CsDof* promoter region

**Fig. 6:**
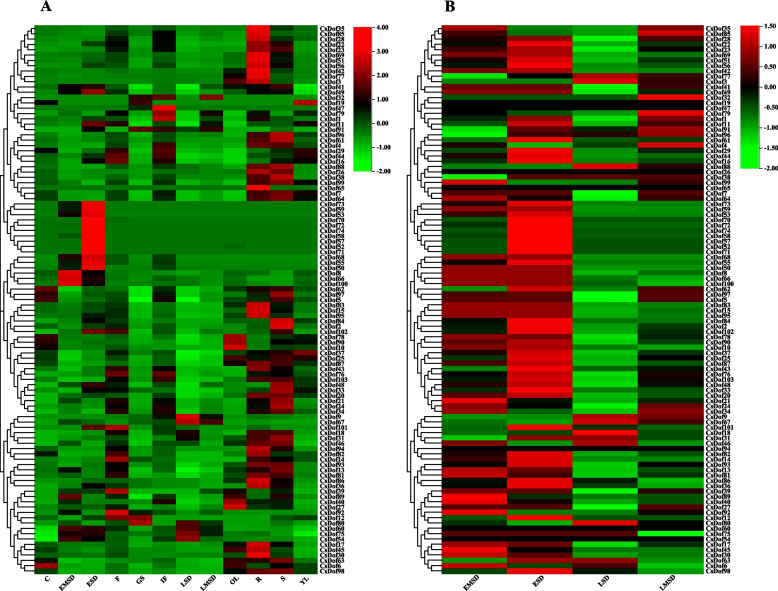
Heat map representation and hierarchical clustering of the *CsDof* gene expression profiles in twelve tissues. **A** Expression profile of *CsDof* genes in 12 tissues. **B** Expression profiles of *CsDof* genes in seeds at different developmental stages. The expression levels of *CsDof* genes were showed by different colors on the right scale. C, cotyledon; EMSD, early-mid seed development; ESD, early seed development; F, flower; GS, germinating seed; IF, inflorescence; LSD, late seed development; LMSD, late-mid seed development; OL, mature leaf; R, root; S, stem; YL, young leaf

In order to further understand the transcriptional regulation mechanism, the 2000-bp promoter region upstream from the starting codes of *CsDof* genes in *C. sativa* was taken for cis-element analysis by PlantCARE database. As a result (Table 1 and Additional file 8), a variety of cis-acting elements were identified, including growth and development elements, stress response elements, light response elements, hormone response elements and so on. Strikingly, 70 and 98% of the *CsDof* genes were detected to contain light- and hormone-responsive elements in their promoters, respectively, indicating that most *CsDof* genes may mediate the regulation of light- or hormone-induced life processes. Moreover, several MYB binding sites including MBS, MBSI, MRE and CCAAT-box associated with drought, photoresponse and flavonoid biosynthesis were identified in many *CsDof* gene promoters, demonstrating that these CsDof proteins may interact with MYB TFs to involve in regulation of drought stress response, photoperiod and flavonoid biosynthesis. In addition, 62% of the *CsDof* genes contained cis-elements responsive to environmental stresses (TC-rich repeats and LTR) and 18% contained WUN-motif, suggesting that *CsDof* gene is likely to be involved in stress responses, such as low-temperature stress, wound reaction and so on. Interestingly, nine *CsDof* genes were identified to possess seed-specific regulatory elements (RY-element, CATGCA (TG)), including *CsDof 10*, *CsDof 12*, *CsDof 19*, *CsDof 32*, *CsDof 47*, *CsDof 51*, *CsDof 52*, *CsDof 55* and *CsDof 89*. Except for *CsDof 19* and *CsDof 47*, the remaining seven genes were expressed to different degrees during seed development (Fig. 6B). Notably, RY-element is an important target of the seed-specific TFs ABI3 and FUS3 [46], and ABI3 and FUS3 may interact with Dof members.

**Table 1:**
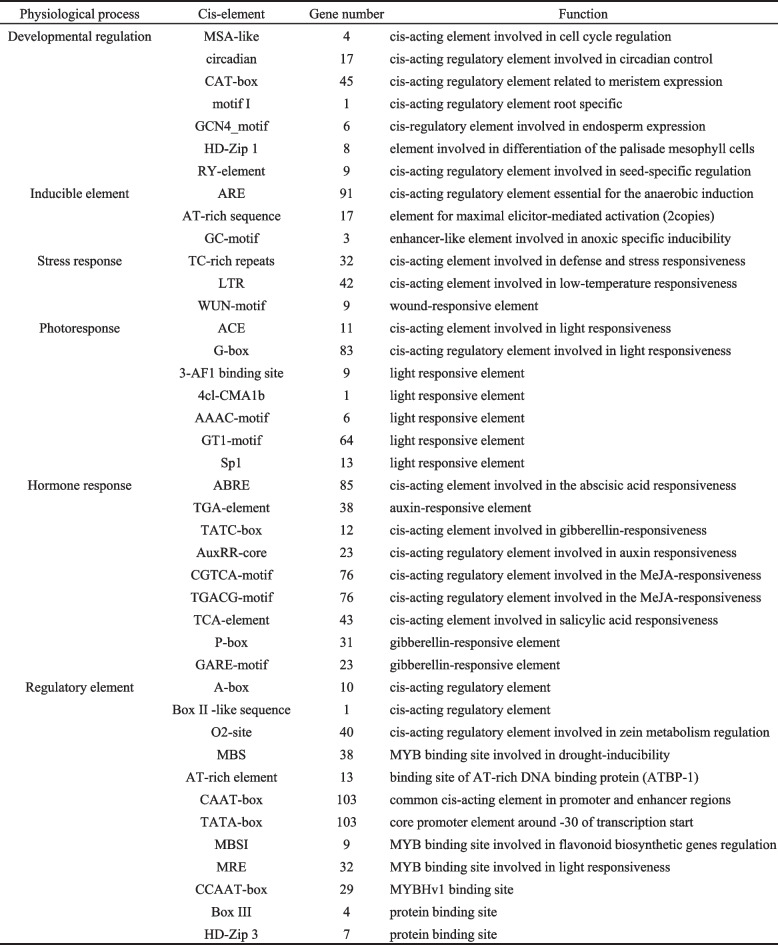
Cis-element regulatory elements in *CsDof* gene promoters.

### Expression patterns of *CsDof* genes in various *C. sativa* tissues

Analysis of gene expression pattern can provide important information to identify biological functions of an interest gene. To investigate the expression patterns of *CsDof* genes, the expression data of *CsDof* genes deposited in the publicly available database (FPKM values) were used to do such analysis in twelve tissues of *C. sativa* (Additional file 9), including root (R), stem (S), young leaf (YL), mature leaf (OL), flower (F), inflorescence (IF), early seed development (ESD), early-mid seed development (EMSD), mid-late seed development (LMSD), late seed development (LSD), germinating seed (GS) and cotyledons (C). As shown in Fig. 6A, a heat map of the expression profiles for *CsDof* genes demonstrated that most *CsDof* genes (up to 90%) were expressed in multiple organs despite their expression levels were different in these tissues, particularly higher expression in roots and stems. The tissue-specific expression pattern was also detected for a number of *CsDof* genes. For example, several *CsDof* genes were only highly expressed during seed germination, including *CsDof 12*, *CsDof 80* and *CsDof 91*. Moreover, during seed development (Fig. 6B), most *CsDof* genes showed specifically high expression in the early stage, including *CsDof 52*, *CsDof 57*, *CsDof 58* and *CsDof 71*. Whereas, small parts of *CsDof* genes such as *CsDof 9* and *CsDof 67* exhibited large expression levels in the middle and latest ages. These expressing analysis indicate that most *CsDof* genes may function constitutively during plant growth and development, but a number of them act differentially in various tissues/organs and different seed development stages.

### CsDof TFs regulate expressions of the genes related to lipid biosynthesis

**Fig. 7:**
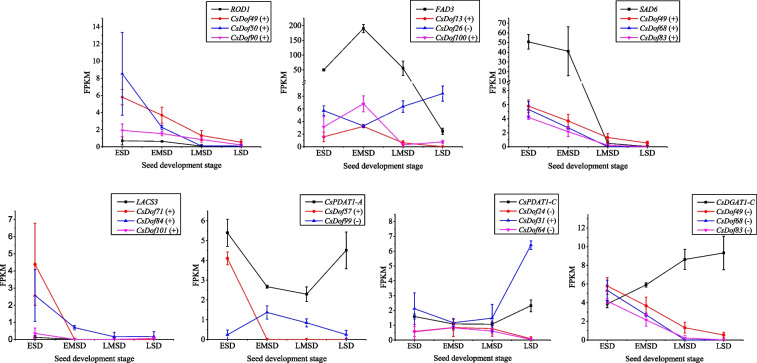
FPKM variation trend of genes at different seed developmental stages of *C. sativa*. FPKM variation trend of these *Dof* genes was extremely correlated with the FPKM variation trend of downstream genes. ESD, early seed development; EMSD, early-mid seed development; LMSD, late-mid seed development; LSD, late seed development. “+” stands for positive regulation, “–” stands for negative regulation. Data are means ± SE calculated from three biological replicates

The biosynthetic steps of lipids/oils in oilseeds mainly include the biosynthesis of fatty acid precursors, biosynthesis of fatty acid in plastids, and the assembly of triacylglycerol process in the endoplasmic reticulum. The Dof TF can specifically recognize and bind to the A/TAAAG (CTTTA/T) motif for activation and transcription regulation of the target genes [47]. In order to identify whether CsDof TFs regulate expressions of the lipid-related genes, the upstream 2000-bp promoter sequences derived from a number of lipid-related genes were taken to detect the cis-elements. As shown in Table 2, seven enzyme genes related to oil biosynthesis were found to contain the Dof-binding motif in their promoters. These seven lipid-related genes included three fatty acid desaturases (ROD1, FAD3 and SAD6), a long-chainacyl-CoA synthase (LACS3), and two acyltransferases (PDAT1 and DGAT1). Further analysis of expression patterns of these seven enzyme genes and several *CsDof* genes revealed that these enzyme gene expressions were extremely positive or negative correlated with the *CsDof’*s FPKM (Fig. 7). Thirteen pairs of enzyme genes and *CsDofs* were detected to be positive correlation, including *ROD1* with each of *CsDof 49*, *50* and *90*, *FAD3* with *CsDof 13* and *100*, *SAD6* with *CsDof 49*, *68* and *83*, *PDAT1-A* with *CsDof 57*, *PDAT1-C* with *CsDof 31*, *LACS3* with *CsDof 71*, *84* and *101*. However, seven pairs of enzyme genes and *CsDof*s were negative correlation, such as *FAD3* with *CsDof 26*, *PDAT1-A* with *CsDof 99*, *PDAT1-C* with *CsDof 24* and *64*, *DGAT1-A* with *CsDof 49*, *68* and *83*, respectively. These CsDof TFs may directly regulate the expression of these seven lipid-related enzymes in *C. sativa*, and consequently mediating oil biosynthesis and accumulation.

**Table 2:**
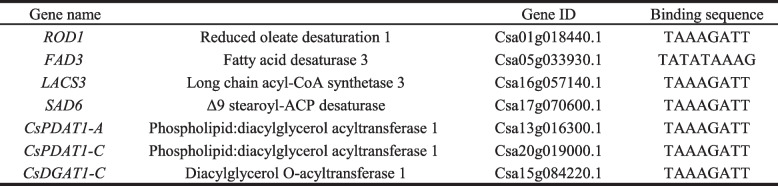
Prediction of the target gene of *CsDof* related to lipid metabolism.

### Several *CsDof* genes mediate plant responses to salt stress

**Fig. 8:**
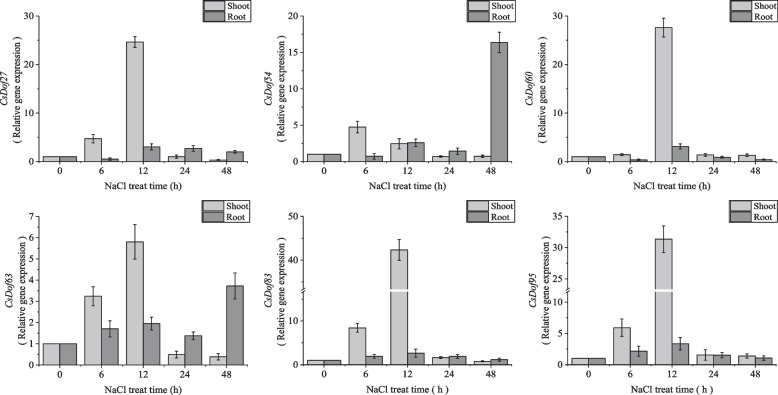
Expression profiles of six *CsDof* genes in roots and shoots under salt stress. Data are means ± SE calculated from three biological replicates. The relative gene expression levels were calculated relative to 0 h and using the 2^−ΔΔCt^ method

In order to identify the CsDof TFs that may mediate regulation of stress responses, six candidate *CsDof* genes were selected to examine their expression profiles in *C. sativa* seedlings treated by salt stress (150 mM NaCl). These six *CsDof* genes were screened based on the transcriptome data (FPKM values) derived from *C. sativa* seedlings under salt treatment (Additional file 10). Subsequently, the temporal expression profiles of the six *CsDof* genes (Fig. 8) were obtained in shoots and roots of *C. sativa* under salt treatment by quantitative RT-PCR (qRT-PCR). Overall, these six *CsDof* genes were more responsive in the shoots than in the roots. The relative expression of all six genes in the shoots tended to increase firstly and then decrease. Furthermore, with the exception of *CsDof 54*, which reached its maximum relative expression at 6 h after salt treatment, the relative expression of the other five genes (*CsDof 27*, *60*, *63*, *83* and *95*) all peaked at 12 h after salt treatment. In the roots, generally, the four genes (*CsDof 27*, *60*, *83* and *95*) showed similar expression trends to that in shoots, also reaching the maximum relative expression at 12 h after salt treatment, but the expression levels were much less than that in shoots. The other two genes (*CsDof 54* and *63*) showed an increase at 12 h and then a decrease at 24 h after slat treatment, but increased to the maximum relative expression at 48 h after salt treatment. These results suggest that the six *CsDof* genes may positively regulate plant response to salt stress, with higher effects in shoots than in roots.

## Discussion

### Characteristics and evolution among CsDof family members

As a class of plant-specific TFs, the Dof TF family has important functions in plant growth and development and metabolic pathways. The Dof TF has been identified in a variety of plants, such as *C. reinhardtii*, *Arabidopsis*, soybean and others. *C. sativa,* as an environmentally friendly oil crop rich in beneficial unsaturated fatty acids, has become an important target for commercial food and biofuel production. In this study, 103 CsDof proteins were identified for the first time from *C. sativa*, and they were divided into four groups and nine subgroups, similar to the grouping in *Arabidopsis* and rice [27, 29]. Group C has the largest number of *Dof* members, followed by groups B, D and A. *AtDof 2.5* and *AtDof 3.7* genes from group C are involved in the regulation of seed germination and *AtDof 3.4* derived from group D_2_ is involved in defense responses [28]. These laid the foundation for the mining of functional *Dof* genes in *C. sativa*. 103 CsDof proteins contain at least one C_2_-C_2_ type zinc finger domain, and the domains of each group were highly conserved. According to the constructed chromosome position of *CsDof* genes, all candidate *CsDof* genes present uneven distribution. Like other *Dof* genes in rice and *Arabidopsis*, most of *C. sativa Dof* genes had few introns (0–2) (Fig. 3C). The motif analysis showed that motif 1 was uniformly observed in all Dof proteins (Fig. 3B), similar to *Arabidopsis*, rice and tomato. This result indicated that the evolution of CsDof transcription factors was conserved in plant development.

Gene duplication is an important way for organisms to acquire new genes and create genetic novelty [48]. The plasticity of a genome or species to adapt to environmental changes is closely related to gene duplication [49–51]. It has made great contributions to the development and evolution of various organisms. In other words, gene duplication is the key force behind the expansion of gene families. In the present study, 82 segmental duplication events were detected for *CsDof* gene family, revealing that segmental duplication was the main force behind the expansion of the *CsDof* gene family. Selection pressure calculations showed that Ka/Ks of 98.80% duplicated gene pairs were less than 0.8, indicating that the *CsDof* gene family was mainly subject to purifying selection during evolution, and such positive selection may produce beneficial mutations for *C. sativa*. Similarly, in *B. napus*, segmental replication was also detected as the main force for Dof TF family expansion, and this family experienced purifying selection during its evolution [52].

Furthermore, syntenic analysis for Camelina and *Arabidopsis* or *B. napus* revealed a large number of syntenic gene pairs between *CsDofs* and *AtDofs* or *BnaDofs*. In comparison, larger numbers of genes were detected to be located on the syntenic locus in *Arabidopsis* and *C. sativa* chromosomes. Moreover, most of the pairwise orthologs genes were found between *Arabidopsis* and *C. sativa*. In view of this, the expansion of *Dof* gene family in *C. sativa* is later than *AtDof*s went through.

*C. sativa* is a representative species of a whole-genome triplication event relative to the cruciferous model *Arabidopsis* [53]. One gene in *Arabidopsis* corresponds to three homologous genes in the Camelina genome. Such triplication was also detected for *CsDof* genes in Camelina. 24 *Arabidopsis CsDof* genes were detected to have three syntenic copies in *C. sativa* genome (Additional file 5). For example, *Csa14g009010* (*CsDof 78*), *Csa17g011020* (*CsDof 90*) and *Csa03g011080* (*CsDof 10*) in Camelina were identified as the ortholog of *AT1G07640* (*AtDof 1.1*) in *Arabidopsis*. Additionally, another 7 *Arabidopsis AtDof* genes were detected to have 1 or 2 syntenic copies in Camelina. It should be noted that this triplication was not detected in Camelina for all of *Arabidopsis AtDof* genes [54]. In other words, some *Arabidopsis* genes had non homologous genes detected in Camelina. This may be due to gene loss or deletion. It was reported that chromosomes recombine as species undergo polyploidy events, and this process is often accompanied by the loss of large numbers of genes [55]. At the same time, gene loss could promote the evolution of species to a certain extent. Moreover, the intergenic collinearity can also be disrupted by gene loss. Extensive genetic segregation and genome reorganization weakened its similarity to the ancestral species [56].

### Interaction of CsDof TF with other factors

The Dof domain is considered as a bi-functional domain that combines DNA and interacts with proteins. In *Arabidopsis,* the interaction was found between Dof domain protein (OBP1) and bZIP protein related to stress response for the first time [57]. Another example is the interaction between maize Dof domain protein (PBF) and bZIP protein Opaque2 (O2), a genetically identified endosperm specifically expressed transcriptional regulator [58]. Interestingly, the seed-specific regulatory elements (RY-elements) were found in the upstream promoter region of a set of *CsDof* genes. It is noteworthy that this cis-element is an important binding target of ABI3 and FUS3 TFs. Therefore, it is possible that CsDof can interact with ABI3 and FUS3, playing a role in seed development and substance metabolism. In addition, MYB binding sites were largely distributed in a number of CsDof gene promoters, indicating that Dof TF may interact with MYB. In fact, it was proved that MYB interacts with Dof TFs [36]. MYB TFs are related to flavonoid biosynthesis, biotic and abiotic stress, and regulation of plant development. Dof and MYB TFs participate jointly in some physiological processes in plants through positive or negative regulation, including light response, metabolic regulation and stress response. Collectively, CsDof may interact with multiple TFs (e.g. ABI3, FUS3 and MYB) to participate in a variety of physiological processes, such as seed development, substance metabolism and stress response, in *C. sativa*.

### CsDof mediate lipid/oil biosynthesis in *C. sativa* seeds

TFs are emerging as key factors in metabolic regulation to facilitate the biosynthesis of various bioactive substances. By targeting master TFs, entire metabolic pathways can be upregulated, even if not all the influence factors are known [17]. For the regulation of TAG biosynthesis, several master TFs have been identified, including LEC1 [17], WRI1 [59] and FUS3 [19]. The targeted regulation of Dof TFs also led to improve lipid content [22, 39–41]. Our transcriptome analysis revealed that more than half of the genes were differentially expressed in various tissues of *C. sativa*, indicating that CsDof TFs may play importantly in plant growth and development despite of functional differences in different tissues. Some of *CsDof* genes were specifically expressed at different stages of seed development, and their expression patterns were consistent with the trend of lipids/oil accumulation in developing seeds. These CsDof TF members may mediate seed lipid/oil metabolism in *C. sativa*. To confirm this hypothesis, we examined the cis-elements distributed in the upstream promoter of several enzyme genes involved in lipid/oil biosynthesis, As expected, A/TAAAG (CTTTA/T) motif, the typical binding sits of Dof TFs [47], was detected in these lipid-related enzyme genes, such as *ROD1*, *FAD3*, *SAD6*, *LACS3*, *PDAT1* and *DGAT1*. Dof TFs can indeed regulate the transcriptional level of *LACS* through weak binding to this target elements [39, 40, 47]. Thus, a set of CsDof TFs may mediate the regulation of lipid/oil biosynthesis in Camelina developing seeds although the regulatory mechanism needs to be further elucidated.

### CsDof participate plant respond to a variety of abiotic stresses

TFs can specifically recognize cis-elements in gene promoter to activate or inhibit the spatiotemporal expression of the target genes. TFs, thereby, directly or indirectly display their functions. A large number of genes related to abiotic stress were examined by genome-wide analysis. So far, a number of TFs involved in stress response have been identified successively, including NAC [24], bZIP [21], WRKY [25], MYB [20] and more. In order to explore whether CsDofs are also involved in abiotic stress response, we conducted two experiments. Firstly, we identified a large number of cis-acting elements in 2000 bp upstream promoter region of the CsDof TF family members. Many cis-element identified are those sites related to abiotic stress (TC-rich repeats, LTR and WUN-motif) [60, 61], suggesting a role for CsDof TFs in plant stress response. Secondly, we confirmed that a number of *CsDof* genes can indeed respond differentially to salt stress signals in both roots and shoots. These results indicate that CsDof TFs are involved in stress regulation of *C. sativa*, perhaps not limited to salt stress. In supporting our results, numerous studies reported that Dof TFs play crucial roles in abiotic stress response and regulation. For example, after a variety of stress treatments included IAA, ABA, SA, Cr^3+^, Cd^2+^, NaCl, PEG, cold (4 °C) and heat (42 °C), the expression of *TaDof* genes in wheat were significantly up-regulated or down-regulated [42]. *Dof* gene also showed differential response pattern under drought stress treatment in *Camellia sinensis* [62]. In addition, rice *OsDof 15* was found to be a salt-responsive gene, which inhibited primary root growth after salt stress induction [63].

On the other hand, 98% of the promoters of *CsDof* genes also contain a large number of hormone response elements (ABRE, CGTCA-motif TGACG-motif and others), suggesting that *CsDof* genes possibly participate in stress regulatory through responding phytohormones. Phytohormones, such as abscisic acid (ABA), jasmonates (JA), salicylic acid (SA) and others, play a major role in mediating plant abiotic and biotic stress responses. For example, under drought stress, ABA can regulate stomatal closure to maintain cellular water balance [64]. Taken together, *CsDof* genes can positively respond to salt stress, and may play a positive function through the feedback of hormone signals.

In addition, CsDofs may mediate stress responses through regulating lipid/oil-related gene expressions. Plant abiotic stress tolerance is conferred by many interrelated mechanisms. Under abiotic stress such as cold stress and salt stress, lipid/oil biosynthesis in plant tissues was detected to be increased, accompanied by membrane lipid remodeling and changes in lipid profiles, which benefit the membrane integrity, and therefore, contribute to the maintenance of plant growth and development and the enhanced tolerance to stresses [65]. Our data showed that several *CsDof* genes (e.g. *CsDof 13, CsDof 49, CsDof 57* and *CsDof 71*) were highly co-expressed with lipid/oil biosynthesis-related enzyme genes (e.g. *FAD3, SAD6, ROD1, PDAT1-A* and *LACS3*). This indicates that some CsDof members may mediate lipid/oil biosynthesis by regulating the corresponding enzyme gene expressions, thus promoting stress tolerance of *C. sativa* plants. Further investigations need to verify this putative mechanism of CsDof-mediating lipid synthesis and stress responses. Lipid metabolism is closely involved in stress response. *GhDof1*-overexpressed cotton plant exhibited significantly higher salt and cold tolerance than the wild-type cotton, and the seed oil content in some transgenic cottons was significantly increased [66]. In the present study, CsDof 83 was detected to be involved in both the regulation of oil synthesis gene expressions and response to salt stress despite further verification is needed.

## Conclusions

In summary, 103 CsDof TF members were firstly identified from the genome of *C. sativa* in this study, and they were divided into four primary groups. Many orthologous *Dof* gene pairs were identified between every two genomes of *C. sativa*, *Arabidopsis* and *B. napus*, indicating a high synteny among the three genomes. Segmental duplication was the main force of *CsDof* gene family expansion. And almost all *CsDof* genes experienced a purifying selection during their evolution. More than half of *CsDof* genes were expressed in multiple tissues, with a number of them showing tissue-specific expression, suggesting that *CsDof* genes function differentially in different tissues of *C. sativa.* Notably, a number of CsDofs (CsDof 27, 54, 60, 63, 83 and 95) were detected to play crucial roles in response abiotic stress while a set of CsDofs (CsDof 13, 24, 26, 31, 49, 50, 57, 64, 68, 71, 83, 84, 90, 99, 100 and 101) may mediate lipid metabolism in *C. sativa* developing seeds. Overall, the present findings provide a theoretical basis for further studies on *Dof* gene functions in *C. sativa* and the mining of *Dof* genes mediating oil anabolism and abiotic stress responses.

## Materials and methods

### Sequence identification

The complete genome, protein and CDS sequence files for *C. sativa* were downloaded from Ensembl Plants and the NCBI database. 36 *Arabidopsis* Dof protein sequences were downloaded from the Tair database. Dof structural domain HMM (Hidden Markov Model) model (PF02701) was extracted from the EMBL-Pfam protein family database. The *Arabidopsis* Dofs were used as the queries to identify *C. sativa* Dof proteins using BLASTP. The Dof domain integrity of the resulting proteins was verified using SMART [67], EMBL-Pfam [68] and CD-Search [69]. Members of the *C. sativa* Dof TF family were identified, and namely as CsDof. The ProtParam from ExPasy online tools was used to calculate the physicochemical propertie of the identified CsDofs [70], including protein sequence length (SL), molecular weight (MW) and isoelectric point (pI).

### Multiple sequence alignment and phylogenetic analysis

Multiple sequence alignment of CsDof proteins was performed using ClustalX. Phylogenetic analysis of the reported AtDof (Additional file 6) with the identified CsDof proteins was carried out using MEGA7.0 [70]. A phylogenetic tree was constructed using the neighbour-joining method (NJ) with 1000 bootstrap replicates. All the identified CsDofs were classified into different groups according to the classification of AtDofs [27].

### Chromosomal localization, gene structure and motif analysis

The chromosomal positions of the *CsDof* genes were extracted from the genome annotation file (GFF3) and visualized by TBtools [45], followed by gene structure analysis. Conserved motif (Motif) analysis was performed using the online tool MEME [71]. Parameters were set as follows: the maximum number of motifs, 10; the minimum motif width, 6; and the maximum motif width, 50. A total of 10 conserved motifs were identified and the results were embellished using TBtools [45].

### Gene duplication and selection pressure analysis

The Multiple Collinearity Scan tool kit (MCScanX) in TBtools [45] was used to examine segmental duplication events in the genome of *C. sativa*, after which non-synonymous substitution (Ka) and synonymous substitution (Ks) values were calculated for the segmental duplication gene pairs. The gene duplications were dated using the formula *T = Ks/2r* [72]. The *r* is the rate of substitution, which was estimated as 8.22 × 10^− 9^ [53]. The synteny relation between each two genomes of the three closely-related species (*C. sativa* and *Arabidopsis*, and *B. napus*) was analyzed using MCScanX, and the mutual homologous gene pairs were counted. Genomic data for *B. napus* were downloaded from GenoScope [73].

### Analysis of cis-acting elements

The 2000 bp promoter region upstream of the 103 *CsDof* genes of *C. sativa* (Additional file 11) was analyzed using the PlantCARE database [74], while the sequence of the upstream promoter regions was extracted using TBtools.

### Expression profiling of CsDof genes in different *C. sativa* tissues

Transcriptomic data for *C. sativa* were obtained from a publicly available database (Additional file 9) [75]. A total of 12 tissues at different developmental stages were used for *CsDof* gene expression profiling, including Cotyledon (C), Germinating seed (GS), Inflorescence (IF), Flower (F), Early-mid seed development (EMSD), Early seed development (ESD), Late seed development (LSD), Late-mid seed development (LMSD), Mature leaf (OL), Root (R), Stem (S), Young leaf (YL). Expression profiles of *CsDof* genes were mapped using TBtools.

### Target gene prediction

To investigate whether CsDof TFs regulate expressions of the genes related to lipid/oil biosynthesis, partial sequences of genes involved in lipid synthesis were downloaded. The 2000 bp promoter sequences upstream of the seven relevant genes were extracted using TBtools (Additional file 12), followed by cis-acting element analysis in the PlantCARE database. The target gene screening was carried out using the Dof TF’s ability to specifically identify and bind the A/TAAAG (CTTTA/T) motif [47]. To further analyze the expression relationship between these seven genes and CsDof genes, we analyze the co-expression pattern of the downstream genes regulated by CsDof transcription factors, according to the transcriptional expression (FPKM) data of genes at different seed development stages [75].

### Plant material and salt stress treatment

To investigate the response of *CsDof*s under salt stress condition in *C. sativa* seedlings, we carried out a response pattern analysis of the selected genes from publicly available databases (Additional file 10) [44]. *C. sativa* cultivar “SC-N1”, was commercially planted in the Taigu district, Shanxi province, China (E112.32°, N37.26°) for several years. This cultivar was selected as the plant material for the experiments. The plants were grown at 23 °C with a photoperiod of 16-h light/8-h dark and were treated with 150 mM NaCl solution on the three-week seedlings [70]. Samples were taken at 0, 6, 12, 24 and 48 h after the salt stress treatment. The seedling samples were immediately frozen in liquid N_2_ and stored at − 80 °C for follow-up RNA extraction. TaKaRa RNAiso Plus kit was used for RNA extraction. Reagents for reverse transcription and qRT-PCR were purchased from TransGen. The *β-actin* gene of *C. sativa* was used as an internal reference gene. All the primer pairs used for qRT-PCR analysis are shown in Additional file 13. Three biological replicates were performed for each of these experiments.

## Supplementary Information

**Additional file 1: Table S1.** Detailed information of all identified *C. sativa* Dof proteins.

**Additional file 2: Fig. S1.** Details of 10 Motifs of CsDofs.

**Additional file 3: Fig. S2.** Chromosomal distribution of 103 *CsDof* genes.

**Additional file 4: Table S2.** Segmental duplication of *CsDof* among *C. sativa* chromosomes.

**Additional file 5: Table S3.** All gene pairs of *C. sativa* and *A. thaliana*.

**Additional file 6: Table S4.** All accession numbers of sequences used in this article.

**Additional file 7: Table S5.** All gene pairs of *C. sativa* and *B. napus*.

**Additional file 8: Table S6.** Details of the cis-elements identified in *CsDof* gene family.

**Additional file 9: Table S7.** Expression levels (FPKM values) of 103 *CsDof* genes across 12 different tissue samples.

**Additional file 10: Table S8.** Expression levels (FPKM values) of 103 *CsDof* genes in roots and shoots under salt stress.

**Additional file 11: Table S9.** The 2000 bp promoter region upstream of the *CsDof* genes.

**Additional file 12: Table S10.** The 2000 bp promoter region upstream of the *CsDof*`s target genes.

**Additional file 13: Table S11.** The primer sequences of 6 *CsDof* genes used for qRT-PCR.

**Additional file 14: Fig. S3.** The total lipid content in leaves of wild type (WT) and CsDof13-transgenic lines (T1 tobacco plants). Fig. S4. The Malondialdehyde (MDA) content of wild type (WT) and CsDof27-transgenic lines (T1 tobacco plants) before and after salt stress.

## Data Availability

All data generated or analyzed in this study are included in this published article and its Supplementary Material. The datasets generated and analyzed during the current study are available from the corresponding author on reasonable request. The analysis websites used in this study are as follows: Ensembl Plants (https://plants.ensembl.org/Camelina_sativa/Info/Index), NCBI (https://www.ncbi.nlm.nih.gov/), Tair database (https://www.arabidopsis.org/index.jsp), the EMBL-Pfam protein family database (https://pfam.xfam.org/family/PF02701), SMART (http://smart.embl-heidelberg.de/), Batch CD-Search (https://www.ncbi.nlm.nih.gov/Structure/bwrpsb/bwrpsb.cgi), ExPasy (http://web.expasy.org/protparam/), MEME (https://meme-suite.org/meme/tools/meme), GenoScope (https://www.genoscope.cns.fr/brassicanapus/), and PlantCARE (http://bioinformatics.psb.ugent.be/webtools/plantcare/html/).
