# Supplementary material for: Genome-wide identification and functional analysis of Dof transcription factor family in Camelina sativa
Source: BMC Genomics. 2022 Dec 8;23:812. doi: 10.1186/s12864-022-09056-9 (PMC9730592; doi:10.1186/s12864-022-09056-9)
Supplement: Supplementary file 1 — Additional file 1: Table S1. Detailed information of all identified C. sativa Dof proteins. [file 12864_2022_9056_MOESM1_ESM.pdf]

**Table S1. Detailed information of all identified *C. sativa* Dof proteins.**

| Name | Gene ID | Chr. | Start | End | SL | MW(Da) | pI | Group |
|---|---|---|---|---|---|---|---|---|
| CsDof1 | Csa00734s020.1 | Scaffold00734 | 1511 | 2239 | 243 | 27544.9 | 4.47 | $C_{2.2}$ |
| CsDof2 | Csa01g024360.1 | 1 | 10042478 | 10044111 | 201 | 22163.6 | 9.41 | A |
| CsDof3 | Csa02g002370.1 | 2 | 812220 | 814123 | 297 | 33428.7 | 7.32 | $C_{2.1}$ |
| CsDof4 | Csa02g067130.1 | 2 | 25104024 | 25105348 | 260 | 28312.1 | 8.76 | $C_1$ |
| CsDof5 | Csa02g067810.1 | 2 | 25414668 | 25416314 | 278 | 30027.7 | 6.5 | A |
| CsDof6 | Csa02g070560.1 | 2 | 26283466 | 26285344 | 301 | 33565.5 | 8.81 | $D_1$ |
| CsDof7 | Csa02g071140.1 | 2 | 26595217 | 26596461 | 312 | 33738 | 8.85 | $C_1$ |
| CsDof8 | Csa02g073390.1 | 2 | 27544030 | 27545368 | 322 | 35137.8 | 9.03 | $B_2$ |
| CsDof9 | Csa02g075810.1 | 2 | 28627949 | 28629196 | 227 | 24045.4 | 9.26 | $D_2$ |
| CsDof10 | Csa03g011080.1 | 3 | 3158381 | 3160557 | 338 | 36291.9 | 9.6 | $B_1$ |
| CsDof11 | Csa03g025140.1 | 3 | 9700513 | 9701298 | 262 | 29583.1 | 4.47 | $C_{2.2}$ |
| CsDof12 | Csa03g028730.1 | 3 | 11573172 | 11574397 | 373 | 41675.5 | 7.13 | $D_1$ |
| CsDof13 | Csa03g032300.1 | 3 | 13562484 | 13563246 | 176 | 19374.8 | 9.53 | $D_1$ |
| CsDof14 | Csa03g051350.1 | 3 | 21808702 | 21809584 | 218 | 22745.7 | 8.2 | $D_2$ |
| CsDof15 | Csa03g058660.1 | 3 | 25496746 | 25497919 | 220 | 23861.3 | 9.17 | A |
| CsDof16 | Csa04g026300.1 | 4 | 12854002 | 12855214 | 244 | 26472.3 | 9.33 | $C_1$ |
| CsDof17 | Csa04g030380.1 | 4 | 14525989 | 14528110 | 459 | 51013.4 | 7.93 | $D_1$ |
| CsDof18 | Csa04g036060.1 | 4 | 17170621 | 17171838 | 316 | 33442 | 9.48 | $D_2$ |
| CsDof19 | Csa04g038300.1 | 4 | 18491878 | 18492627 | 250 | 27124.7 | 5.04 | $C_{2.2}$ |
| CsDof20 | Csa04g041620.1 | 4 | 20303062 | 20305318 | 380 | 41888.8 | 9.17 | $B_1$ |
| CsDof21 | Csa04g051210.1 | 4 | 24247602 | 24249595 | 323 | 34663.6 | 9.1 | $B_1$ |
| CsDof22 | Csa04g065700.1 | 4 | 29412600 | 29414878 | 371 | 40477.4 | 9.34 | $C_{2.1}$ |
| CsDof23 | Csa05g002560.1 | 5 | 747122 | 749769 | 375 | 41135 | 9.14 | $C_{2.1}$ |
| CsDof24 | Csa05g016230.1 | 5 | 6030856 | 6032684 | 323 | 34663.6 | 9.1 | $B_1$ |
| CsDof25 | Csa05g035600.1 | 5 | 13669977 | 13672948 | 345 | 37041.4 | 9.65 | $B_1$ |
| CsDof26 | Csa05g038090.1 | 5 | 14240921 | 14242753 | 288 | 31402.4 | 8.28 | $C_1$ |
| CsDof27 | Csa05g086300.1 | 5 | 31136601 | 31138757 | 402 | 44393.8 | 9.34 | $D_1$ |
| CsDof28 | Csa05g094020.1 | 5 | 34030314 | 34032624 | 297 | 32895.3 | 9.71 | $C_{2.1}$ |
| CsDof29 | Csa06g019350.1 | 6 | 10364886 | 10366022 | 245 | 26643.5 | 9.33 | $C_1$ |
| CsDof30 | Csa06g021150.1 | 6 | 11744399 | 11746425 | 459 | 50866.2 | 8.06 | $D_1$ |

| Name | Gene ID | Chr. | Start | End | SL | MW(Da) | pI | Group |
|---|---|---|---|---|---|---|---|---|
| CsDof31 | Csa06g024450.1 | 6 | 13954375 | 13955701 | 255 | 26431.1 | 8.33 | $D_2$ |
| CsDof32 | Csa06g026770.1 | 6 | 15180762 | 15181517 | 252 | 27324.9 | 5.25 | $C_{2.2}$ |
| CsDof33 | Csa06g029980.1 | 6 | 16816184 | 16818380 | 359 | 39124.5 | 8.77 | $B_1$ |
| CsDof34 | Csa06g040340.1 | 6 | 20430747 | 20432478 | 325 | 34859.8 | 9.1 | $B_1$ |
| CsDof35 | Csa07g002770.1 | 7 | 898561 | 901037 | 297 | 32911.3 | 9.71 | $C_{2.1}$ |
| CsDof36 | Csa07g007240.1 | 7 | 2776067 | 2777245 | 174 | 19240.9 | 9.95 | $D_1$ |
| CsDof37 | Csa07g015680.1 | 7 | 7361190 | 7363778 | 346 | 36910.2 | 9.81 | $B_1$ |
| CsDof38 | Csa07g016040.1 | 7 | 7763652 | 7765348 | 346 | 38057.2 | 8.04 | $C_1$ |
| CsDof39 | Csa07g036310.1 | 7 | 19629890 | 19632718 | 457 | 49871.7 | 9.14 | $D_1$ |
| CsDof40 | Csa07g036310.2 | 7 | 19629890 | 19632718 | 404 | 44387.8 | 9.34 | $D_1$ |
| CsDof41 | Csa07g066190.1 | 7 | 33600335 | 33602555 | 337 | 36330.6 | 8.48 | $C_{2.1}$ |
| CsDof42 | Csa08g053300.1 | 8 | 22789191 | 22790319 | 298 | 33645.8 | 7.05 | $C_{2.1}$ |
| CsDof43 | Csa08g062200.1 | 8 | 27021304 | 27023299 | 398 | 42183.1 | 9.18 | $B_1$ |
| CsDof44 | Csa09g039660.1 | 9 | 14874134 | 14875566 | 244 | 26569.4 | 9.33 | $C_1$ |
| CsDof45 | Csa09g047820.1 | 9 | 17272901 | 17274891 | 456 | 50656.9 | 7.93 | $D_1$ |
| CsDof46 | Csa09g053360.1 | 9 | 20946925 | 20947683 | 253 | 26221.9 | 8.5 | $D_2$ |
| CsDof47 | Csa09g059550.1 | 9 | 22556481 | 22557230 | 250 | 27159.7 | 4.89 | $C_{2.2}$ |
| CsDof48 | Csa09g067350.1 | 9 | 25203256 | 25205551 | 358 | 38969.3 | 8.56 | $B_1$ |
| CsDof49 | Csa09g099480.1 | 9 | 38038132 | 38039862 | 342 | 36543.8 | 7.63 | $C_{2.1}$ |
| CsDof50 | Csa10g003120.1 | 10 | 993796 | 994641 | 236 | 26052.1 | 9.88 | $B_2$ |
| CsDof51 | Csa10g018980.1 | 10 | 8034126 | 8036354 | 355 | 38214.6 | 7.84 | $C_{2.1}$ |
| CsDof52 | Csa10g022390.1 | 10 | 9945469 | 9946308 | 263 | 29454.6 | 8.74 | $C_3$ |
| CsDof53 | Csa10g022470.1 | 10 | 9975446 | 9976503 | 212 | 23502.9 | 9.04 | $C_3$ |
| CsDof54 | Csa10g046910.1 | 10 | 22637181 | 22639673 | 461 | 50017.2 | 5.27 | $D_1$ |
| CsDof55 | Csa11g003480.1 | 11 | 1150206 | 1151031 | 250 | 27530.7 | 10.06 | $B_2$ |
| CsDof56 | Csa11g020730.1 | 11 | 9427399 | 9430227 | 587 | 63056.4 | 7.2 | $C_{2.1}$ |
| CsDof57 | Csa11g025410.1 | 11 | 11790424 | 11791206 | 261 | 29308.3 | 8.93 | $C_3$ |
| CsDof58 | Csa11g025430.1 | 11 | 11804952 | 11805737 | 262 | 29328.2 | 8.21 | $C_3$ |
| CsDof59 | Csa11g025480.1 | 11 | 11834449 | 11835491 | 247 | 27455.3 | 8.07 | $C_3$ |
| CsDof60 | Csa11g055680.1 | 11 | 26661088 | 26663396 | 461 | 50032.9 | 5.01 | $D_1$ |
| CsDof61 | Csa11g094130.1 | 11 | 45729174 | 45730543 | 265 | 28818.7 | 8.57 | $C_1$ |

| Name | Gene ID | Chr. | Start | End | SL | MW(Da) | pI | Group |
|---|---|---|---|---|---|---|---|---|
| CsDof62 | Csa11g094710.1 | 11 | 46040949 | 46042851 | 308 | 32806.7 | 7.05 | A |
| CsDof63 | Csa11g099290.1 | 11 | 46879042 | 46880808 | 301 | 33534.5 | 8.71 | $D_1$ |
| CsDof64 | Csa11g099790.1 | 11 | 47150078 | 47151961 | 422 | 45252 | 8.54 | $C_1$ |
| CsDof65 | Csa11g100300.1 | 11 | 47398410 | 47404243 | 447 | 46561.5 | 6.76 | $C_1$ |
| CsDof66 | Csa11g102460.1 | 11 | 48403866 | 48405309 | 320 | 35003.6 | 9.03 | $B_2$ |
| CsDof67 | Csa11g103980.1 | 11 | 49196591 | 49197484 | 226 | 23886.2 | 9.26 | $D_2$ |
| CsDof68 | Csa12g003340.1 | 12 | 1081406 | 1082311 | 245 | 27261.5 | 9.63 | $B_2$ |
| CsDof69 | Csa12g030340.1 | 12 | 9216706 | 9218934 | 355 | 38241.7 | 7.84 | $C_{2.1}$ |
| CsDof70 | Csa12g037400.1 | 12 | 12490613 | 12494580 | 548 | 61181.4 | 6.69 | $C_3$ |
| CsDof71 | Csa12g037460.1 | 12 | 12548694 | 12549507 | 262 | 29293.2 | 8.55 | $C_3$ |
| CsDof72 | Csa12g037480.1 | 12 | 12568810 | 12569798 | 255 | 28491.3 | 8.13 | $C_3$ |
| CsDof73 | Csa12g037530.1 | 12 | 12587269 | 12588117 | 210 | 23226.7 | 9.01 | $C_3$ |
| CsDof74 | Csa12g042020.1 | 12 | 13579393 | 13580178 | 262 | 29262.3 | 8.81 | $C_3$ |
| CsDof75 | Csa12g081790.1 | 12 | 29677128 | 29679440 | 532 | 58409.7 | 7.34 | $D_1$ |
| CsDof76 | Csa13g002360.1 | 13 | 653090 | 655090 | 401 | 42408.4 | 9.36 | $B_1$ |
| CsDof77 | Csa13g056100.1 | 13 | 23317719 | 23319726 | 298 | 33497.7 | 7.18 | $C_{2.1}$ |
| CsDof78 | Csa14g009010.1 | 14 | 3182958 | 3185106 | 338 | 36288.9 | 9.6 | $B_1$ |
| CsDof79 | Csa14g026580.1 | 14 | 10410267 | 10411430 | 262 | 29542 | 4.53 | $C_{2.2}$ |
| CsDof80 | Csa14g031920.1 | 14 | 12388826 | 12390053 | 373 | 41563.4 | 6.67 | $D_1$ |
| CsDof81 | Csa14g036700.1 | 14 | 14705836 | 14706892 | 176 | 19402.8 | 9.53 | $D_1$ |
| CsDof82 | Csa14g051530.1 | 14 | 23704179 | 23705150 | 218 | 22668.6 | 7.85 | $D_2$ |
| CsDof83 | Csa14g059960.1 | 14 | 27993778 | 27994881 | 220 | 24150.7 | 9.21 | A |
| CsDof84 | Csa15g031650.1 | 15 | 12308092 | 12308691 | 200 | 22080.6 | 9.41 | A |
| CsDof85 | Csa16g003010.1 | 16 | 1083925 | 1086375 | 285 | 31472.7 | 9.86 | $C_{2.1}$ |
| CsDof86 | Csa16g007040.1 | 16 | 2722225 | 2723024 | 174 | 19243.8 | 9.77 | $D_1$ |
| CsDof87 | Csa16g016240.1 | 16 | 6557535 | 6562202 | 350 | 37467.8 | 9.76 | $B_1$ |
| CsDof88 | Csa16g016640.1 | 16 | 6947869 | 6950240 | 225 | 24485.8 | 8.79 | $C_1$ |
| CsDof89 | Csa16g031000.1 | 16 | 16450510 | 16452381 | 433 | 47841 | 9.32 | $D_1$ |
| CsDof90 | Csa17g011020.1 | 17 | 3043278 | 3045269 | 342 | 36732.3 | 9.6 | $B_1$ |
| CsDof91 | Csa17g027260.1 | 17 | 10021615 | 10023000 | 359 | 40773.6 | 6.95 | $C_{2.2}$ |
| CsDof92 | Csa17g034060.1 | 17 | 12361528 | 12363002 | 371 | 41525.4 | 7.13 | $D_1$ |

| Name | Gene ID | Chr. | Start | End | SL | MW(Da) | pI | Group |
|---|---|---|---|---|---|---|---|---|
| CsDof93 | Csa17g041880.1 | 17 | 14836571 | 14837612 | 176 | 19388.8 | 9.52 | $D_1$ |
| CsDof94 | Csa17g073130.1 | 17 | 26338345 | 26339380 | 315 | 32285.9 | 5.11 | $D_2$ |
| CsDof95 | Csa17g090650.1 | 17 | 31096635 | 31098146 | 219 | 24071.6 | 8.94 | A |
| CsDof96 | Csa18g033540.1 | 18 | 16930288 | 16932921 | 245 | 26760.5 | 9.5 | $C_1$ |
| CsDof97 | Csa18g034230.1 | 18 | 17313844 | 17315504 | 308 | 32767.6 | 6.99 | A |
| CsDof98 | Csa18g035890.1 | 18 | 18171584 | 18173438 | 301 | 33687.8 | 8.86 | $D_1$ |
| CsDof99 | Csa18g036390.1 | 18 | 18423753 | 18425674 | 432 | 46419.7 | 8.9 | $C_1$ |
| CsDof100 | Csa18g039010.1 | 18 | 19620276 | 19621697 | 320 | 34976.6 | 9.03 | $B_2$ |
| CsDof101 | Csa18g040580.1 | 18 | 20377061 | 20379652 | 325 | 34089.2 | 8.39 | $D_2$ |
| CsDof102 | Csa19g029310.1 | 19 | 11438070 | 11439465 | 309 | 34059.1 | 9.3 | A |
| CsDof103 | Csa20g002520.1 | 20 | 715040 | 717031 | 401 | 42398.3 | 9.36 | $B_1$ |
