# Supplementary material for: Genome-wide identification and functional analysis of Dof transcription factor family in Camelina sativa
Source: BMC Genomics. 2022 Dec 8;23:812. doi: 10.1186/s12864-022-09056-9 (PMC9730592; doi:10.1186/s12864-022-09056-9)
Supplement: Supplementary file 2 — Additional file 2: Fig. S1. Details of 10 Motifs of CsDofs. [file 12864_2022_9056_MOESM2_ESM.pdf]

Fig. S1 Details of 10 Motifs of CsDofs**.**

Domain	E-value	Sites	Width	Consensus sequence
Motif 1	2.9e-4978	103	50	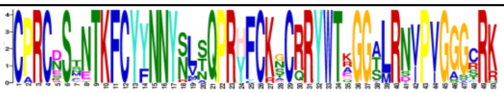
Motif 2	1.6e-185	10	33	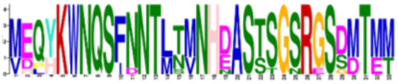
Motif 3	8.5e-246	10	50	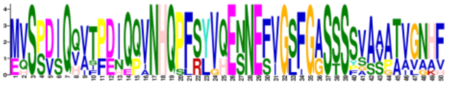
Motif 4	2.9e-219	9	41	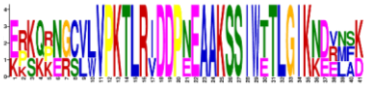
Motif 5	3.2e-214	7	50	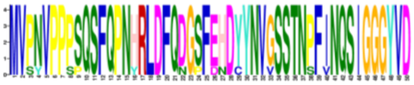
Motif 6	1.7e-161	21	21	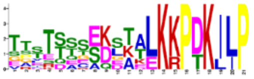
Motif 7	2.9e-161	45	11	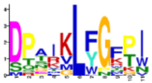
Motif 8	4.9e-184	15	29	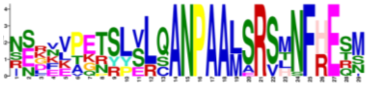
Motif 9	1.1e-154	60	21	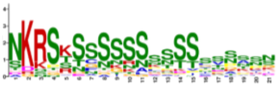
Motif 10	2.1e-144	18	21	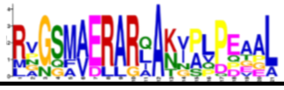
