# Supplementary material for: Genome-wide identification and functional analysis of Dof transcription factor family in Camelina sativa
Source: BMC Genomics. 2022 Dec 8;23:812. doi: 10.1186/s12864-022-09056-9 (PMC9730592; doi:10.1186/s12864-022-09056-9)
Supplement: Supplementary file 4 — Additional file 4: Table S2. Segmental duplication of CsDof among C. sativa chromosomes. [file 12864_2022_9056_MOESM4_ESM.pdf]

Table S2 Duplication gene pair of *CsDof*

Duplication gene pair		Ka	Ks	Ka/Ks	Divergence time (MYA)
Csa04g051210	Csa05g016230	0.003	0.125	0.022	7.60
Csa14g036700	Csa03g032300	0.003	0.093	0.027	5.66
Csa04g051210	Csa06g040340	0.004	0.09	0.045	5.47
Csa17g041880	Csa03g032300	0.008	0.121	0.063	7.36
Csa11g099790	Csa02g071140	0.008	0.098	0.079	5.96
Csa05g016230	Csa06g040340	0.007	0.085	0.08	5.17
Csa04g038300	Csa06g026770	0.006	0.075	0.083	4.56
Csa04g030380	Csa09g047820	0.012	0.144	0.086	8.76
Csa10g018980	Csa11g020730	0.006	0.071	0.087	4.32
Csa11g094710	Csa18g034230	0.006	0.053	0.109	3.22
Csa05g035600	Csa07g015680	0.006	0.058	0.111	3.53
Csa11g102460	Csa02g073390	0.007	0.062	0.112	3.77
Csa17g073130	Csa03g051350	0.002	0.018	0.114	1.09
Csa11g020730	Csa12g030340	0.009	0.075	0.114	4.56
Csa11g094130	Csa02g067130	0.012	0.098	0.12	5.96
Csa18g039010	Csa02g073390	0.007	0.057	0.121	3.47
Csa10g046910	Csa12g081790	0.013	0.11	0.121	6.69
Csa20g002520	Csa08g062200	0.002	0.018	0.124	1.09
Csa18g035890	Csa02g070560	0.012	0.092	0.126	5.60
Csa11g055680	Csa12g081790	0.016	0.112	0.141	6.81
Csa06g021150	Csa09g047820	0.019	0.136	0.141	8.27
Csa16g016640	Csa07g016040	0.006	0.041	0.142	2.49
Csa15g031650	Csa19g029310	0.002	0.015	0.147	0.91
Csa18g036390	Csa02g071140	0.013	0.085	0.15	5.17
Csa13g056100	Csa08g053300	0.007	0.048	0.151	2.92
Csa14g009010	Csa03g011080	0.004	0.026	0.152	1.58
Csa04g065700	Csa05g094020	0.177	1.16	0.153	70.56
Csa14g051530	Csa03g051350	0.013	0.084	0.153	5.11
Csa14g026580	Csa03g025140	0.02	0.129	0.155	7.85
Csa04g065700	Csa05g002560	0.014	0.079	0.171	4.81
Csa04g030380	Csa06g021150	0.019	0.103	0.185	6.27
Csa13g002360	Csa08g062200	0.003	0.018	0.186	1.09
Csa05g094020	Csa07g002770	0.007	0.037	0.195	2.25
Csa04g041620	Csa06g029980	0.017	0.088	0.197	5.35
Csa04g026300	Csa09g039660	0.018	0.089	0.205	5.41

Duplication gene pair		Ka	Ks	Ka/Ks	Divergence time (MYA)
Csa01g024360	Csa15g031650	0.013	0.054	0.246	3.28
Csa11g094130	Csa04g026300	0.195	0.786	0.249	47.81
Csa06g019350	Csa09g039660	0.024	0.095	0.249	5.78
Csa11g099290	Csa18g035890	0.017	0.069	0.252	4.20
Csa16g016240	Csa07g015680	0.011	0.043	0.265	2.62
Csa04g038300	Csa09g059550	0.018	0.066	0.271	4.01
Csa05g038090	Csa07g016040	0.011	0.039	0.272	2.37
Csa06g026770	Csa09g059550	0.018	0.066	0.272	4.01
Csa17g027260	Csa03g025140	0.017	0.062	0.275	3.77
Csa11g003480	Csa12g003340	0.029	0.1	0.286	6.08
Csa10g046910	Csa11g055680	0.014	0.049	0.29	2.98
Csa18g040580	Csa06g024450	0.28	0.963	0.291	58.58
Csa11g103980	Csa09g053360	0.269	0.914	0.294	55.60
Csa10g003120	Csa12g003340	0.034	0.114	0.298	6.93
Csa18g033540	Csa02g067130	0.036	0.114	0.315	6.93
Csa10g003120	Csa11g003480	0.019	0.059	0.32	3.59
Csa05g086300	Csa07g036310	0.027	0.08	0.331	4.87
Csa03g028730	Csa07g036310	0.285	0.834	0.342	50.73
Csa11g025410	Csa12g037460	0.041	0.117	0.348	7.12
Csa10g022390	Csa12g037460	0.039	0.109	0.357	6.63
Csa14g031920	Csa07g036310	0.306	0.853	0.359	51.89
Csa02g002370	Csa08g053300	0.018	0.051	0.36	3.10
Csa17g034060	Csa07g036310	0.282	0.781	0.361	47.51
Csa01g024360	Csa19g029310	0.011	0.03	0.365	1.82
Csa06g024450	Csa09g053360	0.018	0.049	0.37	2.98
Csa11g094130	Csa18g033540	0.042	0.11	0.384	6.69
Csa04g041620	Csa09g067350	0.024	0.056	0.42	3.41
Csa18g034230	Csa02g067810	0.024	0.055	0.431	3.35
Csa17g090650	Csa03g058660	0.025	0.05	0.496	3.04
Csa04g026300	Csa06g019350	0.009	0.018	0.502	1.09
Csa11g094710	Csa02g067810	0.023	0.041	0.556	2.49
Csa10g022390	Csa11g025410	0.03	0.049	0.621	2.98
Csa04g036060	Csa06g024450	0.027	0.044	0.628	2.68
Csa07g066190	Csa09g099480	0.066	0.1	0.661	6.08
Csa04g036060	Csa09g053360	0.022	0.032	0.671	1.95

Duplication gene pair		Ka	Ks	Ka/Ks	Divergence time (MYA)	Duplication gene pair		Ka	Ks	Ka/Ks	Divergence time (MYA)
Csa06g029980	Csa09g067350	0.013	0.065	0.209	3.95	Csa16g031000	Csa07g036310	0.111	0.163	0.683	9.91
Csa17g011020	Csa03g011080	0.007	0.03	0.216	1.82	Csa10g022470	Csa11g025480	0.042	0.06	0.693	3.65
Csa11g099290	Csa02g070560	0.017	0.08	0.217	4.87	Csa14g059960	Csa03g058660	0.027	0.035	0.767	2.13
Csa16g003010	Csa07g002770	0.006	0.027	0.223	1.64	Csa16g007040	Csa07g007240	0.017	0.021	0.789	1.28
Csa10g018980	Csa12g030340	0.005	0.021	0.23	1.28	Csa11g025480	Csa12g037530	0.035	0.044	0.792	2.68
Csa11g094130	Csa06g019350	0.202	0.83	0.243	50.49	Csa10g022470	Csa12g037530	0.03	0.028	1.061	1.70
