# Supplementary material for: Genome-wide identification and functional analysis of Dof transcription factor family in Camelina sativa
Source: BMC Genomics. 2022 Dec 8;23:812. doi: 10.1186/s12864-022-09056-9 (PMC9730592; doi:10.1186/s12864-022-09056-9)
Supplement: Supplementary file 5 — Additional file 5: Table S3. All gene pairs of C. sativa and A. thaliana. [file 12864_2022_9056_MOESM5_ESM.pdf]

Table S3. All gene pairs of *C. sativa* and *A. thaliana*

C. sativa	*A. thaliana*
Csa14g009010	AT1G07640
Csa17g011020	AT1G07640
Csa03g011080	AT1G07640
Csa14g026580	AT1G21340
Csa17g027260	AT1G21340
Csa03g025140	AT1G21340
Csa14g031920	AT1G26790
Csa17g034060	AT1G26790
Csa03g028730	AT1G26790
Csa14g036700	AT1G29160
Csa17g041880	AT1G29160
Csa03g032300	AT1G29160
Csa03g058660	AT1G51700
Csa09g099480	AT1G64620
Csa16g031000	AT1G69570
Csa05g086300	AT1G69570
Csa07g036310	AT1G69570
Csa05g038090	AT2G28510
Csa07g016040	AT2G28510
Csa16g016240	AT2G28810
Csa05g035600	AT2G28810
Csa07g015680	AT2G28810
Csa16g007040	AT2G34140
Csa07g007240	AT2G34140
Csa04g051210	AT2G37590
Csa05g016230	AT2G37590
Csa06g040340	AT2G37590
Csa04g065700	AT2G46590
Csa05g002560	AT2G46590
Csa01g024360	AT3G21270
Csa15g031650	AT3G21270
Csa19g029310	AT3G21270
Csa04g026300	AT3G45610
Csa06g019350	AT3G45610
Csa09g039660	AT3G45610
Csa04g030380	AT3G47500
Csa06g021150	AT3G47500
Csa09g047820	AT3G47500
Csa04g036060	AT3G50410
Csa06g024450	AT3G50410
Csa09g053360	AT3G50410
Csa04g038300	AT3G52440
Csa06g026770	AT3G52440
Csa09g059550	AT3G52440
Csa04g041620	AT3G55370
Csa06g029980	AT3G55370
Csa09g067350	AT3G55370
Csa16g003010	AT3G61850
Csa05g094020	AT3G61850
Csa07g002770	AT3G61850
Csa13g056100	AT4G00940

C. sativa	*A. thaliana*
Csa02g002370	AT4G00940
Csa08g053300	AT4G00940
Csa10g022390	AT4G21080
Csa11g025480	AT4G21080
Csa12g037400	AT4G21080
Csa10g018980	AT4G24060
Csa11g020730	AT4G24060
Csa12g030340	AT4G24060
Csa11g003480	AT4G38000
Csa12g003340	AT4G38000
Csa13g002360	AT5G02460
Csa20g002520	AT5G02460
Csa08g062200	AT5G02460
Csa10g046910	AT5G39660
Csa11g055680	AT5G39660
Csa12g081790	AT5G39660
Csa11g094130	AT5G60200
Csa18g033540	AT5G60200
Csa02g067130	AT5G60200
Csa11g094710	AT5G60850
Csa18g034230	AT5G60850
Csa02g067810	AT5G60850
Csa11g099290	AT5G62430
Csa18g035890	AT5G62430
Csa02g070560	AT5G62430
Csa11g099790	AT5G62940
Csa18g036390	AT5G62940
Csa02g071140	AT5G62940
Csa11g102460	AT5G65590
Csa18g039010	AT5G65590
Csa11g103980	AT5G66940
Csa18g040580	AT5G66940
Csa02g075810	AT5G66940
