# Supplementary material for: Genome-wide identification and functional analysis of Dof transcription factor family in Camelina sativa
Source: BMC Genomics. 2022 Dec 8;23:812. doi: 10.1186/s12864-022-09056-9 (PMC9730592; doi:10.1186/s12864-022-09056-9)
Supplement: Supplementary file 6 — Additional file 6: Table S4. All accession numbers of sequences used in this article. [file 12864_2022_9056_MOESM6_ESM.pdf]

Table S4. All accession numbers of sequences used in this article.

Species	Gene name	Gene ID
A. thaliana	*AtDof1.1*	At1g07640
	AtDof1.2	At1g21340
	AtDof1.3	At1g26790
	AtDof1.4	At1g28310
	AtDof1.5	At1g29160
	AtDof1.6	At1g47650
	AtDof1.7	At1g51700
	AtDof1.8	At1g64620
	AtDof1.10	At1g69570
	AtDof2.1	At2g28510
	AtDof2.2	At2g28810
	AtDof2.3	At2g34140
	AtDof2.4	At2g37590
	AtDof2.5	At2g46590
	AtDof3.1	At3g21270
	AtDof3.2	At3g45610
	AtDof3.3	At3g47500
	AtDof3.4	At3g50410
	AtDof3.5	At3g52440
	AtDof3.6	At3g55370
	AtDof3.7	At3g61850
	AtDof4.1	At4g00940
	AtDof4.2	At4g21030
	AtDof4.3	At4g21040
	AtDof4.4	At4g21050
	AtDof4.5	At4g21080
	AtDof4.6	At4g24060
	AtDof4.7	At4g38000
	AtDof5.1	At5g02460
	AtDof5.2	At5g39660
	AtDof5.3	At5g60200
	AtDof5.4	At5g60850
	AtDof5.5	At5g62430
	AtDof5.6	At5g62940
	AtDof5.7	At5g65590
	AtDof5.8	At5g66940

Species	Gene ID
B. napus	BnaA01g00130D
	BnaA01g21270D
	BnaA01g25190D
	BnaA02g06470D

Species	Gene ID
B. napus	BnaA03g17300D
	BnaA03g21490D
	BnaA03g36180D
	BnaA03g39760D
	BnaA03g41790D
	BnaA04g16440D
	BnaA04g16710D
	BnaA04g19980D
	BnaA04g27160D
	BnaA05g15330D
	BnaA06g15080D
	BnaA06g24490D
	BnaA07g08180D
	BnaA07g08490D
	BnaA07g12230D
	BnaA07g24230D
	BnaA08g01830D
	BnaA08g03730D
	BnaA08g09880D
	BnaA08g09890D
	BnaA08g09900D
	BnaA08g09920D
	BnaA08g15120D
	BnaA08g19870D
	BnaA08g29080D
	BnaA09g05460D
	BnaA09g06280D
	BnaA09g07670D
	BnaA09g29270D
	BnaA09g34950D
	BnaA09g49290D
	BnaA10g13030D
	BnaA10g27100D
	BnaC01g01040D
	BnaC01g12440D
	BnaC02g41820D
	BnaC02g43890D
	BnaC03g20790D
	BnaC03g25870D
	BnaC03g42040D
	BnaC03g58500D
	BnaC03g58930D

Species	Gene ID
B. napus	BnaC03g62060D
	BnaC03g63990D
	BnaC03g64440D
	BnaC03g64450D
	BnaC03g64460D
	BnaC04g00830D
	BnaC04g31720D
	BnaC04g44370D
	BnaC04g45310D
	BnaC04g50910D
	BnaC05g05320D
	BnaC05g16580D
	BnaC05g22130D
	BnaC06g01120D
	BnaC06g08980D
	BnaC06g12530D
	BnaC07g14530D
	BnaC07g16430D
	BnaC07g31770D
	BnaC07g32880D
	BnaC07g36540D
	BnaC08g21910D
	BnaC09g05040D
	BnaC09g07610D
	BnaC09g12040D
