# Supplementary material for: Genome-wide identification and functional analysis of Dof transcription factor family in Camelina sativa
Source: BMC Genomics. 2022 Dec 8;23:812. doi: 10.1186/s12864-022-09056-9 (PMC9730592; doi:10.1186/s12864-022-09056-9)
Supplement: Supplementary file 7 — Additional file 7: Table S5. All gene pairs of C. sativa and B. napus. [file 12864_2022_9056_MOESM7_ESM.pdf]

**Table S5. All gene pairs of *C. sativa* and *B. napus.***

| *C. sativa* | *B. napus* |
|---|---|
| Csa01g024360 | BnaA01g25190D |
| Csa01g024360 | BnaA03g36180D |
| Csa01g024360 | BnaC03g42040D |
| Csa10g003120 | BnaA01g00130D |
| Csa10g003120 | BnaC01g01040D |
| Csa10g022470 | BnaC03g64440D |
| Csa10g022470 | BnaC03g63990D |
| Csa10g046910 | BnaC04g31720D |
| Csa10g022470 | BnaC07g36540D |
| Csa11g003480 | BnaA01g00130D |
| Csa11g094130 | BnaA02g06470D |
| Csa11g094710 | BnaA03g39760D |
| Csa11g103980 | BnaA07g12230D |
| Csa11g103980 | BnaA09g07670D |
| Csa11g094130 | BnaA10g13030D |
| Csa11g003480 | BnaC01g01040D |
| Csa11g102460 | BnaC02g43890D |
| Csa11g025480 | BnaC03g64440D |
| Csa11g055680 | BnaC04g31720D |
| Csa11g103980 | BnaC07g16430D |
| Csa11g025480 | BnaC07g36540D |
| Csa11g103980 | BnaC09g07610D |
| Csa12g003340 | BnaA01g00130D |
| Csa12g003340 | BnaC01g01040D |
| Csa12g037400 | BnaC03g64440D |
| Csa12g081790 | BnaC04g31720D |
| Csa12g037400 | BnaC07g36540D |
| Csa14g059960 | BnaA05g15330D |
| Csa14g026580 | BnaA06g15080D |
| Csa14g036700 | BnaA07g08180D |
| Csa14g031920 | BnaA08g19870D |
| Csa14g051530 | BnaA08g03730D |
| Csa14g009010 | BnaA08g29080D |
| Csa14g059960 | BnaA08g01830D |
| Csa14g031920 | BnaA09g29270D |
| Csa14g036700 | BnaC03g58930D |
| Csa14g026580 | BnaC05g16580D |
| Csa14g009010 | BnaC05g05320D |
| Csa14g036700 | BnaC05g22130D |
| Csa14g059960 | BnaC06g08980D |
| Csa14g026580 | BnaC07g14530D |
| Csa15g031650 | BnaA01g25190D |
| Csa15g031650 | BnaA03g36180D |
| Csa15g031650 | BnaC03g42040D |
| Csa16g007040 | BnaA04g19980D |
| Csa16g016240 | BnaA04g16710D |
| Csa16g016640 | BnaA04g16440D |
| Csa16g031000 | BnaA07g24230D |
| Csa16g007040 | BnaC04g44370D |

| *C. sativa* | *B. napus* |
|---|---|
| Csa17g090650 | BnaA05g15330D |
| Csa17g027260 | BnaA06g15080D |
| Csa17g041880 | BnaA07g08180D |
| Csa17g034060 | BnaA08g19870D |
| Csa17g073130 | BnaA08g03730D |
| Csa17g011020 | BnaA08g29080D |
| Csa17g034060 | BnaA09g29270D |
| Csa17g041880 | BnaC03g58930D |
| Csa17g011020 | BnaC05g05320D |
| Csa17g041880 | BnaC05g22130D |
| Csa17g027260 | BnaC05g16580D |
| Csa17g090650 | BnaC06g08980D |
| Csa17g027260 | BnaC07g14530D |
| Csa18g033540 | BnaA02g06470D |
| Csa18g034230 | BnaA03g39760D |
| Csa18g040580 | BnaA07g12230D |
| Csa18g040580 | BnaA09g07670D |
| Csa18g033540 | BnaA10g13030D |
| Csa18g039010 | BnaC02g43890D |
| Csa18g040580 | BnaC07g16430D |
| Csa18g040580 | BnaC09g07610D |
| Csa19g029310 | BnaA01g25190D |
| Csa19g029310 | BnaA03g36180D |
| Csa19g029310 | BnaC03g42040D |
| Csa02g067130 | BnaA02g06470D |
| Csa02g067810 | BnaA03g39760D |
| Csa02g075810 | BnaA07g12230D |
| Csa02g075810 | BnaA09g07670D |
| Csa02g067130 | BnaA10g13030D |
| Csa02g075810 | BnaC07g16430D |
| Csa02g075810 | BnaC09g07610D |
| Csa03g058660 | BnaA05g15330D |
| Csa03g025140 | BnaA06g15080D |
| Csa03g032300 | BnaA07g08180D |
| Csa03g051350 | BnaA08g03730D |
| Csa03g028730 | BnaA08g19870D |
| Csa03g011080 | BnaA08g29080D |
| Csa03g028730 | BnaA09g29270D |
| Csa03g032300 | BnaC03g58930D |
| Csa03g011080 | BnaC05g05320D |
| Csa03g025140 | BnaC05g16580D |
| Csa03g032300 | BnaC05g22130D |
| Csa03g058660 | BnaC06g08980D |
| Csa03g025140 | BnaC07g14530D |
| Csa04g036060 | BnaA01g21270D |
| Csa04g065700 | BnaA03g21490D |
| Csa04g041620 | BnaA09g34950D |
| Csa04g065700 | BnaC03g25870D |
| Csa04g065700 | BnaC04g50910D |
| Csa04g065700 | BnaC04g00830D |
| Csa05g002560 | BnaA03g21490D |

| *C. sativa* | *B. napus* |
|---|---|
| Csa05g035600 | BnaA04g16710D |
| Csa05g086300 | BnaA07g24230D |
| Csa05g002560 | BnaC03g25870D |
| Csa05g002560 | BnaC04g50910D |
| Csa05g002560 | BnaC04g00830D |
| Csa06g024450 | BnaA01g21270D |
| Csa06g029980 | BnaA09g34950D |
| Csa06g024450 | BnaC08g21910D |
| Csa07g007240 | BnaA04g19980D |
| Csa07g015680 | BnaA04g16710D |
| Csa07g036310 | BnaA07g24230D |
| Csa07g007240 | BnaC04g44370D |
| Csa09g053360 | BnaA01g21270D |
| Csa09g067350 | BnaA09g34950D |
| Csa09g099480 | BnaC09g12040D |
