# Supplementary material for: Genome-wide identification and functional analysis of Dof transcription factor family in Camelina sativa
Source: BMC Genomics. 2022 Dec 8;23:812. doi: 10.1186/s12864-022-09056-9 (PMC9730592; doi:10.1186/s12864-022-09056-9)
Supplement: Supplementary file 8 — Additional file 8: Table S6. Details of the cis-elements identified in CsDof gene family. [file 12864_2022_9056_MOESM8_ESM.pdf]

Table S6. Details of the cis-elements identified in *CsDof* gene family.

Name	Cis-element	Start position	Stop position	Function
CsDof1	ABRE	1737	1742	abscisic acid responsiveness
CsDof1	TC-rich repeats	510	519	cis-acting element involved in defense and stress responsiveness
CsDof1	LTR	1042	1048	cis-acting element involved in low-temperature responsiveness
CsDof1	TCA-element	1200	1209	cis-acting element involved in salicylic acid responsiveness
CsDof1	ARE	30	36	cis-acting regulatory element essential for the anaerobic induction
CsDof1	ARE	1127	1133	cis-acting regulatory element essential for the anaerobic induction
CsDof1	ARE	1145	1151	cis-acting regulatory element essential for the anaerobic induction
CsDof1	AuxRR-core	82	89	cis-acting regulatory element involved in auxin responsiveness
CsDof1	G-Box	1737	1743	cis-acting regulatory element involved in light responsiveness
CsDof1	G-box	1178	1184	cis-acting regulatory element involved in light responsiveness
CsDof1	G-box	1737	1745	cis-acting regulatory element involved in light responsiveness
CsDof1	CGTCA-motif	365	370	cis-acting regulatory element involved in the MeJA-responsiveness
CsDof1	TGACG-motif	365	370	cis-acting regulatory element involved in the MeJA-responsiveness
CsDof1	CAAT-box	72	77	common cis-acting element in promoter and enhancer regions
CsDof1	CAAT-box	121	126	common cis-acting element in promoter and enhancer regions
CsDof1	CAAT-box	443	448	common cis-acting element in promoter and enhancer regions
CsDof1	CAAT-box	667	672	common cis-acting element in promoter and enhancer regions
CsDof1	CAAT-box	733	738	common cis-acting element in promoter and enhancer regions
CsDof1	CAAT-box	764	769	common cis-acting element in promoter and enhancer regions
CsDof1	CAAT-box	797	802	common cis-acting element in promoter and enhancer regions
CsDof1	CAAT-box	803	808	common cis-acting element in promoter and enhancer regions
CsDof1	CAAT-box	922	927	common cis-acting element in promoter and enhancer regions
CsDof1	CAAT-box	1064	1069	common cis-acting element in promoter and enhancer regions
CsDof1	CAAT-box	1218	1223	common cis-acting element in promoter and enhancer regions
CsDof1	CAAT-box	1244	1249	common cis-acting element in promoter and enhancer regions
CsDof1	CAAT-box	1409	1414	common cis-acting element in promoter and enhancer regions
CsDof1	CAAT-box	1594	1599	common cis-acting element in promoter and enhancer regions
CsDof1	CAAT-box	1674	1679	common cis-acting element in promoter and enhancer regions
CsDof1	CAAT-box	1898	1903	common cis-acting element in promoter and enhancer regions
CsDof1	CAAT-box	1956	1961	common cis-acting element in promoter and enhancer regions
CsDof1	TATA-box	92	98	core promoter element around -30 of transcription start
CsDof1	TATA-box	93	99	core promoter element around -30 of transcription start
CsDof1	TATA-box	94	98	core promoter element around -30 of transcription start
CsDof1	TATA-box	259	263	core promoter element around -30 of transcription start
CsDof1	TATA-box	291	297	core promoter element around -30 of transcription start
CsDof1	TATA-box	292	298	core promoter element around -30 of transcription start
CsDof1	TATA-box	293	299	core promoter element around -30 of transcription start
CsDof1	TATA-box	294	298	core promoter element around -30 of transcription start
CsDof1	TATA-box	302	306	core promoter element around -30 of transcription start
CsDof1	TATA-box	307	311	core promoter element around -30 of transcription start
CsDof1	TATA-box	322	328	core promoter element around -30 of transcription start
CsDof1	TATA-box	323	328	core promoter element around -30 of transcription start
CsDof1	TATA-box	324	328	core promoter element around -30 of transcription start
CsDof1	TATA-box	327	333	core promoter element around -30 of transcription start
CsDof1	TATA-box	328	333	core promoter element around -30 of transcription start
CsDof1	TATA-box	329	333	core promoter element around -30 of transcription start
CsDof1	TATA-box	355	361	core promoter element around -30 of transcription start
CsDof1	TATA-box	356	362	core promoter element around -30 of transcription start
CsDof1	TATA-box	357	363	core promoter element around -30 of transcription start
CsDof1	TATA-box	358	362	core promoter element around -30 of transcription start

Name	Cis-element	Start position	Stop position	Function
CsDof1	TATA-box	492	499	core promoter element around -30 of transcription start
CsDof1	TATA-box	493	499	core promoter element around -30 of transcription start
CsDof1	TATA-box	494	499	core promoter element around -30 of transcription start
CsDof1	TATA-box	495	499	core promoter element around -30 of transcription start
CsDof1	TATA-box	621	627	core promoter element around -30 of transcription start
CsDof1	TATA-box	622	626	core promoter element around -30 of transcription start
CsDof1	TATA-box	749	755	core promoter element around -30 of transcription start
CsDof1	TATA-box	751	755	core promoter element around -30 of transcription start
CsDof1	TATA-box	821	825	core promoter element around -30 of transcription start
CsDof1	TATA-box	829	835	core promoter element around -30 of transcription start
CsDof1	TATA-box	830	835	core promoter element around -30 of transcription start
CsDof1	TATA-box	831	835	core promoter element around -30 of transcription start
CsDof1	TATA-box	1157	1165	core promoter element around -30 of transcription start
CsDof1	TATA-box	1158	1165	core promoter element around -30 of transcription start
CsDof1	TATA-box	1159	1165	core promoter element around -30 of transcription start
CsDof1	TATA-box	1160	1165	core promoter element around -30 of transcription start
CsDof1	TATA-box	1161	1165	core promoter element around -30 of transcription start
CsDof1	TATA-box	1231	1237	core promoter element around -30 of transcription start
CsDof1	TATA-box	1232	1237	core promoter element around -30 of transcription start
CsDof1	TATA-box	1233	1237	core promoter element around -30 of transcription start
CsDof1	TATA-box	1302	1306	core promoter element around -30 of transcription start
CsDof1	TATA-box	1337	1344	core promoter element around -30 of transcription start
CsDof1	TATA-box	1370	1376	core promoter element around -30 of transcription start
CsDof1	TATA-box	1371	1377	core promoter element around -30 of transcription start
CsDof1	TATA-box	1372	1376	core promoter element around -30 of transcription start
CsDof1	TATA-box	1537	1543	core promoter element around -30 of transcription start
CsDof1	TATA-box	1538	1543	core promoter element around -30 of transcription start
CsDof1	TATA-box	1539	1543	core promoter element around -30 of transcription start
CsDof1	TATA-box	1604	1612	core promoter element around -30 of transcription start
CsDof1	TATA-box	1652	1658	core promoter element around -30 of transcription start
CsDof1	TATA-box	1653	1658	core promoter element around -30 of transcription start
CsDof1	TATA-box	1654	1658	core promoter element around -30 of transcription start
CsDof1	TATA-box	1822	1826	core promoter element around -30 of transcription start
CsDof1	TATA-box	1841	1847	core promoter element around -30 of transcription start
CsDof1	TATA-box	1842	1848	core promoter element around -30 of transcription start
CsDof1	TATA-box	1843	1849	core promoter element around -30 of transcription start
CsDof1	TATA-box	1844	1850	core promoter element around -30 of transcription start
CsDof1	TATA-box	1846	1850	core promoter element around -30 of transcription start
CsDof1	TATA-box	1868	1872	core promoter element around -30 of transcription start
CsDof1	TATA-box	1900	1908	core promoter element around -30 of transcription start
CsDof1	TATA-box	1934	1938	core promoter element around -30 of transcription start
CsDof1	Sp1	1732	1738	light responsive element
CsDof1	GT1-motif	559	565	light responsive element
CsDof1	GT1-motif	1457	1464	light responsive element
CsDof1	MRE	612	619	MYB binding site involved in light responsiveness
CsDof2	ABRE	799	804	abscisic acid responsiveness
CsDof2	TGA-element	456	462	auxin-responsive element
CsDof2	TGA-element	1807	1813	auxin-responsive element
CsDof2	AT-rich element	1259	1269	binding site of AT-rich DNA binding protein (ATBP-1)
CsDof2	TCA-element	661	670	cis-acting element involved in salicylic acid responsiveness
CsDof2	ARE	690	696	cis-acting regulatory element essential for the anaerobic induction

Name	Cis-element	Start position	Stop position	Function
CsDof2	ARE	1112	1118	cis-acting regulatory element essential for the anaerobic induction
CsDof2	ARE	1316	1322	cis-acting regulatory element essential for the anaerobic induction
CsDof2	G-box	798	804	cis-acting regulatory element involved in light responsiveness
CsDof2	CGTCA-motif	874	879	cis-acting regulatory element involved in the MeJA-responsiveness
CsDof2	CGTCA-motif	898	903	cis-acting regulatory element involved in the MeJA-responsiveness
CsDof2	TGACG-motif	874	879	cis-acting regulatory element involved in the MeJA-responsiveness
CsDof2	TGACG-motif	898	903	cis-acting regulatory element involved in the MeJA-responsiveness
CsDof2	CAAT-box	26	31	common cis-acting element in promoter and enhancer regions
CsDof2	CAAT-box	78	83	common cis-acting element in promoter and enhancer regions
CsDof2	CAAT-box	717	722	common cis-acting element in promoter and enhancer regions
CsDof2	CAAT-box	720	725	common cis-acting element in promoter and enhancer regions
CsDof2	CAAT-box	769	774	common cis-acting element in promoter and enhancer regions
CsDof2	CAAT-box	1030	1035	common cis-acting element in promoter and enhancer regions
CsDof2	CAAT-box	1048	1053	common cis-acting element in promoter and enhancer regions
CsDof2	CAAT-box	1253	1258	common cis-acting element in promoter and enhancer regions
CsDof2	CAAT-box	1289	1294	common cis-acting element in promoter and enhancer regions
CsDof2	CAAT-box	1346	1351	common cis-acting element in promoter and enhancer regions
CsDof2	CAAT-box	1377	1382	common cis-acting element in promoter and enhancer regions
CsDof2	CAAT-box	1429	1434	common cis-acting element in promoter and enhancer regions
CsDof2	TATA-box	139	145	core promoter element around -30 of transcription start
CsDof2	TATA-box	140	144	core promoter element around -30 of transcription start
CsDof2	TATA-box	165	174	core promoter element around -30 of transcription start
CsDof2	TATA-box	166	173	core promoter element around -30 of transcription start
CsDof2	TATA-box	167	173	core promoter element around -30 of transcription start
CsDof2	TATA-box	168	175	core promoter element around -30 of transcription start
CsDof2	TATA-box	169	175	core promoter element around -30 of transcription start
CsDof2	TATA-box	171	175	core promoter element around -30 of transcription start
CsDof2	TATA-box	177	186	core promoter element around -30 of transcription start
CsDof2	TATA-box	178	184	core promoter element around -30 of transcription start
CsDof2	TATA-box	179	186	core promoter element around -30 of transcription start
CsDof2	TATA-box	180	186	core promoter element around -30 of transcription start
CsDof2	TATA-box	181	187	core promoter element around -30 of transcription start
CsDof2	TATA-box	182	186	core promoter element around -30 of transcription start
CsDof2	TATA-box	304	311	core promoter element around -30 of transcription start
CsDof2	TATA-box	330	336	core promoter element around -30 of transcription start
CsDof2	TATA-box	331	335	core promoter element around -30 of transcription start
CsDof2	TATA-box	336	344	core promoter element around -30 of transcription start
CsDof2	TATA-box	414	418	core promoter element around -30 of transcription start
CsDof2	TATA-box	532	539	core promoter element around -30 of transcription start
CsDof2	TATA-box	533	539	core promoter element around -30 of transcription start
CsDof2	TATA-box	534	540	core promoter element around -30 of transcription start
CsDof2	TATA-box	535	539	core promoter element around -30 of transcription start
CsDof2	TATA-box	644	651	core promoter element around -30 of transcription start
CsDof2	TATA-box	645	651	core promoter element around -30 of transcription start
CsDof2	TATA-box	646	653	core promoter element around -30 of transcription start
CsDof2	TATA-box	647	653	core promoter element around -30 of transcription start
CsDof2	TATA-box	648	654	core promoter element around -30 of transcription start
CsDof2	TATA-box	649	653	core promoter element around -30 of transcription start
CsDof2	TATA-box	729	735	core promoter element around -30 of transcription start
CsDof2	TATA-box	731	735	core promoter element around -30 of transcription start
CsDof2	TATA-box	774	780	core promoter element around -30 of transcription start

Name	Cis-element	Start position	Stop position	Function
CsDof2	TATA-box	775	780	core promoter element around -30 of transcription start
CsDof2	TATA-box	776	780	core promoter element around -30 of transcription start
CsDof2	TATA-box	959	965	core promoter element around -30 of transcription start
CsDof2	TATA-box	960	967	core promoter element around -30 of transcription start
CsDof2	TATA-box	961	967	core promoter element around -30 of transcription start
CsDof2	TATA-box	962	968	core promoter element around -30 of transcription start
CsDof2	TATA-box	963	967	core promoter element around -30 of transcription start
CsDof2	TATA-box	1162	1166	core promoter element around -30 of transcription start
CsDof2	TATA-box	1490	1496	core promoter element around -30 of transcription start
CsDof2	TATA-box	1491	1495	core promoter element around -30 of transcription start
CsDof2	TATA-box	1516	1525	core promoter element around -30 of transcription start
CsDof2	TATA-box	1517	1524	core promoter element around -30 of transcription start
CsDof2	TATA-box	1518	1524	core promoter element around -30 of transcription start
CsDof2	TATA-box	1519	1526	core promoter element around -30 of transcription start
CsDof2	TATA-box	1520	1526	core promoter element around -30 of transcription start
CsDof2	TATA-box	1522	1526	core promoter element around -30 of transcription start
CsDof2	TATA-box	1528	1537	core promoter element around -30 of transcription start
CsDof2	TATA-box	1529	1535	core promoter element around -30 of transcription start
CsDof2	TATA-box	1530	1537	core promoter element around -30 of transcription start
CsDof2	TATA-box	1531	1537	core promoter element around -30 of transcription start
CsDof2	TATA-box	1532	1538	core promoter element around -30 of transcription start
CsDof2	TATA-box	1533	1537	core promoter element around -30 of transcription start
CsDof2	TATA-box	1655	1662	core promoter element around -30 of transcription start
CsDof2	TATA-box	1681	1687	core promoter element around -30 of transcription start
CsDof2	TATA-box	1682	1686	core promoter element around -30 of transcription start
CsDof2	TATA-box	1687	1695	core promoter element around -30 of transcription start
CsDof2	TATA-box	1765	1769	core promoter element around -30 of transcription start
CsDof2	TATA-box	1883	1890	core promoter element around -30 of transcription start
CsDof2	TATA-box	1884	1890	core promoter element around -30 of transcription start
CsDof2	TATA-box	1885	1891	core promoter element around -30 of transcription start
CsDof2	TATA-box	1886	1890	core promoter element around -30 of transcription start
CsDof2	GT1-motif	44	50	light responsive element
CsDof2	GT1-motif	1238	1244	light responsive element
CsDof2	GT1-motif	1395	1401	light responsive element
CsDof2	Sp1	842	848	light responsive element
CsDof2	MRE	913	920	MYB binding site involved in light responsiveness
CsDof3	TGA-element	1773	1779	auxin-responsive element
CsDof3	TATC-box	463	470	cis-acting element involved in gibberellin-responsiveness
CsDof3	ARE	591	597	cis-acting regulatory element essential for the anaerobic induction
CsDof3	ARE	1140	1146	cis-acting regulatory element essential for the anaerobic induction
CsDof3	ARE	1207	1213	cis-acting regulatory element essential for the anaerobic induction
CsDof3	ARE	1543	1549	cis-acting regulatory element essential for the anaerobic induction
CsDof3	circadian	1614	1623	cis-acting regulatory element involved in circadian control
CsDof3	CAAT-box	25	30	common cis-acting element in promoter and enhancer regions
CsDof3	CAAT-box	38	43	common cis-acting element in promoter and enhancer regions
CsDof3	CAAT-box	44	49	common cis-acting element in promoter and enhancer regions
CsDof3	CAAT-box	229	234	common cis-acting element in promoter and enhancer regions
CsDof3	CAAT-box	315	320	common cis-acting element in promoter and enhancer regions
CsDof3	CAAT-box	481	489	common cis-acting element in promoter and enhancer regions
CsDof3	CAAT-box	483	488	common cis-acting element in promoter and enhancer regions
CsDof3	CAAT-box	537	542	common cis-acting element in promoter and enhancer regions

Name	Cis-element	Start position	Stop position	Function
CsDof3	CAAT-box	588	593	common cis-acting element in promoter and enhancer regions
CsDof3	CAAT-box	982	987	common cis-acting element in promoter and enhancer regions
CsDof3	CAAT-box	1021	1026	common cis-acting element in promoter and enhancer regions
CsDof3	CAAT-box	1489	1494	common cis-acting element in promoter and enhancer regions
CsDof3	CAAT-box	1627	1632	common cis-acting element in promoter and enhancer regions
CsDof3	CAAT-box	1732	1737	common cis-acting element in promoter and enhancer regions
CsDof3	CAAT-box	1857	1862	common cis-acting element in promoter and enhancer regions
CsDof3	CAAT-box	1878	1883	common cis-acting element in promoter and enhancer regions
CsDof3	TATA-box	134	138	core promoter element around -30 of transcription start
CsDof3	TATA-box	237	243	core promoter element around -30 of transcription start
CsDof3	TATA-box	238	243	core promoter element around -30 of transcription start
CsDof3	TATA-box	239	243	core promoter element around -30 of transcription start
CsDof3	TATA-box	284	296	core promoter element around -30 of transcription start
CsDof3	TATA-box	292	298	core promoter element around -30 of transcription start
CsDof3	TATA-box	293	297	core promoter element around -30 of transcription start
CsDof3	TATA-box	321	328	core promoter element around -30 of transcription start
CsDof3	TATA-box	322	328	core promoter element around -30 of transcription start
CsDof3	TATA-box	323	328	core promoter element around -30 of transcription start
CsDof3	TATA-box	324	328	core promoter element around -30 of transcription start
CsDof3	TATA-box	326	334	core promoter element around -30 of transcription start
CsDof3	TATA-box	333	339	core promoter element around -30 of transcription start
CsDof3	TATA-box	334	339	core promoter element around -30 of transcription start
CsDof3	TATA-box	335	339	core promoter element around -30 of transcription start
CsDof3	TATA-box	350	354	core promoter element around -30 of transcription start
CsDof3	TATA-box	403	410	core promoter element around -30 of transcription start
CsDof3	TATA-box	424	430	core promoter element around -30 of transcription start
CsDof3	TATA-box	425	429	core promoter element around -30 of transcription start
CsDof3	TATA-box	495	501	core promoter element around -30 of transcription start
CsDof3	TATA-box	496	501	core promoter element around -30 of transcription start
CsDof3	TATA-box	497	501	core promoter element around -30 of transcription start
CsDof3	TATA-box	508	512	core promoter element around -30 of transcription start
CsDof3	TATA-box	614	622	core promoter element around -30 of transcription start
CsDof3	TATA-box	644	652	core promoter element around -30 of transcription start
CsDof3	TATA-box	679	685	core promoter element around -30 of transcription start
CsDof3	TATA-box	680	685	core promoter element around -30 of transcription start
CsDof3	TATA-box	681	685	core promoter element around -30 of transcription start
CsDof3	TATA-box	720	726	core promoter element around -30 of transcription start
CsDof3	TATA-box	721	725	core promoter element around -30 of transcription start
CsDof3	TATA-box	752	759	core promoter element around -30 of transcription start
CsDof3	TATA-box	753	759	core promoter element around -30 of transcription start
CsDof3	TATA-box	755	759	core promoter element around -30 of transcription start
CsDof3	TATA-box	778	787	core promoter element around -30 of transcription start
CsDof3	TATA-box	779	786	core promoter element around -30 of transcription start
CsDof3	TATA-box	780	786	core promoter element around -30 of transcription start
CsDof3	TATA-box	781	788	core promoter element around -30 of transcription start
CsDof3	TATA-box	782	788	core promoter element around -30 of transcription start
CsDof3	TATA-box	783	789	core promoter element around -30 of transcription start
CsDof3	TATA-box	784	788	core promoter element around -30 of transcription start
CsDof3	TATA-box	918	924	core promoter element around -30 of transcription start
CsDof3	TATA-box	919	924	core promoter element around -30 of transcription start
CsDof3	TATA-box	920	924	core promoter element around -30 of transcription start

Name	Cis-element	Start position	Stop position	Function
CsDof3	TATA-box	931	937	core promoter element around -30 of transcription start
CsDof3	TATA-box	932	939	core promoter element around -30 of transcription start
CsDof3	TATA-box	933	939	core promoter element around -30 of transcription start
CsDof3	TATA-box	935	939	core promoter element around -30 of transcription start
CsDof3	TATA-box	955	962	core promoter element around -30 of transcription start
CsDof3	TATA-box	956	962	core promoter element around -30 of transcription start
CsDof3	TATA-box	957	962	core promoter element around -30 of transcription start
CsDof3	TATA-box	958	962	core promoter element around -30 of transcription start
CsDof3	TATA-box	962	968	core promoter element around -30 of transcription start
CsDof3	TATA-box	963	968	core promoter element around -30 of transcription start
CsDof3	TATA-box	964	968	core promoter element around -30 of transcription start
CsDof3	TATA-box	996	1004	core promoter element around -30 of transcription start
CsDof3	TATA-box	998	1006	core promoter element around -30 of transcription start
CsDof3	TATA-box	1012	1017	core promoter element around -30 of transcription start
CsDof3	TATA-box	1013	1017	core promoter element around -30 of transcription start
CsDof3	TATA-box	1034	1038	core promoter element around -30 of transcription start
CsDof3	TATA-box	1063	1068	core promoter element around -30 of transcription start
CsDof3	TATA-box	1064	1068	core promoter element around -30 of transcription start
CsDof3	TATA-box	1246	1258	core promoter element around -30 of transcription start
CsDof3	TATA-box	1255	1259	core promoter element around -30 of transcription start
CsDof3	TATA-box	1288	1294	core promoter element around -30 of transcription start
CsDof3	TATA-box	1289	1293	core promoter element around -30 of transcription start
CsDof3	TATA-box	1316	1322	core promoter element around -30 of transcription start
CsDof3	TATA-box	1317	1322	core promoter element around -30 of transcription start
CsDof3	TATA-box	1318	1322	core promoter element around -30 of transcription start
CsDof3	TATA-box	1637	1645	core promoter element around -30 of transcription start
CsDof3	TATA-box	1787	1791	core promoter element around -30 of transcription start
CsDof3	TATA-box	1889	1898	core promoter element around -30 of transcription start
CsDof3	TATA-box	1890	1897	core promoter element around -30 of transcription start
CsDof3	TATA-box	1891	1897	core promoter element around -30 of transcription start
CsDof3	TATA-box	1892	1897	core promoter element around -30 of transcription start
CsDof3	TATA-box	1893	1897	core promoter element around -30 of transcription start
CsDof3	TATA-box	1971	1977	core promoter element around -30 of transcription start
CsDof3	TATA-box	1972	1977	core promoter element around -30 of transcription start
CsDof3	TATA-box	1973	1977	core promoter element around -30 of transcription start
CsDof3	TATA-box	1992	1997	core promoter element around -30 of transcription start
CsDof3	TATA-box	1993	1997	core promoter element around -30 of transcription start
CsDof3	AT-rich sequence	1845	1854	element for maximal elicitor-mediated activation (2copies)
CsDof3	GT1-motif	629	636	light responsive element
CsDof3	GT1-motif	974	980	light responsive element
CsDof4	ABRE	1700	1705	abscisic acid responsiveness
CsDof4	TGA-element	1204	1210	auxin-responsive element
CsDof4	LTR	327	333	cis-acting element involved in low-temperature responsiveness
CsDof4	LTR	1293	1299	cis-acting element involved in low-temperature responsiveness
CsDof4	Unnamed_1	360	371	cis-acting element involved in phytochrome down-regulation expression
CsDof4	ARE	96	102	cis-acting regulatory element essential for the anaerobic induction
CsDof4	ARE	861	867	cis-acting regulatory element essential for the anaerobic induction
CsDof4	AuxRR-core	998	1005	cis-acting regulatory element involved in auxin responsiveness
CsDof4	G-Box	1700	1706	cis-acting regulatory element involved in light responsiveness
CsDof4	G-box	185	191	cis-acting regulatory element involved in light responsiveness
CsDof4	CAT-box	1268	1274	cis-acting regulatory element related to meristem expression

Name	Cis-element	Start position	Stop position	Function
CsDof4	CAAT-box	297	302	common cis-acting element in promoter and enhancer regions
CsDof4	CAAT-box	344	349	common cis-acting element in promoter and enhancer regions
CsDof4	CAAT-box	431	436	common cis-acting element in promoter and enhancer regions
CsDof4	CAAT-box	437	442	common cis-acting element in promoter and enhancer regions
CsDof4	CAAT-box	671	676	common cis-acting element in promoter and enhancer regions
CsDof4	CAAT-box	749	754	common cis-acting element in promoter and enhancer regions
CsDof4	CAAT-box	789	794	common cis-acting element in promoter and enhancer regions
CsDof4	CAAT-box	868	873	common cis-acting element in promoter and enhancer regions
CsDof4	CAAT-box	1066	1071	common cis-acting element in promoter and enhancer regions
CsDof4	CAAT-box	1197	1202	common cis-acting element in promoter and enhancer regions
CsDof4	CAAT-box	1260	1265	common cis-acting element in promoter and enhancer regions
CsDof4	CAAT-box	1416	1421	common cis-acting element in promoter and enhancer regions
CsDof4	CAAT-box	1429	1434	common cis-acting element in promoter and enhancer regions
CsDof4	CAAT-box	1695	1700	common cis-acting element in promoter and enhancer regions
CsDof4	CAAT-box	1710	1715	common cis-acting element in promoter and enhancer regions
CsDof4	TATA-box	15	19	core promoter element around -30 of transcription start
CsDof4	TATA-box	31	38	core promoter element around -30 of transcription start
CsDof4	TATA-box	86	93	core promoter element around -30 of transcription start
CsDof4	TATA-box	209	213	core promoter element around -30 of transcription start
CsDof4	TATA-box	214	218	core promoter element around -30 of transcription start
CsDof4	TATA-box	251	255	core promoter element around -30 of transcription start
CsDof4	TATA-box	256	260	core promoter element around -30 of transcription start
CsDof4	TATA-box	410	416	core promoter element around -30 of transcription start
CsDof4	TATA-box	412	416	core promoter element around -30 of transcription start
CsDof4	TATA-box	487	493	core promoter element around -30 of transcription start
CsDof4	TATA-box	488	494	core promoter element around -30 of transcription start
CsDof4	TATA-box	489	495	core promoter element around -30 of transcription start
CsDof4	TATA-box	490	496	core promoter element around -30 of transcription start
CsDof4	TATA-box	491	497	core promoter element around -30 of transcription start
CsDof4	TATA-box	492	496	core promoter element around -30 of transcription start
CsDof4	TATA-box	540	546	core promoter element around -30 of transcription start
CsDof4	TATA-box	541	545	core promoter element around -30 of transcription start
CsDof4	TATA-box	804	810	core promoter element around -30 of transcription start
CsDof4	TATA-box	805	811	core promoter element around -30 of transcription start
CsDof4	TATA-box	806	812	core promoter element around -30 of transcription start
CsDof4	TATA-box	807	813	core promoter element around -30 of transcription start
CsDof4	TATA-box	808	814	core promoter element around -30 of transcription start
CsDof4	TATA-box	809	815	core promoter element around -30 of transcription start
CsDof4	TATA-box	810	816	core promoter element around -30 of transcription start
CsDof4	TATA-box	811	817	core promoter element around -30 of transcription start
CsDof4	TATA-box	812	816	core promoter element around -30 of transcription start
CsDof4	TATA-box	877	883	core promoter element around -30 of transcription start
CsDof4	TATA-box	878	882	core promoter element around -30 of transcription start
CsDof4	TATA-box	953	957	core promoter element around -30 of transcription start
CsDof4	TATA-box	1177	1181	core promoter element around -30 of transcription start
CsDof4	TATA-box	1224	1233	core promoter element around -30 of transcription start
CsDof4	TATA-box	1225	1232	core promoter element around -30 of transcription start
CsDof4	TATA-box	1226	1232	core promoter element around -30 of transcription start
CsDof4	TATA-box	1227	1234	core promoter element around -30 of transcription start
CsDof4	TATA-box	1228	1234	core promoter element around -30 of transcription start
CsDof4	TATA-box	1229	1235	core promoter element around -30 of transcription start

Name	Cis-element	Start position	Stop position	Function
CsDof4	TATA-box	1230	1236	core promoter element around -30 of transcription start
CsDof4	TATA-box	1231	1237	core promoter element around -30 of transcription start
CsDof4	TATA-box	1232	1236	core promoter element around -30 of transcription start
CsDof4	TATA-box	1301	1305	core promoter element around -30 of transcription start
CsDof4	TATA-box	1306	1311	core promoter element around -30 of transcription start
CsDof4	TATA-box	1307	1311	core promoter element around -30 of transcription start
CsDof4	TATA-box	1346	1352	core promoter element around -30 of transcription start
CsDof4	TATA-box	1347	1351	core promoter element around -30 of transcription start
CsDof4	TATA-box	1397	1403	core promoter element around -30 of transcription start
CsDof4	TATA-box	1398	1403	core promoter element around -30 of transcription start
CsDof4	TATA-box	1399	1403	core promoter element around -30 of transcription start
CsDof4	TATA-box	1412	1416	core promoter element around -30 of transcription start
CsDof4	TATA-box	1443	1451	core promoter element around -30 of transcription start
CsDof4	TATA-box	1568	1574	core promoter element around -30 of transcription start
CsDof4	TATA-box	1569	1574	core promoter element around -30 of transcription start
CsDof4	TATA-box	1570	1574	core promoter element around -30 of transcription start
CsDof4	TATA-box	1657	1663	core promoter element around -30 of transcription start
CsDof4	TATA-box	1658	1665	core promoter element around -30 of transcription start
CsDof4	TATA-box	1659	1665	core promoter element around -30 of transcription start
CsDof4	TATA-box	1660	1666	core promoter element around -30 of transcription start
CsDof4	TATA-box	1661	1665	core promoter element around -30 of transcription start
CsDof4	TATA-box	1838	1844	core promoter element around -30 of transcription start
CsDof4	TATA-box	1840	1846	core promoter element around -30 of transcription start
CsDof4	TATA-box	1841	1847	core promoter element around -30 of transcription start
CsDof4	TATA-box	1842	1848	core promoter element around -30 of transcription start
CsDof4	TATA-box	1844	1848	core promoter element around -30 of transcription start
CsDof4	TATA-box	1942	1948	core promoter element around -30 of transcription start
CsDof4	TATA-box	1943	1947	core promoter element around -30 of transcription start
CsDof4	AT-rich sequence	566	575	element for maximal elicitor-mediated activation (2copies)
CsDof4	P-box	1864	1871	gibberellin-responsive element
CsDof4	GT1-motif	622	628	light responsive element
CsDof4	GT1-motif	1246	1252	light responsive element
CsDof4	MBS	592	598	MYB binding site involved in drought-inducibility
CsDof4	CCAAT-box	1057	1063	MYBHv1 binding site
CsDof5	ABRE	1220	1225	abscisic acid responsiveness
CsDof5	ABRE	1593	1599	abscisic acid responsiveness
CsDof5	ABRE	1594	1599	abscisic acid responsiveness
CsDof5	TGA-element	485	491	auxin-responsive element
CsDof5	LTR	1766	1772	cis-acting element involved in low-temperature responsiveness
CsDof5	ARE	31	37	cis-acting regulatory element essential for the anaerobic induction
CsDof5	ARE	408	414	cis-acting regulatory element essential for the anaerobic induction
CsDof5	ARE	986	992	cis-acting regulatory element essential for the anaerobic induction
CsDof5	G-Box	1220	1226	cis-acting regulatory element involved in light responsiveness
CsDof5	G-Box	1593	1599	cis-acting regulatory element involved in light responsiveness
CsDof5	G-box	1593	1599	cis-acting regulatory element involved in light responsiveness
CsDof5	TGACG-motif	1569	1574	cis-acting regulatory element involved in the MeJA-responsiveness
CsDof5	TGACG-motif	1748	1753	cis-acting regulatory element involved in the MeJA-responsiveness
CsDof5	CGTCA-motif	1569	1574	cis-acting regulatory element involved in the MeJA-responsiveness
CsDof5	CGTCA-motif	1748	1753	cis-acting regulatory element involved in the MeJA-responsiveness
CsDof5	O2-site	1313	1322	cis-acting regulatory element involved in zein metabolism regulation
CsDof5	CAAT-box	235	240	common cis-acting element in promoter and enhancer regions

Name	Cis-element	Start position	Stop position	Function
CsDof5	CAAT-box	333	338	common cis-acting element in promoter and enhancer regions
CsDof5	CAAT-box	609	614	common cis-acting element in promoter and enhancer regions
CsDof5	CAAT-box	617	622	common cis-acting element in promoter and enhancer regions
CsDof5	CAAT-box	703	708	common cis-acting element in promoter and enhancer regions
CsDof5	CAAT-box	780	785	common cis-acting element in promoter and enhancer regions
CsDof5	CAAT-box	841	846	common cis-acting element in promoter and enhancer regions
CsDof5	CAAT-box	1004	1009	common cis-acting element in promoter and enhancer regions
CsDof5	CAAT-box	1554	1559	common cis-acting element in promoter and enhancer regions
CsDof5	CAAT-box	1573	1578	common cis-acting element in promoter and enhancer regions
CsDof5	CAAT-box	1636	1641	common cis-acting element in promoter and enhancer regions
CsDof5	CAAT-box	1752	1757	common cis-acting element in promoter and enhancer regions
CsDof5	CAAT-box	1784	1789	common cis-acting element in promoter and enhancer regions
CsDof5	TATA-box	36	44	core promoter element around -30 of transcription start
CsDof5	TATA-box	40	46	core promoter element around -30 of transcription start
CsDof5	TATA-box	42	46	core promoter element around -30 of transcription start
CsDof5	TATA-box	75	81	core promoter element around -30 of transcription start
CsDof5	TATA-box	76	81	core promoter element around -30 of transcription start
CsDof5	TATA-box	77	81	core promoter element around -30 of transcription start
CsDof5	TATA-box	141	145	core promoter element around -30 of transcription start
CsDof5	TATA-box	227	231	core promoter element around -30 of transcription start
CsDof5	TATA-box	288	292	core promoter element around -30 of transcription start
CsDof5	TATA-box	531	537	core promoter element around -30 of transcription start
CsDof5	TATA-box	532	537	core promoter element around -30 of transcription start
CsDof5	TATA-box	533	537	core promoter element around -30 of transcription start
CsDof5	TATA-box	707	711	core promoter element around -30 of transcription start
CsDof5	TATA-box	1204	1208	core promoter element around -30 of transcription start
CsDof5	TATA-box	1254	1258	core promoter element around -30 of transcription start
CsDof5	TATA-box	1347	1356	core promoter element around -30 of transcription start
CsDof5	TATA-box	1639	1645	core promoter element around -30 of transcription start
CsDof5	TATA-box	1640	1644	core promoter element around -30 of transcription start
CsDof5	TATA-box	1773	1779	core promoter element around -30 of transcription start
CsDof5	TATA-box	1775	1781	core promoter element around -30 of transcription start
CsDof5	TATA-box	1776	1782	core promoter element around -30 of transcription start
CsDof5	TATA-box	1777	1781	core promoter element around -30 of transcription start
CsDof5	TATA-box	1901	1909	core promoter element around -30 of transcription start
CsDof5	TATA-box	1915	1924	core promoter element around -30 of transcription start
CsDof5	TATA-box	1916	1923	core promoter element around -30 of transcription start
CsDof5	TATA-box	1917	1923	core promoter element around -30 of transcription start
CsDof5	TATA-box	1918	1925	core promoter element around -30 of transcription start
CsDof5	TATA-box	1919	1925	core promoter element around -30 of transcription start
CsDof5	TATA-box	1921	1925	core promoter element around -30 of transcription start
CsDof5	TATA-box	1927	1933	core promoter element around -30 of transcription start
CsDof5	TATA-box	1928	1934	core promoter element around -30 of transcription start
CsDof5	TATA-box	1929	1935	core promoter element around -30 of transcription start
CsDof5	TATA-box	1930	1936	core promoter element around -30 of transcription start
CsDof5	TATA-box	1931	1937	core promoter element around -30 of transcription start
CsDof5	TATA-box	1932	1938	core promoter element around -30 of transcription start
CsDof5	TATA-box	1933	1939	core promoter element around -30 of transcription start
CsDof5	TATA-box	1934	1940	core promoter element around -30 of transcription start
CsDof5	TATA-box	1935	1941	core promoter element around -30 of transcription start
CsDof5	TATA-box	1936	1942	core promoter element around -30 of transcription start

Name	Cis-element	Start position	Stop position	Function
CsDof5	TATA-box	1937	1943	core promoter element around -30 of transcription start
CsDof5	TATA-box	1938	1944	core promoter element around -30 of transcription start
CsDof5	TATA-box	1939	1945	core promoter element around -30 of transcription start
CsDof5	TATA-box	1940	1946	core promoter element around -30 of transcription start
CsDof5	TATA-box	1941	1947	core promoter element around -30 of transcription start
CsDof5	TATA-box	1942	1948	core promoter element around -30 of transcription start
CsDof5	TATA-box	1943	1949	core promoter element around -30 of transcription start
CsDof5	TATA-box	1944	1950	core promoter element around -30 of transcription start
CsDof5	TATA-box	1945	1951	core promoter element around -30 of transcription start
CsDof5	TATA-box	1946	1952	core promoter element around -30 of transcription start
CsDof5	TATA-box	1947	1953	core promoter element around -30 of transcription start
CsDof5	TATA-box	1948	1954	core promoter element around -30 of transcription start
CsDof5	TATA-box	1949	1955	core promoter element around -30 of transcription start
CsDof5	TATA-box	1950	1956	core promoter element around -30 of transcription start
CsDof5	TATA-box	1951	1957	core promoter element around -30 of transcription start
CsDof5	TATA-box	1952	1958	core promoter element around -30 of transcription start
CsDof5	TATA-box	1954	1958	core promoter element around -30 of transcription start
CsDof5	TATA-box	1989	1998	core promoter element around -30 of transcription start
CsDof5	TATA-box	1990	1997	core promoter element around -30 of transcription start
CsDof5	TATA-box	1991	1997	core promoter element around -30 of transcription start
CsDof5	TATA-box	1992	1997	core promoter element around -30 of transcription start
CsDof5	TATA-box	1993	1997	core promoter element around -30 of transcription start
CsDof5	GARE-motif	1415	1422	gibberellin-responsive element
CsDof5	MRE	340	347	MYB binding site involved in light responsiveness
CsDof6	ABRE	879	884	abscisic acid responsiveness
CsDof6	ABRE	1025	1030	abscisic acid responsiveness
CsDof6	ABRE	1353	1358	abscisic acid responsiveness
CsDof6	TC-rich repeats	739	748	cis-acting element involved in defense and stress responsiveness
CsDof6	TCA-element	527	536	cis-acting element involved in salicylic acid responsiveness
CsDof6	ARE	171	177	cis-acting regulatory element essential for the anaerobic induction
CsDof6	ARE	626	632	cis-acting regulatory element essential for the anaerobic induction
CsDof6	ARE	1284	1290	cis-acting regulatory element essential for the anaerobic induction
CsDof6	ARE	1507	1513	cis-acting regulatory element essential for the anaerobic induction
CsDof6	ARE	1878	1884	cis-acting regulatory element essential for the anaerobic induction
CsDof6	G-box	878	884	cis-acting regulatory element involved in light responsiveness
CsDof6	G-box	1025	1031	cis-acting regulatory element involved in light responsiveness
CsDof6	G-box	1353	1359	cis-acting regulatory element involved in light responsiveness
CsDof6	CGTCA-motif	474	479	cis-acting regulatory element involved in the MeJA-responsiveness
CsDof6	CGTCA-motif	877	882	cis-acting regulatory element involved in the MeJA-responsiveness
CsDof6	TGACG-motif	474	479	cis-acting regulatory element involved in the MeJA-responsiveness
CsDof6	TGACG-motif	877	882	cis-acting regulatory element involved in the MeJA-responsiveness
CsDof6	O2-site	535	544	cis-acting regulatory element involved in zein metabolism regulation
CsDof6	O2-site	875	884	cis-acting regulatory element involved in zein metabolism regulation
CsDof6	O2-site	1817	1826	cis-acting regulatory element involved in zein metabolism regulation
CsDof6	CAAT-box	30	35	common cis-acting element in promoter and enhancer regions
CsDof6	CAAT-box	169	174	common cis-acting element in promoter and enhancer regions
CsDof6	CAAT-box	257	262	common cis-acting element in promoter and enhancer regions
CsDof6	CAAT-box	283	288	common cis-acting element in promoter and enhancer regions
CsDof6	CAAT-box	310	315	common cis-acting element in promoter and enhancer regions
CsDof6	CAAT-box	382	387	common cis-acting element in promoter and enhancer regions
CsDof6	CAAT-box	492	497	common cis-acting element in promoter and enhancer regions

Name	Cis-element	Start position	Stop position	Function
CsDof6	CAAT-box	603	612	common cis-acting element in promoter and enhancer regions
CsDof6	CAAT-box	605	610	common cis-acting element in promoter and enhancer regions
CsDof6	CAAT-box	1137	1142	common cis-acting element in promoter and enhancer regions
CsDof6	CAAT-box	1267	1272	common cis-acting element in promoter and enhancer regions
CsDof6	CAAT-box	1309	1314	common cis-acting element in promoter and enhancer regions
CsDof6	CAAT-box	1338	1343	common cis-acting element in promoter and enhancer regions
CsDof6	CAAT-box	1451	1456	common cis-acting element in promoter and enhancer regions
CsDof6	CAAT-box	1458	1463	common cis-acting element in promoter and enhancer regions
CsDof6	CAAT-box	1510	1515	common cis-acting element in promoter and enhancer regions
CsDof6	CAAT-box	1653	1658	common cis-acting element in promoter and enhancer regions
CsDof6	CAAT-box	1680	1685	common cis-acting element in promoter and enhancer regions
CsDof6	CAAT-box	1861	1866	common cis-acting element in promoter and enhancer regions
CsDof6	CAAT-box	1892	1897	common cis-acting element in promoter and enhancer regions
CsDof6	CAAT-box	1978	1983	common cis-acting element in promoter and enhancer regions
CsDof6	TATA-box	128	133	core promoter element around -30 of transcription start
CsDof6	TATA-box	129	133	core promoter element around -30 of transcription start
CsDof6	TATA-box	191	198	core promoter element around -30 of transcription start
CsDof6	TATA-box	192	198	core promoter element around -30 of transcription start
CsDof6	TATA-box	193	198	core promoter element around -30 of transcription start
CsDof6	TATA-box	194	198	core promoter element around -30 of transcription start
CsDof6	TATA-box	210	214	core promoter element around -30 of transcription start
CsDof6	TATA-box	226	232	core promoter element around -30 of transcription start
CsDof6	TATA-box	227	233	core promoter element around -30 of transcription start
CsDof6	TATA-box	228	232	core promoter element around -30 of transcription start
CsDof6	TATA-box	266	270	core promoter element around -30 of transcription start
CsDof6	TATA-box	272	278	core promoter element around -30 of transcription start
CsDof6	TATA-box	273	279	core promoter element around -30 of transcription start
CsDof6	TATA-box	274	278	core promoter element around -30 of transcription start
CsDof6	TATA-box	464	470	core promoter element around -30 of transcription start
CsDof6	TATA-box	465	469	core promoter element around -30 of transcription start
CsDof6	TATA-box	629	635	core promoter element around -30 of transcription start
CsDof6	TATA-box	630	635	core promoter element around -30 of transcription start
CsDof6	TATA-box	631	635	core promoter element around -30 of transcription start
CsDof6	TATA-box	984	989	core promoter element around -30 of transcription start
CsDof6	TATA-box	985	989	core promoter element around -30 of transcription start
CsDof6	TATA-box	1084	1092	core promoter element around -30 of transcription start
CsDof6	TATA-box	1176	1180	core promoter element around -30 of transcription start
CsDof6	TATA-box	1264	1268	core promoter element around -30 of transcription start
CsDof6	TATA-box	1481	1485	core promoter element around -30 of transcription start
CsDof6	TATA-box	1502	1507	core promoter element around -30 of transcription start
CsDof6	TATA-box	1503	1507	core promoter element around -30 of transcription start
CsDof6	TATA-box	1516	1521	core promoter element around -30 of transcription start
CsDof6	TATA-box	1517	1521	core promoter element around -30 of transcription start
CsDof6	TATA-box	1523	1529	core promoter element around -30 of transcription start
CsDof6	TATA-box	1524	1528	core promoter element around -30 of transcription start
CsDof6	TATA-box	1538	1544	core promoter element around -30 of transcription start
CsDof6	TATA-box	1539	1544	core promoter element around -30 of transcription start
CsDof6	TATA-box	1540	1544	core promoter element around -30 of transcription start
CsDof6	TATA-box	1724	1730	core promoter element around -30 of transcription start
CsDof6	TATA-box	1725	1730	core promoter element around -30 of transcription start
CsDof6	TATA-box	1726	1730	core promoter element around -30 of transcription start

Name	Cis-element	Start position	Stop position	Function
CsDof6	TATA-box	1733	1737	core promoter element around -30 of transcription start
CsDof6	GARE-motif	327	334	gibberellin-responsive element
CsDof6	MBS	3	9	MYB binding site involved in drought-inducibility
CsDof6	MBS	552	558	MYB binding site involved in drought-inducibility
CsDof8	ABRE	77	82	abscisic acid responsiveness
CsDof8	TCA-element	638	647	cis-acting element involved in salicylic acid responsiveness
CsDof8	TCA-element	1191	1200	cis-acting element involved in salicylic acid responsiveness
CsDof8	ARE	1087	1093	cis-acting regulatory element essential for the anaerobic induction
CsDof8	G-box	77	83	cis-acting regulatory element involved in light responsiveness
CsDof8	TGACG-motif	931	936	cis-acting regulatory element involved in the MeJA-responsiveness
CsDof8	TGACG-motif	1621	1626	cis-acting regulatory element involved in the MeJA-responsiveness
CsDof8	CGTCA-motif	931	936	cis-acting regulatory element involved in the MeJA-responsiveness
CsDof8	CGTCA-motif	1621	1626	cis-acting regulatory element involved in the MeJA-responsiveness
CsDof8	O2-site	1985	1994	cis-acting regulatory element involved in zein metabolism regulation
CsDof8	CAT-box	1327	1333	cis-acting regulatory element related to meristem expression
CsDof8	CAAT-box	48	53	common cis-acting element in promoter and enhancer regions
CsDof8	CAAT-box	127	132	common cis-acting element in promoter and enhancer regions
CsDof8	CAAT-box	133	138	common cis-acting element in promoter and enhancer regions
CsDof8	CAAT-box	349	354	common cis-acting element in promoter and enhancer regions
CsDof8	CAAT-box	674	679	common cis-acting element in promoter and enhancer regions
CsDof8	CAAT-box	694	699	common cis-acting element in promoter and enhancer regions
CsDof8	CAAT-box	731	736	common cis-acting element in promoter and enhancer regions
CsDof8	CAAT-box	860	865	common cis-acting element in promoter and enhancer regions
CsDof8	CAAT-box	867	872	common cis-acting element in promoter and enhancer regions
CsDof8	CAAT-box	912	917	common cis-acting element in promoter and enhancer regions
CsDof8	CAAT-box	936	941	common cis-acting element in promoter and enhancer regions
CsDof8	CAAT-box	1077	1082	common cis-acting element in promoter and enhancer regions
CsDof8	CAAT-box	1162	1167	common cis-acting element in promoter and enhancer regions
CsDof8	CAAT-box	1266	1271	common cis-acting element in promoter and enhancer regions
CsDof8	CAAT-box	1582	1587	common cis-acting element in promoter and enhancer regions
CsDof8	TATA-box	28	35	core promoter element around -30 of transcription start
CsDof8	TATA-box	217	223	core promoter element around -30 of transcription start
CsDof8	TATA-box	218	224	core promoter element around -30 of transcription start
CsDof8	TATA-box	219	225	core promoter element around -30 of transcription start
CsDof8	TATA-box	220	224	core promoter element around -30 of transcription start
CsDof8	TATA-box	288	292	core promoter element around -30 of transcription start
CsDof8	TATA-box	306	312	core promoter element around -30 of transcription start
CsDof8	TATA-box	307	312	core promoter element around -30 of transcription start
CsDof8	TATA-box	308	312	core promoter element around -30 of transcription start
CsDof8	TATA-box	322	328	core promoter element around -30 of transcription start
CsDof8	TATA-box	323	330	core promoter element around -30 of transcription start
CsDof8	TATA-box	324	330	core promoter element around -30 of transcription start
CsDof8	TATA-box	326	330	core promoter element around -30 of transcription start
CsDof8	TATA-box	356	362	core promoter element around -30 of transcription start
CsDof8	TATA-box	357	364	core promoter element around -30 of transcription start
CsDof8	TATA-box	358	364	core promoter element around -30 of transcription start
CsDof8	TATA-box	360	364	core promoter element around -30 of transcription start
CsDof8	TATA-box	380	386	core promoter element around -30 of transcription start
CsDof8	TATA-box	381	386	core promoter element around -30 of transcription start
CsDof8	TATA-box	382	386	core promoter element around -30 of transcription start
CsDof8	TATA-box	451	459	core promoter element around -30 of transcription start

Name	Cis-element	Start position	Stop position	Function
CsDof8	TATA-box	601	607	core promoter element around -30 of transcription start
CsDof8	TATA-box	602	606	core promoter element around -30 of transcription start
CsDof8	TATA-box	649	655	core promoter element around -30 of transcription start
CsDof8	TATA-box	650	654	core promoter element around -30 of transcription start
CsDof8	TATA-box	654	660	core promoter element around -30 of transcription start
CsDof8	TATA-box	655	661	core promoter element around -30 of transcription start
CsDof8	TATA-box	657	661	core promoter element around -30 of transcription start
CsDof8	TATA-box	752	756	core promoter element around -30 of transcription start
CsDof8	TATA-box	765	771	core promoter element around -30 of transcription start
CsDof8	TATA-box	766	772	core promoter element around -30 of transcription start
CsDof8	TATA-box	767	771	core promoter element around -30 of transcription start
CsDof8	TATA-box	920	926	core promoter element around -30 of transcription start
CsDof8	TATA-box	921	928	core promoter element around -30 of transcription start
CsDof8	TATA-box	922	928	core promoter element around -30 of transcription start
CsDof8	TATA-box	923	929	core promoter element around -30 of transcription start
CsDof8	TATA-box	924	930	core promoter element around -30 of transcription start
CsDof8	TATA-box	925	931	core promoter element around -30 of transcription start
CsDof8	TATA-box	926	930	core promoter element around -30 of transcription start
CsDof8	TATA-box	946	952	core promoter element around -30 of transcription start
CsDof8	TATA-box	947	951	core promoter element around -30 of transcription start
CsDof8	TATA-box	1058	1066	core promoter element around -30 of transcription start
CsDof8	TATA-box	1061	1068	core promoter element around -30 of transcription start
CsDof8	TATA-box	1062	1068	core promoter element around -30 of transcription start
CsDof8	TATA-box	1063	1069	core promoter element around -30 of transcription start
CsDof8	TATA-box	1064	1068	core promoter element around -30 of transcription start
CsDof8	TATA-box	1122	1128	core promoter element around -30 of transcription start
CsDof8	TATA-box	1123	1129	core promoter element around -30 of transcription start
CsDof8	TATA-box	1124	1130	core promoter element around -30 of transcription start
CsDof8	TATA-box	1125	1131	core promoter element around -30 of transcription start
CsDof8	TATA-box	1126	1130	core promoter element around -30 of transcription start
CsDof8	TATA-box	1132	1136	core promoter element around -30 of transcription start
CsDof8	TATA-box	1171	1177	core promoter element around -30 of transcription start
CsDof8	TATA-box	1173	1179	core promoter element around -30 of transcription start
CsDof8	TATA-box	1174	1180	core promoter element around -30 of transcription start
CsDof8	TATA-box	1175	1181	core promoter element around -30 of transcription start
CsDof8	TATA-box	1177	1181	core promoter element around -30 of transcription start
CsDof8	TATA-box	1190	1194	core promoter element around -30 of transcription start
CsDof8	TATA-box	1222	1231	core promoter element around -30 of transcription start
CsDof8	TATA-box	1223	1229	core promoter element around -30 of transcription start
CsDof8	TATA-box	1224	1228	core promoter element around -30 of transcription start
CsDof8	TATA-box	1302	1309	core promoter element around -30 of transcription start
CsDof8	TATA-box	1303	1309	core promoter element around -30 of transcription start
CsDof8	TATA-box	1304	1309	core promoter element around -30 of transcription start
CsDof8	TATA-box	1305	1309	core promoter element around -30 of transcription start
CsDof8	TATA-box	1416	1422	core promoter element around -30 of transcription start
CsDof8	TATA-box	1417	1423	core promoter element around -30 of transcription start
CsDof8	TATA-box	1418	1422	core promoter element around -30 of transcription start
CsDof8	TATA-box	1435	1439	core promoter element around -30 of transcription start
CsDof8	TATA-box	1521	1525	core promoter element around -30 of transcription start
CsDof8	TATA-box	1559	1563	core promoter element around -30 of transcription start
CsDof8	TATA-box	1610	1614	core promoter element around -30 of transcription start

Name	Cis-element	Start position	Stop position	Function
CsDof8	TATA-box	1718	1722	core promoter element around -30 of transcription start
CsDof8	TATA-box	1738	1746	core promoter element around -30 of transcription start
CsDof8	TATA-box	1743	1749	core promoter element around -30 of transcription start
CsDof8	TATA-box	1744	1750	core promoter element around -30 of transcription start
CsDof8	TATA-box	1745	1751	core promoter element around -30 of transcription start
CsDof8	TATA-box	1746	1750	core promoter element around -30 of transcription start
CsDof8	TATA-box	1789	1795	core promoter element around -30 of transcription start
CsDof8	TATA-box	1790	1796	core promoter element around -30 of transcription start
CsDof8	TATA-box	1791	1797	core promoter element around -30 of transcription start
CsDof8	TATA-box	1792	1798	core promoter element around -30 of transcription start
CsDof8	TATA-box	1793	1797	core promoter element around -30 of transcription start
CsDof8	TATA-box	1804	1808	core promoter element around -30 of transcription start
CsDof8	TATA-box	1809	1815	core promoter element around -30 of transcription start
CsDof8	TATA-box	1810	1814	core promoter element around -30 of transcription start
CsDof8	TATA-box	1821	1827	core promoter element around -30 of transcription start
CsDof8	TATA-box	1822	1828	core promoter element around -30 of transcription start
CsDof8	TATA-box	1823	1829	core promoter element around -30 of transcription start
CsDof8	TATA-box	1824	1828	core promoter element around -30 of transcription start
CsDof8	TATA-box	1846	1852	core promoter element around -30 of transcription start
CsDof8	TATA-box	1847	1853	core promoter element around -30 of transcription start
CsDof8	TATA-box	1848	1852	core promoter element around -30 of transcription start
CsDof8	TATA-box	1858	1862	core promoter element around -30 of transcription start
CsDof8	GARE-motif	1569	1576	gibberellin-responsive element
CsDof8	GT1-motif	199	205	light responsive element
CsDof8	MBS	295	301	MYB binding site involved in drought-inducibility
CsDof8	MBS	1291	1297	MYB binding site involved in drought-inducibility
CsDof8	MBSI	1227	1237.5	MYB binding site involved in flavonoid biosynthetic genes regulation
CsDof8	MRE	223	230	MYB binding site involved in light responsiveness
CsDof9	ABRE	1038	1043	abscisic acid responsiveness
CsDof9	ABRE	1414	1419	abscisic acid responsiveness
CsDof9	ABRE	1648	1653	abscisic acid responsiveness
CsDof9	TATC-box	467	474	cis-acting element involved in gibberellin-responsiveness
CsDof9	TCA-element	1442	1451	cis-acting element involved in salicylic acid responsiveness
CsDof9	A-box	380	386	cis-acting regulatory element
CsDof9	A-box	532	538	cis-acting regulatory element
CsDof9	AuxRR-core	329	336	cis-acting regulatory element involved in auxin responsiveness
CsDof9	AuxRR-core	1181	1188	cis-acting regulatory element involved in auxin responsiveness
CsDof9	G-box	1035	1044	cis-acting regulatory element involved in light responsiveness
CsDof9	G-box	1038	1044	cis-acting regulatory element involved in light responsiveness
CsDof9	G-box	1414	1420	cis-acting regulatory element involved in light responsiveness
CsDof9	G-Box	1647	1653	cis-acting regulatory element involved in light responsiveness
CsDof9	CGTCA-motif	388	393	cis-acting regulatory element involved in the MeJA-responsiveness
CsDof9	CGTCA-motif	1570	1575	cis-acting regulatory element involved in the MeJA-responsiveness
CsDof9	TGACG-motif	388	393	cis-acting regulatory element involved in the MeJA-responsiveness
CsDof9	TGACG-motif	1570	1575	cis-acting regulatory element involved in the MeJA-responsiveness
CsDof9	CAAT-box	265	270	common cis-acting element in promoter and enhancer regions
CsDof9	CAAT-box	306	311	common cis-acting element in promoter and enhancer regions
CsDof9	CAAT-box	314	319	common cis-acting element in promoter and enhancer regions
CsDof9	CAAT-box	495	500	common cis-acting element in promoter and enhancer regions
CsDof9	CAAT-box	601	606	common cis-acting element in promoter and enhancer regions
CsDof9	CAAT-box	637	646	common cis-acting element in promoter and enhancer regions

Name	Cis-element	Start position	Stop position	Function
CsDof9	CAAT-box	639	644	common cis-acting element in promoter and enhancer regions
CsDof9	CAAT-box	754	762	common cis-acting element in promoter and enhancer regions
CsDof9	CAAT-box	755	760	common cis-acting element in promoter and enhancer regions
CsDof9	CAAT-box	791	796	common cis-acting element in promoter and enhancer regions
CsDof9	CAAT-box	832	837	common cis-acting element in promoter and enhancer regions
CsDof9	CAAT-box	1368	1373	common cis-acting element in promoter and enhancer regions
CsDof9	CAAT-box	1599	1604	common cis-acting element in promoter and enhancer regions
CsDof9	CAAT-box	1656	1661	common cis-acting element in promoter and enhancer regions
CsDof9	TATA-box	3	8	core promoter element around -30 of transcription start
CsDof9	TATA-box	4	8	core promoter element around -30 of transcription start
CsDof9	TATA-box	76	82	core promoter element around -30 of transcription start
CsDof9	TATA-box	77	81	core promoter element around -30 of transcription start
CsDof9	TATA-box	160	166	core promoter element around -30 of transcription start
CsDof9	TATA-box	161	166	core promoter element around -30 of transcription start
CsDof9	TATA-box	162	166	core promoter element around -30 of transcription start
CsDof9	TATA-box	259	266	core promoter element around -30 of transcription start
CsDof9	TATA-box	671	675	core promoter element around -30 of transcription start
CsDof9	TATA-box	816	820	core promoter element around -30 of transcription start
CsDof9	TATA-box	937	946	core promoter element around -30 of transcription start
CsDof9	TATA-box	938	945	core promoter element around -30 of transcription start
CsDof9	TATA-box	939	945	core promoter element around -30 of transcription start
CsDof9	TATA-box	940	945	core promoter element around -30 of transcription start
CsDof9	TATA-box	941	945	core promoter element around -30 of transcription start
CsDof9	TATA-box	984	990	core promoter element around -30 of transcription start
CsDof9	TATA-box	985	989	core promoter element around -30 of transcription start
CsDof9	TATA-box	1008	1015	core promoter element around -30 of transcription start
CsDof9	TATA-box	1082	1089	core promoter element around -30 of transcription start
CsDof9	TATA-box	1083	1089	core promoter element around -30 of transcription start
CsDof9	TATA-box	1084	1089	core promoter element around -30 of transcription start
CsDof9	TATA-box	1085	1089	core promoter element around -30 of transcription start
CsDof9	TATA-box	1136	1140	core promoter element around -30 of transcription start
CsDof9	TATA-box	1186	1192	core promoter element around -30 of transcription start
CsDof9	TATA-box	1187	1191	core promoter element around -30 of transcription start
CsDof9	TATA-box	1273	1279	core promoter element around -30 of transcription start
CsDof9	TATA-box	1274	1279	core promoter element around -30 of transcription start
CsDof9	TATA-box	1275	1279	core promoter element around -30 of transcription start
CsDof9	TATA-box	1308	1312	core promoter element around -30 of transcription start
CsDof9	TATA-box	1320	1326	core promoter element around -30 of transcription start
CsDof9	TATA-box	1321	1326	core promoter element around -30 of transcription start
CsDof9	TATA-box	1322	1326	core promoter element around -30 of transcription start
CsDof9	TATA-box	1454	1462	core promoter element around -30 of transcription start
CsDof9	TATA-box	1630	1635	core promoter element around -30 of transcription start
CsDof9	TATA-box	1631	1635	core promoter element around -30 of transcription start
CsDof9	TATA-box	1747	1751	core promoter element around -30 of transcription start
CsDof9	GARE-motif	521	528	gibberellin-responsive element
CsDof9	P-box	1419	1426	gibberellin-responsive element
CsDof10	ABRE	179	184	abscisic acid responsiveness
CsDof10	ABRE	1061	1070	abscisic acid responsiveness
CsDof10	ABRE	1063	1068	abscisic acid responsiveness
CsDof10	ABRE	1068	1073	abscisic acid responsiveness
CsDof10	TGA-element	771	777	auxin-responsive element

Name	Cis-element	Start position	Stop position	Function
CsDof10	LTR	454	460	cis-acting element involved in low-temperature responsiveness
CsDof10	LTR	1710	1716	cis-acting element involved in low-temperature responsiveness
CsDof10	TCA-element	996	1006	cis-acting element involved in salicylic acid responsiveness
CsDof10	ARE	285	291	cis-acting regulatory element essential for the anaerobic induction
CsDof10	ARE	1288	1294	cis-acting regulatory element essential for the anaerobic induction
CsDof10	ARE	1338	1344	cis-acting regulatory element essential for the anaerobic induction
CsDof10	circadian	433	442	cis-acting regulatory element involved in circadian control
CsDof10	circadian	1041	1050	cis-acting regulatory element involved in circadian control
CsDof10	G-box	178	184	cis-acting regulatory element involved in light responsiveness
CsDof10	G-box	573	582	cis-acting regulatory element involved in light responsiveness
CsDof10	G-box	1063	1069	cis-acting regulatory element involved in light responsiveness
CsDof10	G-box	1067	1073	cis-acting regulatory element involved in light responsiveness
CsDof10	RY-element	321	329	cis-acting regulatory element involved in seed-specific regulation
CsDof10	CAAT-box	189	194	common cis-acting element in promoter and enhancer regions
CsDof10	CAAT-box	236	241	common cis-acting element in promoter and enhancer regions
CsDof10	CAAT-box	388	393	common cis-acting element in promoter and enhancer regions
CsDof10	CAAT-box	609	614	common cis-acting element in promoter and enhancer regions
CsDof10	CAAT-box	822	827	common cis-acting element in promoter and enhancer regions
CsDof10	CAAT-box	975	980	common cis-acting element in promoter and enhancer regions
CsDof10	CAAT-box	982	987	common cis-acting element in promoter and enhancer regions
CsDof10	CAAT-box	1028	1033	common cis-acting element in promoter and enhancer regions
CsDof10	CAAT-box	1156	1161	common cis-acting element in promoter and enhancer regions
CsDof10	CAAT-box	1281	1286	common cis-acting element in promoter and enhancer regions
CsDof10	CAAT-box	1631	1636	common cis-acting element in promoter and enhancer regions
CsDof10	CAAT-box	1776	1781	common cis-acting element in promoter and enhancer regions
CsDof10	CAAT-box	1909	1914	common cis-acting element in promoter and enhancer regions
CsDof10	TATA-box	1	5	core promoter element around -30 of transcription start
CsDof10	TATA-box	24	28	core promoter element around -30 of transcription start
CsDof10	TATA-box	57	64	core promoter element around -30 of transcription start
CsDof10	TATA-box	58	64	core promoter element around -30 of transcription start
CsDof10	TATA-box	59	64	core promoter element around -30 of transcription start
CsDof10	TATA-box	60	64	core promoter element around -30 of transcription start
CsDof10	TATA-box	107	115	core promoter element around -30 of transcription start
CsDof10	TATA-box	108	115	core promoter element around -30 of transcription start
CsDof10	TATA-box	109	115	core promoter element around -30 of transcription start
CsDof10	TATA-box	110	115	core promoter element around -30 of transcription start
CsDof10	TATA-box	111	115	core promoter element around -30 of transcription start
CsDof10	TATA-box	124	130	core promoter element around -30 of transcription start
CsDof10	TATA-box	126	130	core promoter element around -30 of transcription start
CsDof10	TATA-box	138	142	core promoter element around -30 of transcription start
CsDof10	TATA-box	294	300	core promoter element around -30 of transcription start
CsDof10	TATA-box	295	301	core promoter element around -30 of transcription start
CsDof10	TATA-box	296	300	core promoter element around -30 of transcription start
CsDof10	TATA-box	412	417	core promoter element around -30 of transcription start
CsDof10	TATA-box	413	417	core promoter element around -30 of transcription start
CsDof10	TATA-box	473	480	core promoter element around -30 of transcription start
CsDof10	TATA-box	474	480	core promoter element around -30 of transcription start
CsDof10	TATA-box	475	480	core promoter element around -30 of transcription start
CsDof10	TATA-box	476	480	core promoter element around -30 of transcription start
CsDof10	TATA-box	753	757	core promoter element around -30 of transcription start
CsDof10	TATA-box	835	842	core promoter element around -30 of transcription start

Name	Cis-element	Start position	Stop position	Function
CsDof10	TATA-box	836	842	core promoter element around -30 of transcription start
CsDof10	TATA-box	837	842	core promoter element around -30 of transcription start
CsDof10	TATA-box	838	842	core promoter element around -30 of transcription start
CsDof10	TATA-box	931	937	core promoter element around -30 of transcription start
CsDof10	TATA-box	932	937	core promoter element around -30 of transcription start
CsDof10	TATA-box	933	937	core promoter element around -30 of transcription start
CsDof10	TATA-box	985	991	core promoter element around -30 of transcription start
CsDof10	TATA-box	986	990	core promoter element around -30 of transcription start
CsDof10	TATA-box	1053	1057	core promoter element around -30 of transcription start
CsDof10	TATA-box	1131	1137	core promoter element around -30 of transcription start
CsDof10	TATA-box	1132	1137	core promoter element around -30 of transcription start
CsDof10	TATA-box	1133	1137	core promoter element around -30 of transcription start
CsDof10	TATA-box	1181	1187	core promoter element around -30 of transcription start
CsDof10	TATA-box	1182	1187	core promoter element around -30 of transcription start
CsDof10	TATA-box	1183	1187	core promoter element around -30 of transcription start
CsDof10	TATA-box	1268	1272	core promoter element around -30 of transcription start
CsDof10	TATA-box	1274	1281	core promoter element around -30 of transcription start
CsDof10	TATA-box	1289	1298	core promoter element around -30 of transcription start
CsDof10	TATA-box	1291	1297	core promoter element around -30 of transcription start
CsDof10	TATA-box	1292	1299	core promoter element around -30 of transcription start
CsDof10	TATA-box	1293	1299	core promoter element around -30 of transcription start
CsDof10	TATA-box	1294	1300	core promoter element around -30 of transcription start
CsDof10	TATA-box	1295	1299	core promoter element around -30 of transcription start
CsDof10	TATA-box	1414	1421	core promoter element around -30 of transcription start
CsDof10	TATA-box	1415	1421	core promoter element around -30 of transcription start
CsDof10	TATA-box	1416	1421	core promoter element around -30 of transcription start
CsDof10	TATA-box	1417	1421	core promoter element around -30 of transcription start
CsDof10	TATA-box	1445	1450	core promoter element around -30 of transcription start
CsDof10	TATA-box	1446	1450	core promoter element around -30 of transcription start
CsDof10	TATA-box	1451	1458	core promoter element around -30 of transcription start
CsDof10	TATA-box	1457	1463	core promoter element around -30 of transcription start
CsDof10	TATA-box	1458	1464	core promoter element around -30 of transcription start
CsDof10	TATA-box	1459	1465	core promoter element around -30 of transcription start
CsDof10	TATA-box	1460	1464	core promoter element around -30 of transcription start
CsDof10	TATA-box	1490	1495	core promoter element around -30 of transcription start
CsDof10	TATA-box	1491	1495	core promoter element around -30 of transcription start
CsDof10	TATA-box	1503	1507	core promoter element around -30 of transcription start
CsDof10	TATA-box	1527	1533	core promoter element around -30 of transcription start
CsDof10	TATA-box	1528	1534	core promoter element around -30 of transcription start
CsDof10	TATA-box	1529	1535	core promoter element around -30 of transcription start
CsDof10	TATA-box	1530	1536	core promoter element around -30 of transcription start
CsDof10	TATA-box	1531	1535	core promoter element around -30 of transcription start
CsDof10	TATA-box	1564	1569	core promoter element around -30 of transcription start
CsDof10	TATA-box	1565	1569	core promoter element around -30 of transcription start
CsDof10	TATA-box	1640	1644	core promoter element around -30 of transcription start
CsDof10	TATA-box	1652	1656	core promoter element around -30 of transcription start
CsDof10	TATA-box	1678	1684	core promoter element around -30 of transcription start
CsDof10	TATA-box	1679	1684	core promoter element around -30 of transcription start
CsDof10	TATA-box	1680	1684	core promoter element around -30 of transcription start
CsDof10	TATA-box	1687	1693	core promoter element around -30 of transcription start
CsDof10	TATA-box	1688	1694	core promoter element around -30 of transcription start

Name	Cis-element	Start position	Stop position	Function
CsDof10	TATA-box	1690	1694	core promoter element around -30 of transcription start
CsDof10	3-AF1 binding site	1551	1561	light responsive element
CsDof10	3-AF1 binding site	1857	1867	light responsive element
CsDof11	ABRE	741	747	abscisic acid responsiveness
CsDof11	ABRE	742	747	abscisic acid responsiveness
CsDof11	ABRE	1796	1801	abscisic acid responsiveness
CsDof11	TC-rich repeats	636	645	cis-acting element involved in defense and stress responsiveness
CsDof11	ACE	631	640	cis-acting element involved in light responsiveness
CsDof11	LTR	404	410	cis-acting element involved in low-temperature responsiveness
CsDof11	TCA-element	1551	1560	cis-acting element involved in salicylic acid responsiveness
CsDof11	G-Box	741	747	cis-acting regulatory element involved in light responsiveness
CsDof11	G-Box	1796	1802	cis-acting regulatory element involved in light responsiveness
CsDof11	G-box	741	747	cis-acting regulatory element involved in light responsiveness
CsDof11	G-box	1796	1804	cis-acting regulatory element involved in light responsiveness
CsDof11	CGTCA-motif	777	782	cis-acting regulatory element involved in the MeJA-responsiveness
CsDof11	CGTCA-motif	905	910	cis-acting regulatory element involved in the MeJA-responsiveness
CsDof11	TGACG-motif	777	782	cis-acting regulatory element involved in the MeJA-responsiveness
CsDof11	TGACG-motif	905	910	cis-acting regulatory element involved in the MeJA-responsiveness
CsDof11	CAAT-box	91	96	common cis-acting element in promoter and enhancer regions
CsDof11	CAAT-box	360	365	common cis-acting element in promoter and enhancer regions
CsDof11	CAAT-box	399	404	common cis-acting element in promoter and enhancer regions
CsDof11	CAAT-box	505	510	common cis-acting element in promoter and enhancer regions
CsDof11	CAAT-box	546	551	common cis-acting element in promoter and enhancer regions
CsDof11	CAAT-box	687	692	common cis-acting element in promoter and enhancer regions
CsDof11	CAAT-box	848	853	common cis-acting element in promoter and enhancer regions
CsDof11	CAAT-box	866	871	common cis-acting element in promoter and enhancer regions
CsDof11	CAAT-box	931	936	common cis-acting element in promoter and enhancer regions
CsDof11	CAAT-box	1046	1051	common cis-acting element in promoter and enhancer regions
CsDof11	CAAT-box	1171	1176	common cis-acting element in promoter and enhancer regions
CsDof11	CAAT-box	1454	1459	common cis-acting element in promoter and enhancer regions
CsDof11	CAAT-box	1563	1568	common cis-acting element in promoter and enhancer regions
CsDof11	CAAT-box	1590	1595	common cis-acting element in promoter and enhancer regions
CsDof11	CAAT-box	1697	1702	common cis-acting element in promoter and enhancer regions
CsDof11	CAAT-box	1733	1738	common cis-acting element in promoter and enhancer regions
CsDof11	CAAT-box	1954	1959	common cis-acting element in promoter and enhancer regions
CsDof11	TATA-box	20	27	core promoter element around -30 of transcription start
CsDof11	TATA-box	21	27	core promoter element around -30 of transcription start
CsDof11	TATA-box	22	27	core promoter element around -30 of transcription start
CsDof11	TATA-box	23	27	core promoter element around -30 of transcription start
CsDof11	TATA-box	256	262	core promoter element around -30 of transcription start
CsDof11	TATA-box	257	262	core promoter element around -30 of transcription start
CsDof11	TATA-box	258	262	core promoter element around -30 of transcription start
CsDof11	TATA-box	297	304	core promoter element around -30 of transcription start
CsDof11	TATA-box	298	304	core promoter element around -30 of transcription start
CsDof11	TATA-box	299	305	core promoter element around -30 of transcription start
CsDof11	TATA-box	300	304	core promoter element around -30 of transcription start
CsDof11	TATA-box	312	318	core promoter element around -30 of transcription start
CsDof11	TATA-box	313	318	core promoter element around -30 of transcription start
CsDof11	TATA-box	314	318	core promoter element around -30 of transcription start
CsDof11	TATA-box	329	336	core promoter element around -30 of transcription start
CsDof11	TATA-box	330	336	core promoter element around -30 of transcription start

Name	Cis-element	Start position	Stop position	Function
CsDof11	TATA-box	331	336	core promoter element around -30 of transcription start
CsDof11	TATA-box	332	336	core promoter element around -30 of transcription start
CsDof11	TATA-box	447	454	core promoter element around -30 of transcription start
CsDof11	TATA-box	600	605	core promoter element around -30 of transcription start
CsDof11	TATA-box	601	605	core promoter element around -30 of transcription start
CsDof11	TATA-box	799	803	core promoter element around -30 of transcription start
CsDof11	TATA-box	934	940	core promoter element around -30 of transcription start
CsDof11	TATA-box	935	941	core promoter element around -30 of transcription start
CsDof11	TATA-box	937	941	core promoter element around -30 of transcription start
CsDof11	TATA-box	961	967	core promoter element around -30 of transcription start
CsDof11	TATA-box	962	969	core promoter element around -30 of transcription start
CsDof11	TATA-box	963	969	core promoter element around -30 of transcription start
CsDof11	TATA-box	964	970	core promoter element around -30 of transcription start
CsDof11	TATA-box	965	969	core promoter element around -30 of transcription start
CsDof11	TATA-box	975	979	core promoter element around -30 of transcription start
CsDof11	TATA-box	998	1004	core promoter element around -30 of transcription start
CsDof11	TATA-box	1000	1006	core promoter element around -30 of transcription start
CsDof11	TATA-box	1001	1007	core promoter element around -30 of transcription start
CsDof11	TATA-box	1002	1006	core promoter element around -30 of transcription start
CsDof11	TATA-box	1021	1025	core promoter element around -30 of transcription start
CsDof11	TATA-box	1268	1274	core promoter element around -30 of transcription start
CsDof11	TATA-box	1269	1273	core promoter element around -30 of transcription start
CsDof11	TATA-box	1312	1318	core promoter element around -30 of transcription start
CsDof11	TATA-box	1313	1318	core promoter element around -30 of transcription start
CsDof11	TATA-box	1314	1318	core promoter element around -30 of transcription start
CsDof11	TATA-box	1502	1507	core promoter element around -30 of transcription start
CsDof11	TATA-box	1503	1507	core promoter element around -30 of transcription start
CsDof11	TATA-box	1515	1523	core promoter element around -30 of transcription start
CsDof11	TATA-box	1703	1709	core promoter element around -30 of transcription start
CsDof11	TATA-box	1704	1711	core promoter element around -30 of transcription start
CsDof11	TATA-box	1705	1711	core promoter element around -30 of transcription start
CsDof11	TATA-box	1706	1712	core promoter element around -30 of transcription start
CsDof11	TATA-box	1707	1711	core promoter element around -30 of transcription start
CsDof11	TATA-box	1888	1894	core promoter element around -30 of transcription start
CsDof11	TATA-box	1889	1895	core promoter element around -30 of transcription start
CsDof11	TATA-box	1890	1896	core promoter element around -30 of transcription start
CsDof11	TATA-box	1891	1895	core promoter element around -30 of transcription start
CsDof11	TATA-box	1916	1920	core promoter element around -30 of transcription start
CsDof11	Sp1	1791	1797	light responsive element
CsDof11	MBSI	1657	1667.5	MYB binding site involved in flavonoid biosynthetic genes regulation
CsDof12	ABRE	112	117	abscisic acid responsiveness
CsDof12	ABRE	214	219	abscisic acid responsiveness
CsDof12	ABRE	396	401	abscisic acid responsiveness
CsDof12	ABRE	1685	1690	abscisic acid responsiveness
CsDof12	ABRE	1829	1834	abscisic acid responsiveness
CsDof12	LTR	245	251	cis-acting element involved in low-temperature responsiveness
CsDof12	TCA-element	1005	1014	cis-acting element involved in salicylic acid responsiveness
CsDof12	TCA-element	1899	1908	cis-acting element involved in salicylic acid responsiveness
CsDof12	SARE	1008	1019	cis-acting element involved in salicylic acid responsiveness
CsDof12	ARE	728	734	cis-acting regulatory element essential for the anaerobic induction
CsDof12	ARE	819	825	cis-acting regulatory element essential for the anaerobic induction

Name	Cis-element	Start position	Stop position	Function
CsDof12	ARE	975	981	cis-acting regulatory element essential for the anaerobic induction
CsDof12	ARE	1237	1243	cis-acting regulatory element essential for the anaerobic induction
CsDof12	AuxRR-core	411	418	cis-acting regulatory element involved in auxin responsiveness
CsDof12	G-box	111	117	cis-acting regulatory element involved in light responsiveness
CsDof12	G-box	213	219	cis-acting regulatory element involved in light responsiveness
CsDof12	G-box	396	402	cis-acting regulatory element involved in light responsiveness
CsDof12	G-box	1684	1690	cis-acting regulatory element involved in light responsiveness
CsDof12	G-box	1828	1834	cis-acting regulatory element involved in light responsiveness
CsDof12	RY-element	1278	1286	cis-acting regulatory element involved in seed-specific regulation
CsDof12	CGTCA-motif	212	217	cis-acting regulatory element involved in the MeJA-responsiveness
CsDof12	CGTCA-motif	1683	1688	cis-acting regulatory element involved in the MeJA-responsiveness
CsDof12	TGACG-motif	212	217	cis-acting regulatory element involved in the MeJA-responsiveness
CsDof12	TGACG-motif	1683	1688	cis-acting regulatory element involved in the MeJA-responsiveness
CsDof12	O2-site	210	219	cis-acting regulatory element involved in zein metabolism regulation
CsDof12	CAT-box	522	528	cis-acting regulatory element related to meristem expression
CsDof12	CAT-box	1719	1725	cis-acting regulatory element related to meristem expression
CsDof12	CAAT-box	38	43	common cis-acting element in promoter and enhancer regions
CsDof12	CAAT-box	83	88	common cis-acting element in promoter and enhancer regions
CsDof12	CAAT-box	136	141	common cis-acting element in promoter and enhancer regions
CsDof12	CAAT-box	281	286	common cis-acting element in promoter and enhancer regions
CsDof12	CAAT-box	310	315	common cis-acting element in promoter and enhancer regions
CsDof12	CAAT-box	347	352	common cis-acting element in promoter and enhancer regions
CsDof12	CAAT-box	434	439	common cis-acting element in promoter and enhancer regions
CsDof12	CAAT-box	666	671	common cis-acting element in promoter and enhancer regions
CsDof12	CAAT-box	669	674	common cis-acting element in promoter and enhancer regions
CsDof12	CAAT-box	791	796	common cis-acting element in promoter and enhancer regions
CsDof12	CAAT-box	796	801	common cis-acting element in promoter and enhancer regions
CsDof12	CAAT-box	874	879	common cis-acting element in promoter and enhancer regions
CsDof12	CAAT-box	1016	1021	common cis-acting element in promoter and enhancer regions
CsDof12	CAAT-box	1408	1413	common cis-acting element in promoter and enhancer regions
CsDof12	CAAT-box	1460	1465	common cis-acting element in promoter and enhancer regions
CsDof12	CAAT-box	1475	1483	common cis-acting element in promoter and enhancer regions
CsDof12	CAAT-box	1476	1481	common cis-acting element in promoter and enhancer regions
CsDof12	CAAT-box	1580	1585	common cis-acting element in promoter and enhancer regions
CsDof12	CAAT-box	1676	1681	common cis-acting element in promoter and enhancer regions
CsDof12	CAAT-box	1708	1713	common cis-acting element in promoter and enhancer regions
CsDof12	CAAT-box	1931	1936	common cis-acting element in promoter and enhancer regions
CsDof12	CAAT-box	1958	1963	common cis-acting element in promoter and enhancer regions
CsDof12	TATA-box	3	10	core promoter element around -30 of transcription start
CsDof12	TATA-box	4	10	core promoter element around -30 of transcription start
CsDof12	TATA-box	5	10	core promoter element around -30 of transcription start
CsDof12	TATA-box	6	10	core promoter element around -30 of transcription start
CsDof12	TATA-box	34	40	core promoter element around -30 of transcription start
CsDof12	TATA-box	35	39	core promoter element around -30 of transcription start
CsDof12	TATA-box	326	333	core promoter element around -30 of transcription start
CsDof12	TATA-box	429	433	core promoter element around -30 of transcription start
CsDof12	TATA-box	538	544	core promoter element around -30 of transcription start
CsDof12	TATA-box	540	544	core promoter element around -30 of transcription start
CsDof12	TATA-box	851	860	core promoter element around -30 of transcription start
CsDof12	TATA-box	852	859	core promoter element around -30 of transcription start
CsDof12	TATA-box	853	859	core promoter element around -30 of transcription start

Name	Cis-element	Start position	Stop position	Function
CsDof12	TATA-box	854	859	core promoter element around -30 of transcription start
CsDof12	TATA-box	855	859	core promoter element around -30 of transcription start
CsDof12	TATA-box	923	931	core promoter element around -30 of transcription start
CsDof12	TATA-box	1003	1009	core promoter element around -30 of transcription start
CsDof12	TATA-box	1004	1008	core promoter element around -30 of transcription start
CsDof12	TATA-box	1045	1049	core promoter element around -30 of transcription start
CsDof12	TATA-box	1121	1128	core promoter element around -30 of transcription start
CsDof12	TATA-box	1122	1128	core promoter element around -30 of transcription start
CsDof12	TATA-box	1123	1128	core promoter element around -30 of transcription start
CsDof12	TATA-box	1124	1128	core promoter element around -30 of transcription start
CsDof12	TATA-box	1126	1134	core promoter element around -30 of transcription start
CsDof12	TATA-box	1127	1134	core promoter element around -30 of transcription start
CsDof12	TATA-box	1128	1134	core promoter element around -30 of transcription start
CsDof12	TATA-box	1129	1134	core promoter element around -30 of transcription start
CsDof12	TATA-box	1130	1134	core promoter element around -30 of transcription start
CsDof12	TATA-box	1146	1154	core promoter element around -30 of transcription start
CsDof12	TATA-box	1217	1226	core promoter element around -30 of transcription start
CsDof12	TATA-box	1218	1225	core promoter element around -30 of transcription start
CsDof12	TATA-box	1219	1225	core promoter element around -30 of transcription start
CsDof12	TATA-box	1220	1227	core promoter element around -30 of transcription start
CsDof12	TATA-box	1221	1227	core promoter element around -30 of transcription start
CsDof12	TATA-box	1222	1228	core promoter element around -30 of transcription start
CsDof12	TATA-box	1223	1227	core promoter element around -30 of transcription start
CsDof12	TATA-box	1376	1382	core promoter element around -30 of transcription start
CsDof12	TATA-box	1377	1383	core promoter element around -30 of transcription start
CsDof12	TATA-box	1378	1384	core promoter element around -30 of transcription start
CsDof12	TATA-box	1379	1385	core promoter element around -30 of transcription start
CsDof12	TATA-box	1380	1386	core promoter element around -30 of transcription start
CsDof12	TATA-box	1381	1385	core promoter element around -30 of transcription start
CsDof12	TATA-box	1510	1514	core promoter element around -30 of transcription start
CsDof12	TATA-box	1515	1519	core promoter element around -30 of transcription start
CsDof12	TATA-box	1523	1527	core promoter element around -30 of transcription start
CsDof12	TATA-box	1556	1560	core promoter element around -30 of transcription start
CsDof12	TATA-box	1595	1602	core promoter element around -30 of transcription start
CsDof12	TATA-box	1903	1911	core promoter element around -30 of transcription start
CsDof12	TATA-box	1906	1911	core promoter element around -30 of transcription start
CsDof12	TATA-box	1907	1911	core promoter element around -30 of transcription start
CsDof12	P-box	464	471	gibberellin-responsive element
CsDof12	MBS	596	602	MYB binding site involved in drought-inducibility
CsDof12	MRE	1252	1259	MYB binding site involved in light responsiveness
CsDof13	ABRE	207	216	abscisic acid responsiveness
CsDof13	ABRE	210	215	abscisic acid responsiveness
CsDof13	ABRE	1190	1195	abscisic acid responsiveness
CsDof13	ABRE	1209	1214	abscisic acid responsiveness
CsDof13	ACE	1770	1779	cis-acting element involved in light responsiveness
CsDof13	TCA-element	1889	1898	cis-acting element involved in salicylic acid responsiveness
CsDof13	ARE	1531	1537	cis-acting regulatory element essential for the anaerobic induction
CsDof13	G-box	209	215	cis-acting regulatory element involved in light responsiveness
CsDof13	G-box	1190	1196	cis-acting regulatory element involved in light responsiveness
CsDof13	G-Box	1209	1215	cis-acting regulatory element involved in light responsiveness
CsDof13	O2-site	654	663	cis-acting regulatory element involved in zein metabolism regulation

Name	Cis-element	Start position	Stop position	Function
CsDof13	CAT-box	1482	1488	cis-acting regulatory element related to meristem expression
CsDof13	CAAT-box	48	53	common cis-acting element in promoter and enhancer regions
CsDof13	CAAT-box	267	272	common cis-acting element in promoter and enhancer regions
CsDof13	CAAT-box	332	337	common cis-acting element in promoter and enhancer regions
CsDof13	CAAT-box	344	349	common cis-acting element in promoter and enhancer regions
CsDof13	CAAT-box	788	793	common cis-acting element in promoter and enhancer regions
CsDof13	CAAT-box	1013	1018	common cis-acting element in promoter and enhancer regions
CsDof13	CAAT-box	1140	1145	common cis-acting element in promoter and enhancer regions
CsDof13	CAAT-box	1166	1171	common cis-acting element in promoter and enhancer regions
CsDof13	CAAT-box	1204	1209	common cis-acting element in promoter and enhancer regions
CsDof13	CAAT-box	1433	1438	common cis-acting element in promoter and enhancer regions
CsDof13	CAAT-box	1535	1540	common cis-acting element in promoter and enhancer regions
CsDof13	CAAT-box	1874	1879	common cis-acting element in promoter and enhancer regions
CsDof13	TATA-box	31	37	core promoter element around -30 of transcription start
CsDof13	TATA-box	33	37	core promoter element around -30 of transcription start
CsDof13	TATA-box	38	42	core promoter element around -30 of transcription start
CsDof13	TATA-box	64	68	core promoter element around -30 of transcription start
CsDof13	TATA-box	69	73	core promoter element around -30 of transcription start
CsDof13	TATA-box	198	206	core promoter element around -30 of transcription start
CsDof13	TATA-box	226	234	core promoter element around -30 of transcription start
CsDof13	TATA-box	237	245	core promoter element around -30 of transcription start
CsDof13	TATA-box	435	439	core promoter element around -30 of transcription start
CsDof13	TATA-box	447	451	core promoter element around -30 of transcription start
CsDof13	TATA-box	557	562	core promoter element around -30 of transcription start
CsDof13	TATA-box	558	562	core promoter element around -30 of transcription start
CsDof13	TATA-box	612	616	core promoter element around -30 of transcription start
CsDof13	TATA-box	850	856	core promoter element around -30 of transcription start
CsDof13	TATA-box	851	856	core promoter element around -30 of transcription start
CsDof13	TATA-box	852	856	core promoter element around -30 of transcription start
CsDof13	TATA-box	861	869	core promoter element around -30 of transcription start
CsDof13	TATA-box	872	878	core promoter element around -30 of transcription start
CsDof13	TATA-box	873	877	core promoter element around -30 of transcription start
CsDof13	TATA-box	1002	1008	core promoter element around -30 of transcription start
CsDof13	TATA-box	1003	1009	core promoter element around -30 of transcription start
CsDof13	TATA-box	1005	1009	core promoter element around -30 of transcription start
CsDof13	TATA-box	1017	1025	core promoter element around -30 of transcription start
CsDof13	TATA-box	1018	1025	core promoter element around -30 of transcription start
CsDof13	TATA-box	1019	1025	core promoter element around -30 of transcription start
CsDof13	TATA-box	1020	1025	core promoter element around -30 of transcription start
CsDof13	TATA-box	1021	1025	core promoter element around -30 of transcription start
CsDof13	TATA-box	1101	1105	core promoter element around -30 of transcription start
CsDof13	TATA-box	1108	1112	core promoter element around -30 of transcription start
CsDof13	TATA-box	1286	1293	core promoter element around -30 of transcription start
CsDof13	TATA-box	1287	1293	core promoter element around -30 of transcription start
CsDof13	TATA-box	1288	1294	core promoter element around -30 of transcription start
CsDof13	TATA-box	1289	1293	core promoter element around -30 of transcription start
CsDof13	TATA-box	1321	1327	core promoter element around -30 of transcription start
CsDof13	TATA-box	1322	1327	core promoter element around -30 of transcription start
CsDof13	TATA-box	1323	1327	core promoter element around -30 of transcription start
CsDof13	TATA-box	1538	1544	core promoter element around -30 of transcription start
CsDof13	TATA-box	1539	1545	core promoter element around -30 of transcription start

Name	Cis-element	Start position	Stop position	Function
CsDof13	TATA-box	1540	1546	core promoter element around -30 of transcription start
CsDof13	TATA-box	1541	1545	core promoter element around -30 of transcription start
CsDof13	TATA-box	1620	1624	core promoter element around -30 of transcription start
CsDof13	TATA-box	1627	1631	core promoter element around -30 of transcription start
CsDof13	TATA-box	1640	1644	core promoter element around -30 of transcription start
CsDof13	TATA-box	1722	1728	core promoter element around -30 of transcription start
CsDof13	TATA-box	1723	1729	core promoter element around -30 of transcription start
CsDof13	TATA-box	1724	1730	core promoter element around -30 of transcription start
CsDof13	TATA-box	1725	1729	core promoter element around -30 of transcription start
CsDof13	TATA-box	1792	1799	core promoter element around -30 of transcription start
CsDof13	TATA-box	1793	1799	core promoter element around -30 of transcription start
CsDof13	TATA-box	1794	1799	core promoter element around -30 of transcription start
CsDof13	TATA-box	1795	1799	core promoter element around -30 of transcription start
CsDof13	TATA-box	1849	1855	core promoter element around -30 of transcription start
CsDof13	TATA-box	1850	1854	core promoter element around -30 of transcription start
CsDof13	TATA-box	1885	1892	core promoter element around -30 of transcription start
CsDof13	TATA-box	1907	1915	core promoter element around -30 of transcription start
CsDof13	TATA-box	1910	1915	core promoter element around -30 of transcription start
CsDof13	TATA-box	1911	1915	core promoter element around -30 of transcription start
CsDof13	P-box	1514	1521	gibberellin-responsive element
CsDof13	AAAC-motif	1124	1135	light responsive element
CsDof13	MBS	1418	1424	MYB binding site involved in drought-inducibility
CsDof13	MRE	1122	1129	MYB binding site involved in light responsiveness
CsDof14	ABRE	1217	1222	abscisic acid responsiveness
CsDof14	TGA-element	1619	1625	auxin-responsive element
CsDof14	TGA-element	1973	1979	auxin-responsive element
CsDof14	AT-rich element	691	701	binding site of AT-rich DNA binding protein (ATBP-1)
CsDof14	AT-rich element	1409	1419	binding site of AT-rich DNA binding protein (ATBP-1)
CsDof14	ARE	221	227	cis-acting regulatory element essential for the anaerobic induction
CsDof14	ARE	1031	1037	cis-acting regulatory element essential for the anaerobic induction
CsDof14	ARE	1250	1256	cis-acting regulatory element essential for the anaerobic induction
CsDof14	ARE	1893	1899	cis-acting regulatory element essential for the anaerobic induction
CsDof14	circadian	1411	1420	cis-acting regulatory element involved in circadian control
CsDof14	G-box	1213	1219	cis-acting regulatory element involved in light responsiveness
CsDof14	G-box	1216	1222	cis-acting regulatory element involved in light responsiveness
CsDof14	TGACG-motif	1142	1147	cis-acting regulatory element involved in the MeJA-responsiveness
CsDof14	TGACG-motif	1863	1868	cis-acting regulatory element involved in the MeJA-responsiveness
CsDof14	CGTCA-motif	1142	1147	cis-acting regulatory element involved in the MeJA-responsiveness
CsDof14	CGTCA-motif	1863	1868	cis-acting regulatory element involved in the MeJA-responsiveness
CsDof14	O2-site	116	124	cis-acting regulatory element involved in zein metabolism regulation
CsDof14	CAT-box	1076	1082	cis-acting regulatory element related to meristem expression
CsDof14	CAAT-box	38	43	common cis-acting element in promoter and enhancer regions
CsDof14	CAAT-box	117	122	common cis-acting element in promoter and enhancer regions
CsDof14	CAAT-box	249	254	common cis-acting element in promoter and enhancer regions
CsDof14	CAAT-box	290	295	common cis-acting element in promoter and enhancer regions
CsDof14	CAAT-box	465	470	common cis-acting element in promoter and enhancer regions
CsDof14	CAAT-box	592	597	common cis-acting element in promoter and enhancer regions
CsDof14	CAAT-box	659	664	common cis-acting element in promoter and enhancer regions
CsDof14	CAAT-box	694	699	common cis-acting element in promoter and enhancer regions
CsDof14	CAAT-box	711	720	common cis-acting element in promoter and enhancer regions
CsDof14	CAAT-box	713	718	common cis-acting element in promoter and enhancer regions

Name	Cis-element	Start position	Stop position	Function
CsDof14	CAAT-box	881	886	common cis-acting element in promoter and enhancer regions
CsDof14	CAAT-box	1026	1031	common cis-acting element in promoter and enhancer regions
CsDof14	CAAT-box	1059	1064	common cis-acting element in promoter and enhancer regions
CsDof14	CAAT-box	1312	1317	common cis-acting element in promoter and enhancer regions
CsDof14	CAAT-box	1325	1330	common cis-acting element in promoter and enhancer regions
CsDof14	CAAT-box	1387	1392	common cis-acting element in promoter and enhancer regions
CsDof14	CAAT-box	1407	1412	common cis-acting element in promoter and enhancer regions
CsDof14	CAAT-box	1433	1438	common cis-acting element in promoter and enhancer regions
CsDof14	CAAT-box	1493	1498	common cis-acting element in promoter and enhancer regions
CsDof14	CAAT-box	1728	1733	common cis-acting element in promoter and enhancer regions
CsDof14	CAAT-box	1818	1823	common cis-acting element in promoter and enhancer regions
CsDof14	CAAT-box	1956	1961	common cis-acting element in promoter and enhancer regions
CsDof14	TATA-box	99	105	core promoter element around -30 of transcription start
CsDof14	TATA-box	101	105	core promoter element around -30 of transcription start
CsDof14	TATA-box	137	143	core promoter element around -30 of transcription start
CsDof14	TATA-box	139	145	core promoter element around -30 of transcription start
CsDof14	TATA-box	141	145	core promoter element around -30 of transcription start
CsDof14	TATA-box	266	270	core promoter element around -30 of transcription start
CsDof14	TATA-box	426	432	core promoter element around -30 of transcription start
CsDof14	TATA-box	427	433	core promoter element around -30 of transcription start
CsDof14	TATA-box	428	432	core promoter element around -30 of transcription start
CsDof14	TATA-box	727	733	core promoter element around -30 of transcription start
CsDof14	TATA-box	729	733	core promoter element around -30 of transcription start
CsDof14	TATA-box	856	861	core promoter element around -30 of transcription start
CsDof14	TATA-box	857	861	core promoter element around -30 of transcription start
CsDof14	TATA-box	1368	1373	core promoter element around -30 of transcription start
CsDof14	TATA-box	1369	1373	core promoter element around -30 of transcription start
CsDof14	TATA-box	1428	1434	core promoter element around -30 of transcription start
CsDof14	TATA-box	1429	1434	core promoter element around -30 of transcription start
CsDof14	TATA-box	1430	1434	core promoter element around -30 of transcription start
CsDof14	TATA-box	1456	1462	core promoter element around -30 of transcription start
CsDof14	TATA-box	1457	1462	core promoter element around -30 of transcription start
CsDof14	TATA-box	1458	1462	core promoter element around -30 of transcription start
CsDof14	TATA-box	1498	1504	core promoter element around -30 of transcription start
CsDof14	TATA-box	1499	1503	core promoter element around -30 of transcription start
CsDof14	TATA-box	1522	1528	core promoter element around -30 of transcription start
CsDof14	TATA-box	1523	1528	core promoter element around -30 of transcription start
CsDof14	TATA-box	1524	1528	core promoter element around -30 of transcription start
CsDof14	TATA-box	1543	1551	core promoter element around -30 of transcription start
CsDof14	TATA-box	1549	1553	core promoter element around -30 of transcription start
CsDof14	TATA-box	1614	1619	core promoter element around -30 of transcription start
CsDof14	TATA-box	1615	1619	core promoter element around -30 of transcription start
CsDof14	TATA-box	1731	1737	core promoter element around -30 of transcription start
CsDof14	TATA-box	1732	1737	core promoter element around -30 of transcription start
CsDof14	TATA-box	1733	1737	core promoter element around -30 of transcription start
CsDof14	TATA-box	1837	1843	core promoter element around -30 of transcription start
CsDof14	TATA-box	1838	1842	core promoter element around -30 of transcription start
CsDof14	TATA-box	1886	1892	core promoter element around -30 of transcription start
CsDof14	TATA-box	1887	1893	core promoter element around -30 of transcription start
CsDof14	TATA-box	1888	1892	core promoter element around -30 of transcription start
CsDof14	TATA-box	1996	2001	core promoter element around -30 of transcription start

Name	Cis-element	Start position	Stop position	Function
CsDof14	TATA-box	1997	2001	core promoter element around -30 of transcription start
CsDof14	HD-Zip 1	291	299	element involved in differentiation of the palisade mesophyll cells
CsDof14	GARE-motif	501	508	gibberellin-responsive element
CsDof14	GARE-motif	516	523	gibberellin-responsive element
CsDof14	3-AF1 binding site	1507	1517	light responsive element
CsDof14	GT1-motif	450	456	light responsive element
CsDof14	GT1-motif	746	752	light responsive element
CsDof14	MBS	682	688	MYB binding site involved in drought-inducibility
CsDof14	MBS	733	739	MYB binding site involved in drought-inducibility
CsDof14	MBS	1122	1128	MYB binding site involved in drought-inducibility
CsDof15	ACE	161	170	cis-acting element involved in light responsiveness
CsDof15	LTR	1021	1027	cis-acting element involved in low-temperature responsiveness
CsDof15	TCA-element	1875	1884	cis-acting element involved in salicylic acid responsiveness
CsDof15	ARE	523	529	cis-acting regulatory element essential for the anaerobic induction
CsDof15	ARE	1364	1370	cis-acting regulatory element essential for the anaerobic induction
CsDof15	ARE	1516	1522	cis-acting regulatory element essential for the anaerobic induction
CsDof15	CGTCA-motif	380	385	cis-acting regulatory element involved in the MeJA-responsiveness
CsDof15	CGTCA-motif	514	519	cis-acting regulatory element involved in the MeJA-responsiveness
CsDof15	CGTCA-motif	590	595	cis-acting regulatory element involved in the MeJA-responsiveness
CsDof15	TGACG-motif	380	385	cis-acting regulatory element involved in the MeJA-responsiveness
CsDof15	TGACG-motif	514	519	cis-acting regulatory element involved in the MeJA-responsiveness
CsDof15	TGACG-motif	590	595	cis-acting regulatory element involved in the MeJA-responsiveness
CsDof15	CAAT-box	176	181	common cis-acting element in promoter and enhancer regions
CsDof15	CAAT-box	218	223	common cis-acting element in promoter and enhancer regions
CsDof15	CAAT-box	434	439	common cis-acting element in promoter and enhancer regions
CsDof15	CAAT-box	455	460	common cis-acting element in promoter and enhancer regions
CsDof15	CAAT-box	812	817	common cis-acting element in promoter and enhancer regions
CsDof15	CAAT-box	820	825	common cis-acting element in promoter and enhancer regions
CsDof15	CAAT-box	855	860	common cis-acting element in promoter and enhancer regions
CsDof15	CAAT-box	903	908	common cis-acting element in promoter and enhancer regions
CsDof15	CAAT-box	980	985	common cis-acting element in promoter and enhancer regions
CsDof15	CAAT-box	1155	1160	common cis-acting element in promoter and enhancer regions
CsDof15	CAAT-box	1608	1613	common cis-acting element in promoter and enhancer regions
CsDof15	CAAT-box	1806	1811	common cis-acting element in promoter and enhancer regions
CsDof15	CAAT-box	1862	1867	common cis-acting element in promoter and enhancer regions
CsDof15	TATA-box	29	33	core promoter element around -30 of transcription start
CsDof15	TATA-box	44	50	core promoter element around -30 of transcription start
CsDof15	TATA-box	45	50	core promoter element around -30 of transcription start
CsDof15	TATA-box	46	50	core promoter element around -30 of transcription start
CsDof15	TATA-box	148	152	core promoter element around -30 of transcription start
CsDof15	TATA-box	349	355	core promoter element around -30 of transcription start
CsDof15	TATA-box	350	355	core promoter element around -30 of transcription start
CsDof15	TATA-box	351	355	core promoter element around -30 of transcription start
CsDof15	TATA-box	578	585	core promoter element around -30 of transcription start
CsDof15	TATA-box	655	659	core promoter element around -30 of transcription start
CsDof15	TATA-box	860	867	core promoter element around -30 of transcription start
CsDof15	TATA-box	1067	1075	core promoter element around -30 of transcription start
CsDof15	TATA-box	1143	1147	core promoter element around -30 of transcription start
CsDof15	TATA-box	1191	1195	core promoter element around -30 of transcription start
CsDof15	TATA-box	1240	1245	core promoter element around -30 of transcription start
CsDof15	TATA-box	1241	1245	core promoter element around -30 of transcription start

Name	Cis-element	Start position	Stop position	Function
CsDof15	TATA-box	1366	1375	core promoter element around -30 of transcription start
CsDof15	TATA-box	1367	1374	core promoter element around -30 of transcription start
CsDof15	TATA-box	1368	1374	core promoter element around -30 of transcription start
CsDof15	TATA-box	1369	1376	core promoter element around -30 of transcription start
CsDof15	TATA-box	1370	1376	core promoter element around -30 of transcription start
CsDof15	TATA-box	1371	1377	core promoter element around -30 of transcription start
CsDof15	TATA-box	1372	1376	core promoter element around -30 of transcription start
CsDof15	TATA-box	1418	1426	core promoter element around -30 of transcription start
CsDof15	TATA-box	1425	1431	core promoter element around -30 of transcription start
CsDof15	TATA-box	1426	1431	core promoter element around -30 of transcription start
CsDof15	TATA-box	1427	1431	core promoter element around -30 of transcription start
CsDof15	TATA-box	1461	1468	core promoter element around -30 of transcription start
CsDof15	TATA-box	1462	1468	core promoter element around -30 of transcription start
CsDof15	TATA-box	1463	1469	core promoter element around -30 of transcription start
CsDof15	TATA-box	1464	1470	core promoter element around -30 of transcription start
CsDof15	TATA-box	1465	1471	core promoter element around -30 of transcription start
CsDof15	TATA-box	1466	1470	core promoter element around -30 of transcription start
CsDof15	TATA-box	1561	1568	core promoter element around -30 of transcription start
CsDof15	TATA-box	1643	1647	core promoter element around -30 of transcription start
CsDof15	TATA-box	1686	1690	core promoter element around -30 of transcription start
CsDof15	TATA-box	1709	1715	core promoter element around -30 of transcription start
CsDof15	TATA-box	1710	1714	core promoter element around -30 of transcription start
CsDof15	TATA-box	1840	1844	core promoter element around -30 of transcription start
CsDof15	TATA-box	1908	1917	core promoter element around -30 of transcription start
CsDof15	P-box	966	973	gibberellin-responsive element
CsDof15	GT1-motif	876	882	light responsive element
CsDof15	3-AF1 binding site	1899	1909	light responsive element
CsDof15	MBSI	1491	1501.5	MYB binding site involved in flavonoid biosynthetic genes regulation
CsDof15	MRE	1038	1045	MYB binding site involved in light responsiveness
CsDof15	MRE	1170	1177	MYB binding site involved in light responsiveness
CsDof15	WUN-motif	1339	1348	wound-responsive element
CsDof16	ABRE	502	509	abscisic acid responsiveness
CsDof16	ABRE	1017	1022	abscisic acid responsiveness
CsDof16	ABRE	1757	1762	abscisic acid responsiveness
CsDof16	LTR	1577	1583	cis-acting element involved in low-temperature responsiveness
CsDof16	ARE	1520	1526	cis-acting regulatory element essential for the anaerobic induction
CsDof16	circadian	553	562	cis-acting regulatory element involved in circadian control
CsDof16	G-box	778	784	cis-acting regulatory element involved in light responsiveness
CsDof16	G-Box	1016	1022	cis-acting regulatory element involved in light responsiveness
CsDof16	G-Box	1757	1763	cis-acting regulatory element involved in light responsiveness
CsDof16	TGACG-motif	547	552	cis-acting regulatory element involved in the MeJA-responsiveness
CsDof16	CGTCA-motif	547	552	cis-acting regulatory element involved in the MeJA-responsiveness
CsDof16	O2-site	871	881	cis-acting regulatory element involved in zein metabolism regulation
CsDof16	CAAT-box	86	91	common cis-acting element in promoter and enhancer regions
CsDof16	CAAT-box	482	487	common cis-acting element in promoter and enhancer regions
CsDof16	CAAT-box	541	546	common cis-acting element in promoter and enhancer regions
CsDof16	CAAT-box	616	621	common cis-acting element in promoter and enhancer regions
CsDof16	CAAT-box	642	647	common cis-acting element in promoter and enhancer regions
CsDof16	CAAT-box	796	801	common cis-acting element in promoter and enhancer regions
CsDof16	CAAT-box	906	911	common cis-acting element in promoter and enhancer regions
CsDof16	CAAT-box	1324	1329	common cis-acting element in promoter and enhancer regions

Name	Cis-element	Start position	Stop position	Function
CsDof16	CAAT-box	1550	1555	common cis-acting element in promoter and enhancer regions
CsDof16	CAAT-box	1642	1647	common cis-acting element in promoter and enhancer regions
CsDof16	CAAT-box	1840	1845	common cis-acting element in promoter and enhancer regions
CsDof16	TATA-box	2	9	core promoter element around -30 of transcription start
CsDof16	TATA-box	160	164	core promoter element around -30 of transcription start
CsDof16	TATA-box	349	353	core promoter element around -30 of transcription start
CsDof16	TATA-box	558	564	core promoter element around -30 of transcription start
CsDof16	TATA-box	559	563	core promoter element around -30 of transcription start
CsDof16	TATA-box	565	571	core promoter element around -30 of transcription start
CsDof16	TATA-box	567	573	core promoter element around -30 of transcription start
CsDof16	TATA-box	568	574	core promoter element around -30 of transcription start
CsDof16	TATA-box	569	575	core promoter element around -30 of transcription start
CsDof16	TATA-box	570	576	core promoter element around -30 of transcription start
CsDof16	TATA-box	571	577	core promoter element around -30 of transcription start
CsDof16	TATA-box	572	578	core promoter element around -30 of transcription start
CsDof16	TATA-box	573	579	core promoter element around -30 of transcription start
CsDof16	TATA-box	574	580	core promoter element around -30 of transcription start
CsDof16	TATA-box	575	581	core promoter element around -30 of transcription start
CsDof16	TATA-box	576	582	core promoter element around -30 of transcription start
CsDof16	TATA-box	577	583	core promoter element around -30 of transcription start
CsDof16	TATA-box	578	584	core promoter element around -30 of transcription start
CsDof16	TATA-box	579	583	core promoter element around -30 of transcription start
CsDof16	TATA-box	588	594	core promoter element around -30 of transcription start
CsDof16	TATA-box	589	593	core promoter element around -30 of transcription start
CsDof16	TATA-box	668	674	core promoter element around -30 of transcription start
CsDof16	TATA-box	669	675	core promoter element around -30 of transcription start
CsDof16	TATA-box	670	674	core promoter element around -30 of transcription start
CsDof16	TATA-box	693	700	core promoter element around -30 of transcription start
CsDof16	TATA-box	897	903	core promoter element around -30 of transcription start
CsDof16	TATA-box	898	904	core promoter element around -30 of transcription start
CsDof16	TATA-box	899	905	core promoter element around -30 of transcription start
CsDof16	TATA-box	900	904	core promoter element around -30 of transcription start
CsDof16	TATA-box	928	932	core promoter element around -30 of transcription start
CsDof16	TATA-box	941	948	core promoter element around -30 of transcription start
CsDof16	TATA-box	966	971	core promoter element around -30 of transcription start
CsDof16	TATA-box	967	971	core promoter element around -30 of transcription start
CsDof16	TATA-box	1001	1007	core promoter element around -30 of transcription start
CsDof16	TATA-box	1002	1007	core promoter element around -30 of transcription start
CsDof16	TATA-box	1003	1007	core promoter element around -30 of transcription start
CsDof16	TATA-box	1069	1075	core promoter element around -30 of transcription start
CsDof16	TATA-box	1070	1077	core promoter element around -30 of transcription start
CsDof16	TATA-box	1071	1077	core promoter element around -30 of transcription start
CsDof16	TATA-box	1073	1077	core promoter element around -30 of transcription start
CsDof16	TATA-box	1078	1084	core promoter element around -30 of transcription start
CsDof16	TATA-box	1079	1085	core promoter element around -30 of transcription start
CsDof16	TATA-box	1080	1086	core promoter element around -30 of transcription start
CsDof16	TATA-box	1081	1087	core promoter element around -30 of transcription start
CsDof16	TATA-box	1083	1087	core promoter element around -30 of transcription start
CsDof16	TATA-box	1221	1230	core promoter element around -30 of transcription start
CsDof16	TATA-box	1387	1395	core promoter element around -30 of transcription start
CsDof16	TATA-box	1388	1395	core promoter element around -30 of transcription start

Name	Cis-element	Start position	Stop position	Function
CsDof16	TATA-box	1389	1395	core promoter element around -30 of transcription start
CsDof16	TATA-box	1390	1395	core promoter element around -30 of transcription start
CsDof16	TATA-box	1391	1395	core promoter element around -30 of transcription start
CsDof16	TATA-box	1479	1488	core promoter element around -30 of transcription start
CsDof16	TATA-box	1480	1487	core promoter element around -30 of transcription start
CsDof16	TATA-box	1481	1487	core promoter element around -30 of transcription start
CsDof16	TATA-box	1482	1487	core promoter element around -30 of transcription start
CsDof16	TATA-box	1483	1487	core promoter element around -30 of transcription start
CsDof16	TATA-box	1499	1504	core promoter element around -30 of transcription start
CsDof16	TATA-box	1500	1504	core promoter element around -30 of transcription start
CsDof16	TATA-box	1512	1518	core promoter element around -30 of transcription start
CsDof16	TATA-box	1513	1517	core promoter element around -30 of transcription start
CsDof16	TATA-box	1628	1632	core promoter element around -30 of transcription start
CsDof16	TATA-box	1632	1641	core promoter element around -30 of transcription start
CsDof16	TATA-box	1633	1639	core promoter element around -30 of transcription start
CsDof16	TATA-box	1634	1641	core promoter element around -30 of transcription start
CsDof16	TATA-box	1635	1641	core promoter element around -30 of transcription start
CsDof16	TATA-box	1636	1642	core promoter element around -30 of transcription start
CsDof16	TATA-box	1637	1641	core promoter element around -30 of transcription start
CsDof16	TATA-box	1701	1709	core promoter element around -30 of transcription start
CsDof16	TATA-box	1815	1821	core promoter element around -30 of transcription start
CsDof16	TATA-box	1816	1820	core promoter element around -30 of transcription start
CsDof16	TATA-box	1872	1878	core promoter element around -30 of transcription start
CsDof16	TATA-box	1873	1879	core promoter element around -30 of transcription start
CsDof16	TATA-box	1874	1880	core promoter element around -30 of transcription start
CsDof16	TATA-box	1876	1880	core promoter element around -30 of transcription start
CsDof16	CCAAT-box	1540	1546	MYBHv1 binding site
CsDof17	ABRE	159	165	abscisic acid responsiveness
CsDof17	ABRE	160	165	abscisic acid responsiveness
CsDof17	ABRE	267	272	abscisic acid responsiveness
CsDof17	ABRE	395	400	abscisic acid responsiveness
CsDof17	ABRE	1826	1831	abscisic acid responsiveness
CsDof17	TGA-element	1494	1500	auxin-responsive element
CsDof17	TC-rich repeats	383	392	cis-acting element involved in defense and stress responsiveness
CsDof17	TCA-element	219	228	cis-acting element involved in salicylic acid responsiveness
CsDof17	TCA-element	1930	1939	cis-acting element involved in salicylic acid responsiveness
CsDof17	ARE	1094	1100	cis-acting regulatory element essential for the anaerobic induction
CsDof17	ARE	1509	1515	cis-acting regulatory element essential for the anaerobic induction
CsDof17	G-box	159	165	cis-acting regulatory element involved in light responsiveness
CsDof17	G-box	395	401	cis-acting regulatory element involved in light responsiveness
CsDof17	G-box	1825	1831	cis-acting regulatory element involved in light responsiveness
CsDof17	G-Box	159	165	cis-acting regulatory element involved in light responsiveness
CsDof17	G-Box	266	272	cis-acting regulatory element involved in light responsiveness
CsDof17	G-Box	1318	1328	cis-acting regulatory element involved in light responsiveness
CsDof17	CGTCA-motif	397	402	cis-acting regulatory element involved in the MeJA-responsiveness
CsDof17	CGTCA-motif	1824	1829	cis-acting regulatory element involved in the MeJA-responsiveness
CsDof17	TGACG-motif	397	402	cis-acting regulatory element involved in the MeJA-responsiveness
CsDof17	TGACG-motif	1824	1829	cis-acting regulatory element involved in the MeJA-responsiveness
CsDof17	O2-site	1151	1160	cis-acting regulatory element involved in zein metabolism regulation
CsDof17	CAAT-box	42	47	common cis-acting element in promoter and enhancer regions
CsDof17	CAAT-box	308	313	common cis-acting element in promoter and enhancer regions

Name	Cis-element	Start position	Stop position	Function
CsDof17	CAAT-box	329	334	common cis-acting element in promoter and enhancer regions
CsDof17	CAAT-box	380	385	common cis-acting element in promoter and enhancer regions
CsDof17	CAAT-box	449	454	common cis-acting element in promoter and enhancer regions
CsDof17	CAAT-box	472	477	common cis-acting element in promoter and enhancer regions
CsDof17	CAAT-box	659	664	common cis-acting element in promoter and enhancer regions
CsDof17	CAAT-box	670	675	common cis-acting element in promoter and enhancer regions
CsDof17	CAAT-box	689	694	common cis-acting element in promoter and enhancer regions
CsDof17	CAAT-box	743	748	common cis-acting element in promoter and enhancer regions
CsDof17	CAAT-box	785	790	common cis-acting element in promoter and enhancer regions
CsDof17	CAAT-box	811	816	common cis-acting element in promoter and enhancer regions
CsDof17	CAAT-box	900	905	common cis-acting element in promoter and enhancer regions
CsDof17	CAAT-box	1059	1064	common cis-acting element in promoter and enhancer regions
CsDof17	CAAT-box	1092	1097	common cis-acting element in promoter and enhancer regions
CsDof17	CAAT-box	1119	1124	common cis-acting element in promoter and enhancer regions
CsDof17	CAAT-box	1144	1149	common cis-acting element in promoter and enhancer regions
CsDof17	CAAT-box	1204	1209	common cis-acting element in promoter and enhancer regions
CsDof17	CAAT-box	1213	1218	common cis-acting element in promoter and enhancer regions
CsDof17	CAAT-box	1248	1253	common cis-acting element in promoter and enhancer regions
CsDof17	CAAT-box	1433	1438	common cis-acting element in promoter and enhancer regions
CsDof17	CAAT-box	1601	1606	common cis-acting element in promoter and enhancer regions
CsDof17	CAAT-box	1812	1817	common cis-acting element in promoter and enhancer regions
CsDof17	TATA-box	2	10	core promoter element around -30 of transcription start
CsDof17	TATA-box	66	73	core promoter element around -30 of transcription start
CsDof17	TATA-box	67	73	core promoter element around -30 of transcription start
CsDof17	TATA-box	68	73	core promoter element around -30 of transcription start
CsDof17	TATA-box	69	73	core promoter element around -30 of transcription start
CsDof17	TATA-box	83	90	core promoter element around -30 of transcription start
CsDof17	TATA-box	84	90	core promoter element around -30 of transcription start
CsDof17	TATA-box	85	90	core promoter element around -30 of transcription start
CsDof17	TATA-box	86	90	core promoter element around -30 of transcription start
CsDof17	TATA-box	521	530	core promoter element around -30 of transcription start
CsDof17	TATA-box	527	531	core promoter element around -30 of transcription start
CsDof17	TATA-box	530	536	core promoter element around -30 of transcription start
CsDof17	TATA-box	531	536	core promoter element around -30 of transcription start
CsDof17	TATA-box	532	536	core promoter element around -30 of transcription start
CsDof17	TATA-box	541	545	core promoter element around -30 of transcription start
CsDof17	TATA-box	558	564	core promoter element around -30 of transcription start
CsDof17	TATA-box	559	565	core promoter element around -30 of transcription start
CsDof17	TATA-box	561	565	core promoter element around -30 of transcription start
CsDof17	TATA-box	682	688	core promoter element around -30 of transcription start
CsDof17	TATA-box	683	687	core promoter element around -30 of transcription start
CsDof17	TATA-box	700	709	core promoter element around -30 of transcription start
CsDof17	TATA-box	702	710	core promoter element around -30 of transcription start
CsDof17	TATA-box	708	712	core promoter element around -30 of transcription start
CsDof17	TATA-box	798	804	core promoter element around -30 of transcription start
CsDof17	TATA-box	799	803	core promoter element around -30 of transcription start
CsDof17	TATA-box	876	880	core promoter element around -30 of transcription start
CsDof17	TATA-box	984	993	core promoter element around -30 of transcription start
CsDof17	TATA-box	985	992	core promoter element around -30 of transcription start
CsDof17	TATA-box	986	992	core promoter element around -30 of transcription start
CsDof17	TATA-box	987	992	core promoter element around -30 of transcription start

Name	Cis-element	Start position	Stop position	Function
CsDof17	TATA-box	988	992	core promoter element around -30 of transcription start
CsDof17	TATA-box	1072	1080	core promoter element around -30 of transcription start
CsDof17	TATA-box	1075	1080	core promoter element around -30 of transcription start
CsDof17	TATA-box	1076	1080	core promoter element around -30 of transcription start
CsDof17	TATA-box	1131	1135	core promoter element around -30 of transcription start
CsDof17	TATA-box	1241	1246	core promoter element around -30 of transcription start
CsDof17	TATA-box	1242	1246	core promoter element around -30 of transcription start
CsDof17	TATA-box	1253	1260	core promoter element around -30 of transcription start
CsDof17	TATA-box	1356	1362	core promoter element around -30 of transcription start
CsDof17	TATA-box	1357	1361	core promoter element around -30 of transcription start
CsDof17	TATA-box	1410	1416	core promoter element around -30 of transcription start
CsDof17	TATA-box	1411	1415	core promoter element around -30 of transcription start
CsDof17	TATA-box	1553	1562	core promoter element around -30 of transcription start
CsDof17	TATA-box	1554	1561	core promoter element around -30 of transcription start
CsDof17	TATA-box	1555	1561	core promoter element around -30 of transcription start
CsDof17	TATA-box	1556	1563	core promoter element around -30 of transcription start
CsDof17	TATA-box	1557	1563	core promoter element around -30 of transcription start
CsDof17	TATA-box	1558	1564	core promoter element around -30 of transcription start
CsDof17	TATA-box	1559	1563	core promoter element around -30 of transcription start
CsDof17	TATA-box	1596	1602	core promoter element around -30 of transcription start
CsDof17	TATA-box	1598	1602	core promoter element around -30 of transcription start
CsDof17	TATA-box	1613	1620	core promoter element around -30 of transcription start
CsDof17	TATA-box	1614	1620	core promoter element around -30 of transcription start
CsDof17	TATA-box	1615	1620	core promoter element around -30 of transcription start
CsDof17	TATA-box	1616	1620	core promoter element around -30 of transcription start
CsDof17	TATA-box	1703	1709	core promoter element around -30 of transcription start
CsDof17	TATA-box	1704	1709	core promoter element around -30 of transcription start
CsDof17	TATA-box	1705	1709	core promoter element around -30 of transcription start
CsDof17	TATA-box	1718	1724	core promoter element around -30 of transcription start
CsDof17	TATA-box	1719	1723	core promoter element around -30 of transcription start
CsDof17	TATA-box	1845	1852	core promoter element around -30 of transcription start
CsDof17	GARE-motif	23	30	gibberellin-responsive element
CsDof17	GT1-motif	323	329	light responsive element
CsDof17	MBS	1081	1087	MYB binding site involved in drought-inducibility
CsDof17	MBS	1451	1457	MYB binding site involved in drought-inducibility
CsDof17	MRE	933	940	MYB binding site involved in light responsiveness
CsDof18	ABRE	827	832	abscisic acid responsiveness
CsDof18	ABRE	1134	1141	abscisic acid responsiveness
CsDof18	ABRE	1344	1349	abscisic acid responsiveness
CsDof18	TGA-element	519	525	auxin-responsive element
CsDof18	TGA-element	1555	1561	auxin-responsive element
CsDof18	ARE	1216	1222	cis-acting regulatory element essential for the anaerobic induction
CsDof18	ARE	1511	1517	cis-acting regulatory element essential for the anaerobic induction
CsDof18	G-Box	826	832	cis-acting regulatory element involved in light responsiveness
CsDof18	G-Box	1343	1349	cis-acting regulatory element involved in light responsiveness
CsDof18	CAT-box	476	482	cis-acting regulatory element related to meristem expression
CsDof18	CAAT-box	27	32	common cis-acting element in promoter and enhancer regions
CsDof18	CAAT-box	50	55	common cis-acting element in promoter and enhancer regions
CsDof18	CAAT-box	154	159	common cis-acting element in promoter and enhancer regions
CsDof18	CAAT-box	299	304	common cis-acting element in promoter and enhancer regions
CsDof18	CAAT-box	302	307	common cis-acting element in promoter and enhancer regions

Name	Cis-element	Start position	Stop position	Function
CsDof18	CAAT-box	353	358	common cis-acting element in promoter and enhancer regions
CsDof18	CAAT-box	422	427	common cis-acting element in promoter and enhancer regions
CsDof18	CAAT-box	552	557	common cis-acting element in promoter and enhancer regions
CsDof18	CAAT-box	555	560	common cis-acting element in promoter and enhancer regions
CsDof18	CAAT-box	660	665	common cis-acting element in promoter and enhancer regions
CsDof18	CAAT-box	721	726	common cis-acting element in promoter and enhancer regions
CsDof18	CAAT-box	754	759	common cis-acting element in promoter and enhancer regions
CsDof18	CAAT-box	776	781	common cis-acting element in promoter and enhancer regions
CsDof18	CAAT-box	792	797	common cis-acting element in promoter and enhancer regions
CsDof18	CAAT-box	943	948	common cis-acting element in promoter and enhancer regions
CsDof18	CAAT-box	1012	1017	common cis-acting element in promoter and enhancer regions
CsDof18	CAAT-box	1079	1084	common cis-acting element in promoter and enhancer regions
CsDof18	CAAT-box	1154	1159	common cis-acting element in promoter and enhancer regions
CsDof18	CAAT-box	1581	1586	common cis-acting element in promoter and enhancer regions
CsDof18	CAAT-box	1620	1625	common cis-acting element in promoter and enhancer regions
CsDof18	CAAT-box	1859	1869	common cis-acting element in promoter and enhancer regions
CsDof18	CAAT-box	1862	1867	common cis-acting element in promoter and enhancer regions
CsDof18	TATA-box	68	73	core promoter element around -30 of transcription start
CsDof18	TATA-box	69	73	core promoter element around -30 of transcription start
CsDof18	TATA-box	186	191	core promoter element around -30 of transcription start
CsDof18	TATA-box	187	191	core promoter element around -30 of transcription start
CsDof18	TATA-box	231	235	core promoter element around -30 of transcription start
CsDof18	TATA-box	240	246	core promoter element around -30 of transcription start
CsDof18	TATA-box	241	247	core promoter element around -30 of transcription start
CsDof18	TATA-box	242	248	core promoter element around -30 of transcription start
CsDof18	TATA-box	243	247	core promoter element around -30 of transcription start
CsDof18	TATA-box	312	318	core promoter element around -30 of transcription start
CsDof18	TATA-box	314	318	core promoter element around -30 of transcription start
CsDof18	TATA-box	387	394	core promoter element around -30 of transcription start
CsDof18	TATA-box	388	394	core promoter element around -30 of transcription start
CsDof18	TATA-box	390	394	core promoter element around -30 of transcription start
CsDof18	TATA-box	512	519	core promoter element around -30 of transcription start
CsDof18	TATA-box	513	519	core promoter element around -30 of transcription start
CsDof18	TATA-box	514	519	core promoter element around -30 of transcription start
CsDof18	TATA-box	515	519	core promoter element around -30 of transcription start
CsDof18	TATA-box	539	543	core promoter element around -30 of transcription start
CsDof18	TATA-box	583	594	core promoter element around -30 of transcription start
CsDof18	TATA-box	585	589	core promoter element around -30 of transcription start
CsDof18	TATA-box	631	635	core promoter element around -30 of transcription start
CsDof18	TATA-box	724	730	core promoter element around -30 of transcription start
CsDof18	TATA-box	725	730	core promoter element around -30 of transcription start
CsDof18	TATA-box	726	730	core promoter element around -30 of transcription start
CsDof18	TATA-box	803	810	core promoter element around -30 of transcription start
CsDof18	TATA-box	957	961	core promoter element around -30 of transcription start
CsDof18	TATA-box	965	969	core promoter element around -30 of transcription start
CsDof18	TATA-box	1044	1051	core promoter element around -30 of transcription start
CsDof18	TATA-box	1045	1051	core promoter element around -30 of transcription start
CsDof18	TATA-box	1046	1052	core promoter element around -30 of transcription start
CsDof18	TATA-box	1047	1051	core promoter element around -30 of transcription start
CsDof18	TATA-box	1051	1057	core promoter element around -30 of transcription start
CsDof18	TATA-box	1052	1059	core promoter element around -30 of transcription start

Name	Cis-element	Start position	Stop position	Function
CsDof18	TATA-box	1053	1059	core promoter element around -30 of transcription start
CsDof18	TATA-box	1054	1060	core promoter element around -30 of transcription start
CsDof18	TATA-box	1055	1059	core promoter element around -30 of transcription start
CsDof18	TATA-box	1064	1070	core promoter element around -30 of transcription start
CsDof18	TATA-box	1065	1069	core promoter element around -30 of transcription start
CsDof18	TATA-box	1220	1227	core promoter element around -30 of transcription start
CsDof18	TATA-box	1221	1227	core promoter element around -30 of transcription start
CsDof18	TATA-box	1222	1227	core promoter element around -30 of transcription start
CsDof18	TATA-box	1223	1227	core promoter element around -30 of transcription start
CsDof18	TATA-box	1242	1247	core promoter element around -30 of transcription start
CsDof18	TATA-box	1243	1247	core promoter element around -30 of transcription start
CsDof18	TATA-box	1330	1336	core promoter element around -30 of transcription start
CsDof18	TATA-box	1331	1337	core promoter element around -30 of transcription start
CsDof18	TATA-box	1332	1338	core promoter element around -30 of transcription start
CsDof18	TATA-box	1333	1337	core promoter element around -30 of transcription start
CsDof18	TATA-box	1421	1428	core promoter element around -30 of transcription start
CsDof18	TATA-box	1422	1428	core promoter element around -30 of transcription start
CsDof18	TATA-box	1423	1428	core promoter element around -30 of transcription start
CsDof18	TATA-box	1424	1428	core promoter element around -30 of transcription start
CsDof18	TATA-box	1452	1456	core promoter element around -30 of transcription start
CsDof18	TATA-box	1499	1505	core promoter element around -30 of transcription start
CsDof18	TATA-box	1500	1505	core promoter element around -30 of transcription start
CsDof18	TATA-box	1501	1505	core promoter element around -30 of transcription start
CsDof18	TATA-box	1607	1611	core promoter element around -30 of transcription start
CsDof18	TATA-box	1614	1618	core promoter element around -30 of transcription start
CsDof18	TATA-box	1645	1651	core promoter element around -30 of transcription start
CsDof18	TATA-box	1646	1650	core promoter element around -30 of transcription start
CsDof18	TATA-box	1868	1877	core promoter element around -30 of transcription start
CsDof18	P-box	607	614	gibberellin-responsive element
CsDof18	GT1-motif	1015	1021	light responsive element
CsDof18	GT1-motif	1166	1172	light responsive element
CsDof18	GT1-motif	1925	1932	light responsive element
CsDof18	GT1-motif	1926	1932	light responsive element
CsDof18	Sp1	467	473	light responsive element
CsDof18	MBSI	501	511.5	MYB binding site involved in flavonoid biosynthetic genes regulation
CsDof18	CCAAT-box	1517	1523	MYBHv1 binding site
CsDof19	ABRE	1897	1903	abscisic acid responsiveness
CsDof19	ABRE	1898	1903	abscisic acid responsiveness
CsDof19	TC-rich repeats	100	109	cis-acting element involved in defense and stress responsiveness
CsDof19	LTR	51	57	cis-acting element involved in low-temperature responsiveness
CsDof19	ARE	1090	1096	cis-acting regulatory element essential for the anaerobic induction
CsDof19	G-Box	1897	1903	cis-acting regulatory element involved in light responsiveness
CsDof19	G-box	1389	1395	cis-acting regulatory element involved in light responsiveness
CsDof19	G-box	1896	1905	cis-acting regulatory element involved in light responsiveness
CsDof19	G-box	1897	1903	cis-acting regulatory element involved in light responsiveness
CsDof19	RY-element	1943	1951	cis-acting regulatory element involved in seed-specific regulation
CsDof19	TGACG-motif	898	903	cis-acting regulatory element involved in the MeJA-responsiveness
CsDof19	CGTCA-motif	898	903	cis-acting regulatory element involved in the MeJA-responsiveness
CsDof19	O2-site	677	685	cis-acting regulatory element involved in zein metabolism regulation
CsDof19	CAT-box	466	472	cis-acting regulatory element related to meristem expression
CsDof19	CAAT-box	161	166	common cis-acting element in promoter and enhancer regions

Name	Cis-element	Start position	Stop position	Function
CsDof19	CAAT-box	742	747	common cis-acting element in promoter and enhancer regions
CsDof19	CAAT-box	1229	1234	common cis-acting element in promoter and enhancer regions
CsDof19	CAAT-box	1283	1288	common cis-acting element in promoter and enhancer regions
CsDof19	CAAT-box	1467	1472	common cis-acting element in promoter and enhancer regions
CsDof19	CAAT-box	1640	1645	common cis-acting element in promoter and enhancer regions
CsDof19	CAAT-box	1659	1664	common cis-acting element in promoter and enhancer regions
CsDof19	CAAT-box	1861	1866	common cis-acting element in promoter and enhancer regions
CsDof19	TATA-box	45	51	core promoter element around -30 of transcription start
CsDof19	TATA-box	46	50	core promoter element around -30 of transcription start
CsDof19	TATA-box	140	146	core promoter element around -30 of transcription start
CsDof19	TATA-box	141	146	core promoter element around -30 of transcription start
CsDof19	TATA-box	142	146	core promoter element around -30 of transcription start
CsDof19	TATA-box	488	495	core promoter element around -30 of transcription start
CsDof19	TATA-box	950	954	core promoter element around -30 of transcription start
CsDof19	TATA-box	1015	1020	core promoter element around -30 of transcription start
CsDof19	TATA-box	1016	1020	core promoter element around -30 of transcription start
CsDof19	TATA-box	1099	1105	core promoter element around -30 of transcription start
CsDof19	TATA-box	1101	1105	core promoter element around -30 of transcription start
CsDof19	TATA-box	1129	1138	core promoter element around -30 of transcription start
CsDof19	TATA-box	1130	1137	core promoter element around -30 of transcription start
CsDof19	TATA-box	1131	1137	core promoter element around -30 of transcription start
CsDof19	TATA-box	1132	1137	core promoter element around -30 of transcription start
CsDof19	TATA-box	1133	1137	core promoter element around -30 of transcription start
CsDof19	TATA-box	1279	1283	core promoter element around -30 of transcription start
CsDof19	TATA-box	1396	1402	core promoter element around -30 of transcription start
CsDof19	TATA-box	1397	1401	core promoter element around -30 of transcription start
CsDof19	TATA-box	1479	1485	core promoter element around -30 of transcription start
CsDof19	TATA-box	1480	1486	core promoter element around -30 of transcription start
CsDof19	TATA-box	1482	1486	core promoter element around -30 of transcription start
CsDof19	TATA-box	1496	1501	core promoter element around -30 of transcription start
CsDof19	TATA-box	1497	1501	core promoter element around -30 of transcription start
CsDof19	TATA-box	1521	1527	core promoter element around -30 of transcription start
CsDof19	TATA-box	1522	1526	core promoter element around -30 of transcription start
CsDof19	TATA-box	1570	1575	core promoter element around -30 of transcription start
CsDof19	TATA-box	1571	1575	core promoter element around -30 of transcription start
CsDof19	TATA-box	1574	1580	core promoter element around -30 of transcription start
CsDof19	TATA-box	1575	1580	core promoter element around -30 of transcription start
CsDof19	TATA-box	1576	1580	core promoter element around -30 of transcription start
CsDof19	TATA-box	1601	1607	core promoter element around -30 of transcription start
CsDof19	TATA-box	1602	1608	core promoter element around -30 of transcription start
CsDof19	TATA-box	1604	1608	core promoter element around -30 of transcription start
CsDof19	TATA-box	1773	1777	core promoter element around -30 of transcription start
CsDof19	TATA-box	1789	1795	core promoter element around -30 of transcription start
CsDof19	TATA-box	1790	1796	core promoter element around -30 of transcription start
CsDof19	TATA-box	1791	1795	core promoter element around -30 of transcription start
CsDof19	TATA-box	1953	1959	core promoter element around -30 of transcription start
CsDof19	TATA-box	1954	1960	core promoter element around -30 of transcription start
CsDof19	TATA-box	1955	1959	core promoter element around -30 of transcription start
CsDof19	P-box	401	408	gibberellin-responsive element
CsDof19	GT1-motif	1733	1740	light responsive element
CsDof19	GT1-motif	1734	1740	light responsive element

Name	Cis-element	Start position	Stop position	Function
CsDof19	GT1-motif	1812	1818	light responsive element
CsDof19	CCAAT-box	84	90	MYBHv1 binding site
CsDof20	TGA-element	1353	1359	auxin-responsive element
CsDof20	TC-rich repeats	1332	1341	cis-acting element involved in defense and stress responsiveness
CsDof20	ARE	801	807	cis-acting regulatory element essential for the anaerobic induction
CsDof20	AuxRR-core	333	340	cis-acting regulatory element involved in auxin responsiveness
CsDof20	CGTCA-motif	1718	1723	cis-acting regulatory element involved in the MeJA-responsiveness
CsDof20	TGACG-motif	1718	1723	cis-acting regulatory element involved in the MeJA-responsiveness
CsDof20	CAAT-box	64	69	common cis-acting element in promoter and enhancer regions
CsDof20	CAAT-box	81	86	common cis-acting element in promoter and enhancer regions
CsDof20	CAAT-box	88	93	common cis-acting element in promoter and enhancer regions
CsDof20	CAAT-box	206	211	common cis-acting element in promoter and enhancer regions
CsDof20	CAAT-box	354	359	common cis-acting element in promoter and enhancer regions
CsDof20	CAAT-box	365	370	common cis-acting element in promoter and enhancer regions
CsDof20	CAAT-box	375	380	common cis-acting element in promoter and enhancer regions
CsDof20	CAAT-box	636	641	common cis-acting element in promoter and enhancer regions
CsDof20	CAAT-box	641	646	common cis-acting element in promoter and enhancer regions
CsDof20	CAAT-box	690	695	common cis-acting element in promoter and enhancer regions
CsDof20	CAAT-box	699	704	common cis-acting element in promoter and enhancer regions
CsDof20	CAAT-box	759	764	common cis-acting element in promoter and enhancer regions
CsDof20	CAAT-box	1053	1058	common cis-acting element in promoter and enhancer regions
CsDof20	CAAT-box	1066	1071	common cis-acting element in promoter and enhancer regions
CsDof20	CAAT-box	1087	1092	common cis-acting element in promoter and enhancer regions
CsDof20	CAAT-box	1632	1637	common cis-acting element in promoter and enhancer regions
CsDof20	TATA-box	153	160	core promoter element around -30 of transcription start
CsDof20	TATA-box	154	160	core promoter element around -30 of transcription start
CsDof20	TATA-box	155	160	core promoter element around -30 of transcription start
CsDof20	TATA-box	156	160	core promoter element around -30 of transcription start
CsDof20	TATA-box	214	219	core promoter element around -30 of transcription start
CsDof20	TATA-box	215	219	core promoter element around -30 of transcription start
CsDof20	TATA-box	262	268	core promoter element around -30 of transcription start
CsDof20	TATA-box	263	269	core promoter element around -30 of transcription start
CsDof20	TATA-box	264	270	core promoter element around -30 of transcription start
CsDof20	TATA-box	265	269	core promoter element around -30 of transcription start
CsDof20	TATA-box	269	278	core promoter element around -30 of transcription start
CsDof20	TATA-box	500	504	core promoter element around -30 of transcription start
CsDof20	TATA-box	506	510	core promoter element around -30 of transcription start
CsDof20	TATA-box	535	541	core promoter element around -30 of transcription start
CsDof20	TATA-box	536	540	core promoter element around -30 of transcription start
CsDof20	TATA-box	571	576	core promoter element around -30 of transcription start
CsDof20	TATA-box	572	576	core promoter element around -30 of transcription start
CsDof20	TATA-box	654	660	core promoter element around -30 of transcription start
CsDof20	TATA-box	655	660	core promoter element around -30 of transcription start
CsDof20	TATA-box	656	660	core promoter element around -30 of transcription start
CsDof20	TATA-box	710	716	core promoter element around -30 of transcription start
CsDof20	TATA-box	711	716	core promoter element around -30 of transcription start
CsDof20	TATA-box	712	716	core promoter element around -30 of transcription start
CsDof20	TATA-box	880	884	core promoter element around -30 of transcription start
CsDof20	TATA-box	910	919	core promoter element around -30 of transcription start
CsDof20	TATA-box	911	918	core promoter element around -30 of transcription start
CsDof20	TATA-box	912	918	core promoter element around -30 of transcription start

Name	Cis-element	Start position	Stop position	Function
CsDof20	TATA-box	913	918	core promoter element around -30 of transcription start
CsDof20	TATA-box	914	918	core promoter element around -30 of transcription start
CsDof20	TATA-box	941	948	core promoter element around -30 of transcription start
CsDof20	TATA-box	942	948	core promoter element around -30 of transcription start
CsDof20	TATA-box	943	948	core promoter element around -30 of transcription start
CsDof20	TATA-box	944	948	core promoter element around -30 of transcription start
CsDof20	TATA-box	1075	1081	core promoter element around -30 of transcription start
CsDof20	TATA-box	1076	1081	core promoter element around -30 of transcription start
CsDof20	TATA-box	1077	1081	core promoter element around -30 of transcription start
CsDof20	TATA-box	1106	1113	core promoter element around -30 of transcription start
CsDof20	TATA-box	1107	1113	core promoter element around -30 of transcription start
CsDof20	TATA-box	1108	1115	core promoter element around -30 of transcription start
CsDof20	TATA-box	1109	1115	core promoter element around -30 of transcription start
CsDof20	TATA-box	1110	1116	core promoter element around -30 of transcription start
CsDof20	TATA-box	1111	1115	core promoter element around -30 of transcription start
CsDof20	TATA-box	1342	1346	core promoter element around -30 of transcription start
CsDof20	TATA-box	1373	1379	core promoter element around -30 of transcription start
CsDof20	TATA-box	1374	1379	core promoter element around -30 of transcription start
CsDof20	TATA-box	1375	1387	core promoter element around -30 of transcription start
CsDof20	TATA-box	1377	1385	core promoter element around -30 of transcription start
CsDof20	TATA-box	1408	1414	core promoter element around -30 of transcription start
CsDof20	TATA-box	1409	1413	core promoter element around -30 of transcription start
CsDof20	TATA-box	1543	1549	core promoter element around -30 of transcription start
CsDof20	TATA-box	1545	1551	core promoter element around -30 of transcription start
CsDof20	TATA-box	1546	1552	core promoter element around -30 of transcription start
CsDof20	TATA-box	1547	1553	core promoter element around -30 of transcription start
CsDof20	TATA-box	1548	1554	core promoter element around -30 of transcription start
CsDof20	TATA-box	1549	1553	core promoter element around -30 of transcription start
CsDof20	TATA-box	1655	1659	core promoter element around -30 of transcription start
CsDof20	TATA-box	1661	1667	core promoter element around -30 of transcription start
CsDof20	TATA-box	1662	1666	core promoter element around -30 of transcription start
CsDof20	TATA-box	1685	1691	core promoter element around -30 of transcription start
CsDof20	TATA-box	1686	1690	core promoter element around -30 of transcription start
CsDof20	GT1-motif	1788	1794	light responsive element
CsDof20	Box III	1604	1615	protein binding site
CsDof21	ABRE	246	251	abscisic acid responsiveness
CsDof21	ABRE	265	270	abscisic acid responsiveness
CsDof21	TGA-element	1010	1016	auxin-responsive element
CsDof21	TGA-element	1356	1362	auxin-responsive element
CsDof21	AT-rich element	1209	1219	binding site of AT-rich DNA binding protein (ATBP-1)
CsDof21	TC-rich repeats	1622	1631	cis-acting element involved in defense and stress responsiveness
CsDof21	TATC-box	478	485	cis-acting element involved in gibberellin-responsiveness
CsDof21	AuxRR-core	978	985	cis-acting regulatory element involved in auxin responsiveness
CsDof21	G-box	187	196	cis-acting regulatory element involved in light responsiveness
CsDof21	G-box	245	251	cis-acting regulatory element involved in light responsiveness
CsDof21	G-box	264	270	cis-acting regulatory element involved in light responsiveness
CsDof21	TGACG-motif	236	241	cis-acting regulatory element involved in the MeJA-responsiveness
CsDof21	TGACG-motif	244	249	cis-acting regulatory element involved in the MeJA-responsiveness
CsDof21	TGACG-motif	897	902	cis-acting regulatory element involved in the MeJA-responsiveness
CsDof21	CGTCA-motif	236	241	cis-acting regulatory element involved in the MeJA-responsiveness
CsDof21	CGTCA-motif	244	249	cis-acting regulatory element involved in the MeJA-responsiveness

Name	Cis-element	Start position	Stop position	Function
CsDof21	CGTCA-motif	897	902	cis-acting regulatory element involved in the MeJA-responsiveness
CsDof21	CAAT-box	64	71	common cis-acting element in promoter and enhancer regions
CsDof21	CAAT-box	334	339	common cis-acting element in promoter and enhancer regions
CsDof21	CAAT-box	655	660	common cis-acting element in promoter and enhancer regions
CsDof21	CAAT-box	672	677	common cis-acting element in promoter and enhancer regions
CsDof21	CAAT-box	783	788	common cis-acting element in promoter and enhancer regions
CsDof21	CAAT-box	1077	1082	common cis-acting element in promoter and enhancer regions
CsDof21	CAAT-box	1476	1481	common cis-acting element in promoter and enhancer regions
CsDof21	CAAT-box	1486	1491	common cis-acting element in promoter and enhancer regions
CsDof21	TATA-box	310	314	core promoter element around -30 of transcription start
CsDof21	TATA-box	331	335	core promoter element around -30 of transcription start
CsDof21	TATA-box	434	440	core promoter element around -30 of transcription start
CsDof21	TATA-box	435	440	core promoter element around -30 of transcription start
CsDof21	TATA-box	436	440	core promoter element around -30 of transcription start
CsDof21	TATA-box	495	502	core promoter element around -30 of transcription start
CsDof21	TATA-box	547	551	core promoter element around -30 of transcription start
CsDof21	TATA-box	633	640	core promoter element around -30 of transcription start
CsDof21	TATA-box	634	640	core promoter element around -30 of transcription start
CsDof21	TATA-box	635	640	core promoter element around -30 of transcription start
CsDof21	TATA-box	636	640	core promoter element around -30 of transcription start
CsDof21	TATA-box	702	706	core promoter element around -30 of transcription start
CsDof21	TATA-box	746	752	core promoter element around -30 of transcription start
CsDof21	TATA-box	747	751	core promoter element around -30 of transcription start
CsDof21	TATA-box	769	773	core promoter element around -30 of transcription start
CsDof21	TATA-box	830	837	core promoter element around -30 of transcription start
CsDof21	TATA-box	1096	1102	core promoter element around -30 of transcription start
CsDof21	TATA-box	1097	1102	core promoter element around -30 of transcription start
CsDof21	TATA-box	1098	1102	core promoter element around -30 of transcription start
CsDof21	TATA-box	1116	1122	core promoter element around -30 of transcription start
CsDof21	TATA-box	1117	1122	core promoter element around -30 of transcription start
CsDof21	TATA-box	1118	1122	core promoter element around -30 of transcription start
CsDof21	TATA-box	1184	1190	core promoter element around -30 of transcription start
CsDof21	TATA-box	1185	1189	core promoter element around -30 of transcription start
CsDof21	TATA-box	1221	1227	core promoter element around -30 of transcription start
CsDof21	TATA-box	1222	1228	core promoter element around -30 of transcription start
CsDof21	TATA-box	1223	1229	core promoter element around -30 of transcription start
CsDof21	TATA-box	1224	1230	core promoter element around -30 of transcription start
CsDof21	TATA-box	1225	1229	core promoter element around -30 of transcription start
CsDof21	TATA-box	1274	1280	core promoter element around -30 of transcription start
CsDof21	TATA-box	1275	1279	core promoter element around -30 of transcription start
CsDof21	TATA-box	1461	1466	core promoter element around -30 of transcription start
CsDof21	TATA-box	1462	1466	core promoter element around -30 of transcription start
CsDof21	TATA-box	1567	1571	core promoter element around -30 of transcription start
CsDof21	TATA-box	1572	1579	core promoter element around -30 of transcription start
CsDof21	TATA-box	1573	1579	core promoter element around -30 of transcription start
CsDof21	TATA-box	1574	1580	core promoter element around -30 of transcription start
CsDof21	TATA-box	1575	1581	core promoter element around -30 of transcription start
CsDof21	TATA-box	1576	1582	core promoter element around -30 of transcription start
CsDof21	TATA-box	1577	1581	core promoter element around -30 of transcription start
CsDof21	TATA-box	1685	1691	core promoter element around -30 of transcription start
CsDof21	TATA-box	1686	1690	core promoter element around -30 of transcription start

Name	Cis-element	Start position	Stop position	Function
CsDof21	TATA-box	1715	1722	core promoter element around -30 of transcription start
CsDof21	TATA-box	1726	1732	core promoter element around -30 of transcription start
CsDof21	TATA-box	1728	1732	core promoter element around -30 of transcription start
CsDof21	TATA-box	1741	1747	core promoter element around -30 of transcription start
CsDof21	TATA-box	1742	1748	core promoter element around -30 of transcription start
CsDof21	TATA-box	1743	1749	core promoter element around -30 of transcription start
CsDof21	TATA-box	1744	1748	core promoter element around -30 of transcription start
CsDof21	TATA-box	1756	1765	core promoter element around -30 of transcription start
CsDof21	TATA-box	1757	1763	core promoter element around -30 of transcription start
CsDof21	TATA-box	1758	1764	core promoter element around -30 of transcription start
CsDof21	TATA-box	1759	1765	core promoter element around -30 of transcription start
CsDof21	TATA-box	1760	1766	core promoter element around -30 of transcription start
CsDof21	TATA-box	1761	1767	core promoter element around -30 of transcription start
CsDof21	TATA-box	1762	1766	core promoter element around -30 of transcription start
CsDof21	GT1-motif	1029	1035	light responsive element
CsDof21	MBS	71	77	MYB binding site involved in drought-inducibility
CsDof21	HD-Zip 3	5	14.5	protein binding site
CsDof21	WUN-motif	1598	1607	wound-responsive element
CsDof22	ABRE	123	132	abscisic acid responsiveness
CsDof22	TGA-element	142	148	auxin-responsive element
CsDof22	TGA-element	186	192	auxin-responsive element
CsDof22	TC-rich repeats	84	93	cis-acting element involved in defense and stress responsiveness
CsDof22	TC-rich repeats	358	367	cis-acting element involved in defense and stress responsiveness
CsDof22	TCA-element	1490	1499	cis-acting element involved in salicylic acid responsiveness
CsDof22	AuxRR-core	1219	1226	cis-acting regulatory element involved in auxin responsiveness
CsDof22	TGACG-motif	896	901	cis-acting regulatory element involved in the MeJA-responsiveness
CsDof22	TGACG-motif	1039	1044	cis-acting regulatory element involved in the MeJA-responsiveness
CsDof22	CGTCA-motif	896	901	cis-acting regulatory element involved in the MeJA-responsiveness
CsDof22	CGTCA-motif	1039	1044	cis-acting regulatory element involved in the MeJA-responsiveness
CsDof22	CAT-box	127	133	cis-acting regulatory element related to meristem expression
CsDof22	CAAT-box	134	139	common cis-acting element in promoter and enhancer regions
CsDof22	CAAT-box	220	225	common cis-acting element in promoter and enhancer regions
CsDof22	CAAT-box	354	359	common cis-acting element in promoter and enhancer regions
CsDof22	CAAT-box	507	512	common cis-acting element in promoter and enhancer regions
CsDof22	CAAT-box	536	541	common cis-acting element in promoter and enhancer regions
CsDof22	CAAT-box	636	641	common cis-acting element in promoter and enhancer regions
CsDof22	CAAT-box	952	957	common cis-acting element in promoter and enhancer regions
CsDof22	CAAT-box	1000	1005	common cis-acting element in promoter and enhancer regions
CsDof22	CAAT-box	1186	1191	common cis-acting element in promoter and enhancer regions
CsDof22	CAAT-box	1214	1219	common cis-acting element in promoter and enhancer regions
CsDof22	CAAT-box	1236	1241	common cis-acting element in promoter and enhancer regions
CsDof22	CAAT-box	1246	1251	common cis-acting element in promoter and enhancer regions
CsDof22	CAAT-box	1344	1349	common cis-acting element in promoter and enhancer regions
CsDof22	CAAT-box	1598	1603	common cis-acting element in promoter and enhancer regions
CsDof22	CAAT-box	1790	1795	common cis-acting element in promoter and enhancer regions
CsDof22	TATA-box	565	571	core promoter element around -30 of transcription start
CsDof22	TATA-box	566	571	core promoter element around -30 of transcription start
CsDof22	TATA-box	567	571	core promoter element around -30 of transcription start
CsDof22	TATA-box	623	629	core promoter element around -30 of transcription start
CsDof22	TATA-box	624	629	core promoter element around -30 of transcription start
CsDof22	TATA-box	625	629	core promoter element around -30 of transcription start

Name	Cis-element	Start position	Stop position	Function
CsDof22	TATA-box	670	674	core promoter element around -30 of transcription start
CsDof22	TATA-box	698	707	core promoter element around -30 of transcription start
CsDof22	TATA-box	699	708	core promoter element around -30 of transcription start
CsDof22	TATA-box	700	706	core promoter element around -30 of transcription start
CsDof22	TATA-box	701	708	core promoter element around -30 of transcription start
CsDof22	TATA-box	702	708	core promoter element around -30 of transcription start
CsDof22	TATA-box	703	709	core promoter element around -30 of transcription start
CsDof22	TATA-box	704	710	core promoter element around -30 of transcription start
CsDof22	TATA-box	705	711	core promoter element around -30 of transcription start
CsDof22	TATA-box	706	710	core promoter element around -30 of transcription start
CsDof22	TATA-box	794	800	core promoter element around -30 of transcription start
CsDof22	TATA-box	795	800	core promoter element around -30 of transcription start
CsDof22	TATA-box	796	800	core promoter element around -30 of transcription start
CsDof22	TATA-box	808	814	core promoter element around -30 of transcription start
CsDof22	TATA-box	810	814	core promoter element around -30 of transcription start
CsDof22	TATA-box	820	826	core promoter element around -30 of transcription start
CsDof22	TATA-box	821	826	core promoter element around -30 of transcription start
CsDof22	TATA-box	822	826	core promoter element around -30 of transcription start
CsDof22	TATA-box	834	839	core promoter element around -30 of transcription start
CsDof22	TATA-box	835	839	core promoter element around -30 of transcription start
CsDof22	TATA-box	838	846	core promoter element around -30 of transcription start
CsDof22	TATA-box	1009	1013	core promoter element around -30 of transcription start
CsDof22	TATA-box	1081	1087	core promoter element around -30 of transcription start
CsDof22	TATA-box	1083	1087	core promoter element around -30 of transcription start
CsDof22	TATA-box	1091	1095	core promoter element around -30 of transcription start
CsDof22	TATA-box	1164	1170	core promoter element around -30 of transcription start
CsDof22	TATA-box	1166	1172	core promoter element around -30 of transcription start
CsDof22	TATA-box	1168	1172	core promoter element around -30 of transcription start
CsDof22	TATA-box	1174	1178	core promoter element around -30 of transcription start
CsDof22	TATA-box	1329	1336	core promoter element around -30 of transcription start
CsDof22	TATA-box	1330	1336	core promoter element around -30 of transcription start
CsDof22	TATA-box	1331	1336	core promoter element around -30 of transcription start
CsDof22	TATA-box	1332	1336	core promoter element around -30 of transcription start
CsDof22	TATA-box	1347	1353	core promoter element around -30 of transcription start
CsDof22	TATA-box	1348	1352	core promoter element around -30 of transcription start
CsDof22	TATA-box	1407	1411	core promoter element around -30 of transcription start
CsDof22	TATA-box	1523	1530	core promoter element around -30 of transcription start
CsDof22	TATA-box	1524	1530	core promoter element around -30 of transcription start
CsDof22	TATA-box	1525	1530	core promoter element around -30 of transcription start
CsDof22	TATA-box	1526	1530	core promoter element around -30 of transcription start
CsDof22	TATA-box	1690	1698	core promoter element around -30 of transcription start
CsDof22	TATA-box	1699	1705	core promoter element around -30 of transcription start
CsDof22	TATA-box	1700	1706	core promoter element around -30 of transcription start
CsDof22	TATA-box	1701	1707	core promoter element around -30 of transcription start
CsDof22	TATA-box	1703	1707	core promoter element around -30 of transcription start
CsDof22	TATA-box	1749	1756	core promoter element around -30 of transcription start
CsDof22	TATA-box	1750	1756	core promoter element around -30 of transcription start
CsDof22	TATA-box	1751	1757	core promoter element around -30 of transcription start
CsDof22	TATA-box	1752	1756	core promoter element around -30 of transcription start
CsDof22	TATA-box	1756	1762	core promoter element around -30 of transcription start
CsDof22	TATA-box	1758	1762	core promoter element around -30 of transcription start

Name	Cis-element	Start position	Stop position	Function
CsDof22	TATA-box	1764	1768	core promoter element around -30 of transcription start
CsDof22	AT-rich sequence	528	537	element for maximal elicitor-mediated activation (2copies)
CsDof22	MRE	684	691	MYB binding site involved in light responsiveness
CsDof22	HD-Zip 3	986	995.5	protein binding site
CsDof23	TC-rich repeats	93	102	cis-acting element involved in defense and stress responsiveness
CsDof23	TCA-element	1483	1492	cis-acting element involved in salicylic acid responsiveness
CsDof23	ARE	746	752	cis-acting regulatory element essential for the anaerobic induction
CsDof23	AuxRR-core	1209	1216	cis-acting regulatory element involved in auxin responsiveness
CsDof23	CGTCA-motif	358	363	cis-acting regulatory element involved in the MeJA-responsiveness
CsDof23	CGTCA-motif	900	905	cis-acting regulatory element involved in the MeJA-responsiveness
CsDof23	CGTCA-motif	1028	1033	cis-acting regulatory element involved in the MeJA-responsiveness
CsDof23	TGACG-motif	358	363	cis-acting regulatory element involved in the MeJA-responsiveness
CsDof23	TGACG-motif	900	905	cis-acting regulatory element involved in the MeJA-responsiveness
CsDof23	TGACG-motif	1028	1033	cis-acting regulatory element involved in the MeJA-responsiveness
CsDof23	CAT-box	1122	1128	cis-acting regulatory element related to meristem expression
CsDof23	CAAT-box	89	94	common cis-acting element in promoter and enhancer regions
CsDof23	CAAT-box	247	252	common cis-acting element in promoter and enhancer regions
CsDof23	CAAT-box	346	351	common cis-acting element in promoter and enhancer regions
CsDof23	CAAT-box	468	473	common cis-acting element in promoter and enhancer regions
CsDof23	CAAT-box	540	545	common cis-acting element in promoter and enhancer regions
CsDof23	CAAT-box	687	692	common cis-acting element in promoter and enhancer regions
CsDof23	CAAT-box	751	756	common cis-acting element in promoter and enhancer regions
CsDof23	CAAT-box	956	961	common cis-acting element in promoter and enhancer regions
CsDof23	CAAT-box	1004	1009	common cis-acting element in promoter and enhancer regions
CsDof23	CAAT-box	1011	1016	common cis-acting element in promoter and enhancer regions
CsDof23	CAAT-box	1176	1181	common cis-acting element in promoter and enhancer regions
CsDof23	CAAT-box	1204	1209	common cis-acting element in promoter and enhancer regions
CsDof23	CAAT-box	1226	1231	common cis-acting element in promoter and enhancer regions
CsDof23	CAAT-box	1236	1241	common cis-acting element in promoter and enhancer regions
CsDof23	CAAT-box	1335	1340	common cis-acting element in promoter and enhancer regions
CsDof23	CAAT-box	1593	1598	common cis-acting element in promoter and enhancer regions
CsDof23	CAAT-box	1788	1793	common cis-acting element in promoter and enhancer regions
CsDof23	TATA-box	222	230	core promoter element around -30 of transcription start
CsDof23	TATA-box	294	300	core promoter element around -30 of transcription start
CsDof23	TATA-box	296	300	core promoter element around -30 of transcription start
CsDof23	TATA-box	334	340	core promoter element around -30 of transcription start
CsDof23	TATA-box	335	339	core promoter element around -30 of transcription start
CsDof23	TATA-box	376	380	core promoter element around -30 of transcription start
CsDof23	TATA-box	398	407	core promoter element around -30 of transcription start
CsDof23	TATA-box	399	408	core promoter element around -30 of transcription start
CsDof23	TATA-box	400	406	core promoter element around -30 of transcription start
CsDof23	TATA-box	401	408	core promoter element around -30 of transcription start
CsDof23	TATA-box	402	408	core promoter element around -30 of transcription start
CsDof23	TATA-box	403	409	core promoter element around -30 of transcription start
CsDof23	TATA-box	404	408	core promoter element around -30 of transcription start
CsDof23	TATA-box	492	496	core promoter element around -30 of transcription start
CsDof23	TATA-box	591	600	core promoter element around -30 of transcription start
CsDof23	TATA-box	592	599	core promoter element around -30 of transcription start
CsDof23	TATA-box	593	599	core promoter element around -30 of transcription start
CsDof23	TATA-box	594	599	core promoter element around -30 of transcription start
CsDof23	TATA-box	595	599	core promoter element around -30 of transcription start

Name	Cis-element	Start position	Stop position	Function
CsDof23	TATA-box	631	642	core promoter element around -30 of transcription start
CsDof23	TATA-box	633	637	core promoter element around -30 of transcription start
CsDof23	TATA-box	708	714	core promoter element around -30 of transcription start
CsDof23	TATA-box	709	714	core promoter element around -30 of transcription start
CsDof23	TATA-box	710	714	core promoter element around -30 of transcription start
CsDof23	TATA-box	724	730	core promoter element around -30 of transcription start
CsDof23	TATA-box	725	730	core promoter element around -30 of transcription start
CsDof23	TATA-box	726	730	core promoter element around -30 of transcription start
CsDof23	TATA-box	764	769	core promoter element around -30 of transcription start
CsDof23	TATA-box	765	769	core promoter element around -30 of transcription start
CsDof23	TATA-box	799	805	core promoter element around -30 of transcription start
CsDof23	TATA-box	800	805	core promoter element around -30 of transcription start
CsDof23	TATA-box	801	805	core promoter element around -30 of transcription start
CsDof23	TATA-box	813	819	core promoter element around -30 of transcription start
CsDof23	TATA-box	815	819	core promoter element around -30 of transcription start
CsDof23	TATA-box	826	832	core promoter element around -30 of transcription start
CsDof23	TATA-box	827	832	core promoter element around -30 of transcription start
CsDof23	TATA-box	828	832	core promoter element around -30 of transcription start
CsDof23	TATA-box	837	842	core promoter element around -30 of transcription start
CsDof23	TATA-box	838	842	core promoter element around -30 of transcription start
CsDof23	TATA-box	1070	1076	core promoter element around -30 of transcription start
CsDof23	TATA-box	1072	1076	core promoter element around -30 of transcription start
CsDof23	TATA-box	1080	1084	core promoter element around -30 of transcription start
CsDof23	TATA-box	1154	1160	core promoter element around -30 of transcription start
CsDof23	TATA-box	1156	1162	core promoter element around -30 of transcription start
CsDof23	TATA-box	1158	1162	core promoter element around -30 of transcription start
CsDof23	TATA-box	1164	1168	core promoter element around -30 of transcription start
CsDof23	TATA-box	1320	1327	core promoter element around -30 of transcription start
CsDof23	TATA-box	1321	1327	core promoter element around -30 of transcription start
CsDof23	TATA-box	1322	1327	core promoter element around -30 of transcription start
CsDof23	TATA-box	1323	1327	core promoter element around -30 of transcription start
CsDof23	TATA-box	1338	1344	core promoter element around -30 of transcription start
CsDof23	TATA-box	1339	1343	core promoter element around -30 of transcription start
CsDof23	TATA-box	1397	1403	core promoter element around -30 of transcription start
CsDof23	TATA-box	1398	1404	core promoter element around -30 of transcription start
CsDof23	TATA-box	1399	1405	core promoter element around -30 of transcription start
CsDof23	TATA-box	1400	1406	core promoter element around -30 of transcription start
CsDof23	TATA-box	1401	1405	core promoter element around -30 of transcription start
CsDof23	TATA-box	1516	1523	core promoter element around -30 of transcription start
CsDof23	TATA-box	1517	1523	core promoter element around -30 of transcription start
CsDof23	TATA-box	1518	1523	core promoter element around -30 of transcription start
CsDof23	TATA-box	1519	1523	core promoter element around -30 of transcription start
CsDof23	TATA-box	1612	1616	core promoter element around -30 of transcription start
CsDof23	TATA-box	1687	1695	core promoter element around -30 of transcription start
CsDof23	TATA-box	1696	1700	core promoter element around -30 of transcription start
CsDof23	TATA-box	1749	1755	core promoter element around -30 of transcription start
CsDof23	TATA-box	1750	1754	core promoter element around -30 of transcription start
CsDof23	TATA-box	1754	1760	core promoter element around -30 of transcription start
CsDof23	TATA-box	1756	1760	core promoter element around -30 of transcription start
CsDof23	TATA-box	1762	1766	core promoter element around -30 of transcription start
CsDof23	AT-rich sequence	239	248	element for maximal elicitor-mediated activation (2copies)

Name	Cis-element	Start position	Stop position	Function
CsDof23	3-AF1 binding site	717	727	light responsive element
CsDof23	MBSI	1521	1531.5	MYB binding site involved in flavonoid biosynthetic genes regulation
CsDof23	MRE	383	390	MYB binding site involved in light responsiveness
CsDof23	HD-Zip 3	990	999.5	protein binding site
CsDof24	TGA-element	305	311	auxin-responsive element
CsDof24	TGA-element	1014	1020	auxin-responsive element
CsDof24	TGA-element	1362	1368	auxin-responsive element
CsDof24	AT-rich element	160	170	binding site of AT-rich DNA binding protein (ATBP-1)
CsDof24	AT-rich element	1217	1227	binding site of AT-rich DNA binding protein (ATBP-1)
CsDof24	TC-rich repeats	1626	1635	cis-acting element involved in defense and stress responsiveness
CsDof24	ARE	1935	1941	cis-acting regulatory element essential for the anaerobic induction
CsDof24	AuxRR-core	982	989	cis-acting regulatory element involved in auxin responsiveness
CsDof24	CAAT-box	25	30	common cis-acting element in promoter and enhancer regions
CsDof24	CAAT-box	292	297	common cis-acting element in promoter and enhancer regions
CsDof24	CAAT-box	301	306	common cis-acting element in promoter and enhancer regions
CsDof24	CAAT-box	421	426	common cis-acting element in promoter and enhancer regions
CsDof24	CAAT-box	1082	1087	common cis-acting element in promoter and enhancer regions
CsDof24	CAAT-box	1349	1354	common cis-acting element in promoter and enhancer regions
CsDof24	CAAT-box	1358	1363	common cis-acting element in promoter and enhancer regions
CsDof24	CAAT-box	1478	1483	common cis-acting element in promoter and enhancer regions
CsDof24	TATA-box	36	41	core promoter element around -30 of transcription start
CsDof24	TATA-box	37	41	core promoter element around -30 of transcription start
CsDof24	TATA-box	45	51	core promoter element around -30 of transcription start
CsDof24	TATA-box	46	51	core promoter element around -30 of transcription start
CsDof24	TATA-box	47	51	core promoter element around -30 of transcription start
CsDof24	TATA-box	135	141	core promoter element around -30 of transcription start
CsDof24	TATA-box	136	140	core promoter element around -30 of transcription start
CsDof24	TATA-box	172	178	core promoter element around -30 of transcription start
CsDof24	TATA-box	173	179	core promoter element around -30 of transcription start
CsDof24	TATA-box	174	180	core promoter element around -30 of transcription start
CsDof24	TATA-box	175	181	core promoter element around -30 of transcription start
CsDof24	TATA-box	176	180	core promoter element around -30 of transcription start
CsDof24	TATA-box	225	231	core promoter element around -30 of transcription start
CsDof24	TATA-box	226	230	core promoter element around -30 of transcription start
CsDof24	TATA-box	388	398	core promoter element around -30 of transcription start
CsDof24	TATA-box	389	396	core promoter element around -30 of transcription start
CsDof24	TATA-box	390	396	core promoter element around -30 of transcription start
CsDof24	TATA-box	391	398	core promoter element around -30 of transcription start
CsDof24	TATA-box	392	398	core promoter element around -30 of transcription start
CsDof24	TATA-box	393	399	core promoter element around -30 of transcription start
CsDof24	TATA-box	394	398	core promoter element around -30 of transcription start
CsDof24	TATA-box	403	408	core promoter element around -30 of transcription start
CsDof24	TATA-box	404	408	core promoter element around -30 of transcription start
CsDof24	TATA-box	427	436	core promoter element around -30 of transcription start
CsDof24	TATA-box	429	433	core promoter element around -30 of transcription start
CsDof24	TATA-box	454	460	core promoter element around -30 of transcription start
CsDof24	TATA-box	455	459	core promoter element around -30 of transcription start
CsDof24	TATA-box	512	516	core promoter element around -30 of transcription start
CsDof24	TATA-box	517	524	core promoter element around -30 of transcription start
CsDof24	TATA-box	518	524	core promoter element around -30 of transcription start
CsDof24	TATA-box	519	525	core promoter element around -30 of transcription start

Name	Cis-element	Start position	Stop position	Function
CsDof24	TATA-box	520	526	core promoter element around -30 of transcription start
CsDof24	TATA-box	521	527	core promoter element around -30 of transcription start
CsDof24	TATA-box	522	528	core promoter element around -30 of transcription start
CsDof24	TATA-box	523	529	core promoter element around -30 of transcription start
CsDof24	TATA-box	524	528	core promoter element around -30 of transcription start
CsDof24	TATA-box	1093	1098	core promoter element around -30 of transcription start
CsDof24	TATA-box	1094	1098	core promoter element around -30 of transcription start
CsDof24	TATA-box	1102	1108	core promoter element around -30 of transcription start
CsDof24	TATA-box	1103	1108	core promoter element around -30 of transcription start
CsDof24	TATA-box	1104	1108	core promoter element around -30 of transcription start
CsDof24	TATA-box	1192	1198	core promoter element around -30 of transcription start
CsDof24	TATA-box	1193	1197	core promoter element around -30 of transcription start
CsDof24	TATA-box	1229	1235	core promoter element around -30 of transcription start
CsDof24	TATA-box	1230	1236	core promoter element around -30 of transcription start
CsDof24	TATA-box	1231	1237	core promoter element around -30 of transcription start
CsDof24	TATA-box	1232	1238	core promoter element around -30 of transcription start
CsDof24	TATA-box	1233	1237	core promoter element around -30 of transcription start
CsDof24	TATA-box	1282	1288	core promoter element around -30 of transcription start
CsDof24	TATA-box	1283	1287	core promoter element around -30 of transcription start
CsDof24	TATA-box	1445	1455	core promoter element around -30 of transcription start
CsDof24	TATA-box	1446	1453	core promoter element around -30 of transcription start
CsDof24	TATA-box	1447	1453	core promoter element around -30 of transcription start
CsDof24	TATA-box	1448	1455	core promoter element around -30 of transcription start
CsDof24	TATA-box	1449	1455	core promoter element around -30 of transcription start
CsDof24	TATA-box	1450	1456	core promoter element around -30 of transcription start
CsDof24	TATA-box	1451	1455	core promoter element around -30 of transcription start
CsDof24	TATA-box	1460	1465	core promoter element around -30 of transcription start
CsDof24	TATA-box	1461	1465	core promoter element around -30 of transcription start
CsDof24	TATA-box	1484	1493	core promoter element around -30 of transcription start
CsDof24	TATA-box	1486	1490	core promoter element around -30 of transcription start
CsDof24	TATA-box	1511	1517	core promoter element around -30 of transcription start
CsDof24	TATA-box	1512	1516	core promoter element around -30 of transcription start
CsDof24	TATA-box	1569	1573	core promoter element around -30 of transcription start
CsDof24	TATA-box	1574	1581	core promoter element around -30 of transcription start
CsDof24	TATA-box	1575	1581	core promoter element around -30 of transcription start
CsDof24	TATA-box	1576	1582	core promoter element around -30 of transcription start
CsDof24	TATA-box	1577	1583	core promoter element around -30 of transcription start
CsDof24	TATA-box	1578	1584	core promoter element around -30 of transcription start
CsDof24	TATA-box	1579	1585	core promoter element around -30 of transcription start
CsDof24	TATA-box	1580	1586	core promoter element around -30 of transcription start
CsDof24	TATA-box	1581	1585	core promoter element around -30 of transcription start
CsDof24	TATA-box	1689	1695	core promoter element around -30 of transcription start
CsDof24	TATA-box	1690	1694	core promoter element around -30 of transcription start
CsDof24	TATA-box	1719	1726	core promoter element around -30 of transcription start
CsDof24	TATA-box	1730	1736	core promoter element around -30 of transcription start
CsDof24	TATA-box	1732	1736	core promoter element around -30 of transcription start
CsDof24	TATA-box	1745	1752	core promoter element around -30 of transcription start
CsDof24	TATA-box	1746	1752	core promoter element around -30 of transcription start
CsDof24	TATA-box	1747	1753	core promoter element around -30 of transcription start
CsDof24	TATA-box	1748	1752	core promoter element around -30 of transcription start
CsDof24	TATA-box	1760	1769	core promoter element around -30 of transcription start

Name	Cis-element	Start position	Stop position	Function
CsDof24	TATA-box	1761	1767	core promoter element around -30 of transcription start
CsDof24	TATA-box	1762	1768	core promoter element around -30 of transcription start
CsDof24	TATA-box	1763	1769	core promoter element around -30 of transcription start
CsDof24	TATA-box	1764	1770	core promoter element around -30 of transcription start
CsDof24	TATA-box	1765	1771	core promoter element around -30 of transcription start
CsDof24	TATA-box	1766	1772	core promoter element around -30 of transcription start
CsDof24	TATA-box	1767	1773	core promoter element around -30 of transcription start
CsDof24	TATA-box	1768	1772	core promoter element around -30 of transcription start
CsDof24	GT1-motif	1033	1039	light responsive element
CsDof24	WUN-motif	1602	1611	wound-responsive element
CsDof25	TGA-element	542	548	auxin-responsive element
CsDof25	TC-rich repeats	1344	1353	cis-acting element involved in defense and stress responsiveness
CsDof25	TC-rich repeats	1794	1803	cis-acting element involved in defense and stress responsiveness
CsDof25	ACE	1349	1358	cis-acting element involved in light responsiveness
CsDof25	TCA-element	1242	1251	cis-acting element involved in salicylic acid responsiveness
CsDof25	ARE	364	370	cis-acting regulatory element essential for the anaerobic induction
CsDof25	ARE	1325	1331	cis-acting regulatory element essential for the anaerobic induction
CsDof25	circadian	584	593	cis-acting regulatory element involved in circadian control
CsDof25	CGTCA-motif	38	43	cis-acting regulatory element involved in the MeJA-responsiveness
CsDof25	TGACG-motif	38	43	cis-acting regulatory element involved in the MeJA-responsiveness
CsDof25	CAAT-box	136	141	common cis-acting element in promoter and enhancer regions
CsDof25	CAAT-box	144	149	common cis-acting element in promoter and enhancer regions
CsDof25	CAAT-box	168	173	common cis-acting element in promoter and enhancer regions
CsDof25	CAAT-box	221	226	common cis-acting element in promoter and enhancer regions
CsDof25	CAAT-box	237	242	common cis-acting element in promoter and enhancer regions
CsDof25	CAAT-box	308	313	common cis-acting element in promoter and enhancer regions
CsDof25	CAAT-box	341	346	common cis-acting element in promoter and enhancer regions
CsDof25	CAAT-box	394	399	common cis-acting element in promoter and enhancer regions
CsDof25	CAAT-box	739	744	common cis-acting element in promoter and enhancer regions
CsDof25	CAAT-box	854	859	common cis-acting element in promoter and enhancer regions
CsDof25	CAAT-box	874	879	common cis-acting element in promoter and enhancer regions
CsDof25	CAAT-box	1057	1062	common cis-acting element in promoter and enhancer regions
CsDof25	CAAT-box	1156	1161	common cis-acting element in promoter and enhancer regions
CsDof25	CAAT-box	1251	1256	common cis-acting element in promoter and enhancer regions
CsDof25	CAAT-box	1424	1429	common cis-acting element in promoter and enhancer regions
CsDof25	CAAT-box	1529	1534	common cis-acting element in promoter and enhancer regions
CsDof25	CAAT-box	1687	1692	common cis-acting element in promoter and enhancer regions
CsDof25	TATA-box	204	213	core promoter element around -30 of transcription start
CsDof25	TATA-box	205	211	core promoter element around -30 of transcription start
CsDof25	TATA-box	206	212	core promoter element around -30 of transcription start
CsDof25	TATA-box	207	213	core promoter element around -30 of transcription start
CsDof25	TATA-box	208	214	core promoter element around -30 of transcription start
CsDof25	TATA-box	209	215	core promoter element around -30 of transcription start
CsDof25	TATA-box	210	216	core promoter element around -30 of transcription start
CsDof25	TATA-box	211	217	core promoter element around -30 of transcription start
CsDof25	TATA-box	212	224	core promoter element around -30 of transcription start
CsDof25	TATA-box	213	219	core promoter element around -30 of transcription start
CsDof25	TATA-box	214	220	core promoter element around -30 of transcription start
CsDof25	TATA-box	215	221	core promoter element around -30 of transcription start
CsDof25	TATA-box	216	222	core promoter element around -30 of transcription start
CsDof25	TATA-box	217	223	core promoter element around -30 of transcription start

Name	Cis-element	Start position	Stop position	Function
CsDof25	TATA-box	218	222	core promoter element around -30 of transcription start
CsDof25	TATA-box	399	404	core promoter element around -30 of transcription start
CsDof25	TATA-box	400	404	core promoter element around -30 of transcription start
CsDof25	TATA-box	450	456	core promoter element around -30 of transcription start
CsDof25	TATA-box	451	455	core promoter element around -30 of transcription start
CsDof25	TATA-box	621	625	core promoter element around -30 of transcription start
CsDof25	TATA-box	656	662	core promoter element around -30 of transcription start
CsDof25	TATA-box	657	663	core promoter element around -30 of transcription start
CsDof25	TATA-box	659	663	core promoter element around -30 of transcription start
CsDof25	TATA-box	681	686	core promoter element around -30 of transcription start
CsDof25	TATA-box	682	686	core promoter element around -30 of transcription start
CsDof25	TATA-box	702	708	core promoter element around -30 of transcription start
CsDof25	TATA-box	703	708	core promoter element around -30 of transcription start
CsDof25	TATA-box	704	708	core promoter element around -30 of transcription start
CsDof25	TATA-box	898	902	core promoter element around -30 of transcription start
CsDof25	TATA-box	969	974	core promoter element around -30 of transcription start
CsDof25	TATA-box	970	974	core promoter element around -30 of transcription start
CsDof25	TATA-box	979	984	core promoter element around -30 of transcription start
CsDof25	TATA-box	980	984	core promoter element around -30 of transcription start
CsDof25	TATA-box	1020	1027	core promoter element around -30 of transcription start
CsDof25	TATA-box	1023	1029	core promoter element around -30 of transcription start
CsDof25	TATA-box	1025	1029	core promoter element around -30 of transcription start
CsDof25	TATA-box	1030	1037	core promoter element around -30 of transcription start
CsDof25	TATA-box	1060	1066	core promoter element around -30 of transcription start
CsDof25	TATA-box	1061	1067	core promoter element around -30 of transcription start
CsDof25	TATA-box	1062	1068	core promoter element around -30 of transcription start
CsDof25	TATA-box	1063	1069	core promoter element around -30 of transcription start
CsDof25	TATA-box	1064	1070	core promoter element around -30 of transcription start
CsDof25	TATA-box	1065	1069	core promoter element around -30 of transcription start
CsDof25	TATA-box	1124	1130	core promoter element around -30 of transcription start
CsDof25	TATA-box	1125	1129	core promoter element around -30 of transcription start
CsDof25	TATA-box	1200	1208	core promoter element around -30 of transcription start
CsDof25	TATA-box	1217	1221	core promoter element around -30 of transcription start
CsDof25	TATA-box	1402	1408	core promoter element around -30 of transcription start
CsDof25	TATA-box	1403	1408	core promoter element around -30 of transcription start
CsDof25	TATA-box	1404	1408	core promoter element around -30 of transcription start
CsDof25	TATA-box	1406	1415	core promoter element around -30 of transcription start
CsDof25	TATA-box	1471	1479	core promoter element around -30 of transcription start
CsDof25	TATA-box	1472	1479	core promoter element around -30 of transcription start
CsDof25	TATA-box	1473	1479	core promoter element around -30 of transcription start
CsDof25	TATA-box	1474	1479	core promoter element around -30 of transcription start
CsDof25	TATA-box	1475	1479	core promoter element around -30 of transcription start
CsDof25	TATA-box	1506	1510	core promoter element around -30 of transcription start
CsDof25	TATA-box	1514	1526	core promoter element around -30 of transcription start
CsDof25	TATA-box	1516	1522	core promoter element around -30 of transcription start
CsDof25	TATA-box	1517	1523	core promoter element around -30 of transcription start
CsDof25	TATA-box	1518	1524	core promoter element around -30 of transcription start
CsDof25	TATA-box	1519	1525	core promoter element around -30 of transcription start
CsDof25	TATA-box	1520	1526	core promoter element around -30 of transcription start
CsDof25	TATA-box	1521	1527	core promoter element around -30 of transcription start
CsDof25	TATA-box	1523	1527	core promoter element around -30 of transcription start

Name	Cis-element	Start position	Stop position	Function
CsDof25	TATA-box	1532	1538	core promoter element around -30 of transcription start
CsDof25	TATA-box	1533	1539	core promoter element around -30 of transcription start
CsDof25	TATA-box	1534	1540	core promoter element around -30 of transcription start
CsDof25	TATA-box	1535	1539	core promoter element around -30 of transcription start
CsDof25	TATA-box	1540	1548	core promoter element around -30 of transcription start
CsDof25	TATA-box	1541	1548	core promoter element around -30 of transcription start
CsDof25	TATA-box	1542	1548	core promoter element around -30 of transcription start
CsDof25	TATA-box	1543	1548	core promoter element around -30 of transcription start
CsDof25	TATA-box	1544	1548	core promoter element around -30 of transcription start
CsDof25	TATA-box	1549	1553	core promoter element around -30 of transcription start
CsDof25	TATA-box	1554	1560	core promoter element around -30 of transcription start
CsDof25	TATA-box	1555	1559	core promoter element around -30 of transcription start
CsDof25	TATA-box	1576	1581	core promoter element around -30 of transcription start
CsDof25	TATA-box	1577	1581	core promoter element around -30 of transcription start
CsDof25	TATA-box	1638	1644	core promoter element around -30 of transcription start
CsDof25	TATA-box	1639	1645	core promoter element around -30 of transcription start
CsDof25	TATA-box	1640	1646	core promoter element around -30 of transcription start
CsDof25	TATA-box	1641	1647	core promoter element around -30 of transcription start
CsDof25	TATA-box	1643	1647	core promoter element around -30 of transcription start
CsDof25	TATA-box	1648	1654	core promoter element around -30 of transcription start
CsDof25	TATA-box	1649	1655	core promoter element around -30 of transcription start
CsDof25	TATA-box	1651	1655	core promoter element around -30 of transcription start
CsDof25	TATA-box	1662	1668	core promoter element around -30 of transcription start
CsDof25	TATA-box	1663	1669	core promoter element around -30 of transcription start
CsDof25	TATA-box	1664	1670	core promoter element around -30 of transcription start
CsDof25	TATA-box	1665	1671	core promoter element around -30 of transcription start
CsDof25	TATA-box	1666	1672	core promoter element around -30 of transcription start
CsDof25	TATA-box	1667	1673	core promoter element around -30 of transcription start
CsDof25	TATA-box	1668	1674	core promoter element around -30 of transcription start
CsDof25	TATA-box	1669	1675	core promoter element around -30 of transcription start
CsDof25	TATA-box	1670	1676	core promoter element around -30 of transcription start
CsDof25	TATA-box	1671	1677	core promoter element around -30 of transcription start
CsDof25	TATA-box	1673	1677	core promoter element around -30 of transcription start
CsDof25	TATA-box	1683	1687	core promoter element around -30 of transcription start
CsDof25	TATA-box	1714	1720	core promoter element around -30 of transcription start
CsDof25	TATA-box	1715	1721	core promoter element around -30 of transcription start
CsDof25	TATA-box	1716	1722	core promoter element around -30 of transcription start
CsDof25	TATA-box	1717	1723	core promoter element around -30 of transcription start
CsDof25	TATA-box	1718	1724	core promoter element around -30 of transcription start
CsDof25	TATA-box	1719	1723	core promoter element around -30 of transcription start
CsDof25	P-box	1841	1848	gibberellin-responsive element
CsDof25	GT1-motif	780	787	light responsive element
CsDof25	GT1-motif	781	787	light responsive element
CsDof25	MBS	73	79	MYB binding site involved in drought-inducibility
CsDof26	ABRE	147	154	abscisic acid responsiveness
CsDof26	ABRE	164	173	abscisic acid responsiveness
CsDof26	TC-rich repeats	121	130	cis-acting element involved in defense and stress responsiveness
CsDof26	ARE	346	352	cis-acting regulatory element essential for the anaerobic induction
CsDof26	circadian	1919	1928	cis-acting regulatory element involved in circadian control
CsDof26	CAAT-box	237	242	common cis-acting element in promoter and enhancer regions
CsDof26	CAAT-box	392	397	common cis-acting element in promoter and enhancer regions

Name	Cis-element	Start position	Stop position	Function
CsDof26	CAAT-box	444	449	common cis-acting element in promoter and enhancer regions
CsDof26	CAAT-box	612	617	common cis-acting element in promoter and enhancer regions
CsDof26	CAAT-box	633	643	common cis-acting element in promoter and enhancer regions
CsDof26	CAAT-box	659	664	common cis-acting element in promoter and enhancer regions
CsDof26	CAAT-box	871	876	common cis-acting element in promoter and enhancer regions
CsDof26	CAAT-box	930	935	common cis-acting element in promoter and enhancer regions
CsDof26	CAAT-box	1065	1070	common cis-acting element in promoter and enhancer regions
CsDof26	CAAT-box	1087	1092	common cis-acting element in promoter and enhancer regions
CsDof26	CAAT-box	1407	1412	common cis-acting element in promoter and enhancer regions
CsDof26	CAAT-box	1566	1571	common cis-acting element in promoter and enhancer regions
CsDof26	CAAT-box	1746	1751	common cis-acting element in promoter and enhancer regions
CsDof26	TATA-box	18	27	core promoter element around -30 of transcription start
CsDof26	TATA-box	20	24	core promoter element around -30 of transcription start
CsDof26	TATA-box	48	52	core promoter element around -30 of transcription start
CsDof26	TATA-box	68	74	core promoter element around -30 of transcription start
CsDof26	TATA-box	69	73	core promoter element around -30 of transcription start
CsDof26	TATA-box	152	156	core promoter element around -30 of transcription start
CsDof26	TATA-box	198	204	core promoter element around -30 of transcription start
CsDof26	TATA-box	199	203	core promoter element around -30 of transcription start
CsDof26	TATA-box	212	216	core promoter element around -30 of transcription start
CsDof26	TATA-box	230	236	core promoter element around -30 of transcription start
CsDof26	TATA-box	231	237	core promoter element around -30 of transcription start
CsDof26	TATA-box	233	237	core promoter element around -30 of transcription start
CsDof26	TATA-box	240	246	core promoter element around -30 of transcription start
CsDof26	TATA-box	241	245	core promoter element around -30 of transcription start
CsDof26	TATA-box	268	274	core promoter element around -30 of transcription start
CsDof26	TATA-box	269	275	core promoter element around -30 of transcription start
CsDof26	TATA-box	270	276	core promoter element around -30 of transcription start
CsDof26	TATA-box	271	275	core promoter element around -30 of transcription start
CsDof26	TATA-box	357	364	core promoter element around -30 of transcription start
CsDof26	TATA-box	400	406	core promoter element around -30 of transcription start
CsDof26	TATA-box	401	406	core promoter element around -30 of transcription start
CsDof26	TATA-box	402	406	core promoter element around -30 of transcription start
CsDof26	TATA-box	528	537	core promoter element around -30 of transcription start
CsDof26	TATA-box	529	536	core promoter element around -30 of transcription start
CsDof26	TATA-box	530	536	core promoter element around -30 of transcription start
CsDof26	TATA-box	531	538	core promoter element around -30 of transcription start
CsDof26	TATA-box	532	538	core promoter element around -30 of transcription start
CsDof26	TATA-box	534	538	core promoter element around -30 of transcription start
CsDof26	TATA-box	541	547	core promoter element around -30 of transcription start
CsDof26	TATA-box	543	547	core promoter element around -30 of transcription start
CsDof26	TATA-box	559	563	core promoter element around -30 of transcription start
CsDof26	TATA-box	572	578	core promoter element around -30 of transcription start
CsDof26	TATA-box	574	578	core promoter element around -30 of transcription start
CsDof26	TATA-box	680	686	core promoter element around -30 of transcription start
CsDof26	TATA-box	682	688	core promoter element around -30 of transcription start
CsDof26	TATA-box	684	688	core promoter element around -30 of transcription start
CsDof26	TATA-box	734	738	core promoter element around -30 of transcription start
CsDof26	TATA-box	740	744	core promoter element around -30 of transcription start
CsDof26	TATA-box	806	811	core promoter element around -30 of transcription start
CsDof26	TATA-box	807	811	core promoter element around -30 of transcription start

Name	Cis-element	Start position	Stop position	Function
CsDof26	TATA-box	830	834	core promoter element around -30 of transcription start
CsDof26	TATA-box	846	852	core promoter element around -30 of transcription start
CsDof26	TATA-box	848	852	core promoter element around -30 of transcription start
CsDof26	TATA-box	875	881	core promoter element around -30 of transcription start
CsDof26	TATA-box	877	881	core promoter element around -30 of transcription start
CsDof26	TATA-box	1001	1012	core promoter element around -30 of transcription start
CsDof26	TATA-box	1003	1011	core promoter element around -30 of transcription start
CsDof26	TATA-box	1004	1011	core promoter element around -30 of transcription start
CsDof26	TATA-box	1005	1011	core promoter element around -30 of transcription start
CsDof26	TATA-box	1006	1011	core promoter element around -30 of transcription start
CsDof26	TATA-box	1007	1011	core promoter element around -30 of transcription start
CsDof26	TATA-box	1047	1051	core promoter element around -30 of transcription start
CsDof26	TATA-box	1077	1081	core promoter element around -30 of transcription start
CsDof26	TATA-box	1132	1138	core promoter element around -30 of transcription start
CsDof26	TATA-box	1133	1138	core promoter element around -30 of transcription start
CsDof26	TATA-box	1134	1138	core promoter element around -30 of transcription start
CsDof26	TATA-box	1551	1559	core promoter element around -30 of transcription start
CsDof26	TATA-box	1556	1562	core promoter element around -30 of transcription start
CsDof26	TATA-box	1557	1561	core promoter element around -30 of transcription start
CsDof26	TATA-box	1646	1652	core promoter element around -30 of transcription start
CsDof26	TATA-box	1647	1651	core promoter element around -30 of transcription start
CsDof26	TATA-box	1661	1668	core promoter element around -30 of transcription start
CsDof26	TATA-box	1768	1774	core promoter element around -30 of transcription start
CsDof26	TATA-box	1769	1773	core promoter element around -30 of transcription start
CsDof26	TATA-box	1801	1810	core promoter element around -30 of transcription start
CsDof26	TATA-box	1802	1811	core promoter element around -30 of transcription start
CsDof26	TATA-box	1803	1809	core promoter element around -30 of transcription start
CsDof26	TATA-box	1804	1811	core promoter element around -30 of transcription start
CsDof26	TATA-box	1805	1811	core promoter element around -30 of transcription start
CsDof26	TATA-box	1806	1812	core promoter element around -30 of transcription start
CsDof26	TATA-box	1807	1811	core promoter element around -30 of transcription start
CsDof26	TATA-box	1951	1957	core promoter element around -30 of transcription start
CsDof26	TATA-box	1952	1958	core promoter element around -30 of transcription start
CsDof26	TATA-box	1953	1957	core promoter element around -30 of transcription start
CsDof26	GT1-motif	1470	1476	light responsive element
CsDof27	ABRE	754	763	abscisic acid responsiveness
CsDof27	ABRE	756	762	abscisic acid responsiveness
CsDof27	ABRE	757	762	abscisic acid responsiveness
CsDof27	ABRE	958	967	abscisic acid responsiveness
CsDof27	ABRE	960	966	abscisic acid responsiveness
CsDof27	ABRE	961	966	abscisic acid responsiveness
CsDof27	ABRE	1049	1055	abscisic acid responsiveness
CsDof27	ABRE	1050	1055	abscisic acid responsiveness
CsDof27	ABRE	1070	1075	abscisic acid responsiveness
CsDof27	ABRE	1119	1125	abscisic acid responsiveness
CsDof27	ABRE	1120	1125	abscisic acid responsiveness
CsDof27	TATC-box	705	712	cis-acting element involved in gibberellin-responsiveness
CsDof27	LTR	1093	1099	cis-acting element involved in low-temperature responsiveness
CsDof27	ARE	1601	1607	cis-acting regulatory element essential for the anaerobic induction
CsDof27	AuxRR-core	1036	1043	cis-acting regulatory element involved in auxin responsiveness
CsDof27	G-box	753	765	cis-acting regulatory element involved in light responsiveness

Name	Cis-element	Start position	Stop position	Function
CsDof27	G-box	754	763	cis-acting regulatory element involved in light responsiveness
CsDof27	G-box	756	762	cis-acting regulatory element involved in light responsiveness
CsDof27	G-box	958	967	cis-acting regulatory element involved in light responsiveness
CsDof27	G-box	960	966	cis-acting regulatory element involved in light responsiveness
CsDof27	G-box	1049	1055	cis-acting regulatory element involved in light responsiveness
CsDof27	G-box	1069	1075	cis-acting regulatory element involved in light responsiveness
CsDof27	G-box	1117	1126	cis-acting regulatory element involved in light responsiveness
CsDof27	G-box	1119	1125	cis-acting regulatory element involved in light responsiveness
CsDof27	G-Box	756	762	cis-acting regulatory element involved in light responsiveness
CsDof27	G-Box	960	966	cis-acting regulatory element involved in light responsiveness
CsDof27	G-Box	1049	1055	cis-acting regulatory element involved in light responsiveness
CsDof27	G-Box	1119	1125	cis-acting regulatory element involved in light responsiveness
CsDof27	TGACG-motif	416	421	cis-acting regulatory element involved in the MeJA-responsiveness
CsDof27	TGACG-motif	427	432	cis-acting regulatory element involved in the MeJA-responsiveness
CsDof27	TGACG-motif	990	995	cis-acting regulatory element involved in the MeJA-responsiveness
CsDof27	TGACG-motif	1068	1073	cis-acting regulatory element involved in the MeJA-responsiveness
CsDof27	TGACG-motif	1377	1382	cis-acting regulatory element involved in the MeJA-responsiveness
CsDof27	TGACG-motif	1878	1883	cis-acting regulatory element involved in the MeJA-responsiveness
CsDof27	CGTCA-motif	416	421	cis-acting regulatory element involved in the MeJA-responsiveness
CsDof27	CGTCA-motif	427	432	cis-acting regulatory element involved in the MeJA-responsiveness
CsDof27	CGTCA-motif	990	995	cis-acting regulatory element involved in the MeJA-responsiveness
CsDof27	CGTCA-motif	1068	1073	cis-acting regulatory element involved in the MeJA-responsiveness
CsDof27	CGTCA-motif	1377	1382	cis-acting regulatory element involved in the MeJA-responsiveness
CsDof27	CGTCA-motif	1878	1883	cis-acting regulatory element involved in the MeJA-responsiveness
CsDof27	CAT-box	1715	1721	cis-acting regulatory element related to meristem expression
CsDof27	CAAT-box	44	49	common cis-acting element in promoter and enhancer regions
CsDof27	CAAT-box	179	184	common cis-acting element in promoter and enhancer regions
CsDof27	CAAT-box	645	650	common cis-acting element in promoter and enhancer regions
CsDof27	CAAT-box	888	893	common cis-acting element in promoter and enhancer regions
CsDof27	CAAT-box	976	981	common cis-acting element in promoter and enhancer regions
CsDof27	CAAT-box	1041	1046	common cis-acting element in promoter and enhancer regions
CsDof27	CAAT-box	1304	1309	common cis-acting element in promoter and enhancer regions
CsDof27	CAAT-box	1409	1414	common cis-acting element in promoter and enhancer regions
CsDof27	TATA-box	109	113	core promoter element around -30 of transcription start
CsDof27	TATA-box	275	283	core promoter element around -30 of transcription start
CsDof27	TATA-box	280	286	core promoter element around -30 of transcription start
CsDof27	TATA-box	281	286	core promoter element around -30 of transcription start
CsDof27	TATA-box	282	286	core promoter element around -30 of transcription start
CsDof27	TATA-box	295	299	core promoter element around -30 of transcription start
CsDof27	TATA-box	353	359	core promoter element around -30 of transcription start
CsDof27	TATA-box	354	358	core promoter element around -30 of transcription start
CsDof27	TATA-box	368	373	core promoter element around -30 of transcription start
CsDof27	TATA-box	369	373	core promoter element around -30 of transcription start
CsDof27	TATA-box	391	395	core promoter element around -30 of transcription start
CsDof27	TATA-box	436	443	core promoter element around -30 of transcription start
CsDof27	TATA-box	437	443	core promoter element around -30 of transcription start
CsDof27	TATA-box	438	443	core promoter element around -30 of transcription start
CsDof27	TATA-box	439	443	core promoter element around -30 of transcription start
CsDof27	TATA-box	481	485	core promoter element around -30 of transcription start
CsDof27	TATA-box	548	554	core promoter element around -30 of transcription start
CsDof27	TATA-box	549	554	core promoter element around -30 of transcription start

Name	Cis-element	Start position	Stop position	Function
CsDof27	TATA-box	550	554	core promoter element around -30 of transcription start
CsDof27	TATA-box	622	628	core promoter element around -30 of transcription start
CsDof27	TATA-box	623	628	core promoter element around -30 of transcription start
CsDof27	TATA-box	624	628	core promoter element around -30 of transcription start
CsDof27	TATA-box	651	656	core promoter element around -30 of transcription start
CsDof27	TATA-box	652	656	core promoter element around -30 of transcription start
CsDof27	TATA-box	909	915	core promoter element around -30 of transcription start
CsDof27	TATA-box	911	915	core promoter element around -30 of transcription start
CsDof27	TATA-box	938	944	core promoter element around -30 of transcription start
CsDof27	TATA-box	939	943	core promoter element around -30 of transcription start
CsDof27	TATA-box	1080	1088	core promoter element around -30 of transcription start
CsDof27	TATA-box	1501	1508	core promoter element around -30 of transcription start
CsDof27	TATA-box	1576	1584	core promoter element around -30 of transcription start
CsDof27	TATA-box	1578	1587	core promoter element around -30 of transcription start
CsDof27	TATA-box	1579	1584	core promoter element around -30 of transcription start
CsDof27	TATA-box	1580	1584	core promoter element around -30 of transcription start
CsDof27	TATA-box	1592	1599	core promoter element around -30 of transcription start
CsDof27	TATA-box	1666	1672	core promoter element around -30 of transcription start
CsDof27	TATA-box	1667	1671	core promoter element around -30 of transcription start
CsDof27	TATA-box	1965	1969	core promoter element around -30 of transcription start
CsDof27	TATA-box	1991	1998	core promoter element around -30 of transcription start
CsDof27	TATA-box	1992	1998	core promoter element around -30 of transcription start
CsDof27	TATA-box	1993	1998	core promoter element around -30 of transcription start
CsDof27	TATA-box	1994	1998	core promoter element around -30 of transcription start
CsDof28	ABRE	177	183	abscisic acid responsiveness
CsDof28	ABRE	178	183	abscisic acid responsiveness
CsDof28	ABRE	628	633	abscisic acid responsiveness
CsDof28	ABRE	655	661	abscisic acid responsiveness
CsDof28	ABRE	656	661	abscisic acid responsiveness
CsDof28	TGA-element	194	200	auxin-responsive element
CsDof28	TGA-element	244	250	auxin-responsive element
CsDof28	ARE	758	764	cis-acting regulatory element essential for the anaerobic induction
CsDof28	ARE	1444	1450	cis-acting regulatory element essential for the anaerobic induction
CsDof28	G-Box	177	183	cis-acting regulatory element involved in light responsiveness
CsDof28	G-Box	628	634	cis-acting regulatory element involved in light responsiveness
CsDof28	G-Box	655	661	cis-acting regulatory element involved in light responsiveness
CsDof28	G-box	177	183	cis-acting regulatory element involved in light responsiveness
CsDof28	G-box	655	661	cis-acting regulatory element involved in light responsiveness
CsDof28	G-box	725	731	cis-acting regulatory element involved in light responsiveness
CsDof28	TGACG-motif	1266	1271	cis-acting regulatory element involved in the MeJA-responsiveness
CsDof28	CGTCA-motif	1266	1271	cis-acting regulatory element involved in the MeJA-responsiveness
CsDof28	CAAT-box	19	24	common cis-acting element in promoter and enhancer regions
CsDof28	CAAT-box	42	47	common cis-acting element in promoter and enhancer regions
CsDof28	CAAT-box	93	98	common cis-acting element in promoter and enhancer regions
CsDof28	CAAT-box	166	171	common cis-acting element in promoter and enhancer regions
CsDof28	CAAT-box	270	275	common cis-acting element in promoter and enhancer regions
CsDof28	CAAT-box	526	531	common cis-acting element in promoter and enhancer regions
CsDof28	CAAT-box	770	775	common cis-acting element in promoter and enhancer regions
CsDof28	CAAT-box	837	842	common cis-acting element in promoter and enhancer regions
CsDof28	CAAT-box	953	958	common cis-acting element in promoter and enhancer regions
CsDof28	CAAT-box	1025	1030	common cis-acting element in promoter and enhancer regions

Name	Cis-element	Start position	Stop position	Function
CsDof28	CAAT-box	1442	1447	common cis-acting element in promoter and enhancer regions
CsDof28	CAAT-box	1468	1473	common cis-acting element in promoter and enhancer regions
CsDof28	CAAT-box	1499	1504	common cis-acting element in promoter and enhancer regions
CsDof28	CAAT-box	1593	1598	common cis-acting element in promoter and enhancer regions
CsDof28	TATA-box	35	41	core promoter element around -30 of transcription start
CsDof28	TATA-box	36	41	core promoter element around -30 of transcription start
CsDof28	TATA-box	37	41	core promoter element around -30 of transcription start
CsDof28	TATA-box	112	118	core promoter element around -30 of transcription start
CsDof28	TATA-box	113	117	core promoter element around -30 of transcription start
CsDof28	TATA-box	432	438	core promoter element around -30 of transcription start
CsDof28	TATA-box	433	439	core promoter element around -30 of transcription start
CsDof28	TATA-box	435	439	core promoter element around -30 of transcription start
CsDof28	TATA-box	437	445	core promoter element around -30 of transcription start
CsDof28	TATA-box	687	694	core promoter element around -30 of transcription start
CsDof28	TATA-box	688	694	core promoter element around -30 of transcription start
CsDof28	TATA-box	689	694	core promoter element around -30 of transcription start
CsDof28	TATA-box	690	694	core promoter element around -30 of transcription start
CsDof28	TATA-box	704	710	core promoter element around -30 of transcription start
CsDof28	TATA-box	705	711	core promoter element around -30 of transcription start
CsDof28	TATA-box	706	710	core promoter element around -30 of transcription start
CsDof28	TATA-box	762	770	core promoter element around -30 of transcription start
CsDof28	TATA-box	765	770	core promoter element around -30 of transcription start
CsDof28	TATA-box	766	770	core promoter element around -30 of transcription start
CsDof28	TATA-box	906	912	core promoter element around -30 of transcription start
CsDof28	TATA-box	908	912	core promoter element around -30 of transcription start
CsDof28	TATA-box	1046	1050	core promoter element around -30 of transcription start
CsDof28	TATA-box	1070	1076	core promoter element around -30 of transcription start
CsDof28	TATA-box	1071	1077	core promoter element around -30 of transcription start
CsDof28	TATA-box	1072	1078	core promoter element around -30 of transcription start
CsDof28	TATA-box	1073	1077	core promoter element around -30 of transcription start
CsDof28	TATA-box	1082	1091	core promoter element around -30 of transcription start
CsDof28	TATA-box	1083	1089	core promoter element around -30 of transcription start
CsDof28	TATA-box	1084	1091	core promoter element around -30 of transcription start
CsDof28	TATA-box	1085	1091	core promoter element around -30 of transcription start
CsDof28	TATA-box	1086	1092	core promoter element around -30 of transcription start
CsDof28	TATA-box	1087	1091	core promoter element around -30 of transcription start
CsDof28	TATA-box	1113	1119	core promoter element around -30 of transcription start
CsDof28	TATA-box	1114	1118	core promoter element around -30 of transcription start
CsDof28	TATA-box	1129	1135	core promoter element around -30 of transcription start
CsDof28	TATA-box	1130	1134	core promoter element around -30 of transcription start
CsDof28	TATA-box	1207	1211	core promoter element around -30 of transcription start
CsDof28	TATA-box	1222	1231	core promoter element around -30 of transcription start
CsDof28	TATA-box	1226	1232	core promoter element around -30 of transcription start
CsDof28	TATA-box	1227	1233	core promoter element around -30 of transcription start
CsDof28	TATA-box	1228	1232	core promoter element around -30 of transcription start
CsDof28	TATA-box	1258	1264	core promoter element around -30 of transcription start
CsDof28	TATA-box	1259	1265	core promoter element around -30 of transcription start
CsDof28	TATA-box	1260	1266	core promoter element around -30 of transcription start
CsDof28	TATA-box	1261	1265	core promoter element around -30 of transcription start
CsDof28	TATA-box	1285	1291	core promoter element around -30 of transcription start
CsDof28	TATA-box	1286	1290	core promoter element around -30 of transcription start

Name	Cis-element	Start position	Stop position	Function
CsDof28	TATA-box	1298	1305	core promoter element around -30 of transcription start
CsDof28	TATA-box	1322	1326	core promoter element around -30 of transcription start
CsDof28	TATA-box	1347	1354	core promoter element around -30 of transcription start
CsDof28	TATA-box	1393	1397	core promoter element around -30 of transcription start
CsDof28	TATA-box	1403	1409	core promoter element around -30 of transcription start
CsDof28	TATA-box	1405	1409	core promoter element around -30 of transcription start
CsDof28	TATA-box	1528	1532	core promoter element around -30 of transcription start
CsDof28	TATA-box	1550	1554	core promoter element around -30 of transcription start
CsDof28	TATA-box	1578	1584	core promoter element around -30 of transcription start
CsDof28	TATA-box	1579	1585	core promoter element around -30 of transcription start
CsDof28	TATA-box	1580	1584	core promoter element around -30 of transcription start
CsDof28	TATA-box	1641	1650	core promoter element around -30 of transcription start
CsDof28	TATA-box	1650	1656	core promoter element around -30 of transcription start
CsDof28	TATA-box	1651	1657	core promoter element around -30 of transcription start
CsDof28	TATA-box	1652	1658	core promoter element around -30 of transcription start
CsDof28	TATA-box	1653	1657	core promoter element around -30 of transcription start
CsDof28	TATA-box	1758	1764	core promoter element around -30 of transcription start
CsDof28	TATA-box	1759	1764	core promoter element around -30 of transcription start
CsDof28	TATA-box	1760	1764	core promoter element around -30 of transcription start
CsDof28	P-box	1731	1738	gibberellin-responsive element
CsDof28	GT1-motif	9	15	light responsive element
CsDof28	Sp1	1310	1316	light responsive element
CsDof28	MBS	287	293	MYB binding site involved in drought-inducibility
CsDof28	MRE	1200	1207	MYB binding site involved in light responsiveness
CsDof28	HD-Zip 3	1537	1547	protein binding site
CsDof29	ABRE	1000	1007	abscisic acid responsiveness
CsDof29	ABRE	1453	1458	abscisic acid responsiveness
CsDof29	ABRE	1750	1755	abscisic acid responsiveness
CsDof29	TGA-element	195	201	auxin-responsive element
CsDof29	ARE	362	368	cis-acting regulatory element essential for the anaerobic induction
CsDof29	ARE	442	448	cis-acting regulatory element essential for the anaerobic induction
CsDof29	G-Box	1452	1458	cis-acting regulatory element involved in light responsiveness
CsDof29	G-Box	1750	1756	cis-acting regulatory element involved in light responsiveness
CsDof29	O2-site	1308	1318	cis-acting regulatory element involved in zein metabolism regulation
CsDof29	GCN4_motif	1096	1103	cis-regulatory element involved in endosperm expression
CsDof29	CAAT-box	166	171	common cis-acting element in promoter and enhancer regions
CsDof29	CAAT-box	180	185	common cis-acting element in promoter and enhancer regions
CsDof29	CAAT-box	287	292	common cis-acting element in promoter and enhancer regions
CsDof29	CAAT-box	342	347	common cis-acting element in promoter and enhancer regions
CsDof29	CAAT-box	466	471	common cis-acting element in promoter and enhancer regions
CsDof29	CAAT-box	581	586	common cis-acting element in promoter and enhancer regions
CsDof29	CAAT-box	980	985	common cis-acting element in promoter and enhancer regions
CsDof29	CAAT-box	1120	1125	common cis-acting element in promoter and enhancer regions
CsDof29	CAAT-box	1343	1348	common cis-acting element in promoter and enhancer regions
CsDof29	CAAT-box	1635	1640	common cis-acting element in promoter and enhancer regions
CsDof29	CAAT-box	1836	1841	common cis-acting element in promoter and enhancer regions
CsDof29	TATA-box	13	17	core promoter element around -30 of transcription start
CsDof29	TATA-box	222	228	core promoter element around -30 of transcription start
CsDof29	TATA-box	223	229	core promoter element around -30 of transcription start
CsDof29	TATA-box	225	229	core promoter element around -30 of transcription start
CsDof29	TATA-box	253	259	core promoter element around -30 of transcription start

Name	Cis-element	Start position	Stop position	Function
CsDof29	TATA-box	254	258	core promoter element around -30 of transcription start
CsDof29	TATA-box	329	333	core promoter element around -30 of transcription start
CsDof29	TATA-box	336	340	core promoter element around -30 of transcription start
CsDof29	TATA-box	356	362	core promoter element around -30 of transcription start
CsDof29	TATA-box	357	362	core promoter element around -30 of transcription start
CsDof29	TATA-box	358	362	core promoter element around -30 of transcription start
CsDof29	TATA-box	371	375	core promoter element around -30 of transcription start
CsDof29	TATA-box	497	504	core promoter element around -30 of transcription start
CsDof29	TATA-box	655	659	core promoter element around -30 of transcription start
CsDof29	TATA-box	786	790	core promoter element around -30 of transcription start
CsDof29	TATA-box	844	848	core promoter element around -30 of transcription start
CsDof29	TATA-box	1026	1033	core promoter element around -30 of transcription start
CsDof29	TATA-box	1027	1033	core promoter element around -30 of transcription start
CsDof29	TATA-box	1028	1033	core promoter element around -30 of transcription start
CsDof29	TATA-box	1029	1033	core promoter element around -30 of transcription start
CsDof29	TATA-box	1036	1048	core promoter element around -30 of transcription start
CsDof29	TATA-box	1038	1044	core promoter element around -30 of transcription start
CsDof29	TATA-box	1039	1045	core promoter element around -30 of transcription start
CsDof29	TATA-box	1040	1046	core promoter element around -30 of transcription start
CsDof29	TATA-box	1041	1047	core promoter element around -30 of transcription start
CsDof29	TATA-box	1042	1048	core promoter element around -30 of transcription start
CsDof29	TATA-box	1043	1049	core promoter element around -30 of transcription start
CsDof29	TATA-box	1044	1050	core promoter element around -30 of transcription start
CsDof29	TATA-box	1045	1051	core promoter element around -30 of transcription start
CsDof29	TATA-box	1046	1052	core promoter element around -30 of transcription start
CsDof29	TATA-box	1047	1053	core promoter element around -30 of transcription start
CsDof29	TATA-box	1048	1054	core promoter element around -30 of transcription start
CsDof29	TATA-box	1049	1053	core promoter element around -30 of transcription start
CsDof29	TATA-box	1053	1059	core promoter element around -30 of transcription start
CsDof29	TATA-box	1055	1061	core promoter element around -30 of transcription start
CsDof29	TATA-box	1056	1062	core promoter element around -30 of transcription start
CsDof29	TATA-box	1057	1061	core promoter element around -30 of transcription start
CsDof29	TATA-box	1064	1070	core promoter element around -30 of transcription start
CsDof29	TATA-box	1065	1071	core promoter element around -30 of transcription start
CsDof29	TATA-box	1066	1072	core promoter element around -30 of transcription start
CsDof29	TATA-box	1067	1071	core promoter element around -30 of transcription start
CsDof29	TATA-box	1145	1151	core promoter element around -30 of transcription start
CsDof29	TATA-box	1146	1152	core promoter element around -30 of transcription start
CsDof29	TATA-box	1147	1151	core promoter element around -30 of transcription start
CsDof29	TATA-box	1169	1176	core promoter element around -30 of transcription start
CsDof29	TATA-box	1336	1342	core promoter element around -30 of transcription start
CsDof29	TATA-box	1337	1341	core promoter element around -30 of transcription start
CsDof29	TATA-box	1365	1369	core promoter element around -30 of transcription start
CsDof29	TATA-box	1377	1384	core promoter element around -30 of transcription start
CsDof29	TATA-box	1402	1407	core promoter element around -30 of transcription start
CsDof29	TATA-box	1403	1407	core promoter element around -30 of transcription start
CsDof29	TATA-box	1437	1443	core promoter element around -30 of transcription start
CsDof29	TATA-box	1438	1443	core promoter element around -30 of transcription start
CsDof29	TATA-box	1439	1443	core promoter element around -30 of transcription start
CsDof29	TATA-box	1503	1507	core promoter element around -30 of transcription start
CsDof29	TATA-box	1508	1514	core promoter element around -30 of transcription start

Name	Cis-element	Start position	Stop position	Function
CsDof29	TATA-box	1509	1515	core promoter element around -30 of transcription start
CsDof29	TATA-box	1510	1516	core promoter element around -30 of transcription start
CsDof29	TATA-box	1511	1517	core promoter element around -30 of transcription start
CsDof29	TATA-box	1512	1518	core promoter element around -30 of transcription start
CsDof29	TATA-box	1513	1519	core promoter element around -30 of transcription start
CsDof29	TATA-box	1514	1520	core promoter element around -30 of transcription start
CsDof29	TATA-box	1515	1521	core promoter element around -30 of transcription start
CsDof29	TATA-box	1516	1522	core promoter element around -30 of transcription start
CsDof29	TATA-box	1517	1523	core promoter element around -30 of transcription start
CsDof29	TATA-box	1518	1524	core promoter element around -30 of transcription start
CsDof29	TATA-box	1519	1525	core promoter element around -30 of transcription start
CsDof29	TATA-box	1521	1525	core promoter element around -30 of transcription start
CsDof29	TATA-box	1629	1635	core promoter element around -30 of transcription start
CsDof29	TATA-box	1630	1634	core promoter element around -30 of transcription start
CsDof29	TATA-box	1669	1675	core promoter element around -30 of transcription start
CsDof29	TATA-box	1671	1675	core promoter element around -30 of transcription start
CsDof29	TATA-box	1810	1816	core promoter element around -30 of transcription start
CsDof29	TATA-box	1811	1815	core promoter element around -30 of transcription start
CsDof29	TATA-box	1868	1874	core promoter element around -30 of transcription start
CsDof29	TATA-box	1869	1875	core promoter element around -30 of transcription start
CsDof29	TATA-box	1870	1876	core promoter element around -30 of transcription start
CsDof29	TATA-box	1872	1876	core promoter element around -30 of transcription start
CsDof29	TATA-box	1900	1909	core promoter element around -30 of transcription start
CsDof29	TATA-box	1901	1907	core promoter element around -30 of transcription start
CsDof29	TATA-box	1902	1909	core promoter element around -30 of transcription start
CsDof29	TATA-box	1903	1909	core promoter element around -30 of transcription start
CsDof29	TATA-box	1904	1910	core promoter element around -30 of transcription start
CsDof29	TATA-box	1905	1909	core promoter element around -30 of transcription start
CsDof29	MBS	421	427	MYB binding site involved in drought-inducibility
CsDof30	ABRE	1420	1425	abscisic acid responsiveness
CsDof30	ARE	1843	1849	cis-acting regulatory element essential for the anaerobic induction
CsDof30	AuxRR-core	1609	1616	cis-acting regulatory element involved in auxin responsiveness
CsDof30	G-box	1419	1425	cis-acting regulatory element involved in light responsiveness
CsDof30	CGTCA-motif	1418	1423	cis-acting regulatory element involved in the MeJA-responsiveness
CsDof30	TGACG-motif	1418	1423	cis-acting regulatory element involved in the MeJA-responsiveness
CsDof30	CAT-box	1031	1037	cis-acting regulatory element related to meristem expression
CsDof30	CAAT-box	7	12	common cis-acting element in promoter and enhancer regions
CsDof30	CAAT-box	32	37	common cis-acting element in promoter and enhancer regions
CsDof30	CAAT-box	93	98	common cis-acting element in promoter and enhancer regions
CsDof30	CAAT-box	190	195	common cis-acting element in promoter and enhancer regions
CsDof30	CAAT-box	367	372	common cis-acting element in promoter and enhancer regions
CsDof30	CAAT-box	711	716	common cis-acting element in promoter and enhancer regions
CsDof30	CAAT-box	767	772	common cis-acting element in promoter and enhancer regions
CsDof30	CAAT-box	877	882	common cis-acting element in promoter and enhancer regions
CsDof30	CAAT-box	1406	1411	common cis-acting element in promoter and enhancer regions
CsDof30	CAAT-box	1655	1660	common cis-acting element in promoter and enhancer regions
CsDof30	CAAT-box	1866	1871	common cis-acting element in promoter and enhancer regions
CsDof30	TATA-box	16	22	core promoter element around -30 of transcription start
CsDof30	TATA-box	17	23	core promoter element around -30 of transcription start
CsDof30	TATA-box	18	24	core promoter element around -30 of transcription start
CsDof30	TATA-box	19	23	core promoter element around -30 of transcription start

Name	Cis-element	Start position	Stop position	Function
CsDof30	TATA-box	64	68	core promoter element around -30 of transcription start
CsDof30	TATA-box	103	109	core promoter element around -30 of transcription start
CsDof30	TATA-box	104	111	core promoter element around -30 of transcription start
CsDof30	TATA-box	105	111	core promoter element around -30 of transcription start
CsDof30	TATA-box	106	112	core promoter element around -30 of transcription start
CsDof30	TATA-box	107	111	core promoter element around -30 of transcription start
CsDof30	TATA-box	247	254	core promoter element around -30 of transcription start
CsDof30	TATA-box	248	254	core promoter element around -30 of transcription start
CsDof30	TATA-box	249	254	core promoter element around -30 of transcription start
CsDof30	TATA-box	250	254	core promoter element around -30 of transcription start
CsDof30	TATA-box	396	401	core promoter element around -30 of transcription start
CsDof30	TATA-box	397	401	core promoter element around -30 of transcription start
CsDof30	TATA-box	460	466	core promoter element around -30 of transcription start
CsDof30	TATA-box	461	465	core promoter element around -30 of transcription start
CsDof30	TATA-box	509	517	core promoter element around -30 of transcription start
CsDof30	TATA-box	514	520	core promoter element around -30 of transcription start
CsDof30	TATA-box	515	521	core promoter element around -30 of transcription start
CsDof30	TATA-box	516	522	core promoter element around -30 of transcription start
CsDof30	TATA-box	517	521	core promoter element around -30 of transcription start
CsDof30	TATA-box	661	670	core promoter element around -30 of transcription start
CsDof30	TATA-box	662	669	core promoter element around -30 of transcription start
CsDof30	TATA-box	663	669	core promoter element around -30 of transcription start
CsDof30	TATA-box	664	671	core promoter element around -30 of transcription start
CsDof30	TATA-box	665	671	core promoter element around -30 of transcription start
CsDof30	TATA-box	666	672	core promoter element around -30 of transcription start
CsDof30	TATA-box	667	671	core promoter element around -30 of transcription start
CsDof30	TATA-box	706	712	core promoter element around -30 of transcription start
CsDof30	TATA-box	708	712	core promoter element around -30 of transcription start
CsDof30	TATA-box	723	729	core promoter element around -30 of transcription start
CsDof30	TATA-box	724	730	core promoter element around -30 of transcription start
CsDof30	TATA-box	725	731	core promoter element around -30 of transcription start
CsDof30	TATA-box	726	730	core promoter element around -30 of transcription start
CsDof30	TATA-box	824	830	core promoter element around -30 of transcription start
CsDof30	TATA-box	826	830	core promoter element around -30 of transcription start
CsDof30	TATA-box	843	849	core promoter element around -30 of transcription start
CsDof30	TATA-box	844	848	core promoter element around -30 of transcription start
CsDof30	TATA-box	856	860	core promoter element around -30 of transcription start
CsDof30	TATA-box	864	868	core promoter element around -30 of transcription start
CsDof30	TATA-box	1208	1214	core promoter element around -30 of transcription start
CsDof30	TATA-box	1209	1215	core promoter element around -30 of transcription start
CsDof30	TATA-box	1210	1214	core promoter element around -30 of transcription start
CsDof30	TATA-box	1220	1226	core promoter element around -30 of transcription start
CsDof30	TATA-box	1221	1228	core promoter element around -30 of transcription start
CsDof30	TATA-box	1222	1228	core promoter element around -30 of transcription start
CsDof30	TATA-box	1224	1228	core promoter element around -30 of transcription start
CsDof30	TATA-box	1231	1235	core promoter element around -30 of transcription start
CsDof30	TATA-box	1248	1252	core promoter element around -30 of transcription start
CsDof30	TATA-box	1298	1304	core promoter element around -30 of transcription start
CsDof30	TATA-box	1299	1304	core promoter element around -30 of transcription start
CsDof30	TATA-box	1300	1304	core promoter element around -30 of transcription start
CsDof30	TATA-box	1310	1316	core promoter element around -30 of transcription start

Name	Cis-element	Start position	Stop position	Function
CsDof30	TATA-box	1311	1317	core promoter element around -30 of transcription start
CsDof30	TATA-box	1312	1318	core promoter element around -30 of transcription start
CsDof30	TATA-box	1313	1317	core promoter element around -30 of transcription start
CsDof30	TATA-box	1439	1446	core promoter element around -30 of transcription start
CsDof30	TATA-box	1440	1446	core promoter element around -30 of transcription start
CsDof30	TATA-box	1441	1446	core promoter element around -30 of transcription start
CsDof30	TATA-box	1442	1446	core promoter element around -30 of transcription start
CsDof30	TATA-box	1620	1632	core promoter element around -30 of transcription start
CsDof30	TATA-box	1622	1628	core promoter element around -30 of transcription start
CsDof30	TATA-box	1623	1629	core promoter element around -30 of transcription start
CsDof30	TATA-box	1624	1630	core promoter element around -30 of transcription start
CsDof30	TATA-box	1625	1631	core promoter element around -30 of transcription start
CsDof30	TATA-box	1626	1632	core promoter element around -30 of transcription start
CsDof30	TATA-box	1627	1633	core promoter element around -30 of transcription start
CsDof30	TATA-box	1628	1634	core promoter element around -30 of transcription start
CsDof30	TATA-box	1629	1635	core promoter element around -30 of transcription start
CsDof30	TATA-box	1630	1636	core promoter element around -30 of transcription start
CsDof30	TATA-box	1631	1637	core promoter element around -30 of transcription start
CsDof30	TATA-box	1632	1638	core promoter element around -30 of transcription start
CsDof30	TATA-box	1633	1639	core promoter element around -30 of transcription start
CsDof30	TATA-box	1634	1640	core promoter element around -30 of transcription start
CsDof30	TATA-box	1635	1641	core promoter element around -30 of transcription start
CsDof30	TATA-box	1636	1642	core promoter element around -30 of transcription start
CsDof30	TATA-box	1637	1643	core promoter element around -30 of transcription start
CsDof30	TATA-box	1639	1643	core promoter element around -30 of transcription start
CsDof30	TATA-box	1660	1664	core promoter element around -30 of transcription start
CsDof30	TATA-box	1835	1841	core promoter element around -30 of transcription start
CsDof30	TATA-box	1836	1842	core promoter element around -30 of transcription start
CsDof30	TATA-box	1837	1841	core promoter element around -30 of transcription start
CsDof30	P-box	306	313	gibberellin-responsive element
CsDof30	GT1-motif	1481	1490	light responsive element
CsDof30	MBS	1847	1853	MYB binding site involved in drought-inducibility
CsDof31	ABRE	603	608	abscisic acid responsiveness
CsDof31	ABRE	931	938	abscisic acid responsiveness
CsDof31	ABRE	1021	1030	abscisic acid responsiveness
CsDof31	ABRE	1024	1029	abscisic acid responsiveness
CsDof31	TGA-element	1357	1363	auxin-responsive element
CsDof31	LTR	126	132	cis-acting element involved in low-temperature responsiveness
CsDof31	ARE	1009	1015	cis-acting regulatory element essential for the anaerobic induction
CsDof31	ARE	1313	1319	cis-acting regulatory element essential for the anaerobic induction
CsDof31	ARE	1840	1846	cis-acting regulatory element essential for the anaerobic induction
CsDof31	G-Box	602	608	cis-acting regulatory element involved in light responsiveness
CsDof31	G-Box	1023	1029	cis-acting regulatory element involved in light responsiveness
CsDof31	CGTCA-motif	1148	1153	cis-acting regulatory element involved in the MeJA-responsiveness
CsDof31	TGACG-motif	1148	1153	cis-acting regulatory element involved in the MeJA-responsiveness
CsDof31	CAT-box	255	261	cis-acting regulatory element related to meristem expression
CsDof31	CAAT-box	87	92	common cis-acting element in promoter and enhancer regions
CsDof31	CAAT-box	90	95	common cis-acting element in promoter and enhancer regions
CsDof31	CAAT-box	142	147	common cis-acting element in promoter and enhancer regions
CsDof31	CAAT-box	211	216	common cis-acting element in promoter and enhancer regions
CsDof31	CAAT-box	333	338	common cis-acting element in promoter and enhancer regions

Name	Cis-element	Start position	Stop position	Function
CsDof31	CAAT-box	437	442	common cis-acting element in promoter and enhancer regions
CsDof31	CAAT-box	499	504	common cis-acting element in promoter and enhancer regions
CsDof31	CAAT-box	532	537	common cis-acting element in promoter and enhancer regions
CsDof31	CAAT-box	554	559	common cis-acting element in promoter and enhancer regions
CsDof31	CAAT-box	570	575	common cis-acting element in promoter and enhancer regions
CsDof31	CAAT-box	720	725	common cis-acting element in promoter and enhancer regions
CsDof31	CAAT-box	1382	1387	common cis-acting element in promoter and enhancer regions
CsDof31	CAAT-box	1421	1426	common cis-acting element in promoter and enhancer regions
CsDof31	CAAT-box	1654	1664	common cis-acting element in promoter and enhancer regions
CsDof31	CAAT-box	1657	1662	common cis-acting element in promoter and enhancer regions
CsDof31	TATA-box	19	23	core promoter element around -30 of transcription start
CsDof31	TATA-box	28	34	core promoter element around -30 of transcription start
CsDof31	TATA-box	29	35	core promoter element around -30 of transcription start
CsDof31	TATA-box	30	36	core promoter element around -30 of transcription start
CsDof31	TATA-box	31	35	core promoter element around -30 of transcription start
CsDof31	TATA-box	100	106	core promoter element around -30 of transcription start
CsDof31	TATA-box	102	106	core promoter element around -30 of transcription start
CsDof31	TATA-box	176	183	core promoter element around -30 of transcription start
CsDof31	TATA-box	177	183	core promoter element around -30 of transcription start
CsDof31	TATA-box	179	183	core promoter element around -30 of transcription start
CsDof31	TATA-box	313	319	core promoter element around -30 of transcription start
CsDof31	TATA-box	315	319	core promoter element around -30 of transcription start
CsDof31	TATA-box	408	412	core promoter element around -30 of transcription start
CsDof31	TATA-box	502	508	core promoter element around -30 of transcription start
CsDof31	TATA-box	503	508	core promoter element around -30 of transcription start
CsDof31	TATA-box	504	508	core promoter element around -30 of transcription start
CsDof31	TATA-box	579	586	core promoter element around -30 of transcription start
CsDof31	TATA-box	734	738	core promoter element around -30 of transcription start
CsDof31	TATA-box	739	743	core promoter element around -30 of transcription start
CsDof31	TATA-box	829	836	core promoter element around -30 of transcription start
CsDof31	TATA-box	830	836	core promoter element around -30 of transcription start
CsDof31	TATA-box	831	837	core promoter element around -30 of transcription start
CsDof31	TATA-box	832	836	core promoter element around -30 of transcription start
CsDof31	TATA-box	836	842	core promoter element around -30 of transcription start
CsDof31	TATA-box	837	844	core promoter element around -30 of transcription start
CsDof31	TATA-box	838	844	core promoter element around -30 of transcription start
CsDof31	TATA-box	839	845	core promoter element around -30 of transcription start
CsDof31	TATA-box	840	844	core promoter element around -30 of transcription start
CsDof31	TATA-box	851	857	core promoter element around -30 of transcription start
CsDof31	TATA-box	853	859	core promoter element around -30 of transcription start
CsDof31	TATA-box	854	860	core promoter element around -30 of transcription start
CsDof31	TATA-box	855	859	core promoter element around -30 of transcription start
CsDof31	TATA-box	861	867	core promoter element around -30 of transcription start
CsDof31	TATA-box	862	866	core promoter element around -30 of transcription start
CsDof31	TATA-box	877	885	core promoter element around -30 of transcription start
CsDof31	TATA-box	1013	1020	core promoter element around -30 of transcription start
CsDof31	TATA-box	1014	1020	core promoter element around -30 of transcription start
CsDof31	TATA-box	1015	1020	core promoter element around -30 of transcription start
CsDof31	TATA-box	1016	1020	core promoter element around -30 of transcription start
CsDof31	TATA-box	1133	1139	core promoter element around -30 of transcription start
CsDof31	TATA-box	1134	1140	core promoter element around -30 of transcription start

Name	Cis-element	Start position	Stop position	Function
CsDof31	TATA-box	1135	1141	core promoter element around -30 of transcription start
CsDof31	TATA-box	1136	1140	core promoter element around -30 of transcription start
CsDof31	TATA-box	1223	1230	core promoter element around -30 of transcription start
CsDof31	TATA-box	1224	1230	core promoter element around -30 of transcription start
CsDof31	TATA-box	1225	1230	core promoter element around -30 of transcription start
CsDof31	TATA-box	1226	1230	core promoter element around -30 of transcription start
CsDof31	TATA-box	1253	1259	core promoter element around -30 of transcription start
CsDof31	TATA-box	1254	1260	core promoter element around -30 of transcription start
CsDof31	TATA-box	1255	1261	core promoter element around -30 of transcription start
CsDof31	TATA-box	1256	1260	core promoter element around -30 of transcription start
CsDof31	TATA-box	1301	1307	core promoter element around -30 of transcription start
CsDof31	TATA-box	1302	1307	core promoter element around -30 of transcription start
CsDof31	TATA-box	1303	1307	core promoter element around -30 of transcription start
CsDof31	TATA-box	1415	1419	core promoter element around -30 of transcription start
CsDof31	TATA-box	1674	1682	core promoter element around -30 of transcription start
CsDof31	TATA-box	1678	1684	core promoter element around -30 of transcription start
CsDof31	TATA-box	1679	1684	core promoter element around -30 of transcription start
CsDof31	TATA-box	1680	1684	core promoter element around -30 of transcription start
CsDof31	TATA-box	1900	1904	core promoter element around -30 of transcription start
CsDof31	AT-rich sequence	764	773	element for maximal elicitor-mediated activation (2copies)
CsDof31	P-box	384	391	gibberellin-responsive element
CsDof31	Sp1	246	252	light responsive element
CsDof31	GT1-motif	557	564	light responsive element
CsDof31	GT1-motif	558	564	light responsive element
CsDof31	GT1-motif	1722	1729	light responsive element
CsDof31	GT1-motif	1723	1729	light responsive element
CsDof31	GT1-motif	1932	1938	light responsive element
CsDof31	MRE	39	46	MYB binding site involved in light responsiveness
CsDof31	CCAAT-box	1319	1325	MYBHv1 binding site
CsDof32	ABRE	1897	1903	abscisic acid responsiveness
CsDof32	ABRE	1898	1903	abscisic acid responsiveness
CsDof32	TC-rich repeats	95	104	cis-acting element involved in defense and stress responsiveness
CsDof32	ARE	1082	1088	cis-acting regulatory element essential for the anaerobic induction
CsDof32	G-box	1387	1393	cis-acting regulatory element involved in light responsiveness
CsDof32	G-box	1896	1905	cis-acting regulatory element involved in light responsiveness
CsDof32	G-box	1897	1903	cis-acting regulatory element involved in light responsiveness
CsDof32	G-Box	1897	1903	cis-acting regulatory element involved in light responsiveness
CsDof32	RY-element	1943	1951	cis-acting regulatory element involved in seed-specific regulation
CsDof32	CGTCA-motif	893	898	cis-acting regulatory element involved in the MeJA-responsiveness
CsDof32	TGACG-motif	893	898	cis-acting regulatory element involved in the MeJA-responsiveness
CsDof32	O2-site	672	680	cis-acting regulatory element involved in zein metabolism regulation
CsDof32	CAT-box	461	467	cis-acting regulatory element related to meristem expression
CsDof32	CAAT-box	156	161	common cis-acting element in promoter and enhancer regions
CsDof32	CAAT-box	475	480	common cis-acting element in promoter and enhancer regions
CsDof32	CAAT-box	712	717	common cis-acting element in promoter and enhancer regions
CsDof32	CAAT-box	737	742	common cis-acting element in promoter and enhancer regions
CsDof32	CAAT-box	929	934	common cis-acting element in promoter and enhancer regions
CsDof32	CAAT-box	1227	1232	common cis-acting element in promoter and enhancer regions
CsDof32	CAAT-box	1465	1470	common cis-acting element in promoter and enhancer regions
CsDof32	CAAT-box	1639	1644	common cis-acting element in promoter and enhancer regions
CsDof32	CAAT-box	1658	1663	common cis-acting element in promoter and enhancer regions

Name	Cis-element	Start position	Stop position	Function
CsDof32	CAAT-box	1861	1866	common cis-acting element in promoter and enhancer regions
CsDof32	TATA-box	41	45	core promoter element around -30 of transcription start
CsDof32	TATA-box	135	141	core promoter element around -30 of transcription start
CsDof32	TATA-box	136	141	core promoter element around -30 of transcription start
CsDof32	TATA-box	137	141	core promoter element around -30 of transcription start
CsDof32	TATA-box	483	490	core promoter element around -30 of transcription start
CsDof32	TATA-box	945	949	core promoter element around -30 of transcription start
CsDof32	TATA-box	1093	1100	core promoter element around -30 of transcription start
CsDof32	TATA-box	1094	1100	core promoter element around -30 of transcription start
CsDof32	TATA-box	1095	1101	core promoter element around -30 of transcription start
CsDof32	TATA-box	1096	1100	core promoter element around -30 of transcription start
CsDof32	TATA-box	1126	1133	core promoter element around -30 of transcription start
CsDof32	TATA-box	1127	1133	core promoter element around -30 of transcription start
CsDof32	TATA-box	1128	1135	core promoter element around -30 of transcription start
CsDof32	TATA-box	1129	1135	core promoter element around -30 of transcription start
CsDof32	TATA-box	1131	1135	core promoter element around -30 of transcription start
CsDof32	TATA-box	1277	1281	core promoter element around -30 of transcription start
CsDof32	TATA-box	1349	1355	core promoter element around -30 of transcription start
CsDof32	TATA-box	1350	1355	core promoter element around -30 of transcription start
CsDof32	TATA-box	1351	1355	core promoter element around -30 of transcription start
CsDof32	TATA-box	1394	1400	core promoter element around -30 of transcription start
CsDof32	TATA-box	1395	1399	core promoter element around -30 of transcription start
CsDof32	TATA-box	1477	1483	core promoter element around -30 of transcription start
CsDof32	TATA-box	1478	1484	core promoter element around -30 of transcription start
CsDof32	TATA-box	1480	1484	core promoter element around -30 of transcription start
CsDof32	TATA-box	1491	1496	core promoter element around -30 of transcription start
CsDof32	TATA-box	1492	1496	core promoter element around -30 of transcription start
CsDof32	TATA-box	1516	1522	core promoter element around -30 of transcription start
CsDof32	TATA-box	1517	1521	core promoter element around -30 of transcription start
CsDof32	TATA-box	1569	1574	core promoter element around -30 of transcription start
CsDof32	TATA-box	1570	1574	core promoter element around -30 of transcription start
CsDof32	TATA-box	1575	1579	core promoter element around -30 of transcription start
CsDof32	TATA-box	1600	1606	core promoter element around -30 of transcription start
CsDof32	TATA-box	1601	1607	core promoter element around -30 of transcription start
CsDof32	TATA-box	1603	1607	core promoter element around -30 of transcription start
CsDof32	TATA-box	1773	1777	core promoter element around -30 of transcription start
CsDof32	TATA-box	1789	1795	core promoter element around -30 of transcription start
CsDof32	TATA-box	1790	1796	core promoter element around -30 of transcription start
CsDof32	TATA-box	1791	1795	core promoter element around -30 of transcription start
CsDof32	TATA-box	1953	1959	core promoter element around -30 of transcription start
CsDof32	TATA-box	1954	1960	core promoter element around -30 of transcription start
CsDof32	TATA-box	1955	1959	core promoter element around -30 of transcription start
CsDof32	P-box	396	403	gibberellin-responsive element
CsDof32	P-box	539	546	gibberellin-responsive element
CsDof32	GT1-motif	1732	1739	light responsive element
CsDof32	GT1-motif	1733	1739	light responsive element
CsDof32	GT1-motif	1812	1818	light responsive element
CsDof32	CCAAT-box	79	85	MYBHv1 binding site
CsDof32	A-box	948	960	sequence conserved in alpha-amylase promoters
CsDof33	TGA-element	1266	1272	auxin-responsive element
CsDof33	TC-rich repeats	1245	1254	cis-acting element involved in defense and stress responsiveness

Name	Cis-element	Start position	Stop position	Function
CsDof33	LTR	523	529	cis-acting element involved in low-temperature responsiveness
CsDof33	ARE	389	395	cis-acting regulatory element essential for the anaerobic induction
CsDof33	ARE	773	779	cis-acting regulatory element essential for the anaerobic induction
CsDof33	ARE	1988	1994	cis-acting regulatory element essential for the anaerobic induction
CsDof33	CGTCA-motif	892	897	cis-acting regulatory element involved in the MeJA-responsiveness
CsDof33	CGTCA-motif	1644	1649	cis-acting regulatory element involved in the MeJA-responsiveness
CsDof33	TGACG-motif	892	897	cis-acting regulatory element involved in the MeJA-responsiveness
CsDof33	TGACG-motif	1644	1649	cis-acting regulatory element involved in the MeJA-responsiveness
CsDof33	O2-site	1867	1875	cis-acting regulatory element involved in zein metabolism regulation
CsDof33	CAAT-box	78	83	common cis-acting element in promoter and enhancer regions
CsDof33	CAAT-box	91	96	common cis-acting element in promoter and enhancer regions
CsDof33	CAAT-box	129	134	common cis-acting element in promoter and enhancer regions
CsDof33	CAAT-box	140	145	common cis-acting element in promoter and enhancer regions
CsDof33	CAAT-box	253	258	common cis-acting element in promoter and enhancer regions
CsDof33	CAAT-box	284	289	common cis-acting element in promoter and enhancer regions
CsDof33	CAAT-box	513	518	common cis-acting element in promoter and enhancer regions
CsDof33	CAAT-box	597	602	common cis-acting element in promoter and enhancer regions
CsDof33	CAAT-box	606	611	common cis-acting element in promoter and enhancer regions
CsDof33	CAAT-box	658	663	common cis-acting element in promoter and enhancer regions
CsDof33	CAAT-box	731	736	common cis-acting element in promoter and enhancer regions
CsDof33	CAAT-box	764	769	common cis-acting element in promoter and enhancer regions
CsDof33	CAAT-box	1012	1017	common cis-acting element in promoter and enhancer regions
CsDof33	CAAT-box	1025	1030	common cis-acting element in promoter and enhancer regions
CsDof33	CAAT-box	1046	1051	common cis-acting element in promoter and enhancer regions
CsDof33	CAAT-box	1515	1520	common cis-acting element in promoter and enhancer regions
CsDof33	CAAT-box	1597	1602	common cis-acting element in promoter and enhancer regions
CsDof33	TATA-box	29	33	core promoter element around -30 of transcription start
CsDof33	TATA-box	52	60	core promoter element around -30 of transcription start
CsDof33	TATA-box	67	73	core promoter element around -30 of transcription start
CsDof33	TATA-box	69	73	core promoter element around -30 of transcription start
CsDof33	TATA-box	222	228	core promoter element around -30 of transcription start
CsDof33	TATA-box	223	227	core promoter element around -30 of transcription start
CsDof33	TATA-box	229	233	core promoter element around -30 of transcription start
CsDof33	TATA-box	267	271	core promoter element around -30 of transcription start
CsDof33	TATA-box	303	310	core promoter element around -30 of transcription start
CsDof33	TATA-box	304	310	core promoter element around -30 of transcription start
CsDof33	TATA-box	305	310	core promoter element around -30 of transcription start
CsDof33	TATA-box	306	310	core promoter element around -30 of transcription start
CsDof33	TATA-box	331	337	core promoter element around -30 of transcription start
CsDof33	TATA-box	332	336	core promoter element around -30 of transcription start
CsDof33	TATA-box	490	494	core promoter element around -30 of transcription start
CsDof33	TATA-box	535	539	core promoter element around -30 of transcription start
CsDof33	TATA-box	615	621	core promoter element around -30 of transcription start
CsDof33	TATA-box	616	621	core promoter element around -30 of transcription start
CsDof33	TATA-box	617	621	core promoter element around -30 of transcription start
CsDof33	TATA-box	677	683	core promoter element around -30 of transcription start
CsDof33	TATA-box	678	683	core promoter element around -30 of transcription start
CsDof33	TATA-box	679	683	core promoter element around -30 of transcription start
CsDof33	TATA-box	779	783	core promoter element around -30 of transcription start
CsDof33	TATA-box	784	790	core promoter element around -30 of transcription start
CsDof33	TATA-box	785	791	core promoter element around -30 of transcription start

Name	Cis-element	Start position	Stop position	Function
CsDof33	TATA-box	787	791	core promoter element around -30 of transcription start
CsDof33	TATA-box	839	843	core promoter element around -30 of transcription start
CsDof33	TATA-box	869	878	core promoter element around -30 of transcription start
CsDof33	TATA-box	870	877	core promoter element around -30 of transcription start
CsDof33	TATA-box	871	877	core promoter element around -30 of transcription start
CsDof33	TATA-box	872	877	core promoter element around -30 of transcription start
CsDof33	TATA-box	873	877	core promoter element around -30 of transcription start
CsDof33	TATA-box	901	907	core promoter element around -30 of transcription start
CsDof33	TATA-box	902	907	core promoter element around -30 of transcription start
CsDof33	TATA-box	903	907	core promoter element around -30 of transcription start
CsDof33	TATA-box	1061	1068	core promoter element around -30 of transcription start
CsDof33	TATA-box	1062	1068	core promoter element around -30 of transcription start
CsDof33	TATA-box	1063	1070	core promoter element around -30 of transcription start
CsDof33	TATA-box	1064	1070	core promoter element around -30 of transcription start
CsDof33	TATA-box	1065	1071	core promoter element around -30 of transcription start
CsDof33	TATA-box	1066	1070	core promoter element around -30 of transcription start
CsDof33	TATA-box	1255	1259	core promoter element around -30 of transcription start
CsDof33	TATA-box	1286	1292	core promoter element around -30 of transcription start
CsDof33	TATA-box	1287	1292	core promoter element around -30 of transcription start
CsDof33	TATA-box	1288	1292	core promoter element around -30 of transcription start
CsDof33	TATA-box	1321	1327	core promoter element around -30 of transcription start
CsDof33	TATA-box	1322	1326	core promoter element around -30 of transcription start
CsDof33	TATA-box	1423	1427	core promoter element around -30 of transcription start
CsDof33	TATA-box	1446	1455	core promoter element around -30 of transcription start
CsDof33	TATA-box	1447	1452	core promoter element around -30 of transcription start
CsDof33	TATA-box	1448	1452	core promoter element around -30 of transcription start
CsDof33	TATA-box	1457	1463	core promoter element around -30 of transcription start
CsDof33	TATA-box	1459	1465	core promoter element around -30 of transcription start
CsDof33	TATA-box	1460	1466	core promoter element around -30 of transcription start
CsDof33	TATA-box	1461	1467	core promoter element around -30 of transcription start
CsDof33	TATA-box	1462	1468	core promoter element around -30 of transcription start
CsDof33	TATA-box	1463	1467	core promoter element around -30 of transcription start
CsDof33	TATA-box	1581	1585	core promoter element around -30 of transcription start
CsDof33	TATA-box	1587	1593	core promoter element around -30 of transcription start
CsDof33	TATA-box	1588	1592	core promoter element around -30 of transcription start
CsDof33	TATA-box	1611	1617	core promoter element around -30 of transcription start
CsDof33	TATA-box	1612	1616	core promoter element around -30 of transcription start
CsDof33	TATA-box	1932	1940	core promoter element around -30 of transcription start
CsDof33	TATA-box	1935	1940	core promoter element around -30 of transcription start
CsDof33	TATA-box	1936	1940	core promoter element around -30 of transcription start
CsDof33	TATA-box	1974	1983	core promoter element around -30 of transcription start
CsDof33	GT1-motif	1719	1725	light responsive element
CsDof33	MBS	576	582	MYB binding site involved in drought-inducibility
CsDof33	Box III	1525	1536	protein binding site
CsDof34	ABRE	443	448	abscisic acid responsiveness
CsDof34	ABRE	543	552	abscisic acid responsiveness
CsDof34	TGA-element	1030	1036	auxin-responsive element
CsDof34	AT-rich element	1230	1240	binding site of AT-rich DNA binding protein (ATBP-1)
CsDof34	TC-rich repeats	1399	1408	cis-acting element involved in defense and stress responsiveness
CsDof34	TC-rich repeats	1612	1621	cis-acting element involved in defense and stress responsiveness
CsDof34	AuxRR-core	998	1005	cis-acting regulatory element involved in auxin responsiveness

Name	Cis-element	Start position	Stop position	Function
CsDof34	G-box	442	448	cis-acting regulatory element involved in light responsiveness
CsDof34	CAAT-box	130	135	common cis-acting element in promoter and enhancer regions
CsDof34	CAAT-box	292	297	common cis-acting element in promoter and enhancer regions
CsDof34	CAAT-box	366	371	common cis-acting element in promoter and enhancer regions
CsDof34	CAAT-box	400	405	common cis-acting element in promoter and enhancer regions
CsDof34	CAAT-box	521	526	common cis-acting element in promoter and enhancer regions
CsDof34	CAAT-box	722	727	common cis-acting element in promoter and enhancer regions
CsDof34	CAAT-box	801	806	common cis-acting element in promoter and enhancer regions
CsDof34	CAAT-box	1098	1103	common cis-acting element in promoter and enhancer regions
CsDof34	CAAT-box	1416	1421	common cis-acting element in promoter and enhancer regions
CsDof34	CAAT-box	1447	1452	common cis-acting element in promoter and enhancer regions
CsDof34	TATA-box	369	375	core promoter element around -30 of transcription start
CsDof34	TATA-box	370	375	core promoter element around -30 of transcription start
CsDof34	TATA-box	371	375	core promoter element around -30 of transcription start
CsDof34	TATA-box	487	491	core promoter element around -30 of transcription start
CsDof34	TATA-box	505	509	core promoter element around -30 of transcription start
CsDof34	TATA-box	555	559	core promoter element around -30 of transcription start
CsDof34	TATA-box	569	575	core promoter element around -30 of transcription start
CsDof34	TATA-box	570	574	core promoter element around -30 of transcription start
CsDof34	TATA-box	685	692	core promoter element around -30 of transcription start
CsDof34	TATA-box	739	746	core promoter element around -30 of transcription start
CsDof34	TATA-box	776	782	core promoter element around -30 of transcription start
CsDof34	TATA-box	777	781	core promoter element around -30 of transcription start
CsDof34	TATA-box	787	791	core promoter element around -30 of transcription start
CsDof34	TATA-box	848	855	core promoter element around -30 of transcription start
CsDof34	TATA-box	1117	1123	core promoter element around -30 of transcription start
CsDof34	TATA-box	1118	1123	core promoter element around -30 of transcription start
CsDof34	TATA-box	1119	1123	core promoter element around -30 of transcription start
CsDof34	TATA-box	1137	1143	core promoter element around -30 of transcription start
CsDof34	TATA-box	1138	1143	core promoter element around -30 of transcription start
CsDof34	TATA-box	1139	1143	core promoter element around -30 of transcription start
CsDof34	TATA-box	1205	1211	core promoter element around -30 of transcription start
CsDof34	TATA-box	1206	1210	core promoter element around -30 of transcription start
CsDof34	TATA-box	1209	1215	core promoter element around -30 of transcription start
CsDof34	TATA-box	1210	1215	core promoter element around -30 of transcription start
CsDof34	TATA-box	1211	1215	core promoter element around -30 of transcription start
CsDof34	TATA-box	1242	1248	core promoter element around -30 of transcription start
CsDof34	TATA-box	1243	1249	core promoter element around -30 of transcription start
CsDof34	TATA-box	1244	1248	core promoter element around -30 of transcription start
CsDof34	TATA-box	1486	1492	core promoter element around -30 of transcription start
CsDof34	TATA-box	1487	1491	core promoter element around -30 of transcription start
CsDof34	TATA-box	1554	1558	core promoter element around -30 of transcription start
CsDof34	TATA-box	1563	1569	core promoter element around -30 of transcription start
CsDof34	TATA-box	1564	1570	core promoter element around -30 of transcription start
CsDof34	TATA-box	1565	1571	core promoter element around -30 of transcription start
CsDof34	TATA-box	1566	1570	core promoter element around -30 of transcription start
CsDof34	TATA-box	1675	1681	core promoter element around -30 of transcription start
CsDof34	TATA-box	1676	1680	core promoter element around -30 of transcription start
CsDof34	TATA-box	1714	1720	core promoter element around -30 of transcription start
CsDof34	TATA-box	1715	1722	core promoter element around -30 of transcription start
CsDof34	TATA-box	1716	1722	core promoter element around -30 of transcription start

Name	Cis-element	Start position	Stop position	Function
CsDof34	TATA-box	1717	1723	core promoter element around -30 of transcription start
CsDof34	TATA-box	1718	1724	core promoter element around -30 of transcription start
CsDof34	TATA-box	1719	1725	core promoter element around -30 of transcription start
CsDof34	TATA-box	1720	1726	core promoter element around -30 of transcription start
CsDof34	TATA-box	1721	1727	core promoter element around -30 of transcription start
CsDof34	TATA-box	1722	1728	core promoter element around -30 of transcription start
CsDof34	TATA-box	1723	1729	core promoter element around -30 of transcription start
CsDof34	TATA-box	1724	1730	core promoter element around -30 of transcription start
CsDof34	TATA-box	1725	1731	core promoter element around -30 of transcription start
CsDof34	TATA-box	1726	1732	core promoter element around -30 of transcription start
CsDof34	TATA-box	1727	1733	core promoter element around -30 of transcription start
CsDof34	TATA-box	1728	1734	core promoter element around -30 of transcription start
CsDof34	TATA-box	1729	1735	core promoter element around -30 of transcription start
CsDof34	TATA-box	1730	1736	core promoter element around -30 of transcription start
CsDof34	TATA-box	1731	1737	core promoter element around -30 of transcription start
CsDof34	TATA-box	1732	1738	core promoter element around -30 of transcription start
CsDof34	TATA-box	1733	1739	core promoter element around -30 of transcription start
CsDof34	TATA-box	1734	1738	core promoter element around -30 of transcription start
CsDof34	TATA-box	1746	1755	core promoter element around -30 of transcription start
CsDof34	TATA-box	1747	1753	core promoter element around -30 of transcription start
CsDof34	TATA-box	1748	1754	core promoter element around -30 of transcription start
CsDof34	TATA-box	1749	1755	core promoter element around -30 of transcription start
CsDof34	TATA-box	1750	1756	core promoter element around -30 of transcription start
CsDof34	TATA-box	1751	1757	core promoter element around -30 of transcription start
CsDof34	TATA-box	1752	1756	core promoter element around -30 of transcription start
CsDof34	GT1-motif	1049	1055	light responsive element
CsDof34	GT1-motif	1343	1349	light responsive element
CsDof34	WUN-motif	1588	1597	wound-responsive element
CsDof35	ABRE	131	137	abscisic acid responsiveness
CsDof35	ABRE	132	137	abscisic acid responsiveness
CsDof35	ABRE	615	620	abscisic acid responsiveness
CsDof35	ABRE	642	648	abscisic acid responsiveness
CsDof35	ABRE	643	648	abscisic acid responsiveness
CsDof35	TGA-element	148	154	auxin-responsive element
CsDof35	TGA-element	198	204	auxin-responsive element
CsDof35	TC-rich repeats	229	238	cis-acting element involved in defense and stress responsiveness
CsDof35	ARE	746	752	cis-acting regulatory element essential for the anaerobic induction
CsDof35	ARE	1460	1466	cis-acting regulatory element essential for the anaerobic induction
CsDof35	G-box	131	137	cis-acting regulatory element involved in light responsiveness
CsDof35	G-box	642	648	cis-acting regulatory element involved in light responsiveness
CsDof35	G-box	713	719	cis-acting regulatory element involved in light responsiveness
CsDof35	G-Box	131	137	cis-acting regulatory element involved in light responsiveness
CsDof35	G-Box	615	621	cis-acting regulatory element involved in light responsiveness
CsDof35	G-Box	642	648	cis-acting regulatory element involved in light responsiveness
CsDof35	CAAT-box	50	55	common cis-acting element in promoter and enhancer regions
CsDof35	CAAT-box	118	123	common cis-acting element in promoter and enhancer regions
CsDof35	CAAT-box	224	229	common cis-acting element in promoter and enhancer regions
CsDof35	CAAT-box	484	489	common cis-acting element in promoter and enhancer regions
CsDof35	CAAT-box	512	517	common cis-acting element in promoter and enhancer regions
CsDof35	CAAT-box	758	763	common cis-acting element in promoter and enhancer regions
CsDof35	CAAT-box	939	944	common cis-acting element in promoter and enhancer regions

Name	Cis-element	Start position	Stop position	Function
CsDof35	CAAT-box	1011	1016	common cis-acting element in promoter and enhancer regions
CsDof35	CAAT-box	1484	1489	common cis-acting element in promoter and enhancer regions
CsDof35	CAAT-box	1515	1520	common cis-acting element in promoter and enhancer regions
CsDof35	CAAT-box	1609	1614	common cis-acting element in promoter and enhancer regions
CsDof35	CAAT-box	1612	1617	common cis-acting element in promoter and enhancer regions
CsDof35	TATA-box	33	39	core promoter element around -30 of transcription start
CsDof35	TATA-box	34	39	core promoter element around -30 of transcription start
CsDof35	TATA-box	35	39	core promoter element around -30 of transcription start
CsDof35	TATA-box	69	75	core promoter element around -30 of transcription start
CsDof35	TATA-box	70	74	core promoter element around -30 of transcription start
CsDof35	TATA-box	382	388	core promoter element around -30 of transcription start
CsDof35	TATA-box	384	388	core promoter element around -30 of transcription start
CsDof35	TATA-box	386	394	core promoter element around -30 of transcription start
CsDof35	TATA-box	465	470	core promoter element around -30 of transcription start
CsDof35	TATA-box	466	470	core promoter element around -30 of transcription start
CsDof35	TATA-box	469	475	core promoter element around -30 of transcription start
CsDof35	TATA-box	470	475	core promoter element around -30 of transcription start
CsDof35	TATA-box	471	475	core promoter element around -30 of transcription start
CsDof35	TATA-box	674	681	core promoter element around -30 of transcription start
CsDof35	TATA-box	675	681	core promoter element around -30 of transcription start
CsDof35	TATA-box	676	681	core promoter element around -30 of transcription start
CsDof35	TATA-box	677	681	core promoter element around -30 of transcription start
CsDof35	TATA-box	692	698	core promoter element around -30 of transcription start
CsDof35	TATA-box	693	699	core promoter element around -30 of transcription start
CsDof35	TATA-box	694	700	core promoter element around -30 of transcription start
CsDof35	TATA-box	695	701	core promoter element around -30 of transcription start
CsDof35	TATA-box	696	702	core promoter element around -30 of transcription start
CsDof35	TATA-box	697	703	core promoter element around -30 of transcription start
CsDof35	TATA-box	698	702	core promoter element around -30 of transcription start
CsDof35	TATA-box	707	713	core promoter element around -30 of transcription start
CsDof35	TATA-box	708	712	core promoter element around -30 of transcription start
CsDof35	TATA-box	750	758	core promoter element around -30 of transcription start
CsDof35	TATA-box	753	758	core promoter element around -30 of transcription start
CsDof35	TATA-box	754	758	core promoter element around -30 of transcription start
CsDof35	TATA-box	896	902	core promoter element around -30 of transcription start
CsDof35	TATA-box	898	902	core promoter element around -30 of transcription start
CsDof35	TATA-box	1056	1062	core promoter element around -30 of transcription start
CsDof35	TATA-box	1057	1061	core promoter element around -30 of transcription start
CsDof35	TATA-box	1066	1075	core promoter element around -30 of transcription start
CsDof35	TATA-box	1067	1073	core promoter element around -30 of transcription start
CsDof35	TATA-box	1068	1075	core promoter element around -30 of transcription start
CsDof35	TATA-box	1069	1075	core promoter element around -30 of transcription start
CsDof35	TATA-box	1070	1076	core promoter element around -30 of transcription start
CsDof35	TATA-box	1071	1075	core promoter element around -30 of transcription start
CsDof35	TATA-box	1206	1210	core promoter element around -30 of transcription start
CsDof35	TATA-box	1257	1263	core promoter element around -30 of transcription start
CsDof35	TATA-box	1258	1264	core promoter element around -30 of transcription start
CsDof35	TATA-box	1259	1265	core promoter element around -30 of transcription start
CsDof35	TATA-box	1260	1264	core promoter element around -30 of transcription start
CsDof35	TATA-box	1284	1290	core promoter element around -30 of transcription start
CsDof35	TATA-box	1285	1289	core promoter element around -30 of transcription start

Name	Cis-element	Start position	Stop position	Function
CsDof35	TATA-box	1297	1304	core promoter element around -30 of transcription start
CsDof35	TATA-box	1321	1325	core promoter element around -30 of transcription start
CsDof35	TATA-box	1346	1353	core promoter element around -30 of transcription start
CsDof35	TATA-box	1395	1399	core promoter element around -30 of transcription start
CsDof35	TATA-box	1405	1411	core promoter element around -30 of transcription start
CsDof35	TATA-box	1407	1411	core promoter element around -30 of transcription start
CsDof35	TATA-box	1420	1428	core promoter element around -30 of transcription start
CsDof35	TATA-box	1544	1548	core promoter element around -30 of transcription start
CsDof35	TATA-box	1566	1570	core promoter element around -30 of transcription start
CsDof35	TATA-box	1592	1598	core promoter element around -30 of transcription start
CsDof35	TATA-box	1593	1600	core promoter element around -30 of transcription start
CsDof35	TATA-box	1594	1600	core promoter element around -30 of transcription start
CsDof35	TATA-box	1595	1601	core promoter element around -30 of transcription start
CsDof35	TATA-box	1596	1600	core promoter element around -30 of transcription start
CsDof35	TATA-box	1657	1666	core promoter element around -30 of transcription start
CsDof35	TATA-box	1667	1671	core promoter element around -30 of transcription start
CsDof35	TATA-box	1672	1676	core promoter element around -30 of transcription start
CsDof35	TATA-box	1778	1784	core promoter element around -30 of transcription start
CsDof35	TATA-box	1779	1784	core promoter element around -30 of transcription start
CsDof35	TATA-box	1780	1784	core promoter element around -30 of transcription start
CsDof35	P-box	1750	1757	gibberellin-responsive element
CsDof35	Sp1	1309	1315	light responsive element
CsDof35	GT1-motif	42	48	light responsive element
CsDof35	MBS	240	246	MYB binding site involved in drought-inducibility
CsDof35	MRE	1180	1187	MYB binding site involved in light responsiveness
CsDof35	CCAAT-box	96	102	MYBHv1 binding site
CsDof35	CCAAT-box	555	561	MYBHv1 binding site
CsDof35	HD-Zip 3	1553	1563	protein binding site
CsDof36	ABRE	1473	1478	abscisic acid responsiveness
CsDof36	ABRE	1582	1587	abscisic acid responsiveness
CsDof36	ABRE	1746	1751	abscisic acid responsiveness
CsDof36	MSA-like	600	609	cis-acting element involved in cell cycle regulation
CsDof36	ARE	282	288	cis-acting regulatory element essential for the anaerobic induction
CsDof36	ARE	1631	1637	cis-acting regulatory element essential for the anaerobic induction
CsDof36	G-Box	1473	1479	cis-acting regulatory element involved in light responsiveness
CsDof36	G-Box	1581	1587	cis-acting regulatory element involved in light responsiveness
CsDof36	G-box	1745	1751	cis-acting regulatory element involved in light responsiveness
CsDof36	TGACG-motif	473	478	cis-acting regulatory element involved in the MeJA-responsiveness
CsDof36	CGTCA-motif	473	478	cis-acting regulatory element involved in the MeJA-responsiveness
CsDof36	CAAT-box	90	95	common cis-acting element in promoter and enhancer regions
CsDof36	CAAT-box	174	179	common cis-acting element in promoter and enhancer regions
CsDof36	CAAT-box	686	691	common cis-acting element in promoter and enhancer regions
CsDof36	CAAT-box	753	758	common cis-acting element in promoter and enhancer regions
CsDof36	CAAT-box	854	859	common cis-acting element in promoter and enhancer regions
CsDof36	CAAT-box	1211	1216	common cis-acting element in promoter and enhancer regions
CsDof36	CAAT-box	1398	1403	common cis-acting element in promoter and enhancer regions
CsDof36	CAAT-box	1443	1448	common cis-acting element in promoter and enhancer regions
CsDof36	CAAT-box	1658	1663	common cis-acting element in promoter and enhancer regions
CsDof36	CAAT-box	1686	1691	common cis-acting element in promoter and enhancer regions
CsDof36	CAAT-box	1711	1716	common cis-acting element in promoter and enhancer regions
CsDof36	TATA-box	110	116	core promoter element around -30 of transcription start

Name	Cis-element	Start position	Stop position	Function
CsDof36	TATA-box	111	117	core promoter element around -30 of transcription start
CsDof36	TATA-box	112	118	core promoter element around -30 of transcription start
CsDof36	TATA-box	113	119	core promoter element around -30 of transcription start
CsDof36	TATA-box	114	118	core promoter element around -30 of transcription start
CsDof36	TATA-box	212	217	core promoter element around -30 of transcription start
CsDof36	TATA-box	213	217	core promoter element around -30 of transcription start
CsDof36	TATA-box	269	275	core promoter element around -30 of transcription start
CsDof36	TATA-box	271	275	core promoter element around -30 of transcription start
CsDof36	TATA-box	325	329	core promoter element around -30 of transcription start
CsDof36	TATA-box	560	567	core promoter element around -30 of transcription start
CsDof36	TATA-box	615	621	core promoter element around -30 of transcription start
CsDof36	TATA-box	616	621	core promoter element around -30 of transcription start
CsDof36	TATA-box	617	621	core promoter element around -30 of transcription start
CsDof36	TATA-box	640	646	core promoter element around -30 of transcription start
CsDof36	TATA-box	642	646	core promoter element around -30 of transcription start
CsDof36	TATA-box	649	653	core promoter element around -30 of transcription start
CsDof36	TATA-box	778	782	core promoter element around -30 of transcription start
CsDof36	TATA-box	857	863	core promoter element around -30 of transcription start
CsDof36	TATA-box	858	862	core promoter element around -30 of transcription start
CsDof36	TATA-box	873	877	core promoter element around -30 of transcription start
CsDof36	TATA-box	917	923	core promoter element around -30 of transcription start
CsDof36	TATA-box	918	922	core promoter element around -30 of transcription start
CsDof36	TATA-box	954	960	core promoter element around -30 of transcription start
CsDof36	TATA-box	955	961	core promoter element around -30 of transcription start
CsDof36	TATA-box	956	962	core promoter element around -30 of transcription start
CsDof36	TATA-box	957	961	core promoter element around -30 of transcription start
CsDof36	TATA-box	1032	1036	core promoter element around -30 of transcription start
CsDof36	TATA-box	1077	1083	core promoter element around -30 of transcription start
CsDof36	TATA-box	1078	1084	core promoter element around -30 of transcription start
CsDof36	TATA-box	1079	1085	core promoter element around -30 of transcription start
CsDof36	TATA-box	1080	1084	core promoter element around -30 of transcription start
CsDof36	TATA-box	1265	1271	core promoter element around -30 of transcription start
CsDof36	TATA-box	1266	1271	core promoter element around -30 of transcription start
CsDof36	TATA-box	1267	1271	core promoter element around -30 of transcription start
CsDof36	TATA-box	1291	1297	core promoter element around -30 of transcription start
CsDof36	TATA-box	1292	1296	core promoter element around -30 of transcription start
CsDof36	TATA-box	1306	1312	core promoter element around -30 of transcription start
CsDof36	TATA-box	1307	1311	core promoter element around -30 of transcription start
CsDof36	TATA-box	1337	1341	core promoter element around -30 of transcription start
CsDof36	TATA-box	1429	1433	core promoter element around -30 of transcription start
CsDof36	TATA-box	1549	1558	core promoter element around -30 of transcription start
CsDof36	TATA-box	1607	1611	core promoter element around -30 of transcription start
CsDof36	TATA-box	1810	1814	core promoter element around -30 of transcription start
CsDof36	TATA-box	1909	1914	core promoter element around -30 of transcription start
CsDof36	TATA-box	1910	1914	core promoter element around -30 of transcription start
CsDof36	TATA-box	1924	1933	core promoter element around -30 of transcription start
CsDof36	TATA-box	1926	1930	core promoter element around -30 of transcription start
CsDof36	P-box	1273	1280	gibberellin-responsive element
CsDof36	MBS	573	579	MYB binding site involved in drought-inducibility
CsDof36	MRE	1136	1143	MYB binding site involved in light responsiveness
CsDof36	CCAAT-box	602	608	MYBHv1 binding site

Name	Cis-element	Start position	Stop position	Function
CsDof37	TGA-element	593	599	auxin-responsive element
CsDof37	TC-rich repeats	1792	1801	cis-acting element involved in defense and stress responsiveness
CsDof37	TCA-element	96	105	cis-acting element involved in salicylic acid responsiveness
CsDof37	TCA-element	1277	1286	cis-acting element involved in salicylic acid responsiveness
CsDof37	ARE	407	413	cis-acting regulatory element essential for the anaerobic induction
CsDof37	ARE	1359	1365	cis-acting regulatory element essential for the anaerobic induction
CsDof37	ARE	1844	1850	cis-acting regulatory element essential for the anaerobic induction
CsDof37	circadian	635	644	cis-acting regulatory element involved in circadian control
CsDof37	TGACG-motif	270	275	cis-acting regulatory element involved in the MeJA-responsiveness
CsDof37	CGTCA-motif	270	275	cis-acting regulatory element involved in the MeJA-responsiveness
CsDof37	CAAT-box	6	11	common cis-acting element in promoter and enhancer regions
CsDof37	CAAT-box	280	285	common cis-acting element in promoter and enhancer regions
CsDof37	CAAT-box	351	356	common cis-acting element in promoter and enhancer regions
CsDof37	CAAT-box	384	389	common cis-acting element in promoter and enhancer regions
CsDof37	CAAT-box	437	442	common cis-acting element in promoter and enhancer regions
CsDof37	CAAT-box	788	793	common cis-acting element in promoter and enhancer regions
CsDof37	CAAT-box	903	908	common cis-acting element in promoter and enhancer regions
CsDof37	CAAT-box	923	928	common cis-acting element in promoter and enhancer regions
CsDof37	CAAT-box	1098	1103	common cis-acting element in promoter and enhancer regions
CsDof37	CAAT-box	1459	1464	common cis-acting element in promoter and enhancer regions
CsDof37	CAAT-box	1685	1690	common cis-acting element in promoter and enhancer regions
CsDof37	TATA-box	41	47	core promoter element around -30 of transcription start
CsDof37	TATA-box	42	47	core promoter element around -30 of transcription start
CsDof37	TATA-box	43	47	core promoter element around -30 of transcription start
CsDof37	TATA-box	198	204	core promoter element around -30 of transcription start
CsDof37	TATA-box	199	206	core promoter element around -30 of transcription start
CsDof37	TATA-box	200	206	core promoter element around -30 of transcription start
CsDof37	TATA-box	201	207	core promoter element around -30 of transcription start
CsDof37	TATA-box	202	206	core promoter element around -30 of transcription start
CsDof37	TATA-box	290	297	core promoter element around -30 of transcription start
CsDof37	TATA-box	442	449	core promoter element around -30 of transcription start
CsDof37	TATA-box	443	449	core promoter element around -30 of transcription start
CsDof37	TATA-box	445	449	core promoter element around -30 of transcription start
CsDof37	TATA-box	450	455	core promoter element around -30 of transcription start
CsDof37	TATA-box	451	455	core promoter element around -30 of transcription start
CsDof37	TATA-box	472	478	core promoter element around -30 of transcription start
CsDof37	TATA-box	474	478	core promoter element around -30 of transcription start
CsDof37	TATA-box	501	507	core promoter element around -30 of transcription start
CsDof37	TATA-box	502	506	core promoter element around -30 of transcription start
CsDof37	TATA-box	672	676	core promoter element around -30 of transcription start
CsDof37	TATA-box	729	735	core promoter element around -30 of transcription start
CsDof37	TATA-box	730	735	core promoter element around -30 of transcription start
CsDof37	TATA-box	731	735	core promoter element around -30 of transcription start
CsDof37	TATA-box	751	757	core promoter element around -30 of transcription start
CsDof37	TATA-box	752	757	core promoter element around -30 of transcription start
CsDof37	TATA-box	753	757	core promoter element around -30 of transcription start
CsDof37	TATA-box	939	943	core promoter element around -30 of transcription start
CsDof37	TATA-box	946	951	core promoter element around -30 of transcription start
CsDof37	TATA-box	947	951	core promoter element around -30 of transcription start
CsDof37	TATA-box	1010	1015	core promoter element around -30 of transcription start
CsDof37	TATA-box	1011	1015	core promoter element around -30 of transcription start

Name	Cis-element	Start position	Stop position	Function
CsDof37	TATA-box	1019	1028	core promoter element around -30 of transcription start
CsDof37	TATA-box	1020	1025	core promoter element around -30 of transcription start
CsDof37	TATA-box	1021	1025	core promoter element around -30 of transcription start
CsDof37	TATA-box	1064	1070	core promoter element around -30 of transcription start
CsDof37	TATA-box	1066	1070	core promoter element around -30 of transcription start
CsDof37	TATA-box	1071	1078	core promoter element around -30 of transcription start
CsDof37	TATA-box	1101	1107	core promoter element around -30 of transcription start
CsDof37	TATA-box	1102	1108	core promoter element around -30 of transcription start
CsDof37	TATA-box	1103	1109	core promoter element around -30 of transcription start
CsDof37	TATA-box	1104	1108	core promoter element around -30 of transcription start
CsDof37	TATA-box	1140	1148	core promoter element around -30 of transcription start
CsDof37	TATA-box	1146	1150	core promoter element around -30 of transcription start
CsDof37	TATA-box	1219	1227	core promoter element around -30 of transcription start
CsDof37	TATA-box	1236	1240	core promoter element around -30 of transcription start
CsDof37	TATA-box	1253	1259	core promoter element around -30 of transcription start
CsDof37	TATA-box	1254	1258	core promoter element around -30 of transcription start
CsDof37	TATA-box	1437	1443	core promoter element around -30 of transcription start
CsDof37	TATA-box	1438	1443	core promoter element around -30 of transcription start
CsDof37	TATA-box	1439	1443	core promoter element around -30 of transcription start
CsDof37	TATA-box	1441	1450	core promoter element around -30 of transcription start
CsDof37	TATA-box	1539	1543	core promoter element around -30 of transcription start
CsDof37	TATA-box	1541	1549	core promoter element around -30 of transcription start
CsDof37	TATA-box	1549	1555	core promoter element around -30 of transcription start
CsDof37	TATA-box	1550	1556	core promoter element around -30 of transcription start
CsDof37	TATA-box	1552	1556	core promoter element around -30 of transcription start
CsDof37	TATA-box	1561	1567	core promoter element around -30 of transcription start
CsDof37	TATA-box	1562	1567	core promoter element around -30 of transcription start
CsDof37	TATA-box	1563	1567	core promoter element around -30 of transcription start
CsDof37	TATA-box	1568	1574	core promoter element around -30 of transcription start
CsDof37	TATA-box	1569	1573	core promoter element around -30 of transcription start
CsDof37	TATA-box	1590	1595	core promoter element around -30 of transcription start
CsDof37	TATA-box	1591	1595	core promoter element around -30 of transcription start
CsDof37	TATA-box	1652	1658	core promoter element around -30 of transcription start
CsDof37	TATA-box	1653	1659	core promoter element around -30 of transcription start
CsDof37	TATA-box	1654	1660	core promoter element around -30 of transcription start
CsDof37	TATA-box	1655	1661	core promoter element around -30 of transcription start
CsDof37	TATA-box	1656	1662	core promoter element around -30 of transcription start
CsDof37	TATA-box	1657	1663	core promoter element around -30 of transcription start
CsDof37	TATA-box	1659	1663	core promoter element around -30 of transcription start
CsDof37	TATA-box	1667	1673	core promoter element around -30 of transcription start
CsDof37	TATA-box	1668	1674	core promoter element around -30 of transcription start
CsDof37	TATA-box	1669	1675	core promoter element around -30 of transcription start
CsDof37	TATA-box	1670	1676	core promoter element around -30 of transcription start
CsDof37	TATA-box	1671	1677	core promoter element around -30 of transcription start
CsDof37	TATA-box	1673	1677	core promoter element around -30 of transcription start
CsDof37	TATA-box	1712	1718	core promoter element around -30 of transcription start
CsDof37	TATA-box	1713	1719	core promoter element around -30 of transcription start
CsDof37	TATA-box	1714	1720	core promoter element around -30 of transcription start
CsDof37	TATA-box	1715	1721	core promoter element around -30 of transcription start
CsDof37	TATA-box	1716	1722	core promoter element around -30 of transcription start
CsDof37	TATA-box	1717	1721	core promoter element around -30 of transcription start

Name	Cis-element	Start position	Stop position	Function
CsDof37	AT-rich sequence	1429	1438	element for maximal elicitor-mediated activation (2copies)
CsDof37	P-box	1839	1846	gibberellin-responsive element
CsDof37	GT1-motif	712	718	light responsive element
CsDof37	GT1-motif	829	836	light responsive element
CsDof37	GT1-motif	830	836	light responsive element
CsDof38	ARE	1937	1943	cis-acting regulatory element essential for the anaerobic induction
CsDof38	CAAT-box	1587	1592	common cis-acting element in promoter and enhancer regions
CsDof38	CAAT-box	1748	1753	common cis-acting element in promoter and enhancer regions
CsDof38	CAAT-box	1921	1926	common cis-acting element in promoter and enhancer regions
CsDof38	TATA-box	1283	1287	core promoter element around -30 of transcription start
CsDof38	TATA-box	1299	1305	core promoter element around -30 of transcription start
CsDof38	TATA-box	1301	1305	core promoter element around -30 of transcription start
CsDof38	TATA-box	1308	1314	core promoter element around -30 of transcription start
CsDof38	TATA-box	1309	1314	core promoter element around -30 of transcription start
CsDof38	TATA-box	1310	1314	core promoter element around -30 of transcription start
CsDof38	TATA-box	1324	1330	core promoter element around -30 of transcription start
CsDof38	TATA-box	1325	1330	core promoter element around -30 of transcription start
CsDof38	TATA-box	1326	1330	core promoter element around -30 of transcription start
CsDof38	TATA-box	1733	1741	core promoter element around -30 of transcription start
CsDof38	TATA-box	1738	1744	core promoter element around -30 of transcription start
CsDof38	TATA-box	1739	1743	core promoter element around -30 of transcription start
CsDof38	TATA-box	1822	1828	core promoter element around -30 of transcription start
CsDof38	TATA-box	1823	1829	core promoter element around -30 of transcription start
CsDof38	TATA-box	1824	1830	core promoter element around -30 of transcription start
CsDof38	TATA-box	1825	1829	core promoter element around -30 of transcription start
CsDof38	TATA-box	1839	1846	core promoter element around -30 of transcription start
CsDof38	TATA-box	1975	1984	core promoter element around -30 of transcription start
CsDof38	TATA-box	1976	1985	core promoter element around -30 of transcription start
CsDof38	TATA-box	1977	1983	core promoter element around -30 of transcription start
CsDof38	TATA-box	1978	1985	core promoter element around -30 of transcription start
CsDof38	TATA-box	1979	1985	core promoter element around -30 of transcription start
CsDof38	TATA-box	1980	1986	core promoter element around -30 of transcription start
CsDof38	TATA-box	1981	1985	core promoter element around -30 of transcription start
CsDof38	GT1-motif	1650	1656	light responsive element
CsDof39	ABRE	692	701	abscisic acid responsiveness
CsDof39	ABRE	694	700	abscisic acid responsiveness
CsDof39	ABRE	695	700	abscisic acid responsiveness
CsDof39	ABRE	891	900	abscisic acid responsiveness
CsDof39	ABRE	893	899	abscisic acid responsiveness
CsDof39	ABRE	894	899	abscisic acid responsiveness
CsDof39	ABRE	982	988	abscisic acid responsiveness
CsDof39	ABRE	983	988	abscisic acid responsiveness
CsDof39	ABRE	1003	1008	abscisic acid responsiveness
CsDof39	ABRE	1052	1058	abscisic acid responsiveness
CsDof39	ABRE	1053	1058	abscisic acid responsiveness
CsDof39	TATC-box	643	650	cis-acting element involved in gibberellin-responsiveness
CsDof39	LTR	1026	1032	cis-acting element involved in low-temperature responsiveness
CsDof39	ARE	12	18	cis-acting regulatory element essential for the anaerobic induction
CsDof39	ARE	62	68	cis-acting regulatory element essential for the anaerobic induction
CsDof39	ARE	1598	1604	cis-acting regulatory element essential for the anaerobic induction
CsDof39	AuxRR-core	969	976	cis-acting regulatory element involved in auxin responsiveness

Name	Cis-element	Start position	Stop position	Function
CsDof39	G-box	88	94	cis-acting regulatory element involved in light responsiveness
CsDof39	G-box	691	703	cis-acting regulatory element involved in light responsiveness
CsDof39	G-box	692	701	cis-acting regulatory element involved in light responsiveness
CsDof39	G-box	694	700	cis-acting regulatory element involved in light responsiveness
CsDof39	G-box	891	900	cis-acting regulatory element involved in light responsiveness
CsDof39	G-box	893	899	cis-acting regulatory element involved in light responsiveness
CsDof39	G-box	982	988	cis-acting regulatory element involved in light responsiveness
CsDof39	G-box	1002	1008	cis-acting regulatory element involved in light responsiveness
CsDof39	G-box	1050	1059	cis-acting regulatory element involved in light responsiveness
CsDof39	G-box	1052	1058	cis-acting regulatory element involved in light responsiveness
CsDof39	G-Box	694	700	cis-acting regulatory element involved in light responsiveness
CsDof39	G-Box	893	899	cis-acting regulatory element involved in light responsiveness
CsDof39	G-Box	982	988	cis-acting regulatory element involved in light responsiveness
CsDof39	G-Box	1052	1058	cis-acting regulatory element involved in light responsiveness
CsDof39	TGACG-motif	364	369	cis-acting regulatory element involved in the MeJA-responsiveness
CsDof39	TGACG-motif	375	380	cis-acting regulatory element involved in the MeJA-responsiveness
CsDof39	TGACG-motif	923	928	cis-acting regulatory element involved in the MeJA-responsiveness
CsDof39	TGACG-motif	1001	1006	cis-acting regulatory element involved in the MeJA-responsiveness
CsDof39	TGACG-motif	1371	1376	cis-acting regulatory element involved in the MeJA-responsiveness
CsDof39	TGACG-motif	1882	1887	cis-acting regulatory element involved in the MeJA-responsiveness
CsDof39	CGTCA-motif	364	369	cis-acting regulatory element involved in the MeJA-responsiveness
CsDof39	CGTCA-motif	375	380	cis-acting regulatory element involved in the MeJA-responsiveness
CsDof39	CGTCA-motif	923	928	cis-acting regulatory element involved in the MeJA-responsiveness
CsDof39	CGTCA-motif	1001	1006	cis-acting regulatory element involved in the MeJA-responsiveness
CsDof39	CGTCA-motif	1371	1376	cis-acting regulatory element involved in the MeJA-responsiveness
CsDof39	CGTCA-motif	1882	1887	cis-acting regulatory element involved in the MeJA-responsiveness
CsDof39	CAT-box	1703	1709	cis-acting regulatory element related to meristem expression
CsDof39	CAAT-box	4	9	common cis-acting element in promoter and enhancer regions
CsDof39	CAAT-box	9	14	common cis-acting element in promoter and enhancer regions
CsDof39	CAAT-box	195	200	common cis-acting element in promoter and enhancer regions
CsDof39	CAAT-box	583	588	common cis-acting element in promoter and enhancer regions
CsDof39	CAAT-box	825	830	common cis-acting element in promoter and enhancer regions
CsDof39	CAAT-box	909	914	common cis-acting element in promoter and enhancer regions
CsDof39	CAAT-box	974	979	common cis-acting element in promoter and enhancer regions
CsDof39	CAAT-box	1403	1408	common cis-acting element in promoter and enhancer regions
CsDof39	CAAT-box	1443	1448	common cis-acting element in promoter and enhancer regions
CsDof39	TATA-box	207	213	core promoter element around -30 of transcription start
CsDof39	TATA-box	208	214	core promoter element around -30 of transcription start
CsDof39	TATA-box	209	213	core promoter element around -30 of transcription start
CsDof39	TATA-box	285	297	core promoter element around -30 of transcription start
CsDof39	TATA-box	287	293	core promoter element around -30 of transcription start
CsDof39	TATA-box	288	294	core promoter element around -30 of transcription start
CsDof39	TATA-box	289	295	core promoter element around -30 of transcription start
CsDof39	TATA-box	290	296	core promoter element around -30 of transcription start
CsDof39	TATA-box	291	297	core promoter element around -30 of transcription start
CsDof39	TATA-box	292	298	core promoter element around -30 of transcription start
CsDof39	TATA-box	293	299	core promoter element around -30 of transcription start
CsDof39	TATA-box	294	300	core promoter element around -30 of transcription start
CsDof39	TATA-box	295	301	core promoter element around -30 of transcription start
CsDof39	TATA-box	296	302	core promoter element around -30 of transcription start
CsDof39	TATA-box	297	303	core promoter element around -30 of transcription start

Name	Cis-element	Start position	Stop position	Function
CsDof39	TATA-box	298	304	core promoter element around -30 of transcription start
CsDof39	TATA-box	299	305	core promoter element around -30 of transcription start
CsDof39	TATA-box	300	306	core promoter element around -30 of transcription start
CsDof39	TATA-box	301	307	core promoter element around -30 of transcription start
CsDof39	TATA-box	302	306	core promoter element around -30 of transcription start
CsDof39	TATA-box	316	321	core promoter element around -30 of transcription start
CsDof39	TATA-box	317	321	core promoter element around -30 of transcription start
CsDof39	TATA-box	339	343	core promoter element around -30 of transcription start
CsDof39	TATA-box	384	391	core promoter element around -30 of transcription start
CsDof39	TATA-box	385	391	core promoter element around -30 of transcription start
CsDof39	TATA-box	386	391	core promoter element around -30 of transcription start
CsDof39	TATA-box	387	391	core promoter element around -30 of transcription start
CsDof39	TATA-box	429	433	core promoter element around -30 of transcription start
CsDof39	TATA-box	533	537	core promoter element around -30 of transcription start
CsDof39	TATA-box	560	566	core promoter element around -30 of transcription start
CsDof39	TATA-box	561	566	core promoter element around -30 of transcription start
CsDof39	TATA-box	562	566	core promoter element around -30 of transcription start
CsDof39	TATA-box	589	594	core promoter element around -30 of transcription start
CsDof39	TATA-box	590	594	core promoter element around -30 of transcription start
CsDof39	TATA-box	846	852	core promoter element around -30 of transcription start
CsDof39	TATA-box	847	853	core promoter element around -30 of transcription start
CsDof39	TATA-box	848	852	core promoter element around -30 of transcription start
CsDof39	TATA-box	871	877	core promoter element around -30 of transcription start
CsDof39	TATA-box	872	876	core promoter element around -30 of transcription start
CsDof39	TATA-box	1013	1021	core promoter element around -30 of transcription start
CsDof39	TATA-box	1306	1312	core promoter element around -30 of transcription start
CsDof39	TATA-box	1307	1312	core promoter element around -30 of transcription start
CsDof39	TATA-box	1308	1312	core promoter element around -30 of transcription start
CsDof39	TATA-box	1573	1581	core promoter element around -30 of transcription start
CsDof39	TATA-box	1576	1581	core promoter element around -30 of transcription start
CsDof39	TATA-box	1577	1581	core promoter element around -30 of transcription start
CsDof39	TATA-box	1589	1596	core promoter element around -30 of transcription start
CsDof39	TATA-box	1966	1970	core promoter element around -30 of transcription start
CsDof39	GT1-motif	254	261	light responsive element
CsDof39	3-AF1 binding site	1633	1643	light responsive element
CsDof39	CCAAT-box	1296	1302	MYBHv1 binding site
CsDof40	ABRE	692	701	abscisic acid responsiveness
CsDof40	ABRE	694	700	abscisic acid responsiveness
CsDof40	ABRE	695	700	abscisic acid responsiveness
CsDof40	ABRE	891	900	abscisic acid responsiveness
CsDof40	ABRE	893	899	abscisic acid responsiveness
CsDof40	ABRE	894	899	abscisic acid responsiveness
CsDof40	ABRE	982	988	abscisic acid responsiveness
CsDof40	ABRE	983	988	abscisic acid responsiveness
CsDof40	ABRE	1003	1008	abscisic acid responsiveness
CsDof40	ABRE	1052	1058	abscisic acid responsiveness
CsDof40	ABRE	1053	1058	abscisic acid responsiveness
CsDof40	TATC-box	643	650	cis-acting element involved in gibberellin-responsiveness
CsDof40	LTR	1026	1032	cis-acting element involved in low-temperature responsiveness
CsDof40	ARE	12	18	cis-acting regulatory element essential for the anaerobic induction
CsDof40	ARE	62	68	cis-acting regulatory element essential for the anaerobic induction

Name	Cis-element	Start position	Stop position	Function
CsDof40	ARE	1598	1604	cis-acting regulatory element essential for the anaerobic induction
CsDof40	AuxRR-core	969	976	cis-acting regulatory element involved in auxin responsiveness
CsDof40	G-box	88	94	cis-acting regulatory element involved in light responsiveness
CsDof40	G-box	691	703	cis-acting regulatory element involved in light responsiveness
CsDof40	G-box	692	701	cis-acting regulatory element involved in light responsiveness
CsDof40	G-box	694	700	cis-acting regulatory element involved in light responsiveness
CsDof40	G-box	891	900	cis-acting regulatory element involved in light responsiveness
CsDof40	G-box	893	899	cis-acting regulatory element involved in light responsiveness
CsDof40	G-box	982	988	cis-acting regulatory element involved in light responsiveness
CsDof40	G-box	1002	1008	cis-acting regulatory element involved in light responsiveness
CsDof40	G-box	1050	1059	cis-acting regulatory element involved in light responsiveness
CsDof40	G-box	1052	1058	cis-acting regulatory element involved in light responsiveness
CsDof40	G-Box	694	700	cis-acting regulatory element involved in light responsiveness
CsDof40	G-Box	893	899	cis-acting regulatory element involved in light responsiveness
CsDof40	G-Box	982	988	cis-acting regulatory element involved in light responsiveness
CsDof40	G-Box	1052	1058	cis-acting regulatory element involved in light responsiveness
CsDof40	CGTCA-motif	364	369	cis-acting regulatory element involved in the MeJA-responsiveness
CsDof40	CGTCA-motif	375	380	cis-acting regulatory element involved in the MeJA-responsiveness
CsDof40	CGTCA-motif	923	928	cis-acting regulatory element involved in the MeJA-responsiveness
CsDof40	CGTCA-motif	1001	1006	cis-acting regulatory element involved in the MeJA-responsiveness
CsDof40	CGTCA-motif	1371	1376	cis-acting regulatory element involved in the MeJA-responsiveness
CsDof40	CGTCA-motif	1882	1887	cis-acting regulatory element involved in the MeJA-responsiveness
CsDof40	TGACG-motif	364	369	cis-acting regulatory element involved in the MeJA-responsiveness
CsDof40	TGACG-motif	375	380	cis-acting regulatory element involved in the MeJA-responsiveness
CsDof40	TGACG-motif	923	928	cis-acting regulatory element involved in the MeJA-responsiveness
CsDof40	TGACG-motif	1001	1006	cis-acting regulatory element involved in the MeJA-responsiveness
CsDof40	TGACG-motif	1371	1376	cis-acting regulatory element involved in the MeJA-responsiveness
CsDof40	TGACG-motif	1882	1887	cis-acting regulatory element involved in the MeJA-responsiveness
CsDof40	CAT-box	1703	1709	cis-acting regulatory element related to meristem expression
CsDof40	CAAT-box	4	9	common cis-acting element in promoter and enhancer regions
CsDof40	CAAT-box	9	14	common cis-acting element in promoter and enhancer regions
CsDof40	CAAT-box	195	200	common cis-acting element in promoter and enhancer regions
CsDof40	CAAT-box	583	588	common cis-acting element in promoter and enhancer regions
CsDof40	CAAT-box	825	830	common cis-acting element in promoter and enhancer regions
CsDof40	CAAT-box	909	914	common cis-acting element in promoter and enhancer regions
CsDof40	CAAT-box	974	979	common cis-acting element in promoter and enhancer regions
CsDof40	CAAT-box	1403	1408	common cis-acting element in promoter and enhancer regions
CsDof40	CAAT-box	1443	1448	common cis-acting element in promoter and enhancer regions
CsDof40	TATA-box	207	213	core promoter element around -30 of transcription start
CsDof40	TATA-box	208	214	core promoter element around -30 of transcription start
CsDof40	TATA-box	209	213	core promoter element around -30 of transcription start
CsDof40	TATA-box	285	297	core promoter element around -30 of transcription start
CsDof40	TATA-box	287	293	core promoter element around -30 of transcription start
CsDof40	TATA-box	288	294	core promoter element around -30 of transcription start
CsDof40	TATA-box	289	295	core promoter element around -30 of transcription start
CsDof40	TATA-box	290	296	core promoter element around -30 of transcription start
CsDof40	TATA-box	291	297	core promoter element around -30 of transcription start
CsDof40	TATA-box	292	298	core promoter element around -30 of transcription start
CsDof40	TATA-box	293	299	core promoter element around -30 of transcription start
CsDof40	TATA-box	294	300	core promoter element around -30 of transcription start
CsDof40	TATA-box	295	301	core promoter element around -30 of transcription start

Name	Cis-element	Start position	Stop position	Function
CsDof40	TATA-box	296	302	core promoter element around -30 of transcription start
CsDof40	TATA-box	297	303	core promoter element around -30 of transcription start
CsDof40	TATA-box	298	304	core promoter element around -30 of transcription start
CsDof40	TATA-box	299	305	core promoter element around -30 of transcription start
CsDof40	TATA-box	300	306	core promoter element around -30 of transcription start
CsDof40	TATA-box	301	307	core promoter element around -30 of transcription start
CsDof40	TATA-box	302	306	core promoter element around -30 of transcription start
CsDof40	TATA-box	316	321	core promoter element around -30 of transcription start
CsDof40	TATA-box	317	321	core promoter element around -30 of transcription start
CsDof40	TATA-box	339	343	core promoter element around -30 of transcription start
CsDof40	TATA-box	384	391	core promoter element around -30 of transcription start
CsDof40	TATA-box	385	391	core promoter element around -30 of transcription start
CsDof40	TATA-box	386	391	core promoter element around -30 of transcription start
CsDof40	TATA-box	387	391	core promoter element around -30 of transcription start
CsDof40	TATA-box	429	433	core promoter element around -30 of transcription start
CsDof40	TATA-box	533	537	core promoter element around -30 of transcription start
CsDof40	TATA-box	560	566	core promoter element around -30 of transcription start
CsDof40	TATA-box	561	566	core promoter element around -30 of transcription start
CsDof40	TATA-box	562	566	core promoter element around -30 of transcription start
CsDof40	TATA-box	589	594	core promoter element around -30 of transcription start
CsDof40	TATA-box	590	594	core promoter element around -30 of transcription start
CsDof40	TATA-box	846	852	core promoter element around -30 of transcription start
CsDof40	TATA-box	847	853	core promoter element around -30 of transcription start
CsDof40	TATA-box	848	852	core promoter element around -30 of transcription start
CsDof40	TATA-box	871	877	core promoter element around -30 of transcription start
CsDof40	TATA-box	872	876	core promoter element around -30 of transcription start
CsDof40	TATA-box	1013	1021	core promoter element around -30 of transcription start
CsDof40	TATA-box	1306	1312	core promoter element around -30 of transcription start
CsDof40	TATA-box	1307	1312	core promoter element around -30 of transcription start
CsDof40	TATA-box	1308	1312	core promoter element around -30 of transcription start
CsDof40	TATA-box	1573	1581	core promoter element around -30 of transcription start
CsDof40	TATA-box	1576	1581	core promoter element around -30 of transcription start
CsDof40	TATA-box	1577	1581	core promoter element around -30 of transcription start
CsDof40	TATA-box	1589	1596	core promoter element around -30 of transcription start
CsDof40	TATA-box	1966	1970	core promoter element around -30 of transcription start
CsDof40	3-AF1 binding site	1633	1643	light responsive element
CsDof40	GT1-motif	254	261	light responsive element
CsDof40	CCAAT-box	1296	1302	MYBHv1 binding site
CsDof41	ABRE	641	650	abscisic acid responsiveness
CsDof41	ABRE	643	649	abscisic acid responsiveness
CsDof41	ABRE	644	649	abscisic acid responsiveness
CsDof41	ABRE	867	875	abscisic acid responsiveness
CsDof41	ABRE	869	874	abscisic acid responsiveness
CsDof41	ABRE	1425	1430	abscisic acid responsiveness
CsDof41	TGA-element	648	654	auxin-responsive element
CsDof41	LTR	975	981	cis-acting element involved in low-temperature responsiveness
CsDof41	TCA-element	367	376	cis-acting element involved in salicylic acid responsiveness
CsDof41	TCA-element	895	904	cis-acting element involved in salicylic acid responsiveness
CsDof41	A-box	460	466	cis-acting regulatory element
CsDof41	A-box	1639	1645	cis-acting regulatory element
CsDof41	ARE	1877	1883	cis-acting regulatory element essential for the anaerobic induction

Name	Cis-element	Start position	Stop position	Function
CsDof41	G-box	642	650	cis-acting regulatory element involved in light responsiveness
CsDof41	G-box	643	649	cis-acting regulatory element involved in light responsiveness
CsDof41	G-box	869	875	cis-acting regulatory element involved in light responsiveness
CsDof41	G-Box	643	649	cis-acting regulatory element involved in light responsiveness
CsDof41	G-Box	1425	1431	cis-acting regulatory element involved in light responsiveness
CsDof41	TGACG-motif	235	240	cis-acting regulatory element involved in the MeJA-responsiveness
CsDof41	TGACG-motif	718	723	cis-acting regulatory element involved in the MeJA-responsiveness
CsDof41	TGACG-motif	1281	1286	cis-acting regulatory element involved in the MeJA-responsiveness
CsDof41	TGACG-motif	1284	1289	cis-acting regulatory element involved in the MeJA-responsiveness
CsDof41	CGTCA-motif	235	240	cis-acting regulatory element involved in the MeJA-responsiveness
CsDof41	CGTCA-motif	718	723	cis-acting regulatory element involved in the MeJA-responsiveness
CsDof41	CGTCA-motif	1281	1286	cis-acting regulatory element involved in the MeJA-responsiveness
CsDof41	CGTCA-motif	1284	1289	cis-acting regulatory element involved in the MeJA-responsiveness
CsDof41	CAT-box	1245	1251	cis-acting regulatory element related to meristem expression
CsDof41	CAAT-box	201	206	common cis-acting element in promoter and enhancer regions
CsDof41	CAAT-box	220	225	common cis-acting element in promoter and enhancer regions
CsDof41	CAAT-box	480	485	common cis-acting element in promoter and enhancer regions
CsDof41	CAAT-box	626	631	common cis-acting element in promoter and enhancer regions
CsDof41	CAAT-box	712	717	common cis-acting element in promoter and enhancer regions
CsDof41	CAAT-box	1012	1017	common cis-acting element in promoter and enhancer regions
CsDof41	CAAT-box	1143	1148	common cis-acting element in promoter and enhancer regions
CsDof41	CAAT-box	1238	1243	common cis-acting element in promoter and enhancer regions
CsDof41	CAAT-box	1836	1841	common cis-acting element in promoter and enhancer regions
CsDof41	TATA-box	2	8	core promoter element around -30 of transcription start
CsDof41	TATA-box	4	8	core promoter element around -30 of transcription start
CsDof41	TATA-box	102	106	core promoter element around -30 of transcription start
CsDof41	TATA-box	172	178	core promoter element around -30 of transcription start
CsDof41	TATA-box	173	177	core promoter element around -30 of transcription start
CsDof41	TATA-box	196	202	core promoter element around -30 of transcription start
CsDof41	TATA-box	197	202	core promoter element around -30 of transcription start
CsDof41	TATA-box	198	202	core promoter element around -30 of transcription start
CsDof41	TATA-box	382	386	core promoter element around -30 of transcription start
CsDof41	TATA-box	673	679	core promoter element around -30 of transcription start
CsDof41	TATA-box	675	681	core promoter element around -30 of transcription start
CsDof41	TATA-box	676	682	core promoter element around -30 of transcription start
CsDof41	TATA-box	677	681	core promoter element around -30 of transcription start
CsDof41	TATA-box	1043	1048	core promoter element around -30 of transcription start
CsDof41	TATA-box	1044	1048	core promoter element around -30 of transcription start
CsDof41	TATA-box	1049	1055	core promoter element around -30 of transcription start
CsDof41	TATA-box	1051	1055	core promoter element around -30 of transcription start
CsDof41	TATA-box	1056	1060	core promoter element around -30 of transcription start
CsDof41	TATA-box	1070	1076	core promoter element around -30 of transcription start
CsDof41	TATA-box	1071	1075	core promoter element around -30 of transcription start
CsDof41	TATA-box	1118	1123	core promoter element around -30 of transcription start
CsDof41	TATA-box	1119	1123	core promoter element around -30 of transcription start
CsDof41	TATA-box	1314	1321	core promoter element around -30 of transcription start
CsDof41	TATA-box	1317	1323	core promoter element around -30 of transcription start
CsDof41	TATA-box	1319	1323	core promoter element around -30 of transcription start
CsDof41	TATA-box	1371	1379	core promoter element around -30 of transcription start
CsDof41	TATA-box	1374	1381	core promoter element around -30 of transcription start
CsDof41	TATA-box	1375	1381	core promoter element around -30 of transcription start

Name	Cis-element	Start position	Stop position	Function
CsDof41	TATA-box	1376	1382	core promoter element around -30 of transcription start
CsDof41	TATA-box	1377	1381	core promoter element around -30 of transcription start
CsDof41	TATA-box	1414	1420	core promoter element around -30 of transcription start
CsDof41	TATA-box	1415	1421	core promoter element around -30 of transcription start
CsDof41	TATA-box	1416	1422	core promoter element around -30 of transcription start
CsDof41	TATA-box	1417	1421	core promoter element around -30 of transcription start
CsDof41	TATA-box	1473	1477	core promoter element around -30 of transcription start
CsDof41	TATA-box	1492	1498	core promoter element around -30 of transcription start
CsDof41	TATA-box	1493	1498	core promoter element around -30 of transcription start
CsDof41	TATA-box	1494	1498	core promoter element around -30 of transcription start
CsDof41	TATA-box	1780	1784	core promoter element around -30 of transcription start
CsDof41	TATA-box	1802	1810	core promoter element around -30 of transcription start
CsDof41	TATA-box	1805	1817	core promoter element around -30 of transcription start
CsDof41	TATA-box	1808	1812	core promoter element around -30 of transcription start
CsDof41	TATA-box	1813	1819	core promoter element around -30 of transcription start
CsDof41	TATA-box	1814	1820	core promoter element around -30 of transcription start
CsDof41	TATA-box	1816	1820	core promoter element around -30 of transcription start
CsDof41	TATA-box	1841	1850	core promoter element around -30 of transcription start
CsDof41	TATA-box	1842	1849	core promoter element around -30 of transcription start
CsDof41	TATA-box	1843	1849	core promoter element around -30 of transcription start
CsDof41	TATA-box	1844	1851	core promoter element around -30 of transcription start
CsDof41	TATA-box	1845	1851	core promoter element around -30 of transcription start
CsDof41	TATA-box	1846	1852	core promoter element around -30 of transcription start
CsDof41	TATA-box	1847	1851	core promoter element around -30 of transcription start
CsDof41	GC-motif	1645	1651	enhancer-like element involved in anoxic specific inducibility
CsDof41	CCAAT-box	779	785	MYBHv1 binding site
CsDof42	ABRE	769	775	abscisic acid responsiveness
CsDof42	ABRE	770	775	abscisic acid responsiveness
CsDof42	ABRE	839	848	abscisic acid responsiveness
CsDof42	ABRE	841	847	abscisic acid responsiveness
CsDof42	ABRE	842	847	abscisic acid responsiveness
CsDof42	TGA-element	1752	1758	auxin-responsive element
CsDof42	ARE	282	288	cis-acting regulatory element essential for the anaerobic induction
CsDof42	ARE	621	627	cis-acting regulatory element essential for the anaerobic induction
CsDof42	ARE	1120	1126	cis-acting regulatory element essential for the anaerobic induction
CsDof42	ARE	1526	1532	cis-acting regulatory element essential for the anaerobic induction
CsDof42	circadian	291	300	cis-acting regulatory element involved in circadian control
CsDof42	circadian	719	728	cis-acting regulatory element involved in circadian control
CsDof42	circadian	1597	1606	cis-acting regulatory element involved in circadian control
CsDof42	G-Box	769	775	cis-acting regulatory element involved in light responsiveness
CsDof42	G-Box	841	847	cis-acting regulatory element involved in light responsiveness
CsDof42	G-box	768	776	cis-acting regulatory element involved in light responsiveness
CsDof42	G-box	769	775	cis-acting regulatory element involved in light responsiveness
CsDof42	G-box	841	847	cis-acting regulatory element involved in light responsiveness
CsDof42	CAAT-box	247	252	common cis-acting element in promoter and enhancer regions
CsDof42	CAAT-box	291	296	common cis-acting element in promoter and enhancer regions
CsDof42	CAAT-box	337	342	common cis-acting element in promoter and enhancer regions
CsDof42	CAAT-box	927	932	common cis-acting element in promoter and enhancer regions
CsDof42	CAAT-box	1472	1477	common cis-acting element in promoter and enhancer regions
CsDof42	CAAT-box	1610	1615	common cis-acting element in promoter and enhancer regions
CsDof42	CAAT-box	1711	1716	common cis-acting element in promoter and enhancer regions

Name	Cis-element	Start position	Stop position	Function
CsDof42	CAAT-box	1870	1875	common cis-acting element in promoter and enhancer regions
CsDof42	CAAT-box	1891	1896	common cis-acting element in promoter and enhancer regions
CsDof42	TATA-box	17	23	core promoter element around -30 of transcription start
CsDof42	TATA-box	18	22	core promoter element around -30 of transcription start
CsDof42	TATA-box	71	77	core promoter element around -30 of transcription start
CsDof42	TATA-box	73	77	core promoter element around -30 of transcription start
CsDof42	TATA-box	97	101	core promoter element around -30 of transcription start
CsDof42	TATA-box	120	126	core promoter element around -30 of transcription start
CsDof42	TATA-box	121	125	core promoter element around -30 of transcription start
CsDof42	TATA-box	235	243	core promoter element around -30 of transcription start
CsDof42	TATA-box	239	248	core promoter element around -30 of transcription start
CsDof42	TATA-box	240	247	core promoter element around -30 of transcription start
CsDof42	TATA-box	241	247	core promoter element around -30 of transcription start
CsDof42	TATA-box	242	247	core promoter element around -30 of transcription start
CsDof42	TATA-box	243	247	core promoter element around -30 of transcription start
CsDof42	TATA-box	294	300	core promoter element around -30 of transcription start
CsDof42	TATA-box	295	299	core promoter element around -30 of transcription start
CsDof42	TATA-box	365	371	core promoter element around -30 of transcription start
CsDof42	TATA-box	366	371	core promoter element around -30 of transcription start
CsDof42	TATA-box	367	371	core promoter element around -30 of transcription start
CsDof42	TATA-box	446	453	core promoter element around -30 of transcription start
CsDof42	TATA-box	449	455	core promoter element around -30 of transcription start
CsDof42	TATA-box	451	455	core promoter element around -30 of transcription start
CsDof42	TATA-box	466	472	core promoter element around -30 of transcription start
CsDof42	TATA-box	468	472	core promoter element around -30 of transcription start
CsDof42	TATA-box	527	536	core promoter element around -30 of transcription start
CsDof42	TATA-box	528	534	core promoter element around -30 of transcription start
CsDof42	TATA-box	529	536	core promoter element around -30 of transcription start
CsDof42	TATA-box	530	536	core promoter element around -30 of transcription start
CsDof42	TATA-box	531	537	core promoter element around -30 of transcription start
CsDof42	TATA-box	532	536	core promoter element around -30 of transcription start
CsDof42	TATA-box	699	706	core promoter element around -30 of transcription start
CsDof42	TATA-box	951	957	core promoter element around -30 of transcription start
CsDof42	TATA-box	952	956	core promoter element around -30 of transcription start
CsDof42	TATA-box	977	981	core promoter element around -30 of transcription start
CsDof42	TATA-box	1094	1100	core promoter element around -30 of transcription start
CsDof42	TATA-box	1095	1101	core promoter element around -30 of transcription start
CsDof42	TATA-box	1096	1100	core promoter element around -30 of transcription start
CsDof42	TATA-box	1183	1189	core promoter element around -30 of transcription start
CsDof42	TATA-box	1185	1189	core promoter element around -30 of transcription start
CsDof42	TATA-box	1192	1198	core promoter element around -30 of transcription start
CsDof42	TATA-box	1194	1198	core promoter element around -30 of transcription start
CsDof42	TATA-box	1227	1239	core promoter element around -30 of transcription start
CsDof42	TATA-box	1236	1240	core promoter element around -30 of transcription start
CsDof42	TATA-box	1620	1628	core promoter element around -30 of transcription start
CsDof42	TATA-box	1766	1770	core promoter element around -30 of transcription start
CsDof42	TATA-box	1785	1791	core promoter element around -30 of transcription start
CsDof42	TATA-box	1787	1793	core promoter element around -30 of transcription start
CsDof42	TATA-box	1788	1794	core promoter element around -30 of transcription start
CsDof42	TATA-box	1789	1795	core promoter element around -30 of transcription start
CsDof42	TATA-box	1790	1796	core promoter element around -30 of transcription start

Name	Cis-element	Start position	Stop position	Function
CsDof42	TATA-box	1791	1797	core promoter element around -30 of transcription start
CsDof42	TATA-box	1792	1798	core promoter element around -30 of transcription start
CsDof42	TATA-box	1793	1799	core promoter element around -30 of transcription start
CsDof42	TATA-box	1794	1800	core promoter element around -30 of transcription start
CsDof42	TATA-box	1795	1801	core promoter element around -30 of transcription start
CsDof42	TATA-box	1796	1802	core promoter element around -30 of transcription start
CsDof42	TATA-box	1797	1803	core promoter element around -30 of transcription start
CsDof42	TATA-box	1798	1804	core promoter element around -30 of transcription start
CsDof42	TATA-box	1799	1805	core promoter element around -30 of transcription start
CsDof42	TATA-box	1800	1806	core promoter element around -30 of transcription start
CsDof42	TATA-box	1801	1805	core promoter element around -30 of transcription start
CsDof42	AT-rich sequence	1858	1867	element for maximal elicitor-mediated activation (2copies)
CsDof42	MRE	1016	1023	MYB binding site involved in light responsiveness
CsDof42	MRE	1541	1548	MYB binding site involved in light responsiveness
CsDof42	CCAAT-box	945	951	MYBHv1 binding site
CsDof43	ABRE	1107	1112	abscisic acid responsiveness
CsDof43	LTR	434	440	cis-acting element involved in low-temperature responsiveness
CsDof43	ARE	189	195	cis-acting regulatory element essential for the anaerobic induction
CsDof43	ARE	370	376	cis-acting regulatory element essential for the anaerobic induction
CsDof43	ARE	858	864	cis-acting regulatory element essential for the anaerobic induction
CsDof43	ARE	1945	1951	cis-acting regulatory element essential for the anaerobic induction
CsDof43	G-box	1107	1113	cis-acting regulatory element involved in light responsiveness
CsDof43	TGACG-motif	887	892	cis-acting regulatory element involved in the MeJA-responsiveness
CsDof43	TGACG-motif	942	947	cis-acting regulatory element involved in the MeJA-responsiveness
CsDof43	CGTCA-motif	887	892	cis-acting regulatory element involved in the MeJA-responsiveness
CsDof43	CGTCA-motif	942	947	cis-acting regulatory element involved in the MeJA-responsiveness
CsDof43	CAAT-box	163	168	common cis-acting element in promoter and enhancer regions
CsDof43	CAAT-box	225	230	common cis-acting element in promoter and enhancer regions
CsDof43	CAAT-box	241	246	common cis-acting element in promoter and enhancer regions
CsDof43	CAAT-box	256	261	common cis-acting element in promoter and enhancer regions
CsDof43	CAAT-box	271	276	common cis-acting element in promoter and enhancer regions
CsDof43	CAAT-box	360	365	common cis-acting element in promoter and enhancer regions
CsDof43	CAAT-box	512	517	common cis-acting element in promoter and enhancer regions
CsDof43	CAAT-box	865	870	common cis-acting element in promoter and enhancer regions
CsDof43	CAAT-box	1349	1354	common cis-acting element in promoter and enhancer regions
CsDof43	CAAT-box	1669	1674	common cis-acting element in promoter and enhancer regions
CsDof43	CAAT-box	1672	1677	common cis-acting element in promoter and enhancer regions
CsDof43	CAAT-box	1728	1733	common cis-acting element in promoter and enhancer regions
CsDof43	TATA-box	168	172	core promoter element around -30 of transcription start
CsDof43	TATA-box	173	177	core promoter element around -30 of transcription start
CsDof43	TATA-box	179	185	core promoter element around -30 of transcription start
CsDof43	TATA-box	180	185	core promoter element around -30 of transcription start
CsDof43	TATA-box	181	185	core promoter element around -30 of transcription start
CsDof43	TATA-box	266	275	core promoter element around -30 of transcription start
CsDof43	TATA-box	312	319	core promoter element around -30 of transcription start
CsDof43	TATA-box	313	319	core promoter element around -30 of transcription start
CsDof43	TATA-box	314	319	core promoter element around -30 of transcription start
CsDof43	TATA-box	315	319	core promoter element around -30 of transcription start
CsDof43	TATA-box	455	459	core promoter element around -30 of transcription start
CsDof43	TATA-box	492	499	core promoter element around -30 of transcription start
CsDof43	TATA-box	621	630	core promoter element around -30 of transcription start

Name	Cis-element	Start position	Stop position	Function
CsDof43	TATA-box	622	628	core promoter element around -30 of transcription start
CsDof43	TATA-box	623	630	core promoter element around -30 of transcription start
CsDof43	TATA-box	624	630	core promoter element around -30 of transcription start
CsDof43	TATA-box	626	630	core promoter element around -30 of transcription start
CsDof43	TATA-box	697	704	core promoter element around -30 of transcription start
CsDof43	TATA-box	698	704	core promoter element around -30 of transcription start
CsDof43	TATA-box	699	704	core promoter element around -30 of transcription start
CsDof43	TATA-box	700	704	core promoter element around -30 of transcription start
CsDof43	TATA-box	704	710	core promoter element around -30 of transcription start
CsDof43	TATA-box	705	709	core promoter element around -30 of transcription start
CsDof43	TATA-box	780	787	core promoter element around -30 of transcription start
CsDof43	TATA-box	781	787	core promoter element around -30 of transcription start
CsDof43	TATA-box	782	788	core promoter element around -30 of transcription start
CsDof43	TATA-box	783	787	core promoter element around -30 of transcription start
CsDof43	TATA-box	962	968	core promoter element around -30 of transcription start
CsDof43	TATA-box	963	968	core promoter element around -30 of transcription start
CsDof43	TATA-box	964	968	core promoter element around -30 of transcription start
CsDof43	TATA-box	1017	1024	core promoter element around -30 of transcription start
CsDof43	TATA-box	1101	1105	core promoter element around -30 of transcription start
CsDof43	TATA-box	1113	1119	core promoter element around -30 of transcription start
CsDof43	TATA-box	1114	1118	core promoter element around -30 of transcription start
CsDof43	TATA-box	1132	1138	core promoter element around -30 of transcription start
CsDof43	TATA-box	1133	1139	core promoter element around -30 of transcription start
CsDof43	TATA-box	1134	1140	core promoter element around -30 of transcription start
CsDof43	TATA-box	1135	1139	core promoter element around -30 of transcription start
CsDof43	TATA-box	1209	1215	core promoter element around -30 of transcription start
CsDof43	TATA-box	1210	1214	core promoter element around -30 of transcription start
CsDof43	TATA-box	1218	1224	core promoter element around -30 of transcription start
CsDof43	TATA-box	1219	1225	core promoter element around -30 of transcription start
CsDof43	TATA-box	1220	1226	core promoter element around -30 of transcription start
CsDof43	TATA-box	1221	1225	core promoter element around -30 of transcription start
CsDof43	TATA-box	1252	1260	core promoter element around -30 of transcription start
CsDof43	TATA-box	1255	1260	core promoter element around -30 of transcription start
CsDof43	TATA-box	1256	1260	core promoter element around -30 of transcription start
CsDof43	TATA-box	1271	1277	core promoter element around -30 of transcription start
CsDof43	TATA-box	1272	1276	core promoter element around -30 of transcription start
CsDof43	TATA-box	1343	1350	core promoter element around -30 of transcription start
CsDof43	TATA-box	1402	1408	core promoter element around -30 of transcription start
CsDof43	TATA-box	1403	1407	core promoter element around -30 of transcription start
CsDof43	TATA-box	1523	1529	core promoter element around -30 of transcription start
CsDof43	TATA-box	1524	1530	core promoter element around -30 of transcription start
CsDof43	TATA-box	1526	1530	core promoter element around -30 of transcription start
CsDof43	TATA-box	1531	1537	core promoter element around -30 of transcription start
CsDof43	TATA-box	1532	1538	core promoter element around -30 of transcription start
CsDof43	TATA-box	1533	1539	core promoter element around -30 of transcription start
CsDof43	TATA-box	1534	1540	core promoter element around -30 of transcription start
CsDof43	TATA-box	1535	1541	core promoter element around -30 of transcription start
CsDof43	TATA-box	1536	1542	core promoter element around -30 of transcription start
CsDof43	TATA-box	1537	1543	core promoter element around -30 of transcription start
CsDof43	TATA-box	1538	1544	core promoter element around -30 of transcription start
CsDof43	TATA-box	1539	1545	core promoter element around -30 of transcription start

Name	Cis-element	Start position	Stop position	Function
CsDof43	TATA-box	1540	1546	core promoter element around -30 of transcription start
CsDof43	TATA-box	1541	1547	core promoter element around -30 of transcription start
CsDof43	TATA-box	1542	1548	core promoter element around -30 of transcription start
CsDof43	TATA-box	1543	1549	core promoter element around -30 of transcription start
CsDof43	TATA-box	1544	1550	core promoter element around -30 of transcription start
CsDof43	TATA-box	1545	1551	core promoter element around -30 of transcription start
CsDof43	TATA-box	1546	1552	core promoter element around -30 of transcription start
CsDof43	TATA-box	1547	1553	core promoter element around -30 of transcription start
CsDof43	TATA-box	1548	1552	core promoter element around -30 of transcription start
CsDof43	TATA-box	1675	1681	core promoter element around -30 of transcription start
CsDof43	TATA-box	1677	1683	core promoter element around -30 of transcription start
CsDof43	TATA-box	1678	1684	core promoter element around -30 of transcription start
CsDof43	TATA-box	1679	1683	core promoter element around -30 of transcription start
CsDof43	TATA-box	1683	1689	core promoter element around -30 of transcription start
CsDof43	TATA-box	1684	1690	core promoter element around -30 of transcription start
CsDof43	TATA-box	1685	1691	core promoter element around -30 of transcription start
CsDof43	TATA-box	1686	1690	core promoter element around -30 of transcription start
CsDof43	TATA-box	1734	1739	core promoter element around -30 of transcription start
CsDof43	TATA-box	1735	1739	core promoter element around -30 of transcription start
CsDof43	TATA-box	1752	1756	core promoter element around -30 of transcription start
CsDof43	TATA-box	1764	1770	core promoter element around -30 of transcription start
CsDof43	TATA-box	1765	1769	core promoter element around -30 of transcription start
CsDof43	TATA-box	1788	1794	core promoter element around -30 of transcription start
CsDof43	TATA-box	1789	1795	core promoter element around -30 of transcription start
CsDof43	TATA-box	1790	1796	core promoter element around -30 of transcription start
CsDof43	TATA-box	1791	1797	core promoter element around -30 of transcription start
CsDof43	TATA-box	1792	1796	core promoter element around -30 of transcription start
CsDof43	TATA-box	1804	1810	core promoter element around -30 of transcription start
CsDof43	TATA-box	1806	1812	core promoter element around -30 of transcription start
CsDof43	TATA-box	1807	1813	core promoter element around -30 of transcription start
CsDof43	TATA-box	1808	1814	core promoter element around -30 of transcription start
CsDof43	TATA-box	1809	1815	core promoter element around -30 of transcription start
CsDof43	TATA-box	1810	1814	core promoter element around -30 of transcription start
CsDof43	TATA-box	1891	1897	core promoter element around -30 of transcription start
CsDof43	TATA-box	1892	1896	core promoter element around -30 of transcription start
CsDof43	AT-rich sequence	393	402	element for maximal elicitor-mediated activation (2copies)
CsDof43	GT1-motif	891	897	light responsive element
CsDof43	MBS	201	207	MYB binding site involved in drought-inducibility
CsDof44	ABRE	1025	1032	abscisic acid responsiveness
CsDof44	ABRE	1381	1386	abscisic acid responsiveness
CsDof44	ABRE	1665	1670	abscisic acid responsiveness
CsDof44	TGA-element	181	187	auxin-responsive element
CsDof44	TCA-element	787	796	cis-acting element involved in salicylic acid responsiveness
CsDof44	ARE	400	406	cis-acting regulatory element essential for the anaerobic induction
CsDof44	ARE	477	483	cis-acting regulatory element essential for the anaerobic induction
CsDof44	ARE	1082	1088	cis-acting regulatory element essential for the anaerobic induction
CsDof44	G-Box	1380	1386	cis-acting regulatory element involved in light responsiveness
CsDof44	G-Box	1665	1671	cis-acting regulatory element involved in light responsiveness
CsDof44	CGTCA-motif	1070	1075	cis-acting regulatory element involved in the MeJA-responsiveness
CsDof44	TGACG-motif	1070	1075	cis-acting regulatory element involved in the MeJA-responsiveness
CsDof44	O2-site	1234	1244	cis-acting regulatory element involved in zein metabolism regulation

Name	Cis-element	Start position	Stop position	Function
CsDof44	CAAT-box	120	125	common cis-acting element in promoter and enhancer regions
CsDof44	CAAT-box	299	304	common cis-acting element in promoter and enhancer regions
CsDof44	CAAT-box	440	445	common cis-acting element in promoter and enhancer regions
CsDof44	CAAT-box	451	456	common cis-acting element in promoter and enhancer regions
CsDof44	CAAT-box	1005	1010	common cis-acting element in promoter and enhancer regions
CsDof44	CAAT-box	1064	1069	common cis-acting element in promoter and enhancer regions
CsDof44	CAAT-box	1076	1081	common cis-acting element in promoter and enhancer regions
CsDof44	CAAT-box	1330	1335	common cis-acting element in promoter and enhancer regions
CsDof44	CAAT-box	1346	1351	common cis-acting element in promoter and enhancer regions
CsDof44	CAAT-box	1546	1551	common cis-acting element in promoter and enhancer regions
CsDof44	CAAT-box	1748	1753	common cis-acting element in promoter and enhancer regions
CsDof44	CAAT-box	1840	1845	common cis-acting element in promoter and enhancer regions
CsDof44	TATA-box	27	31	core promoter element around -30 of transcription start
CsDof44	TATA-box	262	268	core promoter element around -30 of transcription start
CsDof44	TATA-box	263	269	core promoter element around -30 of transcription start
CsDof44	TATA-box	265	269	core promoter element around -30 of transcription start
CsDof44	TATA-box	321	325	core promoter element around -30 of transcription start
CsDof44	TATA-box	372	376	core promoter element around -30 of transcription start
CsDof44	TATA-box	379	383	core promoter element around -30 of transcription start
CsDof44	TATA-box	394	400	core promoter element around -30 of transcription start
CsDof44	TATA-box	395	400	core promoter element around -30 of transcription start
CsDof44	TATA-box	396	400	core promoter element around -30 of transcription start
CsDof44	TATA-box	409	413	core promoter element around -30 of transcription start
CsDof44	TATA-box	435	439	core promoter element around -30 of transcription start
CsDof44	TATA-box	532	539	core promoter element around -30 of transcription start
CsDof44	TATA-box	763	770	core promoter element around -30 of transcription start
CsDof44	TATA-box	869	873	core promoter element around -30 of transcription start
CsDof44	TATA-box	1100	1106	core promoter element around -30 of transcription start
CsDof44	TATA-box	1101	1107	core promoter element around -30 of transcription start
CsDof44	TATA-box	1102	1108	core promoter element around -30 of transcription start
CsDof44	TATA-box	1103	1107	core promoter element around -30 of transcription start
CsDof44	TATA-box	1110	1117	core promoter element around -30 of transcription start
CsDof44	TATA-box	1269	1276	core promoter element around -30 of transcription start
CsDof44	TATA-box	1291	1295	core promoter element around -30 of transcription start
CsDof44	TATA-box	1303	1310	core promoter element around -30 of transcription start
CsDof44	TATA-box	1368	1374	core promoter element around -30 of transcription start
CsDof44	TATA-box	1369	1374	core promoter element around -30 of transcription start
CsDof44	TATA-box	1370	1374	core promoter element around -30 of transcription start
CsDof44	TATA-box	1429	1435	core promoter element around -30 of transcription start
CsDof44	TATA-box	1430	1436	core promoter element around -30 of transcription start
CsDof44	TATA-box	1431	1437	core promoter element around -30 of transcription start
CsDof44	TATA-box	1432	1438	core promoter element around -30 of transcription start
CsDof44	TATA-box	1433	1439	core promoter element around -30 of transcription start
CsDof44	TATA-box	1434	1440	core promoter element around -30 of transcription start
CsDof44	TATA-box	1435	1441	core promoter element around -30 of transcription start
CsDof44	TATA-box	1436	1442	core promoter element around -30 of transcription start
CsDof44	TATA-box	1437	1443	core promoter element around -30 of transcription start
CsDof44	TATA-box	1438	1444	core promoter element around -30 of transcription start
CsDof44	TATA-box	1439	1445	core promoter element around -30 of transcription start
CsDof44	TATA-box	1440	1446	core promoter element around -30 of transcription start
CsDof44	TATA-box	1441	1447	core promoter element around -30 of transcription start

Name	Cis-element	Start position	Stop position	Function
CsDof44	TATA-box	1442	1454	core promoter element around -30 of transcription start
CsDof44	TATA-box	1443	1449	core promoter element around -30 of transcription start
CsDof44	TATA-box	1444	1450	core promoter element around -30 of transcription start
CsDof44	TATA-box	1445	1451	core promoter element around -30 of transcription start
CsDof44	TATA-box	1446	1452	core promoter element around -30 of transcription start
CsDof44	TATA-box	1447	1453	core promoter element around -30 of transcription start
CsDof44	TATA-box	1448	1452	core promoter element around -30 of transcription start
CsDof44	TATA-box	1537	1543	core promoter element around -30 of transcription start
CsDof44	TATA-box	1538	1544	core promoter element around -30 of transcription start
CsDof44	TATA-box	1539	1543	core promoter element around -30 of transcription start
CsDof44	TATA-box	1596	1600	core promoter element around -30 of transcription start
CsDof44	TATA-box	1609	1617	core promoter element around -30 of transcription start
CsDof44	TATA-box	1723	1729	core promoter element around -30 of transcription start
CsDof44	TATA-box	1724	1728	core promoter element around -30 of transcription start
CsDof44	TATA-box	1811	1817	core promoter element around -30 of transcription start
CsDof44	TATA-box	1812	1816	core promoter element around -30 of transcription start
CsDof44	TATA-box	1872	1878	core promoter element around -30 of transcription start
CsDof44	TATA-box	1873	1879	core promoter element around -30 of transcription start
CsDof44	TATA-box	1874	1880	core promoter element around -30 of transcription start
CsDof44	TATA-box	1876	1880	core promoter element around -30 of transcription start
CsDof44	GARE-motif	498	505	gibberellin-responsive element
CsDof45	TGA-element	569	575	auxin-responsive element
CsDof45	TC-rich repeats	281	290	cis-acting element involved in defense and stress responsiveness
CsDof45	ARE	160	166	cis-acting regulatory element essential for the anaerobic induction
CsDof45	ARE	584	590	cis-acting regulatory element essential for the anaerobic induction
CsDof45	G-Box	393	403	cis-acting regulatory element involved in light responsiveness
CsDof45	CGTCA-motif	895	900	cis-acting regulatory element involved in the MeJA-responsiveness
CsDof45	TGACG-motif	895	900	cis-acting regulatory element involved in the MeJA-responsiveness
CsDof45	CAAT-box	121	126	common cis-acting element in promoter and enhancer regions
CsDof45	CAAT-box	158	163	common cis-acting element in promoter and enhancer regions
CsDof45	CAAT-box	185	190	common cis-acting element in promoter and enhancer regions
CsDof45	CAAT-box	210	215	common cis-acting element in promoter and enhancer regions
CsDof45	CAAT-box	278	283	common cis-acting element in promoter and enhancer regions
CsDof45	CAAT-box	322	327	common cis-acting element in promoter and enhancer regions
CsDof45	CAAT-box	679	684	common cis-acting element in promoter and enhancer regions
CsDof45	CAAT-box	883	888	common cis-acting element in promoter and enhancer regions
CsDof45	TATA-box	42	46	core promoter element around -30 of transcription start
CsDof45	TATA-box	134	142	core promoter element around -30 of transcription start
CsDof45	TATA-box	137	142	core promoter element around -30 of transcription start
CsDof45	TATA-box	138	142	core promoter element around -30 of transcription start
CsDof45	TATA-box	197	201	core promoter element around -30 of transcription start
CsDof45	TATA-box	315	320	core promoter element around -30 of transcription start
CsDof45	TATA-box	316	320	core promoter element around -30 of transcription start
CsDof45	TATA-box	327	334	core promoter element around -30 of transcription start
CsDof45	TATA-box	431	437	core promoter element around -30 of transcription start
CsDof45	TATA-box	432	436	core promoter element around -30 of transcription start
CsDof45	TATA-box	485	491	core promoter element around -30 of transcription start
CsDof45	TATA-box	486	490	core promoter element around -30 of transcription start
CsDof45	TATA-box	628	637	core promoter element around -30 of transcription start
CsDof45	TATA-box	629	636	core promoter element around -30 of transcription start
CsDof45	TATA-box	630	636	core promoter element around -30 of transcription start

Name	Cis-element	Start position	Stop position	Function
CsDof45	TATA-box	631	638	core promoter element around -30 of transcription start
CsDof45	TATA-box	632	638	core promoter element around -30 of transcription start
CsDof45	TATA-box	633	639	core promoter element around -30 of transcription start
CsDof45	TATA-box	634	638	core promoter element around -30 of transcription start
CsDof45	TATA-box	674	680	core promoter element around -30 of transcription start
CsDof45	TATA-box	676	680	core promoter element around -30 of transcription start
CsDof45	TATA-box	691	697	core promoter element around -30 of transcription start
CsDof45	TATA-box	692	698	core promoter element around -30 of transcription start
CsDof45	TATA-box	693	699	core promoter element around -30 of transcription start
CsDof45	TATA-box	694	698	core promoter element around -30 of transcription start
CsDof45	TATA-box	777	783	core promoter element around -30 of transcription start
CsDof45	TATA-box	778	783	core promoter element around -30 of transcription start
CsDof45	TATA-box	779	783	core promoter element around -30 of transcription start
CsDof45	TATA-box	789	795	core promoter element around -30 of transcription start
CsDof45	TATA-box	790	794	core promoter element around -30 of transcription start
CsDof45	MBS	143	149	MYB binding site involved in drought-inducibility
CsDof45	MBS	508	514	MYB binding site involved in drought-inducibility
CsDof45	MBS	526	532	MYB binding site involved in drought-inducibility
CsDof45	MRE	24	31	MYB binding site involved in light responsiveness
CsDof46	ABRE	66	71	abscisic acid responsiveness
CsDof46	ABRE	979	986	abscisic acid responsiveness
CsDof46	ABRE	1070	1079	abscisic acid responsiveness
CsDof46	ABRE	1073	1078	abscisic acid responsiveness
CsDof46	ABRE	1161	1166	abscisic acid responsiveness
CsDof46	TGA-element	1005	1011	auxin-responsive element
CsDof46	TGA-element	1393	1399	auxin-responsive element
CsDof46	TCA-element	1983	1992	cis-acting element involved in salicylic acid responsiveness
CsDof46	ARE	1057	1063	cis-acting regulatory element essential for the anaerobic induction
CsDof46	ARE	1348	1354	cis-acting regulatory element essential for the anaerobic induction
CsDof46	ARE	1890	1896	cis-acting regulatory element essential for the anaerobic induction
CsDof46	G-Box	65	71	cis-acting regulatory element involved in light responsiveness
CsDof46	G-Box	1072	1078	cis-acting regulatory element involved in light responsiveness
CsDof46	G-Box	1160	1166	cis-acting regulatory element involved in light responsiveness
CsDof46	CAAT-box	6	11	common cis-acting element in promoter and enhancer regions
CsDof46	CAAT-box	117	122	common cis-acting element in promoter and enhancer regions
CsDof46	CAAT-box	333	338	common cis-acting element in promoter and enhancer regions
CsDof46	CAAT-box	458	463	common cis-acting element in promoter and enhancer regions
CsDof46	CAAT-box	501	506	common cis-acting element in promoter and enhancer regions
CsDof46	CAAT-box	648	653	common cis-acting element in promoter and enhancer regions
CsDof46	CAAT-box	694	699	common cis-acting element in promoter and enhancer regions
CsDof46	CAAT-box	847	852	common cis-acting element in promoter and enhancer regions
CsDof46	CAAT-box	1420	1425	common cis-acting element in promoter and enhancer regions
CsDof46	CAAT-box	1478	1483	common cis-acting element in promoter and enhancer regions
CsDof46	CAAT-box	1711	1716	common cis-acting element in promoter and enhancer regions
CsDof46	TATA-box	155	159	core promoter element around -30 of transcription start
CsDof46	TATA-box	195	201	core promoter element around -30 of transcription start
CsDof46	TATA-box	196	201	core promoter element around -30 of transcription start
CsDof46	TATA-box	197	201	core promoter element around -30 of transcription start
CsDof46	TATA-box	245	253	core promoter element around -30 of transcription start
CsDof46	TATA-box	251	259	core promoter element around -30 of transcription start
CsDof46	TATA-box	253	261	core promoter element around -30 of transcription start

Name	Cis-element	Start position	Stop position	Function
CsDof46	TATA-box	282	286	core promoter element around -30 of transcription start
CsDof46	TATA-box	321	328	core promoter element around -30 of transcription start
CsDof46	TATA-box	322	328	core promoter element around -30 of transcription start
CsDof46	TATA-box	323	328	core promoter element around -30 of transcription start
CsDof46	TATA-box	324	328	core promoter element around -30 of transcription start
CsDof46	TATA-box	370	378	core promoter element around -30 of transcription start
CsDof46	TATA-box	376	384	core promoter element around -30 of transcription start
CsDof46	TATA-box	378	386	core promoter element around -30 of transcription start
CsDof46	TATA-box	407	411	core promoter element around -30 of transcription start
CsDof46	TATA-box	446	453	core promoter element around -30 of transcription start
CsDof46	TATA-box	447	453	core promoter element around -30 of transcription start
CsDof46	TATA-box	448	453	core promoter element around -30 of transcription start
CsDof46	TATA-box	449	453	core promoter element around -30 of transcription start
CsDof46	TATA-box	489	496	core promoter element around -30 of transcription start
CsDof46	TATA-box	490	496	core promoter element around -30 of transcription start
CsDof46	TATA-box	491	496	core promoter element around -30 of transcription start
CsDof46	TATA-box	492	496	core promoter element around -30 of transcription start
CsDof46	TATA-box	609	616	core promoter element around -30 of transcription start
CsDof46	TATA-box	657	662	core promoter element around -30 of transcription start
CsDof46	TATA-box	658	662	core promoter element around -30 of transcription start
CsDof46	TATA-box	676	684	core promoter element around -30 of transcription start
CsDof46	TATA-box	700	704	core promoter element around -30 of transcription start
CsDof46	TATA-box	721	727	core promoter element around -30 of transcription start
CsDof46	TATA-box	722	727	core promoter element around -30 of transcription start
CsDof46	TATA-box	723	727	core promoter element around -30 of transcription start
CsDof46	TATA-box	901	910	core promoter element around -30 of transcription start
CsDof46	TATA-box	902	909	core promoter element around -30 of transcription start
CsDof46	TATA-box	903	909	core promoter element around -30 of transcription start
CsDof46	TATA-box	904	911	core promoter element around -30 of transcription start
CsDof46	TATA-box	905	911	core promoter element around -30 of transcription start
CsDof46	TATA-box	906	912	core promoter element around -30 of transcription start
CsDof46	TATA-box	907	911	core promoter element around -30 of transcription start
CsDof46	TATA-box	916	922	core promoter element around -30 of transcription start
CsDof46	TATA-box	917	924	core promoter element around -30 of transcription start
CsDof46	TATA-box	918	924	core promoter element around -30 of transcription start
CsDof46	TATA-box	919	925	core promoter element around -30 of transcription start
CsDof46	TATA-box	920	926	core promoter element around -30 of transcription start
CsDof46	TATA-box	921	927	core promoter element around -30 of transcription start
CsDof46	TATA-box	922	926	core promoter element around -30 of transcription start
CsDof46	TATA-box	1062	1069	core promoter element around -30 of transcription start
CsDof46	TATA-box	1063	1069	core promoter element around -30 of transcription start
CsDof46	TATA-box	1064	1069	core promoter element around -30 of transcription start
CsDof46	TATA-box	1065	1069	core promoter element around -30 of transcription start
CsDof46	TATA-box	1147	1153	core promoter element around -30 of transcription start
CsDof46	TATA-box	1148	1154	core promoter element around -30 of transcription start
CsDof46	TATA-box	1149	1155	core promoter element around -30 of transcription start
CsDof46	TATA-box	1150	1154	core promoter element around -30 of transcription start
CsDof46	TATA-box	1237	1244	core promoter element around -30 of transcription start
CsDof46	TATA-box	1238	1244	core promoter element around -30 of transcription start
CsDof46	TATA-box	1239	1244	core promoter element around -30 of transcription start
CsDof46	TATA-box	1240	1244	core promoter element around -30 of transcription start

Name	Cis-element	Start position	Stop position	Function
CsDof46	TATA-box	1268	1272	core promoter element around -30 of transcription start
CsDof46	TATA-box	1321	1327	core promoter element around -30 of transcription start
CsDof46	TATA-box	1322	1327	core promoter element around -30 of transcription start
CsDof46	TATA-box	1323	1327	core promoter element around -30 of transcription start
CsDof46	TATA-box	1542	1547	core promoter element around -30 of transcription start
CsDof46	TATA-box	1543	1547	core promoter element around -30 of transcription start
CsDof46	TATA-box	1554	1561	core promoter element around -30 of transcription start
CsDof46	TATA-box	1555	1561	core promoter element around -30 of transcription start
CsDof46	TATA-box	1556	1561	core promoter element around -30 of transcription start
CsDof46	TATA-box	1557	1561	core promoter element around -30 of transcription start
CsDof46	TATA-box	1717	1726	core promoter element around -30 of transcription start
CsDof46	TATA-box	1719	1723	core promoter element around -30 of transcription start
CsDof46	TATA-box	1957	1961	core promoter element around -30 of transcription start
CsDof46	GT1-motif	1010	1016	light responsive element
CsDof46	GT1-motif	1707	1713	light responsive element
CsDof46	GT1-motif	1785	1792	light responsive element
CsDof46	GT1-motif	1786	1792	light responsive element
CsDof46	4cl-CMA1b	201	213	light responsive element
CsDof46	CCAAT-box	1354	1360	MYBHv1 binding site
CsDof47	ABRE	1895	1901	abscisic acid responsiveness
CsDof47	ABRE	1896	1901	abscisic acid responsiveness
CsDof47	TC-rich repeats	89	98	cis-acting element involved in defense and stress responsiveness
CsDof47	TC-rich repeats	1115	1124	cis-acting element involved in defense and stress responsiveness
CsDof47	LTR	40	46	cis-acting element involved in low-temperature responsiveness
CsDof47	ARE	1090	1096	cis-acting regulatory element essential for the anaerobic induction
CsDof47	G-box	1403	1409	cis-acting regulatory element involved in light responsiveness
CsDof47	G-box	1894	1903	cis-acting regulatory element involved in light responsiveness
CsDof47	G-box	1895	1901	cis-acting regulatory element involved in light responsiveness
CsDof47	G-Box	1895	1901	cis-acting regulatory element involved in light responsiveness
CsDof47	RY-element	1943	1951	cis-acting regulatory element involved in seed-specific regulation
CsDof47	CGTCA-motif	887	892	cis-acting regulatory element involved in the MeJA-responsiveness
CsDof47	TGACG-motif	887	892	cis-acting regulatory element involved in the MeJA-responsiveness
CsDof47	O2-site	666	674	cis-acting regulatory element involved in zein metabolism regulation
CsDof47	CAT-box	455	461	cis-acting regulatory element related to meristem expression
CsDof47	CAAT-box	150	155	common cis-acting element in promoter and enhancer regions
CsDof47	CAAT-box	469	474	common cis-acting element in promoter and enhancer regions
CsDof47	CAAT-box	731	736	common cis-acting element in promoter and enhancer regions
CsDof47	CAAT-box	1301	1306	common cis-acting element in promoter and enhancer regions
CsDof47	CAAT-box	1479	1484	common cis-acting element in promoter and enhancer regions
CsDof47	CAAT-box	1521	1526	common cis-acting element in promoter and enhancer regions
CsDof47	CAAT-box	1641	1646	common cis-acting element in promoter and enhancer regions
CsDof47	CAAT-box	1660	1665	common cis-acting element in promoter and enhancer regions
CsDof47	CAAT-box	1860	1865	common cis-acting element in promoter and enhancer regions
CsDof47	TATA-box	34	40	core promoter element around -30 of transcription start
CsDof47	TATA-box	35	39	core promoter element around -30 of transcription start
CsDof47	TATA-box	128	134	core promoter element around -30 of transcription start
CsDof47	TATA-box	129	135	core promoter element around -30 of transcription start
CsDof47	TATA-box	131	135	core promoter element around -30 of transcription start
CsDof47	TATA-box	477	484	core promoter element around -30 of transcription start
CsDof47	TATA-box	490	496	core promoter element around -30 of transcription start
CsDof47	TATA-box	491	495	core promoter element around -30 of transcription start

Name	Cis-element	Start position	Stop position	Function
CsDof47	TATA-box	939	943	core promoter element around -30 of transcription start
CsDof47	TATA-box	1001	1006	core promoter element around -30 of transcription start
CsDof47	TATA-box	1002	1006	core promoter element around -30 of transcription start
CsDof47	TATA-box	1137	1146	core promoter element around -30 of transcription start
CsDof47	TATA-box	1138	1145	core promoter element around -30 of transcription start
CsDof47	TATA-box	1139	1145	core promoter element around -30 of transcription start
CsDof47	TATA-box	1140	1145	core promoter element around -30 of transcription start
CsDof47	TATA-box	1141	1145	core promoter element around -30 of transcription start
CsDof47	TATA-box	1297	1301	core promoter element around -30 of transcription start
CsDof47	TATA-box	1410	1416	core promoter element around -30 of transcription start
CsDof47	TATA-box	1411	1415	core promoter element around -30 of transcription start
CsDof47	TATA-box	1526	1532	core promoter element around -30 of transcription start
CsDof47	TATA-box	1527	1531	core promoter element around -30 of transcription start
CsDof47	TATA-box	1771	1775	core promoter element around -30 of transcription start
CsDof47	TATA-box	1779	1787	core promoter element around -30 of transcription start
CsDof47	TATA-box	1781	1789	core promoter element around -30 of transcription start
CsDof47	TATA-box	1953	1959	core promoter element around -30 of transcription start
CsDof47	TATA-box	1954	1960	core promoter element around -30 of transcription start
CsDof47	TATA-box	1955	1959	core promoter element around -30 of transcription start
CsDof47	P-box	390	397	gibberellin-responsive element
CsDof47	GT1-motif	1731	1738	light responsive element
CsDof47	GT1-motif	1732	1738	light responsive element
CsDof47	GT1-motif	1811	1817	light responsive element
CsDof47	CCAAT-box	73	79	MYBHv1 binding site
CsDof48	TGA-element	1267	1273	auxin-responsive element
CsDof48	TGA-element	1302	1308	auxin-responsive element
CsDof48	TC-rich repeats	1246	1255	cis-acting element involved in defense and stress responsiveness
CsDof48	ACE	190	199	cis-acting element involved in light responsiveness
CsDof48	ARE	774	780	cis-acting regulatory element essential for the anaerobic induction
CsDof48	ARE	1988	1994	cis-acting regulatory element essential for the anaerobic induction
CsDof48	AuxRR-core	303	310	cis-acting regulatory element involved in auxin responsiveness
CsDof48	TGACG-motif	889	894	cis-acting regulatory element involved in the MeJA-responsiveness
CsDof48	TGACG-motif	1636	1641	cis-acting regulatory element involved in the MeJA-responsiveness
CsDof48	CGTCA-motif	889	894	cis-acting regulatory element involved in the MeJA-responsiveness
CsDof48	CGTCA-motif	1636	1641	cis-acting regulatory element involved in the MeJA-responsiveness
CsDof48	CAAT-box	47	52	common cis-acting element in promoter and enhancer regions
CsDof48	CAAT-box	64	69	common cis-acting element in promoter and enhancer regions
CsDof48	CAAT-box	71	76	common cis-acting element in promoter and enhancer regions
CsDof48	CAAT-box	327	332	common cis-acting element in promoter and enhancer regions
CsDof48	CAAT-box	339	344	common cis-acting element in promoter and enhancer regions
CsDof48	CAAT-box	349	354	common cis-acting element in promoter and enhancer regions
CsDof48	CAAT-box	440	445	common cis-acting element in promoter and enhancer regions
CsDof48	CAAT-box	459	464	common cis-acting element in promoter and enhancer regions
CsDof48	CAAT-box	604	609	common cis-acting element in promoter and enhancer regions
CsDof48	CAAT-box	609	614	common cis-acting element in promoter and enhancer regions
CsDof48	CAAT-box	656	661	common cis-acting element in promoter and enhancer regions
CsDof48	CAAT-box	665	670	common cis-acting element in promoter and enhancer regions
CsDof48	CAAT-box	732	737	common cis-acting element in promoter and enhancer regions
CsDof48	CAAT-box	765	770	common cis-acting element in promoter and enhancer regions
CsDof48	CAAT-box	1010	1015	common cis-acting element in promoter and enhancer regions
CsDof48	CAAT-box	1023	1028	common cis-acting element in promoter and enhancer regions

Name	Cis-element	Start position	Stop position	Function
CsDof48	CAAT-box	1044	1049	common cis-acting element in promoter and enhancer regions
CsDof48	CAAT-box	1541	1546	common cis-acting element in promoter and enhancer regions
CsDof48	CAAT-box	1585	1590	common cis-acting element in promoter and enhancer regions
CsDof48	TATA-box	131	138	core promoter element around -30 of transcription start
CsDof48	TATA-box	132	138	core promoter element around -30 of transcription start
CsDof48	TATA-box	133	138	core promoter element around -30 of transcription start
CsDof48	TATA-box	134	138	core promoter element around -30 of transcription start
CsDof48	TATA-box	181	186	core promoter element around -30 of transcription start
CsDof48	TATA-box	182	186	core promoter element around -30 of transcription start
CsDof48	TATA-box	200	204	core promoter element around -30 of transcription start
CsDof48	TATA-box	239	248	core promoter element around -30 of transcription start
CsDof48	TATA-box	331	335	core promoter element around -30 of transcription start
CsDof48	TATA-box	467	471	core promoter element around -30 of transcription start
CsDof48	TATA-box	496	502	core promoter element around -30 of transcription start
CsDof48	TATA-box	497	501	core promoter element around -30 of transcription start
CsDof48	TATA-box	532	537	core promoter element around -30 of transcription start
CsDof48	TATA-box	533	537	core promoter element around -30 of transcription start
CsDof48	TATA-box	622	628	core promoter element around -30 of transcription start
CsDof48	TATA-box	623	628	core promoter element around -30 of transcription start
CsDof48	TATA-box	624	628	core promoter element around -30 of transcription start
CsDof48	TATA-box	676	682	core promoter element around -30 of transcription start
CsDof48	TATA-box	677	682	core promoter element around -30 of transcription start
CsDof48	TATA-box	678	682	core promoter element around -30 of transcription start
CsDof48	TATA-box	745	751	core promoter element around -30 of transcription start
CsDof48	TATA-box	746	750	core promoter element around -30 of transcription start
CsDof48	TATA-box	761	767	core promoter element around -30 of transcription start
CsDof48	TATA-box	762	766	core promoter element around -30 of transcription start
CsDof48	TATA-box	780	784	core promoter element around -30 of transcription start
CsDof48	TATA-box	836	840	core promoter element around -30 of transcription start
CsDof48	TATA-box	866	875	core promoter element around -30 of transcription start
CsDof48	TATA-box	867	874	core promoter element around -30 of transcription start
CsDof48	TATA-box	868	874	core promoter element around -30 of transcription start
CsDof48	TATA-box	869	874	core promoter element around -30 of transcription start
CsDof48	TATA-box	870	874	core promoter element around -30 of transcription start
CsDof48	TATA-box	898	904	core promoter element around -30 of transcription start
CsDof48	TATA-box	899	904	core promoter element around -30 of transcription start
CsDof48	TATA-box	900	904	core promoter element around -30 of transcription start
CsDof48	TATA-box	1059	1066	core promoter element around -30 of transcription start
CsDof48	TATA-box	1060	1066	core promoter element around -30 of transcription start
CsDof48	TATA-box	1061	1068	core promoter element around -30 of transcription start
CsDof48	TATA-box	1062	1068	core promoter element around -30 of transcription start
CsDof48	TATA-box	1063	1069	core promoter element around -30 of transcription start
CsDof48	TATA-box	1064	1070	core promoter element around -30 of transcription start
CsDof48	TATA-box	1065	1071	core promoter element around -30 of transcription start
CsDof48	TATA-box	1066	1070	core promoter element around -30 of transcription start
CsDof48	TATA-box	1256	1260	core promoter element around -30 of transcription start
CsDof48	TATA-box	1287	1293	core promoter element around -30 of transcription start
CsDof48	TATA-box	1288	1293	core promoter element around -30 of transcription start
CsDof48	TATA-box	1289	1293	core promoter element around -30 of transcription start
CsDof48	TATA-box	1322	1328	core promoter element around -30 of transcription start
CsDof48	TATA-box	1323	1327	core promoter element around -30 of transcription start

Name	Cis-element	Start position	Stop position	Function
CsDof48	TATA-box	1424	1428	core promoter element around -30 of transcription start
CsDof48	TATA-box	1439	1445	core promoter element around -30 of transcription start
CsDof48	TATA-box	1440	1445	core promoter element around -30 of transcription start
CsDof48	TATA-box	1441	1445	core promoter element around -30 of transcription start
CsDof48	TATA-box	1447	1453	core promoter element around -30 of transcription start
CsDof48	TATA-box	1448	1453	core promoter element around -30 of transcription start
CsDof48	TATA-box	1449	1453	core promoter element around -30 of transcription start
CsDof48	TATA-box	1458	1464	core promoter element around -30 of transcription start
CsDof48	TATA-box	1460	1466	core promoter element around -30 of transcription start
CsDof48	TATA-box	1461	1467	core promoter element around -30 of transcription start
CsDof48	TATA-box	1462	1468	core promoter element around -30 of transcription start
CsDof48	TATA-box	1463	1469	core promoter element around -30 of transcription start
CsDof48	TATA-box	1464	1470	core promoter element around -30 of transcription start
CsDof48	TATA-box	1465	1471	core promoter element around -30 of transcription start
CsDof48	TATA-box	1466	1472	core promoter element around -30 of transcription start
CsDof48	TATA-box	1467	1473	core promoter element around -30 of transcription start
CsDof48	TATA-box	1468	1474	core promoter element around -30 of transcription start
CsDof48	TATA-box	1469	1475	core promoter element around -30 of transcription start
CsDof48	TATA-box	1470	1476	core promoter element around -30 of transcription start
CsDof48	TATA-box	1471	1477	core promoter element around -30 of transcription start
CsDof48	TATA-box	1472	1478	core promoter element around -30 of transcription start
CsDof48	TATA-box	1473	1479	core promoter element around -30 of transcription start
CsDof48	TATA-box	1474	1480	core promoter element around -30 of transcription start
CsDof48	TATA-box	1475	1481	core promoter element around -30 of transcription start
CsDof48	TATA-box	1476	1482	core promoter element around -30 of transcription start
CsDof48	TATA-box	1477	1483	core promoter element around -30 of transcription start
CsDof48	TATA-box	1478	1482	core promoter element around -30 of transcription start
CsDof48	TATA-box	1513	1519	core promoter element around -30 of transcription start
CsDof48	TATA-box	1514	1518	core promoter element around -30 of transcription start
CsDof48	TATA-box	1520	1526	core promoter element around -30 of transcription start
CsDof48	TATA-box	1521	1526	core promoter element around -30 of transcription start
CsDof48	TATA-box	1522	1526	core promoter element around -30 of transcription start
CsDof48	TATA-box	1547	1552	core promoter element around -30 of transcription start
CsDof48	TATA-box	1548	1552	core promoter element around -30 of transcription start
CsDof48	TATA-box	1575	1581	core promoter element around -30 of transcription start
CsDof48	TATA-box	1576	1580	core promoter element around -30 of transcription start
CsDof48	TATA-box	1598	1607	core promoter element around -30 of transcription start
CsDof48	TATA-box	1599	1605	core promoter element around -30 of transcription start
CsDof48	TATA-box	1600	1606	core promoter element around -30 of transcription start
CsDof48	TATA-box	1601	1607	core promoter element around -30 of transcription start
CsDof48	TATA-box	1602	1608	core promoter element around -30 of transcription start
CsDof48	TATA-box	1603	1609	core promoter element around -30 of transcription start
CsDof48	TATA-box	1604	1610	core promoter element around -30 of transcription start
CsDof48	TATA-box	1606	1610	core promoter element around -30 of transcription start
CsDof48	TATA-box	1971	1980	core promoter element around -30 of transcription start
CsDof48	GT1-motif	1712	1718	light responsive element
CsDof48	Box III	1551	1562	protein binding site
CsDof49	ABRE	584	593	abscisic acid responsiveness
CsDof49	ABRE	586	592	abscisic acid responsiveness
CsDof49	ABRE	587	592	abscisic acid responsiveness
CsDof49	ABRE	836	844	abscisic acid responsiveness

Name	Cis-element	Start position	Stop position	Function
CsDof49	ABRE	838	843	abscisic acid responsiveness
CsDof49	ABRE	1268	1273	abscisic acid responsiveness
CsDof49	TGA-element	591	597	auxin-responsive element
CsDof49	LTR	944	950	cis-acting element involved in low-temperature responsiveness
CsDof49	TCA-element	318	327	cis-acting element involved in salicylic acid responsiveness
CsDof49	TCA-element	864	873	cis-acting element involved in salicylic acid responsiveness
CsDof49	TCA-element	1895	1904	cis-acting element involved in salicylic acid responsiveness
CsDof49	A-box	409	415	cis-acting regulatory element
CsDof49	A-box	1422	1428	cis-acting regulatory element
CsDof49	ARE	1686	1692	cis-acting regulatory element essential for the anaerobic induction
CsDof49	G-box	585	593	cis-acting regulatory element involved in light responsiveness
CsDof49	G-box	586	592	cis-acting regulatory element involved in light responsiveness
CsDof49	G-box	838	844	cis-acting regulatory element involved in light responsiveness
CsDof49	G-Box	586	592	cis-acting regulatory element involved in light responsiveness
CsDof49	G-Box	1268	1274	cis-acting regulatory element involved in light responsiveness
CsDof49	TGACG-motif	183	188	cis-acting regulatory element involved in the MeJA-responsiveness
CsDof49	TGACG-motif	670	675	cis-acting regulatory element involved in the MeJA-responsiveness
CsDof49	TGACG-motif	708	713	cis-acting regulatory element involved in the MeJA-responsiveness
CsDof49	TGACG-motif	1179	1184	cis-acting regulatory element involved in the MeJA-responsiveness
CsDof49	TGACG-motif	1182	1187	cis-acting regulatory element involved in the MeJA-responsiveness
CsDof49	CGTCA-motif	183	188	cis-acting regulatory element involved in the MeJA-responsiveness
CsDof49	CGTCA-motif	670	675	cis-acting regulatory element involved in the MeJA-responsiveness
CsDof49	CGTCA-motif	708	713	cis-acting regulatory element involved in the MeJA-responsiveness
CsDof49	CGTCA-motif	1179	1184	cis-acting regulatory element involved in the MeJA-responsiveness
CsDof49	CGTCA-motif	1182	1187	cis-acting regulatory element involved in the MeJA-responsiveness
CsDof49	CAT-box	1143	1149	cis-acting regulatory element related to meristem expression
CsDof49	CAAT-box	149	154	common cis-acting element in promoter and enhancer regions
CsDof49	CAAT-box	168	173	common cis-acting element in promoter and enhancer regions
CsDof49	CAAT-box	429	434	common cis-acting element in promoter and enhancer regions
CsDof49	CAAT-box	569	574	common cis-acting element in promoter and enhancer regions
CsDof49	CAAT-box	612	617	common cis-acting element in promoter and enhancer regions
CsDof49	CAAT-box	664	669	common cis-acting element in promoter and enhancer regions
CsDof49	CAAT-box	986	991	common cis-acting element in promoter and enhancer regions
CsDof49	CAAT-box	1131	1136	common cis-acting element in promoter and enhancer regions
CsDof49	CAAT-box	1251	1256	common cis-acting element in promoter and enhancer regions
CsDof49	CAAT-box	1631	1636	common cis-acting element in promoter and enhancer regions
CsDof49	CAAT-box	1799	1804	common cis-acting element in promoter and enhancer regions
CsDof49	CAAT-box	1982	1992	common cis-acting element in promoter and enhancer regions
CsDof49	TATA-box	45	49	core promoter element around -30 of transcription start
CsDof49	TATA-box	114	118	core promoter element around -30 of transcription start
CsDof49	TATA-box	119	125	core promoter element around -30 of transcription start
CsDof49	TATA-box	120	126	core promoter element around -30 of transcription start
CsDof49	TATA-box	121	125	core promoter element around -30 of transcription start
CsDof49	TATA-box	144	150	core promoter element around -30 of transcription start
CsDof49	TATA-box	145	150	core promoter element around -30 of transcription start
CsDof49	TATA-box	146	150	core promoter element around -30 of transcription start
CsDof49	TATA-box	333	337	core promoter element around -30 of transcription start
CsDof49	TATA-box	625	631	core promoter element around -30 of transcription start
CsDof49	TATA-box	627	633	core promoter element around -30 of transcription start
CsDof49	TATA-box	628	634	core promoter element around -30 of transcription start
CsDof49	TATA-box	629	633	core promoter element around -30 of transcription start

Name	Cis-element	Start position	Stop position	Function
CsDof49	TATA-box	994	998	core promoter element around -30 of transcription start
CsDof49	TATA-box	1008	1014	core promoter element around -30 of transcription start
CsDof49	TATA-box	1009	1015	core promoter element around -30 of transcription start
CsDof49	TATA-box	1011	1015	core promoter element around -30 of transcription start
CsDof49	TATA-box	1063	1068	core promoter element around -30 of transcription start
CsDof49	TATA-box	1064	1068	core promoter element around -30 of transcription start
CsDof49	TATA-box	1214	1222	core promoter element around -30 of transcription start
CsDof49	TATA-box	1217	1224	core promoter element around -30 of transcription start
CsDof49	TATA-box	1218	1224	core promoter element around -30 of transcription start
CsDof49	TATA-box	1219	1225	core promoter element around -30 of transcription start
CsDof49	TATA-box	1220	1224	core promoter element around -30 of transcription start
CsDof49	TATA-box	1257	1263	core promoter element around -30 of transcription start
CsDof49	TATA-box	1258	1264	core promoter element around -30 of transcription start
CsDof49	TATA-box	1259	1265	core promoter element around -30 of transcription start
CsDof49	TATA-box	1260	1264	core promoter element around -30 of transcription start
CsDof49	TATA-box	1283	1287	core promoter element around -30 of transcription start
CsDof49	TATA-box	1322	1328	core promoter element around -30 of transcription start
CsDof49	TATA-box	1323	1328	core promoter element around -30 of transcription start
CsDof49	TATA-box	1324	1328	core promoter element around -30 of transcription start
CsDof49	TATA-box	1576	1582	core promoter element around -30 of transcription start
CsDof49	TATA-box	1578	1582	core promoter element around -30 of transcription start
CsDof49	TATA-box	1600	1612	core promoter element around -30 of transcription start
CsDof49	TATA-box	1603	1607	core promoter element around -30 of transcription start
CsDof49	TATA-box	1608	1614	core promoter element around -30 of transcription start
CsDof49	TATA-box	1609	1613	core promoter element around -30 of transcription start
CsDof49	TATA-box	1636	1645	core promoter element around -30 of transcription start
CsDof49	TATA-box	1637	1644	core promoter element around -30 of transcription start
CsDof49	TATA-box	1638	1644	core promoter element around -30 of transcription start
CsDof49	TATA-box	1639	1646	core promoter element around -30 of transcription start
CsDof49	TATA-box	1640	1646	core promoter element around -30 of transcription start
CsDof49	TATA-box	1641	1647	core promoter element around -30 of transcription start
CsDof49	TATA-box	1642	1646	core promoter element around -30 of transcription start
CsDof49	TATA-box	1839	1845	core promoter element around -30 of transcription start
CsDof49	TATA-box	1840	1846	core promoter element around -30 of transcription start
CsDof49	TATA-box	1841	1847	core promoter element around -30 of transcription start
CsDof49	TATA-box	1842	1848	core promoter element around -30 of transcription start
CsDof49	TATA-box	1843	1849	core promoter element around -30 of transcription start
CsDof49	TATA-box	1845	1849	core promoter element around -30 of transcription start
CsDof49	GC-motif	1428	1434	enhancer-like element involved in anoxic specific inducibility
CsDof49	P-box	1980	1992	gibberellin-responsive element and part of a light responsive element
CsDof49	GT1-motif	1090	1097	light responsive element
CsDof49	CCAAT-box	734	740	MYBHv1 binding site
CsDof50	ABRE	7	14	abscisic acid responsiveness
CsDof50	AT-rich element	1934	1944	binding site of AT-rich DNA binding protein (ATBP-1)
CsDof50	ARE	257	263	cis-acting regulatory element essential for the anaerobic induction
CsDof50	ARE	746	752	cis-acting regulatory element essential for the anaerobic induction
CsDof50	TGACG-motif	630	635	cis-acting regulatory element involved in the MeJA-responsiveness
CsDof50	TGACG-motif	1899	1904	cis-acting regulatory element involved in the MeJA-responsiveness
CsDof50	CGTCA-motif	630	635	cis-acting regulatory element involved in the MeJA-responsiveness
CsDof50	CGTCA-motif	1899	1904	cis-acting regulatory element involved in the MeJA-responsiveness
CsDof50	O2-site	427	436	cis-acting regulatory element involved in zein metabolism regulation

Name	Cis-element	Start position	Stop position	Function
CsDof50	CAT-box	308	314	cis-acting regulatory element related to meristem expression
CsDof50	CAAT-box	65	70	common cis-acting element in promoter and enhancer regions
CsDof50	CAAT-box	352	357	common cis-acting element in promoter and enhancer regions
CsDof50	CAAT-box	397	402	common cis-acting element in promoter and enhancer regions
CsDof50	CAAT-box	645	650	common cis-acting element in promoter and enhancer regions
CsDof50	CAAT-box	660	667	common cis-acting element in promoter and enhancer regions
CsDof50	CAAT-box	1075	1080	common cis-acting element in promoter and enhancer regions
CsDof50	CAAT-box	1197	1202	common cis-acting element in promoter and enhancer regions
CsDof50	CAAT-box	1399	1404	common cis-acting element in promoter and enhancer regions
CsDof50	CAAT-box	1444	1449	common cis-acting element in promoter and enhancer regions
CsDof50	CAAT-box	1465	1470	common cis-acting element in promoter and enhancer regions
CsDof50	CAAT-box	1624	1629	common cis-acting element in promoter and enhancer regions
CsDof50	CAAT-box	1950	1955	common cis-acting element in promoter and enhancer regions
CsDof50	TATA-box	212	216	core promoter element around -30 of transcription start
CsDof50	TATA-box	349	353	core promoter element around -30 of transcription start
CsDof50	TATA-box	375	379	core promoter element around -30 of transcription start
CsDof50	TATA-box	468	475	core promoter element around -30 of transcription start
CsDof50	TATA-box	471	477	core promoter element around -30 of transcription start
CsDof50	TATA-box	473	477	core promoter element around -30 of transcription start
CsDof50	TATA-box	686	693	core promoter element around -30 of transcription start
CsDof50	TATA-box	687	693	core promoter element around -30 of transcription start
CsDof50	TATA-box	688	693	core promoter element around -30 of transcription start
CsDof50	TATA-box	689	693	core promoter element around -30 of transcription start
CsDof50	TATA-box	836	842	core promoter element around -30 of transcription start
CsDof50	TATA-box	837	842	core promoter element around -30 of transcription start
CsDof50	TATA-box	838	842	core promoter element around -30 of transcription start
CsDof50	TATA-box	849	853	core promoter element around -30 of transcription start
CsDof50	TATA-box	892	898	core promoter element around -30 of transcription start
CsDof50	TATA-box	893	899	core promoter element around -30 of transcription start
CsDof50	TATA-box	894	898	core promoter element around -30 of transcription start
CsDof50	TATA-box	903	912	core promoter element around -30 of transcription start
CsDof50	TATA-box	909	913	core promoter element around -30 of transcription start
CsDof50	TATA-box	1330	1336	core promoter element around -30 of transcription start
CsDof50	TATA-box	1331	1335	core promoter element around -30 of transcription start
CsDof50	TATA-box	1338	1344	core promoter element around -30 of transcription start
CsDof50	TATA-box	1339	1343	core promoter element around -30 of transcription start
CsDof50	TATA-box	1348	1354	core promoter element around -30 of transcription start
CsDof50	TATA-box	1349	1355	core promoter element around -30 of transcription start
CsDof50	TATA-box	1350	1356	core promoter element around -30 of transcription start
CsDof50	TATA-box	1351	1357	core promoter element around -30 of transcription start
CsDof50	TATA-box	1352	1358	core promoter element around -30 of transcription start
CsDof50	TATA-box	1353	1359	core promoter element around -30 of transcription start
CsDof50	TATA-box	1354	1360	core promoter element around -30 of transcription start
CsDof50	TATA-box	1355	1361	core promoter element around -30 of transcription start
CsDof50	TATA-box	1356	1362	core promoter element around -30 of transcription start
CsDof50	TATA-box	1357	1361	core promoter element around -30 of transcription start
CsDof50	TATA-box	1510	1516	core promoter element around -30 of transcription start
CsDof50	TATA-box	1511	1517	core promoter element around -30 of transcription start
CsDof50	TATA-box	1512	1518	core promoter element around -30 of transcription start
CsDof50	TATA-box	1513	1517	core promoter element around -30 of transcription start
CsDof50	TATA-box	1547	1553	core promoter element around -30 of transcription start

Name	Cis-element	Start position	Stop position	Function
CsDof50	TATA-box	1549	1553	core promoter element around -30 of transcription start
CsDof50	TATA-box	1667	1671	core promoter element around -30 of transcription start
CsDof50	TATA-box	1736	1743	core promoter element around -30 of transcription start
CsDof50	TATA-box	1737	1743	core promoter element around -30 of transcription start
CsDof50	TATA-box	1738	1744	core promoter element around -30 of transcription start
CsDof50	TATA-box	1739	1743	core promoter element around -30 of transcription start
CsDof50	TATA-box	1762	1769	core promoter element around -30 of transcription start
CsDof50	TATA-box	1763	1769	core promoter element around -30 of transcription start
CsDof50	TATA-box	1764	1769	core promoter element around -30 of transcription start
CsDof50	TATA-box	1765	1769	core promoter element around -30 of transcription start
CsDof50	TATA-box	1808	1812	core promoter element around -30 of transcription start
CsDof50	TATA-box	1840	1844	core promoter element around -30 of transcription start
CsDof50	TATA-box	1849	1853	core promoter element around -30 of transcription start
CsDof50	TATA-box	1873	1879	core promoter element around -30 of transcription start
CsDof50	TATA-box	1874	1880	core promoter element around -30 of transcription start
CsDof50	TATA-box	1875	1881	core promoter element around -30 of transcription start
CsDof50	TATA-box	1876	1882	core promoter element around -30 of transcription start
CsDof50	TATA-box	1877	1883	core promoter element around -30 of transcription start
CsDof50	TATA-box	1878	1884	core promoter element around -30 of transcription start
CsDof50	TATA-box	1879	1885	core promoter element around -30 of transcription start
CsDof50	TATA-box	1880	1892	core promoter element around -30 of transcription start
CsDof50	TATA-box	1881	1887	core promoter element around -30 of transcription start
CsDof50	TATA-box	1882	1888	core promoter element around -30 of transcription start
CsDof50	TATA-box	1883	1889	core promoter element around -30 of transcription start
CsDof50	TATA-box	1884	1890	core promoter element around -30 of transcription start
CsDof50	TATA-box	1885	1891	core promoter element around -30 of transcription start
CsDof50	TATA-box	1886	1890	core promoter element around -30 of transcription start
CsDof50	TATA-box	1890	1897	core promoter element around -30 of transcription start
CsDof50	TATA-box	1891	1897	core promoter element around -30 of transcription start
CsDof50	TATA-box	1892	1899	core promoter element around -30 of transcription start
CsDof50	TATA-box	1893	1899	core promoter element around -30 of transcription start
CsDof50	TATA-box	1895	1899	core promoter element around -30 of transcription start
CsDof50	TATA-box	1912	1918	core promoter element around -30 of transcription start
CsDof50	TATA-box	1913	1919	core promoter element around -30 of transcription start
CsDof50	TATA-box	1914	1920	core promoter element around -30 of transcription start
CsDof50	TATA-box	1915	1919	core promoter element around -30 of transcription start
CsDof51	ABRE	522	527	abscisic acid responsiveness
CsDof51	TGA-element	1250	1256	auxin-responsive element
CsDof51	TC-rich repeats	1341	1350	cis-acting element involved in defense and stress responsiveness
CsDof51	ACE	431	440	cis-acting element involved in light responsiveness
CsDof51	TCA-element	1912	1921	cis-acting element involved in salicylic acid responsiveness
CsDof51	G-box	521	527	cis-acting regulatory element involved in light responsiveness
CsDof51	RY-element	348	356	cis-acting regulatory element involved in seed-specific regulation
CsDof51	CGTCA-motif	1370	1375	cis-acting regulatory element involved in the MeJA-responsiveness
CsDof51	TGACG-motif	1370	1375	cis-acting regulatory element involved in the MeJA-responsiveness
CsDof51	CAT-box	684	690	cis-acting regulatory element related to meristem expression
CsDof51	GCN4_motif	1221	1228	cis-regulatory element involved in endosperm expression
CsDof51	CAAT-box	76	81	common cis-acting element in promoter and enhancer regions
CsDof51	CAAT-box	406	411	common cis-acting element in promoter and enhancer regions
CsDof51	CAAT-box	409	414	common cis-acting element in promoter and enhancer regions
CsDof51	CAAT-box	451	456	common cis-acting element in promoter and enhancer regions

Name	Cis-element	Start position	Stop position	Function
CsDof51	CAAT-box	743	748	common cis-acting element in promoter and enhancer regions
CsDof51	CAAT-box	769	774	common cis-acting element in promoter and enhancer regions
CsDof51	CAAT-box	774	779	common cis-acting element in promoter and enhancer regions
CsDof51	CAAT-box	866	871	common cis-acting element in promoter and enhancer regions
CsDof51	CAAT-box	913	918	common cis-acting element in promoter and enhancer regions
CsDof51	CAAT-box	916	921	common cis-acting element in promoter and enhancer regions
CsDof51	CAAT-box	1044	1049	common cis-acting element in promoter and enhancer regions
CsDof51	CAAT-box	1255	1260	common cis-acting element in promoter and enhancer regions
CsDof51	CAAT-box	1282	1287	common cis-acting element in promoter and enhancer regions
CsDof51	CAAT-box	1516	1521	common cis-acting element in promoter and enhancer regions
CsDof51	CAAT-box	1619	1624	common cis-acting element in promoter and enhancer regions
CsDof51	CAAT-box	1695	1700	common cis-acting element in promoter and enhancer regions
CsDof51	CAAT-box	1761	1766	common cis-acting element in promoter and enhancer regions
CsDof51	CAAT-box	1810	1815	common cis-acting element in promoter and enhancer regions
CsDof51	CAAT-box	1873	1878	common cis-acting element in promoter and enhancer regions
CsDof51	CAAT-box	1932	1937	common cis-acting element in promoter and enhancer regions
CsDof51	TATA-box	7	11	core promoter element around -30 of transcription start
CsDof51	TATA-box	12	18	core promoter element around -30 of transcription start
CsDof51	TATA-box	13	18	core promoter element around -30 of transcription start
CsDof51	TATA-box	14	18	core promoter element around -30 of transcription start
CsDof51	TATA-box	33	38	core promoter element around -30 of transcription start
CsDof51	TATA-box	34	38	core promoter element around -30 of transcription start
CsDof51	TATA-box	39	47	core promoter element around -30 of transcription start
CsDof51	TATA-box	84	90	core promoter element around -30 of transcription start
CsDof51	TATA-box	85	92	core promoter element around -30 of transcription start
CsDof51	TATA-box	86	92	core promoter element around -30 of transcription start
CsDof51	TATA-box	87	93	core promoter element around -30 of transcription start
CsDof51	TATA-box	88	92	core promoter element around -30 of transcription start
CsDof51	TATA-box	187	194	core promoter element around -30 of transcription start
CsDof51	TATA-box	210	216	core promoter element around -30 of transcription start
CsDof51	TATA-box	212	216	core promoter element around -30 of transcription start
CsDof51	TATA-box	222	228	core promoter element around -30 of transcription start
CsDof51	TATA-box	223	227	core promoter element around -30 of transcription start
CsDof51	TATA-box	244	249	core promoter element around -30 of transcription start
CsDof51	TATA-box	245	249	core promoter element around -30 of transcription start
CsDof51	TATA-box	270	276	core promoter element around -30 of transcription start
CsDof51	TATA-box	271	275	core promoter element around -30 of transcription start
CsDof51	TATA-box	323	329	core promoter element around -30 of transcription start
CsDof51	TATA-box	324	330	core promoter element around -30 of transcription start
CsDof51	TATA-box	326	330	core promoter element around -30 of transcription start
CsDof51	TATA-box	399	405	core promoter element around -30 of transcription start
CsDof51	TATA-box	400	405	core promoter element around -30 of transcription start
CsDof51	TATA-box	401	405	core promoter element around -30 of transcription start
CsDof51	TATA-box	500	506	core promoter element around -30 of transcription start
CsDof51	TATA-box	501	507	core promoter element around -30 of transcription start
CsDof51	TATA-box	503	507	core promoter element around -30 of transcription start
CsDof51	TATA-box	564	570	core promoter element around -30 of transcription start
CsDof51	TATA-box	565	569	core promoter element around -30 of transcription start
CsDof51	TATA-box	668	674	core promoter element around -30 of transcription start
CsDof51	TATA-box	669	673	core promoter element around -30 of transcription start
CsDof51	TATA-box	797	803	core promoter element around -30 of transcription start

Name	Cis-element	Start position	Stop position	Function
CsDof51	TATA-box	798	804	core promoter element around -30 of transcription start
CsDof51	TATA-box	799	805	core promoter element around -30 of transcription start
CsDof51	TATA-box	800	806	core promoter element around -30 of transcription start
CsDof51	TATA-box	801	807	core promoter element around -30 of transcription start
CsDof51	TATA-box	802	808	core promoter element around -30 of transcription start
CsDof51	TATA-box	803	809	core promoter element around -30 of transcription start
CsDof51	TATA-box	804	810	core promoter element around -30 of transcription start
CsDof51	TATA-box	805	811	core promoter element around -30 of transcription start
CsDof51	TATA-box	807	811	core promoter element around -30 of transcription start
CsDof51	TATA-box	952	958	core promoter element around -30 of transcription start
CsDof51	TATA-box	953	957	core promoter element around -30 of transcription start
CsDof51	TATA-box	1003	1009	core promoter element around -30 of transcription start
CsDof51	TATA-box	1005	1009	core promoter element around -30 of transcription start
CsDof51	TATA-box	1301	1309	core promoter element around -30 of transcription start
CsDof51	TATA-box	1310	1314	core promoter element around -30 of transcription start
CsDof51	TATA-box	1433	1437	core promoter element around -30 of transcription start
CsDof51	TATA-box	1549	1553	core promoter element around -30 of transcription start
CsDof51	TATA-box	1622	1628	core promoter element around -30 of transcription start
CsDof51	TATA-box	1623	1628	core promoter element around -30 of transcription start
CsDof51	TATA-box	1624	1628	core promoter element around -30 of transcription start
CsDof51	TATA-box	1637	1643	core promoter element around -30 of transcription start
CsDof51	TATA-box	1638	1644	core promoter element around -30 of transcription start
CsDof51	TATA-box	1639	1645	core promoter element around -30 of transcription start
CsDof51	TATA-box	1640	1644	core promoter element around -30 of transcription start
CsDof51	TATA-box	1644	1650	core promoter element around -30 of transcription start
CsDof51	TATA-box	1645	1649	core promoter element around -30 of transcription start
CsDof51	TATA-box	1666	1671	core promoter element around -30 of transcription start
CsDof51	TATA-box	1667	1671	core promoter element around -30 of transcription start
CsDof51	TATA-box	1749	1754	core promoter element around -30 of transcription start
CsDof51	TATA-box	1750	1754	core promoter element around -30 of transcription start
CsDof51	TATA-box	1753	1759	core promoter element around -30 of transcription start
CsDof51	TATA-box	1754	1759	core promoter element around -30 of transcription start
CsDof51	TATA-box	1755	1759	core promoter element around -30 of transcription start
CsDof51	TATA-box	1898	1905	core promoter element around -30 of transcription start
CsDof51	GT1-motif	649	655	light responsive element
CsDof51	MBS	56	62	MYB binding site involved in drought-inducibility
CsDof51	WUN-motif	780	789	wound-responsive element
CsDof52	ABRE	392	397	abscisic acid responsiveness
CsDof52	ABRE	586	591	abscisic acid responsiveness
CsDof52	ABRE	1411	1416	abscisic acid responsiveness
CsDof52	ABRE	1853	1863	abscisic acid responsiveness
CsDof52	ABRE	1855	1860	abscisic acid responsiveness
CsDof52	TC-rich repeats	1557	1566	cis-acting element involved in defense and stress responsiveness
CsDof52	LTR	863	869	cis-acting element involved in low-temperature responsiveness
CsDof52	TCA-element	134	143	cis-acting element involved in salicylic acid responsiveness
CsDof52	ARE	283	289	cis-acting regulatory element essential for the anaerobic induction
CsDof52	ARE	416	422	cis-acting regulatory element essential for the anaerobic induction
CsDof52	ARE	630	636	cis-acting regulatory element essential for the anaerobic induction
CsDof52	ARE	721	727	cis-acting regulatory element essential for the anaerobic induction
CsDof52	ARE	852	858	cis-acting regulatory element essential for the anaerobic induction
CsDof52	circadian	121	130	cis-acting regulatory element involved in circadian control

Name	Cis-element	Start position	Stop position	Function
CsDof52	G-box	391	397	cis-acting regulatory element involved in light responsiveness
CsDof52	G-box	1411	1417	cis-acting regulatory element involved in light responsiveness
CsDof52	G-Box	585	591	cis-acting regulatory element involved in light responsiveness
CsDof52	G-Box	1855	1861	cis-acting regulatory element involved in light responsiveness
CsDof52	RY-element	86	94	cis-acting regulatory element involved in seed-specific regulation
CsDof52	TGACG-motif	1196	1201	cis-acting regulatory element involved in the MeJA-responsiveness
CsDof52	TGACG-motif	1392	1397	cis-acting regulatory element involved in the MeJA-responsiveness
CsDof52	CGTCA-motif	1196	1201	cis-acting regulatory element involved in the MeJA-responsiveness
CsDof52	CGTCA-motif	1392	1397	cis-acting regulatory element involved in the MeJA-responsiveness
CsDof52	O2-site	397	406	cis-acting regulatory element involved in zein metabolism regulation
CsDof52	CAT-box	1120	1126	cis-acting regulatory element related to meristem expression
CsDof52	CAT-box	1191	1197	cis-acting regulatory element related to meristem expression
CsDof52	CAAT-box	44	49	common cis-acting element in promoter and enhancer regions
CsDof52	CAAT-box	169	174	common cis-acting element in promoter and enhancer regions
CsDof52	CAAT-box	314	319	common cis-acting element in promoter and enhancer regions
CsDof52	CAAT-box	326	331	common cis-acting element in promoter and enhancer regions
CsDof52	CAAT-box	476	481	common cis-acting element in promoter and enhancer regions
CsDof52	CAAT-box	656	661	common cis-acting element in promoter and enhancer regions
CsDof52	CAAT-box	699	704	common cis-acting element in promoter and enhancer regions
CsDof52	CAAT-box	908	913	common cis-acting element in promoter and enhancer regions
CsDof52	CAAT-box	1165	1170	common cis-acting element in promoter and enhancer regions
CsDof52	CAAT-box	1178	1183	common cis-acting element in promoter and enhancer regions
CsDof52	CAAT-box	1275	1280	common cis-acting element in promoter and enhancer regions
CsDof52	CAAT-box	1330	1335	common cis-acting element in promoter and enhancer regions
CsDof52	CAAT-box	1378	1383	common cis-acting element in promoter and enhancer regions
CsDof52	CAAT-box	1649	1654	common cis-acting element in promoter and enhancer regions
CsDof52	TATA-box	291	297	core promoter element around -30 of transcription start
CsDof52	TATA-box	292	298	core promoter element around -30 of transcription start
CsDof52	TATA-box	294	298	core promoter element around -30 of transcription start
CsDof52	TATA-box	685	692	core promoter element around -30 of transcription start
CsDof52	TATA-box	686	692	core promoter element around -30 of transcription start
CsDof52	TATA-box	688	692	core promoter element around -30 of transcription start
CsDof52	TATA-box	880	888	core promoter element around -30 of transcription start
CsDof52	TATA-box	903	908	core promoter element around -30 of transcription start
CsDof52	TATA-box	904	908	core promoter element around -30 of transcription start
CsDof52	TATA-box	1173	1179	core promoter element around -30 of transcription start
CsDof52	TATA-box	1174	1179	core promoter element around -30 of transcription start
CsDof52	TATA-box	1175	1179	core promoter element around -30 of transcription start
CsDof52	TATA-box	1247	1251	core promoter element around -30 of transcription start
CsDof52	TATA-box	1295	1301	core promoter element around -30 of transcription start
CsDof52	TATA-box	1296	1302	core promoter element around -30 of transcription start
CsDof52	TATA-box	1297	1303	core promoter element around -30 of transcription start
CsDof52	TATA-box	1298	1302	core promoter element around -30 of transcription start
CsDof52	TATA-box	1319	1326	core promoter element around -30 of transcription start
CsDof52	TATA-box	1320	1326	core promoter element around -30 of transcription start
CsDof52	TATA-box	1321	1326	core promoter element around -30 of transcription start
CsDof52	TATA-box	1322	1326	core promoter element around -30 of transcription start
CsDof52	TATA-box	1337	1345	core promoter element around -30 of transcription start
CsDof52	TATA-box	1416	1422	core promoter element around -30 of transcription start
CsDof52	TATA-box	1417	1422	core promoter element around -30 of transcription start
CsDof52	TATA-box	1418	1422	core promoter element around -30 of transcription start

Name	Cis-element	Start position	Stop position	Function
CsDof52	TATA-box	1429	1433	core promoter element around -30 of transcription start
CsDof52	TATA-box	1545	1554	core promoter element around -30 of transcription start
CsDof52	TATA-box	1547	1551	core promoter element around -30 of transcription start
CsDof52	TATA-box	1620	1625	core promoter element around -30 of transcription start
CsDof52	TATA-box	1621	1625	core promoter element around -30 of transcription start
CsDof52	TATA-box	1683	1690	core promoter element around -30 of transcription start
CsDof52	TATA-box	1686	1692	core promoter element around -30 of transcription start
CsDof52	TATA-box	1688	1692	core promoter element around -30 of transcription start
CsDof52	TATA-box	1694	1700	core promoter element around -30 of transcription start
CsDof52	TATA-box	1695	1700	core promoter element around -30 of transcription start
CsDof52	TATA-box	1696	1700	core promoter element around -30 of transcription start
CsDof52	TATA-box	1715	1721	core promoter element around -30 of transcription start
CsDof52	TATA-box	1716	1723	core promoter element around -30 of transcription start
CsDof52	TATA-box	1717	1723	core promoter element around -30 of transcription start
CsDof52	TATA-box	1718	1724	core promoter element around -30 of transcription start
CsDof52	TATA-box	1719	1725	core promoter element around -30 of transcription start
CsDof52	TATA-box	1720	1726	core promoter element around -30 of transcription start
CsDof52	TATA-box	1721	1725	core promoter element around -30 of transcription start
CsDof52	TATA-box	1745	1749	core promoter element around -30 of transcription start
CsDof52	TATA-box	1903	1911	core promoter element around -30 of transcription start
CsDof52	TATA-box	1906	1913	core promoter element around -30 of transcription start
CsDof52	TATA-box	1907	1913	core promoter element around -30 of transcription start
CsDof52	TATA-box	1908	1914	core promoter element around -30 of transcription start
CsDof52	TATA-box	1909	1913	core promoter element around -30 of transcription start
CsDof52	TATA-box	1960	1965	core promoter element around -30 of transcription start
CsDof52	TATA-box	1961	1965	core promoter element around -30 of transcription start
CsDof52	GARE-motif	536	543	gibberellin-responsive element
CsDof52	GT1-motif	575	581	light responsive element
CsDof52	MBS	1056	1062	MYB binding site involved in drought-inducibility
CsDof52	CCAAT-box	1132	1138	MYBHv1 binding site
CsDof53	ABRE	1526	1532	abscisic acid responsiveness
CsDof53	ABRE	1527	1532	abscisic acid responsiveness
CsDof53	ABRE	1792	1797	abscisic acid responsiveness
CsDof53	TC-rich repeats	404	413	cis-acting element involved in defense and stress responsiveness
CsDof53	TATC-box	821	828	cis-acting element involved in gibberellin-responsiveness
CsDof53	LTR	190	196	cis-acting element involved in low-temperature responsiveness
CsDof53	LTR	1825	1831	cis-acting element involved in low-temperature responsiveness
CsDof53	ARE	647	653	cis-acting regulatory element essential for the anaerobic induction
CsDof53	ARE	1298	1304	cis-acting regulatory element essential for the anaerobic induction
CsDof53	ARE	1557	1563	cis-acting regulatory element essential for the anaerobic induction
CsDof53	ARE	1576	1582	cis-acting regulatory element essential for the anaerobic induction
CsDof53	AuxRR-core	625	632	cis-acting regulatory element involved in auxin responsiveness
CsDof53	circadian	862	871	cis-acting regulatory element involved in circadian control
CsDof53	G-Box	1526	1532	cis-acting regulatory element involved in light responsiveness
CsDof53	G-Box	1792	1798	cis-acting regulatory element involved in light responsiveness
CsDof53	G-box	968	974	cis-acting regulatory element involved in light responsiveness
CsDof53	G-box	1525	1533	cis-acting regulatory element involved in light responsiveness
CsDof53	G-box	1526	1532	cis-acting regulatory element involved in light responsiveness
CsDof53	CGTCA-motif	533	538	cis-acting regulatory element involved in the MeJA-responsiveness
CsDof53	TGACG-motif	533	538	cis-acting regulatory element involved in the MeJA-responsiveness
CsDof53	CAAT-box	54	59	common cis-acting element in promoter and enhancer regions

Name	Cis-element	Start position	Stop position	Function
CsDof53	CAAT-box	217	222	common cis-acting element in promoter and enhancer regions
CsDof53	CAAT-box	318	323	common cis-acting element in promoter and enhancer regions
CsDof53	CAAT-box	367	377	common cis-acting element in promoter and enhancer regions
CsDof53	CAAT-box	1038	1043	common cis-acting element in promoter and enhancer regions
CsDof53	CAAT-box	1220	1225	common cis-acting element in promoter and enhancer regions
CsDof53	CAAT-box	1281	1286	common cis-acting element in promoter and enhancer regions
CsDof53	CAAT-box	1303	1308	common cis-acting element in promoter and enhancer regions
CsDof53	CAAT-box	1327	1332	common cis-acting element in promoter and enhancer regions
CsDof53	CAAT-box	1636	1641	common cis-acting element in promoter and enhancer regions
CsDof53	CAAT-box	1642	1647	common cis-acting element in promoter and enhancer regions
CsDof53	CAAT-box	1981	1986	common cis-acting element in promoter and enhancer regions
CsDof53	TATA-box	346	351	core promoter element around -30 of transcription start
CsDof53	TATA-box	347	351	core promoter element around -30 of transcription start
CsDof53	TATA-box	722	726	core promoter element around -30 of transcription start
CsDof53	TATA-box	953	959	core promoter element around -30 of transcription start
CsDof53	TATA-box	954	960	core promoter element around -30 of transcription start
CsDof53	TATA-box	955	959	core promoter element around -30 of transcription start
CsDof53	TATA-box	1135	1142	core promoter element around -30 of transcription start
CsDof53	TATA-box	1136	1142	core promoter element around -30 of transcription start
CsDof53	TATA-box	1137	1142	core promoter element around -30 of transcription start
CsDof53	TATA-box	1138	1142	core promoter element around -30 of transcription start
CsDof53	TATA-box	1233	1240	core promoter element around -30 of transcription start
CsDof53	TATA-box	1234	1240	core promoter element around -30 of transcription start
CsDof53	TATA-box	1235	1240	core promoter element around -30 of transcription start
CsDof53	TATA-box	1236	1240	core promoter element around -30 of transcription start
CsDof53	TATA-box	1244	1253	core promoter element around -30 of transcription start
CsDof53	TATA-box	1245	1252	core promoter element around -30 of transcription start
CsDof53	TATA-box	1246	1252	core promoter element around -30 of transcription start
CsDof53	TATA-box	1247	1252	core promoter element around -30 of transcription start
CsDof53	TATA-box	1248	1252	core promoter element around -30 of transcription start
CsDof53	TATA-box	1257	1264	core promoter element around -30 of transcription start
CsDof53	TATA-box	1315	1322	core promoter element around -30 of transcription start
CsDof53	TATA-box	1348	1352	core promoter element around -30 of transcription start
CsDof53	TATA-box	1436	1440	core promoter element around -30 of transcription start
CsDof53	TATA-box	1439	1445	core promoter element around -30 of transcription start
CsDof53	TATA-box	1440	1445	core promoter element around -30 of transcription start
CsDof53	TATA-box	1441	1445	core promoter element around -30 of transcription start
CsDof53	TATA-box	1467	1473	core promoter element around -30 of transcription start
CsDof53	TATA-box	1468	1472	core promoter element around -30 of transcription start
CsDof53	TATA-box	1585	1590	core promoter element around -30 of transcription start
CsDof53	TATA-box	1586	1590	core promoter element around -30 of transcription start
CsDof53	TATA-box	1652	1656	core promoter element around -30 of transcription start
CsDof53	TATA-box	1778	1785	core promoter element around -30 of transcription start
CsDof53	TATA-box	1864	1870	core promoter element around -30 of transcription start
CsDof53	TATA-box	1865	1871	core promoter element around -30 of transcription start
CsDof53	TATA-box	1866	1870	core promoter element around -30 of transcription start
CsDof53	TATA-box	1938	1943	core promoter element around -30 of transcription start
CsDof53	TATA-box	1939	1943	core promoter element around -30 of transcription start
CsDof53	P-box	331	338	gibberellin-responsive element
CsDof54	ABRE	1352	1357	abscisic acid responsiveness
CsDof54	ABRE	1706	1712	abscisic acid responsiveness

Name	Cis-element	Start position	Stop position	Function
CsDof54	ABRE	1707	1712	abscisic acid responsiveness
CsDof54	ABRE	1717	1722	abscisic acid responsiveness
CsDof54	LTR	1856	1862	cis-acting element involved in low-temperature responsiveness
CsDof54	ARE	650	656	cis-acting regulatory element essential for the anaerobic induction
CsDof54	ARE	711	717	cis-acting regulatory element essential for the anaerobic induction
CsDof54	ARE	1860	1866	cis-acting regulatory element essential for the anaerobic induction
CsDof54	G-box	65	71	cis-acting regulatory element involved in light responsiveness
CsDof54	G-box	289	295	cis-acting regulatory element involved in light responsiveness
CsDof54	G-box	1032	1038	cis-acting regulatory element involved in light responsiveness
CsDof54	G-box	1352	1358	cis-acting regulatory element involved in light responsiveness
CsDof54	G-box	1706	1712	cis-acting regulatory element involved in light responsiveness
CsDof54	G-box	1716	1722	cis-acting regulatory element involved in light responsiveness
CsDof54	G-Box	1706	1712	cis-acting regulatory element involved in light responsiveness
CsDof54	TGACG-motif	1065	1070	cis-acting regulatory element involved in the MeJA-responsiveness
CsDof54	TGACG-motif	1715	1720	cis-acting regulatory element involved in the MeJA-responsiveness
CsDof54	TGACG-motif	1729	1734	cis-acting regulatory element involved in the MeJA-responsiveness
CsDof54	CGTCA-motif	1065	1070	cis-acting regulatory element involved in the MeJA-responsiveness
CsDof54	CGTCA-motif	1715	1720	cis-acting regulatory element involved in the MeJA-responsiveness
CsDof54	CGTCA-motif	1729	1734	cis-acting regulatory element involved in the MeJA-responsiveness
CsDof54	O2-site	491	499	cis-acting regulatory element involved in zein metabolism regulation
CsDof54	CAT-box	662	668	cis-acting regulatory element related to meristem expression
CsDof54	CAT-box	1331	1337	cis-acting regulatory element related to meristem expression
CsDof54	CAAT-box	136	141	common cis-acting element in promoter and enhancer regions
CsDof54	CAAT-box	143	148	common cis-acting element in promoter and enhancer regions
CsDof54	CAAT-box	223	228	common cis-acting element in promoter and enhancer regions
CsDof54	CAAT-box	495	500	common cis-acting element in promoter and enhancer regions
CsDof54	CAAT-box	637	642	common cis-acting element in promoter and enhancer regions
CsDof54	CAAT-box	709	714	common cis-acting element in promoter and enhancer regions
CsDof54	CAAT-box	724	729	common cis-acting element in promoter and enhancer regions
CsDof54	CAAT-box	749	754	common cis-acting element in promoter and enhancer regions
CsDof54	CAAT-box	824	829	common cis-acting element in promoter and enhancer regions
CsDof54	CAAT-box	916	921	common cis-acting element in promoter and enhancer regions
CsDof54	CAAT-box	923	928	common cis-acting element in promoter and enhancer regions
CsDof54	CAAT-box	1019	1024	common cis-acting element in promoter and enhancer regions
CsDof54	CAAT-box	1070	1075	common cis-acting element in promoter and enhancer regions
CsDof54	CAAT-box	1081	1086	common cis-acting element in promoter and enhancer regions
CsDof54	CAAT-box	1118	1123	common cis-acting element in promoter and enhancer regions
CsDof54	CAAT-box	1181	1186	common cis-acting element in promoter and enhancer regions
CsDof54	CAAT-box	1226	1231	common cis-acting element in promoter and enhancer regions
CsDof54	CAAT-box	1255	1260	common cis-acting element in promoter and enhancer regions
CsDof54	CAAT-box	1327	1332	common cis-acting element in promoter and enhancer regions
CsDof54	CAAT-box	1424	1429	common cis-acting element in promoter and enhancer regions
CsDof54	CAAT-box	1829	1834	common cis-acting element in promoter and enhancer regions
CsDof54	CAAT-box	1911	1916	common cis-acting element in promoter and enhancer regions
CsDof54	TATA-box	87	93	core promoter element around -30 of transcription start
CsDof54	TATA-box	88	92	core promoter element around -30 of transcription start
CsDof54	TATA-box	129	135	core promoter element around -30 of transcription start
CsDof54	TATA-box	130	137	core promoter element around -30 of transcription start
CsDof54	TATA-box	131	137	core promoter element around -30 of transcription start
CsDof54	TATA-box	132	138	core promoter element around -30 of transcription start
CsDof54	TATA-box	133	137	core promoter element around -30 of transcription start

Name	Cis-element	Start position	Stop position	Function
CsDof54	TATA-box	171	177	core promoter element around -30 of transcription start
CsDof54	TATA-box	172	178	core promoter element around -30 of transcription start
CsDof54	TATA-box	173	179	core promoter element around -30 of transcription start
CsDof54	TATA-box	174	180	core promoter element around -30 of transcription start
CsDof54	TATA-box	176	180	core promoter element around -30 of transcription start
CsDof54	TATA-box	513	522	core promoter element around -30 of transcription start
CsDof54	TATA-box	514	521	core promoter element around -30 of transcription start
CsDof54	TATA-box	515	521	core promoter element around -30 of transcription start
CsDof54	TATA-box	516	523	core promoter element around -30 of transcription start
CsDof54	TATA-box	517	523	core promoter element around -30 of transcription start
CsDof54	TATA-box	518	524	core promoter element around -30 of transcription start
CsDof54	TATA-box	519	523	core promoter element around -30 of transcription start
CsDof54	TATA-box	575	581	core promoter element around -30 of transcription start
CsDof54	TATA-box	576	581	core promoter element around -30 of transcription start
CsDof54	TATA-box	577	581	core promoter element around -30 of transcription start
CsDof54	TATA-box	620	626	core promoter element around -30 of transcription start
CsDof54	TATA-box	621	627	core promoter element around -30 of transcription start
CsDof54	TATA-box	622	628	core promoter element around -30 of transcription start
CsDof54	TATA-box	624	628	core promoter element around -30 of transcription start
CsDof54	TATA-box	626	633	core promoter element around -30 of transcription start
CsDof54	TATA-box	642	649	core promoter element around -30 of transcription start
CsDof54	TATA-box	643	649	core promoter element around -30 of transcription start
CsDof54	TATA-box	644	649	core promoter element around -30 of transcription start
CsDof54	TATA-box	645	649	core promoter element around -30 of transcription start
CsDof54	TATA-box	831	835	core promoter element around -30 of transcription start
CsDof54	TATA-box	836	842	core promoter element around -30 of transcription start
CsDof54	TATA-box	837	842	core promoter element around -30 of transcription start
CsDof54	TATA-box	838	842	core promoter element around -30 of transcription start
CsDof54	TATA-box	848	852	core promoter element around -30 of transcription start
CsDof54	TATA-box	856	861	core promoter element around -30 of transcription start
CsDof54	TATA-box	857	861	core promoter element around -30 of transcription start
CsDof54	TATA-box	926	932	core promoter element around -30 of transcription start
CsDof54	TATA-box	927	931	core promoter element around -30 of transcription start
CsDof54	TATA-box	1150	1154	core promoter element around -30 of transcription start
CsDof54	TATA-box	1189	1196	core promoter element around -30 of transcription start
CsDof54	TATA-box	1190	1196	core promoter element around -30 of transcription start
CsDof54	TATA-box	1191	1196	core promoter element around -30 of transcription start
CsDof54	TATA-box	1192	1196	core promoter element around -30 of transcription start
CsDof54	TATA-box	1197	1204	core promoter element around -30 of transcription start
CsDof54	TATA-box	1206	1213	core promoter element around -30 of transcription start
CsDof54	TATA-box	1207	1213	core promoter element around -30 of transcription start
CsDof54	TATA-box	1208	1213	core promoter element around -30 of transcription start
CsDof54	TATA-box	1209	1213	core promoter element around -30 of transcription start
CsDof54	TATA-box	1233	1237	core promoter element around -30 of transcription start
CsDof54	TATA-box	1360	1367	core promoter element around -30 of transcription start
CsDof54	TATA-box	1361	1367	core promoter element around -30 of transcription start
CsDof54	TATA-box	1362	1367	core promoter element around -30 of transcription start
CsDof54	TATA-box	1363	1367	core promoter element around -30 of transcription start
CsDof54	TATA-box	1379	1386	core promoter element around -30 of transcription start
CsDof54	TATA-box	1380	1386	core promoter element around -30 of transcription start
CsDof54	TATA-box	1381	1388	core promoter element around -30 of transcription start

Name	Cis-element	Start position	Stop position	Function
CsDof54	TATA-box	1382	1388	core promoter element around -30 of transcription start
CsDof54	TATA-box	1384	1388	core promoter element around -30 of transcription start
CsDof54	TATA-box	1389	1395	core promoter element around -30 of transcription start
CsDof54	TATA-box	1390	1395	core promoter element around -30 of transcription start
CsDof54	TATA-box	1391	1403	core promoter element around -30 of transcription start
CsDof54	TATA-box	1419	1425	core promoter element around -30 of transcription start
CsDof54	TATA-box	1420	1424	core promoter element around -30 of transcription start
CsDof54	TATA-box	1484	1491	core promoter element around -30 of transcription start
CsDof54	TATA-box	1532	1539	core promoter element around -30 of transcription start
CsDof54	TATA-box	1681	1685	core promoter element around -30 of transcription start
CsDof54	GC-motif	5	11	enhancer-like element involved in anoxic specific inducibility
CsDof54	P-box	1737	1744	gibberellin-responsive element
CsDof54	MBS	963	969	MYB binding site involved in drought-inducibility
CsDof54	MRE	1158	1165	MYB binding site involved in light responsiveness
CsDof55	ABRE	3	10	abscisic acid responsiveness
CsDof55	AT-rich element	1933	1943	binding site of AT-rich DNA binding protein (ATBP-1)
CsDof55	A-box	202	208	cis-acting regulatory element
CsDof55	ARE	730	736	cis-acting regulatory element essential for the anaerobic induction
CsDof55	RY-element	368	376	cis-acting regulatory element involved in seed-specific regulation
CsDof55	TGACG-motif	1647	1652	cis-acting regulatory element involved in the MeJA-responsiveness
CsDof55	TGACG-motif	1898	1903	cis-acting regulatory element involved in the MeJA-responsiveness
CsDof55	CGTCA-motif	1647	1652	cis-acting regulatory element involved in the MeJA-responsiveness
CsDof55	CGTCA-motif	1898	1903	cis-acting regulatory element involved in the MeJA-responsiveness
CsDof55	O2-site	412	421	cis-acting regulatory element involved in zein metabolism regulation
CsDof55	CAT-box	304	310	cis-acting regulatory element related to meristem expression
CsDof55	CAAT-box	61	66	common cis-acting element in promoter and enhancer regions
CsDof55	CAAT-box	382	387	common cis-acting element in promoter and enhancer regions
CsDof55	CAAT-box	629	634	common cis-acting element in promoter and enhancer regions
CsDof55	CAAT-box	644	651	common cis-acting element in promoter and enhancer regions
CsDof55	CAAT-box	801	806	common cis-acting element in promoter and enhancer regions
CsDof55	CAAT-box	1063	1068	common cis-acting element in promoter and enhancer regions
CsDof55	CAAT-box	1185	1190	common cis-acting element in promoter and enhancer regions
CsDof55	CAAT-box	1393	1398	common cis-acting element in promoter and enhancer regions
CsDof55	CAAT-box	1438	1443	common cis-acting element in promoter and enhancer regions
CsDof55	CAAT-box	1630	1635	common cis-acting element in promoter and enhancer regions
CsDof55	CAAT-box	1761	1766	common cis-acting element in promoter and enhancer regions
CsDof55	CAAT-box	1764	1769	common cis-acting element in promoter and enhancer regions
CsDof55	CAAT-box	1949	1954	common cis-acting element in promoter and enhancer regions
CsDof55	TATA-box	208	212	core promoter element around -30 of transcription start
CsDof55	TATA-box	355	359	core promoter element around -30 of transcription start
CsDof55	TATA-box	452	459	core promoter element around -30 of transcription start
CsDof55	TATA-box	453	459	core promoter element around -30 of transcription start
CsDof55	TATA-box	454	461	core promoter element around -30 of transcription start
CsDof55	TATA-box	455	461	core promoter element around -30 of transcription start
CsDof55	TATA-box	456	462	core promoter element around -30 of transcription start
CsDof55	TATA-box	457	463	core promoter element around -30 of transcription start
CsDof55	TATA-box	459	463	core promoter element around -30 of transcription start
CsDof55	TATA-box	670	677	core promoter element around -30 of transcription start
CsDof55	TATA-box	671	677	core promoter element around -30 of transcription start
CsDof55	TATA-box	672	677	core promoter element around -30 of transcription start
CsDof55	TATA-box	673	677	core promoter element around -30 of transcription start

Name	Cis-element	Start position	Stop position	Function
CsDof55	TATA-box	820	826	core promoter element around -30 of transcription start
CsDof55	TATA-box	821	826	core promoter element around -30 of transcription start
CsDof55	TATA-box	822	826	core promoter element around -30 of transcription start
CsDof55	TATA-box	833	837	core promoter element around -30 of transcription start
CsDof55	TATA-box	887	896	core promoter element around -30 of transcription start
CsDof55	TATA-box	893	897	core promoter element around -30 of transcription start
CsDof55	TATA-box	1318	1324	core promoter element around -30 of transcription start
CsDof55	TATA-box	1319	1323	core promoter element around -30 of transcription start
CsDof55	TATA-box	1327	1331	core promoter element around -30 of transcription start
CsDof55	TATA-box	1336	1342	core promoter element around -30 of transcription start
CsDof55	TATA-box	1337	1343	core promoter element around -30 of transcription start
CsDof55	TATA-box	1338	1344	core promoter element around -30 of transcription start
CsDof55	TATA-box	1339	1345	core promoter element around -30 of transcription start
CsDof55	TATA-box	1340	1346	core promoter element around -30 of transcription start
CsDof55	TATA-box	1341	1347	core promoter element around -30 of transcription start
CsDof55	TATA-box	1342	1348	core promoter element around -30 of transcription start
CsDof55	TATA-box	1343	1349	core promoter element around -30 of transcription start
CsDof55	TATA-box	1344	1350	core promoter element around -30 of transcription start
CsDof55	TATA-box	1345	1351	core promoter element around -30 of transcription start
CsDof55	TATA-box	1346	1352	core promoter element around -30 of transcription start
CsDof55	TATA-box	1347	1353	core promoter element around -30 of transcription start
CsDof55	TATA-box	1348	1354	core promoter element around -30 of transcription start
CsDof55	TATA-box	1349	1353	core promoter element around -30 of transcription start
CsDof55	TATA-box	1516	1522	core promoter element around -30 of transcription start
CsDof55	TATA-box	1517	1523	core promoter element around -30 of transcription start
CsDof55	TATA-box	1518	1524	core promoter element around -30 of transcription start
CsDof55	TATA-box	1519	1523	core promoter element around -30 of transcription start
CsDof55	TATA-box	1545	1551	core promoter element around -30 of transcription start
CsDof55	TATA-box	1547	1551	core promoter element around -30 of transcription start
CsDof55	TATA-box	1552	1558	core promoter element around -30 of transcription start
CsDof55	TATA-box	1554	1558	core promoter element around -30 of transcription start
CsDof55	TATA-box	1673	1677	core promoter element around -30 of transcription start
CsDof55	TATA-box	1742	1749	core promoter element around -30 of transcription start
CsDof55	TATA-box	1743	1749	core promoter element around -30 of transcription start
CsDof55	TATA-box	1744	1750	core promoter element around -30 of transcription start
CsDof55	TATA-box	1745	1749	core promoter element around -30 of transcription start
CsDof55	TATA-box	1774	1780	core promoter element around -30 of transcription start
CsDof55	TATA-box	1775	1779	core promoter element around -30 of transcription start
CsDof55	TATA-box	1854	1858	core promoter element around -30 of transcription start
CsDof55	TATA-box	1862	1868	core promoter element around -30 of transcription start
CsDof55	TATA-box	1864	1868	core promoter element around -30 of transcription start
CsDof55	TATA-box	1884	1890	core promoter element around -30 of transcription start
CsDof55	TATA-box	1885	1889	core promoter element around -30 of transcription start
CsDof55	TATA-box	1889	1896	core promoter element around -30 of transcription start
CsDof55	TATA-box	1890	1896	core promoter element around -30 of transcription start
CsDof55	TATA-box	1891	1898	core promoter element around -30 of transcription start
CsDof55	TATA-box	1892	1898	core promoter element around -30 of transcription start
CsDof55	TATA-box	1894	1898	core promoter element around -30 of transcription start
CsDof55	TATA-box	1911	1917	core promoter element around -30 of transcription start
CsDof55	TATA-box	1912	1918	core promoter element around -30 of transcription start
CsDof55	TATA-box	1913	1919	core promoter element around -30 of transcription start

Name	Cis-element	Start position	Stop position	Function
CsDof55	TATA-box	1914	1920	core promoter element around -30 of transcription start
CsDof55	TATA-box	1916	1920	core promoter element around -30 of transcription start
CsDof55	P-box	266	273	gibberellin-responsive element
CsDof55	GT1-motif	1563	1570	light responsive element
CsDof55	GT1-motif	1564	1570	light responsive element
CsDof56	ABRE	547	552	abscisic acid responsiveness
CsDof56	TGA-element	1261	1267	auxin-responsive element
CsDof56	TC-rich repeats	1351	1360	cis-acting element involved in defense and stress responsiveness
CsDof56	ACE	461	470	cis-acting element involved in light responsiveness
CsDof56	LTR	1	7	cis-acting element involved in low-temperature responsiveness
CsDof56	TCA-element	1911	1920	cis-acting element involved in salicylic acid responsiveness
CsDof56	G-box	546	552	cis-acting regulatory element involved in light responsiveness
CsDof56	TGACG-motif	1380	1385	cis-acting regulatory element involved in the MeJA-responsiveness
CsDof56	CGTCA-motif	1380	1385	cis-acting regulatory element involved in the MeJA-responsiveness
CsDof56	CAT-box	702	708	cis-acting regulatory element related to meristem expression
CsDof56	GCN4_motif	1232	1239	cis-regulatory element involved in endosperm expression
CsDof56	CAAT-box	125	130	common cis-acting element in promoter and enhancer regions
CsDof56	CAAT-box	230	235	common cis-acting element in promoter and enhancer regions
CsDof56	CAAT-box	260	265	common cis-acting element in promoter and enhancer regions
CsDof56	CAAT-box	439	444	common cis-acting element in promoter and enhancer regions
CsDof56	CAAT-box	481	486	common cis-acting element in promoter and enhancer regions
CsDof56	CAAT-box	761	766	common cis-acting element in promoter and enhancer regions
CsDof56	CAAT-box	787	792	common cis-acting element in promoter and enhancer regions
CsDof56	CAAT-box	792	797	common cis-acting element in promoter and enhancer regions
CsDof56	CAAT-box	880	885	common cis-acting element in promoter and enhancer regions
CsDof56	CAAT-box	927	932	common cis-acting element in promoter and enhancer regions
CsDof56	CAAT-box	930	935	common cis-acting element in promoter and enhancer regions
CsDof56	CAAT-box	1082	1087	common cis-acting element in promoter and enhancer regions
CsDof56	CAAT-box	1293	1298	common cis-acting element in promoter and enhancer regions
CsDof56	CAAT-box	1520	1525	common cis-acting element in promoter and enhancer regions
CsDof56	CAAT-box	1623	1628	common cis-acting element in promoter and enhancer regions
CsDof56	CAAT-box	1699	1704	common cis-acting element in promoter and enhancer regions
CsDof56	CAAT-box	1765	1770	common cis-acting element in promoter and enhancer regions
CsDof56	CAAT-box	1810	1815	common cis-acting element in promoter and enhancer regions
CsDof56	CAAT-box	1872	1877	common cis-acting element in promoter and enhancer regions
CsDof56	CAAT-box	1932	1937	common cis-acting element in promoter and enhancer regions
CsDof56	TATA-box	29	35	core promoter element around -30 of transcription start
CsDof56	TATA-box	30	34	core promoter element around -30 of transcription start
CsDof56	TATA-box	72	78	core promoter element around -30 of transcription start
CsDof56	TATA-box	74	78	core promoter element around -30 of transcription start
CsDof56	TATA-box	138	146	core promoter element around -30 of transcription start
CsDof56	TATA-box	190	196	core promoter element around -30 of transcription start
CsDof56	TATA-box	191	197	core promoter element around -30 of transcription start
CsDof56	TATA-box	193	197	core promoter element around -30 of transcription start
CsDof56	TATA-box	358	362	core promoter element around -30 of transcription start
CsDof56	TATA-box	376	382	core promoter element around -30 of transcription start
CsDof56	TATA-box	377	381	core promoter element around -30 of transcription start
CsDof56	TATA-box	393	401	core promoter element around -30 of transcription start
CsDof56	TATA-box	429	435	core promoter element around -30 of transcription start
CsDof56	TATA-box	430	435	core promoter element around -30 of transcription start
CsDof56	TATA-box	431	435	core promoter element around -30 of transcription start

Name	Cis-element	Start position	Stop position	Function
CsDof56	TATA-box	530	536	core promoter element around -30 of transcription start
CsDof56	TATA-box	531	537	core promoter element around -30 of transcription start
CsDof56	TATA-box	533	537	core promoter element around -30 of transcription start
CsDof56	TATA-box	687	691	core promoter element around -30 of transcription start
CsDof56	TATA-box	815	821	core promoter element around -30 of transcription start
CsDof56	TATA-box	816	822	core promoter element around -30 of transcription start
CsDof56	TATA-box	817	823	core promoter element around -30 of transcription start
CsDof56	TATA-box	818	824	core promoter element around -30 of transcription start
CsDof56	TATA-box	819	825	core promoter element around -30 of transcription start
CsDof56	TATA-box	821	825	core promoter element around -30 of transcription start
CsDof56	TATA-box	965	974	core promoter element around -30 of transcription start
CsDof56	TATA-box	966	972	core promoter element around -30 of transcription start
CsDof56	TATA-box	967	973	core promoter element around -30 of transcription start
CsDof56	TATA-box	968	974	core promoter element around -30 of transcription start
CsDof56	TATA-box	969	975	core promoter element around -30 of transcription start
CsDof56	TATA-box	970	976	core promoter element around -30 of transcription start
CsDof56	TATA-box	971	977	core promoter element around -30 of transcription start
CsDof56	TATA-box	972	978	core promoter element around -30 of transcription start
CsDof56	TATA-box	973	979	core promoter element around -30 of transcription start
CsDof56	TATA-box	974	980	core promoter element around -30 of transcription start
CsDof56	TATA-box	975	981	core promoter element around -30 of transcription start
CsDof56	TATA-box	976	982	core promoter element around -30 of transcription start
CsDof56	TATA-box	977	983	core promoter element around -30 of transcription start
CsDof56	TATA-box	978	984	core promoter element around -30 of transcription start
CsDof56	TATA-box	979	985	core promoter element around -30 of transcription start
CsDof56	TATA-box	980	986	core promoter element around -30 of transcription start
CsDof56	TATA-box	981	987	core promoter element around -30 of transcription start
CsDof56	TATA-box	982	988	core promoter element around -30 of transcription start
CsDof56	TATA-box	983	989	core promoter element around -30 of transcription start
CsDof56	TATA-box	984	990	core promoter element around -30 of transcription start
CsDof56	TATA-box	985	991	core promoter element around -30 of transcription start
CsDof56	TATA-box	986	992	core promoter element around -30 of transcription start
CsDof56	TATA-box	987	993	core promoter element around -30 of transcription start
CsDof56	TATA-box	988	994	core promoter element around -30 of transcription start
CsDof56	TATA-box	989	995	core promoter element around -30 of transcription start
CsDof56	TATA-box	990	996	core promoter element around -30 of transcription start
CsDof56	TATA-box	991	995	core promoter element around -30 of transcription start
CsDof56	TATA-box	1042	1048	core promoter element around -30 of transcription start
CsDof56	TATA-box	1043	1047	core promoter element around -30 of transcription start
CsDof56	TATA-box	1301	1307	core promoter element around -30 of transcription start
CsDof56	TATA-box	1302	1308	core promoter element around -30 of transcription start
CsDof56	TATA-box	1303	1307	core promoter element around -30 of transcription start
CsDof56	TATA-box	1320	1326	core promoter element around -30 of transcription start
CsDof56	TATA-box	1321	1325	core promoter element around -30 of transcription start
CsDof56	TATA-box	1437	1441	core promoter element around -30 of transcription start
CsDof56	TATA-box	1553	1557	core promoter element around -30 of transcription start
CsDof56	TATA-box	1626	1632	core promoter element around -30 of transcription start
CsDof56	TATA-box	1627	1632	core promoter element around -30 of transcription start
CsDof56	TATA-box	1628	1632	core promoter element around -30 of transcription start
CsDof56	TATA-box	1641	1647	core promoter element around -30 of transcription start
CsDof56	TATA-box	1642	1648	core promoter element around -30 of transcription start

Name	Cis-element	Start position	Stop position	Function
CsDof56	TATA-box	1643	1649	core promoter element around -30 of transcription start
CsDof56	TATA-box	1644	1648	core promoter element around -30 of transcription start
CsDof56	TATA-box	1648	1654	core promoter element around -30 of transcription start
CsDof56	TATA-box	1649	1653	core promoter element around -30 of transcription start
CsDof56	TATA-box	1670	1675	core promoter element around -30 of transcription start
CsDof56	TATA-box	1671	1675	core promoter element around -30 of transcription start
CsDof56	TATA-box	1753	1758	core promoter element around -30 of transcription start
CsDof56	TATA-box	1754	1758	core promoter element around -30 of transcription start
CsDof56	TATA-box	1897	1904	core promoter element around -30 of transcription start
CsDof56	GARE-motif	67	74	gibberellin-responsive element
CsDof56	GT1-motif	133	139	light responsive element
CsDof56	GT1-motif	149	155	light responsive element
CsDof56	GT1-motif	665	671	light responsive element
CsDof56	WUN-motif	10	19	wound-responsive element
CsDof56	WUN-motif	798	807	wound-responsive element
CsDof57	ABRE	402	407	abscisic acid responsiveness
CsDof57	ABRE	593	598	abscisic acid responsiveness
CsDof57	ABRE	1595	1604	abscisic acid responsiveness
CsDof57	ABRE	1853	1863	abscisic acid responsiveness
CsDof57	ABRE	1855	1860	abscisic acid responsiveness
CsDof57	ARE	426	432	cis-acting regulatory element essential for the anaerobic induction
CsDof57	ARE	629	635	cis-acting regulatory element essential for the anaerobic induction
CsDof57	ARE	721	727	cis-acting regulatory element essential for the anaerobic induction
CsDof57	ARE	852	858	cis-acting regulatory element essential for the anaerobic induction
CsDof57	G-Box	592	598	cis-acting regulatory element involved in light responsiveness
CsDof57	G-Box	1855	1861	cis-acting regulatory element involved in light responsiveness
CsDof57	G-box	401	407	cis-acting regulatory element involved in light responsiveness
CsDof57	O2-site	407	416	cis-acting regulatory element involved in zein metabolism regulation
CsDof57	CAT-box	1136	1142	cis-acting regulatory element related to meristem expression
CsDof57	CAT-box	1207	1213	cis-acting regulatory element related to meristem expression
CsDof57	CAAT-box	483	488	common cis-acting element in promoter and enhancer regions
CsDof57	CAAT-box	655	660	common cis-acting element in promoter and enhancer regions
CsDof57	CAAT-box	665	670	common cis-acting element in promoter and enhancer regions
CsDof57	CAAT-box	699	704	common cis-acting element in promoter and enhancer regions
CsDof57	CAAT-box	910	915	common cis-acting element in promoter and enhancer regions
CsDof57	CAAT-box	1194	1199	common cis-acting element in promoter and enhancer regions
CsDof57	CAAT-box	1291	1296	common cis-acting element in promoter and enhancer regions
CsDof57	CAAT-box	1346	1351	common cis-acting element in promoter and enhancer regions
CsDof57	CAAT-box	1650	1655	common cis-acting element in promoter and enhancer regions
CsDof57	TATA-box	684	693	core promoter element around -30 of transcription start
CsDof57	TATA-box	685	692	core promoter element around -30 of transcription start
CsDof57	TATA-box	686	692	core promoter element around -30 of transcription start
CsDof57	TATA-box	687	692	core promoter element around -30 of transcription start
CsDof57	TATA-box	688	692	core promoter element around -30 of transcription start
CsDof57	TATA-box	882	890	core promoter element around -30 of transcription start
CsDof57	TATA-box	905	910	core promoter element around -30 of transcription start
CsDof57	TATA-box	906	910	core promoter element around -30 of transcription start
CsDof57	TATA-box	997	1001	core promoter element around -30 of transcription start
CsDof57	TATA-box	1015	1019	core promoter element around -30 of transcription start
CsDof57	TATA-box	1263	1267	core promoter element around -30 of transcription start
CsDof57	TATA-box	1311	1317	core promoter element around -30 of transcription start

Name	Cis-element	Start position	Stop position	Function
CsDof57	TATA-box	1312	1318	core promoter element around -30 of transcription start
CsDof57	TATA-box	1313	1319	core promoter element around -30 of transcription start
CsDof57	TATA-box	1314	1318	core promoter element around -30 of transcription start
CsDof57	TATA-box	1335	1342	core promoter element around -30 of transcription start
CsDof57	TATA-box	1336	1342	core promoter element around -30 of transcription start
CsDof57	TATA-box	1337	1342	core promoter element around -30 of transcription start
CsDof57	TATA-box	1338	1342	core promoter element around -30 of transcription start
CsDof57	TATA-box	1353	1361	core promoter element around -30 of transcription start
CsDof57	TATA-box	1432	1438	core promoter element around -30 of transcription start
CsDof57	TATA-box	1433	1438	core promoter element around -30 of transcription start
CsDof57	TATA-box	1434	1438	core promoter element around -30 of transcription start
CsDof57	TATA-box	1563	1567	core promoter element around -30 of transcription start
CsDof57	TATA-box	1621	1626	core promoter element around -30 of transcription start
CsDof57	TATA-box	1622	1626	core promoter element around -30 of transcription start
CsDof57	TATA-box	1684	1691	core promoter element around -30 of transcription start
CsDof57	TATA-box	1687	1693	core promoter element around -30 of transcription start
CsDof57	TATA-box	1689	1693	core promoter element around -30 of transcription start
CsDof57	TATA-box	1695	1701	core promoter element around -30 of transcription start
CsDof57	TATA-box	1696	1701	core promoter element around -30 of transcription start
CsDof57	TATA-box	1697	1701	core promoter element around -30 of transcription start
CsDof57	TATA-box	1716	1722	core promoter element around -30 of transcription start
CsDof57	TATA-box	1717	1722	core promoter element around -30 of transcription start
CsDof57	TATA-box	1718	1722	core promoter element around -30 of transcription start
CsDof57	TATA-box	1746	1750	core promoter element around -30 of transcription start
CsDof57	TATA-box	1903	1911	core promoter element around -30 of transcription start
CsDof57	TATA-box	1906	1913	core promoter element around -30 of transcription start
CsDof57	TATA-box	1907	1913	core promoter element around -30 of transcription start
CsDof57	TATA-box	1908	1914	core promoter element around -30 of transcription start
CsDof57	TATA-box	1909	1913	core promoter element around -30 of transcription start
CsDof57	TATA-box	1960	1965	core promoter element around -30 of transcription start
CsDof57	TATA-box	1961	1965	core promoter element around -30 of transcription start
CsDof57	GARE-motif	543	550	gibberellin-responsive element
CsDof57	GT1-motif	582	588	light responsive element
CsDof57	GT1-motif	1062	1068	light responsive element
CsDof57	MBS	1072	1078	MYB binding site involved in drought-inducibility
CsDof57	CCAAT-box	1148	1154	MYBHv1 binding site
CsDof58	ABRE	562	567	abscisic acid responsiveness
CsDof58	ABRE	627	632	abscisic acid responsiveness
CsDof58	ABRE	1853	1863	abscisic acid responsiveness
CsDof58	ABRE	1855	1860	abscisic acid responsiveness
CsDof58	ARE	271	277	cis-acting regulatory element essential for the anaerobic induction
CsDof58	ARE	448	454	cis-acting regulatory element essential for the anaerobic induction
CsDof58	ARE	638	644	cis-acting regulatory element essential for the anaerobic induction
CsDof58	ARE	729	735	cis-acting regulatory element essential for the anaerobic induction
CsDof58	ARE	860	866	cis-acting regulatory element essential for the anaerobic induction
CsDof58	G-Box	561	567	cis-acting regulatory element involved in light responsiveness
CsDof58	G-Box	627	633	cis-acting regulatory element involved in light responsiveness
CsDof58	G-Box	1855	1861	cis-acting regulatory element involved in light responsiveness
CsDof58	TGACG-motif	807	812	cis-acting regulatory element involved in the MeJA-responsiveness
CsDof58	TGACG-motif	1398	1403	cis-acting regulatory element involved in the MeJA-responsiveness
CsDof58	CGTCA-motif	807	812	cis-acting regulatory element involved in the MeJA-responsiveness

Name	Cis-element	Start position	Stop position	Function
CsDof58	CGTCA-motif	1398	1403	cis-acting regulatory element involved in the MeJA-responsiveness
CsDof58	CAT-box	1136	1142	cis-acting regulatory element related to meristem expression
CsDof58	CAAT-box	288	293	common cis-acting element in promoter and enhancer regions
CsDof58	CAAT-box	376	381	common cis-acting element in promoter and enhancer regions
CsDof58	CAAT-box	664	669	common cis-acting element in promoter and enhancer regions
CsDof58	CAAT-box	674	679	common cis-acting element in promoter and enhancer regions
CsDof58	CAAT-box	708	713	common cis-acting element in promoter and enhancer regions
CsDof58	CAAT-box	916	921	common cis-acting element in promoter and enhancer regions
CsDof58	CAAT-box	1194	1199	common cis-acting element in promoter and enhancer regions
CsDof58	CAAT-box	1297	1302	common cis-acting element in promoter and enhancer regions
CsDof58	CAAT-box	1352	1357	common cis-acting element in promoter and enhancer regions
CsDof58	CAAT-box	1458	1463	common cis-acting element in promoter and enhancer regions
CsDof58	CAAT-box	1649	1654	common cis-acting element in promoter and enhancer regions
CsDof58	TATA-box	156	162	core promoter element around -30 of transcription start
CsDof58	TATA-box	157	162	core promoter element around -30 of transcription start
CsDof58	TATA-box	158	162	core promoter element around -30 of transcription start
CsDof58	TATA-box	181	185	core promoter element around -30 of transcription start
CsDof58	TATA-box	202	208	core promoter element around -30 of transcription start
CsDof58	TATA-box	203	210	core promoter element around -30 of transcription start
CsDof58	TATA-box	204	210	core promoter element around -30 of transcription start
CsDof58	TATA-box	206	210	core promoter element around -30 of transcription start
CsDof58	TATA-box	693	702	core promoter element around -30 of transcription start
CsDof58	TATA-box	694	701	core promoter element around -30 of transcription start
CsDof58	TATA-box	695	701	core promoter element around -30 of transcription start
CsDof58	TATA-box	696	701	core promoter element around -30 of transcription start
CsDof58	TATA-box	697	701	core promoter element around -30 of transcription start
CsDof58	TATA-box	888	896	core promoter element around -30 of transcription start
CsDof58	TATA-box	911	916	core promoter element around -30 of transcription start
CsDof58	TATA-box	912	916	core promoter element around -30 of transcription start
CsDof58	TATA-box	1003	1007	core promoter element around -30 of transcription start
CsDof58	TATA-box	1021	1025	core promoter element around -30 of transcription start
CsDof58	TATA-box	1189	1195	core promoter element around -30 of transcription start
CsDof58	TATA-box	1190	1195	core promoter element around -30 of transcription start
CsDof58	TATA-box	1191	1195	core promoter element around -30 of transcription start
CsDof58	TATA-box	1269	1273	core promoter element around -30 of transcription start
CsDof58	TATA-box	1317	1323	core promoter element around -30 of transcription start
CsDof58	TATA-box	1318	1324	core promoter element around -30 of transcription start
CsDof58	TATA-box	1319	1325	core promoter element around -30 of transcription start
CsDof58	TATA-box	1320	1324	core promoter element around -30 of transcription start
CsDof58	TATA-box	1341	1348	core promoter element around -30 of transcription start
CsDof58	TATA-box	1342	1348	core promoter element around -30 of transcription start
CsDof58	TATA-box	1343	1348	core promoter element around -30 of transcription start
CsDof58	TATA-box	1344	1348	core promoter element around -30 of transcription start
CsDof58	TATA-box	1359	1367	core promoter element around -30 of transcription start
CsDof58	TATA-box	1440	1446	core promoter element around -30 of transcription start
CsDof58	TATA-box	1441	1446	core promoter element around -30 of transcription start
CsDof58	TATA-box	1442	1446	core promoter element around -30 of transcription start
CsDof58	TATA-box	1452	1456	core promoter element around -30 of transcription start
CsDof58	TATA-box	1557	1566	core promoter element around -30 of transcription start
CsDof58	TATA-box	1559	1563	core promoter element around -30 of transcription start
CsDof58	TATA-box	1620	1625	core promoter element around -30 of transcription start

Name	Cis-element	Start position	Stop position	Function
CsDof58	TATA-box	1621	1625	core promoter element around -30 of transcription start
CsDof58	TATA-box	1683	1690	core promoter element around -30 of transcription start
CsDof58	TATA-box	1686	1692	core promoter element around -30 of transcription start
CsDof58	TATA-box	1688	1692	core promoter element around -30 of transcription start
CsDof58	TATA-box	1694	1700	core promoter element around -30 of transcription start
CsDof58	TATA-box	1695	1700	core promoter element around -30 of transcription start
CsDof58	TATA-box	1696	1700	core promoter element around -30 of transcription start
CsDof58	TATA-box	1715	1721	core promoter element around -30 of transcription start
CsDof58	TATA-box	1716	1721	core promoter element around -30 of transcription start
CsDof58	TATA-box	1717	1721	core promoter element around -30 of transcription start
CsDof58	TATA-box	1745	1749	core promoter element around -30 of transcription start
CsDof58	TATA-box	1812	1818	core promoter element around -30 of transcription start
CsDof58	TATA-box	1814	1818	core promoter element around -30 of transcription start
CsDof58	TATA-box	1903	1911	core promoter element around -30 of transcription start
CsDof58	TATA-box	1906	1913	core promoter element around -30 of transcription start
CsDof58	TATA-box	1907	1913	core promoter element around -30 of transcription start
CsDof58	TATA-box	1908	1914	core promoter element around -30 of transcription start
CsDof58	TATA-box	1909	1913	core promoter element around -30 of transcription start
CsDof58	TATA-box	1960	1965	core promoter element around -30 of transcription start
CsDof58	TATA-box	1961	1965	core promoter element around -30 of transcription start
CsDof58	GT1-motif	551	557	light responsive element
CsDof58	GT1-motif	1068	1074	light responsive element
CsDof58	MBS	442	448	MYB binding site involved in drought-inducibility
CsDof58	MBSI	868	879	MYB binding site involved in flavonoid biosynthetic genes regulation
CsDof58	CCAAT-box	1148	1154	MYBHv1 binding site
CsDof59	ABRE	1535	1541	abscisic acid responsiveness
CsDof59	ABRE	1536	1541	abscisic acid responsiveness
CsDof59	ABRE	1800	1805	abscisic acid responsiveness
CsDof59	LTR	532	538	cis-acting element involved in low-temperature responsiveness
CsDof59	ARE	1296	1302	cis-acting regulatory element essential for the anaerobic induction
CsDof59	ARE	1307	1313	cis-acting regulatory element essential for the anaerobic induction
CsDof59	ARE	1564	1570	cis-acting regulatory element essential for the anaerobic induction
CsDof59	ARE	1583	1589	cis-acting regulatory element essential for the anaerobic induction
CsDof59	circadian	511	520	cis-acting regulatory element involved in circadian control
CsDof59	G-Box	1535	1541	cis-acting regulatory element involved in light responsiveness
CsDof59	G-Box	1800	1806	cis-acting regulatory element involved in light responsiveness
CsDof59	G-box	1534	1542	cis-acting regulatory element involved in light responsiveness
CsDof59	G-box	1535	1541	cis-acting regulatory element involved in light responsiveness
CsDof59	O2-site	382	391	cis-acting regulatory element involved in zein metabolism regulation
CsDof59	CAAT-box	821	826	common cis-acting element in promoter and enhancer regions
CsDof59	CAAT-box	863	868	common cis-acting element in promoter and enhancer regions
CsDof59	CAAT-box	1069	1074	common cis-acting element in promoter and enhancer regions
CsDof59	CAAT-box	1233	1238	common cis-acting element in promoter and enhancer regions
CsDof59	CAAT-box	1278	1283	common cis-acting element in promoter and enhancer regions
CsDof59	CAAT-box	1312	1317	common cis-acting element in promoter and enhancer regions
CsDof59	CAAT-box	1336	1341	common cis-acting element in promoter and enhancer regions
CsDof59	CAAT-box	1397	1402	common cis-acting element in promoter and enhancer regions
CsDof59	CAAT-box	1643	1648	common cis-acting element in promoter and enhancer regions
CsDof59	CAAT-box	1649	1654	common cis-acting element in promoter and enhancer regions
CsDof59	CAAT-box	1982	1987	common cis-acting element in promoter and enhancer regions
CsDof59	TATA-box	135	142	core promoter element around -30 of transcription start

Name	Cis-element	Start position	Stop position	Function
CsDof59	TATA-box	599	605	core promoter element around -30 of transcription start
CsDof59	TATA-box	600	606	core promoter element around -30 of transcription start
CsDof59	TATA-box	601	605	core promoter element around -30 of transcription start
CsDof59	TATA-box	626	630	core promoter element around -30 of transcription start
CsDof59	TATA-box	716	722	core promoter element around -30 of transcription start
CsDof59	TATA-box	717	723	core promoter element around -30 of transcription start
CsDof59	TATA-box	718	722	core promoter element around -30 of transcription start
CsDof59	TATA-box	929	934	core promoter element around -30 of transcription start
CsDof59	TATA-box	930	934	core promoter element around -30 of transcription start
CsDof59	TATA-box	957	961	core promoter element around -30 of transcription start
CsDof59	TATA-box	984	990	core promoter element around -30 of transcription start
CsDof59	TATA-box	985	991	core promoter element around -30 of transcription start
CsDof59	TATA-box	986	990	core promoter element around -30 of transcription start
CsDof59	TATA-box	1138	1145	core promoter element around -30 of transcription start
CsDof59	TATA-box	1139	1145	core promoter element around -30 of transcription start
CsDof59	TATA-box	1140	1145	core promoter element around -30 of transcription start
CsDof59	TATA-box	1141	1145	core promoter element around -30 of transcription start
CsDof59	TATA-box	1246	1253	core promoter element around -30 of transcription start
CsDof59	TATA-box	1247	1256	core promoter element around -30 of transcription start
CsDof59	TATA-box	1248	1253	core promoter element around -30 of transcription start
CsDof59	TATA-box	1249	1253	core promoter element around -30 of transcription start
CsDof59	TATA-box	1266	1273	core promoter element around -30 of transcription start
CsDof59	TATA-box	1324	1331	core promoter element around -30 of transcription start
CsDof59	TATA-box	1357	1361	core promoter element around -30 of transcription start
CsDof59	TATA-box	1443	1450	core promoter element around -30 of transcription start
CsDof59	TATA-box	1444	1450	core promoter element around -30 of transcription start
CsDof59	TATA-box	1445	1450	core promoter element around -30 of transcription start
CsDof59	TATA-box	1446	1450	core promoter element around -30 of transcription start
CsDof59	TATA-box	1449	1455	core promoter element around -30 of transcription start
CsDof59	TATA-box	1450	1455	core promoter element around -30 of transcription start
CsDof59	TATA-box	1451	1455	core promoter element around -30 of transcription start
CsDof59	TATA-box	1476	1482	core promoter element around -30 of transcription start
CsDof59	TATA-box	1477	1481	core promoter element around -30 of transcription start
CsDof59	TATA-box	1499	1506	core promoter element around -30 of transcription start
CsDof59	TATA-box	1500	1506	core promoter element around -30 of transcription start
CsDof59	TATA-box	1501	1506	core promoter element around -30 of transcription start
CsDof59	TATA-box	1502	1506	core promoter element around -30 of transcription start
CsDof59	TATA-box	1506	1512	core promoter element around -30 of transcription start
CsDof59	TATA-box	1507	1512	core promoter element around -30 of transcription start
CsDof59	TATA-box	1508	1512	core promoter element around -30 of transcription start
CsDof59	TATA-box	1592	1597	core promoter element around -30 of transcription start
CsDof59	TATA-box	1593	1597	core promoter element around -30 of transcription start
CsDof59	TATA-box	1659	1663	core promoter element around -30 of transcription start
CsDof59	TATA-box	1688	1692	core promoter element around -30 of transcription start
CsDof59	TATA-box	1786	1793	core promoter element around -30 of transcription start
CsDof59	TATA-box	1873	1879	core promoter element around -30 of transcription start
CsDof59	TATA-box	1874	1880	core promoter element around -30 of transcription start
CsDof59	TATA-box	1875	1879	core promoter element around -30 of transcription start
CsDof59	TATA-box	1939	1944	core promoter element around -30 of transcription start
CsDof59	TATA-box	1940	1944	core promoter element around -30 of transcription start
CsDof59	GT1-motif	1421	1428	light responsive element

Name	Cis-element	Start position	Stop position	Function
CsDof60	ABRE	438	447	abscisic acid responsiveness
CsDof60	ABRE	440	445	abscisic acid responsiveness
CsDof60	ABRE	1663	1669	abscisic acid responsiveness
CsDof60	ABRE	1664	1669	abscisic acid responsiveness
CsDof60	ABRE	1674	1679	abscisic acid responsiveness
CsDof60	ARE	770	776	cis-acting regulatory element essential for the anaerobic induction
CsDof60	ARE	831	837	cis-acting regulatory element essential for the anaerobic induction
CsDof60	G-box	192	198	cis-acting regulatory element involved in light responsiveness
CsDof60	G-box	411	417	cis-acting regulatory element involved in light responsiveness
CsDof60	G-box	440	446	cis-acting regulatory element involved in light responsiveness
CsDof60	G-box	1177	1183	cis-acting regulatory element involved in light responsiveness
CsDof60	G-box	1663	1669	cis-acting regulatory element involved in light responsiveness
CsDof60	G-box	1673	1679	cis-acting regulatory element involved in light responsiveness
CsDof60	G-Box	1663	1669	cis-acting regulatory element involved in light responsiveness
CsDof60	TGACG-motif	1672	1677	cis-acting regulatory element involved in the MeJA-responsiveness
CsDof60	CGTCA-motif	1672	1677	cis-acting regulatory element involved in the MeJA-responsiveness
CsDof60	O2-site	611	619	cis-acting regulatory element involved in zein metabolism regulation
CsDof60	CAT-box	782	788	cis-acting regulatory element related to meristem expression
CsDof60	CAAT-box	265	270	common cis-acting element in promoter and enhancer regions
CsDof60	CAAT-box	272	277	common cis-acting element in promoter and enhancer regions
CsDof60	CAAT-box	353	358	common cis-acting element in promoter and enhancer regions
CsDof60	CAAT-box	522	527	common cis-acting element in promoter and enhancer regions
CsDof60	CAAT-box	615	620	common cis-acting element in promoter and enhancer regions
CsDof60	CAAT-box	631	636	common cis-acting element in promoter and enhancer regions
CsDof60	CAAT-box	757	762	common cis-acting element in promoter and enhancer regions
CsDof60	CAAT-box	829	834	common cis-acting element in promoter and enhancer regions
CsDof60	CAAT-box	843	848	common cis-acting element in promoter and enhancer regions
CsDof60	CAAT-box	848	853	common cis-acting element in promoter and enhancer regions
CsDof60	CAAT-box	1035	1040	common cis-acting element in promoter and enhancer regions
CsDof60	CAAT-box	1042	1047	common cis-acting element in promoter and enhancer regions
CsDof60	CAAT-box	1139	1144	common cis-acting element in promoter and enhancer regions
CsDof60	CAAT-box	1184	1189	common cis-acting element in promoter and enhancer regions
CsDof60	CAAT-box	1291	1296	common cis-acting element in promoter and enhancer regions
CsDof60	CAAT-box	1307	1312	common cis-acting element in promoter and enhancer regions
CsDof60	CAAT-box	1684	1689	common cis-acting element in promoter and enhancer regions
CsDof60	CAAT-box	1791	1796	common cis-acting element in promoter and enhancer regions
CsDof60	CAAT-box	1887	1892	common cis-acting element in promoter and enhancer regions
CsDof60	TATA-box	213	219	core promoter element around -30 of transcription start
CsDof60	TATA-box	214	218	core promoter element around -30 of transcription start
CsDof60	TATA-box	256	262	core promoter element around -30 of transcription start
CsDof60	TATA-box	257	264	core promoter element around -30 of transcription start
CsDof60	TATA-box	258	264	core promoter element around -30 of transcription start
CsDof60	TATA-box	259	265	core promoter element around -30 of transcription start
CsDof60	TATA-box	260	266	core promoter element around -30 of transcription start
CsDof60	TATA-box	261	267	core promoter element around -30 of transcription start
CsDof60	TATA-box	262	266	core promoter element around -30 of transcription start
CsDof60	TATA-box	303	309	core promoter element around -30 of transcription start
CsDof60	TATA-box	304	310	core promoter element around -30 of transcription start
CsDof60	TATA-box	305	311	core promoter element around -30 of transcription start
CsDof60	TATA-box	306	310	core promoter element around -30 of transcription start
CsDof60	TATA-box	577	581	core promoter element around -30 of transcription start

Name	Cis-element	Start position	Stop position	Function
CsDof60	TATA-box	633	642	core promoter element around -30 of transcription start
CsDof60	TATA-box	634	641	core promoter element around -30 of transcription start
CsDof60	TATA-box	635	641	core promoter element around -30 of transcription start
CsDof60	TATA-box	636	643	core promoter element around -30 of transcription start
CsDof60	TATA-box	637	643	core promoter element around -30 of transcription start
CsDof60	TATA-box	638	644	core promoter element around -30 of transcription start
CsDof60	TATA-box	639	643	core promoter element around -30 of transcription start
CsDof60	TATA-box	695	701	core promoter element around -30 of transcription start
CsDof60	TATA-box	696	701	core promoter element around -30 of transcription start
CsDof60	TATA-box	697	701	core promoter element around -30 of transcription start
CsDof60	TATA-box	738	744	core promoter element around -30 of transcription start
CsDof60	TATA-box	739	746	core promoter element around -30 of transcription start
CsDof60	TATA-box	740	746	core promoter element around -30 of transcription start
CsDof60	TATA-box	741	747	core promoter element around -30 of transcription start
CsDof60	TATA-box	742	746	core promoter element around -30 of transcription start
CsDof60	TATA-box	761	769	core promoter element around -30 of transcription start
CsDof60	TATA-box	763	772	core promoter element around -30 of transcription start
CsDof60	TATA-box	764	769	core promoter element around -30 of transcription start
CsDof60	TATA-box	765	769	core promoter element around -30 of transcription start
CsDof60	TATA-box	948	954	core promoter element around -30 of transcription start
CsDof60	TATA-box	949	953	core promoter element around -30 of transcription start
CsDof60	TATA-box	953	960	core promoter element around -30 of transcription start
CsDof60	TATA-box	954	960	core promoter element around -30 of transcription start
CsDof60	TATA-box	955	960	core promoter element around -30 of transcription start
CsDof60	TATA-box	956	960	core promoter element around -30 of transcription start
CsDof60	TATA-box	974	979	core promoter element around -30 of transcription start
CsDof60	TATA-box	975	979	core promoter element around -30 of transcription start
CsDof60	TATA-box	1045	1051	core promoter element around -30 of transcription start
CsDof60	TATA-box	1046	1050	core promoter element around -30 of transcription start
CsDof60	TATA-box	1082	1089	core promoter element around -30 of transcription start
CsDof60	TATA-box	1213	1219	core promoter element around -30 of transcription start
CsDof60	TATA-box	1214	1219	core promoter element around -30 of transcription start
CsDof60	TATA-box	1215	1219	core promoter element around -30 of transcription start
CsDof60	TATA-box	1269	1273	core promoter element around -30 of transcription start
CsDof60	TATA-box	1300	1306	core promoter element around -30 of transcription start
CsDof60	TATA-box	1301	1306	core promoter element around -30 of transcription start
CsDof60	TATA-box	1302	1306	core promoter element around -30 of transcription start
CsDof60	TATA-box	1320	1324	core promoter element around -30 of transcription start
CsDof60	TATA-box	1325	1331	core promoter element around -30 of transcription start
CsDof60	TATA-box	1326	1331	core promoter element around -30 of transcription start
CsDof60	TATA-box	1327	1331	core promoter element around -30 of transcription start
CsDof60	TATA-box	1355	1361	core promoter element around -30 of transcription start
CsDof60	TATA-box	1356	1360	core promoter element around -30 of transcription start
CsDof60	TATA-box	1421	1428	core promoter element around -30 of transcription start
CsDof60	TATA-box	1637	1644	core promoter element around -30 of transcription start
CsDof60	MBS	968	974	MYB binding site involved in drought-inducibility
CsDof60	MRE	1277	1284	MYB binding site involved in light responsiveness
CsDof61	ABRE	1694	1699	abscisic acid responsiveness
CsDof61	TGA-element	1142	1148	auxin-responsive element
CsDof61	LTR	180	186	cis-acting element involved in low-temperature responsiveness
CsDof61	LTR	1231	1237	cis-acting element involved in low-temperature responsiveness

Name	Cis-element	Start position	Stop position	Function
CsDof61	Unnamed_1	213	224	cis-acting element involved in phytochrome down-regulation expression
CsDof61	ARE	798	804	cis-acting regulatory element essential for the anaerobic induction
CsDof61	AuxRR-core	935	942	cis-acting regulatory element involved in auxin responsiveness
CsDof61	G-box	34	40	cis-acting regulatory element involved in light responsiveness
CsDof61	G-Box	1694	1700	cis-acting regulatory element involved in light responsiveness
CsDof61	CAT-box	1206	1212	cis-acting regulatory element related to meristem expression
CsDof61	CAAT-box	150	155	common cis-acting element in promoter and enhancer regions
CsDof61	CAAT-box	197	202	common cis-acting element in promoter and enhancer regions
CsDof61	CAAT-box	281	286	common cis-acting element in promoter and enhancer regions
CsDof61	CAAT-box	535	540	common cis-acting element in promoter and enhancer regions
CsDof61	CAAT-box	548	553	common cis-acting element in promoter and enhancer regions
CsDof61	CAAT-box	558	563	common cis-acting element in promoter and enhancer regions
CsDof61	CAAT-box	805	810	common cis-acting element in promoter and enhancer regions
CsDof61	CAAT-box	820	825	common cis-acting element in promoter and enhancer regions
CsDof61	CAAT-box	959	964	common cis-acting element in promoter and enhancer regions
CsDof61	CAAT-box	1004	1009	common cis-acting element in promoter and enhancer regions
CsDof61	CAAT-box	1135	1140	common cis-acting element in promoter and enhancer regions
CsDof61	CAAT-box	1198	1203	common cis-acting element in promoter and enhancer regions
CsDof61	CAAT-box	1334	1339	common cis-acting element in promoter and enhancer regions
CsDof61	CAAT-box	1407	1412	common cis-acting element in promoter and enhancer regions
CsDof61	CAAT-box	1474	1479	common cis-acting element in promoter and enhancer regions
CsDof61	CAAT-box	1482	1487	common cis-acting element in promoter and enhancer regions
CsDof61	CAAT-box	1573	1578	common cis-acting element in promoter and enhancer regions
CsDof61	CAAT-box	1689	1694	common cis-acting element in promoter and enhancer regions
CsDof61	CAAT-box	1704	1709	common cis-acting element in promoter and enhancer regions
CsDof61	TATA-box	62	68	core promoter element around -30 of transcription start
CsDof61	TATA-box	63	69	core promoter element around -30 of transcription start
CsDof61	TATA-box	64	70	core promoter element around -30 of transcription start
CsDof61	TATA-box	65	71	core promoter element around -30 of transcription start
CsDof61	TATA-box	67	71	core promoter element around -30 of transcription start
CsDof61	TATA-box	84	88	core promoter element around -30 of transcription start
CsDof61	TATA-box	89	95	core promoter element around -30 of transcription start
CsDof61	TATA-box	90	95	core promoter element around -30 of transcription start
CsDof61	TATA-box	91	95	core promoter element around -30 of transcription start
CsDof61	TATA-box	104	108	core promoter element around -30 of transcription start
CsDof61	TATA-box	109	113	core promoter element around -30 of transcription start
CsDof61	TATA-box	260	266	core promoter element around -30 of transcription start
CsDof61	TATA-box	262	266	core promoter element around -30 of transcription start
CsDof61	TATA-box	337	343	core promoter element around -30 of transcription start
CsDof61	TATA-box	338	344	core promoter element around -30 of transcription start
CsDof61	TATA-box	339	345	core promoter element around -30 of transcription start
CsDof61	TATA-box	340	344	core promoter element around -30 of transcription start
CsDof61	TATA-box	379	387	core promoter element around -30 of transcription start
CsDof61	TATA-box	387	393	core promoter element around -30 of transcription start
CsDof61	TATA-box	388	392	core promoter element around -30 of transcription start
CsDof61	TATA-box	466	470	core promoter element around -30 of transcription start
CsDof61	TATA-box	528	535	core promoter element around -30 of transcription start
CsDof61	TATA-box	666	670	core promoter element around -30 of transcription start
CsDof61	TATA-box	753	757	core promoter element around -30 of transcription start
CsDof61	TATA-box	814	820	core promoter element around -30 of transcription start
CsDof61	TATA-box	815	819	core promoter element around -30 of transcription start

Name	Cis-element	Start position	Stop position	Function
CsDof61	TATA-box	889	895	core promoter element around -30 of transcription start
CsDof61	TATA-box	890	894	core promoter element around -30 of transcription start
CsDof61	TATA-box	1115	1119	core promoter element around -30 of transcription start
CsDof61	TATA-box	1162	1171	core promoter element around -30 of transcription start
CsDof61	TATA-box	1163	1170	core promoter element around -30 of transcription start
CsDof61	TATA-box	1164	1170	core promoter element around -30 of transcription start
CsDof61	TATA-box	1165	1172	core promoter element around -30 of transcription start
CsDof61	TATA-box	1166	1172	core promoter element around -30 of transcription start
CsDof61	TATA-box	1167	1173	core promoter element around -30 of transcription start
CsDof61	TATA-box	1168	1174	core promoter element around -30 of transcription start
CsDof61	TATA-box	1169	1175	core promoter element around -30 of transcription start
CsDof61	TATA-box	1170	1174	core promoter element around -30 of transcription start
CsDof61	TATA-box	1244	1249	core promoter element around -30 of transcription start
CsDof61	TATA-box	1245	1249	core promoter element around -30 of transcription start
CsDof61	TATA-box	1260	1267	core promoter element around -30 of transcription start
CsDof61	TATA-box	1273	1280	core promoter element around -30 of transcription start
CsDof61	TATA-box	1274	1280	core promoter element around -30 of transcription start
CsDof61	TATA-box	1275	1280	core promoter element around -30 of transcription start
CsDof61	TATA-box	1276	1280	core promoter element around -30 of transcription start
CsDof61	TATA-box	1375	1381	core promoter element around -30 of transcription start
CsDof61	TATA-box	1376	1381	core promoter element around -30 of transcription start
CsDof61	TATA-box	1377	1381	core promoter element around -30 of transcription start
CsDof61	TATA-box	1389	1395	core promoter element around -30 of transcription start
CsDof61	TATA-box	1390	1396	core promoter element around -30 of transcription start
CsDof61	TATA-box	1391	1397	core promoter element around -30 of transcription start
CsDof61	TATA-box	1392	1396	core promoter element around -30 of transcription start
CsDof61	TATA-box	1421	1429	core promoter element around -30 of transcription start
CsDof61	TATA-box	1560	1568	core promoter element around -30 of transcription start
CsDof61	TATA-box	1612	1619	core promoter element around -30 of transcription start
CsDof61	TATA-box	1613	1619	core promoter element around -30 of transcription start
CsDof61	TATA-box	1614	1619	core promoter element around -30 of transcription start
CsDof61	TATA-box	1615	1619	core promoter element around -30 of transcription start
CsDof61	TATA-box	1651	1657	core promoter element around -30 of transcription start
CsDof61	TATA-box	1652	1657	core promoter element around -30 of transcription start
CsDof61	TATA-box	1653	1657	core promoter element around -30 of transcription start
CsDof61	TATA-box	1778	1782	core promoter element around -30 of transcription start
CsDof61	TATA-box	1835	1841	core promoter element around -30 of transcription start
CsDof61	TATA-box	1837	1843	core promoter element around -30 of transcription start
CsDof61	TATA-box	1838	1844	core promoter element around -30 of transcription start
CsDof61	TATA-box	1839	1845	core promoter element around -30 of transcription start
CsDof61	TATA-box	1841	1845	core promoter element around -30 of transcription start
CsDof61	AT-rich sequence	413	422	element for maximal elicitor-mediated activation (2copies)
CsDof61	P-box	1861	1868	gibberellin-responsive element
CsDof61	GT1-motif	744	750	light responsive element
CsDof61	GT1-motif	1184	1190	light responsive element
CsDof61	CCAAT-box	995	1001	MYBHv1 binding site
CsDof62	ABRE	213	218	abscisic acid responsiveness
CsDof62	ABRE	339	345	abscisic acid responsiveness
CsDof62	ABRE	340	345	abscisic acid responsiveness
CsDof62	ABRE	481	486	abscisic acid responsiveness
CsDof62	LTR	1655	1661	cis-acting element involved in low-temperature responsiveness

Name	Cis-element	Start position	Stop position	Function
CsDof62	TCA-element	449	458	cis-acting element involved in salicylic acid responsiveness
CsDof62	TCA-element	1863	1872	cis-acting element involved in salicylic acid responsiveness
CsDof62	G-Box	339	345	cis-acting regulatory element involved in light responsiveness
CsDof62	G-box	212	218	cis-acting regulatory element involved in light responsiveness
CsDof62	G-box	337	346	cis-acting regulatory element involved in light responsiveness
CsDof62	G-box	339	345	cis-acting regulatory element involved in light responsiveness
CsDof62	G-box	480	486	cis-acting regulatory element involved in light responsiveness
CsDof62	CGTCA-motif	211	216	cis-acting regulatory element involved in the MeJA-responsiveness
CsDof62	CGTCA-motif	479	484	cis-acting regulatory element involved in the MeJA-responsiveness
CsDof62	CGTCA-motif	1803	1808	cis-acting regulatory element involved in the MeJA-responsiveness
CsDof62	TGACG-motif	211	216	cis-acting regulatory element involved in the MeJA-responsiveness
CsDof62	TGACG-motif	479	484	cis-acting regulatory element involved in the MeJA-responsiveness
CsDof62	TGACG-motif	1803	1808	cis-acting regulatory element involved in the MeJA-responsiveness
CsDof62	CAT-box	744	750	cis-acting regulatory element related to meristem expression
CsDof62	CAAT-box	70	75	common cis-acting element in promoter and enhancer regions
CsDof62	CAAT-box	122	127	common cis-acting element in promoter and enhancer regions
CsDof62	CAAT-box	148	153	common cis-acting element in promoter and enhancer regions
CsDof62	CAAT-box	178	183	common cis-acting element in promoter and enhancer regions
CsDof62	CAAT-box	297	302	common cis-acting element in promoter and enhancer regions
CsDof62	CAAT-box	386	391	common cis-acting element in promoter and enhancer regions
CsDof62	CAAT-box	433	438	common cis-acting element in promoter and enhancer regions
CsDof62	CAAT-box	444	449	common cis-acting element in promoter and enhancer regions
CsDof62	CAAT-box	718	723	common cis-acting element in promoter and enhancer regions
CsDof62	CAAT-box	785	790	common cis-acting element in promoter and enhancer regions
CsDof62	CAAT-box	839	844	common cis-acting element in promoter and enhancer regions
CsDof62	CAAT-box	872	877	common cis-acting element in promoter and enhancer regions
CsDof62	CAAT-box	927	932	common cis-acting element in promoter and enhancer regions
CsDof62	CAAT-box	1307	1312	common cis-acting element in promoter and enhancer regions
CsDof62	CAAT-box	1397	1402	common cis-acting element in promoter and enhancer regions
CsDof62	CAAT-box	1607	1612	common cis-acting element in promoter and enhancer regions
CsDof62	CAAT-box	1638	1643	common cis-acting element in promoter and enhancer regions
CsDof62	CAAT-box	1666	1671	common cis-acting element in promoter and enhancer regions
CsDof62	CAAT-box	1710	1715	common cis-acting element in promoter and enhancer regions
CsDof62	CAAT-box	1722	1727	common cis-acting element in promoter and enhancer regions
CsDof62	CAAT-box	1807	1812	common cis-acting element in promoter and enhancer regions
CsDof62	TATA-box	13	17	core promoter element around -30 of transcription start
CsDof62	TATA-box	84	91	core promoter element around -30 of transcription start
CsDof62	TATA-box	204	208	core promoter element around -30 of transcription start
CsDof62	TATA-box	517	522	core promoter element around -30 of transcription start
CsDof62	TATA-box	518	522	core promoter element around -30 of transcription start
CsDof62	TATA-box	556	565	core promoter element around -30 of transcription start
CsDof62	TATA-box	562	566	core promoter element around -30 of transcription start
CsDof62	TATA-box	674	680	core promoter element around -30 of transcription start
CsDof62	TATA-box	675	681	core promoter element around -30 of transcription start
CsDof62	TATA-box	677	681	core promoter element around -30 of transcription start
CsDof62	TATA-box	696	700	core promoter element around -30 of transcription start
CsDof62	TATA-box	753	757	core promoter element around -30 of transcription start
CsDof62	TATA-box	798	804	core promoter element around -30 of transcription start
CsDof62	TATA-box	799	805	core promoter element around -30 of transcription start
CsDof62	TATA-box	801	805	core promoter element around -30 of transcription start
CsDof62	TATA-box	863	869	core promoter element around -30 of transcription start

Name	Cis-element	Start position	Stop position	Function
CsDof62	TATA-box	864	869	core promoter element around -30 of transcription start
CsDof62	TATA-box	865	869	core promoter element around -30 of transcription start
CsDof62	TATA-box	876	883	core promoter element around -30 of transcription start
CsDof62	TATA-box	1007	1014	core promoter element around -30 of transcription start
CsDof62	TATA-box	1008	1014	core promoter element around -30 of transcription start
CsDof62	TATA-box	1009	1015	core promoter element around -30 of transcription start
CsDof62	TATA-box	1010	1014	core promoter element around -30 of transcription start
CsDof62	TATA-box	1080	1086	core promoter element around -30 of transcription start
CsDof62	TATA-box	1081	1086	core promoter element around -30 of transcription start
CsDof62	TATA-box	1082	1086	core promoter element around -30 of transcription start
CsDof62	TATA-box	1141	1149	core promoter element around -30 of transcription start
CsDof62	TATA-box	1156	1163	core promoter element around -30 of transcription start
CsDof62	TATA-box	1157	1163	core promoter element around -30 of transcription start
CsDof62	TATA-box	1158	1163	core promoter element around -30 of transcription start
CsDof62	TATA-box	1159	1163	core promoter element around -30 of transcription start
CsDof62	TATA-box	1167	1173	core promoter element around -30 of transcription start
CsDof62	TATA-box	1168	1174	core promoter element around -30 of transcription start
CsDof62	TATA-box	1169	1175	core promoter element around -30 of transcription start
CsDof62	TATA-box	1170	1176	core promoter element around -30 of transcription start
CsDof62	TATA-box	1171	1177	core promoter element around -30 of transcription start
CsDof62	TATA-box	1172	1178	core promoter element around -30 of transcription start
CsDof62	TATA-box	1173	1179	core promoter element around -30 of transcription start
CsDof62	TATA-box	1174	1180	core promoter element around -30 of transcription start
CsDof62	TATA-box	1175	1181	core promoter element around -30 of transcription start
CsDof62	TATA-box	1176	1182	core promoter element around -30 of transcription start
CsDof62	TATA-box	1177	1183	core promoter element around -30 of transcription start
CsDof62	TATA-box	1178	1184	core promoter element around -30 of transcription start
CsDof62	TATA-box	1179	1185	core promoter element around -30 of transcription start
CsDof62	TATA-box	1180	1186	core promoter element around -30 of transcription start
CsDof62	TATA-box	1181	1187	core promoter element around -30 of transcription start
CsDof62	TATA-box	1182	1188	core promoter element around -30 of transcription start
CsDof62	TATA-box	1183	1189	core promoter element around -30 of transcription start
CsDof62	TATA-box	1184	1190	core promoter element around -30 of transcription start
CsDof62	TATA-box	1186	1190	core promoter element around -30 of transcription start
CsDof62	TATA-box	1226	1230	core promoter element around -30 of transcription start
CsDof62	TATA-box	1357	1363	core promoter element around -30 of transcription start
CsDof62	TATA-box	1359	1363	core promoter element around -30 of transcription start
CsDof62	TATA-box	1527	1533	core promoter element around -30 of transcription start
CsDof62	TATA-box	1528	1532	core promoter element around -30 of transcription start
CsDof62	TATA-box	1584	1591	core promoter element around -30 of transcription start
CsDof62	TATA-box	1585	1591	core promoter element around -30 of transcription start
CsDof62	TATA-box	1586	1591	core promoter element around -30 of transcription start
CsDof62	TATA-box	1587	1591	core promoter element around -30 of transcription start
CsDof62	TATA-box	1645	1651	core promoter element around -30 of transcription start
CsDof62	TATA-box	1646	1652	core promoter element around -30 of transcription start
CsDof62	TATA-box	1648	1652	core promoter element around -30 of transcription start
CsDof62	TATA-box	1660	1666	core promoter element around -30 of transcription start
CsDof62	TATA-box	1661	1665	core promoter element around -30 of transcription start
CsDof62	TATA-box	1779	1786	core promoter element around -30 of transcription start
CsDof62	TATA-box	1970	1976	core promoter element around -30 of transcription start
CsDof62	TATA-box	1972	1976	core promoter element around -30 of transcription start

Name	Cis-element	Start position	Stop position	Function
CsDof62	HD-Zip 1	1602	1610	element involved in differentiation of the palisade mesophyll cells
CsDof62	P-box	845	852	gibberellin-responsive element
CsDof62	AAAC-motif	902	913	light responsive element
CsDof62	GT1-motif	427	433	light responsive element
CsDof62	GT1-motif	1376	1383	light responsive element
CsDof62	GT1-motif	1377	1383	light responsive element
CsDof63	ABRE	1373	1378	abscisic acid responsiveness
CsDof63	AT-rich element	491	501	binding site of AT-rich DNA binding protein (ATBP-1)
CsDof63	TC-rich repeats	1345	1354	cis-acting element involved in defense and stress responsiveness
CsDof63	LTR	447	453	cis-acting element involved in low-temperature responsiveness
CsDof63	TCA-element	190	199	cis-acting element involved in salicylic acid responsiveness
CsDof63	ARE	462	468	cis-acting regulatory element essential for the anaerobic induction
CsDof63	ARE	509	515	cis-acting regulatory element essential for the anaerobic induction
CsDof63	ARE	526	532	cis-acting regulatory element essential for the anaerobic induction
CsDof63	ARE	736	742	cis-acting regulatory element essential for the anaerobic induction
CsDof63	ARE	1908	1914	cis-acting regulatory element essential for the anaerobic induction
CsDof63	G-box	59	65	cis-acting regulatory element involved in light responsiveness
CsDof63	G-box	1373	1379	cis-acting regulatory element involved in light responsiveness
CsDof63	TGACG-motif	284	289	cis-acting regulatory element involved in the MeJA-responsiveness
CsDof63	CGTCA-motif	284	289	cis-acting regulatory element involved in the MeJA-responsiveness
CsDof63	O2-site	1847	1856	cis-acting regulatory element involved in zein metabolism regulation
CsDof63	CAAT-box	127	132	common cis-acting element in promoter and enhancer regions
CsDof63	CAAT-box	465	470	common cis-acting element in promoter and enhancer regions
CsDof63	CAAT-box	504	509	common cis-acting element in promoter and enhancer regions
CsDof63	CAAT-box	599	604	common cis-acting element in promoter and enhancer regions
CsDof63	CAAT-box	734	739	common cis-acting element in promoter and enhancer regions
CsDof63	CAAT-box	768	773	common cis-acting element in promoter and enhancer regions
CsDof63	CAAT-box	798	803	common cis-acting element in promoter and enhancer regions
CsDof63	CAAT-box	825	830	common cis-acting element in promoter and enhancer regions
CsDof63	CAAT-box	1016	1021	common cis-acting element in promoter and enhancer regions
CsDof63	CAAT-box	1173	1178	common cis-acting element in promoter and enhancer regions
CsDof63	CAAT-box	1329	1334	common cis-acting element in promoter and enhancer regions
CsDof63	CAAT-box	1358	1363	common cis-acting element in promoter and enhancer regions
CsDof63	CAAT-box	1473	1478	common cis-acting element in promoter and enhancer regions
CsDof63	CAAT-box	1480	1485	common cis-acting element in promoter and enhancer regions
CsDof63	CAAT-box	1494	1499	common cis-acting element in promoter and enhancer regions
CsDof63	CAAT-box	1530	1535	common cis-acting element in promoter and enhancer regions
CsDof63	CAAT-box	1685	1690	common cis-acting element in promoter and enhancer regions
CsDof63	CAAT-box	1722	1727	common cis-acting element in promoter and enhancer regions
CsDof63	CAAT-box	1891	1896	common cis-acting element in promoter and enhancer regions
CsDof63	CAAT-box	1922	1927	common cis-acting element in promoter and enhancer regions
CsDof63	TATA-box	499	503	core promoter element around -30 of transcription start
CsDof63	TATA-box	697	702	core promoter element around -30 of transcription start
CsDof63	TATA-box	698	702	core promoter element around -30 of transcription start
CsDof63	TATA-box	756	763	core promoter element around -30 of transcription start
CsDof63	TATA-box	757	763	core promoter element around -30 of transcription start
CsDof63	TATA-box	758	763	core promoter element around -30 of transcription start
CsDof63	TATA-box	759	763	core promoter element around -30 of transcription start
CsDof63	TATA-box	775	779	core promoter element around -30 of transcription start
CsDof63	TATA-box	787	793	core promoter element around -30 of transcription start
CsDof63	TATA-box	788	794	core promoter element around -30 of transcription start

Name	Cis-element	Start position	Stop position	Function
CsDof63	TATA-box	789	793	core promoter element around -30 of transcription start
CsDof63	TATA-box	989	995	core promoter element around -30 of transcription start
CsDof63	TATA-box	990	994	core promoter element around -30 of transcription start
CsDof63	TATA-box	1092	1096	core promoter element around -30 of transcription start
CsDof63	TATA-box	1102	1107	core promoter element around -30 of transcription start
CsDof63	TATA-box	1103	1107	core promoter element around -30 of transcription start
CsDof63	TATA-box	1226	1230	core promoter element around -30 of transcription start
CsDof63	TATA-box	1293	1297	core promoter element around -30 of transcription start
CsDof63	TATA-box	1468	1474	core promoter element around -30 of transcription start
CsDof63	TATA-box	1469	1474	core promoter element around -30 of transcription start
CsDof63	TATA-box	1470	1474	core promoter element around -30 of transcription start
CsDof63	TATA-box	1524	1529	core promoter element around -30 of transcription start
CsDof63	TATA-box	1525	1529	core promoter element around -30 of transcription start
CsDof63	TATA-box	1543	1549	core promoter element around -30 of transcription start
CsDof63	TATA-box	1544	1548	core promoter element around -30 of transcription start
CsDof63	TATA-box	1558	1564	core promoter element around -30 of transcription start
CsDof63	TATA-box	1559	1564	core promoter element around -30 of transcription start
CsDof63	TATA-box	1560	1564	core promoter element around -30 of transcription start
CsDof63	TATA-box	1616	1620	core promoter element around -30 of transcription start
CsDof63	TATA-box	1686	1698	core promoter element around -30 of transcription start
CsDof63	TATA-box	1688	1694	core promoter element around -30 of transcription start
CsDof63	TATA-box	1689	1695	core promoter element around -30 of transcription start
CsDof63	TATA-box	1690	1696	core promoter element around -30 of transcription start
CsDof63	TATA-box	1691	1697	core promoter element around -30 of transcription start
CsDof63	TATA-box	1692	1698	core promoter element around -30 of transcription start
CsDof63	TATA-box	1693	1699	core promoter element around -30 of transcription start
CsDof63	TATA-box	1694	1700	core promoter element around -30 of transcription start
CsDof63	TATA-box	1695	1701	core promoter element around -30 of transcription start
CsDof63	TATA-box	1696	1702	core promoter element around -30 of transcription start
CsDof63	TATA-box	1697	1701	core promoter element around -30 of transcription start
CsDof63	TATA-box	1765	1771	core promoter element around -30 of transcription start
CsDof63	TATA-box	1766	1771	core promoter element around -30 of transcription start
CsDof63	TATA-box	1767	1771	core promoter element around -30 of transcription start
CsDof63	TATA-box	1774	1778	core promoter element around -30 of transcription start
CsDof63	GARE-motif	842	849	gibberellin-responsive element
CsDof63	GT1-motif	1253	1259	light responsive element
CsDof63	MBS	572	578	MYB binding site involved in drought-inducibility
CsDof63	MBSI	1259	1269.5	MYB binding site involved in flavonoid biosynthetic genes regulation
CsDof63	CCAAT-box	1067	1073	MYBHv1 binding site
CsDof63	CCAAT-box	1657	1663	MYBHv1 binding site
CsDof64	ABRE	316	321	abscisic acid responsiveness
CsDof64	ABRE	974	983	abscisic acid responsiveness
CsDof64	MSA-like	1250	1259	cis-acting element involved in cell cycle regulation
CsDof64	MSA-like	1252	1261	cis-acting element involved in cell cycle regulation
CsDof64	ARE	132	138	cis-acting regulatory element essential for the anaerobic induction
CsDof64	ARE	877	883	cis-acting regulatory element essential for the anaerobic induction
CsDof64	ARE	927	933	cis-acting regulatory element essential for the anaerobic induction
CsDof64	AuxRR-core	1378	1385	cis-acting regulatory element involved in auxin responsiveness
CsDof64	G-box	315	321	cis-acting regulatory element involved in light responsiveness
CsDof64	G-box	973	984	cis-acting regulatory element involved in light responsiveness
CsDof64	CGTCA-motif	11	16	cis-acting regulatory element involved in the MeJA-responsiveness

Name	Cis-element	Start position	Stop position	Function
CsDof64	CGTCA-motif	1177	1182	cis-acting regulatory element involved in the MeJA-responsiveness
CsDof64	CGTCA-motif	1286	1291	cis-acting regulatory element involved in the MeJA-responsiveness
CsDof64	TGACG-motif	11	16	cis-acting regulatory element involved in the MeJA-responsiveness
CsDof64	TGACG-motif	1177	1182	cis-acting regulatory element involved in the MeJA-responsiveness
CsDof64	TGACG-motif	1286	1291	cis-acting regulatory element involved in the MeJA-responsiveness
CsDof64	O2-site	1270	1279	cis-acting regulatory element involved in zein metabolism regulation
CsDof64	CAAT-box	64	69	common cis-acting element in promoter and enhancer regions
CsDof64	CAAT-box	81	86	common cis-acting element in promoter and enhancer regions
CsDof64	CAAT-box	225	230	common cis-acting element in promoter and enhancer regions
CsDof64	CAAT-box	296	301	common cis-acting element in promoter and enhancer regions
CsDof64	CAAT-box	853	858	common cis-acting element in promoter and enhancer regions
CsDof64	CAAT-box	1260	1265	common cis-acting element in promoter and enhancer regions
CsDof64	CAAT-box	1393	1398	common cis-acting element in promoter and enhancer regions
CsDof64	CAAT-box	1487	1492	common cis-acting element in promoter and enhancer regions
CsDof64	CAAT-box	1904	1909	common cis-acting element in promoter and enhancer regions
CsDof64	TATA-box	48	54	core promoter element around -30 of transcription start
CsDof64	TATA-box	49	54	core promoter element around -30 of transcription start
CsDof64	TATA-box	50	54	core promoter element around -30 of transcription start
CsDof64	TATA-box	335	341	core promoter element around -30 of transcription start
CsDof64	TATA-box	336	342	core promoter element around -30 of transcription start
CsDof64	TATA-box	337	341	core promoter element around -30 of transcription start
CsDof64	TATA-box	503	509	core promoter element around -30 of transcription start
CsDof64	TATA-box	504	508	core promoter element around -30 of transcription start
CsDof64	TATA-box	508	514	core promoter element around -30 of transcription start
CsDof64	TATA-box	509	514	core promoter element around -30 of transcription start
CsDof64	TATA-box	510	514	core promoter element around -30 of transcription start
CsDof64	TATA-box	560	566	core promoter element around -30 of transcription start
CsDof64	TATA-box	561	565	core promoter element around -30 of transcription start
CsDof64	TATA-box	1383	1390	core promoter element around -30 of transcription start
CsDof64	TATA-box	1384	1390	core promoter element around -30 of transcription start
CsDof64	TATA-box	1385	1390	core promoter element around -30 of transcription start
CsDof64	TATA-box	1386	1390	core promoter element around -30 of transcription start
CsDof64	TATA-box	1601	1605	core promoter element around -30 of transcription start
CsDof64	TATA-box	1662	1668	core promoter element around -30 of transcription start
CsDof64	TATA-box	1663	1667	core promoter element around -30 of transcription start
CsDof64	TATA-box	1707	1713	core promoter element around -30 of transcription start
CsDof64	TATA-box	1708	1713	core promoter element around -30 of transcription start
CsDof64	TATA-box	1709	1713	core promoter element around -30 of transcription start
CsDof64	TATA-box	1718	1727	core promoter element around -30 of transcription start
CsDof64	TATA-box	1719	1726	core promoter element around -30 of transcription start
CsDof64	TATA-box	1720	1726	core promoter element around -30 of transcription start
CsDof64	TATA-box	1721	1728	core promoter element around -30 of transcription start
CsDof64	TATA-box	1722	1728	core promoter element around -30 of transcription start
CsDof64	TATA-box	1723	1729	core promoter element around -30 of transcription start
CsDof64	TATA-box	1724	1730	core promoter element around -30 of transcription start
CsDof64	TATA-box	1725	1731	core promoter element around -30 of transcription start
CsDof64	TATA-box	1726	1730	core promoter element around -30 of transcription start
CsDof64	TATA-box	1849	1856	core promoter element around -30 of transcription start
CsDof64	TATA-box	1850	1856	core promoter element around -30 of transcription start
CsDof64	TATA-box	1851	1856	core promoter element around -30 of transcription start
CsDof64	TATA-box	1852	1856	core promoter element around -30 of transcription start

Name	Cis-element	Start position	Stop position	Function
CsDof64	TATA-box	1864	1872	core promoter element around -30 of transcription start
CsDof64	HD-Zip 1	1780	1788.5	element involved in differentiation of the palisade mesophyll cells
CsDof64	GARE-motif	1168	1175	gibberellin-responsive element
CsDof64	GT1-motif	834	840	light responsive element
CsDof64	Sp1	542	548	light responsive element
CsDof64	MBS	537	543	MYB binding site involved in drought-inducibility
CsDof64	MBS	599	605	MYB binding site involved in drought-inducibility
CsDof64	MBS	710	716	MYB binding site involved in drought-inducibility
CsDof64	MRE	163	170	MYB binding site involved in light responsiveness
CsDof64	CCAAT-box	1253	1259	MYBHv1 binding site
CsDof64	HD-Zip 3	150	159.5	protein binding site
CsDof65	TGA-element	1771	1777	auxin-responsive element
CsDof65	ARE	770	776	cis-acting regulatory element essential for the anaerobic induction
CsDof65	ARE	953	959	cis-acting regulatory element essential for the anaerobic induction
CsDof65	ARE	1483	1489	cis-acting regulatory element essential for the anaerobic induction
CsDof65	ARE	1666	1672	cis-acting regulatory element essential for the anaerobic induction
CsDof65	TGACG-motif	31	36	cis-acting regulatory element involved in the MeJA-responsiveness
CsDof65	TGACG-motif	1990	1995	cis-acting regulatory element involved in the MeJA-responsiveness
CsDof65	CGTCA-motif	31	36	cis-acting regulatory element involved in the MeJA-responsiveness
CsDof65	CGTCA-motif	1990	1995	cis-acting regulatory element involved in the MeJA-responsiveness
CsDof65	O2-site	914	923	cis-acting regulatory element involved in zein metabolism regulation
CsDof65	O2-site	1627	1636	cis-acting regulatory element involved in zein metabolism regulation
CsDof65	CAAT-box	5	10	common cis-acting element in promoter and enhancer regions
CsDof65	CAAT-box	19	24	common cis-acting element in promoter and enhancer regions
CsDof65	CAAT-box	224	229	common cis-acting element in promoter and enhancer regions
CsDof65	CAAT-box	591	596	common cis-acting element in promoter and enhancer regions
CsDof65	CAAT-box	616	621	common cis-acting element in promoter and enhancer regions
CsDof65	CAAT-box	708	713	common cis-acting element in promoter and enhancer regions
CsDof65	CAAT-box	715	720	common cis-acting element in promoter and enhancer regions
CsDof65	CAAT-box	1304	1309	common cis-acting element in promoter and enhancer regions
CsDof65	CAAT-box	1329	1334	common cis-acting element in promoter and enhancer regions
CsDof65	CAAT-box	1421	1426	common cis-acting element in promoter and enhancer regions
CsDof65	CAAT-box	1428	1433	common cis-acting element in promoter and enhancer regions
CsDof65	TATA-box	119	123	core promoter element around -30 of transcription start
CsDof65	TATA-box	134	139	core promoter element around -30 of transcription start
CsDof65	TATA-box	135	139	core promoter element around -30 of transcription start
CsDof65	TATA-box	234	241	core promoter element around -30 of transcription start
CsDof65	TATA-box	235	241	core promoter element around -30 of transcription start
CsDof65	TATA-box	236	241	core promoter element around -30 of transcription start
CsDof65	TATA-box	237	241	core promoter element around -30 of transcription start
CsDof65	TATA-box	247	253	core promoter element around -30 of transcription start
CsDof65	TATA-box	248	254	core promoter element around -30 of transcription start
CsDof65	TATA-box	249	255	core promoter element around -30 of transcription start
CsDof65	TATA-box	250	254	core promoter element around -30 of transcription start
CsDof65	TATA-box	300	306	core promoter element around -30 of transcription start
CsDof65	TATA-box	301	305	core promoter element around -30 of transcription start
CsDof65	TATA-box	570	577	core promoter element around -30 of transcription start
CsDof65	TATA-box	573	579	core promoter element around -30 of transcription start
CsDof65	TATA-box	575	579	core promoter element around -30 of transcription start
CsDof65	TATA-box	600	606	core promoter element around -30 of transcription start
CsDof65	TATA-box	601	607	core promoter element around -30 of transcription start

Name	Cis-element	Start position	Stop position	Function
CsDof65	TATA-box	603	607	core promoter element around -30 of transcription start
CsDof65	TATA-box	644	648	core promoter element around -30 of transcription start
CsDof65	TATA-box	687	694	core promoter element around -30 of transcription start
CsDof65	TATA-box	688	694	core promoter element around -30 of transcription start
CsDof65	TATA-box	689	694	core promoter element around -30 of transcription start
CsDof65	TATA-box	690	694	core promoter element around -30 of transcription start
CsDof65	TATA-box	1013	1019	core promoter element around -30 of transcription start
CsDof65	TATA-box	1014	1018	core promoter element around -30 of transcription start
CsDof65	TATA-box	1283	1290	core promoter element around -30 of transcription start
CsDof65	TATA-box	1286	1292	core promoter element around -30 of transcription start
CsDof65	TATA-box	1288	1292	core promoter element around -30 of transcription start
CsDof65	TATA-box	1313	1319	core promoter element around -30 of transcription start
CsDof65	TATA-box	1314	1320	core promoter element around -30 of transcription start
CsDof65	TATA-box	1316	1320	core promoter element around -30 of transcription start
CsDof65	TATA-box	1357	1361	core promoter element around -30 of transcription start
CsDof65	TATA-box	1400	1407	core promoter element around -30 of transcription start
CsDof65	TATA-box	1401	1407	core promoter element around -30 of transcription start
CsDof65	TATA-box	1402	1407	core promoter element around -30 of transcription start
CsDof65	TATA-box	1403	1407	core promoter element around -30 of transcription start
CsDof65	TATA-box	1982	1988	core promoter element around -30 of transcription start
CsDof65	TATA-box	1983	1988	core promoter element around -30 of transcription start
CsDof65	TATA-box	1984	1988	core promoter element around -30 of transcription start
CsDof65	AT-rich sequence	252	261	element for maximal elicitor-mediated activation (2copies)
CsDof65	HD-Zip 1	165	173.5	element involved in differentiation of the palisade mesophyll cells
CsDof65	MBS	741	747	MYB binding site involved in drought-inducibility
CsDof65	MBS	1454	1460	MYB binding site involved in drought-inducibility
CsDof66	ABRE	76	81	abscisic acid responsiveness
CsDof66	ABRE	1206	1211	abscisic acid responsiveness
CsDof66	ARE	319	325	cis-acting regulatory element essential for the anaerobic induction
CsDof66	ARE	1049	1055	cis-acting regulatory element essential for the anaerobic induction
CsDof66	G-Box	1205	1211	cis-acting regulatory element involved in light responsiveness
CsDof66	G-box	76	82	cis-acting regulatory element involved in light responsiveness
CsDof66	TGACG-motif	898	903	cis-acting regulatory element involved in the MeJA-responsiveness
CsDof66	CGTCA-motif	898	903	cis-acting regulatory element involved in the MeJA-responsiveness
CsDof66	O2-site	1985	1994	cis-acting regulatory element involved in zein metabolism regulation
CsDof66	CAT-box	1335	1341	cis-acting regulatory element related to meristem expression
CsDof66	CAT-box	1493	1499	cis-acting regulatory element related to meristem expression
CsDof66	CAAT-box	47	52	common cis-acting element in promoter and enhancer regions
CsDof66	CAAT-box	126	131	common cis-acting element in promoter and enhancer regions
CsDof66	CAAT-box	132	137	common cis-acting element in promoter and enhancer regions
CsDof66	CAAT-box	646	651	common cis-acting element in promoter and enhancer regions
CsDof66	CAAT-box	666	671	common cis-acting element in promoter and enhancer regions
CsDof66	CAAT-box	703	708	common cis-acting element in promoter and enhancer regions
CsDof66	CAAT-box	816	821	common cis-acting element in promoter and enhancer regions
CsDof66	CAAT-box	823	828	common cis-acting element in promoter and enhancer regions
CsDof66	CAAT-box	868	873	common cis-acting element in promoter and enhancer regions
CsDof66	CAAT-box	903	908	common cis-acting element in promoter and enhancer regions
CsDof66	CAAT-box	1009	1014	common cis-acting element in promoter and enhancer regions
CsDof66	CAAT-box	1039	1044	common cis-acting element in promoter and enhancer regions
CsDof66	CAAT-box	1274	1279	common cis-acting element in promoter and enhancer regions
CsDof66	CAAT-box	1287	1292	common cis-acting element in promoter and enhancer regions

Name	Cis-element	Start position	Stop position	Function
CsDof66	CAAT-box	1580	1585	common cis-acting element in promoter and enhancer regions
CsDof66	TATA-box	218	224	core promoter element around -30 of transcription start
CsDof66	TATA-box	219	225	core promoter element around -30 of transcription start
CsDof66	TATA-box	220	226	core promoter element around -30 of transcription start
CsDof66	TATA-box	221	225	core promoter element around -30 of transcription start
CsDof66	TATA-box	324	333	core promoter element around -30 of transcription start
CsDof66	TATA-box	325	332	core promoter element around -30 of transcription start
CsDof66	TATA-box	326	332	core promoter element around -30 of transcription start
CsDof66	TATA-box	327	334	core promoter element around -30 of transcription start
CsDof66	TATA-box	328	334	core promoter element around -30 of transcription start
CsDof66	TATA-box	330	334	core promoter element around -30 of transcription start
CsDof66	TATA-box	373	380	core promoter element around -30 of transcription start
CsDof66	TATA-box	422	430	core promoter element around -30 of transcription start
CsDof66	TATA-box	445	449	core promoter element around -30 of transcription start
CsDof66	TATA-box	572	578	core promoter element around -30 of transcription start
CsDof66	TATA-box	573	577	core promoter element around -30 of transcription start
CsDof66	TATA-box	618	625	core promoter element around -30 of transcription start
CsDof66	TATA-box	619	625	core promoter element around -30 of transcription start
CsDof66	TATA-box	620	627	core promoter element around -30 of transcription start
CsDof66	TATA-box	621	627	core promoter element around -30 of transcription start
CsDof66	TATA-box	622	628	core promoter element around -30 of transcription start
CsDof66	TATA-box	623	629	core promoter element around -30 of transcription start
CsDof66	TATA-box	624	630	core promoter element around -30 of transcription start
CsDof66	TATA-box	625	631	core promoter element around -30 of transcription start
CsDof66	TATA-box	626	632	core promoter element around -30 of transcription start
CsDof66	TATA-box	627	633	core promoter element around -30 of transcription start
CsDof66	TATA-box	629	633	core promoter element around -30 of transcription start
CsDof66	TATA-box	724	730	core promoter element around -30 of transcription start
CsDof66	TATA-box	725	731	core promoter element around -30 of transcription start
CsDof66	TATA-box	726	730	core promoter element around -30 of transcription start
CsDof66	TATA-box	885	891	core promoter element around -30 of transcription start
CsDof66	TATA-box	886	892	core promoter element around -30 of transcription start
CsDof66	TATA-box	887	893	core promoter element around -30 of transcription start
CsDof66	TATA-box	888	894	core promoter element around -30 of transcription start
CsDof66	TATA-box	889	895	core promoter element around -30 of transcription start
CsDof66	TATA-box	890	896	core promoter element around -30 of transcription start
CsDof66	TATA-box	891	895	core promoter element around -30 of transcription start
CsDof66	TATA-box	913	919	core promoter element around -30 of transcription start
CsDof66	TATA-box	914	918	core promoter element around -30 of transcription start
CsDof66	TATA-box	1020	1028	core promoter element around -30 of transcription start
CsDof66	TATA-box	1023	1030	core promoter element around -30 of transcription start
CsDof66	TATA-box	1024	1030	core promoter element around -30 of transcription start
CsDof66	TATA-box	1025	1031	core promoter element around -30 of transcription start
CsDof66	TATA-box	1026	1030	core promoter element around -30 of transcription start
CsDof66	TATA-box	1084	1090	core promoter element around -30 of transcription start
CsDof66	TATA-box	1085	1091	core promoter element around -30 of transcription start
CsDof66	TATA-box	1086	1090	core promoter element around -30 of transcription start
CsDof66	TATA-box	1129	1135	core promoter element around -30 of transcription start
CsDof66	TATA-box	1131	1137	core promoter element around -30 of transcription start
CsDof66	TATA-box	1132	1138	core promoter element around -30 of transcription start
CsDof66	TATA-box	1133	1137	core promoter element around -30 of transcription start

Name	Cis-element	Start position	Stop position	Function
CsDof66	TATA-box	1159	1165	core promoter element around -30 of transcription start
CsDof66	TATA-box	1160	1166	core promoter element around -30 of transcription start
CsDof66	TATA-box	1162	1166	core promoter element around -30 of transcription start
CsDof66	TATA-box	1175	1179	core promoter element around -30 of transcription start
CsDof66	TATA-box	1195	1204	core promoter element around -30 of transcription start
CsDof66	TATA-box	1196	1202	core promoter element around -30 of transcription start
CsDof66	TATA-box	1197	1201	core promoter element around -30 of transcription start
CsDof66	TATA-box	1229	1238	core promoter element around -30 of transcription start
CsDof66	TATA-box	1230	1236	core promoter element around -30 of transcription start
CsDof66	TATA-box	1231	1235	core promoter element around -30 of transcription start
CsDof66	TATA-box	1268	1274	core promoter element around -30 of transcription start
CsDof66	TATA-box	1269	1273	core promoter element around -30 of transcription start
CsDof66	TATA-box	1310	1317	core promoter element around -30 of transcription start
CsDof66	TATA-box	1311	1317	core promoter element around -30 of transcription start
CsDof66	TATA-box	1312	1317	core promoter element around -30 of transcription start
CsDof66	TATA-box	1313	1317	core promoter element around -30 of transcription start
CsDof66	TATA-box	1404	1410	core promoter element around -30 of transcription start
CsDof66	TATA-box	1405	1411	core promoter element around -30 of transcription start
CsDof66	TATA-box	1406	1412	core promoter element around -30 of transcription start
CsDof66	TATA-box	1407	1413	core promoter element around -30 of transcription start
CsDof66	TATA-box	1408	1412	core promoter element around -30 of transcription start
CsDof66	TATA-box	1410	1419	core promoter element around -30 of transcription start
CsDof66	TATA-box	1411	1418	core promoter element around -30 of transcription start
CsDof66	TATA-box	1412	1418	core promoter element around -30 of transcription start
CsDof66	TATA-box	1413	1420	core promoter element around -30 of transcription start
CsDof66	TATA-box	1414	1420	core promoter element around -30 of transcription start
CsDof66	TATA-box	1415	1421	core promoter element around -30 of transcription start
CsDof66	TATA-box	1416	1420	core promoter element around -30 of transcription start
CsDof66	TATA-box	1433	1437	core promoter element around -30 of transcription start
CsDof66	TATA-box	1608	1612	core promoter element around -30 of transcription start
CsDof66	TATA-box	1703	1709	core promoter element around -30 of transcription start
CsDof66	TATA-box	1704	1710	core promoter element around -30 of transcription start
CsDof66	TATA-box	1706	1710	core promoter element around -30 of transcription start
CsDof66	TATA-box	1711	1717	core promoter element around -30 of transcription start
CsDof66	TATA-box	1712	1718	core promoter element around -30 of transcription start
CsDof66	TATA-box	1713	1717	core promoter element around -30 of transcription start
CsDof66	TATA-box	1735	1743	core promoter element around -30 of transcription start
CsDof66	TATA-box	1740	1746	core promoter element around -30 of transcription start
CsDof66	TATA-box	1741	1747	core promoter element around -30 of transcription start
CsDof66	TATA-box	1742	1748	core promoter element around -30 of transcription start
CsDof66	TATA-box	1743	1747	core promoter element around -30 of transcription start
CsDof66	TATA-box	1772	1778	core promoter element around -30 of transcription start
CsDof66	TATA-box	1773	1779	core promoter element around -30 of transcription start
CsDof66	TATA-box	1774	1778	core promoter element around -30 of transcription start
CsDof66	TATA-box	1785	1789	core promoter element around -30 of transcription start
CsDof66	TATA-box	1793	1799	core promoter element around -30 of transcription start
CsDof66	TATA-box	1794	1798	core promoter element around -30 of transcription start
CsDof66	TATA-box	1805	1811	core promoter element around -30 of transcription start
CsDof66	TATA-box	1806	1812	core promoter element around -30 of transcription start
CsDof66	TATA-box	1807	1813	core promoter element around -30 of transcription start
CsDof66	TATA-box	1808	1814	core promoter element around -30 of transcription start

Name	Cis-element	Start position	Stop position	Function
CsDof66	TATA-box	1809	1815	core promoter element around -30 of transcription start
CsDof66	TATA-box	1810	1814	core promoter element around -30 of transcription start
CsDof66	TATA-box	1820	1829	core promoter element around -30 of transcription start
CsDof66	TATA-box	1821	1828	core promoter element around -30 of transcription start
CsDof66	TATA-box	1822	1828	core promoter element around -30 of transcription start
CsDof66	TATA-box	1823	1830	core promoter element around -30 of transcription start
CsDof66	TATA-box	1824	1830	core promoter element around -30 of transcription start
CsDof66	TATA-box	1825	1831	core promoter element around -30 of transcription start
CsDof66	TATA-box	1826	1832	core promoter element around -30 of transcription start
CsDof66	TATA-box	1827	1833	core promoter element around -30 of transcription start
CsDof66	TATA-box	1828	1832	core promoter element around -30 of transcription start
CsDof66	TATA-box	1838	1842	core promoter element around -30 of transcription start
CsDof66	GARE-motif	1567	1574	gibberellin-responsive element
CsDof66	MBS	295	301	MYB binding site involved in drought-inducibility
CsDof66	MBS	1521	1527	MYB binding site involved in drought-inducibility
CsDof66	MRE	224	231	MYB binding site involved in light responsiveness
CsDof67	ABRE	1045	1050	abscisic acid responsiveness
CsDof67	ABRE	1422	1430	abscisic acid responsiveness
CsDof67	ABRE	1424	1429	abscisic acid responsiveness
CsDof67	ABRE	1651	1656	abscisic acid responsiveness
CsDof67	TATC-box	500	507	cis-acting element involved in gibberellin-responsiveness
CsDof67	A-box	573	579	cis-acting regulatory element
CsDof67	ARE	1472	1478	cis-acting regulatory element essential for the anaerobic induction
CsDof67	AuxRR-core	1186	1193	cis-acting regulatory element involved in auxin responsiveness
CsDof67	G-Box	1650	1656	cis-acting regulatory element involved in light responsiveness
CsDof67	G-box	1042	1051	cis-acting regulatory element involved in light responsiveness
CsDof67	G-box	1045	1051	cis-acting regulatory element involved in light responsiveness
CsDof67	G-box	1424	1430	cis-acting regulatory element involved in light responsiveness
CsDof67	CGTCA-motif	331	336	cis-acting regulatory element involved in the MeJA-responsiveness
CsDof67	CGTCA-motif	1400	1405	cis-acting regulatory element involved in the MeJA-responsiveness
CsDof67	TGACG-motif	331	336	cis-acting regulatory element involved in the MeJA-responsiveness
CsDof67	TGACG-motif	1400	1405	cis-acting regulatory element involved in the MeJA-responsiveness
CsDof67	O2-site	1476	1485	cis-acting regulatory element involved in zein metabolism regulation
CsDof67	CAAT-box	26	31	common cis-acting element in promoter and enhancer regions
CsDof67	CAAT-box	54	59	common cis-acting element in promoter and enhancer regions
CsDof67	CAAT-box	410	415	common cis-acting element in promoter and enhancer regions
CsDof67	CAAT-box	427	432	common cis-acting element in promoter and enhancer regions
CsDof67	CAAT-box	536	541	common cis-acting element in promoter and enhancer regions
CsDof67	CAAT-box	637	642	common cis-acting element in promoter and enhancer regions
CsDof67	CAAT-box	673	682	common cis-acting element in promoter and enhancer regions
CsDof67	CAAT-box	675	680	common cis-acting element in promoter and enhancer regions
CsDof67	CAAT-box	816	821	common cis-acting element in promoter and enhancer regions
CsDof67	CAAT-box	853	858	common cis-acting element in promoter and enhancer regions
CsDof67	CAAT-box	1010	1015	common cis-acting element in promoter and enhancer regions
CsDof67	CAAT-box	1087	1092	common cis-acting element in promoter and enhancer regions
CsDof67	CAAT-box	1329	1334	common cis-acting element in promoter and enhancer regions
CsDof67	CAAT-box	1374	1379	common cis-acting element in promoter and enhancer regions
CsDof67	CAAT-box	1475	1480	common cis-acting element in promoter and enhancer regions
CsDof67	CAAT-box	1563	1568	common cis-acting element in promoter and enhancer regions
CsDof67	CAAT-box	1607	1612	common cis-acting element in promoter and enhancer regions
CsDof67	TATA-box	469	475	core promoter element around -30 of transcription start

Name	Cis-element	Start position	Stop position	Function
CsDof67	TATA-box	470	474	core promoter element around -30 of transcription start
CsDof67	TATA-box	1018	1025	core promoter element around -30 of transcription start
CsDof67	TATA-box	1019	1025	core promoter element around -30 of transcription start
CsDof67	TATA-box	1020	1025	core promoter element around -30 of transcription start
CsDof67	TATA-box	1021	1025	core promoter element around -30 of transcription start
CsDof67	TATA-box	1138	1142	core promoter element around -30 of transcription start
CsDof67	TATA-box	1192	1196	core promoter element around -30 of transcription start
CsDof67	TATA-box	1244	1250	core promoter element around -30 of transcription start
CsDof67	TATA-box	1245	1250	core promoter element around -30 of transcription start
CsDof67	TATA-box	1246	1250	core promoter element around -30 of transcription start
CsDof67	TATA-box	1285	1290	core promoter element around -30 of transcription start
CsDof67	TATA-box	1286	1290	core promoter element around -30 of transcription start
CsDof67	TATA-box	1314	1318	core promoter element around -30 of transcription start
CsDof67	TATA-box	1620	1628	core promoter element around -30 of transcription start
CsDof67	TATA-box	1632	1639	core promoter element around -30 of transcription start
CsDof67	TATA-box	1633	1639	core promoter element around -30 of transcription start
CsDof67	TATA-box	1634	1640	core promoter element around -30 of transcription start
CsDof67	TATA-box	1635	1639	core promoter element around -30 of transcription start
CsDof67	TATA-box	1750	1754	core promoter element around -30 of transcription start
CsDof68	ABRE	13	20	abscisic acid responsiveness
CsDof68	AT-rich element	1956	1966	binding site of AT-rich DNA binding protein (ATBP-1)
CsDof68	A-box	212	218	cis-acting regulatory element
CsDof68	ARE	765	771	cis-acting regulatory element essential for the anaerobic induction
CsDof68	TGACG-motif	1651	1656	cis-acting regulatory element involved in the MeJA-responsiveness
CsDof68	CGTCA-motif	1651	1656	cis-acting regulatory element involved in the MeJA-responsiveness
CsDof68	O2-site	438	447	cis-acting regulatory element involved in zein metabolism regulation
CsDof68	CAT-box	314	320	cis-acting regulatory element related to meristem expression
CsDof68	CAAT-box	406	411	common cis-acting element in promoter and enhancer regions
CsDof68	CAAT-box	673	680	common cis-acting element in promoter and enhancer regions
CsDof68	CAAT-box	836	841	common cis-acting element in promoter and enhancer regions
CsDof68	CAAT-box	847	852	common cis-acting element in promoter and enhancer regions
CsDof68	CAAT-box	1083	1088	common cis-acting element in promoter and enhancer regions
CsDof68	CAAT-box	1204	1209	common cis-acting element in promoter and enhancer regions
CsDof68	CAAT-box	1397	1402	common cis-acting element in promoter and enhancer regions
CsDof68	CAAT-box	1442	1447	common cis-acting element in promoter and enhancer regions
CsDof68	CAAT-box	1463	1468	common cis-acting element in promoter and enhancer regions
CsDof68	CAAT-box	1634	1639	common cis-acting element in promoter and enhancer regions
CsDof68	CAAT-box	1764	1769	common cis-acting element in promoter and enhancer regions
CsDof68	CAAT-box	1972	1977	common cis-acting element in promoter and enhancer regions
CsDof68	TATA-box	218	222	core promoter element around -30 of transcription start
CsDof68	TATA-box	355	361	core promoter element around -30 of transcription start
CsDof68	TATA-box	356	362	core promoter element around -30 of transcription start
CsDof68	TATA-box	357	363	core promoter element around -30 of transcription start
CsDof68	TATA-box	358	364	core promoter element around -30 of transcription start
CsDof68	TATA-box	359	363	core promoter element around -30 of transcription start
CsDof68	TATA-box	379	383	core promoter element around -30 of transcription start
CsDof68	TATA-box	384	390	core promoter element around -30 of transcription start
CsDof68	TATA-box	385	389	core promoter element around -30 of transcription start
CsDof68	TATA-box	490	494	core promoter element around -30 of transcription start
CsDof68	TATA-box	699	706	core promoter element around -30 of transcription start
CsDof68	TATA-box	700	706	core promoter element around -30 of transcription start

Name	Cis-element	Start position	Stop position	Function
CsDof68	TATA-box	701	706	core promoter element around -30 of transcription start
CsDof68	TATA-box	702	706	core promoter element around -30 of transcription start
CsDof68	TATA-box	855	861	core promoter element around -30 of transcription start
CsDof68	TATA-box	856	861	core promoter element around -30 of transcription start
CsDof68	TATA-box	857	861	core promoter element around -30 of transcription start
CsDof68	TATA-box	870	876	core promoter element around -30 of transcription start
CsDof68	TATA-box	871	877	core promoter element around -30 of transcription start
CsDof68	TATA-box	872	876	core promoter element around -30 of transcription start
CsDof68	TATA-box	900	904	core promoter element around -30 of transcription start
CsDof68	TATA-box	913	919	core promoter element around -30 of transcription start
CsDof68	TATA-box	914	920	core promoter element around -30 of transcription start
CsDof68	TATA-box	915	919	core promoter element around -30 of transcription start
CsDof68	TATA-box	924	933	core promoter element around -30 of transcription start
CsDof68	TATA-box	930	934	core promoter element around -30 of transcription start
CsDof68	TATA-box	1338	1342	core promoter element around -30 of transcription start
CsDof68	TATA-box	1348	1354	core promoter element around -30 of transcription start
CsDof68	TATA-box	1349	1353	core promoter element around -30 of transcription start
CsDof68	TATA-box	1446	1450	core promoter element around -30 of transcription start
CsDof68	TATA-box	1515	1521	core promoter element around -30 of transcription start
CsDof68	TATA-box	1516	1522	core promoter element around -30 of transcription start
CsDof68	TATA-box	1517	1523	core promoter element around -30 of transcription start
CsDof68	TATA-box	1518	1522	core promoter element around -30 of transcription start
CsDof68	TATA-box	1523	1529	core promoter element around -30 of transcription start
CsDof68	TATA-box	1524	1529	core promoter element around -30 of transcription start
CsDof68	TATA-box	1525	1529	core promoter element around -30 of transcription start
CsDof68	TATA-box	1552	1556	core promoter element around -30 of transcription start
CsDof68	TATA-box	1677	1681	core promoter element around -30 of transcription start
CsDof68	TATA-box	1746	1753	core promoter element around -30 of transcription start
CsDof68	TATA-box	1747	1753	core promoter element around -30 of transcription start
CsDof68	TATA-box	1748	1754	core promoter element around -30 of transcription start
CsDof68	TATA-box	1749	1753	core promoter element around -30 of transcription start
CsDof68	TATA-box	1773	1780	core promoter element around -30 of transcription start
CsDof68	TATA-box	1774	1780	core promoter element around -30 of transcription start
CsDof68	TATA-box	1775	1780	core promoter element around -30 of transcription start
CsDof68	TATA-box	1776	1780	core promoter element around -30 of transcription start
CsDof68	TATA-box	1826	1830	core promoter element around -30 of transcription start
CsDof68	TATA-box	1857	1863	core promoter element around -30 of transcription start
CsDof68	TATA-box	1858	1862	core promoter element around -30 of transcription start
CsDof68	TATA-box	1863	1867	core promoter element around -30 of transcription start
CsDof68	TATA-box	1873	1879	core promoter element around -30 of transcription start
CsDof68	TATA-box	1875	1879	core promoter element around -30 of transcription start
CsDof68	TATA-box	1901	1907	core promoter element around -30 of transcription start
CsDof68	TATA-box	1902	1914	core promoter element around -30 of transcription start
CsDof68	TATA-box	1903	1909	core promoter element around -30 of transcription start
CsDof68	TATA-box	1904	1910	core promoter element around -30 of transcription start
CsDof68	TATA-box	1905	1911	core promoter element around -30 of transcription start
CsDof68	TATA-box	1906	1912	core promoter element around -30 of transcription start
CsDof68	TATA-box	1907	1913	core promoter element around -30 of transcription start
CsDof68	TATA-box	1908	1912	core promoter element around -30 of transcription start
CsDof68	TATA-box	1912	1919	core promoter element around -30 of transcription start
CsDof68	TATA-box	1913	1919	core promoter element around -30 of transcription start

Name	Cis-element	Start position	Stop position	Function
CsDof68	TATA-box	1914	1921	core promoter element around -30 of transcription start
CsDof68	TATA-box	1915	1921	core promoter element around -30 of transcription start
CsDof68	TATA-box	1917	1921	core promoter element around -30 of transcription start
CsDof68	TATA-box	1934	1940	core promoter element around -30 of transcription start
CsDof68	TATA-box	1935	1941	core promoter element around -30 of transcription start
CsDof68	TATA-box	1936	1942	core promoter element around -30 of transcription start
CsDof68	TATA-box	1937	1943	core promoter element around -30 of transcription start
CsDof68	TATA-box	1939	1943	core promoter element around -30 of transcription start
CsDof68	AT-rich sequence	412	421	element for maximal elicitor-mediated activation (2copies)
CsDof68	P-box	276	283	gibberellin-responsive element
CsDof69	ABRE	328	333	abscisic acid responsiveness
CsDof69	ABRE	587	594	abscisic acid responsiveness
CsDof69	TGA-element	428	434	auxin-responsive element
CsDof69	TGA-element	584	590	auxin-responsive element
CsDof69	TGA-element	1252	1258	auxin-responsive element
CsDof69	TCA-element	1911	1920	cis-acting element involved in salicylic acid responsiveness
CsDof69	G-box	327	333	cis-acting regulatory element involved in light responsiveness
CsDof69	CGTCA-motif	1375	1380	cis-acting regulatory element involved in the MeJA-responsiveness
CsDof69	TGACG-motif	1375	1380	cis-acting regulatory element involved in the MeJA-responsiveness
CsDof69	CAT-box	720	726	cis-acting regulatory element related to meristem expression
CsDof69	GCN4_motif	1223	1230	cis-regulatory element involved in endosperm expression
CsDof69	CAAT-box	127	132	common cis-acting element in promoter and enhancer regions
CsDof69	CAAT-box	211	216	common cis-acting element in promoter and enhancer regions
CsDof69	CAAT-box	260	265	common cis-acting element in promoter and enhancer regions
CsDof69	CAAT-box	510	515	common cis-acting element in promoter and enhancer regions
CsDof69	CAAT-box	779	784	common cis-acting element in promoter and enhancer regions
CsDof69	CAAT-box	805	810	common cis-acting element in promoter and enhancer regions
CsDof69	CAAT-box	810	815	common cis-acting element in promoter and enhancer regions
CsDof69	CAAT-box	898	903	common cis-acting element in promoter and enhancer regions
CsDof69	CAAT-box	945	950	common cis-acting element in promoter and enhancer regions
CsDof69	CAAT-box	948	953	common cis-acting element in promoter and enhancer regions
CsDof69	CAAT-box	1080	1085	common cis-acting element in promoter and enhancer regions
CsDof69	CAAT-box	1283	1288	common cis-acting element in promoter and enhancer regions
CsDof69	CAAT-box	1515	1520	common cis-acting element in promoter and enhancer regions
CsDof69	CAAT-box	1697	1702	common cis-acting element in promoter and enhancer regions
CsDof69	CAAT-box	1763	1768	common cis-acting element in promoter and enhancer regions
CsDof69	CAAT-box	1810	1815	common cis-acting element in promoter and enhancer regions
CsDof69	CAAT-box	1872	1877	common cis-acting element in promoter and enhancer regions
CsDof69	CAAT-box	1932	1937	common cis-acting element in promoter and enhancer regions
CsDof69	TATA-box	11	17	core promoter element around -30 of transcription start
CsDof69	TATA-box	13	17	core promoter element around -30 of transcription start
CsDof69	TATA-box	24	28	core promoter element around -30 of transcription start
CsDof69	TATA-box	55	60	core promoter element around -30 of transcription start
CsDof69	TATA-box	56	60	core promoter element around -30 of transcription start
CsDof69	TATA-box	81	87	core promoter element around -30 of transcription start
CsDof69	TATA-box	82	86	core promoter element around -30 of transcription start
CsDof69	TATA-box	105	109	core promoter element around -30 of transcription start
CsDof69	TATA-box	201	207	core promoter element around -30 of transcription start
CsDof69	TATA-box	202	207	core promoter element around -30 of transcription start
CsDof69	TATA-box	203	207	core promoter element around -30 of transcription start
CsDof69	TATA-box	311	317	core promoter element around -30 of transcription start

Name	Cis-element	Start position	Stop position	Function
CsDof69	TATA-box	312	318	core promoter element around -30 of transcription start
CsDof69	TATA-box	314	318	core promoter element around -30 of transcription start
CsDof69	TATA-box	396	402	core promoter element around -30 of transcription start
CsDof69	TATA-box	397	402	core promoter element around -30 of transcription start
CsDof69	TATA-box	398	402	core promoter element around -30 of transcription start
CsDof69	TATA-box	432	439	core promoter element around -30 of transcription start
CsDof69	TATA-box	433	439	core promoter element around -30 of transcription start
CsDof69	TATA-box	434	439	core promoter element around -30 of transcription start
CsDof69	TATA-box	435	439	core promoter element around -30 of transcription start
CsDof69	TATA-box	611	615	core promoter element around -30 of transcription start
CsDof69	TATA-box	833	839	core promoter element around -30 of transcription start
CsDof69	TATA-box	834	840	core promoter element around -30 of transcription start
CsDof69	TATA-box	835	841	core promoter element around -30 of transcription start
CsDof69	TATA-box	836	842	core promoter element around -30 of transcription start
CsDof69	TATA-box	837	843	core promoter element around -30 of transcription start
CsDof69	TATA-box	839	843	core promoter element around -30 of transcription start
CsDof69	TATA-box	983	992	core promoter element around -30 of transcription start
CsDof69	TATA-box	984	990	core promoter element around -30 of transcription start
CsDof69	TATA-box	985	991	core promoter element around -30 of transcription start
CsDof69	TATA-box	986	992	core promoter element around -30 of transcription start
CsDof69	TATA-box	987	993	core promoter element around -30 of transcription start
CsDof69	TATA-box	988	994	core promoter element around -30 of transcription start
CsDof69	TATA-box	989	993	core promoter element around -30 of transcription start
CsDof69	TATA-box	1039	1045	core promoter element around -30 of transcription start
CsDof69	TATA-box	1041	1045	core promoter element around -30 of transcription start
CsDof69	TATA-box	1204	1211	core promoter element around -30 of transcription start
CsDof69	TATA-box	1205	1211	core promoter element around -30 of transcription start
CsDof69	TATA-box	1206	1211	core promoter element around -30 of transcription start
CsDof69	TATA-box	1207	1211	core promoter element around -30 of transcription start
CsDof69	TATA-box	1291	1297	core promoter element around -30 of transcription start
CsDof69	TATA-box	1292	1298	core promoter element around -30 of transcription start
CsDof69	TATA-box	1293	1297	core promoter element around -30 of transcription start
CsDof69	TATA-box	1310	1316	core promoter element around -30 of transcription start
CsDof69	TATA-box	1311	1315	core promoter element around -30 of transcription start
CsDof69	TATA-box	1551	1555	core promoter element around -30 of transcription start
CsDof69	TATA-box	1626	1630	core promoter element around -30 of transcription start
CsDof69	TATA-box	1639	1645	core promoter element around -30 of transcription start
CsDof69	TATA-box	1640	1644	core promoter element around -30 of transcription start
CsDof69	TATA-box	1643	1649	core promoter element around -30 of transcription start
CsDof69	TATA-box	1644	1651	core promoter element around -30 of transcription start
CsDof69	TATA-box	1645	1651	core promoter element around -30 of transcription start
CsDof69	TATA-box	1646	1652	core promoter element around -30 of transcription start
CsDof69	TATA-box	1647	1651	core promoter element around -30 of transcription start
CsDof69	TATA-box	1668	1673	core promoter element around -30 of transcription start
CsDof69	TATA-box	1669	1673	core promoter element around -30 of transcription start
CsDof69	TATA-box	1751	1756	core promoter element around -30 of transcription start
CsDof69	TATA-box	1752	1756	core promoter element around -30 of transcription start
CsDof69	TATA-box	1755	1761	core promoter element around -30 of transcription start
CsDof69	TATA-box	1756	1761	core promoter element around -30 of transcription start
CsDof69	TATA-box	1757	1761	core promoter element around -30 of transcription start
CsDof69	TATA-box	1897	1904	core promoter element around -30 of transcription start

Name	Cis-element	Start position	Stop position	Function
CsDof69	GT1-motif	683	689	light responsive element
CsDof69	WUN-motif	816	825	wound-responsive element
CsDof70	ABRE	600	605	abscisic acid responsiveness
CsDof70	ABRE	1434	1440	abscisic acid responsiveness
CsDof70	ABRE	1435	1440	abscisic acid responsiveness
CsDof70	ABRE	1864	1874	abscisic acid responsiveness
CsDof70	ABRE	1866	1871	abscisic acid responsiveness
CsDof70	LTR	866	872	cis-acting element involved in low-temperature responsiveness
CsDof70	ARE	632	638	cis-acting regulatory element essential for the anaerobic induction
CsDof70	ARE	724	730	cis-acting regulatory element essential for the anaerobic induction
CsDof70	ARE	855	861	cis-acting regulatory element essential for the anaerobic induction
CsDof70	G-Box	599	605	cis-acting regulatory element involved in light responsiveness
CsDof70	G-Box	1434	1440	cis-acting regulatory element involved in light responsiveness
CsDof70	G-Box	1866	1872	cis-acting regulatory element involved in light responsiveness
CsDof70	G-box	1433	1441	cis-acting regulatory element involved in light responsiveness
CsDof70	G-box	1434	1440	cis-acting regulatory element involved in light responsiveness
CsDof70	CGTCA-motif	1212	1217	cis-acting regulatory element involved in the MeJA-responsiveness
CsDof70	TGACG-motif	1212	1217	cis-acting regulatory element involved in the MeJA-responsiveness
CsDof70	CAT-box	1139	1145	cis-acting regulatory element related to meristem expression
CsDof70	CAAT-box	658	663	common cis-acting element in promoter and enhancer regions
CsDof70	CAAT-box	668	673	common cis-acting element in promoter and enhancer regions
CsDof70	CAAT-box	702	707	common cis-acting element in promoter and enhancer regions
CsDof70	CAAT-box	910	915	common cis-acting element in promoter and enhancer regions
CsDof70	CAAT-box	1197	1202	common cis-acting element in promoter and enhancer regions
CsDof70	CAAT-box	1294	1299	common cis-acting element in promoter and enhancer regions
CsDof70	CAAT-box	1351	1356	common cis-acting element in promoter and enhancer regions
CsDof70	CAAT-box	1399	1404	common cis-acting element in promoter and enhancer regions
CsDof70	CAAT-box	1458	1463	common cis-acting element in promoter and enhancer regions
CsDof70	CAAT-box	1655	1660	common cis-acting element in promoter and enhancer regions
CsDof70	CAAT-box	1660	1665	common cis-acting element in promoter and enhancer regions
CsDof70	CAAT-box	1944	1949	common cis-acting element in promoter and enhancer regions
CsDof70	TATA-box	691	695	core promoter element around -30 of transcription start
CsDof70	TATA-box	885	891	core promoter element around -30 of transcription start
CsDof70	TATA-box	886	890	core promoter element around -30 of transcription start
CsDof70	TATA-box	905	910	core promoter element around -30 of transcription start
CsDof70	TATA-box	906	910	core promoter element around -30 of transcription start
CsDof70	TATA-box	998	1002	core promoter element around -30 of transcription start
CsDof70	TATA-box	1016	1020	core promoter element around -30 of transcription start
CsDof70	TATA-box	1073	1077	core promoter element around -30 of transcription start
CsDof70	TATA-box	1192	1198	core promoter element around -30 of transcription start
CsDof70	TATA-box	1193	1198	core promoter element around -30 of transcription start
CsDof70	TATA-box	1194	1198	core promoter element around -30 of transcription start
CsDof70	TATA-box	1266	1270	core promoter element around -30 of transcription start
CsDof70	TATA-box	1314	1320	core promoter element around -30 of transcription start
CsDof70	TATA-box	1315	1321	core promoter element around -30 of transcription start
CsDof70	TATA-box	1316	1322	core promoter element around -30 of transcription start
CsDof70	TATA-box	1317	1321	core promoter element around -30 of transcription start
CsDof70	TATA-box	1337	1344	core promoter element around -30 of transcription start
CsDof70	TATA-box	1338	1344	core promoter element around -30 of transcription start
CsDof70	TATA-box	1339	1344	core promoter element around -30 of transcription start
CsDof70	TATA-box	1340	1344	core promoter element around -30 of transcription start

Name	Cis-element	Start position	Stop position	Function
CsDof70	TATA-box	1358	1366	core promoter element around -30 of transcription start
CsDof70	TATA-box	1440	1445	core promoter element around -30 of transcription start
CsDof70	TATA-box	1441	1445	core promoter element around -30 of transcription start
CsDof70	TATA-box	1452	1456	core promoter element around -30 of transcription start
CsDof70	TATA-box	1568	1577	core promoter element around -30 of transcription start
CsDof70	TATA-box	1570	1574	core promoter element around -30 of transcription start
CsDof70	TATA-box	1631	1636	core promoter element around -30 of transcription start
CsDof70	TATA-box	1632	1636	core promoter element around -30 of transcription start
CsDof70	TATA-box	1652	1656	core promoter element around -30 of transcription start
CsDof70	TATA-box	1694	1701	core promoter element around -30 of transcription start
CsDof70	TATA-box	1705	1711	core promoter element around -30 of transcription start
CsDof70	TATA-box	1706	1711	core promoter element around -30 of transcription start
CsDof70	TATA-box	1707	1711	core promoter element around -30 of transcription start
CsDof70	TATA-box	1726	1732	core promoter element around -30 of transcription start
CsDof70	TATA-box	1727	1732	core promoter element around -30 of transcription start
CsDof70	TATA-box	1728	1732	core promoter element around -30 of transcription start
CsDof70	TATA-box	1756	1760	core promoter element around -30 of transcription start
CsDof70	TATA-box	1783	1791	core promoter element around -30 of transcription start
CsDof70	TATA-box	1823	1829	core promoter element around -30 of transcription start
CsDof70	TATA-box	1825	1829	core promoter element around -30 of transcription start
CsDof70	TATA-box	1912	1920	core promoter element around -30 of transcription start
CsDof70	TATA-box	1915	1922	core promoter element around -30 of transcription start
CsDof70	TATA-box	1916	1922	core promoter element around -30 of transcription start
CsDof70	TATA-box	1917	1923	core promoter element around -30 of transcription start
CsDof70	TATA-box	1918	1922	core promoter element around -30 of transcription start
CsDof70	TATA-box	1969	1974	core promoter element around -30 of transcription start
CsDof70	TATA-box	1970	1974	core promoter element around -30 of transcription start
CsDof70	GARE-motif	550	557	gibberellin-responsive element
CsDof70	GT1-motif	589	595	light responsive element
CsDof70	CCAAT-box	1151	1157	MYBHv1 binding site
CsDof71	ABRE	535	540	abscisic acid responsiveness
CsDof71	ABRE	726	731	abscisic acid responsiveness
CsDof71	ABRE	1061	1067	abscisic acid responsiveness
CsDof71	ABRE	1062	1067	abscisic acid responsiveness
CsDof71	ABRE	1421	1427	abscisic acid responsiveness
CsDof71	ABRE	1422	1427	abscisic acid responsiveness
CsDof71	ABRE	1853	1863	abscisic acid responsiveness
CsDof71	ABRE	1855	1860	abscisic acid responsiveness
CsDof71	TC-rich repeats	912	921	cis-acting element involved in defense and stress responsiveness
CsDof71	TCA-element	86	95	cis-acting element involved in salicylic acid responsiveness
CsDof71	TCA-element	259	268	cis-acting element involved in salicylic acid responsiveness
CsDof71	ARE	425	431	cis-acting regulatory element essential for the anaerobic induction
CsDof71	ARE	559	565	cis-acting regulatory element essential for the anaerobic induction
CsDof71	ARE	880	886	cis-acting regulatory element essential for the anaerobic induction
CsDof71	ARE	899	905	cis-acting regulatory element essential for the anaerobic induction
CsDof71	circadian	246	255	cis-acting regulatory element involved in circadian control
CsDof71	G-Box	725	731	cis-acting regulatory element involved in light responsiveness
CsDof71	G-Box	1061	1067	cis-acting regulatory element involved in light responsiveness
CsDof71	G-Box	1421	1427	cis-acting regulatory element involved in light responsiveness
CsDof71	G-Box	1855	1861	cis-acting regulatory element involved in light responsiveness
CsDof71	G-box	534	540	cis-acting regulatory element involved in light responsiveness

Name	Cis-element	Start position	Stop position	Function
CsDof71	G-box	1061	1067	cis-acting regulatory element involved in light responsiveness
CsDof71	G-box	1420	1428	cis-acting regulatory element involved in light responsiveness
CsDof71	G-box	1421	1427	cis-acting regulatory element involved in light responsiveness
CsDof71	G-box	1852	1863	cis-acting regulatory element involved in light responsiveness
CsDof71	CGTCA-motif	171	176	cis-acting regulatory element involved in the MeJA-responsiveness
CsDof71	CGTCA-motif	1202	1207	cis-acting regulatory element involved in the MeJA-responsiveness
CsDof71	TGACG-motif	171	176	cis-acting regulatory element involved in the MeJA-responsiveness
CsDof71	TGACG-motif	1202	1207	cis-acting regulatory element involved in the MeJA-responsiveness
CsDof71	O2-site	540	549	cis-acting regulatory element involved in zein metabolism regulation
CsDof71	CAT-box	1118	1124	cis-acting regulatory element related to meristem expression
CsDof71	CAT-box	1136	1142	cis-acting regulatory element related to meristem expression
CsDof71	CAAT-box	158	163	common cis-acting element in promoter and enhancer regions
CsDof71	CAAT-box	294	299	common cis-acting element in promoter and enhancer regions
CsDof71	CAAT-box	399	404	common cis-acting element in promoter and enhancer regions
CsDof71	CAAT-box	456	461	common cis-acting element in promoter and enhancer regions
CsDof71	CAAT-box	468	473	common cis-acting element in promoter and enhancer regions
CsDof71	CAAT-box	616	621	common cis-acting element in promoter and enhancer regions
CsDof71	CAAT-box	751	756	common cis-acting element in promoter and enhancer regions
CsDof71	CAAT-box	761	766	common cis-acting element in promoter and enhancer regions
CsDof71	CAAT-box	795	800	common cis-acting element in promoter and enhancer regions
CsDof71	CAAT-box	954	959	common cis-acting element in promoter and enhancer regions
CsDof71	CAAT-box	1113	1118	common cis-acting element in promoter and enhancer regions
CsDof71	CAAT-box	1284	1289	common cis-acting element in promoter and enhancer regions
CsDof71	CAAT-box	1338	1343	common cis-acting element in promoter and enhancer regions
CsDof71	CAAT-box	1386	1391	common cis-acting element in promoter and enhancer regions
CsDof71	CAAT-box	1445	1450	common cis-acting element in promoter and enhancer regions
CsDof71	CAAT-box	1649	1654	common cis-acting element in promoter and enhancer regions
CsDof71	TATA-box	70	74	core promoter element around -30 of transcription start
CsDof71	TATA-box	120	126	core promoter element around -30 of transcription start
CsDof71	TATA-box	121	127	core promoter element around -30 of transcription start
CsDof71	TATA-box	122	126	core promoter element around -30 of transcription start
CsDof71	TATA-box	211	215	core promoter element around -30 of transcription start
CsDof71	TATA-box	588	594	core promoter element around -30 of transcription start
CsDof71	TATA-box	590	594	core promoter element around -30 of transcription start
CsDof71	TATA-box	742	747	core promoter element around -30 of transcription start
CsDof71	TATA-box	743	747	core promoter element around -30 of transcription start
CsDof71	TATA-box	780	789	core promoter element around -30 of transcription start
CsDof71	TATA-box	781	788	core promoter element around -30 of transcription start
CsDof71	TATA-box	782	788	core promoter element around -30 of transcription start
CsDof71	TATA-box	783	788	core promoter element around -30 of transcription start
CsDof71	TATA-box	784	788	core promoter element around -30 of transcription start
CsDof71	TATA-box	926	934	core promoter element around -30 of transcription start
CsDof71	TATA-box	949	954	core promoter element around -30 of transcription start
CsDof71	TATA-box	950	954	core promoter element around -30 of transcription start
CsDof71	TATA-box	1189	1195	core promoter element around -30 of transcription start
CsDof71	TATA-box	1190	1195	core promoter element around -30 of transcription start
CsDof71	TATA-box	1191	1195	core promoter element around -30 of transcription start
CsDof71	TATA-box	1256	1260	core promoter element around -30 of transcription start
CsDof71	TATA-box	1304	1310	core promoter element around -30 of transcription start
CsDof71	TATA-box	1305	1311	core promoter element around -30 of transcription start
CsDof71	TATA-box	1306	1312	core promoter element around -30 of transcription start

Name	Cis-element	Start position	Stop position	Function
CsDof71	TATA-box	1307	1311	core promoter element around -30 of transcription start
CsDof71	TATA-box	1327	1334	core promoter element around -30 of transcription start
CsDof71	TATA-box	1328	1334	core promoter element around -30 of transcription start
CsDof71	TATA-box	1329	1334	core promoter element around -30 of transcription start
CsDof71	TATA-box	1330	1334	core promoter element around -30 of transcription start
CsDof71	TATA-box	1345	1353	core promoter element around -30 of transcription start
CsDof71	TATA-box	1427	1432	core promoter element around -30 of transcription start
CsDof71	TATA-box	1428	1432	core promoter element around -30 of transcription start
CsDof71	TATA-box	1439	1443	core promoter element around -30 of transcription start
CsDof71	TATA-box	1620	1625	core promoter element around -30 of transcription start
CsDof71	TATA-box	1621	1625	core promoter element around -30 of transcription start
CsDof71	TATA-box	1683	1690	core promoter element around -30 of transcription start
CsDof71	TATA-box	1686	1692	core promoter element around -30 of transcription start
CsDof71	TATA-box	1688	1692	core promoter element around -30 of transcription start
CsDof71	TATA-box	1694	1700	core promoter element around -30 of transcription start
CsDof71	TATA-box	1695	1700	core promoter element around -30 of transcription start
CsDof71	TATA-box	1696	1700	core promoter element around -30 of transcription start
CsDof71	TATA-box	1715	1721	core promoter element around -30 of transcription start
CsDof71	TATA-box	1716	1721	core promoter element around -30 of transcription start
CsDof71	TATA-box	1717	1721	core promoter element around -30 of transcription start
CsDof71	TATA-box	1812	1818	core promoter element around -30 of transcription start
CsDof71	TATA-box	1814	1818	core promoter element around -30 of transcription start
CsDof71	TATA-box	1841	1848	core promoter element around -30 of transcription start
CsDof71	TATA-box	1903	1911	core promoter element around -30 of transcription start
CsDof71	TATA-box	1906	1913	core promoter element around -30 of transcription start
CsDof71	TATA-box	1907	1913	core promoter element around -30 of transcription start
CsDof71	TATA-box	1908	1914	core promoter element around -30 of transcription start
CsDof71	TATA-box	1909	1913	core promoter element around -30 of transcription start
CsDof71	TATA-box	1960	1965	core promoter element around -30 of transcription start
CsDof71	TATA-box	1961	1965	core promoter element around -30 of transcription start
CsDof71	GARE-motif	676	683	gibberellin-responsive element
CsDof71	GT1-motif	715	721	light responsive element
CsDof71	GT1-motif	1104	1110	light responsive element
CsDof71	MBS	1129	1135	MYB binding site involved in drought-inducibility
CsDof71	MBSI	907	918	MYB binding site involved in flavonoid biosynthetic genes regulation
CsDof71	CCAAT-box	1148	1154	MYBHv1 binding site
CsDof72	ABRE	701	706	abscisic acid responsiveness
CsDof72	ABRE	990	997	abscisic acid responsiveness
CsDof72	ABRE	1413	1418	abscisic acid responsiveness
CsDof72	ABRE	1852	1861	abscisic acid responsiveness
CsDof72	ABRE	1854	1859	abscisic acid responsiveness
CsDof72	LTR	959	965	cis-acting element involved in low-temperature responsiveness
CsDof72	ARE	467	473	cis-acting regulatory element essential for the anaerobic induction
CsDof72	ARE	749	755	cis-acting regulatory element essential for the anaerobic induction
CsDof72	ARE	804	810	cis-acting regulatory element essential for the anaerobic induction
CsDof72	ARE	870	876	cis-acting regulatory element essential for the anaerobic induction
CsDof72	ARE	925	931	cis-acting regulatory element essential for the anaerobic induction
CsDof72	G-box	700	706	cis-acting regulatory element involved in light responsiveness
CsDof72	G-box	1413	1419	cis-acting regulatory element involved in light responsiveness
CsDof72	G-Box	1854	1860	cis-acting regulatory element involved in light responsiveness
CsDof72	TGACG-motif	902	907	cis-acting regulatory element involved in the MeJA-responsiveness

Name	Cis-element	Start position	Stop position	Function
CsDof72	CGTCA-motif	902	907	cis-acting regulatory element involved in the MeJA-responsiveness
CsDof72	O2-site	598	607	cis-acting regulatory element involved in zein metabolism regulation
CsDof72	CAT-box	1024	1030	cis-acting regulatory element related to meristem expression
CsDof72	CAAT-box	95	100	common cis-acting element in promoter and enhancer regions
CsDof72	CAAT-box	137	142	common cis-acting element in promoter and enhancer regions
CsDof72	CAAT-box	376	381	common cis-acting element in promoter and enhancer regions
CsDof72	CAAT-box	494	499	common cis-acting element in promoter and enhancer regions
CsDof72	CAAT-box	689	694	common cis-acting element in promoter and enhancer regions
CsDof72	CAAT-box	873	878	common cis-acting element in promoter and enhancer regions
CsDof72	CAAT-box	1049	1054	common cis-acting element in promoter and enhancer regions
CsDof72	CAAT-box	1278	1283	common cis-acting element in promoter and enhancer regions
CsDof72	CAAT-box	1333	1338	common cis-acting element in promoter and enhancer regions
CsDof72	CAAT-box	1380	1385	common cis-acting element in promoter and enhancer regions
CsDof72	CAAT-box	1648	1653	common cis-acting element in promoter and enhancer regions
CsDof72	TATA-box	14	20	core promoter element around -30 of transcription start
CsDof72	TATA-box	15	19	core promoter element around -30 of transcription start
CsDof72	TATA-box	52	58	core promoter element around -30 of transcription start
CsDof72	TATA-box	53	57	core promoter element around -30 of transcription start
CsDof72	TATA-box	84	90	core promoter element around -30 of transcription start
CsDof72	TATA-box	85	90	core promoter element around -30 of transcription start
CsDof72	TATA-box	86	90	core promoter element around -30 of transcription start
CsDof72	TATA-box	100	104	core promoter element around -30 of transcription start
CsDof72	TATA-box	269	277	core promoter element around -30 of transcription start
CsDof72	TATA-box	483	487	core promoter element around -30 of transcription start
CsDof72	TATA-box	542	549	core promoter element around -30 of transcription start
CsDof72	TATA-box	611	617	core promoter element around -30 of transcription start
CsDof72	TATA-box	612	617	core promoter element around -30 of transcription start
CsDof72	TATA-box	613	617	core promoter element around -30 of transcription start
CsDof72	TATA-box	881	885	core promoter element around -30 of transcription start
CsDof72	TATA-box	916	920	core promoter element around -30 of transcription start
CsDof72	TATA-box	1177	1181	core promoter element around -30 of transcription start
CsDof72	TATA-box	1188	1197	core promoter element around -30 of transcription start
CsDof72	TATA-box	1191	1196	core promoter element around -30 of transcription start
CsDof72	TATA-box	1192	1196	core promoter element around -30 of transcription start
CsDof72	TATA-box	1250	1254	core promoter element around -30 of transcription start
CsDof72	TATA-box	1298	1304	core promoter element around -30 of transcription start
CsDof72	TATA-box	1299	1305	core promoter element around -30 of transcription start
CsDof72	TATA-box	1300	1306	core promoter element around -30 of transcription start
CsDof72	TATA-box	1301	1305	core promoter element around -30 of transcription start
CsDof72	TATA-box	1322	1329	core promoter element around -30 of transcription start
CsDof72	TATA-box	1323	1329	core promoter element around -30 of transcription start
CsDof72	TATA-box	1324	1329	core promoter element around -30 of transcription start
CsDof72	TATA-box	1325	1329	core promoter element around -30 of transcription start
CsDof72	TATA-box	1418	1424	core promoter element around -30 of transcription start
CsDof72	TATA-box	1419	1424	core promoter element around -30 of transcription start
CsDof72	TATA-box	1420	1424	core promoter element around -30 of transcription start
CsDof72	TATA-box	1429	1438	core promoter element around -30 of transcription start
CsDof72	TATA-box	1431	1435	core promoter element around -30 of transcription start
CsDof72	TATA-box	1547	1553	core promoter element around -30 of transcription start
CsDof72	TATA-box	1548	1553	core promoter element around -30 of transcription start
CsDof72	TATA-box	1549	1553	core promoter element around -30 of transcription start

Name	Cis-element	Start position	Stop position	Function
CsDof72	TATA-box	1619	1624	core promoter element around -30 of transcription start
CsDof72	TATA-box	1620	1624	core promoter element around -30 of transcription start
CsDof72	TATA-box	1682	1689	core promoter element around -30 of transcription start
CsDof72	TATA-box	1685	1691	core promoter element around -30 of transcription start
CsDof72	TATA-box	1687	1691	core promoter element around -30 of transcription start
CsDof72	TATA-box	1693	1699	core promoter element around -30 of transcription start
CsDof72	TATA-box	1694	1699	core promoter element around -30 of transcription start
CsDof72	TATA-box	1695	1699	core promoter element around -30 of transcription start
CsDof72	TATA-box	1714	1720	core promoter element around -30 of transcription start
CsDof72	TATA-box	1715	1720	core promoter element around -30 of transcription start
CsDof72	TATA-box	1716	1720	core promoter element around -30 of transcription start
CsDof72	TATA-box	1744	1748	core promoter element around -30 of transcription start
CsDof72	TATA-box	1758	1763	core promoter element around -30 of transcription start
CsDof72	TATA-box	1759	1763	core promoter element around -30 of transcription start
CsDof72	TATA-box	1811	1817	core promoter element around -30 of transcription start
CsDof72	TATA-box	1813	1817	core promoter element around -30 of transcription start
CsDof72	TATA-box	1902	1910	core promoter element around -30 of transcription start
CsDof72	TATA-box	1905	1912	core promoter element around -30 of transcription start
CsDof72	TATA-box	1906	1912	core promoter element around -30 of transcription start
CsDof72	TATA-box	1907	1913	core promoter element around -30 of transcription start
CsDof72	TATA-box	1908	1912	core promoter element around -30 of transcription start
CsDof72	TATA-box	1959	1964	core promoter element around -30 of transcription start
CsDof72	TATA-box	1960	1964	core promoter element around -30 of transcription start
CsDof72	GARE-motif	153	160	gibberellin-responsive element
CsDof72	GT1-motif	643	649	light responsive element
CsDof72	MBS	344	350	MYB binding site involved in drought-inducibility
CsDof72	MBS	575	581	MYB binding site involved in drought-inducibility
CsDof72	Box III	110	121	protein binding site
CsDof73	ABRE	1534	1540	abscisic acid responsiveness
CsDof73	ABRE	1535	1540	abscisic acid responsiveness
CsDof73	ABRE	1803	1808	abscisic acid responsiveness
CsDof73	LTR	1836	1842	cis-acting element involved in low-temperature responsiveness
CsDof73	ARE	1294	1300	cis-acting regulatory element essential for the anaerobic induction
CsDof73	ARE	1305	1311	cis-acting regulatory element essential for the anaerobic induction
CsDof73	ARE	1565	1571	cis-acting regulatory element essential for the anaerobic induction
CsDof73	ARE	1584	1590	cis-acting regulatory element essential for the anaerobic induction
CsDof73	circadian	478	487	cis-acting regulatory element involved in circadian control
CsDof73	G-box	1533	1541	cis-acting regulatory element involved in light responsiveness
CsDof73	G-box	1534	1540	cis-acting regulatory element involved in light responsiveness
CsDof73	G-Box	1534	1540	cis-acting regulatory element involved in light responsiveness
CsDof73	G-Box	1803	1809	cis-acting regulatory element involved in light responsiveness
CsDof73	O2-site	346	355	cis-acting regulatory element involved in zein metabolism regulation
CsDof73	CAAT-box	732	737	common cis-acting element in promoter and enhancer regions
CsDof73	CAAT-box	779	784	common cis-acting element in promoter and enhancer regions
CsDof73	CAAT-box	825	830	common cis-acting element in promoter and enhancer regions
CsDof73	CAAT-box	1046	1051	common cis-acting element in promoter and enhancer regions
CsDof73	CAAT-box	1227	1232	common cis-acting element in promoter and enhancer regions
CsDof73	CAAT-box	1270	1275	common cis-acting element in promoter and enhancer regions
CsDof73	CAAT-box	1288	1293	common cis-acting element in promoter and enhancer regions
CsDof73	CAAT-box	1644	1649	common cis-acting element in promoter and enhancer regions
CsDof73	CAAT-box	1650	1655	common cis-acting element in promoter and enhancer regions

Name	Cis-element	Start position	Stop position	Function
CsDof73	CAAT-box	1982	1987	common cis-acting element in promoter and enhancer regions
CsDof73	TATA-box	99	106	core promoter element around -30 of transcription start
CsDof73	TATA-box	587	593	core promoter element around -30 of transcription start
CsDof73	TATA-box	588	594	core promoter element around -30 of transcription start
CsDof73	TATA-box	589	593	core promoter element around -30 of transcription start
CsDof73	TATA-box	614	618	core promoter element around -30 of transcription start
CsDof73	TATA-box	637	643	core promoter element around -30 of transcription start
CsDof73	TATA-box	638	643	core promoter element around -30 of transcription start
CsDof73	TATA-box	639	643	core promoter element around -30 of transcription start
CsDof73	TATA-box	698	704	core promoter element around -30 of transcription start
CsDof73	TATA-box	699	705	core promoter element around -30 of transcription start
CsDof73	TATA-box	700	704	core promoter element around -30 of transcription start
CsDof73	TATA-box	920	926	core promoter element around -30 of transcription start
CsDof73	TATA-box	921	927	core promoter element around -30 of transcription start
CsDof73	TATA-box	922	926	core promoter element around -30 of transcription start
CsDof73	TATA-box	961	967	core promoter element around -30 of transcription start
CsDof73	TATA-box	962	968	core promoter element around -30 of transcription start
CsDof73	TATA-box	963	967	core promoter element around -30 of transcription start
CsDof73	TATA-box	1143	1150	core promoter element around -30 of transcription start
CsDof73	TATA-box	1144	1150	core promoter element around -30 of transcription start
CsDof73	TATA-box	1145	1150	core promoter element around -30 of transcription start
CsDof73	TATA-box	1146	1150	core promoter element around -30 of transcription start
CsDof73	TATA-box	1240	1247	core promoter element around -30 of transcription start
CsDof73	TATA-box	1241	1250	core promoter element around -30 of transcription start
CsDof73	TATA-box	1242	1247	core promoter element around -30 of transcription start
CsDof73	TATA-box	1243	1247	core promoter element around -30 of transcription start
CsDof73	TATA-box	1251	1260	core promoter element around -30 of transcription start
CsDof73	TATA-box	1252	1259	core promoter element around -30 of transcription start
CsDof73	TATA-box	1253	1259	core promoter element around -30 of transcription start
CsDof73	TATA-box	1254	1259	core promoter element around -30 of transcription start
CsDof73	TATA-box	1255	1259	core promoter element around -30 of transcription start
CsDof73	TATA-box	1264	1271	core promoter element around -30 of transcription start
CsDof73	TATA-box	1322	1329	core promoter element around -30 of transcription start
CsDof73	TATA-box	1356	1360	core promoter element around -30 of transcription start
CsDof73	TATA-box	1475	1481	core promoter element around -30 of transcription start
CsDof73	TATA-box	1476	1480	core promoter element around -30 of transcription start
CsDof73	TATA-box	1498	1504	core promoter element around -30 of transcription start
CsDof73	TATA-box	1499	1505	core promoter element around -30 of transcription start
CsDof73	TATA-box	1501	1505	core promoter element around -30 of transcription start
CsDof73	TATA-box	1593	1598	core promoter element around -30 of transcription start
CsDof73	TATA-box	1594	1598	core promoter element around -30 of transcription start
CsDof73	TATA-box	1660	1664	core promoter element around -30 of transcription start
CsDof73	TATA-box	1689	1693	core promoter element around -30 of transcription start
CsDof73	TATA-box	1789	1796	core promoter element around -30 of transcription start
CsDof73	TATA-box	1876	1882	core promoter element around -30 of transcription start
CsDof73	TATA-box	1877	1883	core promoter element around -30 of transcription start
CsDof73	TATA-box	1878	1882	core promoter element around -30 of transcription start
CsDof73	TATA-box	1942	1947	core promoter element around -30 of transcription start
CsDof73	TATA-box	1943	1947	core promoter element around -30 of transcription start
CsDof73	MBS	1309	1315	MYB binding site involved in drought-inducibility
CsDof74	ABRE	1415	1420	abscisic acid responsiveness

Name	Cis-element	Start position	Stop position	Function
CsDof74	ABRE	1852	1862	abscisic acid responsiveness
CsDof74	ABRE	1854	1859	abscisic acid responsiveness
CsDof74	TC-rich repeats	1117	1126	cis-acting element involved in defense and stress responsiveness
CsDof74	TC-rich repeats	1557	1566	cis-acting element involved in defense and stress responsiveness
CsDof74	TCA-element	266	275	cis-acting element involved in salicylic acid responsiveness
CsDof74	3ox II -like sequence	160	170	cis-acting regulatory element
CsDof74	ARE	1930	1936	cis-acting regulatory element essential for the anaerobic induction
CsDof74	G-Box	1854	1860	cis-acting regulatory element involved in light responsiveness
CsDof74	G-box	1415	1421	cis-acting regulatory element involved in light responsiveness
CsDof74	CGTCA-motif	731	736	cis-acting regulatory element involved in the MeJA-responsiveness
CsDof74	TGACG-motif	731	736	cis-acting regulatory element involved in the MeJA-responsiveness
CsDof74	CAAT-box	42	47	common cis-acting element in promoter and enhancer regions
CsDof74	CAAT-box	48	53	common cis-acting element in promoter and enhancer regions
CsDof74	CAAT-box	75	80	common cis-acting element in promoter and enhancer regions
CsDof74	CAAT-box	207	212	common cis-acting element in promoter and enhancer regions
CsDof74	CAAT-box	322	327	common cis-acting element in promoter and enhancer regions
CsDof74	CAAT-box	412	417	common cis-acting element in promoter and enhancer regions
CsDof74	CAAT-box	421	426	common cis-acting element in promoter and enhancer regions
CsDof74	CAAT-box	429	434	common cis-acting element in promoter and enhancer regions
CsDof74	CAAT-box	548	553	common cis-acting element in promoter and enhancer regions
CsDof74	CAAT-box	668	673	common cis-acting element in promoter and enhancer regions
CsDof74	CAAT-box	735	740	common cis-acting element in promoter and enhancer regions
CsDof74	CAAT-box	866	871	common cis-acting element in promoter and enhancer regions
CsDof74	CAAT-box	896	901	common cis-acting element in promoter and enhancer regions
CsDof74	CAAT-box	999	1004	common cis-acting element in promoter and enhancer regions
CsDof74	CAAT-box	1071	1076	common cis-acting element in promoter and enhancer regions
CsDof74	CAAT-box	1334	1339	common cis-acting element in promoter and enhancer regions
CsDof74	CAAT-box	1382	1387	common cis-acting element in promoter and enhancer regions
CsDof74	CAAT-box	1648	1653	common cis-acting element in promoter and enhancer regions
CsDof74	TATA-box	109	113	core promoter element around -30 of transcription start
CsDof74	TATA-box	257	263	core promoter element around -30 of transcription start
CsDof74	TATA-box	258	262	core promoter element around -30 of transcription start
CsDof74	TATA-box	701	707	core promoter element around -30 of transcription start
CsDof74	TATA-box	702	707	core promoter element around -30 of transcription start
CsDof74	TATA-box	703	707	core promoter element around -30 of transcription start
CsDof74	TATA-box	757	763	core promoter element around -30 of transcription start
CsDof74	TATA-box	758	764	core promoter element around -30 of transcription start
CsDof74	TATA-box	759	763	core promoter element around -30 of transcription start
CsDof74	TATA-box	877	881	core promoter element around -30 of transcription start
CsDof74	TATA-box	916	924	core promoter element around -30 of transcription start
CsDof74	TATA-box	919	926	core promoter element around -30 of transcription start
CsDof74	TATA-box	920	926	core promoter element around -30 of transcription start
CsDof74	TATA-box	921	927	core promoter element around -30 of transcription start
CsDof74	TATA-box	922	926	core promoter element around -30 of transcription start
CsDof74	TATA-box	1052	1059	core promoter element around -30 of transcription start
CsDof74	TATA-box	1053	1059	core promoter element around -30 of transcription start
CsDof74	TATA-box	1054	1060	core promoter element around -30 of transcription start
CsDof74	TATA-box	1055	1059	core promoter element around -30 of transcription start
CsDof74	TATA-box	1074	1080	core promoter element around -30 of transcription start
CsDof74	TATA-box	1075	1079	core promoter element around -30 of transcription start
CsDof74	TATA-box	1097	1103	core promoter element around -30 of transcription start

Name	Cis-element	Start position	Stop position	Function
CsDof74	TATA-box	1098	1103	core promoter element around -30 of transcription start
CsDof74	TATA-box	1099	1103	core promoter element around -30 of transcription start
CsDof74	TATA-box	1104	1110	core promoter element around -30 of transcription start
CsDof74	TATA-box	1105	1110	core promoter element around -30 of transcription start
CsDof74	TATA-box	1106	1110	core promoter element around -30 of transcription start
CsDof74	TATA-box	1139	1146	core promoter element around -30 of transcription start
CsDof74	TATA-box	1140	1146	core promoter element around -30 of transcription start
CsDof74	TATA-box	1141	1146	core promoter element around -30 of transcription start
CsDof74	TATA-box	1142	1146	core promoter element around -30 of transcription start
CsDof74	TATA-box	1154	1160	core promoter element around -30 of transcription start
CsDof74	TATA-box	1155	1159	core promoter element around -30 of transcription start
CsDof74	TATA-box	1162	1169	core promoter element around -30 of transcription start
CsDof74	TATA-box	1163	1169	core promoter element around -30 of transcription start
CsDof74	TATA-box	1164	1169	core promoter element around -30 of transcription start
CsDof74	TATA-box	1165	1169	core promoter element around -30 of transcription start
CsDof74	TATA-box	1175	1179	core promoter element around -30 of transcription start
CsDof74	TATA-box	1182	1188	core promoter element around -30 of transcription start
CsDof74	TATA-box	1183	1187	core promoter element around -30 of transcription start
CsDof74	TATA-box	1204	1208	core promoter element around -30 of transcription start
CsDof74	TATA-box	1226	1232	core promoter element around -30 of transcription start
CsDof74	TATA-box	1227	1233	core promoter element around -30 of transcription start
CsDof74	TATA-box	1228	1234	core promoter element around -30 of transcription start
CsDof74	TATA-box	1229	1233	core promoter element around -30 of transcription start
CsDof74	TATA-box	1323	1330	core promoter element around -30 of transcription start
CsDof74	TATA-box	1324	1330	core promoter element around -30 of transcription start
CsDof74	TATA-box	1325	1330	core promoter element around -30 of transcription start
CsDof74	TATA-box	1326	1330	core promoter element around -30 of transcription start
CsDof74	TATA-box	1420	1426	core promoter element around -30 of transcription start
CsDof74	TATA-box	1421	1426	core promoter element around -30 of transcription start
CsDof74	TATA-box	1422	1426	core promoter element around -30 of transcription start
CsDof74	TATA-box	1431	1440	core promoter element around -30 of transcription start
CsDof74	TATA-box	1433	1437	core promoter element around -30 of transcription start
CsDof74	TATA-box	1544	1553	core promoter element around -30 of transcription start
CsDof74	TATA-box	1546	1550	core promoter element around -30 of transcription start
CsDof74	TATA-box	1619	1624	core promoter element around -30 of transcription start
CsDof74	TATA-box	1620	1624	core promoter element around -30 of transcription start
CsDof74	TATA-box	1682	1689	core promoter element around -30 of transcription start
CsDof74	TATA-box	1685	1691	core promoter element around -30 of transcription start
CsDof74	TATA-box	1687	1691	core promoter element around -30 of transcription start
CsDof74	TATA-box	1693	1699	core promoter element around -30 of transcription start
CsDof74	TATA-box	1694	1699	core promoter element around -30 of transcription start
CsDof74	TATA-box	1695	1699	core promoter element around -30 of transcription start
CsDof74	TATA-box	1714	1720	core promoter element around -30 of transcription start
CsDof74	TATA-box	1715	1720	core promoter element around -30 of transcription start
CsDof74	TATA-box	1716	1720	core promoter element around -30 of transcription start
CsDof74	TATA-box	1744	1748	core promoter element around -30 of transcription start
CsDof74	TATA-box	1902	1910	core promoter element around -30 of transcription start
CsDof74	TATA-box	1905	1912	core promoter element around -30 of transcription start
CsDof74	TATA-box	1906	1912	core promoter element around -30 of transcription start
CsDof74	TATA-box	1907	1913	core promoter element around -30 of transcription start
CsDof74	TATA-box	1908	1912	core promoter element around -30 of transcription start

Name	Cis-element	Start position	Stop position	Function
CsDof74	TATA-box	1959	1965	core promoter element around -30 of transcription start
CsDof74	TATA-box	1960	1965	core promoter element around -30 of transcription start
CsDof74	TATA-box	1961	1965	core promoter element around -30 of transcription start
CsDof74	P-box	282	289	gibberellin-responsive element
CsDof74	GARE-motif	232	239	gibberellin-responsive element
CsDof74	GT1-motif	1211	1217	light responsive element
CsDof74	MBS	34	40	MYB binding site involved in drought-inducibility
CsDof75	ABRE	1689	1695	abscisic acid responsiveness
CsDof75	ABRE	1690	1695	abscisic acid responsiveness
CsDof75	ABRE	1700	1705	abscisic acid responsiveness
CsDof75	TC-rich repeats	1042	1051	cis-acting element involved in defense and stress responsiveness
CsDof75	TC-rich repeats	1081	1090	cis-acting element involved in defense and stress responsiveness
CsDof75	ARE	532	538	cis-acting regulatory element essential for the anaerobic induction
CsDof75	G-Box	1689	1695	cis-acting regulatory element involved in light responsiveness
CsDof75	G-box	1075	1084	cis-acting regulatory element involved in light responsiveness
CsDof75	G-box	1689	1695	cis-acting regulatory element involved in light responsiveness
CsDof75	G-box	1699	1705	cis-acting regulatory element involved in light responsiveness
CsDof75	CGTCA-motif	1034	1039	cis-acting regulatory element involved in the MeJA-responsiveness
CsDof75	CGTCA-motif	1698	1703	cis-acting regulatory element involved in the MeJA-responsiveness
CsDof75	TGACG-motif	1034	1039	cis-acting regulatory element involved in the MeJA-responsiveness
CsDof75	TGACG-motif	1698	1703	cis-acting regulatory element involved in the MeJA-responsiveness
CsDof75	CAAT-box	14	19	common cis-acting element in promoter and enhancer regions
CsDof75	CAAT-box	379	384	common cis-acting element in promoter and enhancer regions
CsDof75	CAAT-box	401	408	common cis-acting element in promoter and enhancer regions
CsDof75	CAAT-box	556	563	common cis-acting element in promoter and enhancer regions
CsDof75	CAAT-box	684	689	common cis-acting element in promoter and enhancer regions
CsDof75	CAAT-box	998	1003	common cis-acting element in promoter and enhancer regions
CsDof75	CAAT-box	1332	1337	common cis-acting element in promoter and enhancer regions
CsDof75	CAAT-box	1389	1394	common cis-acting element in promoter and enhancer regions
CsDof75	CAAT-box	1808	1813	common cis-acting element in promoter and enhancer regions
CsDof75	CAAT-box	1816	1821	common cis-acting element in promoter and enhancer regions
CsDof75	CAAT-box	1894	1899	common cis-acting element in promoter and enhancer regions
CsDof75	TATA-box	9	15	core promoter element around -30 of transcription start
CsDof75	TATA-box	10	16	core promoter element around -30 of transcription start
CsDof75	TATA-box	11	15	core promoter element around -30 of transcription start
CsDof75	TATA-box	19	24	core promoter element around -30 of transcription start
CsDof75	TATA-box	20	24	core promoter element around -30 of transcription start
CsDof75	TATA-box	60	66	core promoter element around -30 of transcription start
CsDof75	TATA-box	62	66	core promoter element around -30 of transcription start
CsDof75	TATA-box	77	82	core promoter element around -30 of transcription start
CsDof75	TATA-box	78	82	core promoter element around -30 of transcription start
CsDof75	TATA-box	88	95	core promoter element around -30 of transcription start
CsDof75	TATA-box	96	102	core promoter element around -30 of transcription start
CsDof75	TATA-box	97	102	core promoter element around -30 of transcription start
CsDof75	TATA-box	98	102	core promoter element around -30 of transcription start
CsDof75	TATA-box	361	369	core promoter element around -30 of transcription start
CsDof75	TATA-box	362	369	core promoter element around -30 of transcription start
CsDof75	TATA-box	363	369	core promoter element around -30 of transcription start
CsDof75	TATA-box	364	369	core promoter element around -30 of transcription start
CsDof75	TATA-box	365	369	core promoter element around -30 of transcription start
CsDof75	TATA-box	479	485	core promoter element around -30 of transcription start

Name	Cis-element	Start position	Stop position	Function
CsDof75	TATA-box	480	484	core promoter element around -30 of transcription start
CsDof75	TATA-box	514	521	core promoter element around -30 of transcription start
CsDof75	TATA-box	535	542	core promoter element around -30 of transcription start
CsDof75	TATA-box	572	578	core promoter element around -30 of transcription start
CsDof75	TATA-box	573	577	core promoter element around -30 of transcription start
CsDof75	TATA-box	594	599	core promoter element around -30 of transcription start
CsDof75	TATA-box	595	599	core promoter element around -30 of transcription start
CsDof75	TATA-box	619	625	core promoter element around -30 of transcription start
CsDof75	TATA-box	620	624	core promoter element around -30 of transcription start
CsDof75	TATA-box	622	630	core promoter element around -30 of transcription start
CsDof75	TATA-box	647	654	core promoter element around -30 of transcription start
CsDof75	TATA-box	679	685	core promoter element around -30 of transcription start
CsDof75	TATA-box	680	686	core promoter element around -30 of transcription start
CsDof75	TATA-box	681	685	core promoter element around -30 of transcription start
CsDof75	TATA-box	689	694	core promoter element around -30 of transcription start
CsDof75	TATA-box	690	694	core promoter element around -30 of transcription start
CsDof75	TATA-box	730	736	core promoter element around -30 of transcription start
CsDof75	TATA-box	732	736	core promoter element around -30 of transcription start
CsDof75	TATA-box	747	752	core promoter element around -30 of transcription start
CsDof75	TATA-box	748	752	core promoter element around -30 of transcription start
CsDof75	TATA-box	758	765	core promoter element around -30 of transcription start
CsDof75	TATA-box	766	772	core promoter element around -30 of transcription start
CsDof75	TATA-box	767	772	core promoter element around -30 of transcription start
CsDof75	TATA-box	768	772	core promoter element around -30 of transcription start
CsDof75	TATA-box	858	864	core promoter element around -30 of transcription start
CsDof75	TATA-box	859	863	core promoter element around -30 of transcription start
CsDof75	TATA-box	995	999	core promoter element around -30 of transcription start
CsDof75	TATA-box	1052	1059	core promoter element around -30 of transcription start
CsDof75	TATA-box	1053	1059	core promoter element around -30 of transcription start
CsDof75	TATA-box	1054	1059	core promoter element around -30 of transcription start
CsDof75	TATA-box	1055	1059	core promoter element around -30 of transcription start
CsDof75	TATA-box	1088	1092	core promoter element around -30 of transcription start
CsDof75	TATA-box	1112	1119	core promoter element around -30 of transcription start
CsDof75	TATA-box	1113	1119	core promoter element around -30 of transcription start
CsDof75	TATA-box	1114	1119	core promoter element around -30 of transcription start
CsDof75	TATA-box	1115	1119	core promoter element around -30 of transcription start
CsDof75	TATA-box	1137	1146	core promoter element around -30 of transcription start
CsDof75	TATA-box	1139	1145	core promoter element around -30 of transcription start
CsDof75	TATA-box	1140	1147	core promoter element around -30 of transcription start
CsDof75	TATA-box	1141	1147	core promoter element around -30 of transcription start
CsDof75	TATA-box	1142	1148	core promoter element around -30 of transcription start
CsDof75	TATA-box	1143	1147	core promoter element around -30 of transcription start
CsDof75	TATA-box	1183	1190	core promoter element around -30 of transcription start
CsDof75	TATA-box	1184	1190	core promoter element around -30 of transcription start
CsDof75	TATA-box	1185	1190	core promoter element around -30 of transcription start
CsDof75	TATA-box	1186	1190	core promoter element around -30 of transcription start
CsDof75	TATA-box	1197	1203	core promoter element around -30 of transcription start
CsDof75	TATA-box	1198	1202	core promoter element around -30 of transcription start
CsDof75	TATA-box	1267	1273	core promoter element around -30 of transcription start
CsDof75	TATA-box	1268	1274	core promoter element around -30 of transcription start
CsDof75	TATA-box	1269	1275	core promoter element around -30 of transcription start

Name	Cis-element	Start position	Stop position	Function
CsDof75	TATA-box	1270	1274	core promoter element around -30 of transcription start
CsDof75	TATA-box	1305	1311	core promoter element around -30 of transcription start
CsDof75	TATA-box	1306	1310	core promoter element around -30 of transcription start
CsDof75	TATA-box	1341	1347	core promoter element around -30 of transcription start
CsDof75	TATA-box	1342	1348	core promoter element around -30 of transcription start
CsDof75	TATA-box	1343	1349	core promoter element around -30 of transcription start
CsDof75	TATA-box	1344	1348	core promoter element around -30 of transcription start
CsDof75	TATA-box	1358	1362	core promoter element around -30 of transcription start
CsDof75	TATA-box	1410	1418	core promoter element around -30 of transcription start
CsDof75	TATA-box	1415	1421	core promoter element around -30 of transcription start
CsDof75	TATA-box	1416	1422	core promoter element around -30 of transcription start
CsDof75	TATA-box	1417	1423	core promoter element around -30 of transcription start
CsDof75	TATA-box	1418	1422	core promoter element around -30 of transcription start
CsDof75	TATA-box	1480	1485	core promoter element around -30 of transcription start
CsDof75	TATA-box	1481	1485	core promoter element around -30 of transcription start
CsDof75	TATA-box	1502	1509	core promoter element around -30 of transcription start
CsDof75	TATA-box	1664	1671	core promoter element around -30 of transcription start
CsDof75	P-box	1720	1727	gibberellin-responsive element
CsDof75	GT1-motif	899	905	light responsive element
CsDof76	ABRE	1094	1099	abscisic acid responsiveness
CsDof76	LTR	395	401	cis-acting element involved in low-temperature responsiveness
CsDof76	ARE	147	153	cis-acting regulatory element essential for the anaerobic induction
CsDof76	ARE	297	303	cis-acting regulatory element essential for the anaerobic induction
CsDof76	ARE	1945	1951	cis-acting regulatory element essential for the anaerobic induction
CsDof76	G-box	1094	1100	cis-acting regulatory element involved in light responsiveness
CsDof76	TGACG-motif	842	847	cis-acting regulatory element involved in the MeJA-responsiveness
CsDof76	TGACG-motif	897	902	cis-acting regulatory element involved in the MeJA-responsiveness
CsDof76	CGTCA-motif	842	847	cis-acting regulatory element involved in the MeJA-responsiveness
CsDof76	CGTCA-motif	897	902	cis-acting regulatory element involved in the MeJA-responsiveness
CsDof76	O2-site	983	992	cis-acting regulatory element involved in zein metabolism regulation
CsDof76	CAT-box	1136	1142	cis-acting regulatory element related to meristem expression
CsDof76	CAAT-box	121	126	common cis-acting element in promoter and enhancer regions
CsDof76	CAAT-box	183	188	common cis-acting element in promoter and enhancer regions
CsDof76	CAAT-box	199	204	common cis-acting element in promoter and enhancer regions
CsDof76	CAAT-box	214	219	common cis-acting element in promoter and enhancer regions
CsDof76	CAAT-box	229	234	common cis-acting element in promoter and enhancer regions
CsDof76	CAAT-box	437	442	common cis-acting element in promoter and enhancer regions
CsDof76	CAAT-box	473	478	common cis-acting element in promoter and enhancer regions
CsDof76	CAAT-box	1351	1356	common cis-acting element in promoter and enhancer regions
CsDof76	CAAT-box	1661	1666	common cis-acting element in promoter and enhancer regions
CsDof76	CAAT-box	1664	1669	common cis-acting element in promoter and enhancer regions
CsDof76	CAAT-box	1720	1725	common cis-acting element in promoter and enhancer regions
CsDof76	CAAT-box	1930	1935	common cis-acting element in promoter and enhancer regions
CsDof76	CAAT-box	1996	2001	common cis-acting element in promoter and enhancer regions
CsDof76	TATA-box	126	130	core promoter element around -30 of transcription start
CsDof76	TATA-box	131	138	core promoter element around -30 of transcription start
CsDof76	TATA-box	137	143	core promoter element around -30 of transcription start
CsDof76	TATA-box	138	143	core promoter element around -30 of transcription start
CsDof76	TATA-box	139	143	core promoter element around -30 of transcription start
CsDof76	TATA-box	224	233	core promoter element around -30 of transcription start
CsDof76	TATA-box	412	422	core promoter element around -30 of transcription start

Name	Cis-element	Start position	Stop position	Function
CsDof76	TATA-box	413	420	core promoter element around -30 of transcription start
CsDof76	TATA-box	414	420	core promoter element around -30 of transcription start
CsDof76	TATA-box	415	422	core promoter element around -30 of transcription start
CsDof76	TATA-box	416	422	core promoter element around -30 of transcription start
CsDof76	TATA-box	418	422	core promoter element around -30 of transcription start
CsDof76	TATA-box	580	589	core promoter element around -30 of transcription start
CsDof76	TATA-box	581	587	core promoter element around -30 of transcription start
CsDof76	TATA-box	582	589	core promoter element around -30 of transcription start
CsDof76	TATA-box	583	589	core promoter element around -30 of transcription start
CsDof76	TATA-box	585	589	core promoter element around -30 of transcription start
CsDof76	TATA-box	652	660	core promoter element around -30 of transcription start
CsDof76	TATA-box	657	663	core promoter element around -30 of transcription start
CsDof76	TATA-box	658	662	core promoter element around -30 of transcription start
CsDof76	TATA-box	913	919	core promoter element around -30 of transcription start
CsDof76	TATA-box	914	919	core promoter element around -30 of transcription start
CsDof76	TATA-box	915	919	core promoter element around -30 of transcription start
CsDof76	TATA-box	969	976	core promoter element around -30 of transcription start
CsDof76	TATA-box	1034	1043	core promoter element around -30 of transcription start
CsDof76	TATA-box	1039	1045	core promoter element around -30 of transcription start
CsDof76	TATA-box	1040	1044	core promoter element around -30 of transcription start
CsDof76	TATA-box	1086	1092	core promoter element around -30 of transcription start
CsDof76	TATA-box	1087	1093	core promoter element around -30 of transcription start
CsDof76	TATA-box	1088	1094	core promoter element around -30 of transcription start
CsDof76	TATA-box	1090	1094	core promoter element around -30 of transcription start
CsDof76	TATA-box	1100	1106	core promoter element around -30 of transcription start
CsDof76	TATA-box	1101	1105	core promoter element around -30 of transcription start
CsDof76	TATA-box	1119	1125	core promoter element around -30 of transcription start
CsDof76	TATA-box	1120	1126	core promoter element around -30 of transcription start
CsDof76	TATA-box	1121	1127	core promoter element around -30 of transcription start
CsDof76	TATA-box	1122	1126	core promoter element around -30 of transcription start
CsDof76	TATA-box	1206	1212	core promoter element around -30 of transcription start
CsDof76	TATA-box	1207	1211	core promoter element around -30 of transcription start
CsDof76	TATA-box	1215	1221	core promoter element around -30 of transcription start
CsDof76	TATA-box	1216	1222	core promoter element around -30 of transcription start
CsDof76	TATA-box	1217	1223	core promoter element around -30 of transcription start
CsDof76	TATA-box	1218	1222	core promoter element around -30 of transcription start
CsDof76	TATA-box	1250	1258	core promoter element around -30 of transcription start
CsDof76	TATA-box	1253	1258	core promoter element around -30 of transcription start
CsDof76	TATA-box	1254	1258	core promoter element around -30 of transcription start
CsDof76	TATA-box	1269	1275	core promoter element around -30 of transcription start
CsDof76	TATA-box	1270	1274	core promoter element around -30 of transcription start
CsDof76	TATA-box	1310	1314	core promoter element around -30 of transcription start
CsDof76	TATA-box	1345	1352	core promoter element around -30 of transcription start
CsDof76	TATA-box	1432	1440	core promoter element around -30 of transcription start
CsDof76	TATA-box	1438	1446	core promoter element around -30 of transcription start
CsDof76	TATA-box	1511	1517	core promoter element around -30 of transcription start
CsDof76	TATA-box	1512	1518	core promoter element around -30 of transcription start
CsDof76	TATA-box	1513	1519	core promoter element around -30 of transcription start
CsDof76	TATA-box	1514	1520	core promoter element around -30 of transcription start
CsDof76	TATA-box	1515	1521	core promoter element around -30 of transcription start
CsDof76	TATA-box	1516	1522	core promoter element around -30 of transcription start

Name	Cis-element	Start position	Stop position	Function
CsDof76	TATA-box	1517	1523	core promoter element around -30 of transcription start
CsDof76	TATA-box	1518	1524	core promoter element around -30 of transcription start
CsDof76	TATA-box	1519	1525	core promoter element around -30 of transcription start
CsDof76	TATA-box	1520	1526	core promoter element around -30 of transcription start
CsDof76	TATA-box	1522	1526	core promoter element around -30 of transcription start
CsDof76	TATA-box	1527	1533	core promoter element around -30 of transcription start
CsDof76	TATA-box	1528	1534	core promoter element around -30 of transcription start
CsDof76	TATA-box	1529	1535	core promoter element around -30 of transcription start
CsDof76	TATA-box	1530	1542	core promoter element around -30 of transcription start
CsDof76	TATA-box	1531	1537	core promoter element around -30 of transcription start
CsDof76	TATA-box	1532	1538	core promoter element around -30 of transcription start
CsDof76	TATA-box	1533	1539	core promoter element around -30 of transcription start
CsDof76	TATA-box	1534	1540	core promoter element around -30 of transcription start
CsDof76	TATA-box	1535	1541	core promoter element around -30 of transcription start
CsDof76	TATA-box	1536	1548	core promoter element around -30 of transcription start
CsDof76	TATA-box	1538	1546	core promoter element around -30 of transcription start
CsDof76	TATA-box	1539	1546	core promoter element around -30 of transcription start
CsDof76	TATA-box	1540	1546	core promoter element around -30 of transcription start
CsDof76	TATA-box	1541	1546	core promoter element around -30 of transcription start
CsDof76	TATA-box	1542	1546	core promoter element around -30 of transcription start
CsDof76	TATA-box	1667	1673	core promoter element around -30 of transcription start
CsDof76	TATA-box	1669	1675	core promoter element around -30 of transcription start
CsDof76	TATA-box	1670	1676	core promoter element around -30 of transcription start
CsDof76	TATA-box	1671	1675	core promoter element around -30 of transcription start
CsDof76	TATA-box	1675	1681	core promoter element around -30 of transcription start
CsDof76	TATA-box	1676	1682	core promoter element around -30 of transcription start
CsDof76	TATA-box	1677	1683	core promoter element around -30 of transcription start
CsDof76	TATA-box	1678	1682	core promoter element around -30 of transcription start
CsDof76	TATA-box	1726	1731	core promoter element around -30 of transcription start
CsDof76	TATA-box	1727	1731	core promoter element around -30 of transcription start
CsDof76	TATA-box	1744	1748	core promoter element around -30 of transcription start
CsDof76	TATA-box	1757	1763	core promoter element around -30 of transcription start
CsDof76	TATA-box	1758	1762	core promoter element around -30 of transcription start
CsDof76	TATA-box	1779	1785	core promoter element around -30 of transcription start
CsDof76	TATA-box	1780	1786	core promoter element around -30 of transcription start
CsDof76	TATA-box	1781	1787	core promoter element around -30 of transcription start
CsDof76	TATA-box	1782	1788	core promoter element around -30 of transcription start
CsDof76	TATA-box	1783	1789	core promoter element around -30 of transcription start
CsDof76	TATA-box	1784	1790	core promoter element around -30 of transcription start
CsDof76	TATA-box	1785	1791	core promoter element around -30 of transcription start
CsDof76	TATA-box	1787	1791	core promoter element around -30 of transcription start
CsDof76	TATA-box	1803	1809	core promoter element around -30 of transcription start
CsDof76	TATA-box	1805	1811	core promoter element around -30 of transcription start
CsDof76	TATA-box	1806	1812	core promoter element around -30 of transcription start
CsDof76	TATA-box	1807	1813	core promoter element around -30 of transcription start
CsDof76	TATA-box	1808	1814	core promoter element around -30 of transcription start
CsDof76	TATA-box	1809	1813	core promoter element around -30 of transcription start
CsDof76	TATA-box	1890	1896	core promoter element around -30 of transcription start
CsDof76	TATA-box	1891	1895	core promoter element around -30 of transcription start
CsDof76	GT1-motif	846	853	light responsive element
CsDof76	MBS	159	165	MYB binding site involved in drought-inducibility

Name	Cis-element	Start position	Stop position	Function
CsDof76	MBS	1275	1281	MYB binding site involved in drought-inducibility
CsDof77	ABRE	701	707	abscisic acid responsiveness
CsDof77	ABRE	702	707	abscisic acid responsiveness
CsDof77	TGA-element	1757	1763	auxin-responsive element
CsDof77	TATC-box	761	768	cis-acting element involved in gibberellin-responsiveness
CsDof77	ARE	173	179	cis-acting regulatory element essential for the anaerobic induction
CsDof77	ARE	191	197	cis-acting regulatory element essential for the anaerobic induction
CsDof77	ARE	554	560	cis-acting regulatory element essential for the anaerobic induction
CsDof77	ARE	1025	1031	cis-acting regulatory element essential for the anaerobic induction
CsDof77	ARE	1069	1075	cis-acting regulatory element essential for the anaerobic induction
CsDof77	ARE	1487	1493	cis-acting regulatory element essential for the anaerobic induction
CsDof77	circadian	651	660	cis-acting regulatory element involved in circadian control
CsDof77	circadian	1602	1611	cis-acting regulatory element involved in circadian control
CsDof77	G-Box	701	707	cis-acting regulatory element involved in light responsiveness
CsDof77	G-box	700	708	cis-acting regulatory element involved in light responsiveness
CsDof77	G-box	701	707	cis-acting regulatory element involved in light responsiveness
CsDof77	CAAT-box	710	715	common cis-acting element in promoter and enhancer regions
CsDof77	CAAT-box	1157	1162	common cis-acting element in promoter and enhancer regions
CsDof77	CAAT-box	1433	1438	common cis-acting element in promoter and enhancer regions
CsDof77	CAAT-box	1716	1721	common cis-acting element in promoter and enhancer regions
CsDof77	CAAT-box	1873	1878	common cis-acting element in promoter and enhancer regions
CsDof77	CAAT-box	1895	1900	common cis-acting element in promoter and enhancer regions
CsDof77	TATA-box	5	10	core promoter element around -30 of transcription start
CsDof77	TATA-box	6	10	core promoter element around -30 of transcription start
CsDof77	TATA-box	23	29	core promoter element around -30 of transcription start
CsDof77	TATA-box	24	30	core promoter element around -30 of transcription start
CsDof77	TATA-box	26	30	core promoter element around -30 of transcription start
CsDof77	TATA-box	139	147	core promoter element around -30 of transcription start
CsDof77	TATA-box	143	152	core promoter element around -30 of transcription start
CsDof77	TATA-box	144	151	core promoter element around -30 of transcription start
CsDof77	TATA-box	145	151	core promoter element around -30 of transcription start
CsDof77	TATA-box	146	151	core promoter element around -30 of transcription start
CsDof77	TATA-box	147	151	core promoter element around -30 of transcription start
CsDof77	TATA-box	201	207	core promoter element around -30 of transcription start
CsDof77	TATA-box	202	206	core promoter element around -30 of transcription start
CsDof77	TATA-box	359	365	core promoter element around -30 of transcription start
CsDof77	TATA-box	361	365	core promoter element around -30 of transcription start
CsDof77	TATA-box	377	383	core promoter element around -30 of transcription start
CsDof77	TATA-box	379	383	core promoter element around -30 of transcription start
CsDof77	TATA-box	884	890	core promoter element around -30 of transcription start
CsDof77	TATA-box	885	889	core promoter element around -30 of transcription start
CsDof77	TATA-box	942	947	core promoter element around -30 of transcription start
CsDof77	TATA-box	943	947	core promoter element around -30 of transcription start
CsDof77	TATA-box	948	954	core promoter element around -30 of transcription start
CsDof77	TATA-box	949	956	core promoter element around -30 of transcription start
CsDof77	TATA-box	950	956	core promoter element around -30 of transcription start
CsDof77	TATA-box	951	957	core promoter element around -30 of transcription start
CsDof77	TATA-box	952	956	core promoter element around -30 of transcription start
CsDof77	TATA-box	1131	1137	core promoter element around -30 of transcription start
CsDof77	TATA-box	1133	1137	core promoter element around -30 of transcription start
CsDof77	TATA-box	1140	1146	core promoter element around -30 of transcription start

Name	Cis-element	Start position	Stop position	Function
CsDof77	TATA-box	1142	1146	core promoter element around -30 of transcription start
CsDof77	TATA-box	1227	1233	core promoter element around -30 of transcription start
CsDof77	TATA-box	1228	1232	core promoter element around -30 of transcription start
CsDof77	TATA-box	1625	1633	core promoter element around -30 of transcription start
CsDof77	TATA-box	1771	1775	core promoter element around -30 of transcription start
CsDof77	TATA-box	1790	1796	core promoter element around -30 of transcription start
CsDof77	TATA-box	1792	1798	core promoter element around -30 of transcription start
CsDof77	TATA-box	1793	1799	core promoter element around -30 of transcription start
CsDof77	TATA-box	1794	1800	core promoter element around -30 of transcription start
CsDof77	TATA-box	1795	1801	core promoter element around -30 of transcription start
CsDof77	TATA-box	1796	1802	core promoter element around -30 of transcription start
CsDof77	TATA-box	1797	1803	core promoter element around -30 of transcription start
CsDof77	TATA-box	1798	1804	core promoter element around -30 of transcription start
CsDof77	TATA-box	1799	1805	core promoter element around -30 of transcription start
CsDof77	TATA-box	1800	1806	core promoter element around -30 of transcription start
CsDof77	TATA-box	1801	1807	core promoter element around -30 of transcription start
CsDof77	TATA-box	1802	1808	core promoter element around -30 of transcription start
CsDof77	TATA-box	1803	1809	core promoter element around -30 of transcription start
CsDof77	TATA-box	1804	1808	core promoter element around -30 of transcription start
CsDof77	TATA-box	1973	1979	core promoter element around -30 of transcription start
CsDof77	TATA-box	1974	1979	core promoter element around -30 of transcription start
CsDof77	TATA-box	1975	1979	core promoter element around -30 of transcription start
CsDof77	AT-rich sequence	1861	1870	element for maximal elicitor-mediated activation (2copies)
CsDof77	MBSI	1039	1049.5	MYB binding site involved in flavonoid biosynthetic genes regulation
CsDof77	MRE	1500	1507	MYB binding site involved in light responsiveness
CsDof77	CCAAT-box	878	884	MYBHv1 binding site
CsDof78	ABRE	175	184	abscisic acid responsiveness
CsDof78	ABRE	177	182	abscisic acid responsiveness
CsDof78	ABRE	182	187	abscisic acid responsiveness
CsDof78	ABRE	448	453	abscisic acid responsiveness
CsDof78	ABRE	746	754	abscisic acid responsiveness
CsDof78	ABRE	748	753	abscisic acid responsiveness
CsDof78	ACE	746	755	cis-acting element involved in light responsiveness
CsDof78	LTR	798	804	cis-acting element involved in low-temperature responsiveness
CsDof78	LTR	1710	1716	cis-acting element involved in low-temperature responsiveness
CsDof78	TCA-element	136	146	cis-acting element involved in salicylic acid responsiveness
CsDof78	SARE	1051	1062	cis-acting element involved in salicylic acid responsiveness
CsDof78	ARE	377	383	cis-acting regulatory element essential for the anaerobic induction
CsDof78	ARE	421	427	cis-acting regulatory element essential for the anaerobic induction
CsDof78	G-box	177	183	cis-acting regulatory element involved in light responsiveness
CsDof78	G-box	181	187	cis-acting regulatory element involved in light responsiveness
CsDof78	G-box	448	454	cis-acting regulatory element involved in light responsiveness
CsDof78	G-box	748	754	cis-acting regulatory element involved in light responsiveness
CsDof78	O2-site	29	37	cis-acting regulatory element involved in zein metabolism regulation
CsDof78	CAAT-box	115	120	common cis-acting element in promoter and enhancer regions
CsDof78	CAAT-box	370	375	common cis-acting element in promoter and enhancer regions
CsDof78	CAAT-box	731	736	common cis-acting element in promoter and enhancer regions
CsDof78	CAAT-box	805	810	common cis-acting element in promoter and enhancer regions
CsDof78	CAAT-box	867	872	common cis-acting element in promoter and enhancer regions
CsDof78	CAAT-box	999	1004	common cis-acting element in promoter and enhancer regions
CsDof78	CAAT-box	1107	1112	common cis-acting element in promoter and enhancer regions

Name	Cis-element	Start position	Stop position	Function
CsDof78	CAAT-box	1631	1636	common cis-acting element in promoter and enhancer regions
CsDof78	CAAT-box	1776	1781	common cis-acting element in promoter and enhancer regions
CsDof78	CAAT-box	1909	1914	common cis-acting element in promoter and enhancer regions
CsDof78	TATA-box	71	77	core promoter element around -30 of transcription start
CsDof78	TATA-box	72	77	core promoter element around -30 of transcription start
CsDof78	TATA-box	73	77	core promoter element around -30 of transcription start
CsDof78	TATA-box	103	110	core promoter element around -30 of transcription start
CsDof78	TATA-box	126	130	core promoter element around -30 of transcription start
CsDof78	TATA-box	167	171	core promoter element around -30 of transcription start
CsDof78	TATA-box	270	275	core promoter element around -30 of transcription start
CsDof78	TATA-box	271	275	core promoter element around -30 of transcription start
CsDof78	TATA-box	288	296	core promoter element around -30 of transcription start
CsDof78	TATA-box	356	360	core promoter element around -30 of transcription start
CsDof78	TATA-box	380	388	core promoter element around -30 of transcription start
CsDof78	TATA-box	489	497	core promoter element around -30 of transcription start
CsDof78	TATA-box	497	504	core promoter element around -30 of transcription start
CsDof78	TATA-box	498	504	core promoter element around -30 of transcription start
CsDof78	TATA-box	499	504	core promoter element around -30 of transcription start
CsDof78	TATA-box	500	504	core promoter element around -30 of transcription start
CsDof78	TATA-box	530	535	core promoter element around -30 of transcription start
CsDof78	TATA-box	531	535	core promoter element around -30 of transcription start
CsDof78	TATA-box	544	548	core promoter element around -30 of transcription start
CsDof78	TATA-box	549	556	core promoter element around -30 of transcription start
CsDof78	TATA-box	555	561	core promoter element around -30 of transcription start
CsDof78	TATA-box	556	562	core promoter element around -30 of transcription start
CsDof78	TATA-box	557	563	core promoter element around -30 of transcription start
CsDof78	TATA-box	558	562	core promoter element around -30 of transcription start
CsDof78	TATA-box	590	595	core promoter element around -30 of transcription start
CsDof78	TATA-box	591	595	core promoter element around -30 of transcription start
CsDof78	TATA-box	603	607	core promoter element around -30 of transcription start
CsDof78	TATA-box	627	633	core promoter element around -30 of transcription start
CsDof78	TATA-box	628	634	core promoter element around -30 of transcription start
CsDof78	TATA-box	629	635	core promoter element around -30 of transcription start
CsDof78	TATA-box	630	636	core promoter element around -30 of transcription start
CsDof78	TATA-box	631	635	core promoter element around -30 of transcription start
CsDof78	TATA-box	664	669	core promoter element around -30 of transcription start
CsDof78	TATA-box	665	669	core promoter element around -30 of transcription start
CsDof78	TATA-box	740	744	core promoter element around -30 of transcription start
CsDof78	TATA-box	752	756	core promoter element around -30 of transcription start
CsDof78	TATA-box	766	772	core promoter element around -30 of transcription start
CsDof78	TATA-box	767	772	core promoter element around -30 of transcription start
CsDof78	TATA-box	768	772	core promoter element around -30 of transcription start
CsDof78	TATA-box	775	781	core promoter element around -30 of transcription start
CsDof78	TATA-box	776	782	core promoter element around -30 of transcription start
CsDof78	TATA-box	778	782	core promoter element around -30 of transcription start
CsDof78	TATA-box	1163	1170	core promoter element around -30 of transcription start
CsDof78	TATA-box	1414	1421	core promoter element around -30 of transcription start
CsDof78	TATA-box	1415	1421	core promoter element around -30 of transcription start
CsDof78	TATA-box	1416	1421	core promoter element around -30 of transcription start
CsDof78	TATA-box	1417	1421	core promoter element around -30 of transcription start
CsDof78	TATA-box	1445	1450	core promoter element around -30 of transcription start

Name	Cis-element	Start position	Stop position	Function
CsDof78	TATA-box	1446	1450	core promoter element around -30 of transcription start
CsDof78	TATA-box	1451	1458	core promoter element around -30 of transcription start
CsDof78	TATA-box	1457	1463	core promoter element around -30 of transcription start
CsDof78	TATA-box	1458	1464	core promoter element around -30 of transcription start
CsDof78	TATA-box	1459	1465	core promoter element around -30 of transcription start
CsDof78	TATA-box	1460	1464	core promoter element around -30 of transcription start
CsDof78	TATA-box	1490	1495	core promoter element around -30 of transcription start
CsDof78	TATA-box	1491	1495	core promoter element around -30 of transcription start
CsDof78	TATA-box	1503	1507	core promoter element around -30 of transcription start
CsDof78	TATA-box	1527	1533	core promoter element around -30 of transcription start
CsDof78	TATA-box	1528	1534	core promoter element around -30 of transcription start
CsDof78	TATA-box	1529	1535	core promoter element around -30 of transcription start
CsDof78	TATA-box	1530	1536	core promoter element around -30 of transcription start
CsDof78	TATA-box	1531	1535	core promoter element around -30 of transcription start
CsDof78	TATA-box	1564	1569	core promoter element around -30 of transcription start
CsDof78	TATA-box	1565	1569	core promoter element around -30 of transcription start
CsDof78	TATA-box	1640	1644	core promoter element around -30 of transcription start
CsDof78	TATA-box	1652	1656	core promoter element around -30 of transcription start
CsDof78	TATA-box	1678	1684	core promoter element around -30 of transcription start
CsDof78	TATA-box	1679	1684	core promoter element around -30 of transcription start
CsDof78	TATA-box	1680	1684	core promoter element around -30 of transcription start
CsDof78	TATA-box	1687	1693	core promoter element around -30 of transcription start
CsDof78	TATA-box	1688	1694	core promoter element around -30 of transcription start
CsDof78	TATA-box	1690	1694	core promoter element around -30 of transcription start
CsDof78	3-AF1 binding site	651	661	light responsive element
CsDof78	3-AF1 binding site	948	958	light responsive element
CsDof78	3-AF1 binding site	1171	1181	light responsive element
CsDof78	3-AF1 binding site	1551	1561	light responsive element
CsDof78	3-AF1 binding site	1857	1867	light responsive element
CsDof79	ABRE	468	473	abscisic acid responsiveness
CsDof79	ABRE	744	750	abscisic acid responsiveness
CsDof79	ABRE	745	750	abscisic acid responsiveness
CsDof79	ABRE	1548	1557	abscisic acid responsiveness
CsDof79	ABRE	1807	1812	abscisic acid responsiveness
CsDof79	TCA-element	1307	1317	cis-acting element involved in salicylic acid responsiveness
CsDof79	TCA-element	1532	1541	cis-acting element involved in salicylic acid responsiveness
CsDof79	ARE	1453	1459	cis-acting regulatory element essential for the anaerobic induction
CsDof79	G-box	28	37	cis-acting regulatory element involved in light responsiveness
CsDof79	G-box	468	474	cis-acting regulatory element involved in light responsiveness
CsDof79	G-box	744	750	cis-acting regulatory element involved in light responsiveness
CsDof79	G-box	1512	1518	cis-acting regulatory element involved in light responsiveness
CsDof79	G-box	1807	1815	cis-acting regulatory element involved in light responsiveness
CsDof79	G-Box	744	750	cis-acting regulatory element involved in light responsiveness
CsDof79	G-Box	1807	1813	cis-acting regulatory element involved in light responsiveness
CsDof79	CAT-box	1937	1943	cis-acting regulatory element related to meristem expression
CsDof79	CAT-box	1941	1947	cis-acting regulatory element related to meristem expression
CsDof79	CAAT-box	75	80	common cis-acting element in promoter and enhancer regions
CsDof79	CAAT-box	99	104	common cis-acting element in promoter and enhancer regions
CsDof79	CAAT-box	189	194	common cis-acting element in promoter and enhancer regions
CsDof79	CAAT-box	296	301	common cis-acting element in promoter and enhancer regions
CsDof79	CAAT-box	337	342	common cis-acting element in promoter and enhancer regions

Name	Cis-element	Start position	Stop position	Function
CsDof79	CAAT-box	506	511	common cis-acting element in promoter and enhancer regions
CsDof79	CAAT-box	851	856	common cis-acting element in promoter and enhancer regions
CsDof79	CAAT-box	869	874	common cis-acting element in promoter and enhancer regions
CsDof79	CAAT-box	1409	1414	common cis-acting element in promoter and enhancer regions
CsDof79	CAAT-box	1544	1549	common cis-acting element in promoter and enhancer regions
CsDof79	CAAT-box	1577	1582	common cis-acting element in promoter and enhancer regions
CsDof79	CAAT-box	1653	1658	common cis-acting element in promoter and enhancer regions
CsDof79	CAAT-box	1687	1692	common cis-acting element in promoter and enhancer regions
CsDof79	CAAT-box	1744	1749	common cis-acting element in promoter and enhancer regions
CsDof79	CAAT-box	1958	1963	common cis-acting element in promoter and enhancer regions
CsDof79	TATA-box	53	59	core promoter element around -30 of transcription start
CsDof79	TATA-box	54	59	core promoter element around -30 of transcription start
CsDof79	TATA-box	55	59	core promoter element around -30 of transcription start
CsDof79	TATA-box	78	84	core promoter element around -30 of transcription start
CsDof79	TATA-box	79	86	core promoter element around -30 of transcription start
CsDof79	TATA-box	80	86	core promoter element around -30 of transcription start
CsDof79	TATA-box	81	87	core promoter element around -30 of transcription start
CsDof79	TATA-box	82	86	core promoter element around -30 of transcription start
CsDof79	TATA-box	92	98	core promoter element around -30 of transcription start
CsDof79	TATA-box	93	98	core promoter element around -30 of transcription start
CsDof79	TATA-box	94	98	core promoter element around -30 of transcription start
CsDof79	TATA-box	119	125	core promoter element around -30 of transcription start
CsDof79	TATA-box	120	126	core promoter element around -30 of transcription start
CsDof79	TATA-box	121	125	core promoter element around -30 of transcription start
CsDof79	TATA-box	237	244	core promoter element around -30 of transcription start
CsDof79	TATA-box	410	417	core promoter element around -30 of transcription start
CsDof79	TATA-box	446	452	core promoter element around -30 of transcription start
CsDof79	TATA-box	447	451	core promoter element around -30 of transcription start
CsDof79	TATA-box	529	535	core promoter element around -30 of transcription start
CsDof79	TATA-box	530	534	core promoter element around -30 of transcription start
CsDof79	TATA-box	579	583	core promoter element around -30 of transcription start
CsDof79	TATA-box	608	613	core promoter element around -30 of transcription start
CsDof79	TATA-box	609	613	core promoter element around -30 of transcription start
CsDof79	TATA-box	802	806	core promoter element around -30 of transcription start
CsDof79	TATA-box	822	828	core promoter element around -30 of transcription start
CsDof79	TATA-box	823	827	core promoter element around -30 of transcription start
CsDof79	TATA-box	962	966	core promoter element around -30 of transcription start
CsDof79	TATA-box	988	994	core promoter element around -30 of transcription start
CsDof79	TATA-box	989	994	core promoter element around -30 of transcription start
CsDof79	TATA-box	990	994	core promoter element around -30 of transcription start
CsDof79	TATA-box	1009	1013	core promoter element around -30 of transcription start
CsDof79	TATA-box	1029	1035	core promoter element around -30 of transcription start
CsDof79	TATA-box	1030	1036	core promoter element around -30 of transcription start
CsDof79	TATA-box	1031	1037	core promoter element around -30 of transcription start
CsDof79	TATA-box	1032	1036	core promoter element around -30 of transcription start
CsDof79	TATA-box	1035	1041	core promoter element around -30 of transcription start
CsDof79	TATA-box	1036	1043	core promoter element around -30 of transcription start
CsDof79	TATA-box	1037	1043	core promoter element around -30 of transcription start
CsDof79	TATA-box	1039	1043	core promoter element around -30 of transcription start
CsDof79	TATA-box	1045	1051	core promoter element around -30 of transcription start
CsDof79	TATA-box	1046	1052	core promoter element around -30 of transcription start

Name	Cis-element	Start position	Stop position	Function
CsDof79	TATA-box	1047	1051	core promoter element around -30 of transcription start
CsDof79	TATA-box	1059	1065	core promoter element around -30 of transcription start
CsDof79	TATA-box	1060	1066	core promoter element around -30 of transcription start
CsDof79	TATA-box	1061	1067	core promoter element around -30 of transcription start
CsDof79	TATA-box	1062	1068	core promoter element around -30 of transcription start
CsDof79	TATA-box	1063	1069	core promoter element around -30 of transcription start
CsDof79	TATA-box	1065	1069	core promoter element around -30 of transcription start
CsDof79	TATA-box	1278	1284	core promoter element around -30 of transcription start
CsDof79	TATA-box	1279	1283	core promoter element around -30 of transcription start
CsDof79	TATA-box	1320	1326	core promoter element around -30 of transcription start
CsDof79	TATA-box	1321	1326	core promoter element around -30 of transcription start
CsDof79	TATA-box	1322	1326	core promoter element around -30 of transcription start
CsDof79	TATA-box	1487	1495	core promoter element around -30 of transcription start
CsDof79	TATA-box	1558	1564	core promoter element around -30 of transcription start
CsDof79	TATA-box	1559	1564	core promoter element around -30 of transcription start
CsDof79	TATA-box	1560	1564	core promoter element around -30 of transcription start
CsDof79	TATA-box	1714	1720	core promoter element around -30 of transcription start
CsDof79	TATA-box	1715	1722	core promoter element around -30 of transcription start
CsDof79	TATA-box	1716	1722	core promoter element around -30 of transcription start
CsDof79	TATA-box	1717	1723	core promoter element around -30 of transcription start
CsDof79	TATA-box	1718	1722	core promoter element around -30 of transcription start
CsDof79	TATA-box	1899	1905	core promoter element around -30 of transcription start
CsDof79	TATA-box	1900	1906	core promoter element around -30 of transcription start
CsDof79	TATA-box	1901	1907	core promoter element around -30 of transcription start
CsDof79	TATA-box	1902	1906	core promoter element around -30 of transcription start
CsDof79	TATA-box	1927	1931	core promoter element around -30 of transcription start
CsDof79	TATA-box	1991	1995	core promoter element around -30 of transcription start
CsDof79	AT-rich sequence	532	541	element for maximal elicitor-mediated activation (2copies)
CsDof79	AT-rich sequence	1325	1334	element for maximal elicitor-mediated activation (2copies)
CsDof79	Sp1	1802	1808	light responsive element
CsDof79	GT1-motif	502	508	light responsive element
CsDof80	ABRE	1684	1689	abscisic acid responsiveness
CsDof80	ABRE	1829	1834	abscisic acid responsiveness
CsDof80	LTR	969	975	cis-acting element involved in low-temperature responsiveness
CsDof80	TCA-element	604	613	cis-acting element involved in salicylic acid responsiveness
CsDof80	TCA-element	1643	1652	cis-acting element involved in salicylic acid responsiveness
CsDof80	TCA-element	1899	1908	cis-acting element involved in salicylic acid responsiveness
CsDof80	SARE	607	618	cis-acting element involved in salicylic acid responsiveness
CsDof80	ARE	327	333	cis-acting regulatory element essential for the anaerobic induction
CsDof80	ARE	418	424	cis-acting regulatory element essential for the anaerobic induction
CsDof80	ARE	574	580	cis-acting regulatory element essential for the anaerobic induction
CsDof80	ARE	836	842	cis-acting regulatory element essential for the anaerobic induction
CsDof80	AuxRR-core	10	17	cis-acting regulatory element involved in auxin responsiveness
CsDof80	G-box	1683	1689	cis-acting regulatory element involved in light responsiveness
CsDof80	G-box	1828	1834	cis-acting regulatory element involved in light responsiveness
CsDof80	TGACG-motif	1682	1687	cis-acting regulatory element involved in the MeJA-responsiveness
CsDof80	CGTCA-motif	1682	1687	cis-acting regulatory element involved in the MeJA-responsiveness
CsDof80	CAT-box	121	127	cis-acting regulatory element related to meristem expression
CsDof80	CAT-box	1718	1724	cis-acting regulatory element related to meristem expression
CsDof80	GCN4_motif	1050	1057	cis-regulatory element involved in endosperm expression
CsDof80	CAAT-box	33	38	common cis-acting element in promoter and enhancer regions

Name	Cis-element	Start position	Stop position	Function
CsDof80	CAAT-box	265	270	common cis-acting element in promoter and enhancer regions
CsDof80	CAAT-box	268	273	common cis-acting element in promoter and enhancer regions
CsDof80	CAAT-box	390	395	common cis-acting element in promoter and enhancer regions
CsDof80	CAAT-box	395	400	common cis-acting element in promoter and enhancer regions
CsDof80	CAAT-box	473	478	common cis-acting element in promoter and enhancer regions
CsDof80	CAAT-box	615	620	common cis-acting element in promoter and enhancer regions
CsDof80	CAAT-box	959	964	common cis-acting element in promoter and enhancer regions
CsDof80	CAAT-box	1011	1016	common cis-acting element in promoter and enhancer regions
CsDof80	CAAT-box	1027	1032	common cis-acting element in promoter and enhancer regions
CsDof80	CAAT-box	1190	1195	common cis-acting element in promoter and enhancer regions
CsDof80	CAAT-box	1229	1234	common cis-acting element in promoter and enhancer regions
CsDof80	CAAT-box	1707	1712	common cis-acting element in promoter and enhancer regions
CsDof80	CAAT-box	1931	1936	common cis-acting element in promoter and enhancer regions
CsDof80	CAAT-box	1958	1963	common cis-acting element in promoter and enhancer regions
CsDof80	TATA-box	28	32	core promoter element around -30 of transcription start
CsDof80	TATA-box	139	143	core promoter element around -30 of transcription start
CsDof80	TATA-box	450	459	core promoter element around -30 of transcription start
CsDof80	TATA-box	451	458	core promoter element around -30 of transcription start
CsDof80	TATA-box	452	458	core promoter element around -30 of transcription start
CsDof80	TATA-box	453	458	core promoter element around -30 of transcription start
CsDof80	TATA-box	454	458	core promoter element around -30 of transcription start
CsDof80	TATA-box	539	544	core promoter element around -30 of transcription start
CsDof80	TATA-box	540	544	core promoter element around -30 of transcription start
CsDof80	TATA-box	602	608	core promoter element around -30 of transcription start
CsDof80	TATA-box	603	607	core promoter element around -30 of transcription start
CsDof80	TATA-box	644	648	core promoter element around -30 of transcription start
CsDof80	TATA-box	720	727	core promoter element around -30 of transcription start
CsDof80	TATA-box	721	727	core promoter element around -30 of transcription start
CsDof80	TATA-box	722	727	core promoter element around -30 of transcription start
CsDof80	TATA-box	723	727	core promoter element around -30 of transcription start
CsDof80	TATA-box	725	733	core promoter element around -30 of transcription start
CsDof80	TATA-box	726	733	core promoter element around -30 of transcription start
CsDof80	TATA-box	727	733	core promoter element around -30 of transcription start
CsDof80	TATA-box	728	733	core promoter element around -30 of transcription start
CsDof80	TATA-box	729	733	core promoter element around -30 of transcription start
CsDof80	TATA-box	745	753	core promoter element around -30 of transcription start
CsDof80	TATA-box	816	825	core promoter element around -30 of transcription start
CsDof80	TATA-box	817	824	core promoter element around -30 of transcription start
CsDof80	TATA-box	818	824	core promoter element around -30 of transcription start
CsDof80	TATA-box	819	826	core promoter element around -30 of transcription start
CsDof80	TATA-box	820	826	core promoter element around -30 of transcription start
CsDof80	TATA-box	821	827	core promoter element around -30 of transcription start
CsDof80	TATA-box	822	826	core promoter element around -30 of transcription start
CsDof80	TATA-box	1017	1021	core promoter element around -30 of transcription start
CsDof80	TATA-box	1061	1065	core promoter element around -30 of transcription start
CsDof80	TATA-box	1066	1070	core promoter element around -30 of transcription start
CsDof80	TATA-box	1073	1078	core promoter element around -30 of transcription start
CsDof80	TATA-box	1074	1078	core promoter element around -30 of transcription start
CsDof80	TATA-box	1096	1100	core promoter element around -30 of transcription start
CsDof80	TATA-box	1139	1144	core promoter element around -30 of transcription start
CsDof80	TATA-box	1140	1144	core promoter element around -30 of transcription start

Name	Cis-element	Start position	Stop position	Function
CsDof80	TATA-box	1169	1176	core promoter element around -30 of transcription start
CsDof80	TATA-box	1170	1176	core promoter element around -30 of transcription start
CsDof80	TATA-box	1171	1176	core promoter element around -30 of transcription start
CsDof80	TATA-box	1172	1176	core promoter element around -30 of transcription start
CsDof80	TATA-box	1232	1238	core promoter element around -30 of transcription start
CsDof80	TATA-box	1234	1238	core promoter element around -30 of transcription start
CsDof80	TATA-box	1277	1283	core promoter element around -30 of transcription start
CsDof80	TATA-box	1278	1282	core promoter element around -30 of transcription start
CsDof80	TATA-box	1291	1300	core promoter element around -30 of transcription start
CsDof80	TATA-box	1294	1301	core promoter element around -30 of transcription start
CsDof80	TATA-box	1295	1301	core promoter element around -30 of transcription start
CsDof80	TATA-box	1296	1303	core promoter element around -30 of transcription start
CsDof80	TATA-box	1297	1303	core promoter element around -30 of transcription start
CsDof80	TATA-box	1299	1303	core promoter element around -30 of transcription start
CsDof80	TATA-box	1337	1343	core promoter element around -30 of transcription start
CsDof80	TATA-box	1339	1345	core promoter element around -30 of transcription start
CsDof80	TATA-box	1340	1346	core promoter element around -30 of transcription start
CsDof80	TATA-box	1341	1347	core promoter element around -30 of transcription start
CsDof80	TATA-box	1342	1348	core promoter element around -30 of transcription start
CsDof80	TATA-box	1343	1349	core promoter element around -30 of transcription start
CsDof80	TATA-box	1344	1350	core promoter element around -30 of transcription start
CsDof80	TATA-box	1345	1351	core promoter element around -30 of transcription start
CsDof80	TATA-box	1347	1351	core promoter element around -30 of transcription start
CsDof80	TATA-box	1352	1358	core promoter element around -30 of transcription start
CsDof80	TATA-box	1353	1357	core promoter element around -30 of transcription start
CsDof80	TATA-box	1366	1373	core promoter element around -30 of transcription start
CsDof80	TATA-box	1367	1373	core promoter element around -30 of transcription start
CsDof80	TATA-box	1368	1375	core promoter element around -30 of transcription start
CsDof80	TATA-box	1369	1375	core promoter element around -30 of transcription start
CsDof80	TATA-box	1370	1376	core promoter element around -30 of transcription start
CsDof80	TATA-box	1371	1375	core promoter element around -30 of transcription start
CsDof80	TATA-box	1382	1389	core promoter element around -30 of transcription start
CsDof80	TATA-box	1383	1389	core promoter element around -30 of transcription start
CsDof80	TATA-box	1384	1389	core promoter element around -30 of transcription start
CsDof80	TATA-box	1385	1389	core promoter element around -30 of transcription start
CsDof80	TATA-box	1402	1408	core promoter element around -30 of transcription start
CsDof80	TATA-box	1403	1409	core promoter element around -30 of transcription start
CsDof80	TATA-box	1404	1408	core promoter element around -30 of transcription start
CsDof80	TATA-box	1406	1414	core promoter element around -30 of transcription start
CsDof80	TATA-box	1407	1414	core promoter element around -30 of transcription start
CsDof80	TATA-box	1408	1414	core promoter element around -30 of transcription start
CsDof80	TATA-box	1409	1414	core promoter element around -30 of transcription start
CsDof80	TATA-box	1410	1414	core promoter element around -30 of transcription start
CsDof80	TATA-box	1442	1448	core promoter element around -30 of transcription start
CsDof80	TATA-box	1443	1448	core promoter element around -30 of transcription start
CsDof80	TATA-box	1444	1448	core promoter element around -30 of transcription start
CsDof80	TATA-box	1497	1504	core promoter element around -30 of transcription start
CsDof80	TATA-box	1498	1504	core promoter element around -30 of transcription start
CsDof80	TATA-box	1499	1504	core promoter element around -30 of transcription start
CsDof80	TATA-box	1500	1504	core promoter element around -30 of transcription start
CsDof80	TATA-box	1529	1536	core promoter element around -30 of transcription start

Name	Cis-element	Start position	Stop position	Function
CsDof80	TATA-box	1530	1536	core promoter element around -30 of transcription start
CsDof80	TATA-box	1531	1536	core promoter element around -30 of transcription start
CsDof80	TATA-box	1532	1536	core promoter element around -30 of transcription start
CsDof80	TATA-box	1547	1551	core promoter element around -30 of transcription start
CsDof80	TATA-box	1573	1577	core promoter element around -30 of transcription start
CsDof80	TATA-box	1609	1616	core promoter element around -30 of transcription start
CsDof80	TATA-box	1903	1911	core promoter element around -30 of transcription start
CsDof80	TATA-box	1906	1911	core promoter element around -30 of transcription start
CsDof80	TATA-box	1907	1911	core promoter element around -30 of transcription start
CsDof80	P-box	63	70	gibberellin-responsive element
CsDof80	GARE-motif	113	120	gibberellin-responsive element
CsDof80	GT1-motif	1211	1217	light responsive element
CsDof80	MBS	195	201	MYB binding site involved in drought-inducibility
CsDof80	MRE	851	858	MYB binding site involved in light responsiveness
CsDof81	ABRE	153	162	abscisic acid responsiveness
CsDof81	ABRE	156	161	abscisic acid responsiveness
CsDof81	ABRE	1187	1192	abscisic acid responsiveness
CsDof81	ACE	1776	1785	cis-acting element involved in light responsiveness
CsDof81	TCA-element	659	668	cis-acting element involved in salicylic acid responsiveness
CsDof81	TCA-element	1891	1900	cis-acting element involved in salicylic acid responsiveness
CsDof81	ARE	1237	1243	cis-acting regulatory element essential for the anaerobic induction
CsDof81	ARE	1544	1550	cis-acting regulatory element essential for the anaerobic induction
CsDof81	G-box	155	161	cis-acting regulatory element involved in light responsiveness
CsDof81	G-Box	1187	1193	cis-acting regulatory element involved in light responsiveness
CsDof81	O2-site	633	642	cis-acting regulatory element involved in zein metabolism regulation
CsDof81	O2-site	664	673	cis-acting regulatory element involved in zein metabolism regulation
CsDof81	CAAT-box	213	218	common cis-acting element in promoter and enhancer regions
CsDof81	CAAT-box	278	283	common cis-acting element in promoter and enhancer regions
CsDof81	CAAT-box	290	295	common cis-acting element in promoter and enhancer regions
CsDof81	CAAT-box	384	389	common cis-acting element in promoter and enhancer regions
CsDof81	CAAT-box	430	438	common cis-acting element in promoter and enhancer regions
CsDof81	CAAT-box	431	436	common cis-acting element in promoter and enhancer regions
CsDof81	CAAT-box	780	785	common cis-acting element in promoter and enhancer regions
CsDof81	CAAT-box	786	791	common cis-acting element in promoter and enhancer regions
CsDof81	CAAT-box	855	860	common cis-acting element in promoter and enhancer regions
CsDof81	CAAT-box	1008	1013	common cis-acting element in promoter and enhancer regions
CsDof81	CAAT-box	1135	1140	common cis-acting element in promoter and enhancer regions
CsDof81	CAAT-box	1161	1166	common cis-acting element in promoter and enhancer regions
CsDof81	CAAT-box	1182	1187	common cis-acting element in promoter and enhancer regions
CsDof81	CAAT-box	1548	1553	common cis-acting element in promoter and enhancer regions
CsDof81	CAAT-box	1877	1882	common cis-acting element in promoter and enhancer regions
CsDof81	TATA-box	13	17	core promoter element around -30 of transcription start
CsDof81	TATA-box	18	22	core promoter element around -30 of transcription start
CsDof81	TATA-box	172	180	core promoter element around -30 of transcription start
CsDof81	TATA-box	183	191	core promoter element around -30 of transcription start
CsDof81	TATA-box	381	385	core promoter element around -30 of transcription start
CsDof81	TATA-box	391	395	core promoter element around -30 of transcription start
CsDof81	TATA-box	400	406	core promoter element around -30 of transcription start
CsDof81	TATA-box	401	406	core promoter element around -30 of transcription start
CsDof81	TATA-box	402	406	core promoter element around -30 of transcription start
CsDof81	TATA-box	527	533	core promoter element around -30 of transcription start

Name	Cis-element	Start position	Stop position	Function
CsDof81	TATA-box	528	533	core promoter element around -30 of transcription start
CsDof81	TATA-box	529	533	core promoter element around -30 of transcription start
CsDof81	TATA-box	583	587	core promoter element around -30 of transcription start
CsDof81	TATA-box	839	847	core promoter element around -30 of transcription start
CsDof81	TATA-box	842	847	core promoter element around -30 of transcription start
CsDof81	TATA-box	843	847	core promoter element around -30 of transcription start
CsDof81	TATA-box	850	856	core promoter element around -30 of transcription start
CsDof81	TATA-box	851	856	core promoter element around -30 of transcription start
CsDof81	TATA-box	852	856	core promoter element around -30 of transcription start
CsDof81	TATA-box	888	892	core promoter element around -30 of transcription start
CsDof81	TATA-box	1012	1020	core promoter element around -30 of transcription start
CsDof81	TATA-box	1013	1020	core promoter element around -30 of transcription start
CsDof81	TATA-box	1014	1020	core promoter element around -30 of transcription start
CsDof81	TATA-box	1015	1020	core promoter element around -30 of transcription start
CsDof81	TATA-box	1016	1020	core promoter element around -30 of transcription start
CsDof81	TATA-box	1096	1100	core promoter element around -30 of transcription start
CsDof81	TATA-box	1103	1107	core promoter element around -30 of transcription start
CsDof81	TATA-box	1205	1209	core promoter element around -30 of transcription start
CsDof81	TATA-box	1243	1247	core promoter element around -30 of transcription start
CsDof81	TATA-box	1266	1273	core promoter element around -30 of transcription start
CsDof81	TATA-box	1267	1273	core promoter element around -30 of transcription start
CsDof81	TATA-box	1268	1274	core promoter element around -30 of transcription start
CsDof81	TATA-box	1269	1273	core promoter element around -30 of transcription start
CsDof81	TATA-box	1301	1307	core promoter element around -30 of transcription start
CsDof81	TATA-box	1302	1307	core promoter element around -30 of transcription start
CsDof81	TATA-box	1303	1307	core promoter element around -30 of transcription start
CsDof81	TATA-box	1334	1341	core promoter element around -30 of transcription start
CsDof81	TATA-box	1345	1349	core promoter element around -30 of transcription start
CsDof81	TATA-box	1551	1557	core promoter element around -30 of transcription start
CsDof81	TATA-box	1552	1556	core promoter element around -30 of transcription start
CsDof81	TATA-box	1626	1633	core promoter element around -30 of transcription start
CsDof81	TATA-box	1627	1633	core promoter element around -30 of transcription start
CsDof81	TATA-box	1628	1633	core promoter element around -30 of transcription start
CsDof81	TATA-box	1629	1633	core promoter element around -30 of transcription start
CsDof81	TATA-box	1636	1640	core promoter element around -30 of transcription start
CsDof81	TATA-box	1679	1683	core promoter element around -30 of transcription start
CsDof81	TATA-box	1730	1736	core promoter element around -30 of transcription start
CsDof81	TATA-box	1731	1735	core promoter element around -30 of transcription start
CsDof81	TATA-box	1798	1805	core promoter element around -30 of transcription start
CsDof81	TATA-box	1799	1805	core promoter element around -30 of transcription start
CsDof81	TATA-box	1800	1805	core promoter element around -30 of transcription start
CsDof81	TATA-box	1801	1805	core promoter element around -30 of transcription start
CsDof81	TATA-box	1852	1858	core promoter element around -30 of transcription start
CsDof81	TATA-box	1853	1857	core promoter element around -30 of transcription start
CsDof81	TATA-box	1887	1894	core promoter element around -30 of transcription start
CsDof81	TATA-box	1909	1917	core promoter element around -30 of transcription start
CsDof81	TATA-box	1912	1917	core promoter element around -30 of transcription start
CsDof81	TATA-box	1913	1917	core promoter element around -30 of transcription start
CsDof81	AAAC-motif	1119	1130	light responsive element
CsDof81	MRE	1117	1124	MYB binding site involved in light responsiveness
CsDof82	ABRE	1219	1224	abscisic acid responsiveness

Name	Cis-element	Start position	Stop position	Function
CsDof82	TGA-element	1972	1978	auxin-responsive element
CsDof82	ARE	211	217	cis-acting regulatory element essential for the anaerobic induction
CsDof82	ARE	1030	1036	cis-acting regulatory element essential for the anaerobic induction
CsDof82	ARE	1886	1892	cis-acting regulatory element essential for the anaerobic induction
CsDof82	G-box	1215	1221	cis-acting regulatory element involved in light responsiveness
CsDof82	G-box	1218	1224	cis-acting regulatory element involved in light responsiveness
CsDof82	CGTCA-motif	1141	1146	cis-acting regulatory element involved in the MeJA-responsiveness
CsDof82	CGTCA-motif	1368	1373	cis-acting regulatory element involved in the MeJA-responsiveness
CsDof82	CGTCA-motif	1862	1867	cis-acting regulatory element involved in the MeJA-responsiveness
CsDof82	TGACG-motif	1141	1146	cis-acting regulatory element involved in the MeJA-responsiveness
CsDof82	TGACG-motif	1368	1373	cis-acting regulatory element involved in the MeJA-responsiveness
CsDof82	TGACG-motif	1862	1867	cis-acting regulatory element involved in the MeJA-responsiveness
CsDof82	O2-site	114	122	cis-acting regulatory element involved in zein metabolism regulation
CsDof82	CAT-box	1075	1081	cis-acting regulatory element related to meristem expression
CsDof82	CAAT-box	36	41	common cis-acting element in promoter and enhancer regions
CsDof82	CAAT-box	115	120	common cis-acting element in promoter and enhancer regions
CsDof82	CAAT-box	160	165	common cis-acting element in promoter and enhancer regions
CsDof82	CAAT-box	280	285	common cis-acting element in promoter and enhancer regions
CsDof82	CAAT-box	456	461	common cis-acting element in promoter and enhancer regions
CsDof82	CAAT-box	603	608	common cis-acting element in promoter and enhancer regions
CsDof82	CAAT-box	667	672	common cis-acting element in promoter and enhancer regions
CsDof82	CAAT-box	864	869	common cis-acting element in promoter and enhancer regions
CsDof82	CAAT-box	872	877	common cis-acting element in promoter and enhancer regions
CsDof82	CAAT-box	1025	1030	common cis-acting element in promoter and enhancer regions
CsDof82	CAAT-box	1058	1063	common cis-acting element in promoter and enhancer regions
CsDof82	CAAT-box	1165	1170	common cis-acting element in promoter and enhancer regions
CsDof82	CAAT-box	1310	1315	common cis-acting element in promoter and enhancer regions
CsDof82	CAAT-box	1403	1408	common cis-acting element in promoter and enhancer regions
CsDof82	CAAT-box	1418	1423	common cis-acting element in promoter and enhancer regions
CsDof82	CAAT-box	1427	1432	common cis-acting element in promoter and enhancer regions
CsDof82	CAAT-box	1437	1442	common cis-acting element in promoter and enhancer regions
CsDof82	CAAT-box	1501	1506	common cis-acting element in promoter and enhancer regions
CsDof82	CAAT-box	1593	1598	common cis-acting element in promoter and enhancer regions
CsDof82	CAAT-box	1738	1743	common cis-acting element in promoter and enhancer regions
CsDof82	CAAT-box	1780	1785	common cis-acting element in promoter and enhancer regions
CsDof82	TATA-box	97	103	core promoter element around -30 of transcription start
CsDof82	TATA-box	99	103	core promoter element around -30 of transcription start
CsDof82	TATA-box	133	139	core promoter element around -30 of transcription start
CsDof82	TATA-box	135	139	core promoter element around -30 of transcription start
CsDof82	TATA-box	256	260	core promoter element around -30 of transcription start
CsDof82	TATA-box	417	423	core promoter element around -30 of transcription start
CsDof82	TATA-box	418	424	core promoter element around -30 of transcription start
CsDof82	TATA-box	419	423	core promoter element around -30 of transcription start
CsDof82	TATA-box	533	542	core promoter element around -30 of transcription start
CsDof82	TATA-box	534	540	core promoter element around -30 of transcription start
CsDof82	TATA-box	535	539	core promoter element around -30 of transcription start
CsDof82	TATA-box	688	692	core promoter element around -30 of transcription start
CsDof82	TATA-box	723	728	core promoter element around -30 of transcription start
CsDof82	TATA-box	724	728	core promoter element around -30 of transcription start
CsDof82	TATA-box	839	843	core promoter element around -30 of transcription start
CsDof82	TATA-box	850	856	core promoter element around -30 of transcription start

Name	Cis-element	Start position	Stop position	Function
CsDof82	TATA-box	851	855	core promoter element around -30 of transcription start
CsDof82	TATA-box	961	965	core promoter element around -30 of transcription start
CsDof82	TATA-box	1332	1336	core promoter element around -30 of transcription start
CsDof82	TATA-box	1336	1343	core promoter element around -30 of transcription start
CsDof82	TATA-box	1337	1343	core promoter element around -30 of transcription start
CsDof82	TATA-box	1338	1343	core promoter element around -30 of transcription start
CsDof82	TATA-box	1339	1343	core promoter element around -30 of transcription start
CsDof82	TATA-box	1362	1368	core promoter element around -30 of transcription start
CsDof82	TATA-box	1363	1368	core promoter element around -30 of transcription start
CsDof82	TATA-box	1364	1368	core promoter element around -30 of transcription start
CsDof82	TATA-box	1397	1403	core promoter element around -30 of transcription start
CsDof82	TATA-box	1398	1403	core promoter element around -30 of transcription start
CsDof82	TATA-box	1399	1403	core promoter element around -30 of transcription start
CsDof82	TATA-box	1464	1470	core promoter element around -30 of transcription start
CsDof82	TATA-box	1465	1470	core promoter element around -30 of transcription start
CsDof82	TATA-box	1466	1470	core promoter element around -30 of transcription start
CsDof82	TATA-box	1544	1552	core promoter element around -30 of transcription start
CsDof82	TATA-box	1550	1554	core promoter element around -30 of transcription start
CsDof82	TATA-box	1600	1604	core promoter element around -30 of transcription start
CsDof82	TATA-box	1619	1624	core promoter element around -30 of transcription start
CsDof82	TATA-box	1620	1624	core promoter element around -30 of transcription start
CsDof82	TATA-box	1658	1662	core promoter element around -30 of transcription start
CsDof82	TATA-box	1741	1747	core promoter element around -30 of transcription start
CsDof82	TATA-box	1742	1747	core promoter element around -30 of transcription start
CsDof82	TATA-box	1743	1747	core promoter element around -30 of transcription start
CsDof82	TATA-box	1821	1830	core promoter element around -30 of transcription start
CsDof82	TATA-box	1822	1828	core promoter element around -30 of transcription start
CsDof82	TATA-box	1823	1827	core promoter element around -30 of transcription start
CsDof82	TATA-box	1836	1840	core promoter element around -30 of transcription start
CsDof82	TATA-box	1879	1885	core promoter element around -30 of transcription start
CsDof82	TATA-box	1880	1886	core promoter element around -30 of transcription start
CsDof82	TATA-box	1881	1885	core promoter element around -30 of transcription start
CsDof82	TATA-box	1958	1964	core promoter element around -30 of transcription start
CsDof82	TATA-box	1959	1964	core promoter element around -30 of transcription start
CsDof82	TATA-box	1960	1964	core promoter element around -30 of transcription start
CsDof82	HD-Zip 1	281	289	element involved in differentiation of the palisade mesophyll cells
CsDof82	GARE-motif	524	531	gibberellin-responsive element
CsDof82	GT1-motif	441	447	light responsive element
CsDof82	GT1-motif	740	746	light responsive element
CsDof82	MBS	237	243	MYB binding site involved in drought-inducibility
CsDof82	MBS	1121	1127	MYB binding site involved in drought-inducibility
CsDof82	MBS	1849	1855	MYB binding site involved in drought-inducibility
CsDof83	LTR	895	901	cis-acting element involved in low-temperature responsiveness
CsDof83	LTR	1048	1054	cis-acting element involved in low-temperature responsiveness
CsDof83	TCA-element	1913	1922	cis-acting element involved in salicylic acid responsiveness
CsDof83	ARE	273	279	cis-acting regulatory element essential for the anaerobic induction
CsDof83	TGACG-motif	50	55	cis-acting regulatory element involved in the MeJA-responsiveness
CsDof83	TGACG-motif	757	762	cis-acting regulatory element involved in the MeJA-responsiveness
CsDof83	CGTCA-motif	50	55	cis-acting regulatory element involved in the MeJA-responsiveness
CsDof83	CGTCA-motif	757	762	cis-acting regulatory element involved in the MeJA-responsiveness
CsDof83	O2-site	47	56	cis-acting regulatory element involved in zein metabolism regulation

Name	Cis-element	Start position	Stop position	Function
CsDof83	CAAT-box	103	108	common cis-acting element in promoter and enhancer regions
CsDof83	CAAT-box	451	456	common cis-acting element in promoter and enhancer regions
CsDof83	CAAT-box	646	651	common cis-acting element in promoter and enhancer regions
CsDof83	CAAT-box	1079	1084	common cis-acting element in promoter and enhancer regions
CsDof83	CAAT-box	1183	1188	common cis-acting element in promoter and enhancer regions
CsDof83	CAAT-box	1241	1246	common cis-acting element in promoter and enhancer regions
CsDof83	CAAT-box	1392	1397	common cis-acting element in promoter and enhancer regions
CsDof83	CAAT-box	1443	1448	common cis-acting element in promoter and enhancer regions
CsDof83	CAAT-box	1506	1511	common cis-acting element in promoter and enhancer regions
CsDof83	CAAT-box	1635	1640	common cis-acting element in promoter and enhancer regions
CsDof83	CAAT-box	1709	1714	common cis-acting element in promoter and enhancer regions
CsDof83	CAAT-box	1833	1838	common cis-acting element in promoter and enhancer regions
CsDof83	CAAT-box	1900	1905	common cis-acting element in promoter and enhancer regions
CsDof83	TATA-box	14	19	core promoter element around -30 of transcription start
CsDof83	TATA-box	15	19	core promoter element around -30 of transcription start
CsDof83	TATA-box	135	142	core promoter element around -30 of transcription start
CsDof83	TATA-box	136	142	core promoter element around -30 of transcription start
CsDof83	TATA-box	137	142	core promoter element around -30 of transcription start
CsDof83	TATA-box	138	142	core promoter element around -30 of transcription start
CsDof83	TATA-box	181	189	core promoter element around -30 of transcription start
CsDof83	TATA-box	188	194	core promoter element around -30 of transcription start
CsDof83	TATA-box	189	194	core promoter element around -30 of transcription start
CsDof83	TATA-box	190	194	core promoter element around -30 of transcription start
CsDof83	TATA-box	218	225	core promoter element around -30 of transcription start
CsDof83	TATA-box	219	225	core promoter element around -30 of transcription start
CsDof83	TATA-box	220	227	core promoter element around -30 of transcription start
CsDof83	TATA-box	221	227	core promoter element around -30 of transcription start
CsDof83	TATA-box	222	228	core promoter element around -30 of transcription start
CsDof83	TATA-box	223	227	core promoter element around -30 of transcription start
CsDof83	TATA-box	318	325	core promoter element around -30 of transcription start
CsDof83	TATA-box	634	641	core promoter element around -30 of transcription start
CsDof83	TATA-box	635	641	core promoter element around -30 of transcription start
CsDof83	TATA-box	636	641	core promoter element around -30 of transcription start
CsDof83	TATA-box	637	641	core promoter element around -30 of transcription start
CsDof83	TATA-box	699	706	core promoter element around -30 of transcription start
CsDof83	TATA-box	709	713	core promoter element around -30 of transcription start
CsDof83	TATA-box	1207	1211	core promoter element around -30 of transcription start
CsDof83	TATA-box	1268	1274	core promoter element around -30 of transcription start
CsDof83	TATA-box	1269	1273	core promoter element around -30 of transcription start
CsDof83	TATA-box	1342	1348	core promoter element around -30 of transcription start
CsDof83	TATA-box	1343	1347	core promoter element around -30 of transcription start
CsDof83	TATA-box	1412	1417	core promoter element around -30 of transcription start
CsDof83	TATA-box	1413	1417	core promoter element around -30 of transcription start
CsDof83	TATA-box	1433	1441	core promoter element around -30 of transcription start
CsDof83	TATA-box	1450	1457	core promoter element around -30 of transcription start
CsDof83	TATA-box	1515	1522	core promoter element around -30 of transcription start
CsDof83	TATA-box	1516	1522	core promoter element around -30 of transcription start
CsDof83	TATA-box	1517	1523	core promoter element around -30 of transcription start
CsDof83	TATA-box	1518	1522	core promoter element around -30 of transcription start
CsDof83	TATA-box	1559	1563	core promoter element around -30 of transcription start
CsDof83	TATA-box	1865	1871	core promoter element around -30 of transcription start

Name	Cis-element	Start position	Stop position	Function
CsDof83	TATA-box	1867	1871	core promoter element around -30 of transcription start
CsDof83	TATA-box	1920	1929	core promoter element around -30 of transcription start
CsDof83	GARE-motif	1282	1289	gibberellin-responsive element
CsDof83	GARE-motif	1290	1297	gibberellin-responsive element
CsDof83	WUN-motif	108	117	wound-responsive element
CsDof84	ABRE	186	191	abscisic acid responsiveness
CsDof84	ABRE	410	416	abscisic acid responsiveness
CsDof84	ABRE	411	416	abscisic acid responsiveness
CsDof84	ABRE	847	852	abscisic acid responsiveness
CsDof84	ABRE	1109	1114	abscisic acid responsiveness
CsDof84	TGA-element	1802	1808	auxin-responsive element
CsDof84	AT-rich element	1309	1319	binding site of AT-rich DNA binding protein (ATBP-1)
CsDof84	TCA-element	712	721	cis-acting element involved in salicylic acid responsiveness
CsDof84	A-box	321	327	cis-acting regulatory element
CsDof84	A-box	509	515	cis-acting regulatory element
CsDof84	ARE	741	747	cis-acting regulatory element essential for the anaerobic induction
CsDof84	ARE	1162	1168	cis-acting regulatory element essential for the anaerobic induction
CsDof84	ARE	1366	1372	cis-acting regulatory element essential for the anaerobic induction
CsDof84	G-Box	186	192	cis-acting regulatory element involved in light responsiveness
CsDof84	G-Box	410	416	cis-acting regulatory element involved in light responsiveness
CsDof84	G-box	410	416	cis-acting regulatory element involved in light responsiveness
CsDof84	G-box	846	852	cis-acting regulatory element involved in light responsiveness
CsDof84	G-box	1108	1114	cis-acting regulatory element involved in light responsiveness
CsDof84	CGTCA-motif	365	370	cis-acting regulatory element involved in the MeJA-responsiveness
CsDof84	CGTCA-motif	922	927	cis-acting regulatory element involved in the MeJA-responsiveness
CsDof84	CGTCA-motif	946	951	cis-acting regulatory element involved in the MeJA-responsiveness
CsDof84	TGACG-motif	365	370	cis-acting regulatory element involved in the MeJA-responsiveness
CsDof84	TGACG-motif	922	927	cis-acting regulatory element involved in the MeJA-responsiveness
CsDof84	TGACG-motif	946	951	cis-acting regulatory element involved in the MeJA-responsiveness
CsDof84	CAAT-box	131	136	common cis-acting element in promoter and enhancer regions
CsDof84	CAAT-box	211	216	common cis-acting element in promoter and enhancer regions
CsDof84	CAAT-box	265	270	common cis-acting element in promoter and enhancer regions
CsDof84	CAAT-box	273	278	common cis-acting element in promoter and enhancer regions
CsDof84	CAAT-box	346	351	common cis-acting element in promoter and enhancer regions
CsDof84	CAAT-box	441	446	common cis-acting element in promoter and enhancer regions
CsDof84	CAAT-box	529	534	common cis-acting element in promoter and enhancer regions
CsDof84	CAAT-box	550	555	common cis-acting element in promoter and enhancer regions
CsDof84	CAAT-box	630	635	common cis-acting element in promoter and enhancer regions
CsDof84	CAAT-box	771	776	common cis-acting element in promoter and enhancer regions
CsDof84	CAAT-box	819	824	common cis-acting element in promoter and enhancer regions
CsDof84	CAAT-box	1050	1055	common cis-acting element in promoter and enhancer regions
CsDof84	CAAT-box	1076	1081	common cis-acting element in promoter and enhancer regions
CsDof84	CAAT-box	1094	1099	common cis-acting element in promoter and enhancer regions
CsDof84	CAAT-box	1303	1308	common cis-acting element in promoter and enhancer regions
CsDof84	CAAT-box	1339	1344	common cis-acting element in promoter and enhancer regions
CsDof84	CAAT-box	1396	1401	common cis-acting element in promoter and enhancer regions
CsDof84	CAAT-box	1427	1432	common cis-acting element in promoter and enhancer regions
CsDof84	CAAT-box	1482	1487	common cis-acting element in promoter and enhancer regions
CsDof84	CAAT-box	1872	1877	common cis-acting element in promoter and enhancer regions
CsDof84	CAAT-box	1947	1952	common cis-acting element in promoter and enhancer regions
CsDof84	TATA-box	18	24	core promoter element around -30 of transcription start

Name	Cis-element	Start position	Stop position	Function
CsDof84	TATA-box	19	24	core promoter element around -30 of transcription start
CsDof84	TATA-box	20	24	core promoter element around -30 of transcription start
CsDof84	TATA-box	98	106	core promoter element around -30 of transcription start
CsDof84	TATA-box	102	110	core promoter element around -30 of transcription start
CsDof84	TATA-box	115	119	core promoter element around -30 of transcription start
CsDof84	TATA-box	164	168	core promoter element around -30 of transcription start
CsDof84	TATA-box	695	702	core promoter element around -30 of transcription start
CsDof84	TATA-box	696	702	core promoter element around -30 of transcription start
CsDof84	TATA-box	697	704	core promoter element around -30 of transcription start
CsDof84	TATA-box	698	704	core promoter element around -30 of transcription start
CsDof84	TATA-box	699	705	core promoter element around -30 of transcription start
CsDof84	TATA-box	700	704	core promoter element around -30 of transcription start
CsDof84	TATA-box	780	786	core promoter element around -30 of transcription start
CsDof84	TATA-box	782	786	core promoter element around -30 of transcription start
CsDof84	TATA-box	1004	1010	core promoter element around -30 of transcription start
CsDof84	TATA-box	1005	1012	core promoter element around -30 of transcription start
CsDof84	TATA-box	1006	1012	core promoter element around -30 of transcription start
CsDof84	TATA-box	1007	1013	core promoter element around -30 of transcription start
CsDof84	TATA-box	1008	1012	core promoter element around -30 of transcription start
CsDof84	TATA-box	1211	1215	core promoter element around -30 of transcription start
CsDof84	TATA-box	1538	1544	core promoter element around -30 of transcription start
CsDof84	TATA-box	1539	1543	core promoter element around -30 of transcription start
CsDof84	TATA-box	1646	1653	core promoter element around -30 of transcription start
CsDof84	TATA-box	1684	1692	core promoter element around -30 of transcription start
CsDof84	TATA-box	1760	1764	core promoter element around -30 of transcription start
CsDof84	3-AF1 binding site	44	54	light responsive element
CsDof84	GT1-motif	1288	1294	light responsive element
CsDof84	GT1-motif	1448	1454	light responsive element
CsDof84	GT1-motif	1814	1820	light responsive element
CsDof84	Sp1	890	896	light responsive element
CsDof84	MRE	961	968	MYB binding site involved in light responsiveness
CsDof85	ARE	83	89	cis-acting regulatory element essential for the anaerobic induction
CsDof85	ARE	801	807	cis-acting regulatory element essential for the anaerobic induction
CsDof85	ARE	1204	1210	cis-acting regulatory element essential for the anaerobic induction
CsDof85	ARE	1486	1492	cis-acting regulatory element essential for the anaerobic induction
CsDof85	circadian	559	568	cis-acting regulatory element involved in circadian control
CsDof85	G-box	47	53	cis-acting regulatory element involved in light responsiveness
CsDof85	CGTCA-motif	592	597	cis-acting regulatory element involved in the MeJA-responsiveness
CsDof85	TGACG-motif	592	597	cis-acting regulatory element involved in the MeJA-responsiveness
CsDof85	CAAT-box	95	100	common cis-acting element in promoter and enhancer regions
CsDof85	CAAT-box	163	168	common cis-acting element in promoter and enhancer regions
CsDof85	CAAT-box	274	279	common cis-acting element in promoter and enhancer regions
CsDof85	CAAT-box	347	352	common cis-acting element in promoter and enhancer regions
CsDof85	CAAT-box	753	758	common cis-acting element in promoter and enhancer regions
CsDof85	CAAT-box	832	837	common cis-acting element in promoter and enhancer regions
CsDof85	CAAT-box	860	865	common cis-acting element in promoter and enhancer regions
CsDof85	CAAT-box	905	910	common cis-acting element in promoter and enhancer regions
CsDof85	CAAT-box	1550	1555	common cis-acting element in promoter and enhancer regions
CsDof85	CAAT-box	1698	1703	common cis-acting element in promoter and enhancer regions
CsDof85	CAAT-box	1805	1810	common cis-acting element in promoter and enhancer regions
CsDof85	TATA-box	8	15	core promoter element around -30 of transcription start

Name	Cis-element	Start position	Stop position	Function
CsDof85	TATA-box	9	15	core promoter element around -30 of transcription start
CsDof85	TATA-box	10	15	core promoter element around -30 of transcription start
CsDof85	TATA-box	11	15	core promoter element around -30 of transcription start
CsDof85	TATA-box	26	32	core promoter element around -30 of transcription start
CsDof85	TATA-box	27	33	core promoter element around -30 of transcription start
CsDof85	TATA-box	28	34	core promoter element around -30 of transcription start
CsDof85	TATA-box	29	35	core promoter element around -30 of transcription start
CsDof85	TATA-box	30	36	core promoter element around -30 of transcription start
CsDof85	TATA-box	31	37	core promoter element around -30 of transcription start
CsDof85	TATA-box	32	36	core promoter element around -30 of transcription start
CsDof85	TATA-box	42	46	core promoter element around -30 of transcription start
CsDof85	TATA-box	87	95	core promoter element around -30 of transcription start
CsDof85	TATA-box	90	95	core promoter element around -30 of transcription start
CsDof85	TATA-box	91	95	core promoter element around -30 of transcription start
CsDof85	TATA-box	232	238	core promoter element around -30 of transcription start
CsDof85	TATA-box	234	238	core promoter element around -30 of transcription start
CsDof85	TATA-box	392	398	core promoter element around -30 of transcription start
CsDof85	TATA-box	393	399	core promoter element around -30 of transcription start
CsDof85	TATA-box	394	400	core promoter element around -30 of transcription start
CsDof85	TATA-box	395	399	core promoter element around -30 of transcription start
CsDof85	TATA-box	404	413	core promoter element around -30 of transcription start
CsDof85	TATA-box	405	411	core promoter element around -30 of transcription start
CsDof85	TATA-box	406	413	core promoter element around -30 of transcription start
CsDof85	TATA-box	407	413	core promoter element around -30 of transcription start
CsDof85	TATA-box	408	414	core promoter element around -30 of transcription start
CsDof85	TATA-box	409	413	core promoter element around -30 of transcription start
CsDof85	TATA-box	432	437	core promoter element around -30 of transcription start
CsDof85	TATA-box	433	437	core promoter element around -30 of transcription start
CsDof85	TATA-box	530	536	core promoter element around -30 of transcription start
CsDof85	TATA-box	531	535	core promoter element around -30 of transcription start
CsDof85	TATA-box	611	617	core promoter element around -30 of transcription start
CsDof85	TATA-box	612	616	core promoter element around -30 of transcription start
CsDof85	TATA-box	624	631	core promoter element around -30 of transcription start
CsDof85	TATA-box	648	652	core promoter element around -30 of transcription start
CsDof85	TATA-box	673	680	core promoter element around -30 of transcription start
CsDof85	TATA-box	718	724	core promoter element around -30 of transcription start
CsDof85	TATA-box	719	723	core promoter element around -30 of transcription start
CsDof85	TATA-box	729	735	core promoter element around -30 of transcription start
CsDof85	TATA-box	731	735	core promoter element around -30 of transcription start
CsDof85	TATA-box	761	767	core promoter element around -30 of transcription start
CsDof85	TATA-box	762	768	core promoter element around -30 of transcription start
CsDof85	TATA-box	764	768	core promoter element around -30 of transcription start
CsDof85	TATA-box	883	887	core promoter element around -30 of transcription start
CsDof85	TATA-box	952	961	core promoter element around -30 of transcription start
CsDof85	TATA-box	961	967	core promoter element around -30 of transcription start
CsDof85	TATA-box	962	968	core promoter element around -30 of transcription start
CsDof85	TATA-box	963	969	core promoter element around -30 of transcription start
CsDof85	TATA-box	964	968	core promoter element around -30 of transcription start
CsDof85	TATA-box	1069	1075	core promoter element around -30 of transcription start
CsDof85	TATA-box	1070	1075	core promoter element around -30 of transcription start
CsDof85	TATA-box	1071	1075	core promoter element around -30 of transcription start

Name	Cis-element	Start position	Stop position	Function
CsDof85	TATA-box	1348	1352	core promoter element around -30 of transcription start
CsDof85	TATA-box	1404	1410	core promoter element around -30 of transcription start
CsDof85	TATA-box	1405	1410	core promoter element around -30 of transcription start
CsDof85	TATA-box	1406	1410	core promoter element around -30 of transcription start
CsDof85	TATA-box	1419	1431	core promoter element around -30 of transcription start
CsDof85	TATA-box	1421	1427	core promoter element around -30 of transcription start
CsDof85	TATA-box	1422	1426	core promoter element around -30 of transcription start
CsDof85	TATA-box	1427	1433	core promoter element around -30 of transcription start
CsDof85	TATA-box	1428	1434	core promoter element around -30 of transcription start
CsDof85	TATA-box	1430	1434	core promoter element around -30 of transcription start
CsDof85	TATA-box	1440	1446	core promoter element around -30 of transcription start
CsDof85	TATA-box	1442	1446	core promoter element around -30 of transcription start
CsDof85	TATA-box	1487	1496	core promoter element around -30 of transcription start
CsDof85	TATA-box	1489	1495	core promoter element around -30 of transcription start
CsDof85	TATA-box	1490	1497	core promoter element around -30 of transcription start
CsDof85	TATA-box	1491	1497	core promoter element around -30 of transcription start
CsDof85	TATA-box	1492	1498	core promoter element around -30 of transcription start
CsDof85	TATA-box	1493	1499	core promoter element around -30 of transcription start
CsDof85	TATA-box	1494	1500	core promoter element around -30 of transcription start
CsDof85	TATA-box	1495	1501	core promoter element around -30 of transcription start
CsDof85	TATA-box	1496	1502	core promoter element around -30 of transcription start
CsDof85	TATA-box	1497	1503	core promoter element around -30 of transcription start
CsDof85	TATA-box	1498	1504	core promoter element around -30 of transcription start
CsDof85	TATA-box	1499	1505	core promoter element around -30 of transcription start
CsDof85	TATA-box	1500	1506	core promoter element around -30 of transcription start
CsDof85	TATA-box	1501	1507	core promoter element around -30 of transcription start
CsDof85	TATA-box	1502	1508	core promoter element around -30 of transcription start
CsDof85	TATA-box	1503	1509	core promoter element around -30 of transcription start
CsDof85	TATA-box	1505	1509	core promoter element around -30 of transcription start
CsDof85	TATA-box	1512	1519	core promoter element around -30 of transcription start
CsDof85	TATA-box	1513	1519	core promoter element around -30 of transcription start
CsDof85	TATA-box	1514	1519	core promoter element around -30 of transcription start
CsDof85	TATA-box	1515	1519	core promoter element around -30 of transcription start
CsDof85	TATA-box	1530	1536	core promoter element around -30 of transcription start
CsDof85	TATA-box	1531	1538	core promoter element around -30 of transcription start
CsDof85	TATA-box	1532	1538	core promoter element around -30 of transcription start
CsDof85	TATA-box	1533	1539	core promoter element around -30 of transcription start
CsDof85	TATA-box	1534	1538	core promoter element around -30 of transcription start
CsDof85	TATA-box	1558	1565	core promoter element around -30 of transcription start
CsDof85	TATA-box	1559	1565	core promoter element around -30 of transcription start
CsDof85	TATA-box	1560	1567	core promoter element around -30 of transcription start
CsDof85	TATA-box	1561	1567	core promoter element around -30 of transcription start
CsDof85	TATA-box	1562	1568	core promoter element around -30 of transcription start
CsDof85	TATA-box	1563	1567	core promoter element around -30 of transcription start
CsDof85	TATA-box	1631	1637	core promoter element around -30 of transcription start
CsDof85	TATA-box	1632	1637	core promoter element around -30 of transcription start
CsDof85	TATA-box	1633	1637	core promoter element around -30 of transcription start
CsDof85	TATA-box	1660	1667	core promoter element around -30 of transcription start
CsDof85	TATA-box	1661	1667	core promoter element around -30 of transcription start
CsDof85	TATA-box	1662	1667	core promoter element around -30 of transcription start
CsDof85	TATA-box	1663	1667	core promoter element around -30 of transcription start

Name	Cis-element	Start position	Stop position	Function
CsDof85	TATA-box	1833	1837	core promoter element around -30 of transcription start
CsDof85	TATA-box	1838	1844	core promoter element around -30 of transcription start
CsDof85	TATA-box	1839	1845	core promoter element around -30 of transcription start
CsDof85	TATA-box	1840	1846	core promoter element around -30 of transcription start
CsDof85	TATA-box	1841	1847	core promoter element around -30 of transcription start
CsDof85	TATA-box	1842	1848	core promoter element around -30 of transcription start
CsDof85	TATA-box	1843	1849	core promoter element around -30 of transcription start
CsDof85	TATA-box	1844	1850	core promoter element around -30 of transcription start
CsDof85	TATA-box	1845	1851	core promoter element around -30 of transcription start
CsDof85	TATA-box	1847	1851	core promoter element around -30 of transcription start
CsDof85	TATA-box	1853	1859	core promoter element around -30 of transcription start
CsDof85	TATA-box	1855	1859	core promoter element around -30 of transcription start
CsDof85	TATA-box	1875	1879	core promoter element around -30 of transcription start
CsDof85	TATA-box	1939	1943	core promoter element around -30 of transcription start
CsDof85	P-box	1042	1049	gibberellin-responsive element
CsDof85	P-box	1710	1717	gibberellin-responsive element
CsDof85	P-box	1817	1824	gibberellin-responsive element
CsDof85	Sp1	636	642	light responsive element
CsDof85	GT1-motif	1813	1819	light responsive element
CsDof85	MRE	518	525	MYB binding site involved in light responsiveness
CsDof86	ABRE	1495	1500	abscisic acid responsiveness
CsDof86	ABRE	1588	1593	abscisic acid responsiveness
CsDof86	ABRE	1750	1755	abscisic acid responsiveness
CsDof86	MSA-like	36	45	cis-acting element involved in cell cycle regulation
CsDof86	ARE	1637	1643	cis-acting regulatory element essential for the anaerobic induction
CsDof86	G-Box	1495	1501	cis-acting regulatory element involved in light responsiveness
CsDof86	G-Box	1587	1593	cis-acting regulatory element involved in light responsiveness
CsDof86	G-box	1749	1755	cis-acting regulatory element involved in light responsiveness
CsDof86	CGTCA-motif	1157	1162	cis-acting regulatory element involved in the MeJA-responsiveness
CsDof86	TGACG-motif	1157	1162	cis-acting regulatory element involved in the MeJA-responsiveness
CsDof86	CAAT-box	276	281	common cis-acting element in promoter and enhancer regions
CsDof86	CAAT-box	319	324	common cis-acting element in promoter and enhancer regions
CsDof86	CAAT-box	545	550	common cis-acting element in promoter and enhancer regions
CsDof86	CAAT-box	597	602	common cis-acting element in promoter and enhancer regions
CsDof86	CAAT-box	936	941	common cis-acting element in promoter and enhancer regions
CsDof86	CAAT-box	999	1004	common cis-acting element in promoter and enhancer regions
CsDof86	CAAT-box	1285	1290	common cis-acting element in promoter and enhancer regions
CsDof86	CAAT-box	1339	1344	common cis-acting element in promoter and enhancer regions
CsDof86	CAAT-box	1371	1376	common cis-acting element in promoter and enhancer regions
CsDof86	CAAT-box	1465	1470	common cis-acting element in promoter and enhancer regions
CsDof86	CAAT-box	1664	1669	common cis-acting element in promoter and enhancer regions
CsDof86	CAAT-box	1687	1692	common cis-acting element in promoter and enhancer regions
CsDof86	CAAT-box	1900	1905	common cis-acting element in promoter and enhancer regions
CsDof86	TATA-box	51	57	core promoter element around -30 of transcription start
CsDof86	TATA-box	52	57	core promoter element around -30 of transcription start
CsDof86	TATA-box	53	57	core promoter element around -30 of transcription start
CsDof86	TATA-box	76	82	core promoter element around -30 of transcription start
CsDof86	TATA-box	78	82	core promoter element around -30 of transcription start
CsDof86	TATA-box	97	101	core promoter element around -30 of transcription start
CsDof86	TATA-box	142	148	core promoter element around -30 of transcription start
CsDof86	TATA-box	143	149	core promoter element around -30 of transcription start

Name	Cis-element	Start position	Stop position	Function
CsDof86	TATA-box	144	150	core promoter element around -30 of transcription start
CsDof86	TATA-box	145	149	core promoter element around -30 of transcription start
CsDof86	TATA-box	315	321	core promoter element around -30 of transcription start
CsDof86	TATA-box	316	320	core promoter element around -30 of transcription start
CsDof86	TATA-box	330	336	core promoter element around -30 of transcription start
CsDof86	TATA-box	331	336	core promoter element around -30 of transcription start
CsDof86	TATA-box	332	336	core promoter element around -30 of transcription start
CsDof86	TATA-box	379	385	core promoter element around -30 of transcription start
CsDof86	TATA-box	381	385	core promoter element around -30 of transcription start
CsDof86	TATA-box	428	432	core promoter element around -30 of transcription start
CsDof86	TATA-box	627	633	core promoter element around -30 of transcription start
CsDof86	TATA-box	628	632	core promoter element around -30 of transcription start
CsDof86	TATA-box	652	658	core promoter element around -30 of transcription start
CsDof86	TATA-box	653	657	core promoter element around -30 of transcription start
CsDof86	TATA-box	725	731	core promoter element around -30 of transcription start
CsDof86	TATA-box	726	732	core promoter element around -30 of transcription start
CsDof86	TATA-box	728	732	core promoter element around -30 of transcription start
CsDof86	TATA-box	814	821	core promoter element around -30 of transcription start
CsDof86	TATA-box	815	821	core promoter element around -30 of transcription start
CsDof86	TATA-box	816	821	core promoter element around -30 of transcription start
CsDof86	TATA-box	817	821	core promoter element around -30 of transcription start
CsDof86	TATA-box	909	915	core promoter element around -30 of transcription start
CsDof86	TATA-box	910	922	core promoter element around -30 of transcription start
CsDof86	TATA-box	914	920	core promoter element around -30 of transcription start
CsDof86	TATA-box	916	920	core promoter element around -30 of transcription start
CsDof86	TATA-box	1025	1032	core promoter element around -30 of transcription start
CsDof86	TATA-box	1078	1084	core promoter element around -30 of transcription start
CsDof86	TATA-box	1079	1083	core promoter element around -30 of transcription start
CsDof86	TATA-box	1135	1141	core promoter element around -30 of transcription start
CsDof86	TATA-box	1136	1142	core promoter element around -30 of transcription start
CsDof86	TATA-box	1137	1143	core promoter element around -30 of transcription start
CsDof86	TATA-box	1138	1144	core promoter element around -30 of transcription start
CsDof86	TATA-box	1139	1145	core promoter element around -30 of transcription start
CsDof86	TATA-box	1141	1145	core promoter element around -30 of transcription start
CsDof86	TATA-box	1185	1191	core promoter element around -30 of transcription start
CsDof86	TATA-box	1186	1190	core promoter element around -30 of transcription start
CsDof86	TATA-box	1203	1210	core promoter element around -30 of transcription start
CsDof86	TATA-box	1204	1210	core promoter element around -30 of transcription start
CsDof86	TATA-box	1205	1211	core promoter element around -30 of transcription start
CsDof86	TATA-box	1206	1210	core promoter element around -30 of transcription start
CsDof86	TATA-box	1223	1231	core promoter element around -30 of transcription start
CsDof86	TATA-box	1244	1252	core promoter element around -30 of transcription start
CsDof86	TATA-box	1255	1262	core promoter element around -30 of transcription start
CsDof86	TATA-box	1256	1262	core promoter element around -30 of transcription start
CsDof86	TATA-box	1257	1263	core promoter element around -30 of transcription start
CsDof86	TATA-box	1258	1262	core promoter element around -30 of transcription start
CsDof86	TATA-box	1292	1299	core promoter element around -30 of transcription start
CsDof86	TATA-box	1300	1306	core promoter element around -30 of transcription start
CsDof86	TATA-box	1301	1305	core promoter element around -30 of transcription start
CsDof86	TATA-box	1354	1361	core promoter element around -30 of transcription start
CsDof86	TATA-box	1405	1411	core promoter element around -30 of transcription start

Name	Cis-element	Start position	Stop position	Function
CsDof86	TATA-box	1406	1412	core promoter element around -30 of transcription start
CsDof86	TATA-box	1407	1413	core promoter element around -30 of transcription start
CsDof86	TATA-box	1408	1412	core promoter element around -30 of transcription start
CsDof86	TATA-box	1418	1424	core promoter element around -30 of transcription start
CsDof86	TATA-box	1419	1425	core promoter element around -30 of transcription start
CsDof86	TATA-box	1420	1426	core promoter element around -30 of transcription start
CsDof86	TATA-box	1421	1425	core promoter element around -30 of transcription start
CsDof86	TATA-box	1451	1455	core promoter element around -30 of transcription start
CsDof86	TATA-box	1543	1550	core promoter element around -30 of transcription start
CsDof86	TATA-box	1544	1550	core promoter element around -30 of transcription start
CsDof86	TATA-box	1545	1550	core promoter element around -30 of transcription start
CsDof86	TATA-box	1546	1550	core promoter element around -30 of transcription start
CsDof86	TATA-box	1554	1563	core promoter element around -30 of transcription start
CsDof86	TATA-box	1613	1617	core promoter element around -30 of transcription start
CsDof86	TATA-box	1715	1722	core promoter element around -30 of transcription start
CsDof86	TATA-box	1814	1818	core promoter element around -30 of transcription start
CsDof86	TATA-box	1913	1918	core promoter element around -30 of transcription start
CsDof86	TATA-box	1914	1918	core promoter element around -30 of transcription start
CsDof86	TATA-box	1928	1938	core promoter element around -30 of transcription start
CsDof86	TATA-box	1930	1934	core promoter element around -30 of transcription start
CsDof86	GT1-motif	877	884	light responsive element
CsDof86	GT1-motif	878	884	light responsive element
CsDof86	GT1-motif	1318	1325	light responsive element
CsDof86	MBS	8	14	MYB binding site involved in drought-inducibility
CsDof86	MRE	201	208	MYB binding site involved in light responsiveness
CsDof86	CCAAT-box	38	44	MYBHv1 binding site
CsDof87	TC-rich repeats	1794	1803	cis-acting element involved in defense and stress responsiveness
CsDof87	TATC-box	823	830	cis-acting element involved in gibberellin-responsiveness
CsDof87	ACE	888	897	cis-acting element involved in light responsiveness
CsDof87	ACE	1362	1371	cis-acting element involved in light responsiveness
CsDof87	TCA-element	1256	1265	cis-acting element involved in salicylic acid responsiveness
CsDof87	A-box	873	879	cis-acting regulatory element
CsDof87	ARE	572	578	cis-acting regulatory element essential for the anaerobic induction
CsDof87	ARE	627	633	cis-acting regulatory element essential for the anaerobic induction
CsDof87	ARE	678	684	cis-acting regulatory element essential for the anaerobic induction
CsDof87	ARE	1338	1344	cis-acting regulatory element essential for the anaerobic induction
CsDof87	CAAT-box	24	29	common cis-acting element in promoter and enhancer regions
CsDof87	CAAT-box	45	50	common cis-acting element in promoter and enhancer regions
CsDof87	CAAT-box	570	575	common cis-acting element in promoter and enhancer regions
CsDof87	CAAT-box	676	681	common cis-acting element in promoter and enhancer regions
CsDof87	CAAT-box	905	910	common cis-acting element in promoter and enhancer regions
CsDof87	CAAT-box	1058	1063	common cis-acting element in promoter and enhancer regions
CsDof87	CAAT-box	1154	1159	common cis-acting element in promoter and enhancer regions
CsDof87	CAAT-box	1265	1270	common cis-acting element in promoter and enhancer regions
CsDof87	CAAT-box	1437	1442	common cis-acting element in promoter and enhancer regions
CsDof87	CAAT-box	1687	1692	common cis-acting element in promoter and enhancer regions
CsDof87	TATA-box	69	73	core promoter element around -30 of transcription start
CsDof87	TATA-box	75	81	core promoter element around -30 of transcription start
CsDof87	TATA-box	76	81	core promoter element around -30 of transcription start
CsDof87	TATA-box	77	81	core promoter element around -30 of transcription start
CsDof87	TATA-box	112	118	core promoter element around -30 of transcription start

Name	Cis-element	Start position	Stop position	Function
CsDof87	TATA-box	113	119	core promoter element around -30 of transcription start
CsDof87	TATA-box	115	119	core promoter element around -30 of transcription start
CsDof87	TATA-box	126	130	core promoter element around -30 of transcription start
CsDof87	TATA-box	155	161	core promoter element around -30 of transcription start
CsDof87	TATA-box	156	161	core promoter element around -30 of transcription start
CsDof87	TATA-box	157	161	core promoter element around -30 of transcription start
CsDof87	TATA-box	175	181	core promoter element around -30 of transcription start
CsDof87	TATA-box	176	180	core promoter element around -30 of transcription start
CsDof87	TATA-box	201	209	core promoter element around -30 of transcription start
CsDof87	TATA-box	202	209	core promoter element around -30 of transcription start
CsDof87	TATA-box	203	209	core promoter element around -30 of transcription start
CsDof87	TATA-box	204	209	core promoter element around -30 of transcription start
CsDof87	TATA-box	205	209	core promoter element around -30 of transcription start
CsDof87	TATA-box	214	220	core promoter element around -30 of transcription start
CsDof87	TATA-box	216	220	core promoter element around -30 of transcription start
CsDof87	TATA-box	226	232	core promoter element around -30 of transcription start
CsDof87	TATA-box	227	232	core promoter element around -30 of transcription start
CsDof87	TATA-box	228	232	core promoter element around -30 of transcription start
CsDof87	TATA-box	283	289	core promoter element around -30 of transcription start
CsDof87	TATA-box	284	288	core promoter element around -30 of transcription start
CsDof87	TATA-box	298	302	core promoter element around -30 of transcription start
CsDof87	TATA-box	366	370	core promoter element around -30 of transcription start
CsDof87	TATA-box	415	421	core promoter element around -30 of transcription start
CsDof87	TATA-box	416	421	core promoter element around -30 of transcription start
CsDof87	TATA-box	417	421	core promoter element around -30 of transcription start
CsDof87	TATA-box	451	455	core promoter element around -30 of transcription start
CsDof87	TATA-box	475	481	core promoter element around -30 of transcription start
CsDof87	TATA-box	476	481	core promoter element around -30 of transcription start
CsDof87	TATA-box	477	481	core promoter element around -30 of transcription start
CsDof87	TATA-box	485	489	core promoter element around -30 of transcription start
CsDof87	TATA-box	670	674	core promoter element around -30 of transcription start
CsDof87	TATA-box	736	742	core promoter element around -30 of transcription start
CsDof87	TATA-box	737	744	core promoter element around -30 of transcription start
CsDof87	TATA-box	738	744	core promoter element around -30 of transcription start
CsDof87	TATA-box	739	745	core promoter element around -30 of transcription start
CsDof87	TATA-box	740	746	core promoter element around -30 of transcription start
CsDof87	TATA-box	741	747	core promoter element around -30 of transcription start
CsDof87	TATA-box	742	748	core promoter element around -30 of transcription start
CsDof87	TATA-box	743	749	core promoter element around -30 of transcription start
CsDof87	TATA-box	744	756	core promoter element around -30 of transcription start
CsDof87	TATA-box	745	751	core promoter element around -30 of transcription start
CsDof87	TATA-box	746	752	core promoter element around -30 of transcription start
CsDof87	TATA-box	747	753	core promoter element around -30 of transcription start
CsDof87	TATA-box	748	754	core promoter element around -30 of transcription start
CsDof87	TATA-box	749	755	core promoter element around -30 of transcription start
CsDof87	TATA-box	750	754	core promoter element around -30 of transcription start
CsDof87	TATA-box	756	763	core promoter element around -30 of transcription start
CsDof87	TATA-box	757	763	core promoter element around -30 of transcription start
CsDof87	TATA-box	758	765	core promoter element around -30 of transcription start
CsDof87	TATA-box	759	765	core promoter element around -30 of transcription start
CsDof87	TATA-box	760	766	core promoter element around -30 of transcription start

Name	Cis-element	Start position	Stop position	Function
CsDof87	TATA-box	761	765	core promoter element around -30 of transcription start
CsDof87	TATA-box	765	771	core promoter element around -30 of transcription start
CsDof87	TATA-box	766	778	core promoter element around -30 of transcription start
CsDof87	TATA-box	768	776	core promoter element around -30 of transcription start
CsDof87	TATA-box	769	776	core promoter element around -30 of transcription start
CsDof87	TATA-box	770	776	core promoter element around -30 of transcription start
CsDof87	TATA-box	771	776	core promoter element around -30 of transcription start
CsDof87	TATA-box	772	776	core promoter element around -30 of transcription start
CsDof87	TATA-box	788	794	core promoter element around -30 of transcription start
CsDof87	TATA-box	789	795	core promoter element around -30 of transcription start
CsDof87	TATA-box	790	796	core promoter element around -30 of transcription start
CsDof87	TATA-box	791	797	core promoter element around -30 of transcription start
CsDof87	TATA-box	792	796	core promoter element around -30 of transcription start
CsDof87	TATA-box	807	813	core promoter element around -30 of transcription start
CsDof87	TATA-box	808	812	core promoter element around -30 of transcription start
CsDof87	TATA-box	819	825	core promoter element around -30 of transcription start
CsDof87	TATA-box	820	826	core promoter element around -30 of transcription start
CsDof87	TATA-box	821	825	core promoter element around -30 of transcription start
CsDof87	TATA-box	829	835	core promoter element around -30 of transcription start
CsDof87	TATA-box	830	836	core promoter element around -30 of transcription start
CsDof87	TATA-box	832	836	core promoter element around -30 of transcription start
CsDof87	TATA-box	852	856	core promoter element around -30 of transcription start
CsDof87	TATA-box	910	915	core promoter element around -30 of transcription start
CsDof87	TATA-box	911	915	core promoter element around -30 of transcription start
CsDof87	TATA-box	974	979	core promoter element around -30 of transcription start
CsDof87	TATA-box	975	979	core promoter element around -30 of transcription start
CsDof87	TATA-box	984	989	core promoter element around -30 of transcription start
CsDof87	TATA-box	985	989	core promoter element around -30 of transcription start
CsDof87	TATA-box	1021	1028	core promoter element around -30 of transcription start
CsDof87	TATA-box	1024	1030	core promoter element around -30 of transcription start
CsDof87	TATA-box	1026	1030	core promoter element around -30 of transcription start
CsDof87	TATA-box	1031	1038	core promoter element around -30 of transcription start
CsDof87	TATA-box	1061	1067	core promoter element around -30 of transcription start
CsDof87	TATA-box	1062	1068	core promoter element around -30 of transcription start
CsDof87	TATA-box	1063	1069	core promoter element around -30 of transcription start
CsDof87	TATA-box	1064	1068	core promoter element around -30 of transcription start
CsDof87	TATA-box	1097	1105	core promoter element around -30 of transcription start
CsDof87	TATA-box	1099	1107	core promoter element around -30 of transcription start
CsDof87	TATA-box	1105	1109	core promoter element around -30 of transcription start
CsDof87	TATA-box	1122	1128	core promoter element around -30 of transcription start
CsDof87	TATA-box	1123	1127	core promoter element around -30 of transcription start
CsDof87	TATA-box	1198	1206	core promoter element around -30 of transcription start
CsDof87	TATA-box	1215	1219	core promoter element around -30 of transcription start
CsDof87	TATA-box	1232	1238	core promoter element around -30 of transcription start
CsDof87	TATA-box	1233	1237	core promoter element around -30 of transcription start
CsDof87	TATA-box	1519	1527	core promoter element around -30 of transcription start
CsDof87	TATA-box	1527	1533	core promoter element around -30 of transcription start
CsDof87	TATA-box	1528	1534	core promoter element around -30 of transcription start
CsDof87	TATA-box	1530	1534	core promoter element around -30 of transcription start
CsDof87	TATA-box	1545	1551	core promoter element around -30 of transcription start
CsDof87	TATA-box	1546	1552	core promoter element around -30 of transcription start

Name	Cis-element	Start position	Stop position	Function
CsDof87	TATA-box	1547	1553	core promoter element around -30 of transcription start
CsDof87	TATA-box	1548	1554	core promoter element around -30 of transcription start
CsDof87	TATA-box	1549	1553	core promoter element around -30 of transcription start
CsDof87	TATA-box	1552	1564	core promoter element around -30 of transcription start
CsDof87	TATA-box	1554	1562	core promoter element around -30 of transcription start
CsDof87	TATA-box	1555	1562	core promoter element around -30 of transcription start
CsDof87	TATA-box	1556	1562	core promoter element around -30 of transcription start
CsDof87	TATA-box	1557	1562	core promoter element around -30 of transcription start
CsDof87	TATA-box	1558	1562	core promoter element around -30 of transcription start
CsDof87	TATA-box	1568	1574	core promoter element around -30 of transcription start
CsDof87	TATA-box	1569	1573	core promoter element around -30 of transcription start
CsDof87	TATA-box	1590	1595	core promoter element around -30 of transcription start
CsDof87	TATA-box	1591	1595	core promoter element around -30 of transcription start
CsDof87	TATA-box	1652	1658	core promoter element around -30 of transcription start
CsDof87	TATA-box	1653	1659	core promoter element around -30 of transcription start
CsDof87	TATA-box	1654	1660	core promoter element around -30 of transcription start
CsDof87	TATA-box	1655	1661	core promoter element around -30 of transcription start
CsDof87	TATA-box	1656	1662	core promoter element around -30 of transcription start
CsDof87	TATA-box	1657	1663	core promoter element around -30 of transcription start
CsDof87	TATA-box	1658	1664	core promoter element around -30 of transcription start
CsDof87	TATA-box	1659	1665	core promoter element around -30 of transcription start
CsDof87	TATA-box	1660	1666	core promoter element around -30 of transcription start
CsDof87	TATA-box	1661	1667	core promoter element around -30 of transcription start
CsDof87	TATA-box	1662	1668	core promoter element around -30 of transcription start
CsDof87	TATA-box	1663	1669	core promoter element around -30 of transcription start
CsDof87	TATA-box	1664	1670	core promoter element around -30 of transcription start
CsDof87	TATA-box	1665	1671	core promoter element around -30 of transcription start
CsDof87	TATA-box	1666	1672	core promoter element around -30 of transcription start
CsDof87	TATA-box	1667	1673	core promoter element around -30 of transcription start
CsDof87	TATA-box	1668	1674	core promoter element around -30 of transcription start
CsDof87	TATA-box	1669	1675	core promoter element around -30 of transcription start
CsDof87	TATA-box	1670	1676	core promoter element around -30 of transcription start
CsDof87	TATA-box	1671	1677	core promoter element around -30 of transcription start
CsDof87	TATA-box	1672	1678	core promoter element around -30 of transcription start
CsDof87	TATA-box	1673	1679	core promoter element around -30 of transcription start
CsDof87	TATA-box	1674	1680	core promoter element around -30 of transcription start
CsDof87	TATA-box	1675	1681	core promoter element around -30 of transcription start
CsDof87	TATA-box	1676	1682	core promoter element around -30 of transcription start
CsDof87	TATA-box	1677	1683	core promoter element around -30 of transcription start
CsDof87	TATA-box	1679	1683	core promoter element around -30 of transcription start
CsDof87	TATA-box	1714	1720	core promoter element around -30 of transcription start
CsDof87	TATA-box	1715	1721	core promoter element around -30 of transcription start
CsDof87	TATA-box	1716	1722	core promoter element around -30 of transcription start
CsDof87	TATA-box	1717	1723	core promoter element around -30 of transcription start
CsDof87	TATA-box	1718	1724	core promoter element around -30 of transcription start
CsDof87	TATA-box	1719	1723	core promoter element around -30 of transcription start
CsDof87	AT-rich sequence	1407	1416	element for maximal elicitor-mediated activation (2copies)
CsDof87	HD-Zip 1	604	612.5	element involved in differentiation of the palisade mesophyll cells
CsDof87	P-box	1841	1848	gibberellin-responsive element
CsDof88	ABRE	1855	1860	abscisic acid responsiveness
CsDof88	ARE	508	514	cis-acting regulatory element essential for the anaerobic induction

Name	Cis-element	Start position	Stop position	Function
CsDof88	ARE	1310	1316	cis-acting regulatory element essential for the anaerobic induction
CsDof88	ARE	1951	1957	cis-acting regulatory element essential for the anaerobic induction
CsDof88	circadian	1470	1479	cis-acting regulatory element involved in circadian control
CsDof88	G-box	1855	1861	cis-acting regulatory element involved in light responsiveness
CsDof88	TGACG-motif	1178	1183	cis-acting regulatory element involved in the MeJA-responsiveness
CsDof88	TGACG-motif	1886	1891	cis-acting regulatory element involved in the MeJA-responsiveness
CsDof88	CGTCA-motif	1178	1183	cis-acting regulatory element involved in the MeJA-responsiveness
CsDof88	CGTCA-motif	1886	1891	cis-acting regulatory element involved in the MeJA-responsiveness
CsDof88	motif I	1852	1862	cis-acting regulatory element root specific
CsDof88	CAAT-box	421	426	common cis-acting element in promoter and enhancer regions
CsDof88	CAAT-box	496	501	common cis-acting element in promoter and enhancer regions
CsDof88	CAAT-box	528	533	common cis-acting element in promoter and enhancer regions
CsDof88	CAAT-box	712	717	common cis-acting element in promoter and enhancer regions
CsDof88	CAAT-box	814	819	common cis-acting element in promoter and enhancer regions
CsDof88	CAAT-box	840	845	common cis-acting element in promoter and enhancer regions
CsDof88	CAAT-box	963	968	common cis-acting element in promoter and enhancer regions
CsDof88	CAAT-box	1585	1590	common cis-acting element in promoter and enhancer regions
CsDof88	CAAT-box	1609	1614	common cis-acting element in promoter and enhancer regions
CsDof88	CAAT-box	1620	1625	common cis-acting element in promoter and enhancer regions
CsDof88	CAAT-box	1725	1730	common cis-acting element in promoter and enhancer regions
CsDof88	CAAT-box	1728	1733	common cis-acting element in promoter and enhancer regions
CsDof88	CAAT-box	1911	1916	common cis-acting element in promoter and enhancer regions
CsDof88	CAAT-box	1958	1963	common cis-acting element in promoter and enhancer regions
CsDof88	TATA-box	12	19	core promoter element around -30 of transcription start
CsDof88	TATA-box	363	369	core promoter element around -30 of transcription start
CsDof88	TATA-box	364	368	core promoter element around -30 of transcription start
CsDof88	TATA-box	553	560	core promoter element around -30 of transcription start
CsDof88	TATA-box	621	628	core promoter element around -30 of transcription start
CsDof88	TATA-box	736	742	core promoter element around -30 of transcription start
CsDof88	TATA-box	737	742	core promoter element around -30 of transcription start
CsDof88	TATA-box	738	742	core promoter element around -30 of transcription start
CsDof88	TATA-box	748	752	core promoter element around -30 of transcription start
CsDof88	TATA-box	1108	1116	core promoter element around -30 of transcription start
CsDof88	TATA-box	1113	1119	core promoter element around -30 of transcription start
CsDof88	TATA-box	1114	1118	core promoter element around -30 of transcription start
CsDof88	TATA-box	1137	1143	core promoter element around -30 of transcription start
CsDof88	TATA-box	1138	1143	core promoter element around -30 of transcription start
CsDof88	TATA-box	1139	1143	core promoter element around -30 of transcription start
CsDof88	TATA-box	1198	1204	core promoter element around -30 of transcription start
CsDof88	TATA-box	1199	1203	core promoter element around -30 of transcription start
CsDof88	TATA-box	1213	1220	core promoter element around -30 of transcription start
CsDof88	TATA-box	1216	1222	core promoter element around -30 of transcription start
CsDof88	TATA-box	1218	1222	core promoter element around -30 of transcription start
CsDof88	TATA-box	1315	1321	core promoter element around -30 of transcription start
CsDof88	TATA-box	1316	1320	core promoter element around -30 of transcription start
CsDof88	TATA-box	1352	1361	core promoter element around -30 of transcription start
CsDof88	TATA-box	1353	1362	core promoter element around -30 of transcription start
CsDof88	TATA-box	1354	1360	core promoter element around -30 of transcription start
CsDof88	TATA-box	1355	1362	core promoter element around -30 of transcription start
CsDof88	TATA-box	1356	1362	core promoter element around -30 of transcription start
CsDof88	TATA-box	1357	1363	core promoter element around -30 of transcription start

Name	Cis-element	Start position	Stop position	Function
CsDof88	TATA-box	1358	1362	core promoter element around -30 of transcription start
CsDof88	TATA-box	1581	1585	core promoter element around -30 of transcription start
CsDof88	TATA-box	1872	1879	core promoter element around -30 of transcription start
CsDof88	TATA-box	1873	1879	core promoter element around -30 of transcription start
CsDof88	TATA-box	1874	1880	core promoter element around -30 of transcription start
CsDof88	TATA-box	1875	1879	core promoter element around -30 of transcription start
CsDof88	P-box	387	394	gibberellin-responsive element
CsDof88	P-box	1972	1979	gibberellin-responsive element
CsDof88	GT1-motif	473	479	light responsive element
CsDof88	GT1-motif	1027	1033	light responsive element
CsDof88	MRE	207	214	MYB binding site involved in light responsiveness
CsDof89	ABRE	923	932	abscisic acid responsiveness
CsDof89	ABRE	925	931	abscisic acid responsiveness
CsDof89	ABRE	926	931	abscisic acid responsiveness
CsDof89	ABRE	1120	1129	abscisic acid responsiveness
CsDof89	ABRE	1122	1128	abscisic acid responsiveness
CsDof89	ABRE	1123	1128	abscisic acid responsiveness
CsDof89	ABRE	1211	1217	abscisic acid responsiveness
CsDof89	ABRE	1212	1217	abscisic acid responsiveness
CsDof89	ABRE	1232	1237	abscisic acid responsiveness
CsDof89	ABRE	1282	1288	abscisic acid responsiveness
CsDof89	ABRE	1283	1288	abscisic acid responsiveness
CsDof89	AT-rich element	465	475	binding site of AT-rich DNA binding protein (ATBP-1)
CsDof89	TATC-box	43	50	cis-acting element involved in gibberellin-responsiveness
CsDof89	TATC-box	874	881	cis-acting element involved in gibberellin-responsiveness
CsDof89	LTR	1256	1262	cis-acting element involved in low-temperature responsiveness
CsDof89	ARE	1789	1795	cis-acting regulatory element essential for the anaerobic induction
CsDof89	AuxRR-core	1198	1205	cis-acting regulatory element involved in auxin responsiveness
CsDof89	G-box	922	934	cis-acting regulatory element involved in light responsiveness
CsDof89	G-box	923	932	cis-acting regulatory element involved in light responsiveness
CsDof89	G-box	925	931	cis-acting regulatory element involved in light responsiveness
CsDof89	G-box	1120	1129	cis-acting regulatory element involved in light responsiveness
CsDof89	G-box	1122	1128	cis-acting regulatory element involved in light responsiveness
CsDof89	G-box	1211	1217	cis-acting regulatory element involved in light responsiveness
CsDof89	G-box	1231	1237	cis-acting regulatory element involved in light responsiveness
CsDof89	G-box	1280	1289	cis-acting regulatory element involved in light responsiveness
CsDof89	G-box	1282	1288	cis-acting regulatory element involved in light responsiveness
CsDof89	G-Box	925	931	cis-acting regulatory element involved in light responsiveness
CsDof89	G-Box	1122	1128	cis-acting regulatory element involved in light responsiveness
CsDof89	G-Box	1211	1217	cis-acting regulatory element involved in light responsiveness
CsDof89	G-Box	1282	1288	cis-acting regulatory element involved in light responsiveness
CsDof89	RY-element	1146	1154	cis-acting regulatory element involved in seed-specific regulation
CsDof89	CGTCA-motif	592	597	cis-acting regulatory element involved in the MeJA-responsiveness
CsDof89	CGTCA-motif	607	612	cis-acting regulatory element involved in the MeJA-responsiveness
CsDof89	CGTCA-motif	1152	1157	cis-acting regulatory element involved in the MeJA-responsiveness
CsDof89	CGTCA-motif	1230	1235	cis-acting regulatory element involved in the MeJA-responsiveness
CsDof89	CGTCA-motif	1558	1563	cis-acting regulatory element involved in the MeJA-responsiveness
CsDof89	TGACG-motif	592	597	cis-acting regulatory element involved in the MeJA-responsiveness
CsDof89	TGACG-motif	607	612	cis-acting regulatory element involved in the MeJA-responsiveness
CsDof89	TGACG-motif	1152	1157	cis-acting regulatory element involved in the MeJA-responsiveness
CsDof89	TGACG-motif	1230	1235	cis-acting regulatory element involved in the MeJA-responsiveness

Name	Cis-element	Start position	Stop position	Function
CsDof89	TGACG-motif	1558	1563	cis-acting regulatory element involved in the MeJA-responsiveness
CsDof89	CAT-box	1893	1899	cis-acting regulatory element related to meristem expression
CsDof89	CAAT-box	3	8	common cis-acting element in promoter and enhancer regions
CsDof89	CAAT-box	188	193	common cis-acting element in promoter and enhancer regions
CsDof89	CAAT-box	210	215	common cis-acting element in promoter and enhancer regions
CsDof89	CAAT-box	282	287	common cis-acting element in promoter and enhancer regions
CsDof89	CAAT-box	299	304	common cis-acting element in promoter and enhancer regions
CsDof89	CAAT-box	336	341	common cis-acting element in promoter and enhancer regions
CsDof89	CAAT-box	377	382	common cis-acting element in promoter and enhancer regions
CsDof89	CAAT-box	387	392	common cis-acting element in promoter and enhancer regions
CsDof89	CAAT-box	423	428	common cis-acting element in promoter and enhancer regions
CsDof89	CAAT-box	428	433	common cis-acting element in promoter and enhancer regions
CsDof89	CAAT-box	526	531	common cis-acting element in promoter and enhancer regions
CsDof89	CAAT-box	814	819	common cis-acting element in promoter and enhancer regions
CsDof89	CAAT-box	1055	1060	common cis-acting element in promoter and enhancer regions
CsDof89	CAAT-box	1138	1143	common cis-acting element in promoter and enhancer regions
CsDof89	CAAT-box	1203	1208	common cis-acting element in promoter and enhancer regions
CsDof89	CAAT-box	1590	1595	common cis-acting element in promoter and enhancer regions
CsDof89	CAAT-box	1630	1635	common cis-acting element in promoter and enhancer regions
CsDof89	CAAT-box	1939	1946	common cis-acting element in promoter and enhancer regions
CsDof89	TATA-box	19	24	core promoter element around -30 of transcription start
CsDof89	TATA-box	20	24	core promoter element around -30 of transcription start
CsDof89	TATA-box	138	147	core promoter element around -30 of transcription start
CsDof89	TATA-box	139	146	core promoter element around -30 of transcription start
CsDof89	TATA-box	140	146	core promoter element around -30 of transcription start
CsDof89	TATA-box	141	146	core promoter element around -30 of transcription start
CsDof89	TATA-box	142	146	core promoter element around -30 of transcription start
CsDof89	TATA-box	287	292	core promoter element around -30 of transcription start
CsDof89	TATA-box	288	292	core promoter element around -30 of transcription start
CsDof89	TATA-box	361	365	core promoter element around -30 of transcription start
CsDof89	TATA-box	543	547	core promoter element around -30 of transcription start
CsDof89	TATA-box	567	571	core promoter element around -30 of transcription start
CsDof89	TATA-box	616	623	core promoter element around -30 of transcription start
CsDof89	TATA-box	617	623	core promoter element around -30 of transcription start
CsDof89	TATA-box	618	623	core promoter element around -30 of transcription start
CsDof89	TATA-box	619	623	core promoter element around -30 of transcription start
CsDof89	TATA-box	659	665	core promoter element around -30 of transcription start
CsDof89	TATA-box	661	665	core promoter element around -30 of transcription start
CsDof89	TATA-box	764	768	core promoter element around -30 of transcription start
CsDof89	TATA-box	791	797	core promoter element around -30 of transcription start
CsDof89	TATA-box	792	797	core promoter element around -30 of transcription start
CsDof89	TATA-box	793	797	core promoter element around -30 of transcription start
CsDof89	TATA-box	820	825	core promoter element around -30 of transcription start
CsDof89	TATA-box	821	825	core promoter element around -30 of transcription start
CsDof89	TATA-box	1076	1082	core promoter element around -30 of transcription start
CsDof89	TATA-box	1077	1083	core promoter element around -30 of transcription start
CsDof89	TATA-box	1078	1082	core promoter element around -30 of transcription start
CsDof89	TATA-box	1101	1107	core promoter element around -30 of transcription start
CsDof89	TATA-box	1102	1106	core promoter element around -30 of transcription start
CsDof89	TATA-box	1243	1251	core promoter element around -30 of transcription start
CsDof89	TATA-box	1546	1554	core promoter element around -30 of transcription start

Name	Cis-element	Start position	Stop position	Function
CsDof89	TATA-box	1764	1772	core promoter element around -30 of transcription start
CsDof89	TATA-box	1766	1775	core promoter element around -30 of transcription start
CsDof89	TATA-box	1767	1772	core promoter element around -30 of transcription start
CsDof89	TATA-box	1768	1772	core promoter element around -30 of transcription start
CsDof89	TATA-box	1780	1787	core promoter element around -30 of transcription start
CsDof89	TATA-box	1841	1846	core promoter element around -30 of transcription start
CsDof89	TATA-box	1842	1846	core promoter element around -30 of transcription start
CsDof89	AT-rich sequence	256	265	element for maximal elicitor-mediated activation (2copies)
CsDof89	HD-Zip 1	243	251	element involved in differentiation of the palisade mesophyll cells
CsDof89	CCAAT-box	1515	1521	MYBHv1 binding site
CsDof90	ABRE	1055	1064	abscisic acid responsiveness
CsDof90	ABRE	1057	1062	abscisic acid responsiveness
CsDof90	ABRE	1354	1359	abscisic acid responsiveness
CsDof90	LTR	1714	1720	cis-acting element involved in low-temperature responsiveness
CsDof90	TCA-element	990	1000	cis-acting element involved in salicylic acid responsiveness
CsDof90	ARE	654	660	cis-acting regulatory element essential for the anaerobic induction
CsDof90	ARE	1273	1279	cis-acting regulatory element essential for the anaerobic induction
CsDof90	ARE	1327	1333	cis-acting regulatory element essential for the anaerobic induction
CsDof90	G-box	1057	1063	cis-acting regulatory element involved in light responsiveness
CsDof90	G-box	1354	1360	cis-acting regulatory element involved in light responsiveness
CsDof90	CAAT-box	825	830	common cis-acting element in promoter and enhancer regions
CsDof90	CAAT-box	837	842	common cis-acting element in promoter and enhancer regions
CsDof90	CAAT-box	1022	1027	common cis-acting element in promoter and enhancer regions
CsDof90	CAAT-box	1153	1158	common cis-acting element in promoter and enhancer regions
CsDof90	CAAT-box	1266	1271	common cis-acting element in promoter and enhancer regions
CsDof90	CAAT-box	1777	1782	common cis-acting element in promoter and enhancer regions
CsDof90	CAAT-box	1908	1913	common cis-acting element in promoter and enhancer regions
CsDof90	TATA-box	737	743	core promoter element around -30 of transcription start
CsDof90	TATA-box	738	745	core promoter element around -30 of transcription start
CsDof90	TATA-box	739	745	core promoter element around -30 of transcription start
CsDof90	TATA-box	740	746	core promoter element around -30 of transcription start
CsDof90	TATA-box	741	745	core promoter element around -30 of transcription start
CsDof90	TATA-box	889	893	core promoter element around -30 of transcription start
CsDof90	TATA-box	926	932	core promoter element around -30 of transcription start
CsDof90	TATA-box	927	932	core promoter element around -30 of transcription start
CsDof90	TATA-box	928	932	core promoter element around -30 of transcription start
CsDof90	TATA-box	980	984	core promoter element around -30 of transcription start
CsDof90	TATA-box	1017	1023	core promoter element around -30 of transcription start
CsDof90	TATA-box	1018	1023	core promoter element around -30 of transcription start
CsDof90	TATA-box	1019	1023	core promoter element around -30 of transcription start
CsDof90	TATA-box	1047	1051	core promoter element around -30 of transcription start
CsDof90	TATA-box	1128	1134	core promoter element around -30 of transcription start
CsDof90	TATA-box	1129	1134	core promoter element around -30 of transcription start
CsDof90	TATA-box	1130	1134	core promoter element around -30 of transcription start
CsDof90	TATA-box	1175	1180	core promoter element around -30 of transcription start
CsDof90	TATA-box	1176	1180	core promoter element around -30 of transcription start
CsDof90	TATA-box	1193	1201	core promoter element around -30 of transcription start
CsDof90	TATA-box	1252	1256	core promoter element around -30 of transcription start
CsDof90	TATA-box	1274	1283	core promoter element around -30 of transcription start
CsDof90	TATA-box	1276	1282	core promoter element around -30 of transcription start
CsDof90	TATA-box	1277	1284	core promoter element around -30 of transcription start

Name	Cis-element	Start position	Stop position	Function
CsDof90	TATA-box	1278	1284	core promoter element around -30 of transcription start
CsDof90	TATA-box	1279	1285	core promoter element around -30 of transcription start
CsDof90	TATA-box	1280	1286	core promoter element around -30 of transcription start
CsDof90	TATA-box	1281	1287	core promoter element around -30 of transcription start
CsDof90	TATA-box	1282	1288	core promoter element around -30 of transcription start
CsDof90	TATA-box	1283	1289	core promoter element around -30 of transcription start
CsDof90	TATA-box	1284	1290	core promoter element around -30 of transcription start
CsDof90	TATA-box	1285	1291	core promoter element around -30 of transcription start
CsDof90	TATA-box	1286	1292	core promoter element around -30 of transcription start
CsDof90	TATA-box	1287	1293	core promoter element around -30 of transcription start
CsDof90	TATA-box	1288	1292	core promoter element around -30 of transcription start
CsDof90	TATA-box	1406	1412	core promoter element around -30 of transcription start
CsDof90	TATA-box	1407	1412	core promoter element around -30 of transcription start
CsDof90	TATA-box	1408	1412	core promoter element around -30 of transcription start
CsDof90	TATA-box	1436	1441	core promoter element around -30 of transcription start
CsDof90	TATA-box	1437	1441	core promoter element around -30 of transcription start
CsDof90	TATA-box	1455	1462	core promoter element around -30 of transcription start
CsDof90	TATA-box	1461	1467	core promoter element around -30 of transcription start
CsDof90	TATA-box	1462	1468	core promoter element around -30 of transcription start
CsDof90	TATA-box	1463	1469	core promoter element around -30 of transcription start
CsDof90	TATA-box	1464	1468	core promoter element around -30 of transcription start
CsDof90	TATA-box	1495	1500	core promoter element around -30 of transcription start
CsDof90	TATA-box	1496	1500	core promoter element around -30 of transcription start
CsDof90	TATA-box	1506	1512	core promoter element around -30 of transcription start
CsDof90	TATA-box	1508	1512	core promoter element around -30 of transcription start
CsDof90	TATA-box	1532	1538	core promoter element around -30 of transcription start
CsDof90	TATA-box	1533	1539	core promoter element around -30 of transcription start
CsDof90	TATA-box	1534	1540	core promoter element around -30 of transcription start
CsDof90	TATA-box	1535	1541	core promoter element around -30 of transcription start
CsDof90	TATA-box	1536	1540	core promoter element around -30 of transcription start
CsDof90	TATA-box	1569	1574	core promoter element around -30 of transcription start
CsDof90	TATA-box	1570	1574	core promoter element around -30 of transcription start
CsDof90	TATA-box	1644	1648	core promoter element around -30 of transcription start
CsDof90	TATA-box	1656	1660	core promoter element around -30 of transcription start
CsDof90	TATA-box	1682	1688	core promoter element around -30 of transcription start
CsDof90	TATA-box	1683	1688	core promoter element around -30 of transcription start
CsDof90	TATA-box	1684	1688	core promoter element around -30 of transcription start
CsDof90	TATA-box	1691	1697	core promoter element around -30 of transcription start
CsDof90	TATA-box	1692	1698	core promoter element around -30 of transcription start
CsDof90	TATA-box	1694	1698	core promoter element around -30 of transcription start
CsDof90	3-AF1 binding site	1556	1566	light responsive element
CsDof90	3-AF1 binding site	1858	1868	light responsive element
CsDof91	ABRE	710	716	abscisic acid responsiveness
CsDof91	ABRE	711	716	abscisic acid responsiveness
CsDof91	ABRE	1805	1810	abscisic acid responsiveness
CsDof91	TC-rich repeats	950	959	cis-acting element involved in defense and stress responsiveness
CsDof91	LTR	376	382	cis-acting element involved in low-temperature responsiveness
CsDof91	TCA-element	1309	1318	cis-acting element involved in salicylic acid responsiveness
CsDof91	ARE	861	867	cis-acting regulatory element essential for the anaerobic induction
CsDof91	ARE	1482	1488	cis-acting regulatory element essential for the anaerobic induction
CsDof91	G-box	84	93	cis-acting regulatory element involved in light responsiveness

Name	Cis-element	Start position	Stop position	Function
CsDof91	G-box	710	716	cis-acting regulatory element involved in light responsiveness
CsDof91	G-box	1517	1523	cis-acting regulatory element involved in light responsiveness
CsDof91	G-box	1805	1813	cis-acting regulatory element involved in light responsiveness
CsDof91	G-Box	710	716	cis-acting regulatory element involved in light responsiveness
CsDof91	G-Box	1805	1811	cis-acting regulatory element involved in light responsiveness
CsDof91	TGACG-motif	1123	1128	cis-acting regulatory element involved in the MeJA-responsiveness
CsDof91	CGTCA-motif	1123	1128	cis-acting regulatory element involved in the MeJA-responsiveness
CsDof91	CAAT-box	32	37	common cis-acting element in promoter and enhancer regions
CsDof91	CAAT-box	172	177	common cis-acting element in promoter and enhancer regions
CsDof91	CAAT-box	371	376	common cis-acting element in promoter and enhancer regions
CsDof91	CAAT-box	480	485	common cis-acting element in promoter and enhancer regions
CsDof91	CAAT-box	521	526	common cis-acting element in promoter and enhancer regions
CsDof91	CAAT-box	617	622	common cis-acting element in promoter and enhancer regions
CsDof91	CAAT-box	817	822	common cis-acting element in promoter and enhancer regions
CsDof91	CAAT-box	835	840	common cis-acting element in promoter and enhancer regions
CsDof91	CAAT-box	1413	1418	common cis-acting element in promoter and enhancer regions
CsDof91	CAAT-box	1577	1582	common cis-acting element in promoter and enhancer regions
CsDof91	CAAT-box	1646	1651	common cis-acting element in promoter and enhancer regions
CsDof91	CAAT-box	1742	1747	common cis-acting element in promoter and enhancer regions
CsDof91	CAAT-box	1956	1961	common cis-acting element in promoter and enhancer regions
CsDof91	TATA-box	266	273	core promoter element around -30 of transcription start
CsDof91	TATA-box	267	273	core promoter element around -30 of transcription start
CsDof91	TATA-box	268	274	core promoter element around -30 of transcription start
CsDof91	TATA-box	269	275	core promoter element around -30 of transcription start
CsDof91	TATA-box	271	275	core promoter element around -30 of transcription start
CsDof91	TATA-box	285	291	core promoter element around -30 of transcription start
CsDof91	TATA-box	286	291	core promoter element around -30 of transcription start
CsDof91	TATA-box	287	291	core promoter element around -30 of transcription start
CsDof91	TATA-box	300	306	core promoter element around -30 of transcription start
CsDof91	TATA-box	301	307	core promoter element around -30 of transcription start
CsDof91	TATA-box	302	308	core promoter element around -30 of transcription start
CsDof91	TATA-box	303	307	core promoter element around -30 of transcription start
CsDof91	TATA-box	421	430	core promoter element around -30 of transcription start
CsDof91	TATA-box	422	429	core promoter element around -30 of transcription start
CsDof91	TATA-box	423	429	core promoter element around -30 of transcription start
CsDof91	TATA-box	424	429	core promoter element around -30 of transcription start
CsDof91	TATA-box	425	429	core promoter element around -30 of transcription start
CsDof91	TATA-box	573	578	core promoter element around -30 of transcription start
CsDof91	TATA-box	574	578	core promoter element around -30 of transcription start
CsDof91	TATA-box	602	608	core promoter element around -30 of transcription start
CsDof91	TATA-box	603	609	core promoter element around -30 of transcription start
CsDof91	TATA-box	604	608	core promoter element around -30 of transcription start
CsDof91	TATA-box	768	772	core promoter element around -30 of transcription start
CsDof91	TATA-box	781	788	core promoter element around -30 of transcription start
CsDof91	TATA-box	782	788	core promoter element around -30 of transcription start
CsDof91	TATA-box	783	789	core promoter element around -30 of transcription start
CsDof91	TATA-box	784	788	core promoter element around -30 of transcription start
CsDof91	TATA-box	870	874	core promoter element around -30 of transcription start
CsDof91	TATA-box	937	941	core promoter element around -30 of transcription start
CsDof91	TATA-box	943	950	core promoter element around -30 of transcription start
CsDof91	TATA-box	972	979	core promoter element around -30 of transcription start

Name	Cis-element	Start position	Stop position	Function
CsDof91	TATA-box	973	979	core promoter element around -30 of transcription start
CsDof91	TATA-box	974	979	core promoter element around -30 of transcription start
CsDof91	TATA-box	975	979	core promoter element around -30 of transcription start
CsDof91	TATA-box	994	998	core promoter element around -30 of transcription start
CsDof91	TATA-box	1014	1020	core promoter element around -30 of transcription start
CsDof91	TATA-box	1015	1021	core promoter element around -30 of transcription start
CsDof91	TATA-box	1016	1022	core promoter element around -30 of transcription start
CsDof91	TATA-box	1017	1023	core promoter element around -30 of transcription start
CsDof91	TATA-box	1018	1024	core promoter element around -30 of transcription start
CsDof91	TATA-box	1019	1025	core promoter element around -30 of transcription start
CsDof91	TATA-box	1020	1026	core promoter element around -30 of transcription start
CsDof91	TATA-box	1021	1027	core promoter element around -30 of transcription start
CsDof91	TATA-box	1022	1028	core promoter element around -30 of transcription start
CsDof91	TATA-box	1023	1029	core promoter element around -30 of transcription start
CsDof91	TATA-box	1024	1030	core promoter element around -30 of transcription start
CsDof91	TATA-box	1025	1031	core promoter element around -30 of transcription start
CsDof91	TATA-box	1026	1032	core promoter element around -30 of transcription start
CsDof91	TATA-box	1027	1033	core promoter element around -30 of transcription start
CsDof91	TATA-box	1028	1034	core promoter element around -30 of transcription start
CsDof91	TATA-box	1029	1035	core promoter element around -30 of transcription start
CsDof91	TATA-box	1030	1036	core promoter element around -30 of transcription start
CsDof91	TATA-box	1031	1037	core promoter element around -30 of transcription start
CsDof91	TATA-box	1032	1038	core promoter element around -30 of transcription start
CsDof91	TATA-box	1033	1039	core promoter element around -30 of transcription start
CsDof91	TATA-box	1034	1040	core promoter element around -30 of transcription start
CsDof91	TATA-box	1035	1041	core promoter element around -30 of transcription start
CsDof91	TATA-box	1036	1042	core promoter element around -30 of transcription start
CsDof91	TATA-box	1037	1043	core promoter element around -30 of transcription start
CsDof91	TATA-box	1038	1044	core promoter element around -30 of transcription start
CsDof91	TATA-box	1039	1045	core promoter element around -30 of transcription start
CsDof91	TATA-box	1040	1046	core promoter element around -30 of transcription start
CsDof91	TATA-box	1041	1047	core promoter element around -30 of transcription start
CsDof91	TATA-box	1042	1048	core promoter element around -30 of transcription start
CsDof91	TATA-box	1043	1049	core promoter element around -30 of transcription start
CsDof91	TATA-box	1045	1049	core promoter element around -30 of transcription start
CsDof91	TATA-box	1061	1067	core promoter element around -30 of transcription start
CsDof91	TATA-box	1062	1068	core promoter element around -30 of transcription start
CsDof91	TATA-box	1063	1069	core promoter element around -30 of transcription start
CsDof91	TATA-box	1064	1070	core promoter element around -30 of transcription start
CsDof91	TATA-box	1065	1069	core promoter element around -30 of transcription start
CsDof91	TATA-box	1280	1286	core promoter element around -30 of transcription start
CsDof91	TATA-box	1281	1285	core promoter element around -30 of transcription start
CsDof91	TATA-box	1322	1328	core promoter element around -30 of transcription start
CsDof91	TATA-box	1323	1330	core promoter element around -30 of transcription start
CsDof91	TATA-box	1324	1330	core promoter element around -30 of transcription start
CsDof91	TATA-box	1326	1330	core promoter element around -30 of transcription start
CsDof91	TATA-box	1498	1506	core promoter element around -30 of transcription start
CsDof91	TATA-box	1556	1560	core promoter element around -30 of transcription start
CsDof91	TATA-box	1710	1716	core promoter element around -30 of transcription start
CsDof91	TATA-box	1711	1718	core promoter element around -30 of transcription start
CsDof91	TATA-box	1712	1718	core promoter element around -30 of transcription start

Name	Cis-element	Start position	Stop position	Function
CsDof91	TATA-box	1713	1719	core promoter element around -30 of transcription start
CsDof91	TATA-box	1714	1720	core promoter element around -30 of transcription start
CsDof91	TATA-box	1716	1720	core promoter element around -30 of transcription start
CsDof91	TATA-box	1897	1903	core promoter element around -30 of transcription start
CsDof91	TATA-box	1898	1904	core promoter element around -30 of transcription start
CsDof91	TATA-box	1899	1905	core promoter element around -30 of transcription start
CsDof91	TATA-box	1900	1904	core promoter element around -30 of transcription start
CsDof91	TATA-box	1925	1929	core promoter element around -30 of transcription start
CsDof91	TATA-box	1958	1966	core promoter element around -30 of transcription start
CsDof91	TATA-box	1991	1995	core promoter element around -30 of transcription start
CsDof91	Sp1	1800	1806	light responsive element
CsDof91	MRE	101	108	MYB binding site involved in light responsiveness
CsDof92	ABRE	1829	1834	abscisic acid responsiveness
CsDof92	TCA-element	1899	1908	cis-acting element involved in salicylic acid responsiveness
CsDof92	G-box	1828	1834	cis-acting regulatory element involved in light responsiveness
CsDof92	CAAT-box	1931	1936	common cis-acting element in promoter and enhancer regions
CsDof92	CAAT-box	1958	1963	common cis-acting element in promoter and enhancer regions
CsDof92	TATA-box	1903	1911	core promoter element around -30 of transcription start
CsDof92	TATA-box	1906	1911	core promoter element around -30 of transcription start
CsDof92	TATA-box	1907	1911	core promoter element around -30 of transcription start
CsDof93	ABRE	1209	1214	abscisic acid responsiveness
CsDof93	ACE	1770	1779	cis-acting element involved in light responsiveness
CsDof93	LTR	214	220	cis-acting element involved in low-temperature responsiveness
CsDof93	LTR	301	307	cis-acting element involved in low-temperature responsiveness
CsDof93	LTR	383	389	cis-acting element involved in low-temperature responsiveness
CsDof93	LTR	392	398	cis-acting element involved in low-temperature responsiveness
CsDof93	TCA-element	673	682	cis-acting element involved in salicylic acid responsiveness
CsDof93	TCA-element	1886	1895	cis-acting element involved in salicylic acid responsiveness
CsDof93	ARE	1259	1265	cis-acting regulatory element essential for the anaerobic induction
CsDof93	ARE	1548	1554	cis-acting regulatory element essential for the anaerobic induction
CsDof93	G-Box	1209	1215	cis-acting regulatory element involved in light responsiveness
CsDof93	O2-site	646	655	cis-acting regulatory element involved in zein metabolism regulation
CsDof93	O2-site	678	687	cis-acting regulatory element involved in zein metabolism regulation
CsDof93	CAAT-box	75	80	common cis-acting element in promoter and enhancer regions
CsDof93	CAAT-box	446	451	common cis-acting element in promoter and enhancer regions
CsDof93	CAAT-box	795	800	common cis-acting element in promoter and enhancer regions
CsDof93	CAAT-box	1015	1020	common cis-acting element in promoter and enhancer regions
CsDof93	CAAT-box	1141	1146	common cis-acting element in promoter and enhancer regions
CsDof93	CAAT-box	1167	1172	common cis-acting element in promoter and enhancer regions
CsDof93	CAAT-box	1204	1209	common cis-acting element in promoter and enhancer regions
CsDof93	CAAT-box	1552	1557	common cis-acting element in promoter and enhancer regions
CsDof93	CAAT-box	1871	1876	common cis-acting element in promoter and enhancer regions
CsDof93	TATA-box	112	119	core promoter element around -30 of transcription start
CsDof93	TATA-box	219	225	core promoter element around -30 of transcription start
CsDof93	TATA-box	220	224	core promoter element around -30 of transcription start
CsDof93	TATA-box	415	421	core promoter element around -30 of transcription start
CsDof93	TATA-box	416	421	core promoter element around -30 of transcription start
CsDof93	TATA-box	417	421	core promoter element around -30 of transcription start
CsDof93	TATA-box	541	546	core promoter element around -30 of transcription start
CsDof93	TATA-box	542	546	core promoter element around -30 of transcription start
CsDof93	TATA-box	596	600	core promoter element around -30 of transcription start

Name	Cis-element	Start position	Stop position	Function
CsDof93	TATA-box	857	862	core promoter element around -30 of transcription start
CsDof93	TATA-box	858	862	core promoter element around -30 of transcription start
CsDof93	TATA-box	878	884	core promoter element around -30 of transcription start
CsDof93	TATA-box	879	883	core promoter element around -30 of transcription start
CsDof93	TATA-box	1020	1026	core promoter element around -30 of transcription start
CsDof93	TATA-box	1021	1028	core promoter element around -30 of transcription start
CsDof93	TATA-box	1022	1028	core promoter element around -30 of transcription start
CsDof93	TATA-box	1024	1028	core promoter element around -30 of transcription start
CsDof93	TATA-box	1032	1038	core promoter element around -30 of transcription start
CsDof93	TATA-box	1033	1037	core promoter element around -30 of transcription start
CsDof93	TATA-box	1102	1106	core promoter element around -30 of transcription start
CsDof93	TATA-box	1109	1113	core promoter element around -30 of transcription start
CsDof93	TATA-box	1231	1240	core promoter element around -30 of transcription start
CsDof93	TATA-box	1233	1241	core promoter element around -30 of transcription start
CsDof93	TATA-box	1238	1244	core promoter element around -30 of transcription start
CsDof93	TATA-box	1239	1243	core promoter element around -30 of transcription start
CsDof93	TATA-box	1286	1293	core promoter element around -30 of transcription start
CsDof93	TATA-box	1287	1293	core promoter element around -30 of transcription start
CsDof93	TATA-box	1288	1294	core promoter element around -30 of transcription start
CsDof93	TATA-box	1289	1293	core promoter element around -30 of transcription start
CsDof93	TATA-box	1323	1329	core promoter element around -30 of transcription start
CsDof93	TATA-box	1324	1329	core promoter element around -30 of transcription start
CsDof93	TATA-box	1325	1329	core promoter element around -30 of transcription start
CsDof93	TATA-box	1330	1334	core promoter element around -30 of transcription start
CsDof93	TATA-box	1358	1365	core promoter element around -30 of transcription start
CsDof93	TATA-box	1555	1561	core promoter element around -30 of transcription start
CsDof93	TATA-box	1556	1560	core promoter element around -30 of transcription start
CsDof93	TATA-box	1615	1622	core promoter element around -30 of transcription start
CsDof93	TATA-box	1616	1622	core promoter element around -30 of transcription start
CsDof93	TATA-box	1617	1622	core promoter element around -30 of transcription start
CsDof93	TATA-box	1618	1622	core promoter element around -30 of transcription start
CsDof93	TATA-box	1625	1629	core promoter element around -30 of transcription start
CsDof93	TATA-box	1675	1681	core promoter element around -30 of transcription start
CsDof93	TATA-box	1676	1681	core promoter element around -30 of transcription start
CsDof93	TATA-box	1677	1681	core promoter element around -30 of transcription start
CsDof93	TATA-box	1724	1730	core promoter element around -30 of transcription start
CsDof93	TATA-box	1725	1729	core promoter element around -30 of transcription start
CsDof93	TATA-box	1792	1799	core promoter element around -30 of transcription start
CsDof93	TATA-box	1793	1799	core promoter element around -30 of transcription start
CsDof93	TATA-box	1794	1799	core promoter element around -30 of transcription start
CsDof93	TATA-box	1795	1799	core promoter element around -30 of transcription start
CsDof93	TATA-box	1846	1852	core promoter element around -30 of transcription start
CsDof93	TATA-box	1847	1851	core promoter element around -30 of transcription start
CsDof93	TATA-box	1882	1889	core promoter element around -30 of transcription start
CsDof93	TATA-box	1906	1915	core promoter element around -30 of transcription start
CsDof93	TATA-box	1908	1912	core promoter element around -30 of transcription start
CsDof93	GT1-motif	97	103	light responsive element
CsDof93	GT1-motif	291	297	light responsive element
CsDof93	AAAC-motif	1125	1136	light responsive element
CsDof93	MRE	1123	1130	MYB binding site involved in light responsiveness
CsDof94	ABRE	643	648	abscisic acid responsiveness

Name	Cis-element	Start position	Stop position	Function
CsDof94	TGA-element	1625	1631	auxin-responsive element
CsDof94	AT-rich element	890	900	binding site of AT-rich DNA binding protein (ATBP-1)
CsDof94	ARE	451	457	cis-acting regulatory element essential for the anaerobic induction
CsDof94	ARE	1283	1289	cis-acting regulatory element essential for the anaerobic induction
CsDof94	ARE	1893	1899	cis-acting regulatory element essential for the anaerobic induction
CsDof94	circadian	892	901	cis-acting regulatory element involved in circadian control
CsDof94	G-box	639	645	cis-acting regulatory element involved in light responsiveness
CsDof94	G-box	642	648	cis-acting regulatory element involved in light responsiveness
CsDof94	TGACG-motif	565	570	cis-acting regulatory element involved in the MeJA-responsiveness
CsDof94	CGTCA-motif	565	570	cis-acting regulatory element involved in the MeJA-responsiveness
CsDof94	CAT-box	499	505	cis-acting regulatory element related to meristem expression
CsDof94	CAT-box	1220	1226	cis-acting regulatory element related to meristem expression
CsDof94	CAAT-box	40	45	common cis-acting element in promoter and enhancer regions
CsDof94	CAAT-box	107	112	common cis-acting element in promoter and enhancer regions
CsDof94	CAAT-box	283	288	common cis-acting element in promoter and enhancer regions
CsDof94	CAAT-box	290	295	common cis-acting element in promoter and enhancer regions
CsDof94	CAAT-box	293	298	common cis-acting element in promoter and enhancer regions
CsDof94	CAAT-box	446	451	common cis-acting element in promoter and enhancer regions
CsDof94	CAAT-box	482	487	common cis-acting element in promoter and enhancer regions
CsDof94	CAAT-box	593	598	common cis-acting element in promoter and enhancer regions
CsDof94	CAAT-box	736	741	common cis-acting element in promoter and enhancer regions
CsDof94	CAAT-box	749	754	common cis-acting element in promoter and enhancer regions
CsDof94	CAAT-box	888	893	common cis-acting element in promoter and enhancer regions
CsDof94	CAAT-box	921	926	common cis-acting element in promoter and enhancer regions
CsDof94	CAAT-box	974	979	common cis-acting element in promoter and enhancer regions
CsDof94	CAAT-box	1016	1021	common cis-acting element in promoter and enhancer regions
CsDof94	CAAT-box	1117	1122	common cis-acting element in promoter and enhancer regions
CsDof94	CAAT-box	1176	1181	common cis-acting element in promoter and enhancer regions
CsDof94	CAAT-box	1430	1435	common cis-acting element in promoter and enhancer regions
CsDof94	CAAT-box	1585	1590	common cis-acting element in promoter and enhancer regions
CsDof94	CAAT-box	1616	1621	common cis-acting element in promoter and enhancer regions
CsDof94	CAAT-box	1632	1637	common cis-acting element in promoter and enhancer regions
CsDof94	CAAT-box	1707	1712	common cis-acting element in promoter and enhancer regions
CsDof94	CAAT-box	1719	1724	common cis-acting element in promoter and enhancer regions
CsDof94	CAAT-box	1784	1789	common cis-acting element in promoter and enhancer regions
CsDof94	CAAT-box	1830	1835	common cis-acting element in promoter and enhancer regions
CsDof94	TATA-box	128	132	core promoter element around -30 of transcription start
CsDof94	TATA-box	163	168	core promoter element around -30 of transcription start
CsDof94	TATA-box	164	168	core promoter element around -30 of transcription start
CsDof94	TATA-box	278	282	core promoter element around -30 of transcription start
CsDof94	TATA-box	382	386	core promoter element around -30 of transcription start
CsDof94	TATA-box	758	762	core promoter element around -30 of transcription start
CsDof94	TATA-box	775	781	core promoter element around -30 of transcription start
CsDof94	TATA-box	776	783	core promoter element around -30 of transcription start
CsDof94	TATA-box	777	783	core promoter element around -30 of transcription start
CsDof94	TATA-box	778	784	core promoter element around -30 of transcription start
CsDof94	TATA-box	779	783	core promoter element around -30 of transcription start
CsDof94	TATA-box	804	808	core promoter element around -30 of transcription start
CsDof94	TATA-box	823	827	core promoter element around -30 of transcription start
CsDof94	TATA-box	832	838	core promoter element around -30 of transcription start
CsDof94	TATA-box	833	840	core promoter element around -30 of transcription start

Name	Cis-element	Start position	Stop position	Function
CsDof94	TATA-box	834	840	core promoter element around -30 of transcription start
CsDof94	TATA-box	835	841	core promoter element around -30 of transcription start
CsDof94	TATA-box	836	840	core promoter element around -30 of transcription start
CsDof94	TATA-box	864	870	core promoter element around -30 of transcription start
CsDof94	TATA-box	865	870	core promoter element around -30 of transcription start
CsDof94	TATA-box	866	870	core promoter element around -30 of transcription start
CsDof94	TATA-box	875	881	core promoter element around -30 of transcription start
CsDof94	TATA-box	876	881	core promoter element around -30 of transcription start
CsDof94	TATA-box	877	881	core promoter element around -30 of transcription start
CsDof94	TATA-box	937	943	core promoter element around -30 of transcription start
CsDof94	TATA-box	938	943	core promoter element around -30 of transcription start
CsDof94	TATA-box	939	943	core promoter element around -30 of transcription start
CsDof94	TATA-box	979	985	core promoter element around -30 of transcription start
CsDof94	TATA-box	980	984	core promoter element around -30 of transcription start
CsDof94	TATA-box	999	1005	core promoter element around -30 of transcription start
CsDof94	TATA-box	1000	1005	core promoter element around -30 of transcription start
CsDof94	TATA-box	1001	1005	core promoter element around -30 of transcription start
CsDof94	TATA-box	1026	1030	core promoter element around -30 of transcription start
CsDof94	TATA-box	1036	1045	core promoter element around -30 of transcription start
CsDof94	TATA-box	1038	1042	core promoter element around -30 of transcription start
CsDof94	TATA-box	1078	1083	core promoter element around -30 of transcription start
CsDof94	TATA-box	1079	1083	core promoter element around -30 of transcription start
CsDof94	TATA-box	1146	1154	core promoter element around -30 of transcription start
CsDof94	TATA-box	1337	1344	core promoter element around -30 of transcription start
CsDof94	TATA-box	1338	1344	core promoter element around -30 of transcription start
CsDof94	TATA-box	1339	1344	core promoter element around -30 of transcription start
CsDof94	TATA-box	1340	1344	core promoter element around -30 of transcription start
CsDof94	TATA-box	1344	1353	core promoter element around -30 of transcription start
CsDof94	TATA-box	1345	1352	core promoter element around -30 of transcription start
CsDof94	TATA-box	1346	1352	core promoter element around -30 of transcription start
CsDof94	TATA-box	1347	1354	core promoter element around -30 of transcription start
CsDof94	TATA-box	1348	1354	core promoter element around -30 of transcription start
CsDof94	TATA-box	1349	1355	core promoter element around -30 of transcription start
CsDof94	TATA-box	1350	1356	core promoter element around -30 of transcription start
CsDof94	TATA-box	1351	1357	core promoter element around -30 of transcription start
CsDof94	TATA-box	1352	1356	core promoter element around -30 of transcription start
CsDof94	TATA-box	1373	1380	core promoter element around -30 of transcription start
CsDof94	TATA-box	1374	1380	core promoter element around -30 of transcription start
CsDof94	TATA-box	1375	1380	core promoter element around -30 of transcription start
CsDof94	TATA-box	1376	1380	core promoter element around -30 of transcription start
CsDof94	TATA-box	1463	1471	core promoter element around -30 of transcription start
CsDof94	TATA-box	1466	1471	core promoter element around -30 of transcription start
CsDof94	TATA-box	1467	1471	core promoter element around -30 of transcription start
CsDof94	TATA-box	1562	1566	core promoter element around -30 of transcription start
CsDof94	TATA-box	1574	1581	core promoter element around -30 of transcription start
CsDof94	TATA-box	1575	1581	core promoter element around -30 of transcription start
CsDof94	TATA-box	1576	1581	core promoter element around -30 of transcription start
CsDof94	TATA-box	1577	1581	core promoter element around -30 of transcription start
CsDof94	TATA-box	1710	1716	core promoter element around -30 of transcription start
CsDof94	TATA-box	1711	1718	core promoter element around -30 of transcription start
CsDof94	TATA-box	1712	1718	core promoter element around -30 of transcription start

Name	Cis-element	Start position	Stop position	Function
CsDof94	TATA-box	1713	1719	core promoter element around -30 of transcription start
CsDof94	TATA-box	1714	1718	core promoter element around -30 of transcription start
CsDof94	TATA-box	1743	1750	core promoter element around -30 of transcription start
CsDof94	TATA-box	1744	1750	core promoter element around -30 of transcription start
CsDof94	TATA-box	1745	1750	core promoter element around -30 of transcription start
CsDof94	TATA-box	1746	1750	core promoter element around -30 of transcription start
CsDof94	TATA-box	1842	1848	core promoter element around -30 of transcription start
CsDof94	TATA-box	1843	1849	core promoter element around -30 of transcription start
CsDof94	TATA-box	1845	1849	core promoter element around -30 of transcription start
CsDof94	TATA-box	1886	1892	core promoter element around -30 of transcription start
CsDof94	TATA-box	1887	1893	core promoter element around -30 of transcription start
CsDof94	TATA-box	1888	1892	core promoter element around -30 of transcription start
CsDof94	TATA-box	1996	2001	core promoter element around -30 of transcription start
CsDof94	TATA-box	1997	2001	core promoter element around -30 of transcription start
CsDof94	GT1-motif	179	185	light responsive element
CsDof94	MBS	545	551	MYB binding site involved in drought-inducibility
CsDof94	MBS	1858	1864	MYB binding site involved in drought-inducibility
CsDof95	ABRE	559	564	abscisic acid responsiveness
CsDof95	LTR	1059	1065	cis-acting element involved in low-temperature responsiveness
CsDof95	TCA-element	678	687	cis-acting element involved in salicylic acid responsiveness
CsDof95	TCA-element	1897	1906	cis-acting element involved in salicylic acid responsiveness
CsDof95	ARE	562	568	cis-acting regulatory element essential for the anaerobic induction
CsDof95	ARE	1706	1712	cis-acting regulatory element essential for the anaerobic induction
CsDof95	G-Box	558	564	cis-acting regulatory element involved in light responsiveness
CsDof95	TGACG-motif	419	424	cis-acting regulatory element involved in the MeJA-responsiveness
CsDof95	TGACG-motif	553	558	cis-acting regulatory element involved in the MeJA-responsiveness
CsDof95	TGACG-motif	629	634	cis-acting regulatory element involved in the MeJA-responsiveness
CsDof95	TGACG-motif	1009	1014	cis-acting regulatory element involved in the MeJA-responsiveness
CsDof95	CGTCA-motif	419	424	cis-acting regulatory element involved in the MeJA-responsiveness
CsDof95	CGTCA-motif	553	558	cis-acting regulatory element involved in the MeJA-responsiveness
CsDof95	CGTCA-motif	629	634	cis-acting regulatory element involved in the MeJA-responsiveness
CsDof95	CGTCA-motif	1009	1014	cis-acting regulatory element involved in the MeJA-responsiveness
CsDof95	GCN4_motif	1018	1025	cis-regulatory element involved in endosperm expression
CsDof95	CAAT-box	24	29	common cis-acting element in promoter and enhancer regions
CsDof95	CAAT-box	224	229	common cis-acting element in promoter and enhancer regions
CsDof95	CAAT-box	259	264	common cis-acting element in promoter and enhancer regions
CsDof95	CAAT-box	279	284	common cis-acting element in promoter and enhancer regions
CsDof95	CAAT-box	473	478	common cis-acting element in promoter and enhancer regions
CsDof95	CAAT-box	494	499	common cis-acting element in promoter and enhancer regions
CsDof95	CAAT-box	849	854	common cis-acting element in promoter and enhancer regions
CsDof95	CAAT-box	857	862	common cis-acting element in promoter and enhancer regions
CsDof95	CAAT-box	892	897	common cis-acting element in promoter and enhancer regions
CsDof95	CAAT-box	939	944	common cis-acting element in promoter and enhancer regions
CsDof95	CAAT-box	1012	1017	common cis-acting element in promoter and enhancer regions
CsDof95	CAAT-box	1015	1020	common cis-acting element in promoter and enhancer regions
CsDof95	CAAT-box	1222	1227	common cis-acting element in promoter and enhancer regions
CsDof95	CAAT-box	1373	1378	common cis-acting element in promoter and enhancer regions
CsDof95	CAAT-box	1884	1889	common cis-acting element in promoter and enhancer regions
CsDof95	TATA-box	79	83	core promoter element around -30 of transcription start
CsDof95	TATA-box	94	100	core promoter element around -30 of transcription start
CsDof95	TATA-box	95	100	core promoter element around -30 of transcription start

Name	Cis-element	Start position	Stop position	Function
CsDof95	TATA-box	96	100	core promoter element around -30 of transcription start
CsDof95	TATA-box	249	255	core promoter element around -30 of transcription start
CsDof95	TATA-box	250	255	core promoter element around -30 of transcription start
CsDof95	TATA-box	251	255	core promoter element around -30 of transcription start
CsDof95	TATA-box	389	395	core promoter element around -30 of transcription start
CsDof95	TATA-box	390	395	core promoter element around -30 of transcription start
CsDof95	TATA-box	391	395	core promoter element around -30 of transcription start
CsDof95	TATA-box	617	624	core promoter element around -30 of transcription start
CsDof95	TATA-box	693	699	core promoter element around -30 of transcription start
CsDof95	TATA-box	694	698	core promoter element around -30 of transcription start
CsDof95	TATA-box	1216	1220	core promoter element around -30 of transcription start
CsDof95	TATA-box	1265	1269	core promoter element around -30 of transcription start
CsDof95	TATA-box	1310	1315	core promoter element around -30 of transcription start
CsDof95	TATA-box	1311	1315	core promoter element around -30 of transcription start
CsDof95	TATA-box	1412	1419	core promoter element around -30 of transcription start
CsDof95	TATA-box	1413	1419	core promoter element around -30 of transcription start
CsDof95	TATA-box	1414	1419	core promoter element around -30 of transcription start
CsDof95	TATA-box	1415	1419	core promoter element around -30 of transcription start
CsDof95	TATA-box	1452	1458	core promoter element around -30 of transcription start
CsDof95	TATA-box	1453	1459	core promoter element around -30 of transcription start
CsDof95	TATA-box	1454	1460	core promoter element around -30 of transcription start
CsDof95	TATA-box	1455	1459	core promoter element around -30 of transcription start
CsDof95	TATA-box	1461	1466	core promoter element around -30 of transcription start
CsDof95	TATA-box	1462	1466	core promoter element around -30 of transcription start
CsDof95	TATA-box	1486	1492	core promoter element around -30 of transcription start
CsDof95	TATA-box	1487	1491	core promoter element around -30 of transcription start
CsDof95	TATA-box	1508	1514	core promoter element around -30 of transcription start
CsDof95	TATA-box	1509	1513	core promoter element around -30 of transcription start
CsDof95	TATA-box	1608	1615	core promoter element around -30 of transcription start
CsDof95	TATA-box	1846	1850	core promoter element around -30 of transcription start
CsDof95	GT1-motif	1172	1178	light responsive element
CsDof95	MRE	1078	1085	MYB binding site involved in light responsiveness
CsDof95	MRE	1250	1257	MYB binding site involved in light responsiveness
CsDof95	WUN-motif	1378	1387	wound-responsive element
CsDof96	ABRE	1701	1706	abscisic acid responsiveness
CsDof96	TGA-element	1154	1160	auxin-responsive element
CsDof96	LTR	1243	1249	cis-acting element involved in low-temperature responsiveness
CsDof96	ARE	807	813	cis-acting regulatory element essential for the anaerobic induction
CsDof96	ARE	1458	1464	cis-acting regulatory element essential for the anaerobic induction
CsDof96	ARE	1713	1719	cis-acting regulatory element essential for the anaerobic induction
CsDof96	AuxRR-core	944	951	cis-acting regulatory element involved in auxin responsiveness
CsDof96	G-Box	1701	1707	cis-acting regulatory element involved in light responsiveness
CsDof96	CAT-box	1218	1224	cis-acting regulatory element related to meristem expression
CsDof96	CAAT-box	814	819	common cis-acting element in promoter and enhancer regions
CsDof96	CAAT-box	829	834	common cis-acting element in promoter and enhancer regions
CsDof96	CAAT-box	1012	1017	common cis-acting element in promoter and enhancer regions
CsDof96	CAAT-box	1126	1131	common cis-acting element in promoter and enhancer regions
CsDof96	CAAT-box	1402	1407	common cis-acting element in promoter and enhancer regions
CsDof96	CAAT-box	1415	1420	common cis-acting element in promoter and enhancer regions
CsDof96	CAAT-box	1486	1491	common cis-acting element in promoter and enhancer regions
CsDof96	CAAT-box	1578	1583	common cis-acting element in promoter and enhancer regions

Name	Cis-element	Start position	Stop position	Function
CsDof96	CAAT-box	1696	1701	common cis-acting element in promoter and enhancer regions
CsDof96	CAAT-box	1711	1716	common cis-acting element in promoter and enhancer regions
CsDof96	TATA-box	752	757	core promoter element around -30 of transcription start
CsDof96	TATA-box	753	757	core promoter element around -30 of transcription start
CsDof96	TATA-box	823	829	core promoter element around -30 of transcription start
CsDof96	TATA-box	824	828	core promoter element around -30 of transcription start
CsDof96	TATA-box	855	863	core promoter element around -30 of transcription start
CsDof96	TATA-box	899	903	core promoter element around -30 of transcription start
CsDof96	TATA-box	1174	1183	core promoter element around -30 of transcription start
CsDof96	TATA-box	1175	1182	core promoter element around -30 of transcription start
CsDof96	TATA-box	1176	1182	core promoter element around -30 of transcription start
CsDof96	TATA-box	1177	1184	core promoter element around -30 of transcription start
CsDof96	TATA-box	1178	1184	core promoter element around -30 of transcription start
CsDof96	TATA-box	1179	1185	core promoter element around -30 of transcription start
CsDof96	TATA-box	1180	1186	core promoter element around -30 of transcription start
CsDof96	TATA-box	1181	1187	core promoter element around -30 of transcription start
CsDof96	TATA-box	1182	1186	core promoter element around -30 of transcription start
CsDof96	TATA-box	1251	1255	core promoter element around -30 of transcription start
CsDof96	TATA-box	1256	1261	core promoter element around -30 of transcription start
CsDof96	TATA-box	1257	1261	core promoter element around -30 of transcription start
CsDof96	TATA-box	1284	1291	core promoter element around -30 of transcription start
CsDof96	TATA-box	1285	1291	core promoter element around -30 of transcription start
CsDof96	TATA-box	1286	1291	core promoter element around -30 of transcription start
CsDof96	TATA-box	1287	1291	core promoter element around -30 of transcription start
CsDof96	TATA-box	1332	1338	core promoter element around -30 of transcription start
CsDof96	TATA-box	1333	1337	core promoter element around -30 of transcription start
CsDof96	TATA-box	1383	1389	core promoter element around -30 of transcription start
CsDof96	TATA-box	1384	1389	core promoter element around -30 of transcription start
CsDof96	TATA-box	1385	1389	core promoter element around -30 of transcription start
CsDof96	TATA-box	1398	1402	core promoter element around -30 of transcription start
CsDof96	TATA-box	1429	1437	core promoter element around -30 of transcription start
CsDof96	TATA-box	1486	1495	core promoter element around -30 of transcription start
CsDof96	TATA-box	1565	1573	core promoter element around -30 of transcription start
CsDof96	TATA-box	1656	1662	core promoter element around -30 of transcription start
CsDof96	TATA-box	1657	1662	core promoter element around -30 of transcription start
CsDof96	TATA-box	1658	1662	core promoter element around -30 of transcription start
CsDof96	TATA-box	1838	1844	core promoter element around -30 of transcription start
CsDof96	TATA-box	1840	1846	core promoter element around -30 of transcription start
CsDof96	TATA-box	1841	1847	core promoter element around -30 of transcription start
CsDof96	TATA-box	1842	1848	core promoter element around -30 of transcription start
CsDof96	TATA-box	1844	1848	core promoter element around -30 of transcription start
CsDof96	P-box	1864	1871	gibberellin-responsive element
CsDof96	GT1-motif	1196	1202	light responsive element
CsDof96	CCAAT-box	1003	1009	MYBHv1 binding site
CsDof97	ABRE	1380	1385	abscisic acid responsiveness
CsDof97	LTR	1640	1646	cis-acting element involved in low-temperature responsiveness
CsDof97	G-box	1379	1385	cis-acting regulatory element involved in light responsiveness
CsDof97	CGTCA-motif	1788	1793	cis-acting regulatory element involved in the MeJA-responsiveness
CsDof97	TGACG-motif	1788	1793	cis-acting regulatory element involved in the MeJA-responsiveness
CsDof97	CAT-box	796	802	cis-acting regulatory element related to meristem expression
CsDof97	CAAT-box	53	58	common cis-acting element in promoter and enhancer regions

Name	Cis-element	Start position	Stop position	Function
CsDof97	CAAT-box	770	775	common cis-acting element in promoter and enhancer regions
CsDof97	CAAT-box	837	842	common cis-acting element in promoter and enhancer regions
CsDof97	CAAT-box	891	896	common cis-acting element in promoter and enhancer regions
CsDof97	CAAT-box	924	929	common cis-acting element in promoter and enhancer regions
CsDof97	CAAT-box	979	984	common cis-acting element in promoter and enhancer regions
CsDof97	CAAT-box	1431	1436	common cis-acting element in promoter and enhancer regions
CsDof97	CAAT-box	1592	1597	common cis-acting element in promoter and enhancer regions
CsDof97	CAAT-box	1623	1628	common cis-acting element in promoter and enhancer regions
CsDof97	CAAT-box	1695	1700	common cis-acting element in promoter and enhancer regions
CsDof97	CAAT-box	1707	1712	common cis-acting element in promoter and enhancer regions
CsDof97	CAAT-box	1792	1797	common cis-acting element in promoter and enhancer regions
CsDof97	CAAT-box	1940	1945	common cis-acting element in promoter and enhancer regions
CsDof97	TATA-box	2	9	core promoter element around -30 of transcription start
CsDof97	TATA-box	133	140	core promoter element around -30 of transcription start
CsDof97	TATA-box	134	140	core promoter element around -30 of transcription start
CsDof97	TATA-box	136	140	core promoter element around -30 of transcription start
CsDof97	TATA-box	748	752	core promoter element around -30 of transcription start
CsDof97	TATA-box	805	809	core promoter element around -30 of transcription start
CsDof97	TATA-box	850	856	core promoter element around -30 of transcription start
CsDof97	TATA-box	851	857	core promoter element around -30 of transcription start
CsDof97	TATA-box	853	857	core promoter element around -30 of transcription start
CsDof97	TATA-box	915	921	core promoter element around -30 of transcription start
CsDof97	TATA-box	916	921	core promoter element around -30 of transcription start
CsDof97	TATA-box	917	921	core promoter element around -30 of transcription start
CsDof97	TATA-box	928	935	core promoter element around -30 of transcription start
CsDof97	TATA-box	1059	1066	core promoter element around -30 of transcription start
CsDof97	TATA-box	1060	1066	core promoter element around -30 of transcription start
CsDof97	TATA-box	1061	1067	core promoter element around -30 of transcription start
CsDof97	TATA-box	1062	1066	core promoter element around -30 of transcription start
CsDof97	TATA-box	1072	1079	core promoter element around -30 of transcription start
CsDof97	TATA-box	1079	1085	core promoter element around -30 of transcription start
CsDof97	TATA-box	1080	1084	core promoter element around -30 of transcription start
CsDof97	TATA-box	1132	1138	core promoter element around -30 of transcription start
CsDof97	TATA-box	1133	1138	core promoter element around -30 of transcription start
CsDof97	TATA-box	1134	1138	core promoter element around -30 of transcription start
CsDof97	TATA-box	1203	1210	core promoter element around -30 of transcription start
CsDof97	TATA-box	1204	1210	core promoter element around -30 of transcription start
CsDof97	TATA-box	1205	1210	core promoter element around -30 of transcription start
CsDof97	TATA-box	1206	1210	core promoter element around -30 of transcription start
CsDof97	TATA-box	1214	1220	core promoter element around -30 of transcription start
CsDof97	TATA-box	1215	1221	core promoter element around -30 of transcription start
CsDof97	TATA-box	1217	1221	core promoter element around -30 of transcription start
CsDof97	TATA-box	1257	1261	core promoter element around -30 of transcription start
CsDof97	TATA-box	1391	1397	core promoter element around -30 of transcription start
CsDof97	TATA-box	1393	1397	core promoter element around -30 of transcription start
CsDof97	TATA-box	1535	1541	core promoter element around -30 of transcription start
CsDof97	TATA-box	1536	1540	core promoter element around -30 of transcription start
CsDof97	TATA-box	1571	1577	core promoter element around -30 of transcription start
CsDof97	TATA-box	1572	1576	core promoter element around -30 of transcription start
CsDof97	TATA-box	1630	1636	core promoter element around -30 of transcription start
CsDof97	TATA-box	1631	1637	core promoter element around -30 of transcription start

Name	Cis-element	Start position	Stop position	Function
CsDof97	TATA-box	1633	1637	core promoter element around -30 of transcription start
CsDof97	TATA-box	1645	1651	core promoter element around -30 of transcription start
CsDof97	TATA-box	1646	1650	core promoter element around -30 of transcription start
CsDof97	TATA-box	1764	1771	core promoter element around -30 of transcription start
CsDof97	TATA-box	1970	1976	core promoter element around -30 of transcription start
CsDof97	TATA-box	1972	1976	core promoter element around -30 of transcription start
CsDof97	HD-Zip 1	1587	1595	element involved in differentiation of the palisade mesophyll cells
CsDof97	P-box	897	904	gibberellin-responsive element
CsDof97	AAAC-motif	28	39	light responsive element
CsDof97	AAAC-motif	954	965	light responsive element
CsDof97	GT1-motif	1410	1417	light responsive element
CsDof97	GT1-motif	1411	1417	light responsive element
CsDof98	ABRE	849	854	abscisic acid responsiveness
CsDof98	ABRE	1349	1354	abscisic acid responsiveness
CsDof98	TC-rich repeats	1321	1330	cis-acting element involved in defense and stress responsiveness
CsDof98	LTR	892	898	cis-acting element involved in low-temperature responsiveness
CsDof98	TCA-element	1260	1270	cis-acting element involved in salicylic acid responsiveness
CsDof98	ARE	77	83	cis-acting regulatory element essential for the anaerobic induction
CsDof98	ARE	604	610	cis-acting regulatory element essential for the anaerobic induction
CsDof98	ARE	1509	1515	cis-acting regulatory element essential for the anaerobic induction
CsDof98	ARE	1885	1891	cis-acting regulatory element essential for the anaerobic induction
CsDof98	G-box	252	261	cis-acting regulatory element involved in light responsiveness
CsDof98	G-box	848	854	cis-acting regulatory element involved in light responsiveness
CsDof98	G-box	1349	1355	cis-acting regulatory element involved in light responsiveness
CsDof98	TGACG-motif	488	493	cis-acting regulatory element involved in the MeJA-responsiveness
CsDof98	TGACG-motif	847	852	cis-acting regulatory element involved in the MeJA-responsiveness
CsDof98	CGTCA-motif	488	493	cis-acting regulatory element involved in the MeJA-responsiveness
CsDof98	CGTCA-motif	847	852	cis-acting regulatory element involved in the MeJA-responsiveness
CsDof98	O2-site	514	523	cis-acting regulatory element involved in zein metabolism regulation
CsDof98	O2-site	845	854	cis-acting regulatory element involved in zein metabolism regulation
CsDof98	O2-site	1825	1834	cis-acting regulatory element involved in zein metabolism regulation
CsDof98	CAAT-box	11	16	common cis-acting element in promoter and enhancer regions
CsDof98	CAAT-box	160	165	common cis-acting element in promoter and enhancer regions
CsDof98	CAAT-box	185	190	common cis-acting element in promoter and enhancer regions
CsDof98	CAAT-box	278	283	common cis-acting element in promoter and enhancer regions
CsDof98	CAAT-box	304	309	common cis-acting element in promoter and enhancer regions
CsDof98	CAAT-box	549	554	common cis-acting element in promoter and enhancer regions
CsDof98	CAAT-box	594	599	common cis-acting element in promoter and enhancer regions
CsDof98	CAAT-box	962	967	common cis-acting element in promoter and enhancer regions
CsDof98	CAAT-box	1141	1146	common cis-acting element in promoter and enhancer regions
CsDof98	CAAT-box	1245	1250	common cis-acting element in promoter and enhancer regions
CsDof98	CAAT-box	1305	1310	common cis-acting element in promoter and enhancer regions
CsDof98	CAAT-box	1334	1339	common cis-acting element in promoter and enhancer regions
CsDof98	CAAT-box	1454	1459	common cis-acting element in promoter and enhancer regions
CsDof98	CAAT-box	1468	1473	common cis-acting element in promoter and enhancer regions
CsDof98	CAAT-box	1512	1517	common cis-acting element in promoter and enhancer regions
CsDof98	CAAT-box	1700	1705	common cis-acting element in promoter and enhancer regions
CsDof98	CAAT-box	1861	1866	common cis-acting element in promoter and enhancer regions
CsDof98	CAAT-box	1868	1873	common cis-acting element in promoter and enhancer regions
CsDof98	CAAT-box	1899	1904	common cis-acting element in promoter and enhancer regions
CsDof98	CAAT-box	1978	1983	common cis-acting element in promoter and enhancer regions

Name	Cis-element	Start position	Stop position	Function
CsDof98	TATA-box	19	25	core promoter element around -30 of transcription start
CsDof98	TATA-box	20	24	core promoter element around -30 of transcription start
CsDof98	TATA-box	97	104	core promoter element around -30 of transcription start
CsDof98	TATA-box	98	104	core promoter element around -30 of transcription start
CsDof98	TATA-box	99	104	core promoter element around -30 of transcription start
CsDof98	TATA-box	100	104	core promoter element around -30 of transcription start
CsDof98	TATA-box	116	120	core promoter element around -30 of transcription start
CsDof98	TATA-box	129	135	core promoter element around -30 of transcription start
CsDof98	TATA-box	130	136	core promoter element around -30 of transcription start
CsDof98	TATA-box	131	135	core promoter element around -30 of transcription start
CsDof98	TATA-box	169	173	core promoter element around -30 of transcription start
CsDof98	TATA-box	200	204	core promoter element around -30 of transcription start
CsDof98	TATA-box	212	219	core promoter element around -30 of transcription start
CsDof98	TATA-box	471	477	core promoter element around -30 of transcription start
CsDof98	TATA-box	473	477	core promoter element around -30 of transcription start
CsDof98	TATA-box	499	503	core promoter element around -30 of transcription start
CsDof98	TATA-box	573	581	core promoter element around -30 of transcription start
CsDof98	TATA-box	956	961	core promoter element around -30 of transcription start
CsDof98	TATA-box	957	961	core promoter element around -30 of transcription start
CsDof98	TATA-box	1057	1061	core promoter element around -30 of transcription start
CsDof98	TATA-box	1067	1072	core promoter element around -30 of transcription start
CsDof98	TATA-box	1068	1072	core promoter element around -30 of transcription start
CsDof98	TATA-box	1088	1096	core promoter element around -30 of transcription start
CsDof98	TATA-box	1090	1099	core promoter element around -30 of transcription start
CsDof98	TATA-box	1197	1201	core promoter element around -30 of transcription start
CsDof98	TATA-box	1498	1503	core promoter element around -30 of transcription start
CsDof98	TATA-box	1499	1503	core promoter element around -30 of transcription start
CsDof98	TATA-box	1503	1509	core promoter element around -30 of transcription start
CsDof98	TATA-box	1504	1509	core promoter element around -30 of transcription start
CsDof98	TATA-box	1505	1509	core promoter element around -30 of transcription start
CsDof98	TATA-box	1525	1531	core promoter element around -30 of transcription start
CsDof98	TATA-box	1526	1530	core promoter element around -30 of transcription start
CsDof98	TATA-box	1540	1546	core promoter element around -30 of transcription start
CsDof98	TATA-box	1541	1546	core promoter element around -30 of transcription start
CsDof98	TATA-box	1542	1546	core promoter element around -30 of transcription start
CsDof98	TATA-box	1671	1677	core promoter element around -30 of transcription start
CsDof98	TATA-box	1672	1678	core promoter element around -30 of transcription start
CsDof98	TATA-box	1673	1677	core promoter element around -30 of transcription start
CsDof98	TATA-box	1743	1749	core promoter element around -30 of transcription start
CsDof98	TATA-box	1744	1749	core promoter element around -30 of transcription start
CsDof98	TATA-box	1745	1749	core promoter element around -30 of transcription start
CsDof98	TATA-box	1752	1756	core promoter element around -30 of transcription start
CsDof98	MRE	142	149	MYB binding site involved in light responsiveness
CsDof98	CCAAT-box	1032	1038	MYBHv1 binding site
CsDof98	CCAAT-box	1638	1644	MYBHv1 binding site
CsDof99	ABRE	996	1005	abscisic acid responsiveness
CsDof99	MSA-like	1278	1287	cis-acting element involved in cell cycle regulation
CsDof99	TC-rich repeats	985	994	cis-acting element involved in defense and stress responsiveness
CsDof99	ARE	898	904	cis-acting regulatory element essential for the anaerobic induction
CsDof99	ARE	947	953	cis-acting regulatory element essential for the anaerobic induction
CsDof99	ARE	1728	1734	cis-acting regulatory element essential for the anaerobic induction

Name	Cis-element	Start position	Stop position	Function
CsDof99	G-box	995	1006	cis-acting regulatory element involved in light responsiveness
CsDof99	CGTCA-motif	120	125	cis-acting regulatory element involved in the MeJA-responsiveness
CsDof99	CGTCA-motif	324	329	cis-acting regulatory element involved in the MeJA-responsiveness
CsDof99	CGTCA-motif	1203	1208	cis-acting regulatory element involved in the MeJA-responsiveness
CsDof99	CGTCA-motif	1312	1317	cis-acting regulatory element involved in the MeJA-responsiveness
CsDof99	TGACG-motif	120	125	cis-acting regulatory element involved in the MeJA-responsiveness
CsDof99	TGACG-motif	324	329	cis-acting regulatory element involved in the MeJA-responsiveness
CsDof99	TGACG-motif	1203	1208	cis-acting regulatory element involved in the MeJA-responsiveness
CsDof99	TGACG-motif	1312	1317	cis-acting regulatory element involved in the MeJA-responsiveness
CsDof99	O2-site	1296	1305	cis-acting regulatory element involved in zein metabolism regulation
CsDof99	CAAT-box	189	194	common cis-acting element in promoter and enhancer regions
CsDof99	CAAT-box	467	472	common cis-acting element in promoter and enhancer regions
CsDof99	CAAT-box	654	659	common cis-acting element in promoter and enhancer regions
CsDof99	CAAT-box	688	693	common cis-acting element in promoter and enhancer regions
CsDof99	CAAT-box	1286	1291	common cis-acting element in promoter and enhancer regions
CsDof99	CAAT-box	1300	1305	common cis-acting element in promoter and enhancer regions
CsDof99	CAAT-box	1433	1438	common cis-acting element in promoter and enhancer regions
CsDof99	CAAT-box	1527	1532	common cis-acting element in promoter and enhancer regions
CsDof99	CAAT-box	1941	1946	common cis-acting element in promoter and enhancer regions
CsDof99	TATA-box	153	160	core promoter element around -30 of transcription start
CsDof99	TATA-box	154	160	core promoter element around -30 of transcription start
CsDof99	TATA-box	155	160	core promoter element around -30 of transcription start
CsDof99	TATA-box	156	160	core promoter element around -30 of transcription start
CsDof99	TATA-box	211	218	core promoter element around -30 of transcription start
CsDof99	TATA-box	212	218	core promoter element around -30 of transcription start
CsDof99	TATA-box	213	218	core promoter element around -30 of transcription start
CsDof99	TATA-box	214	218	core promoter element around -30 of transcription start
CsDof99	TATA-box	448	454	core promoter element around -30 of transcription start
CsDof99	TATA-box	449	453	core promoter element around -30 of transcription start
CsDof99	TATA-box	452	458	core promoter element around -30 of transcription start
CsDof99	TATA-box	453	458	core promoter element around -30 of transcription start
CsDof99	TATA-box	454	458	core promoter element around -30 of transcription start
CsDof99	TATA-box	540	546	core promoter element around -30 of transcription start
CsDof99	TATA-box	541	547	core promoter element around -30 of transcription start
CsDof99	TATA-box	542	548	core promoter element around -30 of transcription start
CsDof99	TATA-box	544	548	core promoter element around -30 of transcription start
CsDof99	TATA-box	732	739	core promoter element around -30 of transcription start
CsDof99	TATA-box	733	739	core promoter element around -30 of transcription start
CsDof99	TATA-box	734	739	core promoter element around -30 of transcription start
CsDof99	TATA-box	735	739	core promoter element around -30 of transcription start
CsDof99	TATA-box	824	830	core promoter element around -30 of transcription start
CsDof99	TATA-box	825	829	core promoter element around -30 of transcription start
CsDof99	TATA-box	827	835	core promoter element around -30 of transcription start
CsDof99	TATA-box	872	880	core promoter element around -30 of transcription start
CsDof99	TATA-box	878	882	core promoter element around -30 of transcription start
CsDof99	TATA-box	968	974	core promoter element around -30 of transcription start
CsDof99	TATA-box	969	974	core promoter element around -30 of transcription start
CsDof99	TATA-box	970	974	core promoter element around -30 of transcription start
CsDof99	TATA-box	1400	1404	core promoter element around -30 of transcription start
CsDof99	TATA-box	1407	1412	core promoter element around -30 of transcription start
CsDof99	TATA-box	1408	1412	core promoter element around -30 of transcription start

Name	Cis-element	Start position	Stop position	Function
CsDof99	TATA-box	1637	1641	core promoter element around -30 of transcription start
CsDof99	TATA-box	1697	1703	core promoter element around -30 of transcription start
CsDof99	TATA-box	1698	1702	core promoter element around -30 of transcription start
CsDof99	TATA-box	1742	1748	core promoter element around -30 of transcription start
CsDof99	TATA-box	1743	1748	core promoter element around -30 of transcription start
CsDof99	TATA-box	1744	1748	core promoter element around -30 of transcription start
CsDof99	TATA-box	1753	1762	core promoter element around -30 of transcription start
CsDof99	TATA-box	1754	1761	core promoter element around -30 of transcription start
CsDof99	TATA-box	1755	1761	core promoter element around -30 of transcription start
CsDof99	TATA-box	1756	1763	core promoter element around -30 of transcription start
CsDof99	TATA-box	1757	1763	core promoter element around -30 of transcription start
CsDof99	TATA-box	1758	1764	core promoter element around -30 of transcription start
CsDof99	TATA-box	1759	1765	core promoter element around -30 of transcription start
CsDof99	TATA-box	1760	1766	core promoter element around -30 of transcription start
CsDof99	TATA-box	1761	1765	core promoter element around -30 of transcription start
CsDof99	TATA-box	1886	1891	core promoter element around -30 of transcription start
CsDof99	TATA-box	1887	1891	core promoter element around -30 of transcription start
CsDof99	AT-rich sequence	149	158	element for maximal elicitor-mediated activation (2copies)
CsDof99	GARE-motif	1194	1201	gibberellin-responsive element
CsDof99	CCAAT-box	1279	1285	MYBHv1 binding site
CsDof99	HD-Zip 3	263	272.5	protein binding site
CsDof100	TCA-element	97	106	cis-acting element involved in salicylic acid responsiveness
CsDof100	TCA-element	609	618	cis-acting element involved in salicylic acid responsiveness
CsDof100	TCA-element	1198	1207	cis-acting element involved in salicylic acid responsiveness
CsDof100	ARE	556	562	cis-acting regulatory element essential for the anaerobic induction
CsDof100	ARE	1068	1074	cis-acting regulatory element essential for the anaerobic induction
CsDof100	TGACG-motif	399	404	cis-acting regulatory element involved in the MeJA-responsiveness
CsDof100	TGACG-motif	911	916	cis-acting regulatory element involved in the MeJA-responsiveness
CsDof100	CGTCA-motif	399	404	cis-acting regulatory element involved in the MeJA-responsiveness
CsDof100	CGTCA-motif	911	916	cis-acting regulatory element involved in the MeJA-responsiveness
CsDof100	O2-site	1985	1994	cis-acting regulatory element involved in zein metabolism regulation
CsDof100	CAT-box	1335	1341	cis-acting regulatory element related to meristem expression
CsDof100	CAT-box	1490	1496	cis-acting regulatory element related to meristem expression
CsDof100	CAAT-box	133	138	common cis-acting element in promoter and enhancer regions
CsDof100	CAAT-box	153	158	common cis-acting element in promoter and enhancer regions
CsDof100	CAAT-box	167	172	common cis-acting element in promoter and enhancer regions
CsDof100	CAAT-box	190	195	common cis-acting element in promoter and enhancer regions
CsDof100	CAAT-box	323	328	common cis-acting element in promoter and enhancer regions
CsDof100	CAAT-box	373	378	common cis-acting element in promoter and enhancer regions
CsDof100	CAAT-box	404	409	common cis-acting element in promoter and enhancer regions
CsDof100	CAAT-box	546	551	common cis-acting element in promoter and enhancer regions
CsDof100	CAAT-box	645	650	common cis-acting element in promoter and enhancer regions
CsDof100	CAAT-box	665	670	common cis-acting element in promoter and enhancer regions
CsDof100	CAAT-box	679	684	common cis-acting element in promoter and enhancer regions
CsDof100	CAAT-box	702	707	common cis-acting element in promoter and enhancer regions
CsDof100	CAAT-box	835	840	common cis-acting element in promoter and enhancer regions
CsDof100	CAAT-box	885	890	common cis-acting element in promoter and enhancer regions
CsDof100	CAAT-box	916	921	common cis-acting element in promoter and enhancer regions
CsDof100	CAAT-box	1058	1063	common cis-acting element in promoter and enhancer regions
CsDof100	CAAT-box	1274	1279	common cis-acting element in promoter and enhancer regions
CsDof100	CAAT-box	1287	1292	common cis-acting element in promoter and enhancer regions

Name	Cis-element	Start position	Stop position	Function
CsDof100	CAAT-box	1579	1584	common cis-acting element in promoter and enhancer regions
CsDof100	TATA-box	60	66	core promoter element around -30 of transcription start
CsDof100	TATA-box	61	65	core promoter element around -30 of transcription start
CsDof100	TATA-box	106	113	core promoter element around -30 of transcription start
CsDof100	TATA-box	107	113	core promoter element around -30 of transcription start
CsDof100	TATA-box	108	113	core promoter element around -30 of transcription start
CsDof100	TATA-box	109	113	core promoter element around -30 of transcription start
CsDof100	TATA-box	113	120	core promoter element around -30 of transcription start
CsDof100	TATA-box	114	120	core promoter element around -30 of transcription start
CsDof100	TATA-box	115	120	core promoter element around -30 of transcription start
CsDof100	TATA-box	116	120	core promoter element around -30 of transcription start
CsDof100	TATA-box	211	215	core promoter element around -30 of transcription start
CsDof100	TATA-box	224	230	core promoter element around -30 of transcription start
CsDof100	TATA-box	225	231	core promoter element around -30 of transcription start
CsDof100	TATA-box	226	230	core promoter element around -30 of transcription start
CsDof100	TATA-box	314	321	core promoter element around -30 of transcription start
CsDof100	TATA-box	330	336	core promoter element around -30 of transcription start
CsDof100	TATA-box	331	336	core promoter element around -30 of transcription start
CsDof100	TATA-box	332	336	core promoter element around -30 of transcription start
CsDof100	TATA-box	343	348	core promoter element around -30 of transcription start
CsDof100	TATA-box	344	348	core promoter element around -30 of transcription start
CsDof100	TATA-box	379	386	core promoter element around -30 of transcription start
CsDof100	TATA-box	380	386	core promoter element around -30 of transcription start
CsDof100	TATA-box	381	386	core promoter element around -30 of transcription start
CsDof100	TATA-box	382	386	core promoter element around -30 of transcription start
CsDof100	TATA-box	390	396	core promoter element around -30 of transcription start
CsDof100	TATA-box	391	397	core promoter element around -30 of transcription start
CsDof100	TATA-box	392	398	core promoter element around -30 of transcription start
CsDof100	TATA-box	393	399	core promoter element around -30 of transcription start
CsDof100	TATA-box	394	398	core promoter element around -30 of transcription start
CsDof100	TATA-box	414	420	core promoter element around -30 of transcription start
CsDof100	TATA-box	415	419	core promoter element around -30 of transcription start
CsDof100	TATA-box	532	538	core promoter element around -30 of transcription start
CsDof100	TATA-box	533	537	core promoter element around -30 of transcription start
CsDof100	TATA-box	591	597	core promoter element around -30 of transcription start
CsDof100	TATA-box	592	598	core promoter element around -30 of transcription start
CsDof100	TATA-box	593	597	core promoter element around -30 of transcription start
CsDof100	TATA-box	618	625	core promoter element around -30 of transcription start
CsDof100	TATA-box	619	625	core promoter element around -30 of transcription start
CsDof100	TATA-box	620	625	core promoter element around -30 of transcription start
CsDof100	TATA-box	621	625	core promoter element around -30 of transcription start
CsDof100	TATA-box	625	632	core promoter element around -30 of transcription start
CsDof100	TATA-box	626	632	core promoter element around -30 of transcription start
CsDof100	TATA-box	627	632	core promoter element around -30 of transcription start
CsDof100	TATA-box	628	632	core promoter element around -30 of transcription start
CsDof100	TATA-box	723	727	core promoter element around -30 of transcription start
CsDof100	TATA-box	736	742	core promoter element around -30 of transcription start
CsDof100	TATA-box	737	743	core promoter element around -30 of transcription start
CsDof100	TATA-box	738	742	core promoter element around -30 of transcription start
CsDof100	TATA-box	826	833	core promoter element around -30 of transcription start
CsDof100	TATA-box	842	848	core promoter element around -30 of transcription start

Name	Cis-element	Start position	Stop position	Function
CsDof100	TATA-box	843	848	core promoter element around -30 of transcription start
CsDof100	TATA-box	844	848	core promoter element around -30 of transcription start
CsDof100	TATA-box	855	860	core promoter element around -30 of transcription start
CsDof100	TATA-box	856	860	core promoter element around -30 of transcription start
CsDof100	TATA-box	891	898	core promoter element around -30 of transcription start
CsDof100	TATA-box	892	898	core promoter element around -30 of transcription start
CsDof100	TATA-box	893	898	core promoter element around -30 of transcription start
CsDof100	TATA-box	894	898	core promoter element around -30 of transcription start
CsDof100	TATA-box	902	908	core promoter element around -30 of transcription start
CsDof100	TATA-box	903	909	core promoter element around -30 of transcription start
CsDof100	TATA-box	904	910	core promoter element around -30 of transcription start
CsDof100	TATA-box	905	911	core promoter element around -30 of transcription start
CsDof100	TATA-box	906	910	core promoter element around -30 of transcription start
CsDof100	TATA-box	926	932	core promoter element around -30 of transcription start
CsDof100	TATA-box	927	931	core promoter element around -30 of transcription start
CsDof100	TATA-box	1044	1050	core promoter element around -30 of transcription start
CsDof100	TATA-box	1045	1049	core promoter element around -30 of transcription start
CsDof100	TATA-box	1103	1109	core promoter element around -30 of transcription start
CsDof100	TATA-box	1104	1110	core promoter element around -30 of transcription start
CsDof100	TATA-box	1105	1109	core promoter element around -30 of transcription start
CsDof100	TATA-box	1111	1115	core promoter element around -30 of transcription start
CsDof100	TATA-box	1150	1156	core promoter element around -30 of transcription start
CsDof100	TATA-box	1152	1158	core promoter element around -30 of transcription start
CsDof100	TATA-box	1153	1159	core promoter element around -30 of transcription start
CsDof100	TATA-box	1154	1158	core promoter element around -30 of transcription start
CsDof100	TATA-box	1180	1186	core promoter element around -30 of transcription start
CsDof100	TATA-box	1181	1187	core promoter element around -30 of transcription start
CsDof100	TATA-box	1183	1187	core promoter element around -30 of transcription start
CsDof100	TATA-box	1188	1194	core promoter element around -30 of transcription start
CsDof100	TATA-box	1189	1194	core promoter element around -30 of transcription start
CsDof100	TATA-box	1190	1194	core promoter element around -30 of transcription start
CsDof100	TATA-box	1197	1201	core promoter element around -30 of transcription start
CsDof100	TATA-box	1229	1238	core promoter element around -30 of transcription start
CsDof100	TATA-box	1230	1236	core promoter element around -30 of transcription start
CsDof100	TATA-box	1231	1235	core promoter element around -30 of transcription start
CsDof100	TATA-box	1268	1274	core promoter element around -30 of transcription start
CsDof100	TATA-box	1269	1273	core promoter element around -30 of transcription start
CsDof100	TATA-box	1310	1317	core promoter element around -30 of transcription start
CsDof100	TATA-box	1311	1317	core promoter element around -30 of transcription start
CsDof100	TATA-box	1312	1317	core promoter element around -30 of transcription start
CsDof100	TATA-box	1313	1317	core promoter element around -30 of transcription start
CsDof100	TATA-box	1410	1416	core promoter element around -30 of transcription start
CsDof100	TATA-box	1411	1417	core promoter element around -30 of transcription start
CsDof100	TATA-box	1412	1418	core promoter element around -30 of transcription start
CsDof100	TATA-box	1413	1419	core promoter element around -30 of transcription start
CsDof100	TATA-box	1414	1420	core promoter element around -30 of transcription start
CsDof100	TATA-box	1415	1421	core promoter element around -30 of transcription start
CsDof100	TATA-box	1416	1422	core promoter element around -30 of transcription start
CsDof100	TATA-box	1417	1423	core promoter element around -30 of transcription start
CsDof100	TATA-box	1418	1422	core promoter element around -30 of transcription start
CsDof100	TATA-box	1424	1430	core promoter element around -30 of transcription start

Name	Cis-element	Start position	Stop position	Function
CsDof100	TATA-box	1425	1431	core promoter element around -30 of transcription start
CsDof100	TATA-box	1426	1430	core promoter element around -30 of transcription start
CsDof100	TATA-box	1607	1611	core promoter element around -30 of transcription start
CsDof100	TATA-box	1681	1687	core promoter element around -30 of transcription start
CsDof100	TATA-box	1682	1686	core promoter element around -30 of transcription start
CsDof100	TATA-box	1702	1708	core promoter element around -30 of transcription start
CsDof100	TATA-box	1703	1709	core promoter element around -30 of transcription start
CsDof100	TATA-box	1705	1709	core promoter element around -30 of transcription start
CsDof100	TATA-box	1730	1738	core promoter element around -30 of transcription start
CsDof100	TATA-box	1735	1741	core promoter element around -30 of transcription start
CsDof100	TATA-box	1736	1742	core promoter element around -30 of transcription start
CsDof100	TATA-box	1737	1743	core promoter element around -30 of transcription start
CsDof100	TATA-box	1738	1742	core promoter element around -30 of transcription start
CsDof100	TATA-box	1771	1777	core promoter element around -30 of transcription start
CsDof100	TATA-box	1772	1778	core promoter element around -30 of transcription start
CsDof100	TATA-box	1773	1777	core promoter element around -30 of transcription start
CsDof100	TATA-box	1784	1788	core promoter element around -30 of transcription start
CsDof100	TATA-box	1789	1795	core promoter element around -30 of transcription start
CsDof100	TATA-box	1790	1794	core promoter element around -30 of transcription start
CsDof100	TATA-box	1801	1807	core promoter element around -30 of transcription start
CsDof100	TATA-box	1802	1808	core promoter element around -30 of transcription start
CsDof100	TATA-box	1803	1809	core promoter element around -30 of transcription start
CsDof100	TATA-box	1804	1810	core promoter element around -30 of transcription start
CsDof100	TATA-box	1805	1811	core promoter element around -30 of transcription start
CsDof100	TATA-box	1806	1810	core promoter element around -30 of transcription start
CsDof100	TATA-box	1816	1825	core promoter element around -30 of transcription start
CsDof100	TATA-box	1817	1824	core promoter element around -30 of transcription start
CsDof100	TATA-box	1818	1824	core promoter element around -30 of transcription start
CsDof100	TATA-box	1819	1826	core promoter element around -30 of transcription start
CsDof100	TATA-box	1820	1826	core promoter element around -30 of transcription start
CsDof100	TATA-box	1821	1827	core promoter element around -30 of transcription start
CsDof100	TATA-box	1822	1828	core promoter element around -30 of transcription start
CsDof100	TATA-box	1823	1829	core promoter element around -30 of transcription start
CsDof100	TATA-box	1824	1828	core promoter element around -30 of transcription start
CsDof100	TATA-box	1834	1838	core promoter element around -30 of transcription start
CsDof100	GARE-motif	1566	1573	gibberellin-responsive element
CsDof100	MBS	1518	1524	MYB binding site involved in drought-inducibility
CsDof101	ABRE	603	608	abscisic acid responsiveness
CsDof101	ABRE	1097	1102	abscisic acid responsiveness
CsDof101	ABRE	1453	1461	abscisic acid responsiveness
CsDof101	ABRE	1455	1460	abscisic acid responsiveness
CsDof101	ABRE	1686	1691	abscisic acid responsiveness
CsDof101	TATC-box	515	522	cis-acting element involved in gibberellin-responsiveness
CsDof101	TCA-element	1275	1284	cis-acting element involved in salicylic acid responsiveness
CsDof101	A-box	429	435	cis-acting regulatory element
CsDof101	A-box	580	586	cis-acting regulatory element
CsDof101	ARE	13	19	cis-acting regulatory element essential for the anaerobic induction
CsDof101	AuxRR-core	1238	1245	cis-acting regulatory element involved in auxin responsiveness
CsDof101	G-box	603	609	cis-acting regulatory element involved in light responsiveness
CsDof101	G-box	1094	1103	cis-acting regulatory element involved in light responsiveness
CsDof101	G-box	1097	1103	cis-acting regulatory element involved in light responsiveness

Name	Cis-element	Start position	Stop position	Function
CsDof101	G-box	1455	1461	cis-acting regulatory element involved in light responsiveness
CsDof101	G-Box	1685	1691	cis-acting regulatory element involved in light responsiveness
CsDof101	CGTCA-motif	438	443	cis-acting regulatory element involved in the MeJA-responsiveness
CsDof101	TGACG-motif	438	443	cis-acting regulatory element involved in the MeJA-responsiveness
CsDof101	O2-site	1501	1510	cis-acting regulatory element involved in zein metabolism regulation
CsDof101	CAAT-box	355	360	common cis-acting element in promoter and enhancer regions
CsDof101	CAAT-box	363	368	common cis-acting element in promoter and enhancer regions
CsDof101	CAAT-box	543	548	common cis-acting element in promoter and enhancer regions
CsDof101	CAAT-box	646	651	common cis-acting element in promoter and enhancer regions
CsDof101	CAAT-box	683	692	common cis-acting element in promoter and enhancer regions
CsDof101	CAAT-box	685	690	common cis-acting element in promoter and enhancer regions
CsDof101	CAAT-box	836	841	common cis-acting element in promoter and enhancer regions
CsDof101	CAAT-box	885	890	common cis-acting element in promoter and enhancer regions
CsDof101	CAAT-box	1063	1068	common cis-acting element in promoter and enhancer regions
CsDof101	CAAT-box	1142	1147	common cis-acting element in promoter and enhancer regions
CsDof101	CAAT-box	1371	1376	common cis-acting element in promoter and enhancer regions
CsDof101	CAAT-box	1409	1414	common cis-acting element in promoter and enhancer regions
CsDof101	CAAT-box	1592	1597	common cis-acting element in promoter and enhancer regions
CsDof101	CAAT-box	1637	1642	common cis-acting element in promoter and enhancer regions
CsDof101	TATA-box	41	46	core promoter element around -30 of transcription start
CsDof101	TATA-box	42	46	core promoter element around -30 of transcription start
CsDof101	TATA-box	113	119	core promoter element around -30 of transcription start
CsDof101	TATA-box	114	118	core promoter element around -30 of transcription start
CsDof101	TATA-box	343	350	core promoter element around -30 of transcription start
CsDof101	TATA-box	382	386	core promoter element around -30 of transcription start
CsDof101	TATA-box	992	1001	core promoter element around -30 of transcription start
CsDof101	TATA-box	993	1000	core promoter element around -30 of transcription start
CsDof101	TATA-box	994	1000	core promoter element around -30 of transcription start
CsDof101	TATA-box	995	1000	core promoter element around -30 of transcription start
CsDof101	TATA-box	996	1000	core promoter element around -30 of transcription start
CsDof101	TATA-box	1020	1026	core promoter element around -30 of transcription start
CsDof101	TATA-box	1021	1026	core promoter element around -30 of transcription start
CsDof101	TATA-box	1022	1026	core promoter element around -30 of transcription start
CsDof101	TATA-box	1071	1078	core promoter element around -30 of transcription start
CsDof101	TATA-box	1072	1078	core promoter element around -30 of transcription start
CsDof101	TATA-box	1073	1078	core promoter element around -30 of transcription start
CsDof101	TATA-box	1074	1078	core promoter element around -30 of transcription start
CsDof101	TATA-box	1193	1197	core promoter element around -30 of transcription start
CsDof101	TATA-box	1325	1330	core promoter element around -30 of transcription start
CsDof101	TATA-box	1326	1330	core promoter element around -30 of transcription start
CsDof101	TATA-box	1356	1360	core promoter element around -30 of transcription start
CsDof101	TATA-box	1669	1674	core promoter element around -30 of transcription start
CsDof101	TATA-box	1670	1674	core promoter element around -30 of transcription start
CsDof101	TATA-box	1769	1773	core promoter element around -30 of transcription start
CsDof101	TATA-box	1896	1902	core promoter element around -30 of transcription start
CsDof101	TATA-box	1897	1901	core promoter element around -30 of transcription start
CsDof101	GARE-motif	569	576	gibberellin-responsive element
CsDof101	GT1-motif	1216	1225	light responsive element
CsDof102	ABRE	118	123	abscisic acid responsiveness
CsDof102	ABRE	344	350	abscisic acid responsiveness
CsDof102	ABRE	345	350	abscisic acid responsiveness

Name	Cis-element	Start position	Stop position	Function
CsDof102	ABRE	787	792	abscisic acid responsiveness
CsDof102	TGA-element	1800	1806	auxin-responsive element
CsDof102	AT-rich element	1243	1253	binding site of AT-rich DNA binding protein (ATBP-1)
CsDof102	TCA-element	650	659	cis-acting element involved in salicylic acid responsiveness
CsDof102	A-box	255	261	cis-acting regulatory element
CsDof102	A-box	443	449	cis-acting regulatory element
CsDof102	ARE	1095	1101	cis-acting regulatory element essential for the anaerobic induction
CsDof102	ARE	1300	1306	cis-acting regulatory element essential for the anaerobic induction
CsDof102	ARE	1384	1390	cis-acting regulatory element essential for the anaerobic induction
CsDof102	G-box	344	350	cis-acting regulatory element involved in light responsiveness
CsDof102	G-box	786	792	cis-acting regulatory element involved in light responsiveness
CsDof102	G-Box	118	124	cis-acting regulatory element involved in light responsiveness
CsDof102	G-Box	344	350	cis-acting regulatory element involved in light responsiveness
CsDof102	TGACG-motif	299	304	cis-acting regulatory element involved in the MeJA-responsiveness
CsDof102	TGACG-motif	516	521	cis-acting regulatory element involved in the MeJA-responsiveness
CsDof102	TGACG-motif	862	867	cis-acting regulatory element involved in the MeJA-responsiveness
CsDof102	TGACG-motif	885	890	cis-acting regulatory element involved in the MeJA-responsiveness
CsDof102	CGTCA-motif	299	304	cis-acting regulatory element involved in the MeJA-responsiveness
CsDof102	CGTCA-motif	516	521	cis-acting regulatory element involved in the MeJA-responsiveness
CsDof102	CGTCA-motif	862	867	cis-acting regulatory element involved in the MeJA-responsiveness
CsDof102	CGTCA-motif	885	890	cis-acting regulatory element involved in the MeJA-responsiveness
CsDof102	CAAT-box	62	67	common cis-acting element in promoter and enhancer regions
CsDof102	CAAT-box	137	142	common cis-acting element in promoter and enhancer regions
CsDof102	CAAT-box	143	148	common cis-acting element in promoter and enhancer regions
CsDof102	CAAT-box	196	201	common cis-acting element in promoter and enhancer regions
CsDof102	CAAT-box	204	209	common cis-acting element in promoter and enhancer regions
CsDof102	CAAT-box	280	285	common cis-acting element in promoter and enhancer regions
CsDof102	CAAT-box	375	380	common cis-acting element in promoter and enhancer regions
CsDof102	CAAT-box	463	468	common cis-acting element in promoter and enhancer regions
CsDof102	CAAT-box	567	572	common cis-acting element in promoter and enhancer regions
CsDof102	CAAT-box	707	712	common cis-acting element in promoter and enhancer regions
CsDof102	CAAT-box	710	715	common cis-acting element in promoter and enhancer regions
CsDof102	CAAT-box	988	993	common cis-acting element in promoter and enhancer regions
CsDof102	CAAT-box	1013	1018	common cis-acting element in promoter and enhancer regions
CsDof102	CAAT-box	1031	1036	common cis-acting element in promoter and enhancer regions
CsDof102	CAAT-box	1237	1242	common cis-acting element in promoter and enhancer regions
CsDof102	CAAT-box	1273	1278	common cis-acting element in promoter and enhancer regions
CsDof102	CAAT-box	1330	1335	common cis-acting element in promoter and enhancer regions
CsDof102	CAAT-box	1361	1366	common cis-acting element in promoter and enhancer regions
CsDof102	CAAT-box	1417	1422	common cis-acting element in promoter and enhancer regions
CsDof102	CAAT-box	1869	1874	common cis-acting element in promoter and enhancer regions
CsDof102	CAAT-box	1947	1952	common cis-acting element in promoter and enhancer regions
CsDof102	CAAT-box	1953	1958	common cis-acting element in promoter and enhancer regions
CsDof102	TATA-box	29	37	core promoter element around -30 of transcription start
CsDof102	TATA-box	33	41	core promoter element around -30 of transcription start
CsDof102	TATA-box	46	50	core promoter element around -30 of transcription start
CsDof102	TATA-box	96	100	core promoter element around -30 of transcription start
CsDof102	TATA-box	631	638	core promoter element around -30 of transcription start
CsDof102	TATA-box	632	638	core promoter element around -30 of transcription start
CsDof102	TATA-box	633	640	core promoter element around -30 of transcription start
CsDof102	TATA-box	634	640	core promoter element around -30 of transcription start

Name	Cis-element	Start position	Stop position	Function
CsDof102	TATA-box	635	641	core promoter element around -30 of transcription start
CsDof102	TATA-box	636	642	core promoter element around -30 of transcription start
CsDof102	TATA-box	637	643	core promoter element around -30 of transcription start
CsDof102	TATA-box	638	642	core promoter element around -30 of transcription start
CsDof102	TATA-box	642	648	core promoter element around -30 of transcription start
CsDof102	TATA-box	643	648	core promoter element around -30 of transcription start
CsDof102	TATA-box	644	648	core promoter element around -30 of transcription start
CsDof102	TATA-box	675	679	core promoter element around -30 of transcription start
CsDof102	TATA-box	719	725	core promoter element around -30 of transcription start
CsDof102	TATA-box	721	725	core promoter element around -30 of transcription start
CsDof102	TATA-box	942	948	core promoter element around -30 of transcription start
CsDof102	TATA-box	943	950	core promoter element around -30 of transcription start
CsDof102	TATA-box	944	950	core promoter element around -30 of transcription start
CsDof102	TATA-box	945	951	core promoter element around -30 of transcription start
CsDof102	TATA-box	946	950	core promoter element around -30 of transcription start
CsDof102	TATA-box	1473	1479	core promoter element around -30 of transcription start
CsDof102	TATA-box	1474	1478	core promoter element around -30 of transcription start
CsDof102	TATA-box	1512	1518	core promoter element around -30 of transcription start
CsDof102	TATA-box	1513	1518	core promoter element around -30 of transcription start
CsDof102	TATA-box	1514	1518	core promoter element around -30 of transcription start
CsDof102	TATA-box	1563	1569	core promoter element around -30 of transcription start
CsDof102	TATA-box	1564	1570	core promoter element around -30 of transcription start
CsDof102	TATA-box	1566	1570	core promoter element around -30 of transcription start
CsDof102	TATA-box	1643	1650	core promoter element around -30 of transcription start
CsDof102	TATA-box	1674	1680	core promoter element around -30 of transcription start
CsDof102	TATA-box	1675	1679	core promoter element around -30 of transcription start
CsDof102	TATA-box	1684	1688	core promoter element around -30 of transcription start
CsDof102	TATA-box	1758	1762	core promoter element around -30 of transcription start
CsDof102	Sp1	830	836	light responsive element
CsDof102	GT1-motif	1222	1228	light responsive element
CsDof102	AAAC-motif	1914	1925	light responsive element
CsDof102	MRE	900	907	MYB binding site involved in light responsiveness
CsDof103	ABRE	1209	1214	abscisic acid responsiveness
CsDof103	LTR	536	542	cis-acting element involved in low-temperature responsiveness
CsDof103	ARE	300	306	cis-acting regulatory element essential for the anaerobic induction
CsDof103	ARE	436	442	cis-acting regulatory element essential for the anaerobic induction
CsDof103	ARE	472	478	cis-acting regulatory element essential for the anaerobic induction
CsDof103	ARE	1945	1951	cis-acting regulatory element essential for the anaerobic induction
CsDof103	G-box	1209	1215	cis-acting regulatory element involved in light responsiveness
CsDof103	TGACG-motif	19	24	cis-acting regulatory element involved in the MeJA-responsiveness
CsDof103	TGACG-motif	964	969	cis-acting regulatory element involved in the MeJA-responsiveness
CsDof103	TGACG-motif	1019	1024	cis-acting regulatory element involved in the MeJA-responsiveness
CsDof103	CGTCA-motif	19	24	cis-acting regulatory element involved in the MeJA-responsiveness
CsDof103	CGTCA-motif	964	969	cis-acting regulatory element involved in the MeJA-responsiveness
CsDof103	CGTCA-motif	1019	1024	cis-acting regulatory element involved in the MeJA-responsiveness
CsDof103	CAT-box	1247	1253	cis-acting regulatory element related to meristem expression
CsDof103	CAAT-box	274	279	common cis-acting element in promoter and enhancer regions
CsDof103	CAAT-box	336	341	common cis-acting element in promoter and enhancer regions
CsDof103	CAAT-box	353	358	common cis-acting element in promoter and enhancer regions
CsDof103	CAAT-box	368	373	common cis-acting element in promoter and enhancer regions
CsDof103	CAAT-box	462	467	common cis-acting element in promoter and enhancer regions

Name	Cis-element	Start position	Stop position	Function
CsDof103	CAAT-box	614	619	common cis-acting element in promoter and enhancer regions
CsDof103	CAAT-box	840	845	common cis-acting element in promoter and enhancer regions
CsDof103	CAAT-box	942	947	common cis-acting element in promoter and enhancer regions
CsDof103	CAAT-box	1463	1468	common cis-acting element in promoter and enhancer regions
CsDof103	CAAT-box	1728	1733	common cis-acting element in promoter and enhancer regions
CsDof103	CAAT-box	1930	1935	common cis-acting element in promoter and enhancer regions
CsDof103	TATA-box	279	283	core promoter element around -30 of transcription start
CsDof103	TATA-box	290	296	core promoter element around -30 of transcription start
CsDof103	TATA-box	291	296	core promoter element around -30 of transcription start
CsDof103	TATA-box	292	296	core promoter element around -30 of transcription start
CsDof103	TATA-box	363	372	core promoter element around -30 of transcription start
CsDof103	TATA-box	557	561	core promoter element around -30 of transcription start
CsDof103	TATA-box	725	734	core promoter element around -30 of transcription start
CsDof103	TATA-box	726	732	core promoter element around -30 of transcription start
CsDof103	TATA-box	727	734	core promoter element around -30 of transcription start
CsDof103	TATA-box	728	734	core promoter element around -30 of transcription start
CsDof103	TATA-box	729	735	core promoter element around -30 of transcription start
CsDof103	TATA-box	730	736	core promoter element around -30 of transcription start
CsDof103	TATA-box	732	736	core promoter element around -30 of transcription start
CsDof103	TATA-box	795	802	core promoter element around -30 of transcription start
CsDof103	TATA-box	796	802	core promoter element around -30 of transcription start
CsDof103	TATA-box	797	802	core promoter element around -30 of transcription start
CsDof103	TATA-box	798	802	core promoter element around -30 of transcription start
CsDof103	TATA-box	802	808	core promoter element around -30 of transcription start
CsDof103	TATA-box	803	807	core promoter element around -30 of transcription start
CsDof103	TATA-box	913	919	core promoter element around -30 of transcription start
CsDof103	TATA-box	914	919	core promoter element around -30 of transcription start
CsDof103	TATA-box	915	919	core promoter element around -30 of transcription start
CsDof103	TATA-box	936	940	core promoter element around -30 of transcription start
CsDof103	TATA-box	1035	1041	core promoter element around -30 of transcription start
CsDof103	TATA-box	1036	1041	core promoter element around -30 of transcription start
CsDof103	TATA-box	1037	1041	core promoter element around -30 of transcription start
CsDof103	TATA-box	1092	1099	core promoter element around -30 of transcription start
CsDof103	TATA-box	1149	1158	core promoter element around -30 of transcription start
CsDof103	TATA-box	1154	1160	core promoter element around -30 of transcription start
CsDof103	TATA-box	1155	1159	core promoter element around -30 of transcription start
CsDof103	TATA-box	1201	1207	core promoter element around -30 of transcription start
CsDof103	TATA-box	1202	1208	core promoter element around -30 of transcription start
CsDof103	TATA-box	1203	1209	core promoter element around -30 of transcription start
CsDof103	TATA-box	1205	1209	core promoter element around -30 of transcription start
CsDof103	TATA-box	1215	1221	core promoter element around -30 of transcription start
CsDof103	TATA-box	1216	1220	core promoter element around -30 of transcription start
CsDof103	TATA-box	1319	1325	core promoter element around -30 of transcription start
CsDof103	TATA-box	1320	1324	core promoter element around -30 of transcription start
CsDof103	TATA-box	1328	1334	core promoter element around -30 of transcription start
CsDof103	TATA-box	1329	1335	core promoter element around -30 of transcription start
CsDof103	TATA-box	1330	1336	core promoter element around -30 of transcription start
CsDof103	TATA-box	1331	1335	core promoter element around -30 of transcription start
CsDof103	TATA-box	1367	1371	core promoter element around -30 of transcription start
CsDof103	TATA-box	1382	1388	core promoter element around -30 of transcription start
CsDof103	TATA-box	1383	1387	core promoter element around -30 of transcription start

Name	Cis-element	Start position	Stop position	Function
CsDof103	TATA-box	1457	1464	core promoter element around -30 of transcription start
CsDof103	TATA-box	1516	1522	core promoter element around -30 of transcription start
CsDof103	TATA-box	1517	1521	core promoter element around -30 of transcription start
CsDof103	TATA-box	1543	1551	core promoter element around -30 of transcription start
CsDof103	TATA-box	1549	1557	core promoter element around -30 of transcription start
CsDof103	TATA-box	1639	1645	core promoter element around -30 of transcription start
CsDof103	TATA-box	1640	1646	core promoter element around -30 of transcription start
CsDof103	TATA-box	1641	1647	core promoter element around -30 of transcription start
CsDof103	TATA-box	1642	1648	core promoter element around -30 of transcription start
CsDof103	TATA-box	1644	1648	core promoter element around -30 of transcription start
CsDof103	TATA-box	1656	1665	core promoter element around -30 of transcription start
CsDof103	TATA-box	1657	1663	core promoter element around -30 of transcription start
CsDof103	TATA-box	1658	1664	core promoter element around -30 of transcription start
CsDof103	TATA-box	1659	1665	core promoter element around -30 of transcription start
CsDof103	TATA-box	1660	1666	core promoter element around -30 of transcription start
CsDof103	TATA-box	1661	1667	core promoter element around -30 of transcription start
CsDof103	TATA-box	1662	1668	core promoter element around -30 of transcription start
CsDof103	TATA-box	1663	1669	core promoter element around -30 of transcription start
CsDof103	TATA-box	1664	1670	core promoter element around -30 of transcription start
CsDof103	TATA-box	1665	1671	core promoter element around -30 of transcription start
CsDof103	TATA-box	1666	1672	core promoter element around -30 of transcription start
CsDof103	TATA-box	1667	1673	core promoter element around -30 of transcription start
CsDof103	TATA-box	1668	1674	core promoter element around -30 of transcription start
CsDof103	TATA-box	1669	1675	core promoter element around -30 of transcription start
CsDof103	TATA-box	1670	1676	core promoter element around -30 of transcription start
CsDof103	TATA-box	1671	1677	core promoter element around -30 of transcription start
CsDof103	TATA-box	1672	1678	core promoter element around -30 of transcription start
CsDof103	TATA-box	1673	1679	core promoter element around -30 of transcription start
CsDof103	TATA-box	1674	1680	core promoter element around -30 of transcription start
CsDof103	TATA-box	1675	1681	core promoter element around -30 of transcription start
CsDof103	TATA-box	1676	1682	core promoter element around -30 of transcription start
CsDof103	TATA-box	1677	1683	core promoter element around -30 of transcription start
CsDof103	TATA-box	1678	1684	core promoter element around -30 of transcription start
CsDof103	TATA-box	1679	1685	core promoter element around -30 of transcription start
CsDof103	TATA-box	1680	1686	core promoter element around -30 of transcription start
CsDof103	TATA-box	1681	1687	core promoter element around -30 of transcription start
CsDof103	TATA-box	1682	1688	core promoter element around -30 of transcription start
CsDof103	TATA-box	1683	1689	core promoter element around -30 of transcription start
CsDof103	TATA-box	1684	1690	core promoter element around -30 of transcription start
CsDof103	TATA-box	1685	1691	core promoter element around -30 of transcription start
CsDof103	TATA-box	1686	1692	core promoter element around -30 of transcription start
CsDof103	TATA-box	1687	1693	core promoter element around -30 of transcription start
CsDof103	TATA-box	1688	1694	core promoter element around -30 of transcription start
CsDof103	TATA-box	1689	1695	core promoter element around -30 of transcription start
CsDof103	TATA-box	1690	1696	core promoter element around -30 of transcription start
CsDof103	TATA-box	1691	1697	core promoter element around -30 of transcription start
CsDof103	TATA-box	1692	1696	core promoter element around -30 of transcription start
CsDof103	TATA-box	1751	1755	core promoter element around -30 of transcription start
CsDof103	TATA-box	1763	1769	core promoter element around -30 of transcription start
CsDof103	TATA-box	1764	1768	core promoter element around -30 of transcription start
CsDof103	TATA-box	1787	1793	core promoter element around -30 of transcription start

Name	Cis-element	Start position	Stop position	Function
CsDof103	TATA-box	1788	1794	core promoter element around -30 of transcription start
CsDof103	TATA-box	1789	1795	core promoter element around -30 of transcription start
CsDof103	TATA-box	1790	1796	core promoter element around -30 of transcription start
CsDof103	TATA-box	1791	1795	core promoter element around -30 of transcription start
CsDof103	TATA-box	1803	1809	core promoter element around -30 of transcription start
CsDof103	TATA-box	1805	1811	core promoter element around -30 of transcription start
CsDof103	TATA-box	1806	1812	core promoter element around -30 of transcription start
CsDof103	TATA-box	1807	1813	core promoter element around -30 of transcription start
CsDof103	TATA-box	1808	1814	core promoter element around -30 of transcription start
CsDof103	TATA-box	1809	1813	core promoter element around -30 of transcription start
CsDof103	TATA-box	1890	1896	core promoter element around -30 of transcription start
CsDof103	TATA-box	1891	1895	core promoter element around -30 of transcription start
CsDof103	AT-rich sequence	495	504	element for maximal elicitor-mediated activation (2copies)
CsDof103	P-box	1058	1065	gibberellin-responsive element
CsDof103	GT1-motif	968	975	light responsive element
CsDof103	MBS	312	318	MYB binding site involved in drought-inducibility
