# Supplementary material for: Genome-wide identification and functional analysis of Dof transcription factor family in Camelina sativa
Source: BMC Genomics. 2022 Dec 8;23:812. doi: 10.1186/s12864-022-09056-9 (PMC9730592; doi:10.1186/s12864-022-09056-9)
Supplement: Supplementary file 9 — Additional file 9: Table S7. Expression levels (FPKM values) of 103 CsDof genes across 12 different tissue samples. [file 12864_2022_9056_MOESM9_ESM.pdf]

Table S7. Expression levels (FPKM values) of 103 CsDof genes across 12 different tissue samples.

Name	*CsDof1*	*CsDof2*	*CsDof3*	*CsDof4*	*CsDof5*	*CsDof6*	*CsDof7*	*CsDof8*	*CsDof9*	*CsDof10*	*CsDof11*	*CsDof12*	*CsDof13*	*CsDof14*	*CsDof15*	*CsDof16*	*CsDof17*	*CsDof18*	*CsDof19*	*CsDof20*	*CsDof21*	*CsDof22*
**C_r1**	0.618335	2.91878	0.924871	1.38355	6.61165	3.35082	2.5508	1.37185	0	4.05686	0.27675	0	0.866932	0.469684	3.22966	0.846489	2.32094	0	0	0.529854	0.104717	1.9571
**C_r2**	0.412242	1.65013	0.438673	1.12343	7.78906	2.03219	1.99353	1.36658	0	3.60898	0.184374	0.11406	2.8886	0	2.89713	1.35016	2.05326	0	0	0.421957	0.208476	1.01025
**C_r3**	0	1.85437	0.612278	1.06816	5.87899	2.53112	1.73777	2.03414	0	3.59169	1.66283	0	1.60238	0	3.62745	1.56802	1.09867	0	0.205399	0.589577	0.145653	0.655294
**EMSD_r1**	0	0.347758	0.423802	0.245907	2.81342	0.466545	0.240127	4.61754	0	1.1887	0.436245	0	3.18402	0	2.8388	0.540616	1.40186	0	0	2.21641	2.19051	1.39897
**EMSD_r2**	0.863824	1.06659	0.0930146	0.216238	3.02695	0	0.844386	12.2626	0.105235	1.04039	1.35511	0	3.03048	0	1.81656	0.356964	1.50012	0	0	0.597515	0.811905	0.996103
**EMSD_r3**	0	0.734634	0.513893	0.748473	1.74507	0	0.730376	5.33756	0	1.25182	0	0	3.4075	0.229926	3.46123	0.82514	1.03193	0.15016	0	1.64581	2.13439	0.316677
**ESD_r1**	1.15314	4.49383	0.244378	0.14231	5.43974	0	0.138879	3.42627	0	1.53199	1.80366	0	2.42282	0	4.78539	1.09786	0.803588	0.428252	0	1.66402	0.773757	3.11374
**ESD_r2**	0	2.47932	0.55437	0.486471	3.02969	0.153357	0.316248	3.02587	0.315268	1.62739	2.41242	0.18187	1.26364	0	3.07227	2.51028	1.90991	0	0	2.42909	0.981645	2.60743
**ESD_r3**	0.548174	2.23031	0.246109	0	1.37312	0	1.39035	3.062	0	0.694899	1.98022	0	1.0304	0.424975	5.07101	4.05651	0.909878	0	0	1.98878	0.393191	3.46614
**F_r1**	0	3.27078	0.332654	2.31928	2.54249	0.733015	4.15114	1.03587	0	2.55961	2.0725	0.431069	2.88334	1.17535	6.89966	4.67836	0.731984	1.16318	0	1.60334	3.69712	4.93435
**F_r2**	0.355474	4.74895	0.152643	2.83994	4.39221	0.504752	1.03959	1.26774	0.17275	0.853083	1.59239	0	2.32707	0.811674	5.21955	2.73549	2.23663	0.8902	0	1.34766	1.81603	4.27356
**F_r3**	1.07102	4.07526	0.787183	2.18961	5.18843	0.34635	1.0693	0.65327	0.44425	2.54522	1.44625	0.101524	5.35792	0.961608	5.35939	3.90758	2.1455	0.823559	0	2.34711	3.76193	5.20427
**GS_r1**	0	0.649713	0.220705	0.205538	0.170405	0	0.1003	0.641719	0.449998	0.8309	0.278327	0.401715	0.96903	0	0.107974	0.962366	0.516497	0	0.298207	0.636866	0.41954	0.762393
**GS_r2**	0.0937665	0.918615	0.673271	0.145986	0.195704	0.0231108	0.0475463	0.698058	1.04303	0.891006	0.169348	0.162157	1.05746	0.0363401	0.102012	0.426857	0.528889	0	0	0.323015	0.319307	0.453166
**GS_r3**	0	0	0	0	0.884632	0	0	0.957562	0	0.966561	0	0	0	0.817401	0	0	0.337886	0	0	1.11048	0	1.13929
**IF_r1**	0.580285	3.20009	0.979463	5.13613	7.60657	0.540527	3.0628	0.763008	1.11039	0.851965	1.03645	0	0.541598	0.438433	2.09931	3.46049	1.25142	1.14491	0	2.15634	1.93694	5.83396
**IF_r2**	2.84687	2.48644	0.911779	4.95112	5.85257	0.670291	2.41626	0.315574	2.40905	0.84822	0.636999	0	3.32589	0	3.34356	2.72648	0.444839	0	0	2.43738	2.40852	3.75124
**IF_r3**	1.50388	1.84682	1.2893	5.74825	4.33343	0.236887	2.92748	0.892375	1.21615	0.750282	1.34693	0	1.87487	0.190632	3.54373	2.33889	0.944253	0.501381	0	1.98289	3.23738	3.98032
**LSD_r1**	0	0	0.665003	0.465098	0.517361	0	0.151274	0.138154	3.46879	0.462486	0.28207	0	0	0	0	0.170963	0.388214	0.466547	0	0	0.105165	0.218461
**LSD_r2**	0	0	1.01175	0.169175	0.28787	0.319942	0.164957	0.300563	3.94666	0.498827	0	0	0	0	0	0	0.523841	1.69717	0	0	0.22739	0
**LSD_r3**	0	0.225565	0.829321	0.322431	0.809324	0	0	0.143597	6.27253	0.384077	0	0	0	0	0	0.355721	0.806094	0.808601	0	0	0	0.340327
**LMSD_r1**	0.577619	0.719905	0.752522	0.582558	2.62598	0	0.711004	0.780984	2.69387	0.175687	1.55568	0	0.270438	0	0	0.48049	1.10517	0.146081	0	0.504174	0.498265	0.413729
**LMSD_r2**	1.01763	0.42842	0.436174	0.608768	1.8431	0	0.297116	0.362273	0.987438	0.609163	1.45826	0	0.951486	0	1.06566	0.670253	0.894427	0.101772	0	0.489982	0.622535	1.00542
**LMSD_r3**	0	0.482285	0.589049	0.513705	2.82447	0	0.835767	0.611523	1.1666	0.205764	0.92158	0	0.641309	0	0.179835	0.188495	0.755263	0.171759	0	0.591001	0.817639	0.727597
**OL_r1**	0	0.742031	12.0139	0.263687	6.10091	1.4995	0	1.41209	0	10.4441	0	0	1.98021	0	4.43154	0	3.98323	0	0	1.45482	0.179702	0.373159
**OL_r2**	0.256938	0.527579	6.89552	0	2.10089	1.30871	0	1.11929	0	5.39062	0.458389	0	4.31345	0	1.58514	0	2.67121	0	0	1.55117	0.34058	0.619109
**OL_r3**	0	1.76406	12.9663	0.157477	3.94582	2.83339	0	1.54338	0	5.82132	0.286913	0	1.49974	0	3.97312	0	2.56158	0	0	1.29669	0.213531	0.665389
**R_r1**	1.31357	2.56677	13.8225	8.14555	4.42189	1.13395	5.29763	1.63529	0.233007	1.18715	1.90187	0	4.28482	1.11064	15.0322	0.793259	8.17329	0.961311	0	4.70269	3.02545	10.3835
**R_r2**	1.45864	2.05024	16.6042	6.75363	5.65846	0.67304	3.988	1.02874	0.518592	1.53361	0.65011	0	4.75937	0.685858	15.9781	1.07865	8.10275	1.24799	0	4.9927	3.06798	7.87408
**R_r3**	0.173649	2.35108	16.7396	3.69223	3.56371	0.488113	3.26818	0.918972	0.334192	0.976363	0.310522	0	2.27068	0.263106	18.4957	1.23	4.63598	1.29197	0	4.31362	2.39394	6.36658
**S_r1**	0.189602	12.4593	1.67749	5.58612	7.76293	1.05811	4.99655	0	0.271716	2.66718	1.01565	0	6.19253	0.715787	7.04591	2.66916	3.2066	1.68105	0	4.41149	4.80105	5.77391
**S_r2**	1.0507	9.0337	3.31883	4.64198	6.02203	1.2212	4.27786	0.804388	0.125439	2.00066	0.938	0.14416	3.67664	0.198302	6.23465	2.13279	3.39099	0.776073	0	4.07283	4.46163	8.26862
**S_r3**	0.225235	13.8577	2.34718	7.01229	6.61756	1.76325	5.77202	0.390207	0.213127	2.5411	0.802762	0	5.24803	0.677177	7.7197	1.81391	3.35074	1.09911	0	5.25209	4.52187	2.61839
**YL_r1**	0.347045	2.03335	0.146437	2.90094	3.9805	0.323253	3.49681	2.12942	0	1.22279	1.23972	0	1.29563	0.262205	2.15223	3.38646	0.641471	0.171174	0.332381	2.2274	0.57917	0.721834
**YL_r2**	0.340104	0.949376	0	1.35069	4.19369	1.59994	3.79014	0.753213	0	3.13717	2.13037	0.188518	2.22478	0.258141	2.83791	2.60354	0.636889	0	0	2.21025	0.574753	1.07423
**YL_r3**	0.311061	1.83505	0.462173	2.15271	3.31918	0.801236	3.30139	1.8513	0	2.20826	1.39052	0	0.871576	0.353427	2.34276	1.52494	0.530874	0.15424	0.149025	2.27484	0.731847	1.03142

Name	*CsDof23*	*CsDof24*	*CsDof25*	*CsDof26*	*CsDof27*	*CsDof28*	*CsDof29*	*CsDof30*	*CsDof31*	*CsDof32*	*CsDof33*	*CsDof34*	*CsDof35*	*CsDof36*	*CsDof37*	*CsDof38*	*CsDof39*	*CsDof40*	*CsDof41*	*CsDof42*	*CsDof43*	*CsDof44*
**C_r1**	0.511221	0.592923	3.68238	9.38113	2.7807	1.00994	3.14338	2.13948	0.554811	0	0.984244	0.25528	1.12296	0	4.17317	5.10377	0	0.953737	1.53652	2.20584	0.72795	0.957674
**C_r2**	0.621695	0.236159	3.68364	10.4683	1.49053	0.595848	3.56495	2.02815	0.27635	0	1.1613	0.0847484	0.541945	0.106726	3.844	6.59579	0	0.140652	1.83531	1.80429	0.345052	1.63541
**C_r3**	0.395181	0.439735	4.43627	11.714	1.78505	1.14446	2.0973	1.65334	0.0642685	0	0.912597	0.207041	1.11236	0.185987	4.3377	6.29571	0.223395	0.611432	1.47235	2.16087	0.313405	1.14109
**EMSD_r1**	0.549706	0.190497	0.626764	3.34417	3.26892	0.180404	0.58935	1.14795	0.8891	0	0.703498	0.20482	0.985935	1.02689	0.998144	0.88429	0	2.21641	2.638	1.35453	0	1.53505
**EMSD_r2**	1.02183	1.42006	0.684252	3.48586	2.78892	1.02789	0.389602	1.65184	1.26902	0	0.693601	0.359503	0.719647	0.6776	1.30891	0.582501	1.98935	0.253631	3.10497	1.3118	0	0.674056
**EMSD_r3**	1.15627	0.92163	0.938049	3.04026	4.15282	0.762791	0.360859	2.07617	1.34929	0	0.424709	0.868531	0.594408	0.782398	1.57231	1.61016	0	2.88017	2.78341	0.827023	0	0.399215
**ESD_r1**	0.864883	0.657592	1.72721	5.54234	3.16137	0.414727	1.54278	3.01079	1.53974	0	2.3237	0.47207	0.659957	1.93361	2.85155	3.57284	0	1.1746	3.02727	1.57194	0.288143	1.64516
**ESD_r2**	1.6778	0.867697	2.19797	5.05447	3.23239	1.05397	0.786539	0.636239	1.4574	0	1.14044	0.133734	0.212523	3.39465	1.83846	2.31856	1.87358	0.560787	3.09235	1.2581	0.43319	2.44259
**ESD_r3**	1.12605	0.221746	2.33135	6.55628	3.61187	1.26132	1.69654	0.574278	3.35241	0	4.30583	0	0.191804	5.04517	2.19364	1.53909	0	1.19327	3.27144	0.938128	0.585831	1.01799
**F_r1**	6.23851	3.28686	3.42952	6.23353	5.66321	2.26292	3.71266	2.31273	3.83988	0	2.75714	2.25015	2.05992	2.01858	6.05153	5.55481	3.12598	0	2.06689	3.8363	2.36039	2.75486
**F_r2**	5.81509	2.32986	2.4675	7.22568	4.8221	4.1508	3.62634	2.47376	2.243	0	2.27559	1.03236	2.00664	2.22506	3.21896	6.05477	1.98371	0	1.30282	1.76248	1.20234	3.63484
**F_r3**	3.89093	2.40803	4.83155	5.51337	6.08097	4.56264	3.71028	2.25817	4.37461	0	2.28985	0.989406	2.62961	2.85622	5.20833	5.50049	0.582827	2.90552	2.02482	2.2091	1.1832	3.09478
**GS_r1**	0.710843	0.356443	1.4222	4.57037	0.928441	0.56208	0.309257	1.25846	0.0463459	0.0982329	1.20503	0.341106	1.09062	1.07395	2.88792	1.79751	0	0.884536	0.34198	1.1915	0.208316	0.594251
**GS_r2**	0.412518	0.625557	1.43685	2.92701	0.548901	0.305501	0.203078	0.687272	0.0440924	0.0892279	0.736162	0.244218	0.85259	0.888052	3.2001	1.24999	0	0.510024	0.427704	0.882284	0.166929	0.413385
**GS_r3**	1.78675	0	1.35553	3.18371	0.747145	0.391908	0	1.06774	0	0	2.2918	0.445587	0.356635	1.12042	2.16129	1.92562	0	1.11048	0.357846	0.591657	0.363273	0.47748
**IF_r1**	4.40672	3.73254	3.45555	8.69188	2.17642	4.77653	3.78371	0.753635	1.28589	0	1.82115	1.1824	2.26554	0.298283	4.56539	10.23	0	1.17619	1.89443	1.26123	1.15407	3.55089
**IF_r2**	5.29079	3.00041	3.26841	7.4928	1.47595	3.87162	5.10518	1.17162	2.87161	0	0.251538	0.880817	1.64361	1.10857	3.19911	7.29815	0	3.89981	0.706805	1.95188	0.956746	2.5177
**IF_r3**	7.14225	2.7007	2.62977	10.1718	2.69724	3.37748	3.75562	0.497342	1.35334	0.237536	1.7793	2.07606	2.57482	0.783302	3.39677	7.4025	0	2.24153	2.00019	2.75783	1.52291	2.66984
**LSD_r1**	0.0854261	0	0.0646585	7.60594	0.214857	0	0.186974	1.02291	6.42625	0	0.109872	0	0.717509	0.648323	0.154861	3.19524	0	0	0.205707	0.342709	0	0
**LSD_r2**	0	0.258484	0.346907	7.83536	0.232382	0	0.205259	0.773706	6.68834	0	0.356664	0.278916	0.110761	0.3542	0.666259	2.87165	0	0.460385	0.222297	0.375119	0	0.149856
**LSD_r3**	0	0	0.201206	9.81189	0.334695	0.117128	0.389207	1.06215	6.09885	0	0.228221	0	0.106434	0.674311	0.321381	2.88798	0	0.221062	0.320401	1.06962	0.108444	0.143199
**LMSD_r1**	0.731058	0.676242	1.049	6.43256	2.44218	1.06716	1.22276	0.775829	1.31586	0.274027	0.416111	0.72728	0.680219	0	0.884752	2.48655	0.635369	0	1.85254	0.160519	0.0989706	0.519253
**LMSD_r2**	0.957093	0.313251	0.597831	5.41979	2.47251	0.963491	0.4879	1.2787	0.640976	0	0.505627	0.252841	0.337184	0.105999	0.970356	3.64262	0	1.67994	1.42098	0.783721	0.0686932	0.180643
**LMSD_r3**	0.570556	1.32222	1.29858	7.26146	1.19289	0.250287	0	1.02285	2.47305	0	0.365908	0.426848	1.13881	0	1.03524	3.07437	0.956942	0	2.17109	0.944592	0.232001	0.152465
**OL_r1**	0.584972	0.406949	5.5453	6.92976	18.5367	0	0	3.49678	5.71079	0	1.50131	0.437988	0.700779	0.826484	3.05106	1.89314	0	5.81928	2.98844	10.4763	0.178461	0
**OL_r2**	0.760499	0.0965626	7.79372	5.84258	9.74194	0.365259	0	1.90471	1.35823	0	0.711716	0.104036	0.331936	0.131377	4.44853	1.23809	2.25238	1.54278	1.99847	5.97359	0.0845499	0.223239
**OL_r3**	0.606955	0.484265	6.43017	9.58827	11.7791	0.686963	0	1.34998	2.69573	0	1.00395	0.391275	1.14464	0.493948	4.32221	3.66833	0.968917	3.3518	2.71482	9.92298	0	0.139919
**R_r1**	11.2666	2.81977	4.97168	46.6841	3.97407	8.5774	1.44716	10.5083	10.1381	0	1.97604	1.6516	13.7943	6.9322	5.64008	19.1615	0	2.46071	3.11708	25.6196	3.37976	1.48894
**R_r2**	12.0112	2.525	5.2064	49.9368	5.03947	11.2275	1.28775	12.4032	7.36231	0	1.25722	0.956007	12.722	7.71321	5.57446	21.8041	0.537406	1.34996	2.52964	31.1563	1.79206	1.18372
**R_r3**	9.59007	2.38154	3.70498	38.1759	5.12894	7.26006	1.24074	7.49777	6.73662	0	2.19557	1.56703	15.8799	6.55091	4.5632	18.7121	0.528171	1.71698	2.28447	28.2594	1.15967	1.52742
**S_r1**	9.5969	4.86995	7.61429	31.6623	4.77526	3.04812	2.02075	3.31811	10.5716	0	5.4118	1.69712	4.06379	4.67188	7.86171	20.1078	1.67601	0.756001	3.76882	5.7621	1.56829	1.57215
**S_r2**	7.67533	5.15741	5.1105	45.3766	5.36191	2.62655	2.1769	2.63789	8.25009	0	3.93008	1.81618	2.72858	3.90928	3.9295	36.9766	0	2.92181	2.82351	8.12313	1.21622	1.48974
**S_r3**	8.3613	5.88753	6.54913	32.3969	6.13441	3.1808	3.04436	2.23487	12.9234	0	3.64091	1.6316	3.25086	2.97935	6.70354	30.1163	0.65991	1.89113	3.55205	5.45428	1.25136	1.75105
**YL_r1**	0.376474	0.65652	5.84317	4.18729	1.06491	1.11776	2.67423	0.112673	0.461402	0	2.17819	0.707111	0.790331	0.178384	7.33742	1.83535	0	0.117232	0	1.31997	1.03525	2.27523
**YL_r2**	0.56107	0.781155	5.81276	5.57953	1.17405	2.21746	4.87546	0	0.913871	0	2.28103	1.26133	1.12061	0.176421	8.90501	2.57503	0	0.465315	0.562216	0.931939	0.570776	3.45531
**YL_r3**	0.850053	1.06611	6.02168	4.55736	0.907701	1.62499	3.333	0.0508532	0.346646	0	2.62092	0	2.29307	0.241053	7.43805	1.6547	0	1.00516	0.409051	2.2077	0.051912	2.05135

Name	*CsDof45*	*CsDof46*	*CsDof47*	*CsDof48*	*CsDof49*	*CsDof50*	*CsDof51*	*CsDof52*	*CsDof53*	*CsDof54*	*CsDof55*	*CsDof56*	*CsDof57*	*CsDof58*	*CsDof59*	*CsDof60*	*CsDof61*	*CsDof62*	*CsDof63*	*CsDof64*	*CsDof65*	*CsDof66*
**C_r1**	4.34786	0.291215	0	0.413968	2.22329	0	1.43036	0	0	0	0	0.722243	0	0	0	0.198386	2.06564	6.61315	0.188142	1.19812	0	0.500788
**C_r2**	2.60391	0.388149	0	0.618096	1.07294	0	2.1511	0	0	0.121592	0	0.58548	0	0	0	0	2.05819	6.85126	0.875636	3.10194	0	0.914337
**C_r3**	3.22037	0	0	1.17562	1.45261	0	1.26025	0	0	0.0424982	0	0.40946	0	0	0	0.229973	2.42659	6.9111	0.958075	3.30432	0	0.986423
**EMSD_r1**	1.19428	1.37478	0	1.499	2.76568	2.36498	1.38253	0	0.689647	3.84	0.814517	0.777081	0	0.436245	1.49676	4.63186	0.236194	0.730105	0	0.769762	0	5.62683
**EMSD_r2**	1.49483	2.44291	0	1.31311	4.63078	2.44377	1.61377	0	0.914033	4.07167	1.26328	0.339752	0	0	2.11134	3.77639	0	0.511146	0	1.01272	0	8.11117
**EMSD_r3**	1.95579	1.431	0	1.50661	3.62833	1.95405	1.29494	0	1.69929	5.50979	0.252773	1.01092	0	0	2.01736	4.13835	0	0.878447	0	0.698436	0	3.28539
**ESD_r1**	1.27339	0.813947	0	2.58074	4.94623	4.63507	2.46503	4.44805	1.81504	2.62229	4.07646	3.55377	3.8867	3.09198	5.92373	3.84696	0.68274	4.01367	0	0.664439	0	2.66214
**ESD_r2**	0.552466	0.637097	0	1.93972	6.73042	7.00927	3.07165	5.44951	1.63071	4.28094	3.35354	1.91052	3.94356	2.11087	5.59958	3.81736	0.932321	1.69233	0.200247	1.00224	0	3.27729
**ESD_r3**	0.995069	0	0	2.52674	5.69783	13.9262	3.41065	1.36029	4.34499	3.62658	0.463534	2.27593	4.47834	0.495055	10.3358	3.54391	2.18903	2.90309	0	0	0	3.03768
**F_r1**	3.20892	2.90529	0	1.30505	3.82366	0	4.57162	0	0	2.31282	0	1.01336	0	0	0	2.75255	4.08254	2.05785	0.472118	2.7169	0	1.26137
**F_r2**	2.32941	1.67496	0	1.6752	2.45388	0	3.30828	0	0	2.33172	0.2968	2.59959	0	0	0	2.75279	3.40775	2.40989	0	3.1847	0	0.289334
**F_r3**	2.53917	2.52998	0	2.29324	2.54305	0.145423	4.23221	0	0	1.97998	0.750903	2.84752	0	0	0	2.19969	3.68181	3.26282	0.110972	1.717	0	0.597474
**GS_r1**	1.84113	0	0.0994022	0.863795	0.438587	0	0.668427	0	0	1.43755	0	0.348105	0	0	0	0.894054	0.345176	0.181413	0.251756	0.040016	0	0.209098
**GS_r2**	0.850622	0	0.045099	1.24612	0.925424	0.230494	0.613782	0	0	0.900483	0	0.168603	0	0	0	0.63777	0.0935731	0.23356	0.442387	0	0	0.159806
**GS_r3**	0.740844	0	0	0	0.706103	0	0.333186	0	0	1.28092	0	0	0	0	0	1.03965	0	0.633149	0	0.418351	0	0.437087
**IF_r1**	1.56936	1.63764	0	1.5313	2.24255	2.79474	3.5253	0	0	2.54022	0.964097	2.22269	0	0	0	1.65064	4.65367	5.02194	0.350077	3.54966	0	1.15956
**IF_r2**	0.975639	2.68134	0.682236	0.952137	1.62704	0	2.41248	0	0	2.95068	0.593289	3.69114	0	0	0	2.50956	2.37595	2.91691	0.432887	2.75591	0	0.863948
**IF_r3**	1.38037	1.18082	0	2.02079	1.72669	1.61815	3.64683	0	0	2.61002	0.20917	1.95947	0	0	0	1.85628	4.43814	3.90716	0.305693	2.72861	0	1.12002
**LSD_r1**	0.745579	0.891555	0	0.415696	0.304379	0.253427	0.765513	0	0	6.15927	0	0.16085	0	0	0	3.98282	0	0.817825	0.1903	0	0	0.377858
**LSD_r2**	0.230359	2.99855	0	0.786295	0.438456	0	1.03269	0	0	5.25455	0	0	0	0	0	4.30141	0.324188	0.68586	0	0.130624	0	0.956878
**LSD_r3**	0.884879	1.86158	0	0.215831	0.842791	0	0.596048	0	0	5.15312	0	0.166919	0	0	0	4.34204	0.154624	0.848926	0	0	0	0.39261
**LMSD_r1**	0.706336	0.272477	0	1.28048	0.673428	0	1.27154	0	0	2.53207	0	0.459242	0	0	0.177468	3.30554	0	1.29483	0	0.455445	0	0.11892
**LMSD_r2**	0.560374	0.191763	0	1.64071	1.80248	0	1.51195	0	0	1.33201	0	0.371212	0	0	0	2.62111	0.681739	1.79562	0	0.553854	0	0.496023
**LMSD_r3**	0.591416	0.969351	0	0.808105	1.46558	0.276872	0.851153	0	0	2.24965	0	0.358304	0	0	0	2.10257	0	1.41526	0	0.801519	0	0.279137
**OL_r1**	8.73506	6.4854	0	1.42082	2.94833	0	2.9458	0.431414	0	1.5728	0	0.964222	0	0	0	1.36186	0.253098	5.44188	1.29046	0	0	0.429614
**OL_r2**	4.39811	0.24155	0	0.336553	2.13561	0	1.93646	0	0	0.744092	0	1.36673	0	0	0	0.403032	0	2.50069	1.23472	0	0	0.61211
**OL_r3**	8.21819	0.302337	0	1.37156	1.545	0	2.33126	0	0	2.70614	0	1.30604	0	0	0	0.909719	0.151055	3.41282	0.38675	0	0	1.15109
**R_r1**	11.7651	4.62791	0	1.17447	4.01262	0	14.6416	0	0	1.46298	0	8.12851	0	0	0	0.920457	13.325	4.7579	0.58927	6.31836	0.141935	0.0647774
**R_r2**	13.1	4.28358	0	1.06999	2.84338	0	14.1712	0	0	1.52415	0	8.31844	0	0	0	0.683321	12.3573	6.28581	1.31084	5.51684	0	0
**R_r3**	6.68197	4.90381	0	0.692441	3.94392	0	16.9025	0	0	1.7365	0	11.9815	0	0	0	1.16126	10.0522	5.35164	0.84174	4.54524	0	0
**S_r1**	3.39095	4.27996	0	3.37105	4.2663	0	5.9782	0	0	1.43586	0	5.41152	0	0	0	1.13645	14.4688	5.18594	0.685557	6.15107	0	0.0756503
**S_r2**	2.74668	4.69401	0	2.67999	3.71368	0	6.20904	0	0	1.98839	0	4.48287	0	0	0	1.15963	18.0596	7.18151	1.42432	5.81264	0	0
**S_r3**	2.85317	5.71358	0	3.00324	2.50262	0	4.85397	0	0	1.35985	0	4.18964	0	0	0	0.982311	17.3366	5.44037	1.6169	6.79943	0	0
**YL_r1**	0	0	0	1.37363	0.223517	0	1.47575	0	0	1.01274	0	1.59506	0	0	0	0	1.63709	3.80411	0.418721	3.1842	0	0.970836
**YL_r2**	0	0	0	1.93128	0.221862	0	1.77933	0	0	0.804656	0	0.792628	0	0	0	0.435523	4.37499	2.58518	0	4.34089	0	0.137462
**YL_r3**	0.0529407	0.2928	0	1.49817	0.554859	0	1.76068	0	0	1.326	0	0.400238	0	0	0	0.693098	2.5083	3.11875	0.47108	3.65051	0	1.25093

Name	*CsDof67*	*CsDof68*	*CsDof69*	*CsDof70*	*CsDof71*	*CsDof72*	*CsDof73*	*CsDof74*	*CsDof75*	*CsDof76*	*CsDof77*	*CsDof78*	*CsDof79*	*CsDof80*	*CsDof81*	*CsDof82*	*CsDof83*	*CsDof84*	*CsDof85*	*CsDof86*	*CsDof87*	*CsDof88*
**C_r1**	0	0	1.43218	0	0	0	0	0	0.111138	0.207127	0.126994	3.62026	0	0	0.738711	1.85574	2.44444	0	0.637256	0.809403	1.71899	1.52197
**C_r2**	0	0	1.52035	0	0	0	0	0	0	0.0687248	0.421589	2.58658	0.10847	0	1.35055	1.37149	3.2495	0.28112	0.79819	0.359432	1.70853	1.91057
**C_r3**	0	0	1.72674	0	0	0	0	0	0.360655	0.072026	0.147139	3.57298	0.0756019	0	1.75274	1.28821	3.234	0.581637	0.640559	0.685929	1.92556	1.60542
**EMSD_r1**	0.365925	2.5164	1.23046	0	0	0.640966	0.488385	0	1.78695	0.333337	0	1.04709	0	0	1.17728	0.327767	1.39301	0.651942	0	1.27693	0.727504	0.540021
**EMSD_r2**	0.161827	2.70181	1.41384	0	0	0.282837	1.73822	0	3.44578	0.146001	0.089423	0.785566	0.229516	0.241209	2.20506	0	2.33107	0.584634	0.449848	0.943923	0.581214	0.237711
**EMSD_r3**	0.225984	2.88386	2.6858	0	0	0	0.612279	0	2.26623	0	0.123442	1.7105	0.159054	0.167313	1.62185	0	2.72416	0.833834	0.685695	0.264774	0.397196	0
**ESD_r1**	0.214497	4.84328	2.46818	1.4872	3.18354	16.2644	2.61089	6.95696	3.49115	0.0956491	0	0.9426	0	0	1.54087	0.191345	3.88301	1.97141	0.391779	0.753342	0.963432	0.469978
**ESD_r2**	0.248513	6.58124	2.97643	4.25107	7.14353	10.3304	2.73691	1.50776	2.55091	0.539192	0.133034	1.6387	0.172612	0	0.785527	0.441005	4.44434	4.29007	0.0731156	3.22777	0.61221	0.179095
**ESD_r3**	0	4.52291	2.15675	0.604059	2.83536	10.6732	5.51287	1.48517	1.66612	0.194494	0.473798	0.699489	0	0	1.01674	0	4.17737	1.44783	0	1.45064	0.643898	0
**F_r1**	0	1.70274	4.33647	0	0	0	0	0	1.12066	1.04476	0.639669	1.8741	0.410224	0	4.17167	1.03372	6.13894	0	1.96817	1.34753	1.79778	2.12416
**F_r2**	0	0.261238	3.97496	0.124083	0	0	0	0	1.54164	1.07765	0	1.18093	0	0.198054	2.55664	2.37737	3.42526	2.41112	1.80265	1.24216	0.692544	2.73243
**F_r3**	0.135148	0.53121	4.35201	0.0642342	0	0	0	0	1.46226	1.11638	0.302926	1.4482	0.483557	0.203048	4.14186	3.63688	4.74886	2.85904	1.91957	3.13703	1.42702	2.50213
**GS_r1**	0.154627	0	0.828629	0	0	0	0	0	1.37311	0.103727	0.424216	0.557702	0.163725	0.114776	0.7413	0.0690075	0	0.141501	0.685012	1.35647	0.647745	1.01783
**GS_r2**	0.357656	0	0.506996	0	0	0	0	0	0.60532	0.349146	0.202521	0.50824	0	0.459446	0.202841	0.83617	0.0549475	0.49546	0.734366	1.07515	0.525869	1.1726
**GS_r3**	0	0	2.00165	0	0	0	0	0	1.55262	0	0.443338	0.648737	0	1.19675	0.643687	0	0	0	0.495142	1.87635	0.784689	0
**IF_r1**	0	1.26897	2.64735	0	0	0	0	0	0.822659	1.7239	0.470542	1.88713	0.606388	0	1.03064	1.15248	2.272	2.38782	4.05033	0	1.995	2.19963
**IF_r2**	0.531311	1.56508	3.51352	0	0	0	0	0	2.55628	0	0.292118	1.06748	0	0	1.27473	0.474458	2.81199	0	1.954	1.24197	1.20297	4.27965
**IF_r3**	0.187427	0.736203	2.87455	0	0	0	0	0	0.904082	1.43238	0.413113	0.830903	0.928685	0	0.300082	0.837299	2.83761	0	2.70905	1.31327	1.27839	3.4357
**LSD_r1**	0.234386	0	0.479058	0	0	0	0	0	4.69033	0	0.766678	0	0	0.173338	0	0	0	0	0.0709044	0.274822	0.0746301	1.53691
**LSD_r2**	2.33666	0	0.413614	0	0	0	0	0	2.78022	0.112407	0	0	0	0.379603	0	0	0	0.499845	0.152342	1.84068	0.318719	1.86897
**LSD_r3**	0.244273	0	0.497344	0	0	0	0	0	2.66774	0	0.132775	0.193356	0	0.721372	0	0	0	0	0.07356	0.286738	0.23206	2.13188
**LMSD_r1**	0.86881	0	0.636568	0	0	0	0	0	2.11403	0	0.120607	0.530608	0.154513	0.162322	0.697876	0	0.495308	0	0.473146	0.252929	0.250482	0.479958
**LMSD_r2**	1.06529	0	1.00925	0	0	0	0	0	1.6883	0.06841	0.167709	0.797279	0.646318	0	1.21826	0.13597	0.115199	0	0.327583	0.177726	0.494243	0.669502
**LMSD_r3**	0	0	0.426115	0	0	0	0	0	2.10707	0.462091	0.424693	0.621471	0.181777	0.382126	0	0	0	0.465004	0.869625	0	0.292345	1.88284
**OL_r1**	0	0	3.1134	0	0	0	0	0	1.52568	0	4.79349	6.6913	0	0	0.316685	1.06053	6.28818	2.15926	0.121548	5.08514	2.11759	0
**OL_r2**	0	0	1.93894	0	0	0	0	0	0.813847	0.0841969	0.517511	3.31685	0	0	3.02883	0.169475	3.57567	0.707644	0	2.23272	2.65497	0
**OL_r3**	0	0	1.9452	0	0	0	0	0	1.02044	0.211156	4.02245	3.30873	0	0	2.27701	0.424601	6.27285	0.884617	0.143914	2.23615	3.14199	0.520472
**R_r1**	0	0	12.4958	0	0	0	0	0	1.37736	0.480799	6.24374	1.00406	0.169822	0	6.07043	2.91357	13.0997	0.681061	6.07185	8.40648	4.16381	7.57131
**R_r2**	0	0	17.9148	0	0	0	0	0	0.830665	0.832793	7.38921	1.22466	0.188949	0	5.78759	4.19983	14.1681	1.51023	8.01939	10.4481	4.49132	9.01105
**R_r3**	0.129314	0	20.8091	0	0	0	0	0	1.42562	0.577433	8.36221	1.08649	0.456038	0	1.75554	2.30774	15.9086	1.42322	7.41345	11.652	3.0349	6.89911
**S_r1**	0	0	7.48231	0	0	0	0	0	0.603718	0.624702	0.76747	2.07001	0.197871	0.104085	2.91523	1.00323	6.99086	5.7271	1.10759	5.27784	3.43074	6.76783
**S_r2**	0	0	6.29669	0	0	0	0	0	1.20765	0.69209	1.38173	1.93687	0	0	4.03778	1.3896	6.16164	3.96807	1.00283	5.48353	2.82541	12.4981
**S_r3**	0	0	5.87275	0	0	0	0	0	0.708701	0.806309	0.721416	1.96813	0	0.122622	4.75908	1.18435	4.49313	7.78464	1.09757	3.71372	3.66932	9.42145
**YL_r1**	0	0	1.26655	0	0	0	0	0	0	0.343646	0	2.05191	0.362643	0	1.23273	1.14873	2.9116	0.475998	0.859482	0	2.34494	1.50338
**YL_r2**	0	0	0.838396	0	0	0	0	0	0.122007	0.454733	0.418311	2.03773	0.358594	0	1.62315	1.13292	2.87709	1.8531	1.01009	1.78005	2.13163	0.92879
**YL_r3**	0	0	1.14351	0	0	0	0	0	0.166481	0.413574	0.190346	2.77913	0	0	0.83222	0.51663	1.57305	2.97506	0.529525	0.135525	1.4328	0.761609

Name	*CsDof89*	*CsDof90*	*CsDof91*	*CsDof92*	*CsDof93*	*CsDof94*	*CsDof95*	*CsDof96*	*CsDof97*	*CsDof98*	*CsDof99*	*CsDof100*	*CsDof101*	*CsDof102*	*CsDof103*
**C_r1**	0.549742	4.53493	0	0	1.88142	0	2.25133	1.24413	7.84736	1.80057	2.44593	0.63766	0	0.219748	0.724943
**C_r2**	0.547635	4.17206	0	0	1.12582	0.142445	2.12375	2.48499	8.30435	1.79418	1.77823	0.677411	0	0	0.206174
**C_r3**	0.382097	4.5876	0	0	2.17744	0.0495379	2.44411	1.72594	7.2274	1.39021	2.05163	0.768359	0	0	0.072026
**EMSD_r1**	1.9825	1.49291	0	0.214684	0.899154	0	2.09384	0.393531	2.04734	0	1.68437	5.52552	0	0.697495	0
**EMSD_r2**	1.16077	1.30773	0	0.094249	1.05698	0	2.00322	0.348539	1.71906	0.105548	1.3949	8.08199	0	0.154102	0.0730003
**EMSD_r3**	1.60422	1.80045	0	0.130204	0.550885	0	2.99292	0.731538	2.05872	0	1.01823	6.81834	0	1.28958	0.10051
**ESD_r1**	1.01706	1.04707	0.287281	0	1.22113	0	5.14706	1.85093	5.77922	0	0	4.95251	0	1.83647	0
**ESD_r2**	0.577446	2.36077	0	0	1.40173	0	2.47712	1.88378	3.42529	0.474372	0.486733	2.53872	0.489069	1.65087	0.215677
**ESD_r3**	0.255614	2.32307	0	0	2.41455	0	7.97677	0.897081	2.58718	0	0.217929	1.90293	0.571981	5.19132	0.194494
**F_r1**	0.691834	1.81961	0	0.33706	5.19329	0	4.18001	4.35509	4.27856	0.377377	2.64169	0.642359	0.777029	1.65119	0.783568
**F_r2**	0.635024	2.02583	0	0.154673	3.25545	0.24682	3.01275	3.43872	3.18762	0.693059	2.82698	0.589422	0.356808	2.78599	0.957913
**F_r3**	1.63609	1.91361	0	0.717695	3.66209	0.251422	6.44004	3.48464	5.39573	1.42574	2.36605	0.456271	0.275068	3.22219	0.806276
**GS_r1**	0.367369	0.997873	0.207452	0.22366	0.566452	0	0.356161	0.250079	0.106219	0.15045	0.0777951	0.681632	0	0	0.276605
**GS_r2**	0.240379	0.397167	0.220923	0.17048	0.589849	0	0.322011	0.115069	0.374274	0.285278	0.130409	0.610045	0	0.170731	0.432276
**GS_r3**	0	1.44018	0	0	2.6228	0	0.413753	0	0	0	0	0.44521	0	0	0
**IF_r1**	0.764415	1.52495	0	0	2.80062	0	3.2908	2.32503	8.43531	0.556874	1.72495	1.41766	0	1.22943	0.766179
**IF_r2**	0.948452	1.65939	0.356937	0	2.16444	0	2.7255	3.43596	4.63429	0.34518	3.48277	0.586713	0	1.01135	1.19099
**IF_r3**	1.1175	0.83854	0	0.217745	1.37562	0.173872	2.79495	5.45226	4.91757	0.609892	3.88432	0.622291	0.376806	0.892131	1.34812
**LSD_r1**	0.553639	0.103493	0	0	0	0	0	1.77099	0.852091	0	0.117089	0.641636	0	0	0
**LSD_r2**	0.150566	0.223706	0.170813	0	0	0	0.129137	0.843726	0.575899	0	0.126867	0.557343	0	0	0
**LSD_r3**	0.43159	0.3224	0	0	0.198084	0	0	1.84665	0.774269	0.15729	0.48652	1.06677	0.162058	0.2322	0
**LMSD_r1**	0.782393	0.78478	0.146972	0	1.77686	0	0.225239	1.86966	2.0181	0	0.996493	0.242224	0	0.620822	0.0985657
**LMSD_r2**	0.5444	1.1574	0.204801	0.176793	0.744624	0	0.62601	1.4757	2.59173	0	0.615368	0.084209	0	0.869254	0
**LMSD_r3**	0.153159	0.574852	0	0	0	0	0.132118	2.21134	2.01082	0	0.909128	0.568649	0	0.488632	0.115523
**OL_r1**	1.17889	8.13618	0	0	0.967846	0	5.6928	0	4.36753	3.86076	0	1.09404	0	1.1301	0.177725
**OL_r2**	1.34564	4.4408	0	0	3.85849	0	2.50863	0	1.72475	1.47109	0	1.14342	0	0	0.336788
**OL_r3**	1.6868	6.72425	0	0	2.12713	0	3.50813	0.257261	2.81149	1.84384	0.118886	0.651566	0	0.453109	0.527889
**R_r1**	0.427391	1.75435	0	0	9.33011	0.112183	9.55505	9.82598	6.01937	3.03835	6.31995	0.264029	0	0.921799	1.22871
**R_r2**	1.10994	1.42071	0	0	6.77268	0.873135	12.6149	8.88173	5.42278	4.42147	4.7577	0.367378	0	0.384318	1.54662
**R_r3**	0.613773	2.12622	0	0	2.52522	0.119855	13.4186	7.53063	5.14452	4.86037	5.26265	0.782847	0.172619	1.23031	0.808406
**S_r1**	1.33003	4.16526	0.0939769	0	6.05575	0.130306	4.43672	12.4519	10.4288	2.72539	7.38462	0.0770756	0	3.07725	1.99905
**S_r2**	1.61175	3.96027	0	0	5.06424	0.180493	3.76588	12.4108	12.05	5.78769	6.81786	0.213485	0	1.66792	0.778601
**S_r3**	0.977334	3.28249	0	0	6.46762	0.30781	5.04242	13.6594	10.1363	4.70313	5.78099	0.181132	0	2.37115	0.806309
**YL_r1**	0.304763	2.73589	0	0	0.837443	0	1.4432	4.17147	2.93287	1.66515	3.99734	0.847807	0	0.245086	0.572744
**YL_r2**	0.301837	4.86491	0	0	1.44695	0	1.43099	5.74459	3.2595	2.14225	3.70827	0.700144	0	0.483023	0.909465
**YL_r3**	1.09908	3.03542	0	0	1.88432	0	1.77545	2.87247	3.22938	1.05044	4.7117	1.78412	0	0.440697	0.46527
