# Supplementary material for: Genome-wide identification and functional analysis of Dof transcription factor family in Camelina sativa
Source: BMC Genomics. 2022 Dec 8;23:812. doi: 10.1186/s12864-022-09056-9 (PMC9730592; doi:10.1186/s12864-022-09056-9)
Supplement: Supplementary file 10 — Additional file 10: Table S8. Expression levels (FPKM values) of 103 CsDof genes in roots and shoots under salt stress. [file 12864_2022_9056_MOESM10_ESM.pdf]

Table S8. Expression levels (FPKM values) of 103 *CsDof* genes in roots and shoots under salt stress.

Name	DH55_root_rep1	DH55_root_rep2	DH55_root_rep3	DH55_shoot_rep1	DH55_shoot_rep2	DH55_shoot_rep3	DH55_salt_root_rep1	DH55_salt_root_rep2	DH55_salt_root_rep3	DH55_salt_shoot_rep1	DH55_salt_shoot_rep2	DH55_salt_shoot_rep3
CsDOF1	-	-	-	-	-	-	-	-	-	-	-	-
CsDOF2	5	5	6	7	7	5	6	6	5	11	10	9
CsDOF3	46	48	43	84	64	79	5	5	5	8	6	8
CsDOF4	10	12	9	9	11	10	3	3	4	2	2	3
CsDOF5	4	6	5	20	16	15	11	9	10	19	19	16
CsDOF6	2	2	1	36	30	35	4	4	5	9	7	6
CsDOF7	-	-	-	-	-	-	-	-	-	-	-	-
CsDOF8	1	2	2	6	3	3	3	2	3	1	2	1
CsDOF9	-	-	-	-	-	-	-	-	-	-	-	-
CsDOF10	7	6	6	9	10	12	8	8	8	4	6	4
CsDOF11	-	-	-	-	-	-	-	-	-	-	-	-
CsDOF12	0	0	0	5	2	3	0	0	0	0	1	1
CsDOF13	6	4	6	5	5	6	1	1	1	1	1	1
CsDOF14	3	4	2	5	4	3	1	1	1	1	1	1
CsDOF15	9	8	6	10	8	10	3	3	3	9	7	15
CsDOF16	-	-	-	-	-	-	-	-	-	-	-	-
CsDOF17	18	19	18	67	58	58	11	11	14	25	25	25
CsDOF18	-	-	-	-	-	-	-	-	-	-	-	-
CsDOF19	-	-	-	-	-	-	-	-	-	-	-	-
CsDOF20	12	13	10	16	13	15	6	5	7	5	5	5
CsDOF21	4	4	4	1	2	1	2	2	2	1	2	2
CsDOF22	7	7	17	23	5	13	1	2	5	7	1	3
CsDOF23	25	25	22	18	18	17	7	6	4	5	5	6
CsDOF24	4	3	3	3	2	2	3	2	2	2	2	4
CsDOF25	37	34	34	64	44	53	31	28	30	25	31	30
CsDOF26	29	32	28	29	30	27	7	8	9	13	12	16
CsDOF27	5	7	10	41	22	32	8	7	9	15	13	19
CsDOF28	20	18	14	10	10	10	2	3	3	2	1	2
CsDOF29	1	2	2	2	2	2	5	3	4	1	2	2
CsDOF30	25	28	23	82	74	71	20	23	23	26	31	26
CsDOF31	18	19	15	12	11	10	12	11	9	5	6	5
CsDOF32	-	-	-	-	-	-	-	-	-	-	-	-
CsDOF33	8	8	7	7	7	9	5	6	5	4	5	5
CsDOF34	-	-	-	-	-	-	-	-	-	-	-	-
CsDOF35	40	40	31	23	21	23	3	4	4	3	3	4
CsDOF36	7	5	7	3	4	3	2	1	1	1	1	1
CsDOF37	32	34	35	64	41	49	22	18	21	17	18	20
CsDOF38	10	14	11	9	12	8	3	4	2	4	5	6
CsDOF39	3	3	4	21	15	16	4	5	5	7	6	8
CsDOF40	-	-	-	-	-	-	-	-	-	-	-	-

Name	DH55_root_rep1	DH55_root_rep2	DH55_root_rep3	DH55_shoot_rep1	DH55_shoot_rep2	DH55_shoot_rep3	DH55_salt_root_rep1	DH55_salt_root_rep2	DH55_salt_root_rep3	DH55_salt_shoot_rep1	DH55_salt_shoot_rep2	DH55_salt_shoot_rep3
CsDOF41	13	15	10	19	14	16	11	12	11	8	9	9
CsDOF42	72	71	63	116	76	92	7	7	6	8	7	10
CsDOF43	6	6	4	7	5	4	1	1	1	1	1	1
CsDOF44	3	3	4	5	4	5	2	3	3	2	1	1
CsDOF45	26	30	28	90	83	84	17	17	23	47	50	43
CsDOF46	11	11	7	7	7	6	8	8	7	4	3	4
CsDOF47	-	-	-	-	-	-	-	-	-	-	-	-
CsDOF48	8	8	9	12	9	13	3	4	5	2	3	4
CsDOF49	21	21	14	21	18	21	16	14	13	11	9	10
CsDOF50	-	-	-	-	-	-	-	-	-	-	-	-
CsDOF51	36	38	39	32	29	25	11	10	8	10	10	12
CsDOF52	-	-	-	-	-	-	-	-	-	-	-	-
CsDOF53	-	-	-	-	-	-	-	-	-	-	-	-
CsDOF54	4	4	5	23	14	20	1	1	1	10	7	11
CsDOF55	-	-	-	-	-	-	-	-	-	-	-	-
CsDOF56	26	28	30	21	20	24	8	7	9	8	7	7
CsDOF57	-	-	-	-	-	-	-	-	-	-	-	-
CsDOF58	-	-	-	-	-	-	-	-	-	-	-	-
CsDOF59	-	-	-	-	-	-	-	-	-	-	-	-
CsDOF60	3	3	4	15	9	12	1	1	1	10	5	15
CsDOF61	15	17	14	17	24	17	5	5	3	3	6	5
CsDOF62	6	6	6	25	17	15	10	9	8	18	20	16
CsDOF63	2	1	1	8	5	7	1	1	2	3	2	2
CsDOF64	6	6	5	9	7	7	3	3	3	3	4	3
CsDOF65	-	-	-	-	-	-	-	-	-	-	-	-
CsDOF66	-	-	-	-	-	-	-	-	-	-	-	-
CsDOF67	-	-	-	-	-	-	-	-	-	-	-	-
CsDOF68	-	-	-	-	-	-	-	-	-	-	-	-
CsDOF69	36	40	37	33	30	28	11	9	9	10	10	13
CsDOF70	-	-	-	-	-	-	-	-	-	-	-	-
CsDOF71	-	-	-	-	-	-	-	-	-	-	-	-
CsDOF72	-	-	-	-	-	-	-	-	-	-	-	-
CsDOF73	-	-	-	-	-	-	-	-	-	-	-	-
CsDOF74	-	-	-	-	-	-	-	-	-	-	-	-
CsDOF75	3	3	4	19	9	16	1	1	1	7	5	8
CsDOF76	2	3	2	3	3	1	1	1	1	0	0	0
CsDOF77	15	15	14	24	18	23	2	1	2	4	3	4
CsDOF78	9	7	6	17	17	20	10	9	9	6	6	6
CsDOF79	-	-	-	-	-	-	-	-	-	-	-	-
CsDOF80	1	1	0	6	3	3	0	0	0	0	0	0
CsDOF81	7	7	8	9	7	7	1	1	1	1	1	1

Name	DH55_root_rep1	DH55_root_rep2	DH55_root_rep3	DH55_shoot_rep1	DH55_shoot_rep2	DH55_shoot_rep3	DH55_salt_root_rep1	DH55_salt_root_rep2	DH55_salt_root_rep3	DH55_salt_shoot_rep1	DH55_salt_shoot_rep2	DH55_salt_shoot_rep3
CsDOF82	5	4	3	4	4	3	2	1	1	1	1	1
CsDOF83	9	11	8	15	10	11	4	3	4	17	11	16
CsDOF84	6	5	6	8	8	4	6	7	6	8	9	8
CsDOF85	18	18	14	13	11	10	2	3	2	1	1	2
CsDOF86	5	5	7	3	4	3	1	2	1	0	1	1
CsDOF87	35	37	34	67	46	56	19	17	18	16	17	16
CsDOF88	14	16	14	18	17	11	4	3	4	3	3	3
CsDOF89	-	-	-	-	-	-	-	-	-	-	-	-
CsDOF90	7	6	5	6	8	8	8	6	5	3	6	5
CsDOF91	-	-	-	-	-	-	-	-	-	-	-	-
CsDOF92	0	0	0	7	5	5	0	0	0	0	0	1
CsDOF93	11	11	12	15	13	15	2	3	2	1	1	2
CsDOF94	4	4	2	3	4	3	1	1	1	1	1	1
CsDOF95	11	12	13	16	15	18	7	5	5	22	13	25
CsDOF96	11	12	11	12	15	13	4	3	4	3	3	4
CsDOF97	7	10	7	24	21	16	11	10	9	23	25	20
CsDOF98	6	4	4	40	33	35	3	3	2	5	3	4
CsDOF99	5	4	5	8	8	5	3	3	3	4	5	5
CsDOF100	-	-	-	-	-	-	-	-	-	-	-	-
CsDOF101	-	-	-	-	-	-	-	-	-	-	-	-
CsDOF102	6	5	7	9	9	7	5	6	7	8	6	8
CsDOF103	-	-	-	-	-	-	-	-	-	-	-	-
