# Supplementary material for: Genome-wide identification and functional analysis of Dof transcription factor family in Camelina sativa
Source: BMC Genomics. 2022 Dec 8;23:812. doi: 10.1186/s12864-022-09056-9 (PMC9730592; doi:10.1186/s12864-022-09056-9)
Supplement: Supplementary file 11 — Additional file 11: Table S9. The 2000 bp promoter region upstream of the CsDof genes. [file 12864_2022_9056_MOESM11_ESM.pdf]

Table S9. The 2000 bp promoter region upstream of the *CsDof* genes.

Name	Gene ID	Promoter sequence
CsDof1	Csa00734s020.1	CATGAGAGGGAGAAAAAGTATTTTGTGTTTGGTTTTTGAGTCTATTTTTTGGTCCAACTCGATCGAATCTCCAAATGACAAGGTCCATC<br>TCTATATATCTTCACTTAATGTTGGAAAGTGATTGGTCTTTCAAAGTCTTTGTAGATTCTTCCATTTTTATTTTTGTTTTCAAAAAGAAA<br>AAGGAATAATTCTAACTTTTGCTGAACAGAGATTATCAACATATTTCTTATCAATTAGCTTCTGATAAGAGTGAACGCGTATAACCTTA<br>AAGGTCTTAACGCGTATTATCGATATATAATAATATAGTATAAACAATAAAGAATTATATTATATCATAGTCTTACTAATACCATAATA<br>TATATTACGTCATAAGAATAGTTGCAAAGGCTGCTTCTAGATACTAGATAGTTAGTATTGAATCAGTCTTAAAGTCTTAACAATGCCAA<br>TATTTCGATTTCCTAAACAAGAAACAACTCCTTAGATCAAAGTTGTTTTATATCATTGAGGTTGTTTTCTTTCAAGGCTACTCATATCAA<br>AATCAAAACGGAGCTGTAATGTGGTTAAAAAGAAAGAAAAGAAATAGCACGGAATCAATACGTATCTTTTTCATAAACCTAATCATAT<br>ATTCAGAAAATGCGTAAATGAAACATGAAAGTTACTGGAGTACCAATCCAAGATTAATGTGGTTCCTAACTTTTACTAAACCGAGAAA<br>ATCAGAAATCCATACCCGTACCAATCCAAGATTGTATGTATAGCTAGCTAAATTGGTCGACTACGTACTGCAAGTTACATAGACCAAA<br>TACAAATGGTAGAAACGAAGTATAACAGATTATAGGCGGAATCGTTTTTGGGGAAAATGAACCACATTTTTATCCCTAAAAATCAAGT<br>AAACAGAAAAACGTAAAAACTCAGCGGAAATTAATTGGTGAATTCATCTTACAATAGAGAATAACATCTTTAATCTTCTCATCCTTGT<br>CCCTGTATTCTACTTACATACATTCAAGGTTAGGTCAAGCAGACAAAAAAACGGGAATGCATCAACCGAAATTTTCTGATGTCTCCTCC<br>AATAGATATCTATCGACTTGAACTAGTGTGACATTTAAAAAAAAAAAAAAAAAACTCTTTGAAACCACTAGAGCAACCTTGGTTTTAA<br>CAATATTTATATGTAGAGATTTACCACGACTGTTAGTAACTGAAAGAAAATGATGGCACAGCCACAAATGTACATACTTTATACGTAT<br>TAATTTGCTCTTGGATGGTATTGTTGATTAAGCTTGAATTAGCTTGAGCCTAAAGTCAAGTATATTTAGGAGTTATCTAATAATTTAGT<br>TCTCCATTTTGTATTAGGAAACTTGTCTAAGAGTTTTTCTATATAAGAATATGTAAGAGGTGTTGCATAACTTATGAGATTTGGGAGAA<br>CAAGAGAGAGCTTTTGGCTTTGAGAGATTTTCCTAAAGGTTAATAAAAGAGAGTAATTCTTTTAGTTCTTGTGTTCTTAAATCTTAAAG<br>AGTTTCTTTGATTCTAGATTGTACATACTTTATACGTATTAATTAGCTCTTGGATGGTATGAAACAAAAACTTTCAGAAATTCATCCAA<br>TCGTTTTATTTAAACTCGGATTATTAAATGAAAATTAATAAACATTTTATTGTCATTATACAAGTTTACGAAGGACCAAATTCGAAGGT<br>AACTTGATGAAGAAGAAACGGAAAAGGAGACGCACGGCTCGGCAACCGCCCACGTTTACTTCGTAAGCCTTCACACATGTATCGATG<br>CACCTCTGCTTCCACACACACATGCTCTTCCTCACCTCTAAGTTCACATATACACACACACAACCCCATATATATAGAGTTAGATGTAC<br>TCGAATATACACCAAGTACCCACTTGTTAGCCTTTCCAATCTTTACACATTCAAAATTCATAAAAAAAAAGTATATGAAAAATGTTGCC<br>GTACATTGGACACAACAGCTACCAGTAGCACCAGTTTCCATCACCGGAG
CsDof2	Csa01g024360.1	TGTTGGAAAAAGTTATTACCCCTCGATTTGTTCAAAGTTTTTTTTAACCATAATGCTCACAACTTTGATTTTTCTACATTTGTAAAAGTA<br>ACTCAAACAATTCTTACTAACTCAAACAATTCTTCGAATTAATTAGACATATAAAAGTAAATATTAATACTTCCGTTTTATATACTCTTT<br>ATATAATCGTTCCGACAAAATGACATCAAACAAAACAACTCTATGATAATAGTACTATTAGTTAAAGAAGTCAACAAGTGAATTGAAA<br>AAAAAAATTTCAACACCATCCTCTTGTCTTCTAATTACAAAAATTAATAATCACATAAATAATATAATACATAAAAATTAAAAAGATAA<br>ATAAGAAGAAGGAAGATTACGTAGGATAACATTAATCTTGTAATTTTGGGTTGCCCTATAAGACTGGTCACACACACCACAGCTCACA<br>AACTTGTCTTGTCGTTTAGACTTTAAACCCAACACTCTCTTCCTCTTCCTTCCTTCTTCTCTCTCTCTCTCTCAATTTTTTTTGTCTTATAT<br>ATGCGATAAGAACCACAATTAATTACTCTATCAAAATCTTTTTTTTGTTTTCTTGATTGNTGAGCCAGAGTGGGTTGTGGAAATGATGA<br>ACTCGATCGGTGCCTTTTTTTATATAATTATTTTCAATCTTTTTAGCTAGCTAATTATGTAATAAACCACTAGATGATGCTTATTTAATG<br>CAAATTTGATTATGTATAAAGAAGACTAGTTTGATTACTTCTAATATTTCTTATTTGATTATAAAAACAAAAATGGAAGTCTACGTGTA<br>GGGCTTGTGCATGGATAAGAAAAACTCATGGGAAATGGGCGGGGCTTCGGGAGAGAACAACTCTTTTCTGACGTTTTTGGTTGTCTTT<br>TGTTTGACGTCTTCTTTGGTTAGGTTTTTTGATTAATTCAGAATTCTATTTTTTTTGTCGGCAAGTTTATATAATTAAGAATTAAAATGG<br>GAATTCTCTTCTAAGCAAAATATTCGAACGTATGTAAAATGTTACTATTTGTTAGAATGTGGTCATTTGCTCCTTCGGTGATTTAATTC<br>CCCTTGCTCAACTTTGTTGCTAATAGAAAACACTTTGACAAACCAAAAATTTTGATCTCTTATGGATTTTATTCCATCGGTTTAATTACT<br>ATAGAGTTAATTGAGAGTCACTAGCTAGTGAGACTTTTGTTCTTGAAAGAAAAAAGGTAATTGTGTTTGTATTCGGGTTAAAGTAAAG<br>TACAAATAATAGAAAACAAAAAGATACGTAGAATCGATATTTGTCTAAACATAAGAAGCCAAACAAAACCATCTTGGCACAAAGACA<br>GGCGTATCCAAATATGTTGGAAAAAGTTATTACCCCTCGATTTGTTCAAAGTTTTTTTTAACCATAATGCTCACAACTTTGATTTTTCTA<br>CATTTGTAAAAGTAACTCAAACAATTCTTACTAACTCAAACAATTCTTCGAATTAATTAGACATATAAAAGTAAATATTAATACTTCCG<br>TTTTATATACTCTTTATATAATCGTTCCGACAAAATGACATCAAACAAAACAACTCTATGATAATAGTACTATTAGTTAAAGAAGTCAA<br>CAAGTGAATTGAAAAAAAAAATTTCAACACCATCCTCTTGTCTTCTAATTACAAAAATTAATAATCACATAAATAATATAATACATAAA<br>AATTAAAAAGATAAATAAGAAGAAGGAAGATTACGTAGGATAACATTAATCTTGTAATTTTGGGTTGCCCTATAAGACTGGTCACACA<br>CACCACAGCTCACAAACTTGTCTTGTCGTTTAGACTTTAAACCCAACACTCTCTTCCTCTTCCTTCCTTCTTCTCTCTCTCTCTCTCAATT<br>TTTTTTGTCTTATATATGCGATAAGAACCACAATTAATTACTCTATCAAAATCTTTTTTTTGTTTTCTTGATTGTTTGATAAATTTTTCAA<br>GAAACACGAAAAGATCATCAAAGAAGAGAAGGAAAA
CsDof3	Csa02g002370.1	TAAGTCAACGAGAGTTTGCGATTTATTTGAAAGTCAACAAATAATTGGTGTTTTTTTTCTCCAAAAGCAGAGTTCGGGCTTTAAATCAA<br>CGAAAATGTAGACTTGGGGATTTTAATGACATTTTTCCCTAATCTATACTAGTATTTTTGCAGCAATTTTTGCTCAAAAATATTTTTTGG<br>AAAGTTTTTACATTTAATGCATTGACTTTAATATTAGTTACCAAAACTTCAAATTTAATTATAGGTAAGATTTTAAAAAATAAGAAAAT<br>CTTTTTACCTCATTCAAACATAAATATATGATTCACTTTCATTTTTATTTGAATTTATATTTAAATTATATTGAATTATTCTATAATACAT<br>TTAGTTAATAAAAATTGTGATTTTTTTCCTGCATATGATGTAATACAAAATTTTAAAAACGGACATATATTACTCAATAGATGAATAAA<br>AAATACGGGTACAATATCCCACATGTCTAAAAAAATTGGGCAATAGTTTATAATCATACTATAAAAGAGACCAAAATGATTCTGAATA<br>CAAATGAATAGAAACCTTAATTTATCAGGCATTCAAACTTTAAAAACTTTTATTTGGTTTATTTTGGTTCTTTATTTTAAAGATTTTTAA<br>AAGGTTAATATGAAAAATATTTAAAACTTTTAAAAAGTTAAATATGATAAATATTATACCGTAGATACACAATAAATATTAAAAATTA<br>AGATGATATATTGTGAGTTAAAATATCTCGGGATGGGTTATATAGCGGGATGGGTTTTACCGATATTTATATAAATTAAAAAAATATC<br>ATTAATTCGATGTTACACGGGTAAAACATCATTTCATTATTTTGTTAACACTGTCAGTATCAGAAAATAGACATATCTAATTAAATTGA<br>TTAAATAAATATTATGGAAAAAATAAATTATATTGCGGTATTATATAGTTTATTTAACAATTATTTTATAATTATAACATATTTAACCA<br>ACAAATACAACTTATTATTTAAATATTTTAGTTATAAATAATTTGTCCCGCGGTATACCGCGGGTCCTAGTTTCTCTTATTGTTATAGA<br>ACTATTTACTATGTTATTGCTTAATTAGTTACATGATTAACGTATCTTTTTCTTTTCTTTTCCTTAATCATGGTTTTAGATTTCAAGTGCT<br>ACATTTTTCAATCAAGGTTTTAGCTTAATTTAGTTAGTTAGATGTATGGTTTGTACACTCTAATTTTTTGGCCTCGAATTGAAAAAAAT<br>ATCAATATAGTATGCTAAATATCAACTACTTTATGATGATATAACCATCTATTAAAAAAAGAGAGAATTATAAGATAGTCAAAACAAC<br>ACACCACTAAAATGCCCATAAAAGTGAAACCCAACACTTCTAAACTTTAGCCTCAAAATATTCTCTCTCCCTCTCTCTCTCTAAGTCAT<br>CTCTTTGAACATCAGGTCAGTTCATCTTCTTCCCTTTTTCTCTTATCTCCTTGTTTCTTCTGCAAATTTCTTTCTCTATTCTTTTTTTTTTT<br>CCCTTTTAACTTTCTTTTGCTTTTTGGTTTATGTGTTTATTAGGGTTTTTTTTGTTGTTTTTTACTTTTCAAAGGTTTACATCTTTCCACCT<br>TCTGATTTCTTTGAAAATTTGTCCCTTATTTAAAAGATATGTCTGGCTTTTTTAGAGATCAGTTCCTAACACCTTTTTGTTAGTGTCTTG<br>CTCGATCGATCCATACGTTTAGAAAGTGATTATTTGTTCCCACTCTCTCAGATCCTTTATTGATTAACTTTGGTCGTTCTCATTCATATA<br>CTAGACATAGTTGGTTCATGGTCTTCGACTTTTTATGTTCTTTCAATAGTTTTTAGTATTTTATTTATTTGTATGTGTCACTTTTTTATTT<br>GTGCTACTTTTTATAAGTTCTACTTTTGTTATCATGTTTTCATATGCTTCATTAGATGGGTTGATCATACATGATTATCACAGGGATTTA<br>TTATAAACTGTAATTGAAGCTTATACATC

Name	Gene ID	Promoter sequence
CsDof4	Csa02g067130.1	ACATGCATTTAATCTATACTTACTTTTTCGTTTTGTAATAGAAAAAGAAACATATTTTCAGTAGACCAACAAAATAATCACTGTTTACA<br>AAAACAAAACCAACAAAATAATCACTAGAGAACTATCCAACATTATTTTATGGTAATAGTTTGAGAGTATGGCTAAGTTACACATTTT<br>CTCTTCCCACGACACATGGTTCAAAAAATCGTATAATATACTCCATAAGATTCTACAGATTAAAAAGCAATACTATAATATAGTATGAT<br>GTAACATATGTGTGGATATGGTCCTTAGACCAAATCTCGGTCTTCATAGAAGTGTCTTTCCCGAAAACCGATGTGTGATTTGTGTCTCT<br>ATGAACTTTAAAATCCCCATCGGATAATGCAAGCACATCATGATGATGATGAGCTATATACAACACATTGTCCGGCCAATTATTTGCTT<br>TTACTTCATCATGTGTTCTATTTACATATCTTATTATTGTGAATATATATAAAGCAATATGTACCTAGAATTATTTTATTCACATTTTTG<br>AAAAAATATATTTCTTTTAATATCCGTAAGAAGTATTTTAGTTTGTGTTACTGATGACAGTTGTTCCAGATTTAAAAACACACATATTT<br>AACCATTTTTCAATTTTGAATATGTATCAATACTGACTTCTACACAACAAATCATTGACAATTATGCTCAACGAATTGCATAAGTAAAA<br>TAGAGGCATAAATTAAGAAGGAAAGAAACTGTAGCCCCAATATGTTTTGACAAAAATAGAAACATTTCTGTCCTATCAAATAAAGAGA<br>ATCTATATATATATAATTGACATTTATGCGTTCTACAATGCAATAAATTCCCATGCTAAGAAACCAACAAATACTAATATAAATTCGAG<br>TATCTGTAATTGTTTTTGCTTTAAACATGGAATTAGAATGAACGATCGAAATAGAAAATCACATATACTTTGTTGTACGAACTACGAAG<br>GTCACTTTGAAACGGTAAGGGTCCATTTAGGAAGAAACAAGACATTGCGATGAGTACGGGACCCGCGTGGTGACAACTCAACGGACC<br>ATTGGGACATAAAACACAGAAATGACAACGAAGCTGAGTGCGTGGGAATCCGACGTCGAACTCCAACCATCTCTCATCCATCATCGTA<br>CGGTTACTATTCATCTTGAATCATATAGATCCGATATCACATTATTTGATAACGACGACTGCAACTCATTCATTTATATATAAGTTGAC<br>AGCTTAACCGATACATTATTGGTGGAGTGGCTAAACGAGCTTAGTGATTATTTCGGACTATAGTTATAGACTTTAGGCTACAAGAGTT<br>TTGTTTTCTACCTCAATATAAAACTACAATTTTGATTAGTAAAAGTTATTGTTTTTTTCGTAAGATATTATACAGTCAAAATATACAAAT<br>GTTAGATTATTTGGTAAATATGTTTAAATATGATTAGTATTATTTTTAAATCATCCAAAACATTAGTATTTTCGAGCAATAAAATAACT<br>CTTTTTTTGAAGTAGTACTCTTAACTAGTCTTGAAGAGAGATGGTAACACATTATTAGTTTATACTTTCCACATGCCTTTATCAAACTA<br>CTAATTATGTAGAGATAAATTTCATAATATGGTTACTGTATGTAGAATTTTTGTTCACATTATATAAATGAATAAACGGCGGCTTTATG<br>TCTGTTCCAAATCACGTTCCGAATTGGTTCACATTATTCTTCTTCTTCATACCACCATTTTGTTTTAAAAATAATTAAATATTGAATAAA<br>ATAATATGAGATCGAGGTCACAACAAGAATTGTGTCTCTCTCTCTGCTCCCACATTCCCATGTATATATACAGATGCTTGGCTTTTCCT<br>TTTGCTTCACTTTTACTTTCTACTCGTATCCATCATTCTCTCTCTGATCTCAAGAAACAATCTCAATCTCCCAACATATAAACACTACTC<br>ACACTCATTACTCTTCAAGCAGCTTCTCAGATTCATCAGCTCTT
CsDof5	Csa02g067810.1	AAAATCTTGAATAGTGACTTGATTAGTTTTTGGTTTTTATGTATAATAATTATTTTAAAAGTAATAAAATAATGATTATAATTTTTATTA<br>AAAAAATAAAAAAATACAATGATGTAGAAAAAAAACATACGTTTATTGCATATAGAAAACATATTACTGCAAAATTACTATCATCATC<br>ATCGAGAACTCCATAATGATGCAGAAATTTCATCCCGAATCCCTTCCATATACTGCATTGGAGTATTGTTTTGCTCTAATTCTTCATCT<br>GAATCAGAATCTTCTTCAGCTATATTACTAGCTATGCGGTCTGAATCGAAGTCAACATCTCGTAGATTTGATAACCTAATAAAATTATG<br>TAATGCCATTGTTGCAGCAATAACTTTTCGTTGAACATGTATGGCATACCTTGGTTTTTCTCTTAAAACTCGCCATTTTGCTTTCCATAC<br>ACCAAAAGTCCGTTCGATGACAGATCGCAGTGAAGAATGTCGTTTATTGAACATTTCTTTGTGACTACTTGGTGGTCTAGCTCTATTAT<br>ATTCGCTAGGATGGTACCTTTCCCCTCGATATGGAGTAAGGAATCCAGGTCGAAGAGCATAACCAGAATCTCCCAAATAATATTTGCC<br>CATAGGTGGAGTTGGAAATTGATGATTCCCCTCCATCGCTAGCATTAGAACCTTAGCATCATGAGCAGACCCGGGAATACCAATATAC<br>GCATATGTAAACAACATGTCCATATTGCATATTGCTAAGATATTCATACTTGTGCCGCTTTTCCTATTCCAATATTTTTCTGAATTTTTT<br>CCCGCAACAGTGACCGGAACATGTGTTCCATCTATTGCACCAATAAAACCTCTAAAGTAAGGCCAGTACCTTCTGTCAGATTGTAACT<br>TTGGATGAGGTTGATTGAGCTCTTCTGGACCAGGTCTAAGCAATTCAACCGCCATGTGTTCGAGGGCATTCAACACTTCGTGAAATTTT<br>CTGCCTATGGTTTCTTGTGAATGACCAAATATTTTTCCAACATACCTCTGAGTTCCGTTTTGAGCGCATATGGTGAGAAAAATTGCAAC<br>GCTTTCCTGGATGCTTACTTCATCGGTTTCCATCAAGTGATGGTCCTCCTTAAGTTTTTCACATAGTTTGATGTACACCGGTGGATACA<br>TCCGAATTATGTCGTAGCATTGTTTCTCATGACCATGAATTAGTTCATATACGTATCTTCGGCCACGTTCTGGATCTTCATTCATCGGTC<br>TCCTTTCTATACTCATGGAGTAAGGATCCATGAATGATTCTCCAAAGTTAATGCTTCCATCTTCTGCCTCATCATCTTCAAGCATTCTGT<br>AAAACGCTTCCCTCTAAAAAAAAAAAAAGAATAATGAATTACTAAGGTGAAAATAGAAGATATTTATTAATTTCAAAAACAACAGAAAA<br>ATAAACAATCATTATTCTAAGCCAAACTAAAAACATTCAAAAAAAGTGAAATAAAAAAGTTTTCTTGTGATAAATATTATTCATTAGC<br>CTATTCAGCTGTTCGGCTTATTCCCGCTGTCCGCTCGAGATATTTGATCCTAGCATCGTCATTGGGTAGTGTTAGATAAACACGTGCAT<br>GCGTCTTGTTGCTTTGAAGCACACTCACCGCTCCCCAATATAAAAAGAGTCATATTGAAGTCCAGAGAGTGTTAGTAGCGATTCAATT<br>GCTCGTTCCAACTTGTTATTTTGGGCTTGCTCATAAATTTCTGTTCGTTTCTTGTAAGCGTCATTTGTCACTTGATTTTCGGTTGTATAT<br>ATTTATTTGTATGCCTATGGCCTTGAATGGCAGTTTAATTTCTAAGACATATCACAGAAATTTAAAAATTAAAAACTGCACCAAAGTTG<br>ATATGTCTTAGACTCAGTACGAGAATAATTTCTAAAGATTAAATACAATTTATATACCATATATATATATATATATATATATATATATA<br>CGTAAACACTAGTCAACTCATTTCAAAACATTTTTTATATGAT
CsDof6	Csa02g070560.1	TTCAGTTGATAGCGTAGTTAATGATAAGCATTGGGGGTGGTCTGGATAATCGTCTCTCATTCAACATTTCACAGCTACATCATTGATCC<br>AGACTTGCATATTCTTCTACTCTGTTACTTGGAGAAAGTTATATTTATTTCATTGTCTTACAATCAGTGCATATGATTGATTGGTTTTTG<br>TTACTCACTTCTTTTATAAACTTCAGATCATATACATACTTGGAAGTATATATGTTAATTTAGGTCTGATACTCTGAATTTGACAGTATA<br>AGTATATATGTTAATTTGAGAAAACAAGATAGAATTTTCAATTTGTTTGGCATCTAACAACAGAGACTGTTGACAGAAAAGAAATAAA<br>AAAACAGAGTAACAAGTGAAATAACCAATGTTTACGAGCCCTGCTAAGAGAAAGTCAAAGCCTTAAACTAAAGCTGTCTTAGGAGTT<br>AGGTCTTAAGCCTCTTACAATATAACTTTTGACGATGAAACTACAAACAAATCTTGTATCATTATTAAAATACACATATTGTAAAAAGA<br>TGATTACATGGAGTGATACAACTGGTTGGACATAAACTAATTAAATTTTCAACTTTTTATTTTTTTTAATGCAAATCTTGGATGGGAAA<br>TAATGGTTTATATTCAAACACATTATGAATTTTAAAGAAATGTTATCTGATATTCGGTATTCGGATGAATATTCTAAAATATCACAAAC<br>ACATTGTCTTTTGAAGGTACAACACTTATTTTCTTACTTTTTTTTTTTTTTTATGTTAAGGAAGCTTCTTAACTACGGGTCTGACTGCAT<br>CTGCATGCAATCAGTTTGTGAAATCTATGATCTGACCAAGAGGTGCAGGTGAGTAACGGACCCACAGGTAGAAGTTGACGTGGAAAA<br>GGCAGAGTCTGGTTAGATCCATGTAAATCAATCCTAAAGACTAAAGAGTTTGGTAAATCATACTATGTGGCAGAGACCTTAAAAGACC<br>TTTTTGGTTATATCTAATGAGAGAGCACCTCATCTCTATTACCTTCTTCACGTACAACTACAACCGTTCACATAATGTTCTATTATTTTT<br>AATCTTCTTTTTTTTTTTTTAAATAGTTCTGACAACAAAATTGAATCGAAAAGGTTTAAATCCTTGTTGCATTTGTATTCCTGTTTAAAC<br>ACAAAGATTCATAGCTTCCTATAATCTTATTAAATCTTCCGTAAAATCTACCTTCTAACAAAAGAAGGTTCTTTAACAAACAAACAATT<br>AGAAAATAAAATAGACGGTATATTTGATATTTCTCTCCAAACCAAAAAAGTGTGGATCGTTTTCAAATGGGGAATTTTCATCCTCCTAC<br>CAACCAATTAAGAAAATGCACGTAACAAGACAGCTCTCGTGTGAGCTGCACCAAAGCCATCCCCTCGATGCTGACATTTACCTACCCA<br>AAAGTTTGTAAACAGTTTACATTAGTTTATTTGTTCCAATTGAAACGAATAAATAGAGTATATCACTGTTTAAGATATGTTATAAAACC<br>AATCTTATACCATATAATAAAGCGTGTTTATAGATCTTGACTAGTTGACTCCATGCTCTTAATTAGGAACACTTCGTTTTTCGTATGTTC<br>CAAGTACTTGTGAGTTTTTTGGTCAATGGAGGTTTAGTGAAAGTCTTAAAACAAATTAAAAAGGTTATTCTTACAAAGATTGGAATAA<br>TTTTAACAAAAAGTTTAAAGAAAATAACTAAAAGATTATATGCTATATGGGTTCAATGGGTTTTGTGGAAAAGAAAAGTAAAATATCA<br>GAGGCCACATAAACTTGTGGTTGAGAATGAAACGAAAATGCTGACATGGCATCCACTTCACTTTTTAAAACGTATCAAAATCCCAAAT<br>CTCACGATTTCCAAACCAAAAAAATTCCAATCTAAACAAACCCATACCTCACAAAAAAAAAAAAAATAGCTTCGAAGAGAAACCTACGA<br>GAAAGTAAAAAAAAAGAAGAAGAACAAATTCAAGGAAAGAAGAAATA

Name	Gene ID	Promoter sequence
CsDof7	Csa02g071140.1	NNNNNNNNNNNNNNNNNNNNNNNNNNNNNNNNNNNNNNNNNNNNNNNNNNNNNNNNNNNNNNNNNNNNNNNNNNNNNNNNNNN<br>NNNNNNNNNNNNNNNNNNNNNNNNNNNNNNNNNNNNNNNNNNNNNNNNNNNNNNNNNNNNNNNNNNNNNNNNNNNNNNNNNNN<br>NNNNNNNNNNNNNNNNNNNNNNNNNNNNNNNNNNNNNNNNNNNNNNNNNNNNNNNNNNNNNNNNNNNNNNNNNNNNNNNNNNN<br>NNNNNNNNNNNNNNNNNNNNNNNNNNNNNNNNNNNNNNNNNNNNNNNNNNNNNNNNNNNNNNNNNNNNNNNNNNNNNNNNNNN<br>NNNNNNNNNNNNNNNNNNNNNNNNNNNNNNNNNNNNNNNNNNNNNNNNNNNNNNNNNNNNNNNNNNNNNNNNNNNNNNNNNNN<br>NNNNNNNNNNNNNNNNNNNNNNNNNNNNNNNNNNNNNNNNNNNNNNNNNNNNNNNNNNNNNNNNNNNNNNNNNNNNNNNNNNN<br>NNNNNNNNNNNNNNNNNNNNNNNNNNNNNNNNNNNNNNNNNNNNNNNNNNNNNNNNNNNNNNNNNNNNNNNNNNNNNNNNNNN<br>NNNNNNNNNNNNNNNNNNNNNNNNNNNNNNNNNNNNNNNNNNNNNNNNNNNNNNNNNNNNNNNNNNNNNNNNNNNNNNNNNNN<br>NNNNNNNNNNNNNNNNNNNNNNNNNNNNNNNNNNNNNNNNNNNNNNNNNNNNNNNNNNNNNNNNNNNNNNNNNNNNNNNNNNN<br>NNNNNNNNNNNNNNNNNNNNNNNNNNNNNNNNNNNNNNNNNNNNNNNNNNNNNNNNNNNNNNNNNNNNNNNNNNNNNNNNNNN<br>NNNNNNNNNNNNNNNNNNNNNNNNNNNNNNNNNNNNNNNNNNNNNNNNNNNNNNNNNNNNNNNNNNNNNNNNNNNNNNNNNNN<br>NNNNNNNNNNNNNNNNNNNNNNNNNNNNNNNNNNNNNNNNNNNNNNNNNNNNNNNNNNNNNNNNNNNNNNNNNNNNNNNNNNN<br>NNNNNNNNNNNNNNNNNNNNNNNNNNNNNNNNNNNNNNNNNNNNNNNNNNNNNNNNNNNNNNNNNNNNNNNNNNNNNNNNNNN<br>NNNNNNNNNNNNNNNNNNNNNNNNNNNNNNNNNNNNNNNNNNNNNNNNNNNNNNNNNNNNNNNNNNNNNNNNNNNNNNNNNNN<br>NNNNNNNNNNNNNNNNNNNNNNNNNNNNNNNNNNNNNNNNNNNNNNNNNNNNNNNNNNNNNNNNNNNNNNNNNNNNNNNNNNN<br>NNNNNNNNNNNNNNNNNNNNNNNNNNNNNNNNNNNNNNNNNNNNNNNNNNNNNNNNNNNNNNNNNNNNNNNNNNNNNNNNNNN<br>NNNNNNNNNNNNNNNNNNNNNNNNNNNNNNNNNNNNNNNNNNNNNNNNNNNNNNNNNNNNNNNNNNNNNNNNNNNNNNNNNNN<br>NNNNNNNNNNNNNNNNNNNNNNNNNNNNNNNNNNNNNNNNNNNNNNNNNNNNNNNNNNNNNNNNNNNNNNNNNNNNNNNNNNN<br>NNNNNNNNNNNNNNNNNNNNNNNNNNNNNNNNNNNNNNNNNNNNNNNNNNNNNNNNNNNNNNNNNNNNNNNNNNNNNNNNNNN<br>NNNNNNNNNNNNNNNNNNNNNNNNNNNNNNNNNNNNNNNNNNNNNNNNNNNNNNNNNNNNNNNNNNNNNNNNNNNNNNNNNNN<br>NNNNNNNNNNNNNNNNNNNNNNNNNNNNNNNNNNNNNNNNNNNNNNNNNNNNNNNNNNNNNNNNNNNNNNNNNNNNNNNNNNN<br>NNNNNNNNNNNNNNNNNNNNNNNNNNNNNNNNNNNNNNNNNNNNNNNNNNNNNNNNNNNNNNNNNNNNNNNNNNNNNNNNNNN<br>NNNNNNNNNNNNNNNNNNNNNNNNNNNNNNNNNNNNNNNNNNNNNNNNNNNNNNNNNNNNNNNNNNNNNNNNNNNNNNNNNNN<br>NNNNNNNNNNNNNNNNNNAGCAGCCGACATACTCGCTGCTTGCAGCACTAGACCACAAGCCTCGGCCGTGGCTGTAGCTGCCGCGGC<br>TCTG
CsDof8	Csa02g073390.1	ATAAATCTCGTAAAGTTATTTTTTTTTTTTTTGTAAAATAAAAACTGGCAAATTACGGCTGGAATAAATGGGAACAGCACGTAAATCAG<br>GCGATATGCTTCTACGACGTTTTTGATAACGATATTACATTTGACCAATCTTTGATGGAAACTTCGTCTTTTACCTTAAGTAATTTTCTT<br>GTCATGTCTCTTTCTCACTTTTAACCTATCACACATAAATATATAACCTAATATTTTTAAACGCCGTAATAAATTTACATAGTTATTGTA<br>TTGTTGGATTTTTCAGCAGTATATCTCAGTTGTTAAAATTATAAATTCGCTAGTTTATATAGTAGTTTTTTTTTTTTTTTCCAATAGTTTA<br>TATAGTAGTTTTATTTCATTATTATAGCTAGCATGCATCTAGATTTTGTTAAAATTTTTTGTTGTTGTTGCATTGCATGTCTCAAGATTA<br>TTAAAGATTAACAAACAAAATCGGTGTTAGTTGTACATTTTGTGGATGATCGGTTTAGTCTCACGAGAGATCCATTCCTCAAGGGGCT<br>CTCACATGTTTCCCATTTTGTCTTCTCCGTTCTCTCGTGTTTCTTCACAGATGGAGACGTTTAATATATCAATCATCGGAACACAATCTC<br>CTCTCTTTTTCCATTTTTTTAATATAATATATACACACTTGTCTGTATTGGCCACATAACTAAAACCCAATTGAATCTGGCTTTGATGGC<br>AGTTTACTTTGAACAAATGTCAATTACTTTTAGCTATAACTGAAGGGTATATATTAGCGATACAGAAACATGTCAGAGAGTTAATCTA<br>ACGTTACTTTCTTACTAAACCCTACTTCTTCTTCTTAGGCCCTCTAGATTACAAATCTCAAATTAATTAACTTCCTTAAAAACATAA<br>CTTAGAAAAACTTCCTCAAATTTAATTATATATATTGACGATTTGGACTCATATAAAATCCAAAACTTTCAGCTAGCTTGTATGCACCC<br>TGAATCACGCCTACATAATACGTATTGATACTTATCCATTTACTCGATTCCTTGGCTAACGCAAAATTTCTAGTTCTTATATATGTTTTG<br>TTCCAATAGTTTTGGTTTCCTTAATAGAGTTGCATACGCTTGACAGCTATATATAAGTATAGCTGTATGTAAATGAAAGAAAATATTAT<br>TTGTGATTGTATATATAGTTTTTACGTATAAAGATGGTGGTGCAGTGCACACTTCACACATATAAAAAAACGTTACCTCAACAATAGA<br>AACAAGACACATAACAAATTCTATCCGATTCGTTCCGATCAACTGCACAGTTTTATATGTTCTTCTGTCTTTTCTGCCACTTCATGTCTC<br>TCTCTCATCTTCTCTTTTTGTCTCTATTATCTCTCACTCATGCCTCTCTCTCTCTCTCTCTCTCGCTCTTTATCTATATAAGTACAATGTA<br>CATATACAAACATTAACTACAAAGAGATTTTATGAAGAATTTAATTAAACAGTTTTAATAAAGGTGGCGGTTTACGATTATTCAAGTCT<br>ATAACTGTGCGAAGGAACAAGAACACCAAGACCAGAGTATACAAGTACAACAGAAATAATATTTGTATTCTCAATATGAAAACTATG<br>ATATACATGTTTTGACGCATTAGATTAATTTTATTATCCGTAATGATCATATCGAACACAATTATCACAGTTTTGATTAATAATAACAT<br>TTTCAAGACAAAATACATACTATAAATTTTTTAATTTATTTTTAAATATATAAAAAATGCCAAACAAAGTTTTCAAACATAAGCCCGAC<br>AGTATATATATCACCCATATACATATATGCTCACATATATATGATAATAATAATTTATCTATATAAATAAGTATAGAAGCATACGAAAC<br>CCTTTTACCGACTGTTGTTGTTGTCTCTCTCCTCTCATACTTTACTTATTACATTCAAAAGCAGGAACCCCCAAAGCTTTCTTCTTCCTT<br>TCATTTTTGCTCTCTCCTCCCCCATCATCATCATC
CsDof9	Csa02g075810.1	AGTTATATTCAGCTTTGAATATTTTTCATAATGGGATGTTTGGTGAAACATGCACCAACAAAACATAAAAAAGCCATATAATTAGGGC<br>CTTAAAATAAAGAACGATTATTGCATGATTTCTTTACACTGTCATGTCAGATTGCATATCATTTTCATACAATTATAGGGATCAGTTCT<br>CAAACAAAAAAAAAGAAAGAGGATAACTAGCCCAACAATGTTATTTACGTTTTTTGCCTCGTAAGTTTGCAATAATTCGGTTTTTGTAT<br>TGGAGGGTGTCTCACATCCACATCTAAATACCAAAAACACAAATCTCATTTGTAAGGCCCATGGTCCATAAGTCGCATACATCTTTAA<br>AAGAAAATAGGCCCATTAGAAATACGGACGGAATGACGAAAACACCTCCTTGGAAGGAAACAAAGAAAGAGAGTACTCTCAGACTAA<br>AAGGAGAGGAAAAGGACAAAAAGAATGGGATAAAGAAAGAGATGAGTGTCAGAATTTGACCAGATTAGACCGGTCGGTACAACAGA<br>GTAAGGACGGCTCTGAACCAGTGTAGACTACGTCTTGGGCACATACTTCTCACATGTCTCTCTCTCTCTCTCTCAAATGTTGGATGCTT<br>TTTGGTGTGAAAGGGAGTGAAGATTTGCATGCTTCTTAATTCTTTAGCTCTCCGTATACACTCTTTTTACCCGACAGCCTTCGCTTCTGT<br>CTCTTCACAAAACGAATCTCTTCTGTCTCTTTTGACCGTTTTAGCCTCCCAATTTTACATTTACAACCCTCTTCTTTCGTTTGGATTTGTC<br>TATTTCTCTTTCTCTCTCTATATCTTAAAGATACATTTGTCTTGTTTCCCCAAGATCATGTTTAGTCAGAACGATTATTGTTACTCCTGT<br>CTACTTGGTCTTCTTGTCTAGTTTNTTTTTTTTTTTTTTTTTTTTTTACTTTTTATAGCTCCTAAACAAGATAAGGTTTAAATGGCAAAAC<br>ATAGCATATAAAATAAAAGAAAACTTGGGTTTTGTAACATGCATAAGATTAGACCATAACACGTAACATTGATAATTTAAGTTGGTCT<br>AATAAACATACTGTGATTTATAACTTTTAGTTAGTGTTGTTTCTAAGGTCCTGAACGCATTGACTCTCGTATAGTAATTGATAGAGCAC<br>ATCTACACACACCATAAATCTCTTAGGTCCATATAATGGAGTAGTAATTTTTTTTTTCTTTTGGGGAAAAGATGACACAACTTAATAAC<br>CATCTTAAATAAGGAAATTAGGCTAGTTATTATAGCCATAAATAAACCCTTAACACTTAACAGTATAATGGTTGAATTATAGGGAACT<br>GCTTTACATCGATAAGCTCAATAGCCTCTCCCACAATTTGCCTATTGTTGCCTACTCTCTTCACTCTTCATTTCCCGTAGACACGTCCTT<br>TTGACAGACGTTATTTTTACCATATTTTTTTTATTTAAAACTAATGTCATCATCCCACTGTTGTTTGTTTGTTTCAACTCATAATAACTC<br>GGAAGTACTAGCTAACAACAAAATCAAAACTTAATTTACTTTAATAGGGTTTTCTAGTGACGCACGAAGCATTTCTGATAATCATGCA<br>AATAATGTCTTACTCTTTACTTAAATCGCTTATAATGCCAAACAAAAACGTGCGACAAATTATGGATTGAGGAGATTAGATGTTTTGTT<br>TGTTTATCCATAATTTTCAAAACAGAAACTCTTGAAAACAGAGCCACCTACGTACAGTATAACAAAGCGAGAGAGAGAGAGAGAGAG<br>AGAGAGAGAGAGAGAGAGAGAGAGAGAGAGAGAGCATATCTCTTTCTATGTTCACAATAAACCCAACACACTCTGTCAAAAAGCTTC<br>ACTCTCTTCTCCTTCCCCATCTCTCTCTCTTCTCCATAAAAGGAACCTCTTAACTCTCAGAAAGTTCCAAAACCTCTAACACTTCTAAGC<br>TTCAAAAACTCTCAACGTTTTCAGCGAATTACCTACCTCTCTTCAAC

Name	Gene ID	Promoter sequence
CsDof10	Csa03g011080.1	TATATCACAATTTTGGTCTTGCGTATATGTTTAAGATCGTTCGTAGAGCTGTTTTATTTTATACTATTTTTTAGGTAATTTTGGTTAGAA<br>AAATGTTCTCTTGTTGTATTTATATTGATGGCGTATATAGTCTTTAATATACGTAAAAGTGTCAGTGATGATATCAACATTTAGGGTTA<br>CGTGAAACACAAATTCCAAGGAAAAAGCCGGAAATATCTCAAAAATGACAATTTATCCAATCTAAAGGGCGAAAGGAGGAAGAAGAA<br>CGATAAGAGTGTACATGATGGTTTTGGTATATAAAAAGGTATTCGTGATGGGTTCATGCATGAACGTCCTTTTATTTTTTTCGTTTATT<br>GAGATTTTTTTTAGACTATTCGCGGTGGAGAGATTTGCCCCACTGGAAGCAGTGTGTTATAATAATGTAGCAAGTAGGCTATCTTTGC<br>CGGTCCCTTTCCGAAAAGGAAGTTTAGCAATTTATAAACGAAACGAGTGATCAATGAAGAGATAAGAGAAAAAAAGAAAGACTAAAG<br>TAGAGAAAATGTCGAGACAAACTGCAAAATGGAGAGCTCTTTTAACAAGTAGTACGTTTTGTGACATAATATTTTGGACCAATAAATA<br>AAAGTTGTAATTAATCGGAAACGAGCGACTCTTTCTTCTCTTTAAAAATCAAACTTCATTATCTACTAGAAGAAGTAAGACCGGCGGT<br>CATGTTGTCTTCTAGTACTCTTGAGTTTTCATAAAATAGGAAAAAATATACTGAAATAATTACGAACGACTCATGTTATTTATTTAGCT<br>TTTACGCTTATTTATGCTACTGAATGCAAATTTTCTTTAATTTATACCATTGCATAATAGACAAGATCCCACAAAAGTGTAATAGGGTA<br>TGATGATGAGATACTTTATTTATGGCATATGGGGCTTGCCATCTACATTATAAAGAATAATTGTAGAAAATAATTTATTTTCGTATCTT<br>AATTTGCCCCAATATAATACATCCATCTTTTTCATTAGGCACTAATTCGTTTCTATTTGGCTAATATCAAAGATGTCTCTATAGAGAGG<br>CACGTACGTGAGAAAAAAGAGAAAGATAGGGAAGAGAGAAGTTGAGTGTTCGGCGTTGGTGAGCTTCCATTATAATGAACAAAACGG<br>CGCAGAATTGGGATTAAGATGATCAGAGTTAATTATATGAGTTTGATTTTTTAAGATTTCCGAGTTTTGATGAAATCTATGACAGGCCA<br>GCGATTACTGTTTAGAATGTTGGAATTAGTATAGATTTTGTAATTTGCATGGTTTATATAATTATTAAAAAAAAATTAATCGACTACGAA<br>AAAAATGAGAAAACCAACAATATTATTGTAGGATAAATACGTAAATGTTTCTAAAAGTTGTAAAAGTACCTCGAAAGATTTTTAAAAT<br>TTATATTAGAAAATTTAGTTAAAAAATTGTTATATTACAAAATATATATTTAACGTTTCTTCAGATTAAAAAAGTTATACAACTTCGTA<br>TAGTTTCGTCGAACGTTTCTTCTATATATATTATTCCATAGACGCCTAGGAGAGGAACCTTATAGGCGAAGGAAAACGTCCCCACTCA<br>AACCCTAAAAATGGGAATCAGAAATAAAAAATAACACACAAATGAAGTATAGAGACGCGTATACACATGAGGATATGAGAGTGGAAT<br>TATAGAAATATATAGAAAGCATAAAAAGAACCGAAATCAAACTCCTTTTCTTTTTTTTTTTCTCATTAAAAAAATGCATTTTCTTGCCTT<br>TAATTCATTTGTCTTTGTTCTCCTTCTCTCTTCTCACTTTCCTTTCTGATTTTGTGCTCTCTTAGCGTTTCTCTTTGGTCGTCTTGCTTCCT<br>CTCTTTCGTTTTGCCTTTGTTTGTTTCTTTTTTTTTTGATTCCCCCACCAATTTTTTCCTTGGAGTTCTTTCCCCTTAAAGATCCATCAAA<br>CTTTATTAAAGAAGGTAGAAGAAAGAGATAATAAAGGGTAGAAGAACA
CsDof11	Csa03g025140.1	TGATTAATTGTCGCAATTCATTTATACGCTAAGAATGTAGAAAATTACTAGTAATATCTAGATAGCGAATTAACATGAAAATCAGTATT<br>GATTGGTAGAATAAATTCACAACTATGATATTTAATGATCTTCATGTTGATTTCCCAACCCAAAAACTTTTATGCTAACAAAATACTAG<br>TTCTTTTTTTTTTTTTTTTTTTTTTTTTTTTTTTTTTTNTTTTAAGTATTAGAGTAATTACATAAGCTTTTTTTCCCTTTATACATTATTAA<br>GTTGCATACTTCACACTTGTAACAAGTTATATATTTTTTTGTTTATATCGAATTAATATTTTATAATTAAAAGTCAAGCAACTTCGAGTC<br>AAATAGGAATTAATAACACAGCTGCGAATTAATAAATGCCAATCCGAAAGAAAAAAAATTAACGTTTGACTTCGTATTATTCACAATT<br>TTGTAGGTATTTTTAATCATTAGACTAGGGATATCGTATCGTAAACACAAAATAGTATTGGGATCGGAGCTTACCATAGATCTAGTTTT<br>AAGGCTGCCCAATCAAAAAACTAATCGGTGGTGATCAACAATGATAAATTAACAGTCAAGTGTTATAATAATGATAAAGTCCTAAAAA<br>TAATAAATACATTAGAGAATTCAACTTGTGGGACTAAGAAAGAGAGATTTCAACTTCGTAGATTTGAAGGAATTGAGAGAAATAAGA<br>GAACCATAGGTCGACACGAGGGTAGAAGCACGTGTTCTCCCGTGCGTGTGGCCCTTAATCTTGGCGTCAATCTATCTTAATCTTACTAT<br>ATTATCATGGTTACATAATCTATGCTCTTAACGGTCACTAATCTACATTTGTACTTGAATCCAACAAATGAACATTTTGGCTAGTAATG<br>AAAATCATACTAATTGACGGATCATATTACTCATTAAACCCAAATATATAGACAAAACCTATCAAACAAGTTTATATATGAAAATATA<br>GTTGAACTGATCGGACATATGTATATATTTTATTGCCATGTCTATATTAGTTACGCAAGTTTATTAGATTTGAGGGTATTAACTATTAA<br>GTATTAACATGGTCAAGGGATAAACAAAATGACAATAATAATAATAATGTACCGACGCTTAAAAGTGTTAAAATGATACTTATTTAGA<br>ATGAGTGCAAGTGATCAAATGTTACGATGCTTTGTTTAATAAATCCAGTAGTGTCTTGCGGTTCACGAGAATAAGATGCTGAGAATGT<br>AAGTGATTAATCATCTACACACTAATATAATATGATGCCCCTAAGTTAATATTTTAGAAAAGAGGCTAATTATAGTAATTTACTTACAT<br>TCAAGGTTAGGTCAAGCAGACCAAAAAAAAAAGAATTACTTTAACTATTTTTTTTTTTTTTTTTTTTTTTTTTTTNTCCTACAATAGATATC<br>TATCGCGCTTGTCCAGTGTAATCACCCATTTCAAATCTAGATGAACTAGTGGATTTAAAAAAACACTTGAGCAACCTTGTTATAACAAT<br>ATTTTTATGTAGAGATTTACCATGACTGTTAACTTGAAGAAAATGATGGCACAAATGCACATACTTTACATTATTTTAATTTGCTCTTG<br>GATGGTATGAAACATAAATTACAAGTTGCATCAACAAAAACTTTCAGAAATTCATCTAACCGTTTATTAAAACTTGGATTATTTAATAA<br>ACATTTTATTGGCATTATATAAGTAAAAACTATTACGAAGGACCAAATTCGGAGGTAACTTGATGAAGAAGAAACGGAAAAGGAGAC<br>GCACGGCTCGGCAACCGCCCACGTTTACTTCGCAAGTCTTCACACATTAATCGATGCACCTCTGCTTCCACACACACATGCTCTTCCCC<br>AACTCTGCGTTCACACAATCCCATATATATTAGAGTTAGATGTACTCGAATATACCACCAAGTGCCCACTTGTGACTTGTTAGATTTTC<br>CAATCTTCTTTACACACCAAAAGTTCATAAAAAGAGTACATGAAAA
CsDof12	Csa03g028730.1	CATTTTATATTTATCATAGTGTTGACTAATCTAATATATTTGTTGCATGGTACTATGGGGACCATGATGAATATTCATACGAATTTGGC<br>TTGTTTTACTTCTTCCGGATCTACGTGAATAATCAGTGAACAATACCAAATAAACAATACCGTGAATCCTTATCAAAATATGTCTTTGT<br>CAATTGTCATTAAGGTCTTGATGAATGTGTCTTTGACGTGAATTCAGAACTTCATGATTATCACAACCGAAACTAAAAATATGTAACCA<br>CAAACTCAAACACCAAATTATTTTTAATTTATTATTACTAAACAAATGAAAGGAAAATTACAAAATATTATCTTAAATAATTTGAAGTA<br>ACTAAGAAGCCCATGGAAAACACTGAGGCAGGCCGCCATCACGTCTAATACGACATGGACCTGCCTCGGTAATATACCAAATACATTC<br>TTGGCAGACTCCAGATACACCTTTTGGATTGTCATCTCTATCTGCTCTAAAAATATCAAACGTAGTTGTGCTGTTGGGCCACTCGAAAC<br>TGCATGTATACGTTGTTGTTTTACGGAGATTGACACGAAACCTGAAATCAAAACTATCTTTCCAGTTGAGTCTTATTACTCCTAAATCA<br>TCCCCTTTGTTTCTACAATGCAAAATCAACATTGCATGTGAGACCAATTTGTTAACAATAGTAACATGTTTAGCTGAAAATGGTAAAAG<br>TCCATCGGTGGACGAATGGTTTTCTAATGATGCCGACGTTTTGAGAACAAAAAGAAATAAGAATAGCACGGAAATGAAAATTTGATTT<br>GTTGAAGAGGCCATGGTTGTGGTTTAGTTTAAGAGATTGTGAGAAAGCTTGGATTTATAGATGAAACTACATTTATTTGGCTTAATGT<br>TTTTACGAAAAAATATCTTCTAAGATAAATGTGAGAATCTTTAGTTTGTAACTGATACAACATACGTAAAAAAAGAAAGATATTGACT<br>GGTTTCTAAATTATTATTATTCTTTGGATATAAAGATGGTCAAATTCATGGAGTTCCCTATTTTCTTCCTATAAACTCTGATTTTGTTAT<br>TTAATAAGCAATTTCTTCCATAGGTATGTATCTAATTTTCATAATGATCATAAAATTTTATATTTATAGCAGCAGATAATTATTTAAAGT<br>TACTTATTTTGTTATTAATATCATAGTGTAATAGGGGAATCTGCTTAGTTTAAAGGTAAATAATTTATATATTACCTACTAAAACCATC<br>AGATTAATTAGGTTCAATTCATAATTCATGAACCATGCATGGGAAAAAAGTTTCAATGTATTAGATTATGGATTTTGTTGGGTTGGTCT<br>ATTTCACACTTTTATTTTAGAATAACATTTAATTCATTATTCATATATATAAGTCTATTACCTCTTACAATCTAATTTGTATTATCTTCG<br>GAAATTCAAAGTAAAAAGAACTCAAACTGTATTACTATTTGGTATTAACAACCCAATTTAATCCTGCCATTGAATTGACTCTAACATAT<br>ACTATAAGTCTATAACGTATGTGATACCAACTTTTAAATTACGTATAGCAGCTGGCGTTTTTTACAACAAATGTTTTCGCACTTTTGTA<br>TTGTTTAATACGAATATTCCAAGAAAAAACAAAAAAAAAAAAAGAAGAAGATTGTACTACATTCTAATGGCCAAACAAATTTTGACGTG<br>GCAAAAAGTAAAGGGAGCCAAATCTTTTGAGTGGCACTGAAAGAGAGATCAAAAAGAGCAATGAAAAAGAGATAGCAAAAGCTCAC<br>TTTTTAGCAACAGCATGTGGCTATCTCTTCTCTACATGTCCCTGTCTCTGCTTACGTGTAATTTTCTCCTCTTTGTGTCTTGTGGCTCTG<br>TGCGTTCCATTATTAATTTAATACCAACCTCAGCCTCTTCTTATACATAGTCTTGTATCTGACCTCAAATAAGGAATAAACACACATAA<br>CATCAAATCTAATCTCCTCTGCTTCATCTTTCTTTTTTCTGTCTTT

Name	Gene ID	Promoter sequence
CsDof13	Csa03g032300.1	AAAAGTACTTAAAAGTTGTTCTTTTGCGACTATATAGTATAAAGTTACCAATGAGAAAAAACATATAGTATAAAAAAAAGCACGAACT<br>TGAGAAAAAAAATAAAGAGAAACATGAACATCGATTAGTGCTTTCCTTTTCCTTTTTTCTTTCTTTTTCTTTTTTCCCTTTCTCTCATCTT<br>TAACGCTTTAGAGTTTAATTTAAATAACGTACGTGAATTATGTTAATTTAAATAAAATATTTAAAGAAACATGCTTTGCTTAACTTTAT<br>TTGATTTTTTTTCCAAGGCAATATCCACTCAACCGTTCTACTAGTTGATCAAGCAGAATTGCACAAATTATTAGTCAAATTTCACCACT<br>ATCCACGGACCATGACATGCAAGAAGGGATAGATGTTCCATTTAAGTTTAACATAGTCTTGTTAGTGCAGCTAGAACTATATTTTTGC<br>ATATACTATTATTATTTTATCCAAGTTCACAATTAAAAAAAAAAAAAGAGAAAAAAGAGAAATTCTCGATTAAATTTCAGTTTTCCCTTT<br>ATTATTTCATCCAAAGAAACTCTTATACTACCAAGAAAAAGAAGAAACTAAAATTTTCCTAATGAAATTTATTTTTCTATACAATACAC<br>AATGAAATAAATAAATGAAGAAAATGGAAGGATGACTTGGAAAAAGGGGGGCCAAAAAGGATGAATTGAAAGCTGATCCCCTCTAGT<br>CATTCAGGCTGCTTATTCATCTCTTCTCCTTTTCTACGCCAACACACCAAACGCTCTTTCTCTTTGGCGGCTTTATCCAAATCTCACTTA<br>CATAATCTTTGCAATCGCATAAGTTTTTTTTCCCTAATTTCTTATGTTAATTATACTTTGTAAAGATTTTCATATAATTTTCTTGGTCTAT<br>GATCTAAACCTTACATTTTAATCTCATATTGAAGAATTGAGGTCGAACCCCTTACACCCAAGCGGTAAAGCAGAAGTGAGAAGTCACC<br>ACTCAAGTTGCTCTCTCGACTCATATATACTTCCAAATATTTATAATTCCAAAAATGTTAGTCAACGATGAAAATAAGAAGAAATAGAT<br>GATGCTGTTGCGAATTTTAAAAGGATAAATAGTATATCATATAGATAACATAATTAGGTTTTGAATGTGATATTTGTTTTGTTTCTGTG<br>ACACAACCCCAATTCAGAAAATATTAAGAACGCACGTACCCCTATCCAAATCACGTTGTCGCACAAAAGTATCAATTTTAAAATATTC<br>AATAATTAATATTGAAACATACATTTTGTGAATTAGTTAGTTATATAAAGAAATATTTAGATAGCTTGTAAATTAATTATAGTAGGTTT<br>GCTTTAACTCGTTTGATGCAACGAAACAAAGCTCATTCTCTGCATATGAAATGATGTTATGAATTTTTGCATGTTTTAGCTAACAACTG<br>AAACAATATCAAATAGTACGAAAGGAAAGCCCGGCTCCAGTCCAAGTTGTTCAACAATAGTGGCTACTAGTGACTTAGAAATAGTATT<br>AGCAAAAGGAGTAAATTCAAAACCAAATATATAATTCACATAAATTCTGATAAACAGTCATGTAAATAAATAAAAAGTGTTAAAACAT<br>CACTATTCCAAAATACCTTCTATAAAATATACGGAAAGACTATAAAGAATGTACTGAAAAAGTTTGAAAAATACACACATTATGGTGA<br>AAAAGCAAAAGAATACAACACATTTTGTCAGCTGATATATATGGATCGATATTTCCTTGAATGCAATAAGCAGTAAATGCGCCTAACA<br>TATTCTTTCATAATATTTTTATATTCTCCCTTACTACAACACTATTGCTTTTCTCTCCTTTCCTACGAAAAAGATATAATAATGTTTTCAT<br>TTTCATACAAATCTTTTTTACAAAAAAGATTGAAATCTTCTTCTTATAAACGTCTCTTCTTCTCTCTTTCATCACTGCAAGAAACTTAAA<br>TCACAACTCTCTCTTCTCTCTCCCTTACTCTCTCTCTCATTTTA
CsDof14	Csa03g051350.1	AAACTCATTGAGAGAAGTCTAGAAATGGTTGAATGCTCCAATGGAATTTTGACATGGAAATTTCTCCTTCAGTAATCATAAAAGAATA<br>GTTTATTTTCTATATACCAGACCACCATCAAATCATCGAGTTTTAACATGTATATAGAATGTTACATCCGAAGCTAACAATATTAGTGT<br>AGCAGCAGAGATCCACTTACCAAGTCCAACAGCCTGAAACTTATGGTTTTGGGAGATGTTCCTGTGTGTCAATTGGGCCAGAGCATAA<br>TATAGACCAGCAACATCAATGAAGCCAATCATTGCTTTCAATGCTTCCTAATACAAGGGAGATTAAACCCGTGCTTAGTCATCTAGATT<br>GTAACATAACAAAACAAGCTAAGCAGTTACAGTAGAAGTTGATAGCAAAAAGAAATTCAACAATGTGCATCTATATAATAAGAAAAG<br>ATAAGTCTGGTTAACATTGTATTATTTGCATATCATTTTACTTAAGAGTAGCAATCTTTCAACAGAGAAATAAACAACAGACTCATTAA<br>AACAAGTGTGAGTGCTACAGTGATCTTAGCTTAGCTACAAGTAAAAATACCTGGTATGGGTCAAATACTTCAGACTCAGATACTTGCA<br>GAAAAGTTGCCAAACAAACAAGCTGCCACGAAAAAACAACCCAATGAGCTGAGAATTAAGTTGCAGTTGCAGATACAAATCAAACAA<br>AGGAAAGATTTGCACAAAGATTGTATACAGTTGACTAACCTTAACCAAAGCAGTAGCAAGGTAAACAACAGCAGCCTTCACGGAGGT<br>TCCAAGTGTGTCATATTCAGATCTGCAGATTCACTTTCCGATTAGAAACTACAATACCAAAACTTATAACTCTCTAATCCTAAATTCAA<br>TTTGGGAATTCTCAGTTCCGCAGAAGCTCATACTAAACCTTAAGATTCTAGATCACGAAGTTTAGAAGTAAGCGATTAAGCATATGCG<br>CAAGAGGATCTAGATACAATCTTAGATTAGGTATGAATAAACTAAGAGATACTAAAATTTGAAACCACAGATCGAGAAAAAAGGAGA<br>GGATTTGTTCATACAAAGGAGTGGCAGAGTAGTAGACGGCATGAGGACCGAATGTGAGAATCGCACAGTTGAAGAAGTGAAAAAGCG<br>TCATCGTCTCTCTTTGTGACACAAAATCGGGAGCTACACGAGTCCGGCGACGCAAAAGACGACGACCACCACGACGTGAAGCTGTGA<br>AATTGAAGAAGAAGAGGTTTGGTTTAGCTGAGAAAATCTGAAAAATCTGATGGGATCAACAAAAGTCTTAATGGGTTCCGAATTGGG<br>TCTCTCTATTGGGCCGCGCAATTTTTAGTCATAGATATGGTTATGTAAGGTTATATGATGATGTTTATCATTTGATAAATCCATACTGT<br>ATTTGATTTCTTTGTGGTAAAATTATATTTGAACTTTTTTTCTTCTGAAATTATAAGTTCATATGAATGCTTATGCATTTTCTCATATTT<br>GATATATGTATTCCTCTCTTTTTAAATTATAATATTTTCTTAAAATTTTAAATATAGTTTGAGCTTTTCGAATTTTTTTTCTTAAGTTGAT<br>GGTAGTATTTTGACTATCTAGTAATCTTATAAACGACTAAACCCGTTTTAGATTGTCTCTATCCTGATTAAATATTGTAGGTGCAAAAT<br>TTCAGACTATTTAAGTGTATTGTTATGAAGGAATTGACCCTAAACAAAAATCAAATTATAAATTGTATCCAAAATCCACTAGCAACCAT<br>TTTTCAATTAATCTATCAACTAAAAGTTTAAAACTTTCAAAATTAAAAAGAACAAATAAAAATGATACTAAATATATCAAAAGAGTAA<br>CTGAAGAAACGTCAAAGGCCTCTCTAATTCTCTATATAAAAACCACAACACCACTCTCACAACATCCCCTTCTCTTTCGCTATCTCTCT<br>TCTTCCTTCTCTCCCAATTAAAACTTCTCTGTCGTTTTCTCATAAACCTCAACTTATA
CsDof15	Csa03g058660.1	AGCTAATCCCATATGACTTTTGGATGGATATAGTTTAGATTTTATTATAATCTTTTCTGGCTCTTTGTGTAATCATTGTTTTATTGATTC<br>TTTTGTTAAACTTGTTTCGATTTTCTTGACTTGTTAAAATAATACATTTCATTTACATATACATGTTCTTCTTACGTATTTAGTTATTTGG<br>CCATTTCATTTTCATTTAGTTATTTTGTTATTATTTATTTGGATTTAATAAAGGATTATTTATTTCACCAAACAAGGTTATTAAGATTTA<br>ACAGAAAGTGTTTGCTGCCTTGTTTATGCATGTTTACTGAATTTCCTTGTGAATTAGGACCTCCGATATCGAAACGAATTATAAACAAA<br>AACGAATAAGGTCAATACGCGTCATTGTGAAAACTGTCTCTTGTGGCTAAGACTTAAGTAACCTGAGCCATCCATTTGTCGTCCTAGA<br>GAGCCACAAATGGTAGCCCGCACAAAAACGATCAGTGATAACGGAAGTCGGCAGCGTAAGGCGATCGTCAAACCTGGTTTCGACACT<br>TGTGTGTGGTGTCTCACGGTTCAATGTATTCACGATAGCATGTACAAAAAAAAACTGACGGTGCTGATTCAATGTTCCATGGTGGGGTT<br>CCAAGTGTAATGATGCCTTTTTTTTTATTAGTATAGATTATTCCTTTTTCATGATCCAACGAAATAATAAGACTTTGCTTGCTACGTACG<br>CTATCTTTTACATGTTTTTTTGTCTGCTAATTCGGAAGCCTTAGTGGGTCAGAGAGCATCATAGCTCTTGAGTTTGTGCGTGCCCTAGT<br>TGATTTAAAATTTGGGACAAATCAAAAAGTCTCTAAACTTACCTGTTTCTATCAAATTACAAAAACTTTATGTTTAACCCTATTAATAA<br>TTAACGTTTAACAAATCTATTTTAATCAAACGAATAACGTTTTGAACATAACTCGAAATCACTAATTTTAAAATCAAAAGGCCTCAAAA<br>TTGGAGTCAGATTTTCATATTCTGAGTACTTAACAACATACCGAAAGTTATCAGGTGAACCTAACTTGGAAGTTTTTTAGATGAAATTT<br>ATGTAGCTCTTTGAACATTCATGGCTCAAAGCTAAAAAGCATAATATGACAGACAGTTCGATTGCCTTGAACATATACTATTAGAATTT<br>GAGCTCGACTATTAGGTTTCAAGTTCAACTAGTATAAGTTTATTTTCTTGTGGACTACGAATGAAATAAAAATGATAAAAGTTATAAGT<br>CAAGGGAACAAAACTCTACGATAGTATTAGTTAAGTCAACAGGTCAATGGTAAAGTTCAACGCGTCGAATCTTGTCAAAGAAAACTAT<br>TGAAGGAAATTTCTAAGAAAGTTCGCTTTGGTTTTATATATTTTTTGAAAAGTTATTGTATCCATAAAAAAACATAACAAAGTATTTAA<br>ATTATATTGAAAATTTTTAAAATTTATTTTAAAATGTTATATATAAAACAAAATTACGCAAGAGAAAAAAAAAAGGTTATTAAAAATCA<br>ACAAAACCATGACTTTTGAAAAACAAAGTAGAAAATTAATAGCAAACTTACAAAATAGAGAAACACAAAGGTCGGCTGCTGTCACCA<br>CAAAGAAGCAAATAAAAAAAACACAGAGGTCAACAACAAGAAATATAGAAAATCATGAAAATCTTCAATAACACAAAAGTCTCTACT<br>ATAGTACACCCCAAAAAAAAAAATATAAAAAAAAAATCAAGAAAGTTTTGAAAAATTGTCATTTTGTGGGTAAAAATAAAATAAAAG<br>AAGAAGAAAACTCTTATTAGTTACCCCACCACCAAATAGAGCTCCACTCTTTATCTCTCTTCTCTCTATAACTTACAACTTCTTCCCCCA<br>AATTCAAGAATCCATTTTTTTTTTGTTGTTGTTGTTTCCTCTCTTTTTTAAAGGTCTCTCTGTGCACAACCCAAAACAAAAACATTTTCT<br>TGTAATTAAGTCAGAGAAGAAAAAAAAAAAAGGGGAAGAAAAAAAAAA

Name	Gene ID	Promoter sequence
CsDof16	Csa04g026300.1	TTACAAAACGTAAACTAGTACATGCAAGAATTTTAAAATAATGGACGCAAAAGTCATGAACCACATGTGCTGCAGACACATAGATATT<br>TGTGGACCTCGGCTCCTGATCAGTCCTCACAGAGAAAATGTGGTCCAGGAAATGATTAATCATGCACCAACTATAGTTATGTAGTATT<br>ATTCTACCCTTTCAAGTTTCACCCAACATTTTGGAGAGTAAAATATTAAACTTAACTGTGACTAAACTAAAAAAGAAAAAGAAAAAAT<br>TAAAAAAGGAGAAGAAAATAGAGAAATATTCTACTTTGTCACACTTAGGACCAGCTTGAGGGAGTGGTGGGATTCACGCGAGAGTAT<br>AAATTTTTGACTAACTTAGAAGATGAAGGTGGGTTCCGCGTGGTGACAACTCAAGAAGAATTGTGACACAAAAGAAGAGATCAAAGA<br>CACATGAAGATGGGTGCCTCAGAAATGGACGTCGTACAAACAACCAATTCTGATACATCATAGTACGGTCCTCGATCACACTTCTCAT<br>CATTTCTACAGAAACAAATCTGACGCCAAAGATATATGTGTATATATATATATATAAACACATATAATATTTAGTAATTGCATATTGTT<br>ATTGGCTCATTGTGTTAAGAATCCCACAAATTTATTATTATCTTAGAAACCCTATATAAGGATCACAATAGTTTAGTTTTTGTATTTAG<br>AAAGTTTCGAACAAAATCCAAAATAGATGCAAACACATATCTTATGACTATTTCTCATGTACATCACGAGATTCACGACCACATTCGA<br>ATTCCAATATGATGAAACGTAAAACTATTTTTTGATCCGAGAGTACTAACTTCGTTTGGTAGTTTCAAACAATATTTCCCACATCATCA<br>CATGTAATGTACTCAATATATAAAATTTGTACATGATAGAACAATCTATATCAACATGTTTTTGTAAATTTACGGAACACATAGTTATA<br>TTGTAAAGTGAAGGATGTGGCTCCATCACAATTATAAAAAAAATCAACGTGTGATGATAATGAAAAATCTTCTTCGCTCACAAGATCT<br>CGATATCGCAATTATATACATATATATAGAGATTTTATTATTAATATGATACCATTGAAGAGCCGATGAACAAGCATAATTTAATTAGA<br>GACAACTAGTCTTAATATCAATGTTCGGTCGGACCGGTCCAACCGCGACTGGTAGACATTCCGGTCTGGGTTACCTCTAAAAACTGGA<br>CAACTAAAAATTCACAAAAAACGGAAAACTCCACTAAAAACCCGTGAACCGGTGGTTGAACCGGCGAATCGGACCAACGAGTCAGTA<br>TCAAATTAAAATATTTTATTTTTATCACATTTCAAACTCAAACAAAATTAAAATTAAATGTTGTTATTTATAGAAAAATCACTTAGTTAT<br>TTTATCTAATCATTTAAAAATGTAATTTTAGTTCTTTAGTACTTCATTATGTATCTTTTGTTCATAGATTTATAGAAAAGTAGATGTTAT<br>ATTTAGAAAATATAAACAAACCAGTAAACTAATTTACCCGTTGTTCAATTGGTCTGACCGATTGACCCAGTGACCCGAAAGATTTCTG<br>GTTCACCTTTCAGTCCGATATTAAAAAAAATGCTTAATATACTTTATATATCCAATCTAAATGAAAATTTAAAAACAGGGTCTAGCTGT<br>AAATCAGAAGTAGGCATGATAAATCTTTATTTTTAAAAACGGCTTCATGGGTTCACATCTCATGCTAAGAGTTGGATCACGTTATTCCC<br>TCATACGGTAATAAACATTTATTAGTAACGTTAAAACATTGAAACAATATAATAATATTGAGGTAAGCCACCAAATTGTCTAGCTACTT<br>CCACATTATTTTTCTATATATAGAGAGATAAGATGCTTGGCTTTTCCTTTATCTCCACTCTCCTTTTCTCCTTTATCTCTCTTCGCTTTAT<br>CTCAGTCTCAATCTTCTCTACATTTCGAAGAAGCTTCTCAATTAGTTGAGAT
CsDof17	Csa04g030380.1	ATTTAAATATTTTACAGGGTTTCAACAGATTTCTGTGTTGACCAATGAAAACAAAAACACTTGTATTTTATAAACACATCCATTTTATA<br>GTTTTTTTTTTTTTTCTCTTTTCCTAATAAACTGTAAAATGAACCAAAAAAAGGGTGATGTCAGAGGAACACGTGAAGGGTTGTGGTTA<br>GATCTTGAGACTTGAGCAAGAGAAAAAGACATGCATCGATCGATCTTTTTGGGTTGATGAAATCCATGAGCAGGTGTGACAGAATCCA<br>ACGTGGCAAAGCACAGACTTTGATTTAGATCCTCCATAGTCCAAATACCTCAACTTGGTTAAATTTGTACAATGTGGCAGTGAGACAT<br>ACCTACATAACCTTTTTGTTAACAACCAATTCTCTATCGACACGTCACTAAATCTTACCTTTCTTTGCTTTTGCCCTTATCCAACCAAAC<br>GTACCAAATCATTCCTTCAATCTCTTTATTTGAGGATTTTGTTTTCCATTCTGGAACTGGAAATTATGTGTAAAGAGATTTCTATATTAT<br>ATTTTCTATAAAACTGAATTTAGATATATAGAAATAATTTATTTTTAAAAAGTAAAGTATTCGATAGATGTGACTCTAAGAGATGTAAT<br>TTTAGAGAAGGTTATTAATTTTATGAATTTAATAGATTTGGAAATCCAAATAAAAATCATATATTATTGGTATTATGATTTAAATATAC<br>TTGTTAAAATTCTAGATTATTCATTTAATTATTTGTTTTTGGATTTCAAAATCCACATTGTGTTTTAAATGGATTTGAAAGTGTAATATA<br>AAAGAATTCAAATTTAAGGATAAAACATGTTATTTCAAAATCCATGTGTTTTGATTTTCATAATTTACAATTCTATATGGGTTTAGAAA<br>TCATTTCTCCAATAACAACTCTACTATTAGTACCATCACAATTAGGTTTGTGATACTAGTATGTTATTTTAATTTATTACTGATCTTTGT<br>ATGATTTATATTAATAAATGGATAAACTTAACTTAATTATCTGAATACTGATCTTTGTATGATGTTGATGTATTGCTCAAATTTTTCTTT<br>TTCTTATATCAACTGCATTCATTGGTTTAAAAAGAACAAAATATCAACAAATCACAAACTATAACAAGCTAACAAATGAGATTACATG<br>GGACCTGATGAAGGGAGAGATAAGTAATGCACTAAAATACAATACCAATTCTCCAAATCTATGTTTTTGAATTCTTTGATGTTATAAC<br>ATTTGTACAAAAGATGTATGATTGTGAAGCTTTCAAAATAAAATTTTTAACATACTTTTGAACTCTGTTTTGCCATGTTGAAATGCTTT<br>TATTTTTATTTTCTATTAGATATAATGTCTCCCTAAAAAGTTTTCTCCATTGTTGAAAAAATCTTGCATCTAAATATATGATGATCATGT<br>GTAAATCAAATGAAGTAGAAGATTCAGTTGTTATCTATTAGAAAGCTTGTATTCAAAATATTCTTTTGTCGTTATGTTTAAAAAACCAT<br>ATTTATGATTTTGTGTTATGGGTTTTCAAAAAAAATTAATTTATATAATAAGATTACTTAATTAAAAAAAAAGTTTATCTTGTATATTT<br>GTAATCCCATTTATATCAGAAACACTTTTGGATAAAAAAAAACTACTATTATTGATACGAATAAAGTTAGAAATTATTACTTGGGACTT<br>ATTATTATTATTATAATACAATAAATATATTTTAGAAAAAAGAACCCGACAACCCGATCCGGTAGTAATGAGGGAAAAGGAAAATATC<br>AGAGGCCACATAAACTTGTGGCTGAGAATACAAATGTGATGCTGACGTGTTACCCTCCTTCTCTTTTGTACGGATTCTCCCCTTAATCC<br>CTAATTTTCATATCTATTCACACTCCTCCCAAACTAAATCTTCCCCAACAACAACAAAAAAAAAGATTGACTAGTGACTACACGAAGCT<br>TTCTCGCGCCTTTGCTTTTTGGTTGAGAAAAAAAAAAAAAA
CsDof18	Csa04g036060.1	ATCCTACTACTCTAGATCATACACTCATTTGTCATACCCTCAAGTTGTTCAAATTGTCATGCTCATCTTATATGTTTTCCATATGTAACA<br>AAATGTAAAAAACGAGATCATTTAGTTTAAAACGGATGAGTATCTTATGAACAACTTTTTTTTATTTGTATTTTTACTACTTATTTTAGA<br>AAAGGTTATATGTTTGTTTTCGTCCTATGATGTTTACTTTAAAATCTTAGTATAATAAAATATATATTGCTTAGATTTAGTGTGTTTATT<br>GTGGTCCTTGAGAGATCAAACTCCACTACAAATTTGGTTGTTGTATATTGACTAAGATAGATTCTGATTCGGTTGTCATAATCAAATCA<br>ACGTAATAATTTCTAACGCGAATGTAGTTATATAGTTTGATCAATGATCATTCATAGTAATACAAATTTTGGTGTTTTTAGTGATGGAA<br>ACACGAGCATAGTCCCCACCGCCCGCCGCCACTACCGTTTTAAATTTAAAAATAACGATTTTTTTTTATAAACGACTGTCACCTGTCTA<br>ATATAGTTTGGATTCAAATTTGTAAAAGTATTCGAAAGAGAACAATCTATAAATAGAGTAATAAACAAACAAAAGGTTTCTGGTACAT<br>TACAATATAGTATTGTCTTTTCTAAGACAGCGAACAAATGAAACAAGATCATTTTTTTTGGACTTTAATTTCAAAAGCCTGCGTGATTG<br>ATCCAACAAATTATAAAACAATACTTTAATTTTCGTTCTCAAATAAAGTAATACAATAAATCCAATTATCCATTTTTCAAATATTTTTTT<br>TTGTAACGAAGACGATAGAAAAACGTGTCCCTTGTTTCCAACTTTCTGGAACATTCATTTTATTCTTTTTTAATTGCAGATGATTTACG<br>CTTACGACACAAAAATATTGATTTTTTTATGAGTTACACAAAATCATTCATTTGAATAAAAACTATACTACTATAAGTTACGGATACTA<br>AAATATTGTTTTTTTAGCAAAACAAAAATATTGGTTAAAAGCGAAATCAGTAGTAAATTAGTTATATAATTATATATTGAAATATATGC<br>ATGAAAAATTTGAAGAAAAATGGAAAGAATCTCAAAAGATAAATGTAGAATTACTTGGAAATGACCGTACGCATTGATAACCCCAAT<br>AGTCTTTTTAACCTGTGTTGCCTACTGCGACCTACTCTATCATTTTGTTTTTTTTTTTGGTTTTTATAATCAACTTGTCTTCGTTATAAAC<br>AGAAGAAGATAAAAATTACTTATGTCATTATTATTAGTTTTGACTGTTGTTCATTACTAGTAGTAATTTAAACTTTAAACATATATAAA<br>AGCAAACGTGATGACCAAACTTTCAACATTATTAGTCACATAAGTACAATAATTATCTTTTTCTCGTTTTGTCAAAAAACTGATTTATA<br>AGGTTGGTAAAGACAAAACTAAAATATACATCATGATGTTTTTTTTCAGATGATTTAGGCAGGTACCCAAAATTATATCAACAAAACC<br>ACAACGGTGAATCATTACCTCGTTGAAGTTTTTTTTTGGTCGTTTTCTCATTTTCCATATGCATCAAATATGTGAGATAGTGTGCCAC<br>ACTATACACTATAGTCAAATATTGCGACGATCATAAGAAAATATATTTTACCTTCATGTTCACCACCCAGGCACTTAAGTAATGTATGT<br>TAAAGATGTGATTACTTTTAATGTTTTTTATTCATTGTTCAAACCTGAAAACTTTGAAAACAGAGCCGTCTACGTATGAAGAGAGCCGA<br>CTTAAGTAGTGTTTATTCACAGGGGTGAAAAAAAACAGAGCGTAACACTGTTTACAATAATAATCCATAGCCGACTCAACCAATTCCT<br>AAAAAAAAAATCTTCATAAAAAGACAAAATCAAAAGACAAGAGCGTGGCCTCTAATTAACCCAACACCCTCTGTCAAAAATAAAAAA<br>TAAAGCCTGAAACTTTCCTCTCCATCCCCATTTCTATCTGCGC

Name	Gene ID	Promoter sequence
CsDof19	Csa04g038300.1	GCTTTCTCACATGTTTCTTAAATCTAGAAACTTTCTTGGTGGGAATATATCCGAAAATAGCTATGAAGCTGGAGTTGCGTTAGCCGTTG<br>AAGCTCTAGGATTCTCTAATGATGAACAAAGTGGTGTATTAGTCAAGGAGTTTATAGAAAGAGCTACAAGAATTGGTCGAGCTCCGAT<br>TCTGAGGTCGGCTGAGTTAGCTAATGAGCTTGCTAGTGTCTTGCCAGCAAGACTCGAGATTCAATGGTACAAAGATCGGTGCGACGCA<br>TCAGAGGAGCAGCTTGGTTACTACGATTTCTTTAAACGTTATTCGTTGAAAAGAGACTTTAAGGTGAACATGAGTCGCAAAAGACTAG<br>CTATGTTTTGGGACAGAGTGCTTGAAATGGTGGAGACGAACGAGTTGCCTTTTGATTTTCATCTAGGGAAGAAGTGGATTTACGCATC<br>TCAATTTTATCAACTCTTAGCTGAGCCACTCGACATCGCGAATTTCTACAAAAACAGAGATGTAAGGAGCGGTGGACATTACTTGGAT<br>GAAGGGAATAGACCAAAAAGGTATGTGGAGATGGATAAGTGGTGGAAAAGAGGTGGAGAGCCAGAGAAGTGTGTGAGAAGCAGATA<br>CACTAGCACTACGCAAGATACTTGCTTTTGGGCTAAACTTGAGGAAGCAAAAGAGTGGTTGGATGAGGTGAGAAGAGAGAGTAGTGA<br>GGCTCAGAGGCAATCTTTGCTACGGGAAAAGATTGTTCTATTTGAGAGTTATGCCGACAAACTGAAGAGAAATAAGGAGGTTTCTGTG<br>GATGTTTTGGCGAAGAACTCGAGTTACACTGTGTGGGTGGAGAATCTAAGAGAGTTCAGGTCGAATATGGGTTATGGAAATGGAATT<br>GAGATGGTTGTTGATGAAGGTGACGCGATGGAGACTTAGTAGGACTAATAACGTATCTAATGTTTGATATGCTATAATAACAAACTTT<br>GTTAGTACTGTTGTTCTTCATTTCATGTAAGACTAATTTCTGATTTATGTTATATCATGTTTATCATTAATGTTCAACAAGTTTCTGCAA<br>GAGTTTCTATTAATGTAAAGTTATTACTCTAATAAAACCATGTTGTATAAGGTAAGCTAATTTTTATGATTTTTTTTTATAGATAGAAA<br>AACGTAAGACCTACATAGGATTCTTGGAGATTGCCATTGTTCACATGATCCAAGAAGCTCATTTCCCCATTCAGATTTTCCTTTCCAAT<br>CCCCCTTCTTTTGTGGGTAATTAATGAAATTAATACAAGTCAAAGTATACAAATAGAGACAGCCCTTGTTCTTCAAAACTTGTGCTTTC<br>TTGTTTCACATCCTCGTTTCTTAGTTTCGTTCTGTTCACAGATCGTTACGCATACCTTACATACCACACGACGATATATGATCTGTCTAC<br>TTACAAGTTCTGATCCTTTACGGCTTTGACTAAATCTTTTCATCGTGTGAATTTCAAATATGTTTCATATATAGTACTCGTTGTTATAAT<br>TAATTCCTACCATTATTGATATAAACTTTTACTTTATTAAAGTGATTTTACATTCTATTTAATAGACTTATATTATAGAAGTCGACAAAA<br>GAGAAGACATATATAGTAATCGTGCAGCCTCCATTAACTAAGCCGTACAAATAGCTCAATGTACTCCAAATAACCCATCTCTCCAAAG<br>AGGCCATAGGCTTCTTAGTTCGCTTCGAAACAATCTCCAAGACGATCTATTAATTAACCATTTTCTCATTCATATCTTATTTTTCCAAAA<br>CATATACTGGTATTTAAGTATATATCAAAGATTCAAAACCCTTAACCCTGTCTCCCCCAAGAAGCAACAGTGAGTGCACCCTTACGAG<br>GCATTTGCTCCCCCACACTTCCTCCACTCTTCAGCCTCCACGTGTTACACTCTCTCCACATGCACAAAGTCACACACACACATTCATGC<br>ATGCGTATATAACACATTGTCCTTGTGAGAGAGGGAGAGTGGAAGAGAGAGAT
CsDof20	Csa04g041620.1	GCTAGTTCATATTACTACTACATAATTTTAAATCATGTTCTTTCTTTGTGTTTCAACTTCATCCAAATTTCCAAGTAATTCAAATGTATT<br>GGAAGTTCTTCTTATCGTTTTGTCTCAGTTTGATCATTTTCTTGTAGTTGAAACGATTTTTTTTTTATAATGAAGATAACGATTACGATT<br>AGTGAGAGACAGAGAGAGATGTTTAATTTGCTGTTATATTAAAATATGTTTGTAAGTATCATTAGATAATCAATTTTAATAATATATAT<br>TTTTAAAGGTCGATTGTGAATTATGTAGTTAGCGATGTTTGCTGTTATGTGTCGACATAGTTTGGTCCATTAAAAAGGTCTGATCAAAT<br>TTTAACATTTGGACCACAAATCTTTCTTTTAGAAATAGCGCGCGGAGCGGACTGGGACACCTTCCTACAAACATGTCCGTCTTTACTAA<br>ATCAGTAAATCATACGTACACCTCACATCTGTAAGCATAAAATAAATCATCATATACATATAGACTGGCCGGTGATCATAATTCGAAA<br>TATATTAATTTCAATCATTCAATGTTATCGGTAAGTTATATTAGGGTCACATCAAGAATCACGAAAGAAAAGAAAGAAACAACCGTGT<br>AGAGTCATCATGATTTGATTTGTTGGCTTAATTATACATTCTAGTTTTTTTATTTAGCTTGTGTTGCAAATTATCCAAATCAGCACATTA<br>TAATTTCTTCTTTGATATCCAAGTTCTGAAAAAAAACGAACGATTATTGGATCACAAAATCTAATAGTATTATCATATGTTTGAAGGAA<br>ACCATATTAAAAGTGTCTACTTTTAATATTAATCATAAGAGTTCTTGGATCATTCTTAAGACTGACCGCTCGGAATCTATAAAATCTCG<br>CCATGCAAAGGTAATTTATTTTTATAAGACAAAAACAACAATAACGAAAATTTATAAGAAAGATAACTAAAATAATACTATGAAAAAC<br>GAAACTTGGGTCACACCCGAGGAAAACAAAGGTACACTCGATTGTGACAACTCCACCAAAGACCACCCCACTACCAATTCACCTTTAT<br>TTGTTTCTTTATAAATTTCCAAATCTTTTTGTTTTTTTTTTTATATATTAATCTAGCTAATTATTCGCCTCTCTCGTTGTTTTTTGACCTTT<br>TAATTAAAAAAATTGAAGGGAGTTGCCTAGGTTTCTCTCTCTGCATTTATTTATTTTTTTATTTTATTTTTTTCTCTCTCTGCATGGCCA<br>GTACTCTTGCTCTTCACATTTCTTTTTGGGCACCACTGTCTGTAAACGTATGCAAAGAAAGAAAAAAAAAAAAAGCTTACATGCTAAGA<br>AAACTATATTTCCTTGTCGTTTCTCTCATAAAAGAATTATATTTAAATTTATCGTTGCTAATGAAATACAATATATTTAGGAAGTGGAA<br>TCGGATCGGACAAGGGACTGAATTATCTACCAACTTAGATTTCAATCGTCTTTTTCTCATTGATTGACAAGTAACATACACAATACTAA<br>CACAGATATGATATCCTTATTAAAAAATGTATATATAATTACTTCATCAAAAATACACACATATGATATCATAGTTTCCTAATGTTGTA<br>GTGAAATTGATCAATTTCATACGGAAACAAATGTGCGAGATTAATATTACTATAACATATATGAAACAAACTCATACTTGATATAAAC<br>AGTGATACAACCAAGACACACACAATGACGTCGTATGACTTGCGAAAATAAGCATAATAGAAAAACTTCTTTAAATCGACACTTAATT<br>CCAAAAAGGTTAACAATAAATAAGAAGGTTTTTTGATGAAAACAAAAAGAAATAAAAGAGCCTAAGAGAATGATGAAAATTGAAAGA<br>GAAAAATAAACAAAAAAAAAAAGAGTAAAGAGAATTAAGAAACACAATAAATTAGACAAAGGAAACTTCATTTCTTCTTCTTTTTATC<br>TCATTCAGCTCCTCTCCTCTCTTCTCTCTCTAGATCAATTCTTTC
CsDof21	Csa04g051210.1	GGTCTTAATGATTACTCGTTGATCGGTTTCACCTGCTGTTGATTTTGAGAATTGTCATGCACTGTTGGCACAGTTGTGTTTGTTGTTGCT<br>GTTTGTGTATGTGTCCACATGTTAAATACACTGGATGCCTGTTTGTCAAGTTCAGTCAAAAAGGAGGAGATTGAAAGTGCAACATGGC<br>TCTGAGGATAACAAGTAGCTGTCAAGTATCTTAGAGGGCTGATATGCTGACATGACATGACGTACTGACGTGGAGAAAGATTGTGGA<br>CGTGCTGACAAGATGCATGGGGATAAAGGAAGAAGAGATCAAGATATACTCGAAGATATTGAGAGTATATTTGATAAGAAGAGATAA<br>GGGAAGATATTTTCTTAGGGATAAGGAATATTTCTTTTTAGTTAAGATACACAAAGATCTAGGTTAGAGATCGTTTGTGGGATTATAA<br>GTAGGCGAGGGCTAGCCGCAAAAAGAGCCAGCACTCGTGGGATAAAGTTGTGAGTTTTGTAAATTTTCAAGCAAAGAAAAAAAGGTTT<br>TGAAAGTGAGTATGTGGAGTATACTCTTTTAGATTTACACAAAGTATTATGTTAGATAGATAGGAGAGTGAGCTTTGTTTTTTAGATCT<br>TGTAAAAAACGAAAGTTTTTATAAAATTGATTAAAAACATTTGCTTAGCGCATTCCAAATGTGATCTTTGTATGTTTGTGTTCTCTATA<br>TTTTAACATAGTTAGCCGTTTTTAGTTTACTATGCGATCAATATATCAATCACACATTCACACTATAGATGACAGATCAAATCTATCGA<br>AATGTGTGGAGAATGTTTTCGTTGTAAGTAAAACTTACAAAAGAAACCTCGGAAAATGGTCCCAAGGCTCAGTGTTTTAGAGTCACGC<br>GGTTCGAGGGAGCGTGACGATCAATAACATCTACTAGAAGATATTGCCATGCAGAGGAAAAAAGCAGAGACAGGGAGAGAGAGAGG<br>GGGAACATGATGGACCGGTCACGATGCTTCGGCTGTCGTCCAACGACCAAAGCACCCATTTTAACCCACTTTTCCTATTTTCAATCTCT<br>TTTTCATTTCTTTTGTTTTCAAATTATTAGAATAAATCATTATATGATCAACGATCCTATTATAATCATTATGTTAACACCTCGAAATTC<br>GGTCTACATAACACCTTTGTCTCCTAAATCCGCTCCATATATTCTACTCGGATCAATCTACACAGAAATCAACTATATATAATCACTAT<br>CAACCAGATAAATCGATTATTTTAGTTGAATCTAAAAATATAAAATAACACATTGTGGAAAATTCTAAGGGACAAGAGTTAACTTTTA<br>CATAATTAAGGATTTCATCCAGTTCGATTAGGTCGTTATGTTTTACAGCAAAAACATTTCTTTGTGCGACAAAAATAAAGAAGTACAA<br>GTTACAACAACATTTATTAATAGGGATGAAACGGGTTTATTTATTAAGTTATAGGCGTTGTTGATTTGATCTACAAATCTCGGTTTACA<br>TGTGTGAATCTATGAAAGCGAGGGCCATCACAAGGCACAAAATAAAAGCTTATGTATTAAGAAAATATACTTATATATATGAATGTAA<br>CTAGTGATAGGAAATTTAAAACCCTAACTAAAGCAAGAAAACAAAAAGAATCATGTCAGTCCCACCCCATATGGAACACAAACCCTAA<br>TAATTACATATATGACAATTATCTTCTTACATCTCCATACAAAATTATTGTATACACACATACATATATATTGACATAGATATATATAA<br>AGGTTCATCCACTTTAAATTTTAGCCATCTTCATTCTCACACTCAACCTTCCTTTCTCTCTCTTTCTAGCTCCCTTCATTCTCATTCTCTC<br>TCGGCTTTGTTTTCTCTTTGATCTGATTCCTCTGTTCTATTTCTAATCCATCACCCACCACGGAAAAGAGAAGGGAAAAAAAAAAAGAAC<br>AAAAAAAAACTTTACTTTAAGAATTTTGAAGAATAAAAACAAAAGAGAATAACA

Name	Gene ID	Promoter sequence
CsDof22	Csa04g065700.1	TTTATCGGGCCACAATTGTTATCGGGCCATAACTGTTATCGGGCCTATCACACTATTTTTTAAAACTCAACATAACTAGAAGAGAAAG<br>AAAACAAAATACACTGCTAACCGACTTAGGAAGGGACAAGTGGCAATTGGCAAGTCGTTAAAGCAAAACGGCAGGCAAGGGAAAGCT<br>GAAACAGTGAAACGACACGAATAGATAGAGATAGAGATATTTTCATTGGGTGGGGGAAGGTGTTGTTGGGTAAGAGAAGACAGATGT<br>CAACATCAACAAAGAAAATTAAAAAAAAAAAAAAAAAAAAAAAAAAAAAAAAAAAACTAGAACAGTCTCTAAGATTTATTATTATTAGTTGT<br>AAATATTTGTAAGAAAAAGGAAAAATCTTAATCACGCCACGAAGTGTGGGACCCAGATCCACCTCCTTTTTGTCGCTTCCTGTCGGTC<br>TTCTCTTCTTCATTCATCTCTTCTTCTCTCCCTCGTCCTCTTTTTCAAAATTCTCCTCCTCGAAAATTCCAAATAGATGCAATCTTGTTTA<br>GTATTTTATTTGTTAACTTAGATTTCTTTTTCGTTTATTATATTATTATTCGTTTGTATTATTGTAGCGATTGCCAAAATGAATCTATGA<br>ATTGATTATAAAGGGGTCAAATTCATTCATTACGATAAAGCAAGCATACAGTATACTATTAGGCATTAGGTTGTTTTTTGCTTTATATA<br>TAACTCATGTAGAATCATCTACTCACCTAGACGGTTCACACGGTTTTATTATGATACCTCTCATGAATTTTTCCGTGTACACATACTTTA<br>TATTTCACAGTATATACTTTTCATTATAAGATCATGTTATAATCTTTATGATCACTTTTAATTAAAATCATATTTAACTTTAGATATGTA<br>TGATGTAATGACGCCTGAAAAAAGCTAAGATTGTTTGACAGAATAGACATACATGTAACTAGATATTGGTGTTGTTTGGTAACATTAT<br>GTGTTATGTCGTAATCATTAAAAACAAATGAACTATAGAATGACAAAGTTGTATTTCAACCATTGACGAATCTCATCGGTTACTTACAC<br>AAAATGGAGATCGTACTGTATATTGATATAGTAAAGTTTTAAAGTCGAGCTAAATTTAGGTGTCAAAGCCAGTAATTCACATGTACGC<br>AAGACATGTGGTGTATATACATATAGGTATGGTATTGGGATTTTAAAAATGCAGCTAGGCGATTTGATGGACCATATGGCCAACAAAT<br>AGTATCCAATCACAAAATGGGAGGCCGAGAAATAGCTTACAACTCATAGTTCCACTCACACTATGCATTATGATACACACACAAAGAG<br>TTTTATATCCCTTCACAAATATATTATTTTAGCAAGAGGACATAATTCCTTACACACGATTTTTTTCTCTTAGAAAAGTATATTTTTTTT<br>TAAGAGGGACCAGAAAGAGCTAGCAAGAGAGAGCGGTCACGAGACATCATCACTAGTCAAAATCAACGAACCCAACTTTTTCTTTTTT<br>TCCTACTTTTAATCTTTTTTATAATCGAAAATTTTAAAAAAGTTTAATCCATAGTTTTCTCCCGAATTAGACGGACGAAATTATGAAATT<br>ACAAATCATAATGTAATCTCTAAATCAAACATTTTTTCATCATGATAATCGTAATGGCATAAACAAAAAAAAATTCTTTCTATTCAATGA<br>ATCATATTTAAACTATATATAGTTTTAATAGAACTGAATTATTCTACTCAAAACACATCTTAGTTATATATGTATAGCTATATTCTCTTA<br>GATAATAAGTTAAAATTTGGGTTCTTCTCTTCTCCGTCTTCTTCGCTGGCTCTTTTTTCATCATCCATAAAGTGAAAGGCAACGCATAG<br>AAGAGCCATCACTCACAAACAAAGTAAAGGGAAAAAAAAAAAAAAAAGTTCTCAAAAAACATTCTTCTCTTTGGCCTTTACTCAAAACCC<br>ACACACACATATCTCTCTCTCTCTCTGGCTTTCTCTTTGTCGGAAGTC
CsDof23	Csa05g002560.1	TGGGGGAAGGTGTTGGGTAAGAGAAGACAGATGTCAACATCAACAAAGAACTACAACAGTCTCTAAGATTATTATTACTTGTAAAAA<br>TATTTGTAAGAAAATAGAAAAATCTCAATCACGCCACGAAGTATGGGACCCACATCCACCTCCTTTTTGTCGCTTCCTGTCGGTCTTCT<br>CTTTACTCCCTCGTCCTCTTTTTCAAAATTCTCCTCCTCGAAAATTTTAAATAGTTTGTTTTAGTATTTTATTTGTTAACTTAGATTTCTT<br>TTTAGTTTATTTTATTATTATTCGTTTGTATAATTGTAGCGATTGCCAAAATGAATCTATGAATTGATATAAAGGAGTCAAATTCATTC<br>ATGACGATAAAGCATACAGTATACTATTAGGTTGTTTTTTTGCTTTATATAACTCATGTAGAATCATTTACACACCTAGACGGTTCACA<br>CGGTTTTATTGTTAGGGCATCTCCAATGGTACTCTATTTTTGCCTCTATAATAGAGGAGTTCTATTTTAGAGGTGAGAATAGGGGAGTT<br>CTACTCCAATGGTCCTCTATTCTCACCTCTAAAATAGAGATTGCTATTTTTTCCTCTATTTATAGGGGAACCCTATTTTTTTTTTACAAA<br>GGTTCCTCTATAAATAGAGAAAAAAATAGCAATCTCTATTTTAGAGGTGAGAATAGAGAACCATTGGAGTAAAACTCACCTCTATTAT<br>AGAGTTCCTCTATTATAGAGGAAGAAATAGGGAAAACCATTGGAGATGGTCTTATACCTCTCATGAATTTTTCCGTGTACACATACTTT<br>ATATTTCATAGTATATACTTTTTCATTATAAGATGTTATAATCTTTTATGATCACTTTTAATTAAAATCATATTTAACTTTAGATATGTA<br>TGATGTAATGACGTCTGAGAAAAGCTAAGATTGTTTGACAGAATAGACATACATGTAGCTTGATATTGGTGTTGTTTGGTAACATTAT<br>GTGTTATGTCGTAATCATTAAAAACAAATGAATTTGTATTTCAACCATTGACGAATCTCATCGGTTACTTACACAAAATGGAGATCGTA<br>CTGTATATTGATATAGTAAAGTTTTAAAGGTCGAGCTAAATTTAGGTGTCAAAGCCACTAATTCACATGTACGCAAGACATGTGGTGT<br>ATATACATATAGGTATGGTATTGGGATTTTAAAAATGTAGCTAGGCGATTTGATGGACCATATGGCCAACAAATAGTATCCAATCACA<br>AAATGGGAGGCCGAGAAATAGCTTACAACTCATAGTTCCACTCACACTATGCATTATGATACACACAACAAAGAGTTTTATATCCATT<br>CACAAATATATTATTTTAGCAAGAGGACATAATTCCTTACACACGATTTTTTCTCTTAGAAAAGTATATATAATTTTTTTAAGAGGGAC<br>CAGAAAGAGCTAGCAAGAGAGAGCGGTCACGAGACATCATCACTAGTCAAAATCAACAAACCCAACTTTTTCTTTTTTTCCTTCTTTTA<br>ATCTTTTTTATAACCGATTTTTTTTAAAAAAAGTTTAATCCATAGTTTTCTCCCGAATTAGACGGACGAAATTATGAAATTACAAATCA<br>TAATGTAATCTCTATATCAAACATTTTTTCATCATGATAATCGTAATGGCATAAACAAAATAATTTTCTTTTCTATTCAATGAATCTTAT<br>TTAAACTATAGTTTTAATTAATAGAACTGAATTATTCTACTCAAAACACATCTTAGTTAATATATGTATAGCTATATTCTCTTAGATAAT<br>AAGTTAAAATTTGGGTTCTTCTCTTCTCCGTCTTCTTCGCTGGCTCTTTTTTCATCATCCATAAAGTGAAAGGCAACGCATAGAAGAGC<br>CATCACTCACAAACAAAGAAAAGGCAAAAAAAAAAAAAAAAAGTTCTCAAAAAACATTCTTCTCTTTGGCCTTTACTCAAAACCCACAC<br>ACACATATCTCTCTCTCTCTCTGGCTTTCTCTTTGTCGGAAGTC
CsDof24	Csa05g016230.1	CTCTTTTTCATTTCTTTTGTTTTTCAAATTATTAGTTATAAATCATTATATGATCAACGATCCTATTGAATAATCATTATGCTAACACCT<br>CGGAATTCGGTCTACATAACACCTTTGTCTCCTAAATCCGCTCCATATATTCTACTCGGATCAATCTACACAGAAATCAACTATATATA<br>ATCACTATCAACCAGATAAATCGATTATTTTAGCTGAATCTAAAAATATAAAATAACACATTGTGGAAATTATTCTAAGGGACAAGAG<br>TTAACTTTTACATAAGGATTTCATCCAATTCGAATTGGTCGTTATGTTTTACAACAAAAACATTTCTTTGTGAAACAATAATAAAGTTT<br>TACAACAACATTTATTAATAGGGATGAAACGGATTTATATATTAAGTTATAGGTGTTATTGTTGATTTGATCTATAAATCTCGGTTTAC<br>ATGTGTGAATATATGAAAGCGAGGACCATCACAAGGCACAAAATTAAAGCTTATGTATTAAGAAAATATACTTATATATATATNNNNN<br>NNNNNNNNNNNNNNNNNNNNNNNNNNNNNNNNNNNNNNNNNNNNNNNNNNNNNNNNNNNNNNNNNNNNNNNNNNNNNNNNNNNNNNNN<br>NNNNNNNNNNNNNNNNNNNNNNNNNNNNNNNNNNNNNNNNNNNNNNNNNNNNNNNNNNNNNNNNNNNNNNNNNNNNNNNNNNNNNNNN<br>NNNNNNNNNNNNNNNNNNNNNNNNNNNNNNNNNNNNNNNNNNNNNNNNNNNNNNNNNNNNNNNNNNNNNNNNNNNNNNNNNNNNNNNN<br>NNNNNNNNNNNNNNNNNNNNNNNNNNNNNNNNNNNNNNNNNNNNNNNNNNNNNNNNNNNNNNNNNNNNNNNNNNNNNNNNNNNNNNNN<br>NNNNNNNNNNNNNNNNNNNNNNNNNNNNNNNNNNNNNNNNNNNNNNNNNNNNNNNNNNNNNNNNNNNNNNNNNNNNNNNNNNNNNNNN<br>NNNNNNNNNNNNNNGAGAGAGAGGGGGAACATGATGGACCGGTCGCGATGCTTCGGCTGTCGTCCAACGACCAAAGCACCCATTTT<br>AACCCACTTTTCCTATTTTCAATCTCTTTTTCATTTCTTTTGTTTTTCAAATTATTAGTTATAAATCATTATATGATCAACGATCCTATTG<br>AATAATCATTATGCTAACACCTCGGAATTCGGTCTACATAACACCTTTGTCTCCTAAATCCGCTCCATATATTCTACTCGGATCAATCT<br>ACACAGAAATCAACTATATATAATCACTATCAACCAGATAAATCGATTATTTTAGCTGAATCTAAAAATATAAAATAACACATTGTGG<br>AAATTATTCTAAGGGACAAGAGTTAACTTTTACATAAGGATTTCATCCAATTCGAATTGGTCGTTATGTTTTACAACAAAAACATTTCT<br>TTGTGAAACAATAATAAAGTTTTACAACAACATTTATTAATAGGGATGAAACGGATTTATATATTAAGTTATAGGTGTTATTGTTGATT<br>TGATCTATAAATCTCGGTTTACATGTGTGAATATATGAAAGCGAGGACCATCACAAGGCACAAAATTAAAGCTTATGTATTAAGAAAA<br>TATACTTATATATATATGAATGAAACTAGTGATAGGAAATTTTAAACCCTAACTAAAGCAAGAAAACAAAAAGAATCATGTCAGTCCC<br>ACCCCATATGGAACACAAACCCTAATAATTACATATATGACAATTATCTTCTTACATCTCCATACAAAATTATTGTATACACACATACT<br>TATATATTGACATAGATATATATATATAAAGGTTCATCCACTTTAAATTTTAGCCATCTTCATTCTCACACTCAACATTCCTTTCTCTCTCT<br>TTCTCCCTTCATTCTCATTCTCTCTCGGCTTTGTTTTCTCTTTGATCTGATTCCTCTATTCTATTTCTAATTCATCACCCACCACGGAAAA<br>GAGAAGAGAAACCAAAAAAGAAAAAAAAACTTTACTTTAAGAATTTTGAAGAATAAAATCAAAAGAGAATAACA

Name	Gene ID	Promoter sequence
CsDof25	Csa05g035600.1	CATAAGGAACAAGATTACTATCGTAGATTTTGAATGGTGACGTAGAACAGTTTCTATTTTACTTCCGTTTAGCAGTTGCATACACCTTT<br>TTATCAAAATAATAACAATAATAATAAATAAATAAAATTTATGGGTATTTGTAAATTTGAATTTTCATAATTATTTTGCCAATGAACTT<br>TCTCTTCATACATTTTTCTTTTATCGATATATATATATATATATTTGTAAAGGTTGAACAAATAGAGATTTTGTCGATTAATTAAGTTGA<br>TCAACCAAAGTAATCACATTCGAGTCTAATTTTTTTGTTCAAATGATTAAAAAAAAAAGATTAAAAGGATAGATTTGATGCTAACCATT<br>CAATGGTGGTTTTACTGTGGCTTGTCAACTTAATTCATTTGTTATATTGCAAGATTACAAGCCTGAATACTTTCGAATGACATAAATAA<br>TTAATATATTAAAAAAATTGATTCAATATTACACCATGGCCATGGAAAATACAAGCATGTAATAGATTGATATCTAATTAGTGAAGGA<br>AGACGAAAACGACAGATCAGGGACAAGGCTAGTCTTGAGTCTTGACCCACAAAGATACCCTAATTAAGCTTTCATGATTACGAATCTA<br>TATGATAATGAACCCTATTAATTAGAAAAGTTAATATATACTTACCATCACCGTACACTTATATTTACACACACATTTAATTATATGCA<br>TAGACAGCATAGTTGATTAAGTGCACTATTTGCGATAGCAAAACAACTTAAAATGATAATTATGAAACATTAACCAAACGTTAATAGT<br>TTAAGTTAACAATGGTCTATCCCTGCCGTGGGTTGGTCTAGAAAAGAGGTATTTATTGGGGGCACGAATATGTAATTTGCTACAACAT<br>CAAACTAAAGTATACAGTCATAGTAAATAATAACCTGAAAATCTAGTAACAATAATAAGACTTTTCTTTAATTTTATTTTGTTATAAAG<br>GCTTATAAAATTGTTTTTTTTCTGCTAACTGAATAATTATTAGTTTTGTATAATTTTGTAAATTTAAATTCTGACTGATACAAATATATA<br>TATTAAGTGCAACCAAAAAGATAAGTACGAATAATATTTTAAGTACGAATAATATTGATATAAAAGCAATACTAGTTATGCAGCATTG<br>ACAAATTCAGAATAACCAGACTCCCAACATTTTAAAATTCTGAAATACATAAAGGACTAGACTATAGATCTAATGACTGGATAGACCA<br>AAAAGATGATTTGCATATTGTGAAAAAAATGTAATTAAAAATTGTTTGATTGTACCACTTTTCGCAAGTCCCTCATATCACTAAACCAG<br>TTTATCTGAACATTTCTCTAACTTATTTTACTTCGTGGTAAAGATAAAACTGATATGATTGTAGTCTTTTATTATAAATAAATCGCTTGA<br>AGCAAATCAAGGTCTTTTGATTGTCTCTACTCTCTAGAACGTCTTTAATTATTTATATTAGACCTTGTAAAATCATATGGAAAATATAC<br>GTAAAATATATATATACACAAATATATAATATTTATAGTATAGATATAATTCGACAGAGAGAACCTTATAGGCGAAGAAATGTCACTC<br>CACTCAAACCCTAAAAAGAGAATCATAACAAAATCAATAATATATATACATATATACACACACATATATATATATATAGACGCATATA<br>CAAATAAACAATGCATGAGAGTGGGAAATATATATAAAAAGAAATAAAGCATGAGAACATGACCCATGTTTAGAAAAGCATTATGTT<br>CTTACTTTATCTTTGTGCTCATTCTCTATCATCATCTTCTCTCTAAACCTCTACTCTTCTCTCTTTCCCTTTTGGTTCTGTGCTCTCCCTCA<br>CATCTTCTTTAGCCTTTGGTTCCCTTCTTTCGTTTTTATTTTGATTCTCCCACCATTTTTGTTCTTTTGAGTTTTAAAGATCCATCAAACT<br>TTATTAAAGAAAGAAGAACAAAGAGATAAAAGGGAAGAAGAACA
CsDof26	Csa05g038090.1	ACACCCCCATAGTTATGCCTATAAAGATGCCTTGTAATGCACTAAACTATAACAGCATAAATAAGCCATATAATAAGATGAAAAATAG<br>GAATGAATCAATCAACATCCTCAATTCTGTGTGTTCTCTAACTAATCGGCCCCACGAGGACCGTATAATTGTACAGCCACGCCGCATCC<br>GATTCCTCATTTTTTATTCAATATATTATTTTAGTATAAAAACATTGCAATCATATATACAAATATAAACTCCTACCAAAAAAAAAATA<br>CATATATAAATTAAACTGTCTAAATTCCGCATCTTCCGTCTATGATCTTCTTACTCATCCTATTACTAAAATAAAATAAAAACCACTCC<br>ATACAAAAAAAGGTTTGAATCGAGAACCAAAAAGAACCAATCTCTTTATATCACAACTAGGTTACCACTTCTTTTTCTTAGTAGCACAA<br>TTTGTATGTTAAGCGTCTTACTAGCAATGGTTGGAGTAGTTTTCTACTTTTCTTGGTTGTACACATATGTAGACGAGAAAATGTATTTA<br>TATAGTATGTATACTTTCATACGCGTATAGAGAATAAATGTATATTCTTACACATACACGGACGCGTAATGGTGTGGAATTTGAAACG<br>AGAATGGGGTTGGGGTTGGTTGATGGTGATGGTGGCTATTGGTGTGGCAGGGTCCACATGTATATAGTTGAACCCACGCCAGATTCG<br>GTCGTCTTGACTTGTGTGTACATAATATACGTATATGTGTACGACCAAAATAAAGCCAACTTATGTTATTAATCAACACCATTCATTTC<br>TAATTGTGTTATATTAACTTTTCGTGAGCAGATATAGACTTTTTGAAGTGTATAAAAATTATTAAGACAATCTCCAATGTATATCTCTA<br>TTTTTTCTTTTAGAGTAACTCAAAAATAGAGCATTGTTTTACTCAAATGTATTACTCTATTTTTAACTCTAAAATAGAGTTAGTGTAAAA<br>AAATAGAGGTGAGAATAAAGTTGCTCTATTTATAGAGCAATCCTATTATTTTCTCTATTTTAGAGTAAAATATAGAATAAGGTTGGAG<br>CAAATGTTGTTCTATAATAGAGATTTGACTCTAAAATAGAGTGGGGTTGGAGATGCCCTAAATATGGATTATAGAATTTTTTTGGTACT<br>TCTAACTTTGTTTTTACATATTCAATAACACACGCACTTTTTTTCTATCACAAAATTAAGAATGATCAATTAGCTATCTTGTCAATTGAA<br>ATTTTTCCTAAGTAGGATAGCGTATTAGTTTTGATGTATGTTTCTTCGGTTTAAACTGATATTCTTCAATGTCTCTTTTGGTTCTCATTG<br>ATCCATAAGATATTTTCTCTTGTCATTTAGTCTTGGGGATTCAATTCATTTCGATTAATATTCTTTTTTAGATATTTGTAGCATCACTAT<br>TGTCTACTTCATTTCTTTTTATTTTTACGTATTCTAGATCTTTTTTGGTTAAACAGATAATTTATCGAAATAAATGTCTTAGAAAAAAAG<br>GAAAATAAAATAAGAAGAAGACTCCTTACGGTTCTAGTTTAAATATATGTAACAAATAAAGTTAAAATTCTAAGTTTGCACTTTGCAC<br>AACAAAAAGAAAGGTTTATCGAGCAAAAGTTTAAGTTAATTAACATATAAGTTGAAGACTTTTGTAAAACATTTCCCGACTGACATGT<br>GAATATCAAAATAATTATTAAAAAATACAACAACAAGTAGCTACAATTAAGAAATAATTTGAATTATGAAAAAAACCCATATATTTTA<br>AAAGGACAGACTTTATCTATTTTGCTTTATATATTAAGAACCGTGCTCTTCTTCTCTATTCATCTCTTGCTCACCACTTTTCTCCAAACT<br>CTCTTTCTTTCTCTCCCTCTCTCTAGTTTCCTTGTTCTTTTGGAGTTCGAGCAAAAATATCAACCATAATTGCTTAGTATTACTATATAA<br>CTACGTAATCGTTGCTGACTTGCTTCTATCCTTTGTTGCATGG
CsDof27	Csa05g086300.1	TTGATCTCCGTGGTGTGATCGTAGTAGGATTACTTGAGGCAGTCAAATAATTAAAAAGAGGATTCCTTTGTAGACGCTGGCGTTTAGC<br>TGTACACCCTTTAATGGGCCTATATGATCAATAACAAAAATACTATCGAATATCAAGTGTTACGGGCCTATGAACTAGATCGTTAATG<br>CCCAAATTTTATCTCATTAGTTTAAAAAGGTTTTAGAGTCCACAATTCCTCGTTTGTAATTGTCAGAACACGGTTTTAGCAAACAATTA<br>ATAGAAATTTAAAGATTATACTCATTTACTATATGCAATCGATTACACTTCCAAAATATTTACTACATGCACTTCCTGACTTCCAAAAT<br>ATAATTATGATAGTTATATGATAATGAAGTATGTAGTATATGATAATTTCCAAAATATTTTCGTCACTCAGACGTCATAGGTTTTATAG<br>TTTCTAAAATATCATTTAATGTTTAAAAATAAAGTGGTATAGTACATAGTACTACATACTCTTTTTTCACAATAGGAACTTTGAACCTT<br>GGCATTCATAAAATGATTATAGGCTTTCATTATGTCAACCTTAGTCAGAGCAACTATGCGAATGTAGTGGACAGTAAGTAGTAAGTAG<br>GATTATAATTGCAAAATACGAAAACAAATGTTATAATAAGAAAAAGAGAAAAGAAAAAAAAAAAACACGATCGGAAAAGACTTAATGG<br>GATAAGAGAAAAAGATATGGTCCAGCCTTATCTCATACCTGGAAATGCCACGTGTCAGACACTACCAGAACATAGCCTCCTCTTACAA<br>CCACAAACTTTTTTTTTCTAATTCTTTTTGATTACTTTCTTTCCCATTAACGTAAAGAACACAACGAACGATGCTAGCTAAGCTATGTTT<br>GGATTTGGATACGCTCACGCTACTATATACTATGAATAACCCCTACAAGAGAATATAAATATGTTCCTTTAATCCACGTGTCGGATACG<br>CATTGGTCACATGCTTGACGAACAGCTAGATTCTGGCAGATGAACTTTTTAAAGAGTCCTGATGGACCAATCCACACGTGCTTTTATTA<br>CAGCTGACGTGGGCTTTTTATGTAGGATGTTTCGGAGACGAAGCGGGTCATGTGCCACGTGGTTACACAACCTGGCATTGACAGTTTA<br>TGCCTTGTCGGATTACTTGTTGTAACTTGTAAGATATGTATTTTGTTACCTATTTCTGTCGAGCGAGAGAGAGAGAGAGAGAGATACC<br>TAATTCTGTATTAACACCCGATTATGCTAACTTTTACCATGATTAAAAAATTGCAACCAGAGAAATTTGAACACTTGTTAAGACATGTT<br>AAATCTTTCAACCGCTGAAAGATTTTATGTTAAGACATAAAATACATACGTCAACGAAAGTAATGTAATAAGAAGTTTCGATTGGTAA<br>TATGAACAGAACTTGAAAACCCTTCTTTTCCTCATAATGTTTTTGTTTATTAGTATGACTTTCATGTTACGTACATCTGACTTTTTTTGT<br>ATCATCCGTTTTGGGTACGTTACGTTAACACAATTTTGAACAAGCAGTAAGATAATACTAATATTTTTATTCTTATAAAAATGCTTTTT<br>GTAATAAACCAGAGGACACTACGAACCAAGTGTTTTTCTTTTCTTCTCTAAATATTCCTTTTCCCTAATAATATATCCGATGTGGCAAC<br>AAAGAGAAAGGTGAGAAAATCTTTTAGAACGCCACTAGAAGAGGGAATCAAAAAGAGCGTGAAAAAGATCTCAAAATGCCCACTTCC<br>TTAATAAAACATGTGGCTCTCACATTTTCACAAGTCTCTCTCTCTCTTTCTCTCTACGCTGACTTGTAATTTTCTCTTTCTCTCTATCTAT<br>CTTCTTCTTCTTTATCGTCATTAATACCCAACCCCAAGCCCCTTGTTTTCTCTCCATGCTCCGATCTTTAAAGAAAGAAAGAAAAAACG<br>ATTTAAACCTTCATATACTTACCTAGAAGTTTCATCATAATTTATAGTT

Name	Gene ID	Promoter sequence
CsDof28	Csa05g094020.1	TTGGAGGTTTAACCGGTTCAAATTCGTTGATTCCTTTATACCAAATCTAATTTAGTTGGTGGAATTCTTGACCAAAAATTATGTTGTTA<br>AGCCCAATTGAATTAATGGGCCATATATTAGAAAAGGCCGGATAATTGATAAACGGAAGCTTCTTCTAGGTGAACGATTTGGTAGAAC<br>ACGTGGACCGTGGTAGGTCGTTCAAGACTGAATAAAACGGCAGACAAGGTAAAGCTAGAATAACAAAACGACACGAGAAGTGGAGA<br>TGATAGATTGGGGTAGGAAAAGACAGTTGTCAACATCTTAAAAAAAAACAACAGTCTCTTAGATTTCAAAAAAGCTAATCACGCCCCA<br>AAACTGGGTTCCACCATCATCCTTTTTGTCGCTTCCTGTCGGTCCTCATCCTAATTCTTTTCCAACCCCTCATCTTTTGAATATATACAT<br>AAATAACCGTTTTACTTTCTTTTGTTAAGTGTTAGACCCAAAAATTCTAGAATTGTATCGATGTGAATGTAAGTACCGGCTAATATTTG<br>TATGCTTAAGTAACTGGTACTGCTTAATTAAATTCCAAAATGGTCATCTGATTACATGAATGTGATTTTAATCATTCTTAGTACGTAAA<br>GGAAGTTGCACGTTGGGTAATTAACGTTTTAATAACACGTGATTTTAATATCGTAAGGATGATTACCATTTATAAACGAAAAGCTATA<br>TATGTAAGATAAAATAAGTCGTGCAGTATTGTTTGTTTGAAAAGTTTCTTTGGTTTCTTATAATTGGCCTCGATTTTCAGTTTATGTGCT<br>TTTACAAAGTTTTTATTTTAGTTATCTGATTAACGCGACATTTGTTGTGTAGCTTTGCCGATTTAAGAATAAATTCATATTAACTTTATG<br>AAGCCATGTGTGGTGTAATGTATAGTGGTCCTTAAACGAAAAAAAAAAAAAAAGCAGATCACAATTCAAATAAAGGGTGAAATTTTGG<br>TGTACCTTTTTCTTCGTCGAAACAACATAATTAAAATAAGATAAAGCATTATTTGCGTCGGAAGAATACTCTATATTCCTGTAAATACA<br>GTCAACATATATATTGTACTTTATATAAAGTTAGAAATTTTAAAAAATCATATATTTTATCGACCATATAATTAAGTCACAACTTTATC<br>ATAAATTAATCAGTAATTAGAATTTCAAGTTCGCCACCGAATTTCGTAACCTAATATACGGAATTATGAGATCTATATAAGGATCTTGA<br>AATTGCCAAGGTAATAATATATATTGACGGATCGTAACCCAACATATAATATTAATTACAAAATGGGTGGGCGGGAATAGTATACAAC<br>TCATAATTCCACTCACATTTTGTATTATTATGATATGAAAATAAATCAACATGCATAAAATAATATAGTCATCTGTATATTACCGTATC<br>ATCTTTGAAAACATAATTTACACATTGGTTTAGTGACAGAATTCAGTCGCAAATCATGAAATGATGACAAGTGATTCAAACAAATTTT<br>AAACACCACGATCACGATGCGTATAACACTGTAATAATTACTCTATACTGAAGAAGATAAAATCACTAGTCTATATATTGAGACAACC<br>AATTCGTTAAATAGAGCAATCAATATTTTAGCCAGATATTATTTTTCCTTTAAAAATATATAAAAGAGGGACCAGAAAAGAACCAGAG<br>AGGGAGGCCATGAGACATTATTATCACTAGTCAAAAACAACAAACCCTCCTTTTGCTTTTTTCATGTAAATTATTATTATATTTCAATT<br>TTGCAGGTTTCTTCTCTTCTTCTTCTTCTTCTTCTTCTTCTTCATCTTGGCTGCTTTCTTTCATCATCCATAAAGTGAAAGCTAACGC<br>ATAGAGAGTCATATCGTCCCAAAAAAAGCAAAAGTCCAAAAGAAAAACTATCTCCAAAAACATTCTCTCTTTGTTCTTTACTAGCTCTT<br>TAGTTTCTCTTTTCTCTCTCTCTCTCTGCCTTTCTCTTTGTCGAAGCTC
CsDof29	Csa06g019350.1	TGATGATTTAAGTATAGTGAATGACTTAGCTAGAGAAGCTTGAGTCTGCTCAAAGCAAGTACAATGCTATGTCCCTAAATGCATGTAG<br>CTTGTATCACTCTTGGTAAAAAGTTCGACTCACCACTAAATCGCTAACGCAAGTTTAAGTTACACCTTTAATAAACGATTGGATCATCA<br>TGATTGGTGATTTTGATGTCGTTCAATCGATGTTAAATTGTGACATATATACAATAAAGAAAATTACTAGATAGAATATAATAACTTGA<br>TTTTACAATTTCTGATGATACAAATTTATTCGATTAGGAAATGCATAAAACACAGTCCTAACTATACAATATACACAAATTAAAGAATC<br>ATTATAAAACCATAATATACAATATTAATGCTCTCTTTTAATTACACTTGTCATCATGTACAACTCAACTGTTTGAACCGTGATCGTGG<br>TTTGCTAAATATTACATCCAACAAATGAATCCAACAATCATATTTTCAAAATTACAAAACGTAAACTAGTACATACAAGAATTTTAAAA<br>TAATGGACGCAAAAGTCATGAACCACATGTGCTACAGACACATAGATATTTGTGGACCTCGGCTCCTGATCAGTCCTCACAGAGAAAA<br>TGTGGTCCAGGAAATGATTAATCATGCACCAACTATAGTTATGTAGTGTTATTCTACCCTTTCAAGTTTCACCCAACATTTTGGAGGGT<br>AAAATATTAAACTTAACTGTGACTTAACTAAAAGAGAAAAATAAAAGATTAAAAAAGGAGAAGAAAATTGAGAAATATACTACTTTG<br>TCACACTTAGGACCAGCTTGAGGTAGTGGTGGGATTCACGCGAGAGTATAATTTTTTGACTAACTTAGAAGATGAAGGTGGGTTCCGC<br>GTGGTGATGACAACTCAAGAAGAATTGTGACACAAAAGAAGAGGTCAAGGACACATGAAGATGGGTGCCTAAGAAATGGACGTCTTA<br>CAAACAACCAATTCTGATACATCATAGTACGGTCCTCGATCACACTTCTCATCATTTATACAGAAATATATATATATATATATGTATATAA<br>ACATATATAATATTTAGTAATTGCATATTGTTATTGACTCATTTTGTTAAAAATCCCACAAATTTATTTTTTCTTAGAAACCCTATATAA<br>GGATCACAATAGTTTAGTTTTGTATTTAGAAAGTTTCGAACAAAATCCAAAATAGATGCAAACACATATCTTATGACTATTTTTCATGT<br>ACATCATCTAAAACTATTTTTCGATCCGAGAGTACTAACTGTGTTTGGTAGTTTCAAACAATATTGCCCACATCATCACATGTAATGTA<br>CTCAACATATAAAATTTGTACATGATAGAACAATCTATATCAACAAGTTTTGTAAATTTACGGAACACATAGTTATATTGTAAAGTGAA<br>GGATGTGGCTCCATCACAATTATAAAAAAAATCAACGTGTGATGATAATGAAAAATCTTCGCTCACAAGATCTCGATATCGCAATATA<br>CATATATATATATATATAGAGAGAGAGAGAGATATTTTATTATTAATATGATACCATTGAAGAGCGGATGAACAAGCATAATTAAATT<br>AGAGACAACTAGTCTTAATCTAAATATTGAAATAATATATCCAATCTAAATGAAGAATTAAAAAAAGGTCTAGTTGTATATCAGAAGT<br>AGGCATAATAAATCTTTTTTTTTTAAAACCGGCTTCATGGGTTCACATCTCATGCTAAGAGTTGGATCACGTTATTCCCTCATACGGTA<br>ATAAACATTGATTATTAACGCTAAAAAAAACTGAAATAATATAAAAAATATTGAGGTAAGCCACCAAATTGTCTAGCTACTTCCACAT<br>TATTTTTCTATATATAGAGAGATAAGATGCTTGGCTTTTCCTTTATATATCTCCACTCTCCTTTCCTCCTTTATCTCTCTTCGCTTTATCT<br>CAGTCTCAATCTTCTCTACATTTCGAAGAAGCTTCTCAATTAGTTGAGAT
CsDof30	Csa06g021150.1	TATTAACAAATCACAATATATAACAAACTAACAAATGAGGGAAGAAAAAAAAACACATAGAGATATACCTTTGTCTGGGACATGATG<br>AAGAAATTTGTATCAATTATATATTTTGGAGAAGGAGGATCTTGACCCATAAGATCATATTCCTTGAGCAACCTTGGCACATCAGATCT<br>GCGTAAGTCAAGACCAATTGACACTCTATTGTCGAGGATATCGCTGACCTCACTGTCTAATTTTTTTTTAATTTATATTTATCTAGAGA<br>CTTGATATCCATAAGTAGAATGGTTGACTGGATTGTAAAACCTTTTGTTTCTAGCGTCTTAGGAGAGATAAGCAATGCACTGGAATAC<br>AATATCAATTCTCCAAATTTATGTTCTTTGAATTCTTTGATGTTATAACATACTTTTGAACTCTGTTTTACCATGTTGAAATGCTTTTAT<br>TTTTATTTTCTATTAGATATAATGTCTCCCTAAAAAGTTGTCTCCATTGTTGAAAAAATCTTGCATTTAAATATATATGATGATCATGTG<br>TAAATCAATTGAAGTGGAAGATTCTGTTTGTTATCTAGTAGAAAGCTTGTAATCAAAATATTCTTTTGTTAGTTATGTTTAAAACCCCA<br>TATTTATGATTTTGTGTTATGGGTTTTCAAAAAAACTTAATTTATATAATAAGATTACTTAATTTTTTTACAAAGGTTTATCTTGTATAT<br>TTGTAATCCCATATATATCAAAAACACTTTTGGACAAAAAAAAAAACTATTGATACAAATAAAGTTAGAAATTATTACTAGGGACTTAT<br>TATTATTATTAGGGTGAATTAGTTGTATAACCAAAATCAATAATATAATTAAGAATATAGCACTATAATTGAAGAAATTTGAAGAATA<br>GGATGAAAGGCATCCACAATTACTATTTTAGTGTTTTAAAGATAGGAATGATGGTGGTGATCGACGATGGTGACCAGAGGTCGGCGA<br>CCGGCAATGGTGGCTGGAGACCAGAGTCCGGCAGTGGTGGTCGGATTCCGGCGGCAGTGGCTGGAGTCCAGCGGCGATGGCCAGAGT<br>CCGGCGGCGGTGGTCGGAGAGGTGATGGTCACCGGCGGTCCGGAAGTAGTGACAGAGGTAGGGAATGATGGTTGTAAAGGGTGGGA<br>ATAGTAGTGATAGGGGGTGGGGATAGTGATAGTGGATGGTGATGATAATGATAAAATAGTATATATTGTAGTTTATATAGAGTATATT<br>ACTTATTGAAATATAGTTTTTTTGTCGGTGATAGAATGCAATTCCTTTTATTATTATTATTATTATACAATAAATATATATTTTTTGAAA<br>AAGAACCCGACAACCCGATCCGGTAGTAATGAGGGAAAAAGAAAATATCGGAGGCCACATAAACTCGTGGCTGAGAATACAAATGTG<br>ATGCTGACGTGTTACCCTCCTTCTCTTTTATACGGATTCTCCCCTTAATCCCTAATTTTCATATCTATTCACACACACTCCTCCCAAACT<br>AAATCTCCCCCAACAACAAAAAAAAAAAAAAAAAAAAANACTAAAGTTTGTTCTCTCTCGCTCTATCTAAAACTCTCAGTTTCCCTTCA<br>CACATATTAGACCATAGGTCCATAGTAAAATATATATATATATATATATACAGTATGGGTGAATTGGTATACATATTTTGTTATGGTTC<br>ATAATAGTAAATTTATTTAGTTGTCATGTTAAACAACAACAATATTTCTAATTTACTAAGGAATAGGAAAGAACTAAAAACTAGTAAA<br>AACAATTTAACAGTTTTTACGGATCACCATAAGAGACATGCGCTAATAATTACATACAACTTATCTATATAATAAACCAACTGTATGTC<br>AGGATTTCAAATTCCAACCATAAAAAAAAAACTCAAAAAAAAAAAAAAAAAAAAAAAAAAAAAAAAAAAAAAAATTGACTAGTGACTAGTG<br>AGATTACACGAAGCCTTCTCGCGCCTTTGCTTTTTTGGTTGAGGAAAAAAAAAAA

Name	Gene ID	Promoter sequence
CsDof31	Csa06g024450.1	TTTACTTTAAAATCTTAGTATAATAAAATATATATTGCTTAGGTTTAGTGTGTTTATTGTGGTCCTTGAGAGATCAAACTCCACTACAA<br>ATTTGGTTGTTGTATATTGACTAAGATAGATTCTGATTTCGGTTGTCATAATCAAATCAACGTAATAATTTCTAACGCGAATGTAGTTA<br>TATAGTTAGATCAATGATCATTCATAGTAATACAAATTTAGTGATGAAAACACGAGCATAGTCCACACCGCCCACCGCCACTACCGTT<br>TTAAAATTAAAAACACAAAAATTGATAAGCGACTGTGTCACCTGTCTATATACAGTTTGAATTAAAATTTGTAAAGTATTCGAAAGGG<br>AACAATCGATAAATAGAGTAATAAACAAACAAAAGGTTTCTGGTACATAACAATATAGTATTGTCTTTTCTAAGACAGCGAACAAATG<br>AAACAAGATCAATTTTTTTTGGACTTTAATTTCAAAAGCCTGCGTGATTGATCCAACAAATTATAAAACAGTACTTTAATTTTCGTTCT<br>CAAATAAAGTAATACAATAAATCCAATTAACCATTTTTCAAATATTTTTTTGTAACGAAGACGATAGAAAAACGTGTCCCTTGTTTCCA<br>ACTTTCTGGAACATTCATTTTATCTTTTTTAATTGCCGATGATTTACGCTTACGACACAAAAATATTGATTTTTTTTTATGAGTTACACA<br>AAATCATTCATTTGAATAAAAACTATACTATAAGTTAGGAGATTTACGAATACTAAAATACTTGTTTTTTCTTTAGCAAAACAAAATAT<br>TAGTCAAAAGCGAAATCAATAGTAATTAGTTATATAATTATATAAATTTATTGTATATAAAATATAAGCATGAAAAATTTAAATAAAA<br>ATCGAAAGATTCTCAAAAGATAAATGTAGAATTACTTGGAAATGACCGTACACATTGATAACCTCAATAGTCTTTTACCTGTGTTGCCT<br>ACTGCGACCTACTCTATCATTTTGTTTTTTTTTGGTTTTTATAATCAACGTGTCTTCGTTATTACACAGAAGAAGATAAAAATTACTTAT<br>GTCATTATTATTAGTTTTGAGTGTTTTGACTGTTGTTCATTACTAGTAGTAATTTAAACTTTAAACATATATAAAAGCAAACGTCATGA<br>CCAAACTTTCAACATTATTAGTCACATAAGTACAATAATTATCTTTTTCTCCTTTGTCAAAAAACTGATTTATAAGGTTGGTAAAGACA<br>AGACTAAAATATATATCATGATGTTTTTTTCAGATGATTTAGGCAGGTACCAAAAATTATATCAACAAAACCACAACGGTGAATCATT<br>ACCTCGTTGAAGTTTTTTTTTTGGTCGTTTCTCATTTTCCATATGCATCAAATATGTGAGATAGTGTGCCACACTACACACTATAGTCAA<br>ATATTGCGACGATCATAAGAAAATATGTTACCTTCATGTTCACCACCAGGCACTTAAGTAATTAATGTATTATTAATGTGATTACTTTT<br>AATGTTTTTTATTCATTGTTCAAAACTGAAAACTTTGAAAACAGAGCCGTCTACGTATGAAGAGAGCCGACTTCGTAGTGTTCATTCAT<br>AGGGATGAAAAAAACAGAGCGTATCACTGTTTACAATAACCCATAGCCGACTCAACCAATTCCTAACAAAAAAATCTTTATAAAAAGA<br>CAAAATTAAAAGACAAGAGCGTGGCCTCTAAAATTAACCAAACACCCTCTGTCAAAAAAAATAAAATAAAGCCTGAAACTTTCTTCTC<br>CATCCCCATTTCTATCTGCGCATGTCTCTCTCTCTCTGTCCTCTTCTCTCATCTCCATAAAAACCAAAAAATCTCACTCTCTCCGTCT<br>CTCGTACCCTAAAGTTTCAACCTTTACCTCTCTATAACAAACCCAAACAATAAAGCTTCAACCTTTAACCTTCGATTCCCGCTCTTTCAA<br>CTCGCATAAAAAAAACAAAAAAGCTTCCATTTTTTCTCCGGTT
CsDof32	Csa06g026770.1	CTCACATGTTTCTTCAATCTAGAAGCTTTCTTGGTGGGAATATACCCAACAATAGCTATGAAGCTGGAGTTGCGTTAGCCGTTGAAGCT<br>CTAGGATTCTCTAATGATGAACAAAGAGGTGTATTAGTCAAGGAGTTTATAGAAAGAGCTACAAGAATTGGTCGAGCTCCGATTCTGA<br>GGTCGGCTGAGTTAGCTAATGAGCTTGCTAGTGTCTTGCCAGCAAGACTCGAGATTCAATGGTACAAAGACCGGTGCGACGCATCAGA<br>GGAGCAGCTTGGTTACTACGATTTCTTTAAACGTTATTCGTTGAAAAGAGACTTTAAGGTGAACATGAGTCGCAAAAGACTAGCTATG<br>TTTTGGGACAGAGTGCTTGAAATGGTGGAGACGAACGAGTTACCTTTTGATTTTCATCTAGGGAAGAAGTGGATTTACGCATCTCAAT<br>TTTATCAACTCTTAGCTGAGCCACTCGACATCGCAAATTTCTACAAAAACAGAGATGTAAGGAGCGGTGGACATTACTTGGATGAAGG<br>GAATAGACCCAAAAGGTATGTGGTGATTGATAAATGGTGGAAAAGAGCTGGAGAGCCTGAGAAGTGTGTGAGAAGCAGATACACTAG<br>CACTACGCAAGATACTTGCTTTTGGGCTAAGCTTGAGGAAGCAAAAGAGTGGTTGGATGAGGTGAGAAGAGAGAGTAGTGAGGCTCA<br>GAGGCAATATTTGCTACGGGAAAAGATTGTTCCATTTGAGAATTATGCCGACAAACTGAAGAGAAATAAGGAGGTTTCTGTGGATGTT<br>TTGGCGAAGAACTCGAGTTACATTATGTGGGTGGAAAATCTAAGAGAGTTCAGGTCGAAAATGGGTTATGGAAATGGAATTGAGATG<br>GTTGTTGATGAAGGTGACGCAATGGAGACTTAGTAGGATTAGTAGCGTATCAAATGTTTGATATGCTATAATAACAAACTCTGTTAGT<br>ACTGCTGTTCTTCATTTCATGTAAGACTTTAATTTCTGATTTATGTTATGTTTATCATTAATGCTCAACAAGTTTCTGCAAGAGTTTCTA<br>TTAATGTAAAGTTATTACTCTAATAAAACCAACATGTTATATAAGGTAAGCTAATTTTTATGATTTTTTTTTTATATAGATAGAAAAAC<br>GTAAGACCTACATAGGAATCTTGGAGATTGCCATTGTTCACATGATCCAAGAAGCTCTTTTCCCCATTCAGATTTTCCTTTCCAATCCC<br>CCTTCTTTTGTGGGTAATTAATGAAACTAATACAAGTCAAAGTATACTAATAGAGACAGCCCTTGTTCTTCAAAACTTGTGCTTTCTTG<br>TTTCACATCCTCGTTTCTTAGTTTCTTTATATTCACAGATCATTACGCATACCTTCATACCACACGACGATATATGATCTGTCTACTTA<br>CAAGTTCTGATCCTTAACGGCTTTGACTAAATCTTTTCATCGTGTGAATTTCAAATATGTTTCATATATAGTACTCGTTATAATTAATTC<br>CTACCATTATTGATATAAACTTTTGCTTTATTAAAGTGATTTTAATTACATTCTATTTAGTAGACTTATAATATAGAAGTCGACAAAAG<br>AGAAGACATATATAGTAATCGTGCAGCCTCCATTAACTAAGCCGTACAAATAGCTCAATGTACTCCAAATAACCCATCTCTCCAAAGA<br>GGCCATAGGCTTCTTAGTTCGCTTCGAAACAATCTCCAAGACGATCTATTAATTAACCATTTTCTTATTCATATCTTATTTTTTCAAAA<br>CATATACTGGTATTTAACTATATATCAAAGATTCAAAACCCTTAACCCTGTCTCCCCCAAGAAGCAACAGTGAGCGCACCCTTACGAG<br>GCATTTGCTCCCCCACACTTCCTCCACTCTCTAGCCTCCACGTGTTACACCCTCTCCACATGCACAAACTCACACACACACATTCATGC<br>ATGCGTATATAACACATTCTCCTTGTGAGAGAGGGAGAGTGGAAGAGAGAGAT
CsDof33	Csa06g029980.1	TTTGATTGACTATTAAAATACAACAATCTATATTATTATTCATGAATTAATTTTATGTATGTCATGTGTATAAGTGAATTTGTCTATTGT<br>ATTTGTAATTTATTGTTAATAATGTTTTCTAAGTATTACAAATTCAATTCCAATACCATAAAATTTTGAATATTCGAAATTATTGTTAAA<br>ATTGTCTCAATTCATAATTTTAAAAAGTTAAACTATTAAAAATATATCTATAAATTAATAATTATTAATTTACAAATTAATTTCACTATA<br>AATCAATAAATGTATTGGTCCCAACATTATTAATTTATAGAGGTTTTATTGTAATTCGAAATATATTATTTTCAATCATTCAATGTTATC<br>GTTAAGTAATCACGAGGTGGTGGGCTAGTGGTTTTAGTCTTGGTAGAAGAGTTGCCCTGTTGGTCTAGGTCAGAGATCGATTCTTTCC<br>AAGCGCAGAATTGCTCAGATTTATCCCTTTAACTACATGAATATAGATTCATTGCTTACAGTCCATTTGCATACCCGAAAAAACTCTAT<br>AGGATACCAGAATCAGGAAAAAAAAGAAAAGAAATAAACAACTGTGTAGAATCATCATGATTTGATTTATTGGCTTAATTATAAATTC<br>TAGTTTTTATTTATCTTGTGTTGTAAATTATCCAAATCACTAAATCAGTACATTATAATTTCTTCTTGATATCGAAGTTCAGAAAAAAA<br>CTGAACGATTTATTAATTGGATCACAAAATCTAATAGTATTATCATTTATTTGAAGGAAACCATATACATATATAGAATCATAAGAGTT<br>CTTGGATCATTCTTAAGACTGACCACTCGGAATCTATAAAATCTCGCCATGCAAAGGTAATCTATTTTTATAAGACGAAAAACACAAT<br>GACGAAAAATTATAAGAAAGATAACTAAAATAATACTATGAAAAACGAAACTTGGGTCACACCCGAGGAAAACAAAGGTACACTCGA<br>TTGTGACAACTCCACCAAAGACCACCCCACTACCAATTCACCTTTATTTGTTTCTTTATTAATTTCCAAATCTTTTTGTTTTTTTATATAT<br>TAATCTAGCTAATTATTCGCCTCTCTCGTTGTTTTTTGACCTTTTAATTAAAAAAATTGAAGGGAGGTGCCTAGGGTTTCTCTCTCTGCA<br>TGGCCAGTACTCTTGCGCTTCACATTTCTTTTTGGGCACCACTGTCTGTAAACGTATGCAAAGAAAGAAAAAAAAGCTTACATGCTAA<br>GAAAACTATATTTCCTTGTCGTTTCTCTCATAAAAGAATTATATTGAAATTTATCGGTGCTAATGAAATACAATATATGTAGAAAGTGG<br>AATCGGATCGGACAAGGGAGTGAATTATCTACCAACTTAGATTTCAATCGTCTTTTTCTCATTGATTGACAAGTAACATACACAATATA<br>CTAACACAGATATGATATCCTTATAAAAAATGTATATATAATTACTTAATCAAAAATACACACATATGATATCATAGTTTCATTCAACC<br>AAATGTTGTAGTGAAATTGATCAATTTCATACGGAGCTATCAGCTATGTGCGAGATTAATATTAGTATAACATATATGAAACAAATTC<br>ATACTTGATATAAACAGTGATACAACCAAGACACACACAATGACGTCGTAGATGATTGACTTGCGAAAATAAGCAAAACAGAAAACT<br>TCTTTAAATCGACACTTAATTCCAAAAAGGTTAACAATAAATAAGAAGGTTTTTTCATGAAAACAAAAAGAAATAAAAGAGACTAAGA<br>GAATGATGAAAATTGAAAGAGAAAAGGAAAAATAAAATAAAAAAGAGTAAAGAGAATTAAGAAACACAATAAATTAAACAAGGAAA<br>CTTCATTTCATCTTCTTTTTATCTCATTCAGCTCCTCTCTTCTCTCTCTTCTCTCTCTAGATCAATTCTTTCTTATATGATGTGATTTTCCACC<br>ATATCTGCGACCTCTTACCTAAAAAAAAAAGAAACCATTTAAAG

Name	Gene ID	Promoter sequence
CsDof34	Csa06g040340.1	CTTGAATCTAACGGTTGAGAATTTCTTGGATATGGTCCAAAGCGGGGCTCCATTCGCAAGGCAGTTTCAAGAGGATGATCTCGTACTC<br>GATAAGATCGACACCGAACTGTTGGGTCAAACAGACGATGGATTGGGGTTGAAGACTCTTGAAGTTGTTAAGCCAACAGTGAGTTGG<br>AAGAGGTTTGAGAAGCTCATGGTTAGGCTTCTAGACCATGAGAATTTCAGGGCTAAGCAATGTAAATAGTTTCTTTTTGTGTTGCCTA<br>ATAACTCTTGATTATTGATATTATTATTCCAATCATTAATTGTGTTACCTACACTTCTTTTAGACTACACTGTACAATATTGTACAAGTT<br>TCTACACATTACCAAATTATAGTTTGTTTGTGACATAGCTCCTCTAATTGGTAAAGCTGCAAAAGTAATAAAGTTGTAGAAAATAAAAT<br>ACGTGGAACGGAAATAACAATGCGAAAAGAAGAAACAACTTGACTATACTATTGACTGAAGATATACATTATGTGCATATTTGTAAAC<br>TTGGAGAATGATTGCACATACGACTATACGAGTACGAAATATAAATGGTAACAGTTTGCTGGTTGCTTAAACTTGAATTGATAATAAT<br>TGTGGAACTGCATAACCATATCCTTTTTTTTTTTTTTTGAACGAAAACATAACCATATCCTAATTTACAAAATTGAGAGCCATTACTTA<br>CCAAACACACAAAATTTGTTTTGTTTGCCGTTTTGTAGTTAGCCGTTTTTAGTTTACGAGGTGATCCATATATCATACTATAGATGACA<br>GATCAAATCTATCGAAATGTGTGGAGAATGTTTTCGTGGTAAGTAAAACTTACAAAAGAAACCTCGGAAAATGGTCCCAAGGCTCAGT<br>GTTTTAGAGTCACGCGGTTCGAGGGAGCGTGATGATCAATAACATCTACGAGAAGATATTGCCATGCAGAGGAAAAAAGCAGAGAGA<br>GCGAGAGAGAGAGAGGGGGAACATGATGGACCGGTCGCGATGCTTCGGCTGTCGTCCAACGACCAAAGCACCCATTTTAACCCACTT<br>TTCCTATTTTCAATCTCTTTTTCATTTCTTTTGTTTTTCAAATTATTAGAATAAATCATTATATGATCAACGATCCTATTATAATCATTAT<br>GCTAACACCTTGGAATTCGGTCTACATAACACCTTTGTCTCCTAAATTCACTCCATATATTATACTCGGATCAATCTACACAGAAATCA<br>ACTATATATCATCACTATTTACCAGATAAAATTCGATTCTTTTAGTTGAATCTAAAAACATAAATTAAAGTAACACATTGTGGTCATTT<br>TTCTAAGGGACAAGGGTTAACTTTTTTACATAAGGACTTCATCCAGTTTGATTACGTCGGTAACTGAATTGTTTTCTAACTAATTAACA<br>AATTAATAATTTTCATCTTGGATTCATAACAAATTAACAACAATTGTGAAAGCTTATGTATTAAGACAATATAAGCTTATGACCGGTGT<br>ATGGATTCATCACAAGGCACAAAATAAAAGCTTATGTATTAAGAACATATACTTACATATATATGAAATGAAACTAGTGATAGGAAAT<br>TTTAAACCCTAACTAAAGCAAGAAAACAAAAAGAATCATGTCAGTCCCACCCCATATGGAACACAAACCCTAATAATTACATATATGA<br>CAATTATCTTCTTACATCTCCATACAACATTATTATATATATATATATATATATATTGACATAGATATATATAAAGGTTCACCCACTTTA<br>AATTTTAGCCATCTTCATTCTCACACTCAACATTCCTTTCTCTCTCTTTCTCCCTTCATTCTCATTCTCTCTCGGCTTTGTTTTCTCTTTGA<br>TCTGATTCCTCTATTCTATTTCTAATCCATCACCCACCACGGAAAAGAGAAGAGGAAAAAAGAAAAAAGAAAAAAAAAACCTTACTTT<br>AAGAATTTTGAAGAATAAAATCAAAAGAGAAAAACAAAGAGAATAACA
CsDof35	Csa07g002770.1	CTAATTAGTTGGTGGGATTCTTGACCAAAAAAATTATATGTGGTTAAACCCAATTAAATTAATGGGCCATATATTAGAAAGGCCGGAG<br>AATGGATCAACGGAAGCTTCTTCTAGGTGATTTGGTAGAGAACACGTGGACCGTGGTAGGTCGTTCAAGACTGAAGAAAACGGCAGA<br>CAAGGTAAAGCTAGAATAACAAAACGACACGAGAAGTGGAGATGATAGATTGGGTAGGAAAACACAGTTGTCAACATATTAAAAAAA<br>ACAGTCTCTTAGATTTCAAAAAAGCTAATCACGCCCCAAAACTGGGTTCCACCATCATCCTTTTTGTCGCTTCCTGTCGGTCCTCTTCCT<br>AATTGTTTTCCAACCCCTCATCTTTTGACTATATACATAAATAACCGTTTAACTTTCTTTTGTTAATATTAGACCAAAAAAAATTCTAGA<br>ACTGTATCGATGTGAAAATTAGTTATATTATAGCCCATGAAATTTGTTTTTGGTAAAATATCCCGTTAAATTTGTGAGGCTTAAGTAAA<br>AGGTACTGTTTAATTAAATTCCTCAACGGTCATCTGATTACATGAATGTGATTTTAATCATTCTTAGTACGTAAAGGAAGTTGCACGTT<br>GGGTAATTAACGTTTTAATAACACGTGATTTTAATATCTTAAGGATGATTACCATTTATAAACGAAAAAGCTATATATATAACATAATA<br>TAAGTCGTGCAGTATTGTTTGTTTGAAAAGTTTCTTTGGTTTCTTATAATTGGCCTCGATTTTCAGTTTATGTGCTTTTACAAAGTTTTT<br>ATTTTAGTTATCTGATTAACGCGACATGTGTTGTGTAGCTTTGCCTATTTAAGAATAAAATCATATTAACTTTATGAAGCCATGTGTGG<br>TGTAATGTGTATAGTGGTCCTTTCACGAAAAAAAAAAGCAGATCACAATTCAAATAAAGGGTGAAATTTTGGTGTACCTTTTTCTTCGT<br>CGAAACAACATAACTAAAATAAGAGAAAGCATTATTTGCGTCCGAAGAATACTCCATATTCCTGTAAATACAGTCAACATATATTGTA<br>CTTTATATAAAGTTAGAAATTTTTAAAAATCATGTTTTATCGACCATATTAAGTCACAACTTTATCATAAATTAATCAGTAATTAGAAT<br>TTCTAAGTTCGCCACCGAATTTCGTAACCTAATCTACCGTAATAGATAAAATATACGGAATTATGAGATCTATGTAAGGATCTTGAAAT<br>TGCCAAGGTAATAATATATATCGACGGATCGTAACCCAACATATAATATTAATTACAAAATGGGTGGGCGGGAATAGTATACAACTCA<br>TAATTCCACTCACATTTTGTATTATTATGATATGAAAATTAATCAACATGCATAAAATAATACTATATTCATCTGTATATTATCGTATTA<br>AAGATTTACAGTATCATCTTTGAAAACATAATTACACATGGTTTAGTGACAGAATTCAGTCCCAAATCATGAAATGATGACAAGTGAT<br>TCAAACAAATTTTAAACACCACGATCACGATGCGTATAACACTGTAATAATTACTCTATACTGAAGAAGATAAAATCACTAGTTTATAT<br>ATTAAGCCAACCAATTTGTTAAATAGAGCAATCAATATTTTAGCCAGATATTTTTTTTCCTTTAAAAATATAGTATAAAAGAGGGACCA<br>GAAAAGAACCAGAGAGGGAGGCCATGAGACATTATTATCACTAGTCAAAAACAACAAACCCTCCTTTTGCATTTTTTCATGTAAATTA<br>TTATTATATTTCAACTTTGCAGGTTTCTTCTCTTCTTCTTCTTCATCTTGGTTGCTTTCTTTCATCATCCATAAAGTGAAAGCTAACGCAT<br>AGAGCCATATCGTCCCAAAAAAAGCAAAAGTCCAAAAGAAAAACTCTCTCCAAAAACATTCTCTCTTTGTTCTTTACTAGCTCTTTAGT<br>TTCTCTTTTCTCTCTCTCTCTCTGCCTTTCTCTTTGTCGAAGCTC
CsDof36	Csa07g007240.1	GGCATATTGTCAGAAAAAGTGTTTTTGCTTAACTTTATCTTTTTTTTTTTCTCAAAGCAATTTCCATTCAAGACACTCCCTCTTTCAATC<br>AAATATCAACGCATTAAAGTATATATATGTGAATCCAACTTGACTTGACTAACGGACCGGATCATACTAGCTAGGTTGATGACCAAAT<br>CTGTATTGATCCAGTTATTGACAATCTGATTCGTTATAAACCGGTTCATCTAAACATGTACCATAGTTTCAATCTGGCCGCTACAATGG<br>GTGTATAAATTTTAAAACCAAAGAGAGTAGTTTATGAATTATGATTTATGAGGAGGCTATACATTGAATGGGATCCACAAGAAGAGCT<br>GACAAAGGGTGAAAGATGTTTTATGAATAAACGATGTCTGAGTCTCAAAAGCTCACACTTTTCTTTTCTTCTCTTTTTCCTACGCAACA<br>CTGACTTATTACCAAGCTTTTTCTTTTTTGACGGCTTTGTCTTTTAATATCACATCTAAAACATAACTAATACTTTTGTTTTTTTTCTTCA<br>ACAAACAGAATTAATTCAAGTAAATACAAAAGTTCATCAACTGTAAAGATAGGAGGAAAATCAATCCAACGGTCTCAAAATTATAAAA<br>ATAAATAAAAGATTAGTATATACCCTATAAGCAGAGCCGGTGACAAGGGGTGCAAGTTTTGCATTTGCACTGAGATCAATGTTTAATT<br>AACATAAAAAGTAGTGTATTTTAAAACTCAAAAAATATTTACAAATTTTTTTCTATGTAATTAAAGTATACTCCCTATGTTTTACAAAG<br>AGTGTCATTTTAGAAAATTTTTTTTGTTACACAAAGAGTGTCATTCTACATTTCCAATATATTTTTTAATGCTATATTTTCTTTTTTACCC<br>CTAAACCCAAAACTTTTTTCATTGAATATAAACTATTAATTACTTATTAAATAAGGGCAAACATATATAATTTTCTTAATTCGTGTGAA<br>ATGTGCGAGAATGACACAAGGGAGTATTTTCTAAAAATTGAACAGACAAAATATACTACTACATACACACAAGTTCATCCAGTTTTTG<br>AAAAGAAAATATATATGGGGCAAAACTCCTAGGTGTCTAGAGAGTTGCACATTTAATTTCTTCTTCAAACCTAATGATAATAGATTATT<br>GTTAAAGTGTTAGTAAAGTGGAACTCGTAACTCGTGCACTGTGAATTCATAATCAAATCCGTAAATTATTAATGCAAAAGTCTTACCA<br>ACACATATTTTTGCTTCTCTTTATAACCCTTTTGTTATTTACTACATATATTGAAGGAAAATATATGTAGCTAATAAAAACTAGGATCA<br>ACTATACTTTCGCTAAGACAGAAATTCTAACTTCGTTGATAATCTATGAAAATACAACACACGCAAATTCGGATGTGAATAGATGGAT<br>CATGGGTATAGAATCTCTTTCAAATAAACTGAGATTACTTTAAGTTGGATCACGTTCAAGCATCCACTAGTCTAAAGTCTAAACATTGT<br>TACGTAATAAATGTATTTTTCATTTATTTCATATTCGTTTTAATAGGAGTAAAGTGGACAAAAAGGGAAAACGTGTTGATAAGACTTCT<br>GCGCTCTATACTACTTTACGGACTCGATGATGGTTTTACAGTAAACTACTTTACTTTCCAATTCTTCTCTCTTTTTTTTTCCTTTCAAATA<br>AATAGCTTTTTAAAGAAAGATTTGTAGCTTGATCTGCACTGGTATTCGTGTTTTACGTGTACATATGAATTCTAGACCCAAAAGTAAAA<br>TAAAATAAAGCGATGAATGCGCCTAATACTATATGCTTTTCAACTTTATCCAACAAAATATTTTATTTAATCTCCCACTACTTCAGTCG<br>CTTTTCATCTCCTTTTCTTTCTCCATTCCTGCTAAAAAGTTATACTATCATCTTTCTATAAAAAGACGTCTTCTCCTATTTCTTACCTGCA<br>ACATACTTTTCATAAGCTAATCTTAATTTCCACTCTATTC

Name	Gene ID	Promoter sequence
CsDof37	Csa07g015680.1	AGAGACAAATTGAAAGATCTCTCTTTTGTTTTTCTTTTTCTTTATAGATAAAAATAAAAGTTGGGACATAAATTAGAAGTTGTAAATGA<br>CAGGTTTCATCTTTTTCATGCTTTTTCCCTTTATTTTTGTTCGTTTGATACCGAGTAAAGTTAATATCTTCATATTAAGATTCAGTCATC<br>CTTGTTAAAATAAATGTTATTATATATTACTGAAATATTAATATTCTTAAGGAACAAGATTACTATCATAGATTTTGAATGGTGGCGTA<br>CTGACGTAGAACAAATAGAGTTTTTGTAGATTAATTAAGTTGATCAACCAAAGTAATTACATTCGAGTCTAATTTTTTTGTTCAAATGA<br>TAAAAAAAAAAAGATTAAAAGGATAGATTTGATGCTAACCATTCAATGGTGGTTTTACTGTGGCTGGTCAACTTAATTCATTTGTTATA<br>TACTTATATTGCAAGATTACAAGCCTATATACTTTCGAATGACATAAATAATTAATATATTAAAAAAATAGATTCAATATTACACCATG<br>GCCATGGAAAATACAAGCATGTAATAGATTGATATCTAATTAGTGAAGGAAGACGAAAACGACAGATCAGGGACAAGGCTAGTCTTG<br>AGTCTTGACCCACAAAGATACCCTAATTAAGCTTTCATGATTACGAATCTATATGATAATGAACCCTATTAAGAAAAGTTAATATTTAC<br>TTAACCATCAGCGTACAATTATATTTACACACACATTTAATTATATGCATAGACAGCGTAGTTGATTAAGTGCACTATTTGCGATAGCA<br>AAACAACTTAAAATAGTAATTATGAAGCATTAACCAAACGTTAATAGTTTAAGTTAACATGGTGTAGCCCTGCCGTGGGTTGGTCTAG<br>AAAAATATGAGTTTATTGGGGGCACGAATATGTAATTTGCTACACTAAAGTATAGAGTTATATTAAATAATAACCTGAAAATCTAGTA<br>ACAACAATAAGACTTTTCTTTAATTTTATTTTGTTATAAAGGCTTATAAAAATTGTTTTTTTCTGCTAACTGAATTATTGTTATTATTGT<br>ATAATTTTGTAAATTTAAATTCTGACTGATACAAATATATAAGTGCAACCAAAAAGATAAGTACGAATAATATTTTAAATATAGTGTGT<br>TATTGAAAAAAAAAAGCAATACTACTTATGCAGCATCGACTCCCAACATTTTAAAATTCTGAACTACATAAAGCACTAGACTATAGATC<br>TAGTGACTGATATATCTAGTGACTGGATAGACCAAAAAGATGTTTTGCATATTGTGAAGAAATGTAATTAAGAATTGTTTGATTGTAC<br>AACTTTTCGCAAGTCCTTCTTATCACTAAACCAGTTTATCTGAACATTTCCCTACCTTATTTTACTTCGTGGTAAAGATAAAACATGATA<br>TGATTGTAGTATTTTATTATAAATAAATCGCTTAAAGCAAATCAAGGTCTTTTGGTTGTATCTACTCTCTAGAACGTCTTTAATTTATTT<br>ATTAGACCTTGTAAATCATATGGAAAATATACATAAAATATATACACACATTATAGATATAATTCGACAGAGAGAACCTTATAGGCGA<br>AGAAATGTCACTCCACTCAAACCCTAAAAAGAGAATCACAACAAAATCAATAATATATATATACACCTATATATATACGCATACACAA<br>ATAAACAATGCATGAGAGTGGGAAATATATATAAAAAGAAATAAAGCATGAGAACATGACCCATGTTTAGAAAAGCATAATGTTCTT<br>ACTTTATCTTTGTGCTCATTCTCTACCATCATCTTCTCTCTAAACCTCTACTCTTCTCTCTTTCCCTTTTGGTTTTGTGCTCTCTCCCTCA<br>CATCTTCTTTAGCCTTTGGTTCCCTTCTTTCGTTTTTATTTTGATTCTCCCACCATTTTTGTTCTTTTGAGTTTTAAAGATCCATCAAACT<br>TTATTAAAGAAAGAAGAACAAAGAGATAAAAGGGAAGAAGAACA
CsDof38	Csa07g016040.1	NNNNNNNNNNNNNNNNNNNNNNNNNNNNNNNNNNNNNNNNNNNNNNNNNNNNNNNNNNNNNNNNNNNNNNNNNNNNNNNNNNN<br>NNNNNNNNNNNNNNNNNNNNNNNNNNNNNNNNNNNNNNNNNNNNNNNNNNNNNNNNNNNNNNNNNNNNNNNNNNNNNNNNNNN<br>NNNNNNNNNNNNNNNNNNNNNNNNNNNNNNNNNNNNNNNNNNNNNNNNNNNNNNNNNNNNNNNNNNNNNNNNNNNNNNNNNNN<br>NNNNNNNNNNNNNNNNNNNNNNNNNNNNNNNNNNNNNNNNNNNNNNNNNNNNNNNNNNNNNNNNNNNNNNNNNNNNNNNNNNN<br>NNNNNNNNNNNNNNNNNNNNNNNNNNNNNNNNNNNNNNNNNNNNNNNNNNNNNNNNNNNNNNNNNNNNNNNNNNNNNNNNNNN<br>NNNNNNNNNNNNNNNNNNNNNNNNNNNNNNNNNNNNNNNNNNNNNNNNNNNNNNNNNNNNNNNNNNNNNNNNNNNNNNNNNNN<br>NNNNNNNNNNNNNNNNNNNNNNNNNNNNNNNNNNNNNNNNNNNNNNNNNNNNNNNNNNNNNNNNNNNNNNNNNNNNNNNNNNN<br>NNNNNNNNNNNNNNNNNNNNNNNNNNNNNNNNNNNNNNNNNNNNNNNNNNNNNNNNNNNNNNNNNNNNNNNNNNNNNNNNNNN<br>NNNNNNNNNNNNNNNNNNNNNNNNNNNNNNNNNNNNNNNNNNNNNNNNNNNNNNNNNNNNNNNNNNNNNNNNNNNNNNNNNNN<br>NNNNNNNNNNNNNNNNNNNNNNNNNNNNNNNNNNNNNNNNNNNNNNNNNNNNNNNNNNNNNNNNNNNNNNNNNNNNNNNNNNN<br>NNNNNNNNNNNNNNNNNNNNNNNNNNNNNNNNNNNNNNNNNNNNNNNNNNNNNNNNNNNNNNNNNNNNNNNNNNNNNNNNNNN<br>NNNNNNNNNNNNNNNNNNNNNNNNNNNNNNNNNNNNNNNNNNNNNNNNNNNNNNNNNNNNNNNNNNNNNNNNNNNNNNNNNNN<br>NNNNNNNNNNNNNNNNNNNNNNNNNNNNNNNNNNNNNNNNNNNNNNNNNNNNNNNNNNNNNNNNNNNNNNNNNNNNNNNNNNN<br>NNNNNNNNNNNNNNNNNNNNNNNNNNNNNNNNNNNNNNNNNNNNNNNNNNNNNNNNNNNNNNNNNNNNNNNNNNNNNNNNNNN<br>NNNNNNNNNNNNNNNNNNNNNNNNNNNNNNNNNNNNNNNNNNNNNNNNNNNNNNNNNNNNNNNNNNNNNNNNNNNNNNNNNNN<br>NNNNNNNNNNNNNNNNNNNNNNNNNNNNNNNNNAGATATAGACTTTGAGAAGTGTATAAGAATTATAAAGCATATGGATTATAGA<br>ATTTTTTTGGTACTTCTAACTTTGTTTTTACATATTCAATAAGACACACACTTCTTTTCTATCACAAAATTAAGAATGATCAATTACTAT<br>CTAGTCAATTGAAATTTTTCCTAAGTAGGATCGCGTATCTTTTTTCTTCGGTTTAAACTGATATTCTTCAATGTCTCTTTTGGTTCTCAT<br>TGATCCATAAGATATTTTCTCTTGTCATTTAGTCTTGGGGATTCAATTCATTTCGATTAATATTCTTTTTTAGATATTTGTAGCATCACT<br>ATTGTCTACTTCCTTTCTTTTTATTTTTACGTATTCTAGATCTTTTTTGGTTAAACAGATAATTTATCAAAAATAAATGTCTTAGAAAAA<br>AAAGAAAAATAAAATAAGAAGAAGACTCCTTACGGTTCTAGTTTAAATATATGTAACAAATAAAGTTAAAATTCTAAGTTTGCACTTT<br>GCACAACAAAAAGAAAGGTTTATCGAGCAAAAGTTTAAGTTAATATATAAGTTGAAGACTTTTGTAAAACATCTCCCGACTCACATGT<br>GAATATCAAAATAATTATTAAAAAAAACAACAAGTAGCTACAATTAAGAAATAATTTGAATTATTAAAAAAACCATAGATTTTAAAAG<br>GACAGACTTTATCTACTTTGCTTTATATATTAAGAACCGTGCTCT
CsDof39	Csa07g036310.1	AGTATTGGATTTGGTTTTCTCTGTGTTAGTATTTATTTTCGTGTCATTAGGTCATTTTAGGTGGTTTAAGAGATGATTTCACTCTGAGTC<br>GTGGTGTGTTATCTATCTCATTGTGATTAAAAAATGCAATGTTTCTCGGTTAGGTAGCGTTGGATCCGCCTAGTGATTCAGAGTTGTAC<br>TTGATCAAACATTGCATTTGGTCCAAGTATATAAAGGTGAATGTTATGTACTTTTGCTTTGGTTGTATCATTTTGGTTAATGAAAAATT<br>GATGTTGAGAAAAAAAAAAATATATATATATATATATAATTATGATAGTTATATGATAATGAAGTATGTAGTATATGATAATTTCCAAA<br>ATATTTTCGTCACTCAGACGTCATAGGTTTTATAGTTTCTAAAATATCATTTAATGTTTAAAAATAAAGTGGTATAGTACATACTCTTTT<br>TTCACAATAGGAACATTGAACCTTGGCATTCATAAAATGATTTTAGGCTTTCATTATGTCAACCTTAGTCACAGCAACAATGCGAATAT<br>AGTGGACAGTAAGTAGTAAGTAGGATTATAATTGCAAAATACGAAAACAAATGTTATAATAAGAAAAAGAGAAAAGAAAAAAAAAAA<br>CACGATCGGAAAAGACTTAATGGGATAAGAGAAAAAGATATGGTCCAGCCTTATCTCATACCTGGAAATGCCACGTGTCAGACACTAC<br>CAGAACATAGCCTCTCTTACCACCACAAACTTTTTTTTCTAATTCTTTTTGATTACTTTCTTTCCCATCATTAACTAAAGAACACAACGA<br>ACGATGCTAGCTAGGTATGTTTGGATTTGGATACGCTCACGCTACTATATATGAATAACCCTACAAGAGAATATAAATATGTTCCCTTA<br>ATCCACGTGTCGAATACGCATTGGTCACATGCGTGACGAACAGCTAGATCCTGGCAGATGAACTTTTTTAAGAGTCCTGATGGACCAA<br>TCCACACGTGCTTTTATTACAGCTGACGTGGGCTTTTTATGTAGGATGTTTCGGAGACGAAGTGGGTCATGTGCCACGTGGTTACACA<br>ACCTGGCATTGACAGTTTATGCCTTCTCGGATTACTTGTTGTAACTTGTAAGATATGTATTTTGTTACCTATTTCTGTCGAGCGAGAGA<br>GAGAGAGAGAGAGAGTGGACATAGATAGATACCTAATTCTGTATTAACTAACACCCGATTAGTAACTTTTACCATGATTAAAAAAATC<br>GCAACCAGAGAAATTCGAACACTTGTTTCTTAACTCTATGTTAAATCTTTCAACCGTTGAACTATTATACTATGAAGTTAGTGTTTCAA<br>AATTTTATGTTAATAGTTAAGGAAGATATTAAATACATACGTCAACGAAAGTAATGTAACAAGAAGTTTCGATTGGTAATATGAACAG<br>AACTTGAAAACCCTTCTTTTCCTCAAATGTTTTTGTTTATTAGTATGACTTTCATGTTACGTACATCTGACTTTTTTTTTATCATCCGTTT<br>TGGGTACGTTACGTTAACACAATTTTGAACAAGCAGTAAGTAACTAGATAATAATATTTTCTTTCTTATAAACACGCTTTTTGTAATAA<br>ACCAGAGGACACTACGAACCAAGTGTTTTTTTTTTCCTCTCTAAATATTCCTTTTAATATCCGATGTGGCAACAAAGAGAAAGGTGAGA<br>AAATCTTTTAGAACGCCACTAGAAGAGGGAATCGAAAAGAGCGTGAAAAAGATCTCAAAATGCCCACTTCCTTAATAAAACATGTGG<br>CTCTCACATTTTCACAAGTCTCTCTCTCTCTTTCTCTCTATGCTGACTTGTAATTTTCTCTTTCTCTCTATCTATCTTCTTTCTTCTTCTTT<br>TATGCACTATCTATCGTCATTAATACCCAACCCCAAGCCCCTTGTTTTCTCTCCATGCTCCGATCTTTAAAGAAAGAATAAAACGATTT<br>AAACCTTCATATACCTAGCTAGAAGTTTCCATCATAATTTAGTT

Name	Gene ID	Promoter sequence
CsDof40	Csa07g036310.2	AGTATTGGATTTGGTTTTCTCTGTGTTAGTATTTATTTTCGTGTCATTAGGTCATTTTAGGTGGTTTAAGAGATGATTTCACTCTGAGTC GTGGTGTGTTATCTATCTCATTGTGATTAAAAAATGCAATGTTTCTCGGTTAGGTAGCGTTGGATCCGCCTAGTGATTCAGAGTTGTAC TTGATCAAACATTGCATTTGGTCCAAGTATATAAAGGTGAATGTTATGTACTTTTGCTTTGGTTGTATCATTTTGGTTAATGAAAAATT GATGTTGAGAAAAAAAAAAATATATATATATATATATAATTATGATAGTTATATGATAATGAAGTATGTAGTATATGATAATTTCCAAA ATATTTTCGTCACTCAGACGTCATAGGTTTTATAGTTTCTAAAATATCATTTAATGTTTAAAAATAAAGTGGTATAGTACATACTCTTTT TTCACAATAGGAACATTGAACCTTGGCATTCATAAAATGATTTTAGGCTTTCATTATGTCAACCTTAGTCACAGCAACAATGCGAATAT AGTGGACAGTAAGTAGTAAGTAGGATTATAATTGCAAAATACGAAAACAAATGTTATAATAAGAAAAAGAGAAAAGAAAAAAAAAAA CACGATCGGAAAAGACTTAATGGGATAAGAGAAAAAGATATGGTCCAGCCTTATCTCATACCTGGAAATGCCACGTGTCAGACACTAC CAGAACATAGCCTCTCTTACCACCACAAACTTTTTTTTCTAATTCTTTTTGATTACTTTCTTTCCCATCATTAACTAAAGAACACAACGA ACGATGCTAGCTAGGTATGTTTGGATTTGGATACGCTCACGCTACTATATATGAATAACCCTACAAGAGAATATAAATATGTTCCCTTA ATCCACGTGTCGAATACGCATTGGTCACATGCGTGACGAACAGCTAGATCCTGGCAGATGAACTTTTTTAAGAGTCCTGATGGACCAA TCCACACGTGCTTTTATTACAGCTGACGTGGGCTTTTTATGTAGGATGTTTCGGAGACGAAGTGGGTCATGTGCCACGTGGTTACACA ACCTGGCATTGACAGTTTATGCCTTCTCGGATTACTTGTTGTAACTTGTAAGATATGTATTTTGTTACCTATTTCTGTCGAGCGAGAGA GAGAGAGAGAGAGAGTGGACATAGATAGATACCTAATTCTGTATTAACTAACACCCGATTAGTAACTTTTACCATGATTAAAAAAATC GCAACCAGAGAAATTCGAACACTTGTTTCTTAACTCTATGTTAAATCTTTCAACCGTTGAACTATTATACTATGAAGTTAGTGTTTCAA AATTTTATGTTAATAGTTAAGGAAGATATTAAATACATACGTCAACGAAAGTAATGTAACAAGAAGTTTCGATTGGTAATATGAACAG AACTTGAAAACCCTTCTTTTCCTCAAATGTTTTTGTTTATTAGTATGACTTTCATGTTACGTACATCTGACTTTTTTTTTATCATCCGTTT TGGGTACGTTACGTTAACACAATTTTGAACAAGCAGTAAGTAACTAGATAATAATATTTTCTTTCTTATAAACACGCTTTTTGTAATAA ACCAGAGGACACTACGAACCAAGTGTTTTTTTTTTCCTCTCTAAATATTCCTTTTAATATCCGATGTGGCAACAAAGAGAAAGGTGAGA AAATCTTTTAGAACGCCACTAGAAGAGGGAATCGAAAAGAGCGTGAAAAAGATCTCAAAATGCCCACTTCCTTAATAAAACATGTGG CTCTCACATTTTCACAAGTCTCTCTCTCTCTTTCTCTCTATGCTGACTTGTAATTTTCTCTTTCTCTCTATCTATCTTCTTTCTTCTTCTTT TATGCACTATCTATCGTCATTAATACCCAACCCCAAGCCCCTTGTTTTCTCTCCATGCTCCGATCTTTAAAGAAAGAATAAAACGATTT AAACCTTCATATACCTAGCTAGAAGTTTCCATCATAATTTAGTT
CsDof41	Csa07g066190.1	ATGTATAATGAAGAAGTCGCATGATGCATAGCGTTTCCATGCAATAGATGTCGATTTAAAGTCGCATATGGAGAAAAATAGAGGCACT AGAAATGTGTTTCTATAAATGATATCCAACATGTAATGGTGATTTTACAAGGGAGAGCCGTGGATAGTGGATACTCAAACAGCATATA ATTTTGCGTGGGGGAATTTATTATATTGGCACACTCAAACAGCATTTGTGAAAATTCTTGACGTAAAGAAAAAAAAAAGAAAAAATGTG TCTAGTCTAAATGTCATAATAATGATAATAATAAATAGGTGTATCCTAAAATCGTAGAACACTGACTAGGGGGTAGATGCTGAGGTGG ATCGCAATAGAAGAAAGAAGATGGTCGGCTATATCTCTCTCTCGATACGTTTCTTACGGCCAGAAATAAGGCAAACAGGTGGGCCTAG CAAAGATGACTAATCTCCACCGTCCAAACTCAATACACACAAATATTCGTTTTTCTCTCCCACAGTCAGAGGTCAGGTCAGGTCAGGTC GACTAACACACCACGATCTAATATTTTCTTCGCTACTTCTAATACAGACTTACTTTTTTGTTATGTTTTGTTTTGGGTGCTCTTCCCTGT GGAGTCCAAATCAGCTAGAGAGACACGTGTCGTTATCACCAAAAGATTCATTGTGTATATATGGGTCCCAGTCGTCTCTGCTGTGATT CCCCCAAATCTGACGTACGGGCTGCAGTTCGCCGCTGGTCTCATCCGCCAAACGCCGGTTACACAGTGGAACAACGGCTGAAAGATGG AAACAGTGTTGAATCTGGACCACTAGGCTAGATTATTAAAATAATAAATAAAATAAAAAACAAACAGGAGAGACACGTACTTTTGGG TTCCATTCCATGTCAGAAGAAGAGGCATTTCTCTTGCTTGTCAAAGACAACTCAGGCAACTCTTGTTCCTGGTAATAATGATATTCTTC TAATTTCGGCAACTCTCTCATCCTCATCACATGTTAAAAGATTTGTGGTATTCTTCAAGGCGATTATTGTCTTATAGTATATAGTATAA ATGTTTTTAATATATTATTAACTTGATAAAAATCCGGGTCCAAAAACCTCTAGTTACTTATACGGTACCTTAGAGCATCTTTATTGGTA GGTTCTGATTAATTAATCTGGTTACGTTTGAAGAAGAAGAAGAAGAGTTGCGGAAGCAGGGCAAGACTCAAGAGTCAAGAGAGATAC TATTGGATAGTGGCGGCTGCTGGCTAGTGGAAGAAGAAGGGACATGACGTCACCAAGTACACCCTTGTTTTCTAGTATTTTGTATACA TATCTCACTAGCTAGCTAGCTACTAATTAAAAATACGTATGGCTATTTCTTATATATTATGGTCTCCTTCAACAATTTTTTTTTCAATAA TATATAAAAACACGTTTCAAAATACTAGTAATATGATCAAACACAACAGGCTAGTATCTATAACAAAATAGCAAGCGTTTATAATTTA ATGAAAGAAATAAAAGAGAGGAGAAATGGTGAGATAATTTACTGAGGTGAGTAGTAGTAGACTAGTAGTTCTAAAGAGAGAGAGAG AGATTAGTTTGACAAAAAAAAAAAAAAAAGAGAGAGAGAGAGAGATTCGTGGGACGGCGGGGGAGCATAGTTTCTCGTTTCAGACTGT GGTGTCTGTATGTCAGAGTCAGAATCAGAATCAGAGTCAGACCAAACCTCTCTCTATTCCTCACATACGAAGAATCCATCAAAGCACA CACACACATCACACCATATACATGTATCTATCTATTAATTTAAATATACATATATACCTTCTTTAAAAGTGACAAATTATTTATATAATT AAAATTTTGTTTGCTTTTTTCAAAAACCAGCAGCTTTAATTACCTTCCCACACACACTTCTCCTCTCTCTCTTTTTCTCTTTCTCTCTCTC CCAAACCTTTAGCTTTCTCCATTTCTTCTTCAGATCAGCTTCAAACTTCCATAGCC
CsDof42	Csa08g053300.1	AGTCAGTGTTACTAGAATATATGAAAATATTCTTGAAATATCATGAATGAGAGCTCTTTGTGTATGTGTGTGTATATGTATGATACAGA TATCGACTATATCCTAAAAGTGGATGTAGAATATATTAGCACTTAACATTGTTTTTGTGGTTGGATCAGACGAATGTGTATTCAGGACT AAAGCAAGTGGTTCCATGGAAGAACAAGAAGACTCCAGTTTGAGATGCTGCCATGTTAAAGATTTATACAAATTAAAACACCCAAAA ATCAAGAAAGGTCCTCTGGTTTCGACAAATATATCTTCAAACTTCTGTTTATGATTTTTTTTTTTTTTTTTCCAATATTTCCAAGAAAAT GACATGACCATTATAAAAATCATTATGAAGCCAGTTTCTTTCACGCTTATTGTTGTTGTTGTGTTTGTAATGTTTGAATATTCTTAAAAT TTTTGTATAGAATCTTACAATGTATAATGGATATTATGCATCATGTTTGTAACAAAAAAAAAAACAAGAACCTTTGAAGACTCTTTATAT ATGTTATCTTCTTGAGAAAATCTGCTTCCTCAGAAGCTCTTTCCGGTAAAAATAACACACATCGAACTTCGATTCGGGAAATAATTAAA CCAAACAAGAAAACATCAAACTTAGATTGTACGTAGAAACAACATGAAGAGAACGGAGGACCAAAATATTACATTTTTTGTATTAGTT CAAACTTCAAAGATTTCAAGAATCATTCAAGAAAATCGAAATGATCTGGATATCCTACACGTGTCGATTCGAAGTTGATCGATGGTGG AGAATGTGTGGACTCTCACTGTTATTTCGTATCCACTCAGACACGTGGTCACGGTCATTCCTTTGAAATTGAGTGATAAGGCCTCAAAC AAGCCAAGTTAACGTTCATATTTTGGGCTAAGTTAAACATTTGGCCCACTTTAAGCCCGTTGATATATGATGTAAGCCTGTTAAGTGGT ATAAACTAAGGAAAAACATCTAAAAATATTAATTTCTCTTAGGTTCAGTNAAAAAAAAAAAAAAAAAAAAAAAAAAAAAAAAAAAAAAA AAAAACTATTTACTATTACATGATTAACGTATATATTTTTTTCTTTCCCTAATTATGGTTTTAGACTTTAAGTGCTACATTTTTCAATCA AGGTTTTAGCTTAATTTAGTTAGTTAGATGTATAGTTTGTATACTCTAATTTTTTTGGCCTCGAATTGAAAAAAATATCAATATAGTAT GCTAAATATCAACTACTTTATGATGATCTAACCATCTATTAAAAAATAGAGAGAATTAAAAGATAGTCAAAACAAACACACCACTAAA ATGCCCATAAAAGTGAAACCCAACACTTCTAAACTTTAGCCTCAAAATATTCTCTCTCCCTCTCTCTCTCTAAGTCATCTCTTTGAACAT CAGGTCAGTTCATCTTCTTCCCTTTTTCTCTTATCTCCTTGTTTCTTCTGCAAATTTCTTTCTCTATTCTTTTTTTTTTTCCCTTTTAACTT TCTTTTGCTTTTTGGTTTATGTGTTTATTAGGTTTTTTTTTTGTTTTGTTTTACTTTTCAAAGGTTACATCTTTCTACCTTCTGATTTCTTT GAAAATTTGTCCCTTATTTAAAAGATATGTCTGGCTCTTTTAGAGATCAGTTCCTAGCACCTTTTTATTAGTGTCTTGCTCGATCCATAC GTTTAGAAAGTGATTATTTGTTCCCACTCTCTCAGATCCTTTATTGATTAACTTTGGTCGTTCTCATTCATATACTAAACATAGCTCCAT GTATATATATATATATATATCTCTTTAGTTGGTTCATGATCTTCGACTTTTTATGTTCTTTCAATAAGTTTTAGTATTTTATTTATTTGTA TGTGTCACTTTTTTATTTGTTCTACTTTTGTTATCATTCATGTTTTCATATGCTTCAGTAGATGGGTTGATCATACATGATTATCACAGG GATTTATTAGAAACTGTAATTGAAGCCTTACATC

Name	Gene ID	Promoter sequence
CsDof43	Csa08g062200.1	CAGCTCTGCTCATTCTTCCTCCAGTTCTTGCATTGCTTCTGGAACCTCAGGCCCGGTTTGCTGGAACTCGGGATCCATTGATACTCGCT<br>GACCAAGAAAACTGAGATTCTATTCCCAAAGTCTCCAAGAAGAAGAAGAAGAAGAAGAGCAAGTGATGATCTAATTGGTATACTATA<br>AAATTATACATCTGGTTTAATGTTCAACTGGGAGAGACTGTAAAATGACAAATTCATAGGCCAACAAATGTTTGTTTATCCAATTTTCC<br>TTTTTATTGGAACTTTCGTGTAATATCAGAAACCGAGTCGGTTTACTTTTATATGAGATTATTGAATTAAACCGAACGTAGCCGAATTA<br>AACCGATTGGCTATCTGGTTTACTGTACTGTATGTAACTAAAATACTGAACAATTTTAGTTTCCGGTTAGGTCCGGTTCCCGAAATAAC<br>GTTAGGCGATCTATAGTTAGTTTAGCTGTTCAATTTACGCGCGTGTACTACAAAAAAAATCTAAAATTCCAATTCCCATGCAAAAGAA<br>AAAAGAGACATCGCGGCGGTTCGAGGGAAAGAAAAGCAAAACAAGTTGGGGACACTTCGGAAGAATGTTGGACTGTTTTAATTAATT<br>TACTTTATATACATTTAAATTATTTTTAGTCTACCAACTCAGTACTTAATCTACTCATCTCTAACAATTTTTAATCATTTTTATAATATAT<br>TACGTAGTGTTGTTCCATTTAGTCCAACCTCTCTTTTATGTTTAATTCGTATTTTGAAAGAAGGGGAACCTTATATATCTTGTTAAGATT<br>CATCGTGAAGTATGTGAAATTGTGATCAATCATTTTCGTTGATATTTCTGTTCTAGCTTGGTTTCATTTGTTTTCAAGTTTGTTGGCTGA<br>CGGTTAACTTCCTGATATGATTTACGTTTTTATCTTTTTATTTTTCATTATGCGTCAGTTATTATGTTTGACTTTATATTTCACATTTTGT<br>TTATCCTTTTTCAGTAAATCTACACATTTCGAAATCTACAAAATCATCCCGATATCACTAATTTATTTAGTGATATTAATCAAGATAAGT<br>GTTTAATTACTTAATTAATTAAAGCTGAGGTATAAACACGTAATATAACACAGTTAAATGCATATATAACTTAGCTGCAGTGCCGGAT<br>CCAAAACATAAAATAACATCATTTATTTTACTTAATGAAAAATTTAGCTGATATAATCCATATATAAAGCACTAGCTAGTGTCAGCTCT<br>AATTTTCTTATACAGGAACGAAGATATAACAATTGCAGATTTTTCTTGAATACTAACTTCACCTAGACCCAACTCTTACGTACGTTTAT<br>CATTTATTTTGTATTTGATATTTTGCACGGTAATAAAATGTTTCTAAAGTTCATGACAAACATCAATATATTATCATTCTTAGTGGTATC<br>TTTTTATGTTTTTAAAATTAAATTAATAAATTGTTATGAGTTTTACCGACTTTTGATTTTTGAAAAACACAACCAGTCAAGCTTAGTACT<br>TTTTGAATATATACATATATATATATATATATATATAATTAGCACACACAGTACAGAAGTAGCAGTATTGACAGAAAGAGAGAATCAGAA<br>AGAAACTTATGAGTTAAGCAATTGAACACACTCACTCTCTCCTCCACCTTCCCAAAACCCTAAGCAAATTTGTATATAATATATAAAAC<br>AAAAAGAGCCTTGCAATAACACTGTGGACACACTCAAATCTTATATTTTTTATTCATCTATAAATAATTAATATATTATTCAAACATAT<br>TCCTCTATATATATGTGGGTATGTATATATATGAATAAAGGTTCTTCCTTTACCTTTGCCTTCTCTCTCTATCTTTCTCTAAGCTCTCCTC<br>TCCTCTTCTTTTCTCTCATATAAGCCTTCACCAAAACAAAAAGCTTTAATTCCTTTTTTTGCTCAAAAACAAAACCATTAAAAAAAAAA<br>CTAAAGAAAGAAATATCATCCAAGAAGAAAGATCAAAG
CsDof44	Csa09g039660.1	GAGATGACTAAAAATGATAATTTAACTATACCGAATGACTTAGCTAGAGAAGCTGGAGTCTGCTCATGGGAAGCAAGTTCAATGCTAT<br>GTCCCTATGTGCATGTAGCTTACTAGCTTGTATTGGTAAAAGTTCGACTCACCTCGAAATCGCTAACGAAAGTTTAAGTTACACCTTTA<br>ATAAACGACTGGATCATCATTATTGTGGATCTTGATGTCGTACAATCAATAATGACATACACTAAAGAAAATTAAAATAACTAGATAT<br>ATAGAATGTTTCTCGCATTTACAATTCTTGATACAAATTATTCGATTAGGAAAAGTATAAAACACAGTCCAAAAAAATGCAATTTTTTA<br>TTTTATTTAATCCTAACTATACAATATACACAAAGAATCATTATAAAACCATAATATACAATATTCATGTTTTCTTTTAATATACATTTG<br>CCATCACAAATCAAGAGTTTGAACCGTGATGGTGGTTTGCTAAACGTTACATCCAACAGATGAATCCAACAATCACATTTTCAAAATT<br>ACAAAACGTAAACTAGTACATGCAAGAATTTTAAAATAATGGACGCAAAAGTCATGAACCACACATGTGCTACAGACACATAGATGTT<br>TCTGGACCTTGGCTCCTGATCAGTCCTCACAGAGAAAATGTGGTCCAAGAAATGATTAATCTCAACTACAATTTATTATTCTATTTTTC<br>TTTCACCCAACATTTTAATAGTAACAGTAAACTTAACTTCGAGGAAACAAAATTACAAAAGAAACTAAAAAAAAAAAAAAAAAGAGGG<br>AAGAAAATAGATAAGTGTACTACTTGTCACACTTAGGACCAGCTTGAGGGAGTGGTGGGATTCACGCGAGAGTATAAATTTTTGACTA<br>ACTTAGAAAATGGAGGTGGGTTCCGCGTGGTGATGACAACTCAAGAAGAATTGTGACACAAAAGAAGATGTCAAGGACACATGAAGA<br>TGGGCGCCTCAGAAATGGACGTCGTACAAACAACCAATTCTGATACATCATAGTACGGTCCTAGATCACACTTCTCATCATTTCTACA<br>GAAACAAATCTGACGCCAAATCAAACCACAAAAAAAAAACATATATAATATTTTGTAATTGCATATTTTTCTTGTCTCATTATTGTGTT<br>AATAATCCCAAAAACATATGAAATCTCAAAAATATTTTTCGATCCGAGAGTACTAACTGTGTTTGGTAGTTTCAAACAATATTTCCCAC<br>ATCATCACATGTAATGTACTCACATACAACTTTTTGTACATGATAGAACAATCTATATCAACATGTTTTGTAAATTTACGGACCACATA<br>GTTATTTGTAAAGTGAAGGATTGGCTCAATCACAATTTTTAATTATAACAATCAACGTGTGATGATAATGAAAATCTTCGCTCACAAG<br>ATCTCGATATCGCAATATATATATATATATATATATATATTTTTTTTTTTATTATTAATATGATACCATTGAAGAGCCGATGAACAAGCAA<br>GATTGAATTAGAGACAACTTGTCTTAATTCTACTATATATTTCCAATCTAAATGAAAAATTAAAAAAGGTCTAGTTGTAAATCAGAAGT<br>AGCTATAGACATGATAAATCTTTATTTTAAAAAACGGCTTCATGGGTTCACATCTCATGCTAAGAGTTGGATCACGTTATTCCCTCATA<br>CGGTAATTAACATTTATTAGTAACGTTAAAACATTGAAACAATATAATAATATTGAGGTAAGCCACCAAATTGTCTAGCTACTTCCACA<br>TTATTTNTAACATTTATTAGTAACGTTAAAAATTGAAACAATATAATAATATTTATTGAGGTAAGCCACCAAATTGTCTAGCTACTTCC<br>ACATTATTTTTCTATATATAGAGAGATAAGATGCTTGGCTTTTCCTTTATCTCCACTCTCTTTTTCTCCTTTATCCCTCTTCGCTTTATCT<br>CAGTCTCAATCTTCTCTACATTTCAAAGAAGCTTCTCAATTAGTTGAGAT
CsDof45	Csa09g047820.1	TTCTACTATTCGTACCATCACAATTAGGTTTGTGATACTAGTATATTATTTTAAATTATTACTCATAAATGGATAAACTTAACTCAATTA<br>TCTGAATATTGATCTTTGTATGATGTTGATCAAATTTTTCTTTTTCTTATATCAACTGCATCCATCCATTGGTTTAAAAAGAACAAAATA<br>TCAACAAATCACAAACTATAACAAGCTAACAAATGAGATTACCTTTATCTGGGATCTGATGAAGGGAGAGAGATAAGTAATGCACTAA<br>AATACAATACCAATTCTCCAACTCTATGTTTTTGAATTCTTTGATGTTATAACATTTGTACAAAAGATGTATGATTGTGAGGCTTTCAA<br>AATTAAAAACTTTAACATACTTTTGAGCTTTGTTTTGCCATGTTGAAATGCTTTTATTTTTATTTTCTATTAAATATAATGTCTCCCTAA<br>AAAGTTTTCTCCATTGTTGAAAAAATCTTGCATCTAAATATATGATTATCATGTGTAAATCAACTGAAGTAGAATATTCAGTTGTTATC<br>TATTAGAAAGCTTGTATTCAAAATATTCTTTTGTCGTTATGTTAAAAAAACCATATTTATGATTTTGTGTTATGGGTTTTCAAAAAAAA<br>TTAATTTATATAATAAGATTACTTAATTTTTTTTTAAAAAGGTTTATCTTGTATATTTGTAATCCCATATATATCAGAAACACTTTTGGAC<br>AAAAAAAATTATTGATACGAGTAAAGTTAGAAATTATTACTAGGGACTTATTATTATTATTATTATACAATAAATATATTTTTTTTAAA<br>AGAACCCGACAACCCGATCCGGTAGTAATGAGGGAAAAGGAAAATATCGGAGGCCACATAAACTTGTGGCTGAGAATACAAATGTGA<br>TGCTGACGTNNNNNNNNNNNNNNNNNNNNNNNNNNNNNNNNNNNNNNNNNNNNNNNNNNNNNNNNNNNNNNNNNNNNNNNN<br>NNNNNNNNNNNNNNNNNNNNNNNNNNNNNNNNNNNNNNNNNNNNNNNNNNNNNNNNNNNNNNNNNNNNNNNNNNNNNNNNNNNN<br>NNNNNNNNNNNNNNNNNNNNNNNNNNNNNNNNNNNNNNNNNNNNNNNNNNNNNNNNNNNNNNNNNNNNNNNNNNNNNNNNNNNN<br>NNNNNNNNNNNNNNNNNNNNNNNNNNNNNNNNNNNNNNNNNNNNNNNNNNNNNNNNNNNNNNNNNNNNNNNNNNNNNNNNNNNN<br>NNNNNNNNNNNNNNNNNNNNNNNNNNNNNNNNNNNNNNNNNNNNNNNNNNNNNNNNNNNNNNNNNNNNNNNNNNNNNNNNNNNN<br>NNNNNNNNNNNNNNNNNNNNNNNNNNNNNNNNNNNNNNNNNNNNNNNNNNNNNNNNNNNNNNNNNNNNNNNNNNNNNNNNNNNN<br>NNNNNNNNNNNNNNNNNNNNNNNNNNNNNNNNNNNNNNNNNNNNNNNNNNNNNNNNNNNNNNNNNNNNNNNNNNNNNNNNNNNN<br>NNNNNNNNNNNNNNNNNNNNNNNNNNNNNNNNNNNNNNNNNNNNNNNNNNNNNNNNNNNNNNNNNNNNNNNNNNNNNNNNNNNN<br>NNNNNNNNNNNNNNNNNNNNNNNNNNNNNNNNNNNNNNNNNNNNNNNNNNNNNNNNNNNNNNNNNNNNNNNNNNNNNNNNNNNN<br>NNNNNNNNNNNNNNNNNNNNNNNNNNNNNNNNNNNNNNNNNNNNNNNNNNNNNNNNNNNNNNNNNNNNNNNNNNNNNNNNNNNN<br>NNNNNNNNNNNNNNNNNNNNNNNNNNNNNNNNNNNNNNNNNNNNNNNNNNNNNNNNNNNNNNNNNNNNNNNNNNNNNNNNNNNN<br>NNNNNNNNNNNNNNNNNNNNNNNNNNNNNNNNNNNNNNNNNNNNNNNNNNNNNNNNNNNNNNNNNNNNNNNNNNNNNNNNNNNN<br>NNNNNNNNNNNNNNNNNNNNNNNNNNNNNNNNNNNNNNNNNNNNNNNNNNNNNNNNNNNNNNNNNNNNNNNNNNNNNNNTTTTT<br>TTTTTTTTGTTGAGGAAAAAAATAAAAAAA

Name	Gene ID	Promoter sequence
CsDof46	Csa09g053360.1	TTTTTATTTGGACGTTTAATACCTCGTCTTTTTTGGTTGGAAGAAAAATACATCATTTAATTAAAACGTGCGATTTAATACCTAAACTA<br>ATAAATTTTGTTAGTTTCATACCTACGATTTGTTGACAAAAATAAAATTCCGTTTTTACCCTTACTATATCAATTAATAATTAATTTTAA<br>ATGATATGACATTACTTTATAAGTTTATCTGAATTAAAATTATTTTATTTTGATTACAATTTTGTTTTAAATATTTAAATAAAATTCTGT<br>TTTTACCCTTACTATATCAATTAATAATTAATTTTAAATGATATGACATTAATTTATAAGTTTATTTGAATTAGAATTAATTTTATTTTA<br>AAAATTTTATTTTAAATATTTAAATAAACTTCCATTTTTACCCTTACTATATCAATTAATAATTAATTTTAAATGATATGACATTAATTT<br>ATAAGTTTATTTGAATTAAAATTAATTTTATTTTAATTAATTTATAAGTGTATTTGAATTAAAATTAATTTTATTTTATGTTAAATGATA<br>TGACCAAGTCTGTGAAAGCGAGAGAGGAGAATAAACATGAATTAGAGTTTGATGAAGATAATTGTATTTTTTTGTAAAACAAAATTGT<br>AATTAAAATAAATAATAATTCAAATAAACTTATAAATTAATGTCATATTATTTAAAATTAATTATTATTTGATATAGTAAGGGTAAAAA<br>CGGAATTATATTTTTGTCAAAAAAACGTAGGTATGAAACTGACAAAATTTATTAGTTTAGGTATTAAATCACATCTTTTAATTAAATGA<br>TGCATTTTTCTTCTAACAGAAAAAACGAGATATTAAACGTCCAAATAAAAAATAGATGAGGTATTTTTAAGCTTTTTTCCCTTTAGCTA<br>GTGGAGTATTTATATAAATTTATTATATATAAGCATGAAAAATTGAAAGAATCTCAAAAGATAAATGTAGAATTACTTGGAAATGACC<br>GTACACATTGATAACCTCAATAGTCGTTTAACCTGTGTTGCCTACTGCGACCTACTCTATCATTTTGTTTTTTTTGGTTTTTTATAATCA<br>ACGTGTCTTCGTTATTACACAGAAGAAGATAAATATTACTACTATGACAACTAGTAGTAATTTAAACTTTAAACATATATAAAAGCAA<br>ACGTGATGACCAAACTTTCAACATTATTAGTCACATAAGTACAATAATTATCTTTTTCTCCTTTGTCAAAAAACTGATTTATAAGGTTG<br>GTAAAGACAAGACTAAAATATACATCATGATGTTTTTTTGATTTTTTCAGATGATTTAGGCAGGTACCAAAATTATACTACTAGTTTAT<br>CGATCAACAAAACCACAACGGTAAATTATTACCTCGTTGAAGTTTTTCTTTTGGGTCGTTTTCTCATTTTTCCATATGCATCAAATATGT<br>GTGAGATATTGTGTCACCCACCCACACTATTAACATAACATAAGATAGTCAAATATTGCGACGATCATAAGAAAATATGTTTTCTTCAT<br>GTCACCACTCATTAGTAGTGTATGTTATAGATGTGATTTTATATTGTAATGTTTTAATTCATTGTTCAAAACTGAAAGCTTTGAAAACA<br>GAGCCGTCTACGTATGAAGAGAGCCGACTTCGTAGTAGTGTTCATTCATAGGGATGAAAAGAACAGAGCGTAACACTGTTTACAATAA<br>TCCATAGCCGACTTAACCAATTCCTATAAACAATAAAATAAAAAAATCTTCATAAAAAGACAAAATTTAAAGACAAGAGCGTGGCCTC<br>TAATTAACCCAACACCCTCTGTCAAAAAAACAAAATAAAGCCTGAAACTTTCTTCTCCATCCCCATTTCTATCTGCGCATGTCTCTCTC<br>TCTCTCTCATCTCCATAAAAACCAAACAAAGTTCCCAAAAAAAATCCCACTCTCTCGTACCCTAAAGTTTCAACCTTTACCTCTCTATA<br>ACAAACCCAAACAAAAAAGCTTCCATTTTTTTCTCCGGTT
CsDof47	Csa09g059550.1	TGTTTCTTAAATCTAGAAACTTTCTTGGTGGGAATATATCCGAAAATAGCTATGAAGCTGGAGTTGCGTTAGCCGTTGAAGCTCTAGG<br>ATTCTCTAATGATGACCAAAGTGGTGTATTAGTCAAGGAATATATAGAAAGAGCTACAAGAATTGGTCGAGCTCCGATTCTGAGGTCG<br>GCTGAGTTAGCTAATGAGCTTGCTAGTGTCTTGCCAGCAAGACTCGAGATTCAATGGTACAAAGATCGTTGCGACGCTTCAGAGGAGC<br>AGCTCGGTTACTACGATTTCTTTAAACGTTATTCGTTGAAAAGAGACTTTAAGGTGAACATGAGTCGCAAAAGACTAGCTATGTTTTG<br>GGACAGAGTGCTTGAAATGGTGGAGACGAACGAGTTGCCTTTTGATTTTCATCTAGGGAAGAAGTGGATTTACGCATCTCAATTTTAT<br>CAACTCTTAGCTGAGCCACTCGACATCGCAAATTTCTACAAAAATAGAGATATAAAGAGCGGTGGGCATTACTTGGATGAAGGGAATA<br>GACCAAAAAGGTATGTGGTGATTGATAAGTGGTGGAAAAGAGCTGGAGAGCCTGAGAAGTGTGTGAGAAGCAGATACACTAGCACTA<br>CGCAAGATACTTGCTTTTGGGCTAAGCTTGAGGAAGCAAAAGAGTGGTTGGATGAGGTGAGAAGAGAGAGTAGTGAGGCTCAGAGGC<br>AATCTTTGCTACGGGAAAAGATTGTTCCATTTGAGAGTTATGCCGACAAACTGAAGAGAAATAAGGAGGTTTCTTTGGATGTTTTGGC<br>GAAGAACTCGAGTTACATTGTCTGGGTGGAGAATCTAAGAGAGTTCAGGTCGAAAATGGGTTATGGAAATGGAATTGAGATGGTTGT<br>TGATGAAGATGACGCAATGGAGACTTAGTAGGATTAATAATGTATCTAATGTTTGATATGCTATAATAACAAACTTTATTAGTACTGTT<br>GTTCTTCATTTCGTGTAAGACTTTCTGATTTATGTTATATCTTCAAAATCATGTATTATCATTAATGTTCAATAAGTTTTTGCAAGAGTT<br>TCTATTAATGTAAAGTTACTGGATACTGATGTAAAACCAACATGTTGTATGATCGATAGTAAGCAAACTTTTATGATATTTTTTTATAG<br>AATAGATAGAAAAAAAAAACGTAAGACCTACATGGGATTCTTGGAGATCCTCATTGTTCACATGATCCAAGAAGCTCTTTTCCCCATT<br>CAGATTTTCCTTTCCAAGCTCCCTTCTTTTGTGGGTAATTAGTGAAGTTAATACAAGTCAAAGTATACAAATAGAGACAGCCCTTGTTC<br>TTCAAAACTTGTGCTTTCCTGTTTCACATCCTCGTTTCTTAGCTTCTTTCTATTCACAGATCGTTACGCATACCTTACCACACGACGATA<br>TATGATCTGTCTACTTACAAGTTCTGATCCTTAACGGCTTTGACTAAATCTTCATCATGTGAATTTCAAATATGTTTCATAGTACTCGTT<br>AGTATTCGTTCCTACCATATTTGATATAATATTTTACTTTATTAAAGTGATTTTACATTCTATTTAATAAACATATTTTAGAAGTCGACA<br>AAATAGAAGACACATAGTAATCGTGCAGTCTCCATTAACTAAGCCGTACAAATAGCTCAATGTACTCCAAATAACCCATCTCTTCAAA<br>GAGGCCATAGGCTTAGTTCGCTTCGAAACAATCTCCAAGACGATCTATTAATTAACCATTTTCTTATTCATATCTTATTATGCAAAAAC<br>ATATACTGGTATTTAAATATTTTATCAAAGATTCAAAACCCTTAACCCTGTCTCCCCCAAGAAGCAACAGTGAGTGCACCCTTACGAGG<br>CATTTGCTCCCCACACTTCCTCCACTCTTCAGCCTCCACGTGTTACACCCTCTCCACATGCACAAACTCACACACACACACATTCATGC<br>ATGCGTATATAACACATTCTCCTTGTGAGAGAGGGAGAGTGGAAGAGAGAGAT
CsDof48	Csa09g067350.1	CTACAGAATTTCAAAGCATGTTCTTTCTTTGTGTTTCAACTTCATCCAAATTTCCAAGTAATTCAAATGTATTGGAACTTCTTCTTATCG<br>TTTTGTCTCAGTTTGATCATTTTCATAGTTGAAACGATTTTTTTATAATGAAGATAACGAATAGTGAGAGACAGAGAGAGATGTTTGCT<br>GTTATATTAAAATACGTTGGTATAATATCATTAGATAATTAATTTTGATAAATATCATTTTTTAATAGGTTGATTATGAATTATGTAGTT<br>AGCGATGTTTGCTGTTATGTGTCGACATAGTCAGGTCCATTTAAAAAGAGGTTTGATCAAATATACATAATTTGGACCACAAATCTTTC<br>TTTTAGAAATCGCGCGCGGAGCGGACTGGGACACCTTCCTACAAACATGTCCGTCTTTACTAAATCTTACGTACCCCTCACATTTGTAA<br>ACATAAATCATCAAATACATATAGACTGGCCGGTGATCATAATTCTAAATATATTAATTTCAATCATTCAATGTTATCGGTAAGTTATA<br>TGAGGGTCACATCAAGAATCACGAAAGAAAAGAAAAAAAAAAAGAAACAACCGTGTTGAATCATCATGATTTGATTTGTTGGCTTAATT<br>ATACATTCTAGTTTTTATTTATCTTGTGTTGCAAATTATCCAAATCAGCACATTATAATTTCTTCTTTGATATCCAAGTTCAGAAAGAAA<br>AAAACACCGAACGATTTATTGGATCACAAAATATAATAGTATTATCATATATTTGAAGGAAACCATATACACAGAATCCTACGAGTTC<br>TTGGATCATTCTTAAGACTGACCGCTCGGAATCTATAAAATCTCGCCATGCAAAGGTAATTTATTTTTATAAGACAAAAAACACAATG<br>ACGAAAAATTATAAGAAAGATAACTAAAATAAATACTATGAAAAACGAAACTTGGGTCACACCCGAGGAAAACAAAGGTACACTCGA<br>TTGTGACAACTCCACCAAAGACCACCCCACTACCAATTCACCTTTATTTGTTTCTTTATTAATTTCCAAATCTTTTTGTTTTTTTATATAT<br>ATTAATCTAGCTAATTATTCGCCTCTCTCGTTGTTTTTTGACCTTTTAATTAAAAAAAATTGAAGGGAGGTGCCTAGGGTTTCTCTCTCT<br>GCATGGCCAGTACTCTTGCTCTTCACATTTCTTTTTGGGCACCACTGTCTGTAAACGTTTGCAAAGAAAGAAAAAAAAGCTTACATGCT<br>AAGAAAACTATATTTCCTTGTCGTTTCTCTCATAAAAGAATTATATTTAATTTTGTCGTTGCTAATGAAATACAATATATTTAGGAAGT<br>GGAATCGGATCGGACAAGTGAGTGAATTATCTACCAACTTAGATTTCAATCGTCTTTTTCTCATTGATTGACAAGTAACATACACAATA<br>TACTAACACACATATTATATCTTTATAAGAAATGTATATATATATATATATATATATTACTTTATTAAAAGAATACACACATATCACAT<br>ATAATATTATAATAGTTTCCTTCAACCAAATGTTATAGTGAAATTGATCAATTTCATAACATATATGAAACAAATTCATACTTGATATA<br>TATATACAGTGATACAACCAAGACACACACAATGACGTCGTAGATGATTGACTTGCGAAAATAAGCAAAACAGAAAAACTTCTTTAAA<br>TCGACACTTAATTCCAAAAAGGTTAACAATAAGTAAGAAGGTTTTTTGATGAAAACAAAAAGAAATAAAAGAGCCTAAGAGAATGAT<br>GAAAATTGAAAGAGAAAGAGAAACAAAATAAAAAAAAAAAGAGTAAAGAGAATTAAGAAACACAATAAATTAAACAAAGGAAACTT<br>CATTTCTTCTTCTATTTATCTCATTCAGCTCCTCTCTTCGCTCTTCTCTCTCTAGATCAATTCTTTCTTGTATGATGTGATTTTCCACCAT<br>ATCTGCGACCTCTTACCTAAAAAAAAAAAGAGAAACCATTTAAAG

Name	Gene ID	Promoter sequence
CsDof49	Csa09g099480.1	AAAGTCGCATATGGAGGAAAATAGAGGCATTAGAAATGTGTTTCTATAAATGATATCCAACATGTAATGATGATTTTACAAGGGAGAG<br>CCGTGGATAGTGGATACCAGCTAACTATAGTATATAATTTTGCGTGGGGGAATTTATTATATTGGCACACTCAAACAGCATTTGTGAA<br>AATTCTTGACGTAAAGAGAAAAAATGAAAAAATGTGTGTAGTCTAAATGTCATAATAATGATAATAATAAATAGGTGTATCCTAAAAT<br>CGTAGAACACTGACTAGGGGGTAGTAGATGCTGAGGTGGATCGCAATAGAAGAAAGAAGATGGTCGGCTATATCTCTCTCGATACGT<br>TTCTTACGGCCAGAAATGAGGCAAACAGGTGGGCCTAGCAAAGATGACTAATCTCCACCGTCCAAACTTAATACACACAAATATTCGA<br>TTTTTCTCTCCCACAGTCAGAGGTCAGGTCAGGTCGACTAACACACCACGATCTAATATTTTGCTTCGCTACTTCTAATACAGACTTAC<br>TTTGTTTGTTTTTGTTTTGGGTGCTATTCCCTGTGGAGTCCAAATCAGCTAGAGAGACACGTGTCGTTATCACCAAAAGATTCATTGGC<br>GCGTGTGTGTATATATGGGTCCCAGTCGTCTCTGCTGTGATTCCCCCAAATCTGACGTACAGGCTGAAGTTCGCCGCTGGTCTACTCAT<br>CCGTCAAACGCCGGTTACACAGTGGAACAACGGCTGAAAGATGGAAACAGTAGTGTCGAATCTGGACCACTAGGCTACATTATTAAA<br>ATAAAATAATAATAATAATAATAATAATAAACAAACAGGAGAGACACGTACTTGTGGGTTCCATTCCATGTCAGAAGAAGAGGCATTT<br>CTCTTGCTTGTCAAAGACAACTCAGGCAACTCTTGTTCCTGGTAATAATGATATTCTTCTAATTTCGGCAACTCTCTCATCCTCATCACA<br>TGTTAAAAGATGAGATTTGTGGTATAAATGTTTTTAATATATACAGTATGATTAACTTAATAAAAATCCGGGTCCAAAAACCTCTAGTT<br>ACTTATACGGTACATTAGTCCCTTACTCTGGTTAATTAATCTGGTTACGTTTGAAGAGTCGAGAGATACTATTGGATGGATTAGTGGCG<br>GCTGCTGGCTAGTGGAAGAAGAAGAGACATGACGTCACTAATACACACACTTGTTTTCTAGTATTTCTTATATATTATGGTCTCCTTCA<br>ACAATTTTTTTTCCAATAATATATAAAAACACGTTTCAAAATACTATATGATCAAACACAACAGGCTAACAAAATAGCAAGCGTTTATA<br>ATTTAAAGAAAGTAATAAAAGAGAGAAGAAATAAATGGTGAGATAATTTACTGAGGTGAGTAGTAGTAGTGGTAAAGAGAGAGAGA<br>GATTCGTGGGACGGCGGGGGAATAGTTTCTCGTTTCAGACTGTGGTGTGTGTGTGTGTGTGTCAGAGTCAGAGTCAGAGTCAGAGTCA<br>GAGTCAGACCAAACCTCTCTCTATCTCTCTCACATACGAAGAAGAATCCATCAAAGCACACACACACATCACACTATATACATGTATCT<br>CTATCTATTAAATATACATATATTCCTTCTTTAAAAGTGACAAATTATTTATATAATTAAAAATTCTCTTTTTTTCGTCTTCTTGCTTTTT<br>TCAAAAACCAGCAGCTTTAATTAAGTACCTTCCCACACACACTTCTCCTCTCTCTCTTTTTCTCTTCTCTCTCCCAAACCTTTTAGCTT<br>TCTTCTTTTCTTCTTCAGATCAGCTTCAAATTTCCATAGCCATGGACACTGCTAAATGGCCTCAGGTATATATATAGTATTTTTTAATTT<br>TTTTTTTTTCAACTTTTCTCCTTTCTCAAAAAAAAAAGTTGGAAGGCTGGTCTAAGTGAAAACGAGAATAATCGTGTGCTTTTTGTTTG<br>TTTCTTGAAAAAAAACAAAAATAAAAAAAAGGAGTTTGTTGTGAAGCCA
CsDof50	Csa10g003120.1	TCGACAAACCCGGTATGCACATCGGTGTGGTAGGATTAGGCGGTTTGGGTCATGTAGGAGTGAAATTTGCCAAGGCTATGGGTTGTAA<br>AGTTACGGTTATCAGTACTTCGGAGGGTAAGAGAGACGAGGCTATTAATCGGCTTGGTGCCGATACCTTCTTGGTGAGCCGTGACCCG<br>AAGCAGGTCAAGGATGCAATGGGGACTATGGATGGTATAATTGATACTGTATCTGCGACTCATCCGCTTCTTCCATTGCTTGGTTTGCT<br>GAAGCATAAGGGAAAACTTGTTATGGTTGGTGCACCGGTGAAGCCACTCGAGCTACCTGTCTTGCCTCTCATCTTTGGTAAGCTATAT<br>TTGTAGTAACATACTAATCCCTATATTAATTTACATGTGCTAAATTTGTAGTGTTTAGTCAATGAATCGCGAGAATGACATGGATTTTG<br>TGTGTGTGTGTGTGTGGTTATGTTCTTTTGTATATCTAGAGAGGAAGATGGTAGTTGGAAGTTTGATAGGAGGGATAAAAGAGACTCA<br>GGAGATGATGGATATGGCCGCGAAACACAACATCACGGCAGATATTGAACTTATCTCTGCGGATTATGTTAACACCGCCATGGAGCG<br>GCTTGAGAAGGCTGACGTTAGGTACCGATTTGTGATTGATGTTGCCAACACTTTGAAGCCTTCTCCTATTTTATAAGTCACTCTTAACA<br>TTAAAATTTCCCCAAAGCTGTTTTTTTCTTTCTTTTGTTTGGTTTGTCGTAGTATTTAAGTTTCGTGTTGGTTCCTGGGGATGTTGTATG<br>ACTGTGTGGTATGCTTTAATCAAAGTAATGAACTTTAATATTATAGTTTCTCTATATGATAAAATTTTAAGGGTAAGCCAAGTATGATC<br>AATTAGTATATATCTAAGATTTCTATAGATAACACTCGAGTGTATCGATGAGATCCTTTAGATGAAAGAATCAAAATCAATTAACAGT<br>GTTAAAATAATCAGTTTATCATTACTCATTAGGCGAGTTATCAACCTGAGAATAAAATTTTTAAGCTAATGTGTGACAAACTGACAATT<br>AGTTATCTTCACATTTGGTGGAAGAGCAATAGAAGTGAAAAGTCAAAGAGGATAATAAATGAATCGTACGTACGACTTGTGTTGTTGT<br>GTTGCTTCAAACAAGAGAGGGGAGACAGTGAAGTTCGAGTTTGTTCCCAATGAGAAAAGCACTCCACTCCTTGATATGGCTTCCCTTT<br>CTTTACCTCGTACCTTTTACCACTCATCTCTCCTTCACTCTCTAGCTCTTCCCTAGGTCGATCTTATCTTGTCTTGTCTGAATATTACATC<br>ATATAATAATATAAGAGCATATATATATATATAAAGGTTTAGAACTGGATGAATTAATTTAGATATTTAGAATTGGTGAATTTTTCACCTA<br>GAGGAACAGCTTAAATAATTCTTAACAAATACAGAACCCACTGCAACAAATCCTGCTTATGGATAGAGTGGTGCTCATTGTTAAACCC<br>TTGATATATAAATGTTAAAATATGACTTAAAACTCTAATCTATATACTGTATTAATTAATCGTATTTAACACTAAAAGAAACTGGCCAG<br>AGATATTTTCCTTGCCAGAAGATTCAATATTTGGCTATTTCACGATAACGGTCACGCTTTTTTCACCTAGCTATACGATTAATGCTAAC<br>GCCTGCGTATCCATGGCGATATGCAACAACTACAAGTTGAATAACACATACTTATATAACTAGCCAGCCAGATACCATTTTATATGAT<br>GTAAAAAAAGAAAAAGAAAAACATTTTATTTAGAATATACACATTCGGTTTAAGACATAAATTAAAATATACATACTATACAACAAGC<br>GTGCATGTACACATATATATATATATATATATTTTATATACGTCAATGCGTTCATATATAAACATATGAAGATTCATAGAAATCACAAGAA<br>CCAATCTCCCCTCTCTTGCCCCATATTACACCATCTCTCTTGCCCCATATC
CsDof51	Csa10g018980.1	TGATAATATACTTTATATCGTGAAAAAAAAAGTTATACTTTATGTACCTTTTCGACAACTGAATTTTTTTCTTTCATTTGATAATTATAT<br>ATTTCTAAAGATAATCTTCTAACAAGATAGTTTAAAAAGGCCACACCTCTATCACTTTGCAAAACGATTTTGTCTTTTAAAAGGTAAAT<br>TGCTGATTTTTGTATTCAATTCTGTGATAATGTATATGTAGCATATATTAGTTGTGTTGATGCGTTATACTTGTAAAGTTGTACGTATCA<br>ATATAATCAAACTAGCTAACAAAGTGTATCAATGTAATTATGTAATTATGTAAATATATAGTTAACTAAATATGTACACATGCATGATG<br>ATACACACATAACGTTAAATTATTTATTATGTATCATATCATTATAACCAATTGGTAATAACTATTACATAACTAACGAATTATCTTCA<br>CTTCCAATTAATGTGAAACATTCTAACAATTTTTTTTTTGATAAAAAGCTAGATATATAGACATATTTAATTTTACGTGTAACAATATC<br>AAAACACACATCATATTAATCTACTAAATATATTTTTTGGCTCAAAATTGTCGAAACACTTACACTATTCCCCATAACTTTCTAGAAAT<br>AATAAATAATTAAGAAATGTTCTTTAACCAAAAAAAAAAAACAATATATGGGTAAAGAAGCCACTTTCCGTGATTTTACTACGAAAAGA<br>GCAGTCGACTTTTGAAACAGAAGAAGCGACCAATCTACTTTAAAGTTCAGGCATTCAAATCAAATTAGGAAATTTTGATGTACTATAT<br>ATATATATAGTTTACAATTTAAAGAACACAAGTGAAATAGTTGTGGACGTTAAGACTTAAATATCCAATACCTAACTAATGACCAAAA<br>TTCTCAACCCGACTCTGATTTTTCAAATTTGAAAGTTCCCTTGAACTGAAAAACAACAAGTGATATATTAAACAATTAAGAAACTGATA<br>GATGATGTTTTATCAGATAACACATGTATATTGATAGTCAAAATATTTCCAAAAGCATAACCATAATTTGCTTGTCGAATAGGATTTAC<br>GTTTGTAACTTCTTTTAGGAGTTAACGCGTTGCTACTGTTTTTGTTTTTTTTTCTTCTTCTAATACGTACCCTTACGAGAATCTGATTAT<br>GTTCTGCACTTATGCTAGAATTTTTATTTTATTTTTTGTCAGCAATTTATTTTATTTTTATGTTGAGTCAAAAATAGTATTTAGAGACTG<br>AAAACGACAAATTCAGTTTAATTTTGATTAGCATCCAATTAGTATTTAATAAATATTTAAAATATACGATAGTATTTTTTTCTGAAAAA<br>TCATGTTATCTTACAAGTTCAGAGACAAAATTGTGACGATCTTGCAAAAGAATATTAATAACATTTTTTAAATCACAATCGCATAATTA<br>AAGAATCTATAATAATTATGTTATTGTAATCAATTTAATTAAGAACTTGATTTTTGGTAATGAATTAAAATGAAATGCAGCATCATCAC<br>TATTTGTTTTATTGAAAGAAAAAAACATTCAGGCTATATGTAATCGTATGTACTTCCACCTAATTAATCCTTCCATGTTCTAAATAATG<br>TGGAGAGTTCTTTGGCAAATTATAGATGCGTGAATATATAATATAAGCCCATACATACATGCTTATAATAAATATGGACGCGTACTAA<br>AGCATTTGTTTATTTTTCAGTACGACAAAGATTCCCACTCGGAATGTTTTATTAATCTTATATTATACAATTTGTTCTCTCTCTCTTTCT<br>CTCTCTCTGTCTAGTCTCTACCATAAAGCAAATGTGATCAGAGACAAGCATACAAAGAAGTTCCCCATTCAAAAAAAGATTTCTCAGC<br>CATCAAATTTTTTCTTTTCTCTTCATCCTACAAAACAAAAACAAAAAGATAGTTTTTTCTTCCAAATACAGAAGTCAATTAAGAAAAAG<br>AACTATCAGTTTTTGTTTTGGCTGATCTGAGGAAGTTCATCT

Name	Gene ID	Promoter sequence
CsDof52	Csa10g022390.1	AGTTCGTATTTATCTCACAACACATTCAAGTCTTGTACAACAACAAATGTCGACACGATCAAGTATTGTTGCCTGAATGTAAGAACATG<br>CATGCATTTTCATGTTTTCTTCAGGATAAACGTTATCTTTGTTGATAAAGATGGAGAGGAAATTCATGTTAAGGTCCCCATTGGAATGA<br>ACATCCTTGAAGCTGCTCATGAAAATGATATTGAACTCGAAGGCACAATCTTCTTCCATTTCTTCNTTTTTTTTTTTTTATTCGTCCGAT<br>GATGCTTTTTGCTTAAACCAAGATATATAGTAACTAATGTCTCAGATTGGTCCTTTCATTGGAAATTTACAGGTTGCTTTTTCTAAATTT<br>TCAGGGGCGTGTGAAGGTTCTCTAGCATGTTCGACGTGCCATGTCATTGTTATGGTAAAACCATCTCTCCCTTACTATCACACACATCT<br>GCATAGATGATTATTGAATTGTATTTTACCAATTAGTTGAAACTGTAATTGTAATAGGACACCGAATACTACAACAAACTAGAAGAAC<br>CAACAGATGAAGAGAATGATATGCTAGATCTTGCTTTTGGGTTAACAGAAACGTGAGCTCTCTCTCTCTCTCTCTTACCACCTTACGTT<br>TTAAATGGTTTCAATCGGGGTTTAATGCAAGCAAATATTGACAATGTTCCATTGAAAATGTTATATAGGTCGCGATTGGGATGTCAAG<br>TCATTGCAAAACCAGAGCTAGATGGAGTACGCTTAGCCATTCCTTCAGCCACCAGAAATTTTGCGGTTGATGGGTTTGTTCCAAAACC<br>TCACTAGTCACACTAAAGCCTCGGAGCTGAGGGGTTATTTTGAGTTTACACTGGTTTACTATCCGAAAAAAAATCTCATTACATAAAAT<br>TACCCTTAGGGAGTTATACAAATGTATTTCAGACGGTGAGATTTTAATTGCAGCCTTGTCTTATTGTTTCTTGAGTTTGATTTAGATTA<br>TTGACAAAGCTAAAGATATTGATGAACAGATCAGAAATTCGAAAGCGCTTTGATCATGTTGAGCATTTAAACCTCAACAGTTGATTAC<br>CCACACAGGAACGGTCTAAAGGGCCTGTAGTCTTAAATGTTGAGTTAGTTTGGGCCACTGTAGGACCGTTGAATTACCTTTAGAGCAA<br>GCAATTAAAACAAATAACTTTATATTTGCCTAAACTAGTGGCGTCAAGACAAAACTTTGTTGTTAGTTCCATCTCTTCAATTTAACAGA<br>CAATATACAACTTTAACTCTTTCTATTTTCACCAATTGTATTTAACTCAAAATATATAAAGTTTCTATTATTAACTTTTATAATAAATTT<br>GTAAATCTTTAAAACAAACTTTTACAATAATATTTTTTGATGCGCCAATAAGATCATGCGTCAACCGCACTCGCTCACACGTATTATAT<br>TTTCTCTATAAACAGATAATCATTAAATCTCTACTAAAATTAATTACTTAGATGCTTTATCATTTAGAAAATTAGGCAATTAAGAACTC<br>TTTCTACCATGTGTAAACTATTTTACATATTTCTCTATAAAAAAAATTATCTAACACATCATAAGATAGAGCTTTAGCACACGCGTCGC<br>TTACAATTACTCTTATCTCTTATAGTAAATGTATGCAATCTATGTTTGATTTGCCTTAAATTGTCATTACACTCTCAATAGATTTTGTAT<br>ATGTTTATATGACTGAATCATTTAATTATATATATCTTGATTCTCCATTTTTCATATACAAACAAGAGTTAGGAATTAATAAATCCTTAG<br>TTTTTGACTAAGCTTAGCCTAAGGTTTATGGTGTGTAGATAGATCTTCAGATTTTATTAATACACAATCTTTGACACGTTGCGCTCTCA<br>ATCTCACATCCTTAAACTCTCAACCCCTATCTCTTCTTATATAAGAAGGGACACCCTCCCAGCACCAAAGATCATAGCAACAACAAAAT<br>CTTATAGTCTAAACCCAAAAAATCAAGTGTTTCTTGAGAGAA
CsDof53	Csa10g022470.1	CGAAGGTGAGTTTGCTACGTCTAGGAGGCTTTAAAGACTTATTATCGTCCCCACAAATCACCACATGATATTTCGCTGTGTTTTATTCT<br>CACTCGTACAGTTCTGACAGTCACTTTTCAGAAGGTCACCCATCCTGAGACTACTCCAGCATAAGCACGCTTAATTGTAAAATTCTCTC<br>ATGACTCTTGACCGAAAAGATAAGTGCACTTAGGTGGCCAAATCAATTCTTTTAAACATCTGCGCAAGTCTCTGATATCGGGGTGTCA<br>TACAAACAATCATCAAACACTCCTTTGATCCTTGTTTTCCAAAGATTCCAACAAATGCAAGGGCCAAAAGGCCATGCGCTTATACCTG<br>AGGGTGGAAGACAGAGTTGGTTGTTTACCAACTTTAGACCCTCTCTTGCATTCTCTACCTGATTGAAGGATAGTTCCTGCTTAAAGAG<br>GGCATAACTCCAAACCTCTTGTGCAAAGGGATAGAGAAAAAGCATATGCATTCGTTTCCACCACACCACACCGGTTTCAGGCTACCTC<br>GACGTCAATATGTCTCTCTGCGAGTTGTTGCTTCGCATGCAAAGCACCATGTAACACTTTCTAGAGGAACAACTTAAATTTTGGTGCCG<br>CATAGATGGACCAAACTTATGCATTCCAAACCACCACCTCCAAACGTTGCCCCATCTCCACTGATACATGTTCCTTTTCCACATAATAA<br>CTTGATCTGGTAGTATAGCAACCAGACAGGTTCCCCAACCAGACTTGTTTATCCTAACTCCCAGCCGGCTCAGTTTGATGCTGAGGAT<br>ATTTTCTTTCTGAGAAGGGAGAAGTATCCCAACCTTTAATTCATCCCACTCACAAGTTCCTTGTTCAAAGAGATCCCTAACAAGCAGAT<br>TCTTCGCTTCCAAGGGGGCAGGACCATAGGGTTGAGTAGGCGATGTAGTCGCTATGCATTATTTTTCTATATAACCATTCTAGTCGTGT<br>AAATTGTATCCATTTTAAGTGCGGTAAATTTCTATGAAAATAATAATTCACTTCCGTGATGCGCCAATGACAAACAACACGAGCATGTT<br>GTGGTTAGGCACAATGTCTTCTAACTCGAAAAAAGAAAATCACTAGGTGGTTATCGAAGCTAATCAGCTTCTTTTATAGTTTTCTCTTC<br>TACACTATTGATATGCTTGTTATCTAGATTTTGGGTATTGTTTTCTACGTAATCTTTTCTATTGTTACCAATTGTACATAATTTATAAAA<br>CTTTTTATAAGAAATTTTGTATTGCACCAACAAGAGCACCAATAAGACCACACATAAACCATTTGTAAAAAATTTTGTATTGCACCAAT<br>GGTAGCACGCGTAAACTATACGCGCTCACACTAATTATCTTTTTTTTTTCTCAATAAATAACATTAATTTCATATTAAAGTTAATAAAT<br>GTCTTTTGTCTTTTCTATATTATAAAAAATTCATTGTTCAAACATCATATAAAGAAATTTCTTCCAAAAATTCATAATTACACACCGTGA<br>GATAAAGCGTGAGCACACGTGTTTTCACATACTCTCTCCTATCATAAAACCATGCATTGCATTTTTGGTTTGCCTTATATTGTCATTAA<br>ACACTCAATGACCCTAGATTTTATTCTGTATGTGGGATTTGACCAATTTAAGTATAGCTTCTTGATTCTTAAATTTTTCACATAGAGAC<br>AAGAGTTAAAATTAAGAAAAAGCTTTGCATCTTTGACTAAGCTTAGCCTAAGGTTTATGGTGTGTAGATAGATCTTCAGATTTTATTAA<br>TACAAAATCTGTGACACGTTTTGCTCATAATCTCACTCTCCTAAACCCCGAAACGTATCTCTTCTTAAATTTCACGCCCAAGAACCTAT<br>ATAAGAAGACACCTTCACACCTTCCACAACACAAAACAGAGCATAGCTAGCAACAACAAGGTGTCAAGGTCTTATAGTTTAAGACTTT<br>TTTTTTTCTTTTTCTTAAGAGTGAATCAAATAGTTCTTGAGAGAAA
CsDof54	Csa10g046910.1	AGGTCGGGGGAACAACAGTCGGAAAATGATAAAATATGATTTTCTTAAGAATCTTTTTTTATCAGTCGTGCCTCCAAGTGGGATGGAT<br>ATATTATTAAATGTGAAATCGAAAGGAAAAAAGAGTCTTTATTATATATTTGAGATTTGGGGTGGTCTGAAATAAAGGTGCCATATAT<br>ATACTTATCCACATAATTGCACTTTTTTGCAATTCGAGTTGGCGTTCAAATACTTATCCACATAGTTGCACTTTTCCAAAATCGACTTG<br>GCGTTGATTAGTAACCACGAGTCCACGACTTCTAACATCGACTTATTACAGCCATGTATCTCCGATATGTTAAAAGGATTTCATGACCA<br>GACAAAGGCAAAAAAAAAAAAAGTGAAACAAAAAAAAACTGAAGTTCCACTAAATACTTTGTCGCTTTAAAAAATATTCTCTCCTTTTTC<br>CAGTAAAGAAATTACTTTAAGGGTGTATTAACATTTAAACAAAATTTAGATGATTTGAGTTTAGATAAGTTATTTATATAATTTTGATT<br>GAATGTTGATAAGTTGTTCTGATACCTATTGAAAATCTTTTAAATTATAACTTTTCTGTTAAATAAAAATGCGTTATGAAAATAATACT<br>ATATATACAAAAATTTCCAATTTTTATAAAAACCAGTCTAAGCCACTAATAGAGAAAAATAATTACTTCAAAAATAGTAAAAATCCAG<br>ATTGGTTTTTTACTTATTTGATTTACCGTTTGCTTTGCTAATTGGAATGTAAATGAGAGGAACATTTAGGTCTAACATCTTAAAACTATT<br>TCAAAATATTATTCAATAACAATCCATTTGAATATAGTTTATAAAAGTCTATAGTTGTTATAACTAGTACTACTTAATTCTTTTCAAAAT<br>GAAGCACAAAATGGTCTTTAGTTAACTATTGGACCAAATATAAACTGAATTAAAATTTAACAAGATTTTTAAAACAGTTGATTTTGGA<br>GATGTCTTGTGGTTGGTGAATTGAAGTGTGATGGTAGTTTGTATTTGTATCTGTGGTCGTGGTGAGTTTCTACTTTCCTCCATGACCAT<br>GACGATTTGCTACTAATTTGTTTATCGGATTTAATTAATATTATCAAAAAAGATTTGTGAAAGTCTTTAGTATCTTTGTTCAAGTATAC<br>GGTTTAGGTTACATTTTCTCTATGACATTTGCTCTTTTATAATTTTGTATCTTTTATAGTTTTTGTTAAGCATTGGGCTATAATGTACCG<br>GTTCATTAATCAAATCCTTACATTCTTAGGAAACAGAACTCGATCATAGGTATGATGGTGTCTTGTGCATTAAGGACTTAACATTGGCC<br>ACTACACCAAGATCACTTCACGTATTTTTTATAATTTTAATTAGTTTTTATATACTTTATATTTATTTTTTTGGTTCGCCCGCTCATATAA<br>TTTGAATGTGTATTTCTTTTTAGTTTTCATAATAAAATTTAGTTAATAAAATAAAAAATTACAAAACTGTAAATGGAGAAAAGGTGACA<br>AAATAACAAAAAAAAAAGTACAAAACTGTAAAATGAAGAAAGAGTAAAAGAAAAAAAAAGTTGCTGACCAGCTTGAAAAGGAAAAC<br>AAAAAGAAAGCCAAAACGAGACGAAAAAGAGAAAACCTTTTAAATTAAAAAACAACACAAAAAAGTCCACAAAAACAAAAGTATAA<br>AACTTGTGGCTGAGAAGCCTCACGTGTAATGACGTGGCACTATTGACGAATCAAAAGGTTGCACAATTCGAAAGATCACACCAAAAA<br>AAACAAAAAAGTGTAATCTTTTTTCTTTTCTCTCTCTCTCGCAGACACAAAATAAACAAATCCGAGAAATACTCAGACAACCACCGAA<br>AACCACAAGACTGTCCTCTCTCTCTCTCTCTCTCTCTTTCTCTCTTTCCACAAATAAAAATTGTAACTTTTTTATTTTCTTCTCGAAAAGT<br>TTCCGCTTTTTTCTCGCTGGAGAAGAAGAAGAAGAAGTGAGAAAAAAAT

Name	Gene ID	Promoter sequence
CsDof55	Csa11g003480.1	CAAACCCGGTATGCACATCGGTGTGGTAGGATTAGGCGGTTTGGGTCATGTAGGAGTGAAATTTGCCAAGGCTATGGGTTGTAAAGTT ACGGTTATCAGCACTTCGGAGGGTAAGAGAGACGAGGCGATTAATCGGCTTGGTGCCGATACCTTCTTGGTGAGCCGTGACCCGAAG CAGGTAAAGGATGCAATGGGGACTATGGACGGTATAATTGATACTGTATCTGCGACTCATCCGCTTCTTCCATTGCTCGGTTTGCTGA AGCAAAAGGGAATACTTGTTATGGTTGGTGCACCGGTGAAGCCACTCGAGCTACCTGTCTTGCCTCTCATCTTTGGTAACAAACTAAT CCCTATATTAATTTTACATGCATGTGCTAAATTTGTAGTGTTTAGTCAATGAATCGCGAGAATGACATGGATTTTGTGTGTGTGTTGTG GTTATGTTATTTTTTATATATAGAGAGAGGAAGATGGTAGTTGGAAGTTTGATAGGAGGGATAAAAGAGACTCAGGAGATGATGGAT ATGGCCGCGAAACACAACATCACGGCGGATATTGAGCTTATCTCTGCGGATTATGTCAACACCGCCATGGAGCGGCTTGAGAAGGCC GACGTTAGGTACCGATTTGTGATTGATGTTGCCAACACTTTGAAGCCTTCTCCTAATTTATAAGTCACACTTAACATTAAAATTTCCCC AAAGCTGTTTTTTTATTTCCTTTGTTTGGTTTGTCGTAGTATTTAAGTTTCGTGTTGGTTCCTGGGGAGGTTGTATGACTGTGTGGTATG CTTTAATCAAATTAATGAACTTGAATATTATAGTTTCTCTATATGATAAAATTTTTAGGGTAACCCAAGTATGATCAATTAGTAAATAT CTAAGATTTCTATAGATAACACTCGAGTGTGTATCGATGAGATCCTTTAGATGAAAGAATCAAAATCAATTAACTGTGTTAAAATAAT CAGTTTATCATTACTCATTAGGCGAGTTCTATCAACCTGATAATAAAATTTTCAAGCTAATGTGTGACAAACTGACAATTAGTTATCTT CATATTTGATGGAAGAGCAATAGAAGTGAAAAGTCAAAGAGGATAATAAATGAATCATACGTACGACTTGTGTTGTTGTGTTGCTTCC AACAAGAGAGGGGAGACAGTGAAGTTCGACTTTGTTCCCAATGCGTAAAGCACTCCACTCCTTGATATGGCTTCCCTTTCTTTACCTCG TACCTTTTACCACTCATCTCTCCTTCACTCTCTAGCTCTTCCCTAGGTCGATCTTATCTTGTCTTGTCTGAATATTACATCATATAATAAT ATACGAGCATATATATATATATATATCAAGGTTTAGAACTGGATGAATTAATTTAGATATTTAGAATTGGTGAATTTTTCAACTAGAGG AACAGCTTAAATAATTCTTAACAAATACAGAACCCAGTCAACCCACTGCAACAAAACCTGCATATGAATTGAGTGGTGCTCACTGTTA AACCCTTATTAATATATAAAATATGACTAAAACTTTAATCTATATACTGTATATGTTAATTAACCGTATTTAATACTAAAAGAAACTGG CTTGGCCAGAGATACTTCCTTGCCAGAAGATTCAATATTTGGCTATTTCACGATGACGGTCACGCTTTTTTCACCTAGCTATACGATTA ATGCTAACGCCTACGTATCCATGGCGATATGCAACAACTACAAGTTGAATAACACATACTTATATAACTAGCCAGATACCAATTTGAT ATGATATAAAAAAAGAAGAAGAAGAAGAAGAAAAAAACATTTTATTTAGAATATGCACATTCGGTTTAATACCTAAATTAAAATATAC ATATGTATACAATAAGCGTGCAAGCATATATTTTATATACGTCAATGCGTTCATATATATACATATGAAGATTCATAGAAATCACAAG AACCAATCTCCCCTCTCTTGCCCCCATATTACACCATCCCTCTTGCCCCATATC
CsDof56	Csa11g020730.1	TTTCGGTTTAGGAAATTTATTAATGTTAATATAATTATGGAGATTGTAAACTTTTGTTATGGAAAATCTGTTGTATATCATTTAAATGT GAGAGATTCTAGCATCCATAAAAATAGTTTTGGAGATTTGATGGGTTAAATCTTTAATGGGTTAAAACTTTAATGAGTAGAGTTGTTT GGAAAAATCGCAATATATAGATGTTGTTAAGATTTTATGGCAATAAATGTAACAAATGTTAGTCAATTTAAGAAAGTTTAGTATTTGA GTTTCTCTGCAATGTTATTTATGGAATTGTTTTAAGAGTTAATGTTGTAAAAAAAAAAAAAAATTAGTAAAACTTAAAGGGCATAAAAC AAAATATACTTCAAAAATGTTAATATAAATGATGATACATACATAAAAGTTAAATTATTTATTATGTATCATATCATTATAATCAATTG GTAATAAGTACTACATAACTAACGAATTATCTTCACTTCCAATAAATGTGAAACATTCTAACAATTTTTTTTTGATAAAAAACCTAGAT ATATAGACATATTTTACGTGTAACAATATCAAAACACACATAATATTAATCTACTAAATATTTTTTTTTGGCTCAAAACTGTCGAAACA CTATTCCCCATAACTTTCTAGAAACACTAATTAAGAAATGTTCTTTAACCCAAAAAAAAAAAAACAGTATATGGGTAAAGAAGCCACTT TCCGTGATTTTACTACGAAAAGAGCAGTCGACTTTTGAAACAGAAGAAGCGACCAATCTACTTTAAAGTTCAGGCATTCAAATCAAAT TAGGAAATTTTGATGTACTATATATATAGTTTACAATTTAAAGAACACAAGTGAAATAGTTGTGGACGTTAAGACTTAAATATCCAAT ACCTAACTAATGACCAAAATTTTCAACCCGACTCTGATTTTTCAAATTTGAAAGTTCCCTTGAACTGAAAAACAACAAGTGATATATAT ATATATATATATATATATATATTAAACAATTAAGAAACTGATAGATGATGTTTTATCAGATAACACACATATATTGATAGTCAAAATAT TTCCAAAAGCATAACCATAATTTGCTTGTCGAATAGGATTTACGTTTGTAACTTCTTTTAGGAGTTAACGCGTTGCTACTGTTTTTGTTT TTTTTCTTCTTCTAATACCTACGAGAATCTGATTATGTTCTGCACTTATGCTAGATTTTTTTTTTATTTATTTTTATGTTGAGTCAAAAAT AGTATTTAGATATTGAAAACGACAAACTCAGTTTAATTTTGATTAACATCCAATTAGTATATAATAAATATTAAAAATATAAGATAGTA TTTTTTCTGAATAATAATGTTATCTTACAAGTTCAGAGACAAAATTGTGACGATTTTGGAAAAGAATAACATTTTTTAATTCACAATCG CATAATTAAAGAATCTATAATAATTAAGTTATTGTAATCAATTTAATTGAGAACTTGATTTTTGGTAATGAATTAAAATGAAATGCAGC ATCATCACTATTTGTTTTATTGAAAGAAAAAAACATTCAGGCTATATGTAATTGTATGTACTTCCACCTAATTAAGCCTTCCATGTTCT AAATAATGTGGAGAGTTCTTTGGCAAATTATAGATGAGTGAATATATAATATAAGCCCATACATACATGCTTATAATAAATATGGACG CGTACTAAAGCATTTGTTTATTTTTCAGTACGACAAAGATTCCCACTCGGAATGTTTTATTAATCTTATATTCTACAATTTGTTCTCTCC CTCTCTCTCTCTGTCTAGTCTCTACCATAAAGCAAATGTGATCAGAGACAAGCATACAAAGAAGTTCCCTATTCAAAAAAGATTTCTCA GCCATCAAATTTTTTCTTTTCTCTTCATCCTACAAAACAAAAACAAAAAGATAGTTTTTTTCTTCCAAATACAGAAGTCAATTAAGAAA AAGAAATATCAGTTTTTGTTTTGGCTGATCTGAGGAAGTTCATCT
CsDof57	Csa11g025410.1	NNNNNNNNNNNNNNNNNNNNNNNNNNNNNNNNNNNNNNNNNNNNNNNNNNNNNNNNNNNNNNNNNNNNNNNNNNNNNNNNNNNNNNNNNNNNNNN NNNNNNNNNNNNNNNNNNNNNNNNNNNNNNNNNNNNNNNNNNNNNNNNNNNNNNNNNNNNNNNNNNNNNNNNNNNNNNNNNNNNNNNNNNNNNNN NNNNNNNNNNNNNNNNNNNNNNNNNNNNNNNNNNNNNNNNNNNNNNNNNNNNNNNNNNNNNNNNNNNNNNNNNNNNNNNNNNNNNNNNNNNNNNN NNNNNNNNNNNNNNNNNNNNNNNNNNNNNNNNNNNNNNNNNNNNNNNNNNNNNNNNNNNNNNNNNNNNNNNNNNNNNNNNNNNNNNNNNNNNNNN NNNNNNNNNNNNNNNNNGGTTGCTTTTTCTAAATTTTCAGGGGCGTGTGAAGGTTCTCTAGCATGTTCGACGTGCCATGTCATTGTTA TGGTAAAACCATCTCTCCCTTACTATCACACATCTACATAGATGATTATGAATTGTATTTTACCAATTAGTTGAAACTGTAATTGTGAT AGGACACCGAATACTACAACAAACTCGAAGAACCAACAGATGAAGAGAATGATATGCTAGATCTTGCTTTTGGGTTAACAGAAACGT GAGTTCTCTCTCTCTCTCTTACGTTTTGGATGGTTTCAATCGGGGTTCAATGCAAGCAAATATTGACAAATGTTCCATTGAAAATGT TTTATAGGTCGCGATTGGGATGTCAAGTCATTGCAAAACCAGAGCTAGATGGAGTACGCTTAGCCATTCCTTCAGCTACCAGAAATTT TGCTGTTGATGGGTTTGTTCCAAAACCTCACTAGTCACACTAAAGCCTCGGAGCTGAGGGGTTATTTTGAGTTTACACTGGTTTACTAT CCGGAAAAAAAAAACTCATTACATAAAATTACCCTTAGGGAGTTATACAAATGTATTTCAGACGGTGAGATTTCAATTGCAGCATTGT CTTATTGTTTCTTGAGTTTGATTTAGATTTTTGACAAAGCTAAAGATATAGATGAACAGATCAATATACATATGATCAGAAATTCGAAA GCGCTTTGATCATGTTGAGCATTTAACCTCAACAGTTGCTTACCCACAGCAGGAACGGTCTAAAGGGCCTGTAGTCTTAAACGTTGAG TTAGTTGGGCCACTGTCGGACCGTTGAATTACCTTTAGAGCAAGCAATTAAAACAAGTAACTTAATATTTGCCTAAGCTAGTGGCGTT AAGACAAAACTTTGTTAGTAGTTCCATCTCTTCAATTTAATAGACAATATACAACTTTAACTCTTTCTATTTTCACCAATTGTGTTTAAC TCAACATATATAAAGTTTCTATTAATAACTTTTATAATAAATTTGTAAATCTTTAGAACAAACTTTTACAATAATTTATTTTGATGTGCC GATAAGATCATGTGTGAACCACACTCGCTCACTCGCATTATATTTTTTCTATTAACAATTCACTATTAAACCTCTACTAAAGTTAATTAT TTAGATGCTTTATCATTTAGAAAATTAGGCAATTAAGAACTCTTTCTACCATGTGTAAACTATTTTACATATTTCTCTATAAGAAAAAT TTATCTAGATAGAATCGGAGGACACGCGGCGCTTACACTCTATCTCTTATAGTAAATGTATGCAATCTATGTTGGATTTGCCTTAAATT GTCATTACACTCTCAATAGATTTTGTATATGTTTATATGACTGAATCATTTAATTATAGATATCTTGATTTTCTATTTTTCATATACAAA CAAGAGTTAGAAATTAATAAATCCTTAGTTTTGACTAAGCTTAGGCTAAGGTTTATGGTGTGTAGTTAGATCTTCAGATTTTATTAATA CACAATCTTTGACACGTTGCGCTCTCAATCTCACTTTCTTAAACTCTCAACCCCTATCTCTTCTTATATAAGAAGGGACACCCTCCCAAC ACCAAAGATCATAGCAACAACAAAGTCTTATAGTCTAAACCCAAAAAATCAAGAGCTTCTTAAGAGAA

Name	Gene ID	Promoter sequence
CsDof58	Csa11g025430.1	CAAAGGAGATAGTTGGTGTTGGTAAGTTTAATGGTGACAATACTAGAGACATGAATGTTGTCGGGAAAAGGATAGAAATCGGCCATT<br>GAAACACCTATTAAGGCTACAATGGCTCTGATACCATGAAGGAACTTTAAAGAGATTGAGACTTTGATATTATAAAGTTCTTTCTCAA<br>TAGTCTATACAAGGTTGTTACAATGTATTATATAGAGTCTTAGGAACCATCTAGACTCCTCTAGGGTTTTCCCATCCTTATCCTACTAA<br>GAGAGCTGGTTTCACCTATTCATCAAATAAGGTTAGAAATGGTTGGAATCTGTTTCCGTGGTGTACTATTTCCTTCTTCTCCGTTCTAA<br>CGTCTCTTTCTGGTGCAGGGGGATTTGTCTTCTTATGTGGGAAGCTTGTTGGACCTTCTCTAGTTTGGGCCTTTCCTTGTGTTCAGCCC<br>AACTGTGGTTTATTACTTTCGATTAGTTAAAACTGTAATTGTGATAGGACACCGAATACTACAACAAACTCGAAGAACCGACAGATGA<br>AGAGAATGATCTTGCTTTTGGGTTAACAGAAACGTGAGTTCTCTCTCTCTCTCTCTCTCTCTCTCTCTCTCTCTCTCTCTCTCTCTC<br>TCTCTCACGTTTTAGATGGTTTCAATCGGGGTTCAATGCAAGCAAATATTGACAAATGTTCTATTGAAAATGTTTTATAGGTCGCGATT<br>GGGATGTCAAGTCATTGCAAACCAGAGCTAGATGGAGTACGCTTAGCCATTCCTTCAGCTACCAGAAATTTTGCTATTGATGGGTTTG<br>TTCCAAAACGTCACTAGTCACACTAAAGCCTCGGAGCTGAGGGGTTATTTTGAGTTTACACTGGTTTACTATCCGTAAAAAAAACTCAT<br>TACATAAAATTACCCTTAGGGAGTTATACAAATGTATTTCAGACGGTGAGATTTCAATTGCAGCATTGTCTTATTGTTTCTTGAGTTTG<br>ATTTAGATTTTTGACAAAGCTAAAGGTATAGATGAACAGATCAATATACATATGATCAGAAATTCGAAAGCGTTTTGATCATGTTGAG<br>CATTTAACCTCAATAGAGCCACAGCAGGAACGGTCTAAAGGGCCTGTAGTCTTAAACGTTGAGTTAGTTGGGCCACTGTAGGGCCGTT<br>GAATTACTTTTAGAGCAAGCAATTAAAACAAGTAACTTTATATTTGCCTAAACTTGTGGCGTTAAGACAAAACTTTGTTGTTAGTTCCA<br>TTTCCATCTCTTCAATTTAATAGACAATATACAACTTTAACTCTTTCTATTTTCACCAATTGTATTTAACTCAACATATATAACGTTTCT<br>ATTAATAACTTTTATAATAAATTTGTCAATCTTTAAAACAAACTTTTACAATAATATTTTTTGATGCGTCAATAAGATCATGCGTGAAT<br>CGCGCACTTGCTCACACATATTATATTTTTCTATAAACAAATCACTATTAAAGTTAATTACTTAGATACTTTGTCATTTAAAAAATTAG<br>GCAATTAAGAACTCTTTCTACCATGTGTAAACTATTTTACATATTTCTCTATAAAAAAAATTCTCTAGATAGAGCCTTTGCACACGCGG<br>CGCTTACAATTACTCTATCTCTTATAGTAAATGTATGCAATCTATGTTTGATTTGCCTTAAATTGTCATTACACTCTCAATAGATTTTGT<br>ATATGTTTATATGACTGAATCATTTAATTATAGATATCTTGATTCTCCATTTTTCATATACAAACAAGAGTTAGAAATTAATAAATCCTT<br>AGTTTTTGACTAAGCTTAGCCTAAGGTTTATGGTGTATAGATAGATCTTCAGATTTTATTAATACACAAGCTTTGACACGTTGCGCTCT<br>CAATCTCACTTTCTTAAACTCTCAACCCCTATCTCTTCTTATATAAGAAGGGACACCCTCCTAACACCAAAGATCATAGCAACAACAAA<br>GTCTTATAGGCTAAACCCAAAAAATCAAGTGCTTCTTGAGAGAA
CsDof59	Csa11g025480.1	GATAAAGCCTACGAAACAAAAGAGCAAGCAAAGGACACAGCTTACAACGCAAAAGAGACGGCTAAGGATTATGCGGAACGGACTAA<br>AGACAAGGTGAACGAAGGTGCGTCTAAGGCAGCGGATAAAGCTGAAGATACAAAAGAAAGAGCTAAGGATTATGCTGAGGATACAA<br>TGGACGACGCAAAAGCGAAAGCTCGAGATGCGAAAGAGAAGGTCAAAGAGTATGGAGAAGATACTAAGGAGAAGGCAGAAGGGATT<br>AAGGAGACTGTGAAGGGTAAAGCTGAGGAGCTTGGAGAGAAAACGAAGGAGACTGTGAAAGGAGCTTGGGAGAGTACCAAAAATGC<br>TGCTCAAACCGTGACTGAAGCTGTGGTTGGGCCGGAAGATGATGCGGAGAAGGCACGGGCTGATATCGATAAAAGCGTGGAAGATCA<br>TACCAAGTCGAAATGGAAGAAACAAGAGGAGAAAGATCGGAAAGAGGATGATTTCATAACATTTAACTAATGAGGAAGACATATCTT<br>TGTGTTGCTCTGTTTTCGGTTTATTCTCTGTTTTCAAACTAAACTTGGTCTCATGTATGTGACAATGTGAGATACATTAGTATATAACTA<br>AATAAGACAAGGGAATCTATACTTTGTGTTCTGTTTTCAGTTTATTCTCTGTTTTCAAACTAAATGTGGTCTCATGTATGTGACAATGT<br>CAATGTGAGATACAATAGTATATAACTAAATAGGACTAGGGAATCTATGGTTATTTATTTTTACGAGTACTAAGCTTTTATTTTGTTAT<br>GTACTTATGTCAATCTTCGTTCGGGCCATAGAAAATTTGCTGTTCGAAGGTCATGATGTTTGTTTAATTTAGTGTTCAAATCAACAAAG<br>TTACAGTTAATTTATCTTGGTGTTTTACAGACTCTTGAAATTATGTTATTTTGTTATAGTTGAAATTCTTTACATTGTTACTATACTAGC<br>TTGCTATGCATTATTTTCTATATAACCATTCTATTCCTCTAAATTGTATCCATTTTAAGTGCTGTAAATTTCTATGAAAATAAAAATTCA<br>CTTCCGTGATGCGCCAATGACAGACAACACGAGCATGCTGCGGTCAGGCACAGTGTCTTCTAACTCGAAGCTAATCAGCTTCTTTTAT<br>AGTTTTCTCTTCTACACTATTGATATGCTTGTTTAACTAGATTTTGGGTATTTTTTTTCTACCTAATCCTTTGATCCTTTCTATTGTTACC<br>AATTGTACATAATTTATAAATCTGTAAGAAATTTTGTACTTCACCAATAAGAGCAGCAATAAAACCACACATAAACCATTTGTAAGAA<br>ATTTTGTATTGCACCAATGAGAGGACGCGTAAACTATACGCGCCCACATTAATTATCTTCTTTTTTTTTCTCAACAAATAACATTAATTT<br>CATATTAAGGTTAATAACTGTCTTTTGTCTTTTTATATTATAAAAAGTCATTGTTCAAACATCATATAATGAAAATTCTGCCAAAATTTT<br>ATAATTATACACCGTGAGATAAAGCGTGAGCACACGTGTTTTCACACTCTCACTTATCATAAAACCATGCATTGCATGTTTGGTTTGCC<br>TTATATTGTCATTAAACACTCAATGACCCTAGATTCTCTTCTGTATGTGGGATTTGACCAATTTAAGTATAGCTTCTTGATTCTTAAACT<br>TTTTCATATAGAGACAAGAGTTAAAATTAAGAAAGTGCTTTGCATCTTTGACTAAGCTTAGCCTAAGGTTTATGGTGTGTAGATAGAT<br>CTTCAGATTTTATTAATACAAAATCTGTGACACGTTTTGCTCATAATCTCACTCTCCTAAACTCCAAAACGTATCTCTTCTTAAATTTCC<br>ACGCCCAAGAACCTATATAAGAAGACACCTTCACACCTTCCACAACACAAAACAGAGCATAGCTAGCAACAACAAGGTCTTATAATTT<br>AAGACTTTTTTTTTTCTTTTTCTTAAGAGAGAATCAAATAGTTCTTGAGAGAA
CsDof60	Csa11g055680.1	NNNNNNNNNNNNNNNNNNNNNNNNNNNNNNNNNNNNNNNNNNNNNNNNNNNNNNNNNNNNNNNNNNNNNNNNNNNNNNNNNNNNNNN<br>NNNNNNNNNNNNNNNNNNNNNNNNNNNNNNNNNNNNNNNNNNNNNNNNNNNTTTTTTTTTTTTTTTTTTTTGACAACGATAAAATATGTTTT<br>TCTTAAGAATCTTTTTTATCAGTCGTGCCTCCAAGTGGGATGATATATTATTAAATGTAAAATCGAAAGGAAAAAAAGAGACTTTATT<br>ATATATATTTGAGATTTGGGGTGGTCTGAAATAATAAAGGTGACATATATAATTATCCACATAATTGCACTTTTTTGCAATTCGAGTTG<br>GCGTTCAAATACTTATCCACATAATTGCACTTTTCCAAAATCGACTTGGCGTTGATTAGTAACCACGACTTCTAACATCGTCTTATTAC<br>AGCCACGTATCTCCGATATGTTAAAAATATTTCTTGACCAGACAAAGGCACAAAAAGAGTGTGAAACAAAAAAAAAACTGTAGTTCCA<br>ATACTTTGTCGCTTAAAAAATATTCTCTCCTTTTTTCCAGTAAAATATTTACTATAAAGGGTGTATTAACATTTAAACAAAATTTAGAT<br>GATTTGAGTTTAGATAACAAATTTATATAATTTTGATTGAATGTAGATAAGTTGTTCTGATACCTATTGAAAATCTTTTAAATTATAAC<br>TTTTCTGTTCAATAAAAATGCGTTATGAAAATAATATTATATAAACAACATTTTCCAATTCTTATAAAAACCAGTCTAAGCCACTAATA<br>GAGAAAAATAATTACTTCAAAAATAGTAAAAATCCAGATTGGTTTTTACTTATTTGATTTGCCGTTTGCTTTACTGATCGGAATGTAAA<br>TGAGAGGAACATTTAGGTCTAACATCTTCAAACTATTCAAAATATTATTCAATAACAATCCATGTGAATATATTTTATAAAAGTCCACA<br>GTTGTTATAACTAATACTACTTAATTCTTTTCAAAATTAAGCATAAAATAGTCTTTTAGTTAACTATTGGACCAAATATAAACTGAATT<br>AAAATTTAATAAGATTTTAAAAATACAAAACGGTTGATTTTCGAGGTGTTTTGTGGTCGGTGAAATGTGATGGTAGTTGTATTTGTATT<br>TATGGTCGCGGTGAGTTCCTTCTTTTCATCACGACGATTTGCTACTAGTTTATGAGATTCATCCGATTATATTAATGTTATCAAAAAAG<br>TTTTGTGAAAGTCTTTAGTATCTTTGTTCAAGTATACTATTTAGGTTACATGACATTTGTTTCTTTATAGATTTGTATCTTTCTATACTTT<br>ATATCTATTTTTTTGGTTCGCCGACTCATATAATTTTAATGTGTATCTCTTTTTAGTTTTCATAATAAAATTTAGTTAATAAAATAAAAA<br>AATTACAAAACTGTAAATGGAGAAAAGGTAAAATTCAGGTGACAAAATAACAAAAAAAAAAAAAAAACTGTAAAATGAAGAAAAAGTA<br>AAAGAAAAAAAGTGCTGACCAGCTTGAAAAGGGAAAAACAAAAAGAAAGCCAAAACGAGACGAAAAAGAGAGAAAAAAAAAAAAGA<br>GAAAACCTTTTAAATTGAAAAACACAAAAAAGTCAACAAAAACAAAAGTACAAAAACTTGTGGCTGAGAAGCCTCACGTGTAATGAC<br>GTGGCACTATTGGCGAATCAAAAAGGTTGCACAATTCGAAAGATCACACAAAAAAAAAAACAAAAAAGTGTAAATCTTTTTCTTTTCT<br>ATCTCTCTCTCGCAGAGACAAAATAAACAAATCCGAGAAATACTCAGACAATCACCGAATACCACAAGACTGCTCTCTCTCTCTCTCTC<br>TCTCTCTCTCTCTCTCTCTCTCTCTCTCTTTCCACAAATAAAAATTGTAACTTTTTTATTTTCTTCTCGAAAAGTTTCCGCTTTTTTCTCG<br>CTGGAGAAGAAGAAGAAGAAGAAGAAGAAGTGAGAAAAAAAAAAAAAGAATCATTGAT

Name	Gene ID	Promoter sequence
CsDof61	Csa11g094130.1	GAGAGTATGGCTAAATTACACATTCTCTCTTCCCACGACACAAGGTTCAAAAAATCGTATCATATATATACTCCATAAGATTCTATAAA TTATAAATCAATACTATAATATAGTATGATTTAACATATGTGTGGATATGGTCCTTAGACCAAATCTCGGTCTTCATAGAAGTGTCTTT CCCGAAAACCGATGTGTGATTTGTGTCTCTATGAACTTTAAAATCCCCATCGGATAATGCAAGCACATCATGATGATGAGCTATATAC AACACATTGTCCGGCCAATTCTTTGCTTTTACTTCATCATGTGTTCTATTTACATATCTTAATTATTGTGATATATAAAGCAATATGTAC CTAGAATTATTTTATTCACATTTTTAAATAATATATTTCTTTTAATATCCGTAAGAAGTATTTTAGTTTGTGTTACTGATGATAGTTGTT CCAGATTTAAAAACACACATATACAACCCTTTTTCAAAAAAGAAAACACACACACACATATTCAATATCAGTACGGACTTCTACAAAA CAAATCATTGACAATTTGCTCAACAAATTGCATAAGTAAAATAGAGGCAGAAATTTAGAAGGAAAAAACTGTAGCCCCGATATGTTTT GACAAAAATAGAAACATTTATGTCCTATCAGATAAAGAGAATCTATAACTATTTTTTTTGTTTTTAAGAGAATCTATTTAGTACGTAAT ATCCGATAGAAGAAACAAGGCTTAAGAAATCGTTAACCGACTATAGCTTTTATGCGTTCTACAATGCAATAAATTCCCATGCTAAGAA ACCAACAAATACTAATATAAATTTGAGTATCTGTAATTGTTTCTGCTTTAAACATGGAATTAGAATGAACGATCGAAATAGAAAATCA CATATATTTTGTTGTACGAACTACGAAGGTCACATTGAAACGGAAGGGGTCCATTTAGGAAGAAACAAGACATTGGCGATGAGTACG GGACCCGCGTGGTGACAACTCAACGGACCATTGGGACATAAAACACAGAAATGACAACGAAGCTGAGTGCGTGGGAATCCGACGTCG AACTCCAACCATCTCTCATCCATCATCGTACGGTTACTATTCATCTTGAATCATATAGATCCGATATCACATTATTTGATAACGACGAC TGCAACTCATTCATTTATATATAAGTTGACAGCTTAACCGATACATTATTGGTGGAGTGGCTAATCGAGCTTAGTGATTATTTCGGACT ATCGTTATAGACTTTTAGGCTACAAAAGTTTTGTTTTATACTTCCCAAGTTTTTTCTGTGGTTGTACTTACAATTAATTACCTCAATAAA ACTACAAATTTTGATTAGTAAAAGTTATTGTTTTTTTCGTAAGATATTATACAGTCAAAATATATAAATGTTAGATTATTTGGTAAATA TGTTTAAATATGATTAGTAGTATTCTGAGATATCAAAATCAACCAAAACATTAGTATTTGCGACCAATAAAATAACTCTTTTTTTTTTG AAGTAGTACTCTCAACAAGTCTTGAAGAGAGATCGTAACACATTGTCATCAGTTTATGTACTTTCCCAATACCTTTATCAAACTACTAA TTATGTAGAGATAAATTTTATAATATGCGTTCTGTATGTAGAATTTTTGTTCACATTATAAAAATGAATAAACGGCGGCTTTATGTCTG TTCCAAATCACGTTCCGAATTGGTTCACATTATTCTTCTTTGTCATACCACCATTTTGTTTAAAAATAATTAAATATTAAATAAAATAAT ACTATAAGGTCGAGGTCACAACAAGAATAGTGTCTCTTCTCTCTGCTCCCACATTCCCATGTATATATACAGATGCTTGGCTTTTCCTT TTGCTTCACTTTTAATTTCTACTCATATCCATCATTCTCTCTCTGATCTCAAGAAACAATCTCAATCTCCCAACACATAAACACTACTCA CACTCATTACTCTTCAAGCAGCTTCTCAGTTTCATCATCAGCTCTT
CsDof62	Csa11g094710.1	TATCATATGTACTATAAATATTTTGAAAGGCCTCTTAAACATTCATAAACCCATGGACTGTAGATCTAAATTTGGTTAGGATGTACAAA AATAGTTGTGATTACTTAATATGACAACCGGACAAATAATTGCGACTACAAGTACCACATTTGTACACATACGCTACATTCTCATTACC AAATAGTTTTATCATTCTTAAGAGATATACGGTGACGTGGTACAAGAGGAACCACTGGAACAGGCCACAGGGCTCTTTGAATCAGCAG ATAAAGCGGGGTGGCCGACAAGTCGACAATCCAATTTATGTGGATATCAAACTCTTACGTTTATTCACCCTCCACGTGCCCTCTTATCC ATCCATATCAACCGCTTAGATCATTCCTTCCCAATTCAACAAAAATCTTGCCCCACTATTATTACGATTAGGGTTAAATTTGTTATGCC AAATCCATCTTTTCAAAAAATTTCTTTGTGAAACTGACGTGTTAATTAAAAGGGTAGACTATGATGAATCGGTTATAACTCAAACGGA AGATATCTATTTTGGAGAAAAAAGATTAATATAGTAAGGTTTTGAACGAAGGAAAGCATTTTACGAAGGACTGTTGTACCACTATGAC AATTGTTAAAATCACTATTCAAGTTTGATGTAATATTCGTGCATCTGAAAGCGATATATACGCTAAAACTAATACTATAATTAATAATT ATTGAAAGATTGGCTTTGTAATTAAATTGTGGCAAGTGGCAACTATATTTCACTGTTCAAACCTTAAACTTACAACAAATGTTAGACGA TATATACTAGATGTTAGTTTCCTTGTAGTTGAAGCAAGGGATTGGACCTTTTGACATTGCCTCCATTATATTTCCAATACAAAAAGTTT CTGAAAACAATGCGCAATCAAAAGCTCTACGCGTAACTTCAAATTCCAAAAGAAGCCTCTCGCGAAAATACCTATTTATGTTTTTGTTT CAATTTCACACGCAAAGTAAAATTCCATACTTATATATTTAATCACAAAAACATAAGTACATAAGAAATTATTCCTTTTTCAAGCAACG TAGTTCAGTGAGATATTATATTATCTTGTGGACTATGTATGTGATATGTCTTAGACTCAGTACGAGAATAATATCTAAAGATTAAATAC AATTTATACAGCATATATATATATATATATATATACGTAAACACTAGTCAACTCAATTCAAACATTTTTCATATAGTACGGGATTAGAA ATTAGTAGGTTATTCAGACTTCTAATTCATTGTTTTGCAGAAGAAAATCATTTTAAAAAGTTTATTGGAAAATATTGATTTTGAATTCA TATTTTATTAATTACATGATCTCATGTATATTCGTTAGAAGATATTAACCTCTCCCAAGAAGTAATTTGCATTACTTGTTTTAAGAAAC AATGTACTAATAGTATGAATTTTCGATGGCGAAACAATAACATTTTTTTTTTAAAGTTTAGTTAAAAAAATCATCGCATTAATATTTTG AAGCTTTTGGGATTCAATATAATTCGATGTGAAACTAAAAGATTTAAAAAAACACGATTATTATTATTGTTTTTTTTATAAATATTTTT GTCAATCATTGGATATTTTGATGCGACTCTTTGGCATACAAATAAATATATACAACCGAAATATAACAAATCGTTTAAATGCGAAGAT ATTAATATTTAATACATACATAATTTGATGGGGCCAAATTTTTGGACTCGAAGCTAACTTCTGAATACAGCCACGCGAGGGGATTGTA AAATACAAAATATCGCATGGGCAGAACCGTCATTTGAAGATAAACTGCCTGTTCGGACATATGAAAGATATGCTTTGTCAGAGAGTTT CAGAAGAAGTCTTTCCCATGCTTACCACTTCACCTTCTCTCTCTTCTCTTCCTATTTCTCTTACCTTCTCCACTGTAATAACCTCGAAAC CCAAACTTGCTTAGATTGTATATTTTTCTTTATCATCTCTCTACGAA
CsDof63	Csa11g099290.1	CCATTGCCTGAAAACATGAAGACTCCTGGAGCTAACGGCAATGGACATGGCGGTGGTGGTCGTGGCGGAGGTGGTGGAAGAAGAGGC GGCCGTGGTGGTGGTAGAGGCGGAAGATTCAGAAGATAAATTTGCTTGATCTTTGATGCCGCCTTGTTTCAGAAGAAAATTTTGAAAT TTTCAGATTTAGTTAAAAACATGGAAGTTATGATCTTGTCGAATGCTATTGTATTTTGTTCGAAAAAAATTGTCAGTTATGACTTATTA TTATTGCTTAAGAAGAAGATGACGAACCTTGTCTCTATCTTCACAACATCAGAAGAAAACACAAGACGCTTCAGTAGATGAGTTCATA AACAAAAAAAACAAAAAAAAAACAAGACGCTTAAAGTCTGGTGTGTTTTCGAAACGTCTTAGACAACCGAAGAAGAACTCAAACTAA TCAGAAACCGAAACCGTGTATCAAACCAATCCCATAGTACTAGACTAAAGTTTGATGTCTATAAATTTGAAACCAAAAAATTCCAAAA ACCAAAACTCGAATCAAAGTCCAGAATTCCTGATTAAACACCTTCAGTTGATAGCGTAGTTAATAATAAGCATTGGGGGTGGTCTGGA TAATCGTCTCTCATTCAACCTTTCACAGCTACATCATTGATCCAGACTTGCATATTCTTCTACTCTGTTACTTGGAGAAAGTTATATTTC ATTGTCTTGCAATCAGTGCATATGATTGATTGGTTTTTGTTACTCGCTTCTTTTATAAACTTCAAATCATATACTTAGAAGTATATATGT TAATTTGGGAAAACAAGATAGAATTTTCAATTTGTTTGGCATCTAACAACAGAGACTGTTCNAAAAAAAAAAAAAAAAAAAACAGAGA GTAACGAGTGAAATAACTAATGTTTACGAGCCCTCCTAAGAGAAAGTCAAGGCTGAAAGCCTTAAACTAAAGCTGTCTAAATGAGTTA GGTCTTAAGCCTCTTACAATATAACTTTCGACGATGAACTACAAACAAATCTTGTATTACCTTCTTCTGCAGACTTCTTATCTTAACGT ACAACTACAACGGTTCACATAATTTAAATTCGTATATGGATGTTATATGGTTGTGATTTTTTAAAATAAATCTGTCAAAAAAACTGAAT CGAAAATGTTTAAATCCTTGTTGCATTTGTCTTCCTACTTAAACACAAAGATTCATATTACTAAATCTTCGGTAAAATATACCTTCTAA CAAAAGAAGGGTTCTTTAACCAAAAAACGATTAGAAAAAATAAAATTAAAGATGGTATATTTCTCTCTAACCCAAAAGTGTGGATCGT TTTCAAATGAGGAATATTAATTCTCCAACCAGCCAATAAAGAATATGCACGTAACAAGACAGCTCTCGTGTGAGCTGCACCAAAGCCA TCCCCTCGATGCTGACATTTACCTACCCAAAAGTTCGTAAACAGTTTACATTAGTTTATATTTGTTCCAATTCAAACGAACAAATAGAG TTGATCACTGTTTAAGATATGTTATAACCAATCTAATACCATATAATAAAGCGTGTTTATAGACCTTGACTAGTTGACTACATGCTCTT AATTAGGAACACTTCGTTCTTTCGTATATTAGGCCTTTCATCCAAGTACTTGTGAATTTTTTGTTCAACGGAAGTTTAGTGAAAGTCTT AATACAAATATATATATATAAAAAATGATATTCTTACAAAGATTGGAATAATTTTAACAAAAGTTTAAAGAAAATAACTAAAAGATTA TATGCTATATGGGTTTTGTGGAAGAAAAGTAAAATATCAGAGGCCACATAAACTTGTGGTTGAGAATGAAACGAAAATGCTGACATG GCATCTACTTCACTTTTTAAAACGTATCAAAATCCCAAATCTCACGATTTCCAAACCAAAAAAATTCCAATCTAAACCCATACCTCAAA AAAAAAAAAAAAAAGCGCTTCGAAGAGAAACCTACGAGAAAAAAAGAAGAAGAAATA

Name	Gene ID	Promoter sequence
CsDof64	Csa11g099790.1	ATTAATTCATCGTCAACCACATTTAACATTTTGCAAGATACAGTAATATTATATTTTCAATTTATTTGAAAAGAGAAAAACAAATTCAT<br>AACTTTAGACTCTCTTTTTATCCTACCGTCGGGTAAATTATCTGGTTTTAATTAACAAGCGTAATGCTTACTCAACCTAAAAAACAAAA<br>TCTGTTTATCACAATCATCAACAAGATTCATCCTCTACATCGAACCATTGGCAACCACCACCAACTAACTTCGTAAAGGTAAACTTTGA<br>TGGAAGCTTTCATTACCAAGATGGAAGTATTGGTGTTGGCTGGATATTACGTGATGATAAAGGAACGTATATATTATCCGGAAGTTCA<br>AAACTAAAGCAAGCAACTACTCCTTTGGAACCGAGGGACTAGCTCTACTACATGCCCTTCAATCGACTTGGTGTTGAGGTTACTGACA<br>AGTAATCTTAGAGGGAGACTGCAAAGCCCTGGATGATCTTTTACATCAACGAAGCATGAATATAATTATAGAGAACCTCCTAGTAGAT<br>ATCCGCAGTTGGGCGGATCACTTAACTAATATATCTTTCTCGCTAGTCCGAAGAGAGTGTAATCAGGCAGTTGATAGATTAGCAAAAC<br>AAGCTCATCTTTAACATGAAACATCTCGTGTAAACTACTACCCTCCATTGTATTTACGGATCGGAGGGAGCTTTTCCAAGATTATGTAA<br>ACAGTTGATGTTCTATTAAAAGACGTTAAAACAAAAAAAAAAAAAAAACAAAATCTGTTTACTAAAAAGCACTGTTACTTACAGCCCTC<br>AAACTTTTGCGTCTTCTTTATCATCGGATCAAACTCTTTAACCTTTATTTCGAAATCCAATAATAAGAAAATATCAAAAGTGGTTTTATT<br>AACTCTTTGAATAATTTACTAATGATTCAGGAAGTCAGAAAAACCAAAAACTTGAATCATTTTATTAACAACACATGTAGGAAAAGTG<br>ACATGTGGCAAGAGAATAGATACTTTGCTTGAGAGGAGCGAGCGAGTGGAACCCATCCCATTACTAGTCCAGTCCCAGTAGTGTCCCA<br>CCATAAGTTCTCAAACTCTCATCTCTTCTTCTGTCCTTCTTTATCGTAAGTCTCAAAACACAGACAAAACTTACGATAAAGATATTATTC<br>TCTCTCACTTTTGTCTCTGTTGTGTGACGATCAAGCCGCTATTTCTGAACCCATCCGACGGCTCACCCATTTCGTTTAGGTCCCATGGCT<br>TTAATCATAACCGTTGGATTTGATAAAGATGACTTGGTCAAGTTGACGTAGACCCACCCAAAGAATGTGGGTCCGAGGATTTTGGAAC<br>CACCGACGCTTTCTTTTTCCTCCTCCTCCGCCACATCCGTATCTACCGGTCCATTTATACGCCAAATCACTCGAAATCAAAGTTTTTAAC<br>TTTTATCATGATCACACCAAAGCGAGGCCGTATTAACTTTTCCAAAATCTAATTTTTGAATAAAAACAAATCTTTGTACCTCGAAACAG<br>AACATATTGATTAATTAATATCTACATATCAAGAAATTAAAGAAATACTGTAGTAAAAATACACAGACACTGAGAATTACTCACCGTA<br>CCCTATATTATTTCTAAAAATAGAAAATTCAAAGGAAAGCTCCATAGTTCTCGTTTTGTTGTCCATATATTAATATTAAACTTCTCAAT<br>AAATGTGAACCAAACTAAGAATTATATCCAGAATTTATATATATTACATTGTCGCTTTAAAACAAGATCACCTGAGTTATCCAAGATCT<br>AAATCAATAATTGAATTTCAGAGAGATACATTAAGCATTTTTCTTTGAAATAATATTTCATTAGTTTGAAATGATTTATAGTAATATTT<br>TTAAATAGAATTAAACTTGGAAAAAAAGAAAGTAAAAAACAAATAAGTGTGAACTAACAATAACTTAAGCAGTAGAAGTAGCTTTGA<br>GTGGAGACAGCAATCCCGAGGAGAAGAAGGGTCCTCTTCTCGCTTCCTT
CsDof65	Csa11g100300.1	TTGGATTTGATAAAGACGATTTGGTCAAGTTGACGTAGACCCACCCTAAGAATGTGGGTCCGAAGATTTTGGAACCACCGACGCTTTC<br>TTTTTCCTCCTTCTCCGCCACATCCGTATCTATACACTTATTAACTTATAGAGACATTTCAAAATGCTTAATGTATCAATAATTGAATTA<br>CCGAGAGATACATTAAGCATTTTTCTTCGAAATAACGTTTTATTAATTTGAAATGATTTATACTAGTAATATATAAAATACTACAGTAG<br>TATTAAACTTGGAAAAAAGGAAGAAAAAAAACATATAAGTGTGAACTAATAGTAACTTAAGTAGTAGAAGTAGCTTTGAGTGGAGAC<br>AGCAATCCCGAGGAGAAGAAGGGTCCTCTTGTCGCTTCCTTCTTAAACACATCCCAACCCCCAAACTCTTCCTTGCCTTTTCATCTCAT<br>TTCTCTTCTCCTCCCTCTCCCTCTCTCTCTTGATTTTGTTCAACAACTTCTCTTAAAATTCTCACTCTCCCCCAAACTAAACAAGCAAAC<br>AAAACACAAAATGGGTCTCACTTCTCTTCAAAATGTTTTTGTATATTGTCTCCTTTCCAAATGAAGATATATAGGACTTCAACCAATTC<br>AAGCTAAATTATTTTGTTAACTATAAACACAAAAATGACAAAATATTATGAAGATCTGTTTGTGTTTTATACGTACTTCATTAAAATTT<br>GAAATTTGGCGGCACTCTAAGGAATATTCCAGTTGGGGATGGATGCCGGAAAAACAAGAAACCAGGTTCCTCTAAATCCTCCTCCTCC<br>ACTTCTTCCGGCAAAAAGCCATCCAACATCGTTACCACTAATACCACTGATCTAATTAGCACATTCTCATCAAAATTACCAAAACTCAC<br>CTCTAGGATTTTCACATTTTGGTGGGATGATGGGGTCTTACTCAACTCCGGAGCACAGTAACGTTGGTTTCTTGGAGAGCAAGTATNN<br>NNNNNNNNNNNNNNNNNNNNNNNNNNGAAAAAAAACATATAAGTGTGAACTAATAGTAACTTAAGTAGTAGAAGTAGCTTTGAGTG<br>GAGACAGCAATCCCGAGGAGAAGAAGGGTCCTCTTGTCGCTTCCTTCTTAAACACATCCCAACCCCCAAACTCTTCCTTGCCTTTTCAT<br>CTCATTTCTCTTCTCCTCCCTCTCCCTCTCTCTCTTGATTTTGTTCAACAACTTCTCTTAAAATTCTCACTCTCCCCCAAACTAAACAAGC<br>AAACAAAACACAAAATGGGTCTCACTTCTCTTCAAAATGTTTTTGTATATTGTCTCCTTTCCAAATGAAGATATATAGGACTTCAACCA<br>ATTCAAGCTAAATTATTTTGTTAACTATAAACACAAAAATGACAAAATATTATGAAGATCTGTTTGTGTTTTATACGTACTTCATTAAA<br>ATTTGAAATTTGGCGGCACTCTAAGGAATATTCCAGTTGGGGATGGATGCCGGAAAAACAAGAAACCAGGTTCCTCTAAATCCTCCTC<br>CTCCACTTCTTCCGGCAAAAAGCCATCCAACATCGTTACCACTAATACCACTGATCTAATTAGCACATTCTCATCAAAATTACCAAAAC<br>TCACCTCTAGGATTTTCACATTTTGGTGGGATGATGGGGTCTTACTCAACTCCGGAGCACAGTAACGTTGGTTTCTTGGAGAGCAAGT<br>ATGGGGGGTTTACTTTCGCAGAGCCCTAGACCTACTGATTTCCTGGACAGTAATTAAGTATGATCTTATGGGAGTGAATAATGACAAC<br>GACCTGGTCATGTTTGATCATGGAAGTAACGGAGATCATCATCATCACAATAATCATCACATCACATGGGTCTGAATCACGGGTTAGA<br>TCTTAATAACAACAATGGTGGATTTAATGGGATTTCTCCGGGAAGCAATGGAAATGGAGGTGGTCTCATGGATATCTCGGCATGCCAA<br>AGACTTATGCTATCTAGTTATGACCATCTAACATTATAATCGTCAAGAAGA
CsDof66	Csa11g102460.1	GTTTTTTTTTTTTTTTCCTTTTTTTCTTTTGTGAAATAAAAACTGGCAAATTACGGCTGGAATAAATGGGAACAGCACGTAAATCAGGT<br>GATATGCTTCTACGACGTTTTTGATAACGATATTGCATTTGACCAATCTTTGATGGAAAAACTTCGTCTTTTAACTTAAGTAATTTTCTT<br>GTCATGTCTCTTTCTCACTTTTAACTTATCACACATAAATATATAACCTAATATTTTTGAACGCCGTAATAAATTTACATAGTTATTGTA<br>TTGTTGGATTTTCAGCAGTAAATCTCAGTTGTTAAAATGATAAATTCGCTGGTTTATTTATATAGTAGTTTTATTTCATTATTTTAGCTA<br>GCATGCATCTAGATTTTGTAAATTTTTTTTTGTTGTTGTTGCATTGCATGCCTCAAGATTATTAAAGATTAACAAACAAAATCGGTATA<br>AGTTGTACATTTTGTGGATGATCGGTTTAGTCTCACGAGAGATCCATTCCTCAAGGGGCTCTCACATGTTTCCCATTTTGTCTTCTCCG<br>TTCTCTCGTGTTTCTTCACAGATGGAGACGTTTAATATATCAATCATCGAAACACAATCTCCTCTATTTTTCCTTTTTTTTTTTATATATA<br>TATACACACTTGTCTGTATTGGCCACATAACTAAAACCCAATTGAATCTGCCTTTGATGGCAGTTCACTTTGTACAAATGTCAATTACT<br>CAAGAGTATATATTAGCGATACAGAAACATGTCAGAGAGTTAATCTAACGTTACTTTCTTACTAAACCCTACTTCTTCTTCTTAGGCCC<br>TCTAGATTTCAAATCTCAAATTAATTAACTTCCTTAAAAACATAACTTAGAAAAACTTCCTCAAATGTTTTAAATTACTATATATATATT<br>GTGACGATTTGGACTCATATAAAATCCAAAACTTTCAGCTAGTATGCACCCTGAATCACGCCTACATAATACGTATTGATACTTATCCC<br>TTTACTCGATTCCTTGGCTAACGCAAATTTCTAGTTCTTATATATGTTTTGTTCCAATAGTTTTGGTTTCCTTAATAGAGTTGCATACGC<br>TTGACAGCTATATAAGTAACTGTATGTAAATGAAAGAAAATATTATTCGTGATTGTATATAATTACACAAAATATGTAATGTAATATAT<br>AGTTTTTACGTATAATATGCACACTTCACACATATAAAAAAACGTGTAATATGCACACTTCACACATATAAAAAACGTTACCGCAACA<br>ATAGAAACAAGACACATATAACAAATTCTATCCGATTTGTTCCGATCAATTGCACAGTTTTATATGTTCTTCTGTCTTTTCTGCCACTTC<br>ATGTCTCTCTCTCATCTTCTCTTTTTGTCTCTATTATCTATCACTCATGCCTCTCTCTCTCTATATATATTTATATAAGTACAATGTACAT<br>ATACAAACATTAACTACAAGGAGATTTTATGAAGAATTTAATCAAACAGTTTTGATAAAAGTGGCGGTTTACGATTATTCAAGTCTAC<br>AACTGTGTGAAATGAACGAGAACACCAAGACCAGAGTTACAAGTACAACAGAAATAATATTTGTATTCTCAATATGAAAACTATGATA<br>TACATGTTTTGACACATTAGATTTTATTGTCCGTAATGTTCATATCGAACACAATTATCACAGTTTTAGTTAATAGAACATTTTCAAGA<br>CAAAATATATACTATATAAAAATTATTTTGAATTAATTTAAATATATAAAAATGCCAAACAAAATCCCGACAGTATATATCACCCATAT<br>ACATCATATATGCTCACATATATATAATTAATAATTTATATATAAATAAGTATAGAAGCATACGAAACCCTTTTACCGACTGTTGTTGT<br>TGTTGTCTCTCTCCTCTCAATACTTTACTTATTACATTCAAAGCAAGAAATCGAAAAAAGGAAGGAACCCCCAAAGCTTTCTTCTTCCT<br>TCATTTTTGCTCTCTCCTCCCCCATCATCATCATC

Name	Gene ID	Promoter sequence
CsDof67	Csa11g103980.1	CATCTACATGGATACCTCCCATCTTCCAATAAAGGATGCAGGTTTTGACATTGCAAATCATACAATCATAGGTGGTTGGATCGTTAGA<br>GATCATCGAGGAAAGGAACTCGTTTGGGGTGCAAAGAAACTAACTGATGCAGAAAACTCACTGGAGGCTGAAGCAAAAGCTTTAATA<br>TTAGCTCTCGAACAAACATTGAAACAAGGATACGATGCAGTCATCTTTGAAGGCGATAATGAATCTTTGTGCAAGTTGATTAATGGCT<br>CTATCATGAATGCATCTCTATCAACGCTTATGCAGGACATAAAGTTTTGGGCATCAAAATTCAGTTTCGTCAGTTTTAAGTTTACACAA<br>CAATGTGGCTCATACTTTAGCAAAATTAGGATGTAGAGATCATGAGTACTATGTTGAATTTGTAATGGTTCGAGATTGGCTAGTTCTTC<br>CTCTTTGTAATGACATTTCCTAAATCAATATAATCAGATTTTGAGTAAAAAAAAAGAATGGGATAAAGAAACTAAGAAAGAGATGAGT<br>GTCAGAATTTGACCAGATTAGACCGGTCGGTACAGCAGAGTAAGGACGGCTCTGAACCAGTGTAGACACATCTTGGGCACATACTTCT<br>CACATGTCTCTCTCTCTCTCAAATGTTGGATGCTTTTTGGTGTGAAAGGGAGTGAAGATTTGCATGCTTCTAGCTCTCCGTATCCATTA<br>TTTTACCCGACAGCCTTCGCTTCTGTCTCTTCACAAAACGAATCTCTTCTGTCTCTTTGACCGTTTTAGCCTCCCACATTTACATTTACA<br>ACCCTCTTCTTTCGTTTGGATTTGTCTCTTTCTCTCTCTCTCTACCTTGAAGATACATTTGTCTTGTTTCCGCAACGCAAGATCATGTTTA<br>GTCAGAAGTCCTACGATTATTGTTACTCCTGTCTACTTGGTCTTGTTGTCTAAATTTTTTTACCTTTTTTAGCTCCTACCCAAGATAAAA<br>AATCATAGCCTAGAAAATAAAAGAAAACATGGATTTGTGATTTTATATGCGTAAGATAGGACCATAACACGTAACATTGATCATTTAA<br>GTTGGTCTAACACATACTGTGATTTGATTTTAGTTAGCGTTGTTTCTGAGGTCCTGAACGCATTGATTCTCGTATAGTAATTGATAGAG<br>CACATCTATTACACACACCATAAATCTCTTAGGTCCATATAGTGTACTAGTATTTTTTTTTTTTTTTTGGGGAAAAGATGACACAACTA<br>TTATAGTTAATAACCATCGTAAATAAGGAAATTACACTAGTTATAGGAGCCATAATAAACCCTTAACAGTATAATGGTTGAATTATTG<br>GGAACTGCTTTACATCGATAAGCTCAATAGTCTCTCCCACAATTTGCCTATTGTTGTTGCCTACTCTCGTCACTCTTTCGTTTCCCGTAG<br>ACACGTACTTTTGACAGACGTTATTTTTTCCATATTTTATTTTTATTTTAAACCAATGTCATCATCTCTTAACTGTTGTTTCAACTCATA<br>ATAACTCGGAAGTACTACCTAACAACAAAATCAAAACTTAATTTACTTTAATTGGGTTTCTAGTCACTCACGAAGCGGATTTCTGATAA<br>TCATGCAAATGATGTCTTTATTTAAATCGCTTATATATGCCAAACGAAAACGTGCGACAGATTATGGATTGAGGAGATTAGATGTTTT<br>GTTTGTTTTCCCATAATTTTCAAAACAGAAAATCTTGAAAACAGAGCCACCTACGTACAGTATAACGAAGCGAGAGAGAGAGAGAGA<br>GAGAGAGAGAGAGACAGAGCATATCTCTTTCAATGTTCACAATAAACCCAACACACTCTGTCAAAAAGCTTCACTCTCTTCTCCTTCCC<br>CATCTCTCTCTCTCTCTCTCTCTCTCTTGTCCATAAAAGGAACCTCTTCCTCTCAGAAAGTTCCAAAACCTCTTAACACTTCTAAGCTTC<br>AAAAACTCTCACCTTTTCAGCGAATTACCTACCTCTCTTCAAAAC
CsDof68	Csa12g003340.1	ATGGGCTCGACAAACCCGGTATGCACATCGGTGTGGTAGGATTAGGCGGTTTAGGTCATGTAGGAGTGAAATTTTCCAAGGCTATGG<br>GTTGTAAAGTAACAGTTATCAGTACTTCGGAGGGTAAGAGAGACGAGGCTATTAATCGGCTTGGTGCCGATACCTTCTTGGTGAGCCG<br>TGACCCGAAGCAGGTCAAGGATGCAATGGGTACTATGGACGGTATAATTGATACTGTATCTGCGACTCATCCGCTTCTTCCATTGCTC<br>GGTTTGCTGAAGCAAAAGGGAATACTTGTTATGGTTGGTGCACCGGTGAAGCCACTCGAGCTACCTGTCTTGCCTCTCATCTTTGGTA<br>AGCTATATATATTAACAAACTAATCCCTATAGATATATGCCATATGTGTGCTAAATTTGTAGTATTTTAAGTCAATGAATCCCGAGAAT<br>GACATGGATTTTGTGTGTTTGTGTGTGTGTGGTTATGTTATTTTGTGCATATAGAGAGGAAGATGATAGTTGGAAGTTTGATAGGAGG<br>GATAAAAGAGACTCAGGAGATGATGGATATGGCCGCGAAACACAACATCACGGCGGATATTGAGCTTATCTCTGCGGATTATGTCAA<br>CACCGCCATGGAGCGGCTTGAGAAGGCCGACGTTAGGTACCGATTCGTGATTGATGTTGCCAACACTTTGAAGCCTTCTCCTAATTTA<br>TAAGTCTTCTAACTCTTAACATTAAAATTTCCCCAAAGCTTTTTTTTCTTCTTCTTTTGTTTGGTTTGTCATAGTATTTAAGTTTCGTGTT<br>GGTTCCTGGGGAGGTTGTATGACTGTGTGGTATGCTTTAATCAAATTAATGAATTTGAATATTATAGTTTCTCTCTATATAATAATATT<br>TTAAGGGTAAGCCAAGTATAATCAATTAGTATATATCTAAGATTTCTATAGATAACACTCTAGTGTATCGATGAGATCCTTTAGATGAA<br>AGAATCAAAATCAATTAACAGTGTTAAAAAAATCAGTTAGGCGAGTTATCAATCTGAAAAATAAAAATTTCAAGCTAATGTGTGACAA<br>ACTGACAATTAGTTATCTTCATATTTGATGGAAGAGCAATAGAAGTGAAAAGTCAAAGAGGATAATAAATGAATCGTACGTACGAATT<br>GTGTTGTTGTGTTGCTTTCAACGAGAGAGGGAGACAGTGAAGTTCGAGTTTGTTCCCAATGCGAAAAGCACTCCACTCCTTGATATGG<br>CTTCCCTTTCTTTACCTCGTACCTTTTACCACTCATCTCTCCTTCACTCTCTAGCTCTTCCCTAGGTCGATCTTATCTTGTCTTGTCTGAA<br>TATTACATCATATAGTAATAATATAAGAGCAAAGTTTTAGAACTGTATGAATTAATTTAGATATTTAGAATTGGTGAATTTTTCACCTA<br>GAGAAACAGCTTAAATAATTCTTAACAAATATAGAACCCACTGCAACAAATCCTGCTTATGAAATGAGTGGTGCTCACTGCTCATTGT<br>TAAACCCTTAATATATAAATTATAAAATATGACTTAAAACTCTAATCTATACACTGTATTAATTAATCGTATTTAATACTAAAAGAAAC<br>TGGCTTGGCCAGAGATATTTTCCTTGCCAGAAGATTCAATATTTGGCTATTTCACGATGACGGTCACGCTTTTTTCACCTAGCTATACG<br>ATTAATGCTAACGCCTGCGTATCCATGGCGATATGCAACAACTACAAGTTGAATAACACATACTTATATAAACTAGCCAGCCAAATAC<br>CATTTTATATGATGTAAAAAAAAAGAAAAAGAAAAAAAAAACATTTTATTTAGAATATACACATTCGGTTTAAGACATAAATTAAAAT<br>ATAATATACAATACTATATACAACAAGCGTGCATGTACACACATATATATATATTTTATATACGTAAATGCGTTCATATATATACATAT<br>GAAGATTCATAGAAATCACAAGAACCAATCTCCCCCCTCTCTTGCCCCATATC
CsDof69	Csa12g030340.1	TCTGTGATGATGTATATGTAGCATATACTAGTTGAGTAGTTGTGTATTGATGCGTTATACTTGTAAAGTTGTATGTATCAATATAATCA<br>AACTAGCTAACAGAGTATATCAATGTAAATATGTAATCAAATACTAGTTAACTAGATATGTACACATGATGATACATACACAAAGTTA<br>AATTATTTATTATGTATCATATCATTATAATCAATTGGTAATAAGTAATAACTACTACATAACTAACGATTTATCTTCACTTCCAATTAC<br>AATGTGAAACATTCTAACAATTTTTTTTTGATAAAAAGCTAGATATATAGACATATTTTACGTGTAACAATATCAAAACACACATAAT<br>ATTAATCTACTAAATACTCTCTGTTTTTCATTAGTTGTCATTATAGAGGATAATTTTGTGTTAAATTAGTTGTCGTTTTATATTTTCATT<br>ACAGTTTTTTTGATAAATTGTACAATTTTATCTTTATTTTTTTAGCTTATTAATTGATGATATCAAATAAAACAATAAATTAATTATGGG<br>TAAAACATAAAAATTAAACATCATTTTAATATGTGTGAAAAACCTTAAACGACCGTATTTTAAAACGGAGGGACTATATTCTTTTGCTC<br>AAAATTGTAAACACTAGTCCCCATAACTTTCTAGAAACAATAATTAAGAAATGTTCTTTAACCCAAAAAAAAAAAAAAACATATGGGT<br>AAAGAAGCCACTTTCCGTGATTTTACTACGAAAAGAGCAGTCGACTTTTGAAACAGAAGAAGCGACCAATCTACTTTAAAGTTCAGGC<br>ATTCAAATCAAATTAGGAAATTTTGATGTACTATATATATAGTTTACAATTTAAAGAACAGAAATGAAATAGTTGTGGACGTTAAGAC<br>TTAAATATCCAATACCTAATTAATGACCAAAATTTTCAACCCGACTCTGATTTTTCAAATTTGAAAGTTCCCTTGAACTGGAAAACAAC<br>AAGTGATATATATATTAAAAAATTAAGAAACTGATAGATGATGTTTTATCAGATAACACATGTATATTGATAGTCAAAATATTTCCAA<br>AAGCATAACCATAATTTGCTTGTCGAATAGGATTTACGTTTGTAACTTCTTTTAGGAGTTAACGCGTTGCTACTGTTTTTGTTTTTTTCT<br>TCTAATACCCTTACGAGAATCTGATTATGTTCTGCACTTATGCTAGAATTTATATTATGTTTATGTTGAGTCAAAAAATAGTAACTTAG<br>ATTGAAAACGACAATTCAATTTAATTTTGATTAACATCCAATTAGTATATAATAAATATTAAAAATATAAGATAGTCTTTTTTCTAACA<br>AAAAAAATCATGTTAGCTTACAAGTTCAGCGACAAAATTGTGACGATTTTGCAAAAGAAAAACATTTTTTTTAATCACAATCGCATGA<br>TTAATGAATACCGATAATAAGTTATTTTAATCAATTTAATTAAGAACTTAATTTTTTGGTAATGAATTAAAACGAAATGCAGCATCACC<br>ACTATTTGTTTTATTGAAAGAAAAAAAAAACATTCAAGCTATATGTAATCGTATGTACTTCCACCTAATTAATCCTTCCATGTTCTAAA<br>TAATGTGGAGAGTTCTTTGGCAAAATATAGATCAGTGAATATATTATATAAGCCCATACATACATGCTTATAATAAATATGGACGCGT<br>ACTAAAGCATTTGTTTATTTTTCAGTACGACAAAGATTCCCACTCGGAATGTTTTATTAATCTTATATTATACAATTTGTTCTCTCTCTT<br>TCTCTCTCTCTGTCTAGTCTCTACCATAAAGCAAATGTGATCAGAGACAAGCATACAAAGAAGTTCCCCATTCAAAAAAGATTTCTCAG<br>CCATCAAATTTTTTCTTTTCTCTTCATCCTACAAAACAAAAACAAAAAGATAGTTTTTTTCTTCCAAATACAGAAGTCAATTAAGAAAA<br>AGAAATATCAGTTTTTGTTTTGGCTGATCTGAGGAAGTTCATCT

Name	Gene ID	Promoter sequence
CsDof70	Csa12g037400.1	NNNNNNNNNNNNNNNNNNNNNNNNNNNNNNNNNNNNNNNNNNNNNNNNNNNNNNNNNNNNNNNNNNNNNNNNNNNNNNNNNNN<br>NNNNNNNNNNNNNNNNNNNNNNNNNNNNNNNNNNNNNNNNNNNNNNNNNNNNNNNNNNNNNNNNNNNNNNNNNNNNNNNNNNN<br>NNNNNNNNNNNNNNNNNNNNNNNNNNNNNNNNNNNNNNNNNNNNNNNNNNNNNNNNNNNNNNNNNNNNNNNNNNNNNNNNNNN<br>NNNNNNNNNNNNNNNNNNNNNNNNNNNNNNNNNNNNNNNNNNNNNNNNNNNNNNNNNNNNNNNNNNNNNNNNNNNNNNNNNNN<br>NNNNNNNNNNNNNNNNNNNNNNNNNNNNNNNNNNNNNNNNNNNNNNNNNNNNNNNNNNNNNNNNNNNNNNNNNNNNNNNNNNN<br>NNNNNNNNNNNNNNNNNNNNNNNNNNNNNNNNNNNNNNNNNNNNNNNNNNNNNNNNNNNNNNNNNNNNNNNNNNNNNNNNNNN<br>NNNNNNNNNNATTGTGATAGGAAACCGAATACTACAACAAACTCGAAGAACCAACAGATGAAGAGAATGATATGCTAGATCTTGCTT<br>TTGGGTTAACAGAAACGTGAGTTATCTCTCTCTCTTACGTTTTAGATGGTTTCAATCGGGGTTCAATACAAGCAAATATTGACAAATGT<br>TCCATTGAAAATGTTCTATAGGTCGCGATTGGGATGTCAAGTCATTGCCAAACCAGAGCTAGATGGAGTACGCTTAGCCATTCCTTCA<br>GCTACCAGAAATTTTGCGGTTGATGGGTTTGTTCCAAAACCTCACTAGTCACACTAAAGCCTCGGAGCTGAGGGGTTATTTTGAGTTT<br>ACACTGGTTTACTATCCGAAAATAAACTCATTACATATAATTGCCCTTAGGGAGTTATACAAATGTATTTCAGACGGTGAGCTTTCAAT<br>TGCAGCATTGTCTTATTGTTTCTTGAGTTTGATTTAGTTTTTTTGACAAAGCTAAAGATATAGATGAACAGATCAATATACATATGATC<br>AGAAATTCGAAAGCGCTTTGATCATGTTGAGCATTTAATCTCAATATACCCACAGTAGGAAGCCAGGAACGGTCTAAAGGGCTTGTAG<br>TCTTAAACGTTGAGTTAGTTGGGCCACTGTAGGACCGTTGAATTACTTTTAGAGCAAGCAATTAAAACAAGTAACTTTATATTTGCCTA<br>AACTAGTGACGTTAAGACAAAACTTTGTTGTTAGTTCCATCTCTTCAATTTAATAGACAATATACAACTTTAACTCTTTCTATTTTCACC<br>AATTGTATTTAACTCAACATATATAAGTTTCTATTAATAACTTTTATAATAATTAATTTGTCAATCTTTAGAACAAACTTTTACAATAAT<br>ATTTTTTGATGCGCCAATAAGATCATGCGTGAATCGCGCACTCGCTCACACGTGTTATATTTTCTCTATAAACAAATCACTATTAAATA<br>TCTACTAAAGTTAATTACTTAGATGCATTGTCATTTAGAAAATTAGGCAATTAACAACTCTTTCTACCATGTGTAAACTATTTTACATA<br>TTTCTCTATAAAAAAAATTATCAAGATAGAGCCTGAGTACACGCGGCACTTACAATTACTCTATCTCTTATAGTAAATGTATGCAATCT<br>ATATTTGATTTGCCTTAAATTGTCATTACACTCTCAATAGATTTTGTATGTGTTTATAAGACTGAATCATTTAATTATAAATATCTTGAT<br>TCTCCATTTTTCATATACAAACAAGAGTTAGAAATTAATAAATCTTTAGTTTTTGACTAAACTTAGCCTAAGGTTTATGGTGTATAGAT<br>AGATCTTCAGATTTTATTAATACACAATCTTTGACACGTTGCGCTCTCAATCTCACTTTCTTAACTCTCAACCCTATCTCTTCTTATATA<br>AGAAGGGACATCCTCCCAACACCAAATATCATAGCAACAACAAAGTCTTATAGTCTAAACCCAAAAAATCAAGTGCTTC
CsDof71	Csa12g037460.1	ATGCCACAAAGGCAAACTAGAAGTTTCCATGAAGCATTTTTCTCGATTAATCATATGTTTTCTGCATCATATAGCACAGCCTCTGAAAA<br>AGGTGGCGAAGAAACAGAAAAGTAAGTTCGTATATATCTCAGAACCACATTCAACTCTTGTACAACAACAAATGTAGACACCGTCATT<br>TATGCACAAGTATTGTTTGCCTGAATGTAATGATATACATGCATATTCATGTTCTTTTCAGGATAAACGTTATCTTTGTTGATAAAGAT<br>GGAGAGGAAATTCATGTTAAGGTCCCCATTGGAATGAACATCCTTGAAGCTGCTCATGAAAATGATATTGAACTCGAAGGCACAATCT<br>TCTTCCATTTCTTCTATTTCTTTTTTTTTTTTTTTTTTTTTTTAAATTTGTTCGATGATGCTTTTTTGCTTAAACCAAGATAGATAGTAACT<br>AATGTCTCAGATTGGTCCTTTCATTGGAAATTTACGGGTTGCTTTTTTCTAAATTTTCAGGGGCGTGTGAAGGTTCTCTAGCATGTTCG<br>ACGTGCCATGTCATTGTTATGGTAAAACCATCTCTCCCGTACAATCACTCATCTATATAGATGATTATGAATTGTGTTTTACCAATTAG<br>TTGAAACTGTAATTGTGATAGGAAACCGAATACTACAACAAACTCGAAGAACCAACAGATGAAGAGAATGATATGCTAGATCTTGCTT<br>TTGGGTTAACAGAAACGTGAACTCTCTCTCTTATATTGACAAATATTGACAAATGTTCCATTGAAAATGTTTTATAGGTCGCGATTGGG<br>ATGTCAAGTCATTGATGGAGTACGCTTAGCCATTCCTTCAGCTACCAGAAATTTTGTTGTTGATGGGTTTGTTCCAAACTGGTTTACGG<br>ATACTAAACTGGTTTACTATCCGTAAAAAAACTCATTACATAAAATTACCCTTAGGGAGTTATACAAATGTATTTCAGACGGTGAGATT<br>TCAATTGCAGCATTGTCTTATTGTTTCTTGAGTTTGATTTAGATTTTTGACAAAGCTAAAGGTACAGATGAACAGATCAATACACGTGA<br>TCAGAAATTCGAAAGCGCTTTGATCATGTTGACCATTTAACCTCAATTGGGCCACTGTAGTCAGTTGGGCCACTGTAGGACCGTTGAA<br>TTACTTTTAGAGCAAGCAATTAAAACAAGTAACTTTATACAACTAGTGACGTTAAGACAAAACTTTGTTGTTAGTTCCATCTCTTCAAT<br>TTAATACACAATATACAACTTTAACTCTTTCTATTTTCACCAATTGTATTTAACTCAACATATATAAGTTTCTATTAATAACTTTTATAA<br>TAAATTTGTCAATCTTTAGAACAAACTTTTACAATAATTTTTTTTGATGCACCAATAAGATCATGCGTGAATCGCGCACTCGCTCACAC<br>GTGTTATATTTTCTCTATAAACAAATCACTATTAAATCTCTACTAAAGGTAATTACTTAGATGCTTTGTCATTTAGAAAATTAGGCAAT<br>TAAGAACTCTTTCTACCATGTGTAAACTATTTTACATATTTCTCTGTAAAAAAATTATCTAGATAGAGCCAGAGTACACGCGGCGGCGA<br>TTACAATTACTCTATCTCTTATAGTAAATGTATGCAATCTATGTTTGATTTGCCTTAAATTGTCATTACACTCTCAATAGATTTTGTATA<br>TGTTTATATGACTGAATCATTTAATTATAGATATCTTGATTCTCCATTTTTCATACACAAACAAGAGTTAGACATTAATAAATCCTTAAT<br>TTTTGACTAAGCTTAGCCTAAGGTTTATGGTGTATAGATAGACCTTTAGATTTTATTAATACAAAATCTTTGACACGTTGCACTCTCAA<br>TCTCACTTTCTTAAACTCTCAACCTTTATCTCTTCTTATATAAGAAGGGACACCATTCCAACACCAAAAATCATAGCAAGAACAAAGTC<br>TTATAGTCTAAACCCAAAAAATCAAGTGCTTCTTGAGAGAA
CsDof72	Csa12g037480.1	TTTTTCTTTCTTAATATATTCTTTGTATTTCTTTCTTCTCATCTCTAGTAAATATATGGTATTAAGATCATTACAATTTAGTTATTATAAA<br>AAAATTGGTATAAAATCCAGTGAATATGATTCTTAGCTAATACACCAAATCCACGGCGAGTCAACAGATGATAATAGAAATTGATGAA<br>GAGAATCAGATGGGGATGTGGCTAAAGAAGATTCTTCTTTTTTAGTTATTAATTATTAGTAACCTTTAGTAAATAATTATTCGTAATAC<br>AATCTTTAGTTATTACTTATTAGGAACCTTTAGTATTTTGCTGATTTAACTCTAGTATTCGTTCCTTTTTTATTACAGTTGTGGTTGGTA<br>ACTTCATGGATTTAGGGATTTGGTGATTGTTGAATTGAAGTTTGATCTGAAAACACCTATCAGAGTATGATCTGATCCGATCATCCAAG<br>CTTATGTTCACAATATGGTTGGTTTGATCGTTTGGTATAGCCTAGTATTGGATCAATTTAGTGTACTAGGAGCTCATATTGCTTCTATC<br>TATTGTTTTGTACGTAGAATCAACCAGAGCGTGTTGTCCAACTGAGAATTGTTAATTCTTAGATGATGTAGATGATTATACTGCTACTG<br>ATTGTGAAAGGGACTTGGGTTAAAAGCACTAGCTAGGCAAGAGAAGCGAGCCAAGAAAGAATCATTGGCCACAGTACGTGCCAGTCA<br>TAAAGTGATTAGAACTTGAAACTGATGATAAGTCAGTGGTTTCTGAATTAGAAAGTATGATTACGGAACGCTAAAACAGTTTCGACTT<br>ATGTGGTTTAAAGCAATTACTAAGTTAGTTCGAGTAGATTTTACTATTTATGTTTCGTTCATAAATAAAAAACCAATGAATATAGGTCA<br>TGTACGAGTCGTCGTCATAGGTAGAATATAGATGTAAACCACTAATAGGTGGGTCAGTGGTAGGATGGATTTCGGGTTGAGAGTGAA<br>ATTCTCAATACTCCCGGGTTCGAATCTCCACGGCTACAAACACCTTAAGTGGCCACCACAGAAAAATTTAACATTGGGCTATGCGGCC<br>TCTAGGAACCACTCTTGCTGCCTTCGGGACGCACGCCAAGCTTTTGGGCTAGTGGGCTAGTCAGGGCCCACTGGGCTTTGGATAAGTG<br>GATTATCAACAAAAAAAAGTAGAATATAGATGTAGTTCTTATAGGAATATGCCGTTTCTATGTCAAAAGAGATACTAACATGCCGTTT<br>CTATGACAATATACAACTTTAACTCTTTCTGTTTTTACCAATTGTATTTAACTCCACATATATAAAGTTTCTATTAATAACTTTTATAAT<br>AAATTTGTAAATCTCTAGAACAAACTTTACAGTAATATTTTTTGATGTGCCAATAAGATCATGCGTGAACCACACTCGCTTACACGTAT<br>TATATTTTCTCTATAAAAAAATCATTATTAAATCTCTACTAACATTAATTACTTAGATGCTTTGTCATTTAGAAAATTAGGCAATTAAG<br>AACTCTTTCTACCATGTGTAAACTATTTTACATATTTCTTTATAAAAAAATTTATCTAACACATCATGAGATAGTGTGAGCACACGCGT<br>CGCTTACATTTACTCTATCTCTTATAGTAAATGTATGCAATCTATGTTTGATTTGCCTTAAATTGTCATTACACTCTCAATAGATTTTGT<br>ATATGTTTATATGACTGAATCATTTAATTATAGATATCTTGATTCTCCCTTTTTCATATACAAACAAGAGTTATAAATTAATAAATCCTT<br>AGTTTTTGACTAAGTTTAGCCTAAGGTTTATGGTGTATAGATAGATCTTCATATTTTATTAATACACAATCTTTGACACGTTACGCTCTC<br>AATCTCACTTTCTTAAACTCTCAACCCCTATCTCTTCTTATATAAGAAGGGACACCCTCCCAACACCAAAAATCAAAGCAACAACAAAG<br>TCTTATAGTCTAAACCCAAAAAATCAAGTGCTTCTTAGAGAGAA

Name	Gene ID	Promoter sequence
CsDof73	Csa12g037530.1	ACAGCGTACAATGCAAAAGAGACGGCTAAGGATTATGCGGAACGGACTAAAGACAAGGTGAACGAAGGTGCGTCTAAGGCAGCTGA<br>TAAAGCTGAAGATACAAAAGAAAGAGCCAAGAGTTATGCTGAGGATACAATGGACGACGCAAAAGCGAAAGCTAGAGATGCGAAAG<br>AGAAGGTCAAAGAGTATGGAGAAGATACTAAGGAGAAGGCAGAAGGGATTAAGGAGACTGTGAAGGGTAAAGCTGAGGAGCTTGGA<br>GAGAAAACGAAGGAGACTGTGAAAGGAGCTTGGGAGAGTACCAAAAATGCTGCTCAAACCGTGACTGAAGCTGTGGTTGGGCCGGA<br>AGATGATGCGGAGAAGGCACGGGCTGATATCGATAAAAGCGTGGAAGATCATACGAAGTCGAAGTGGAAGAAACAAGCAGAGAAAG<br>ATCGGAAAGACGACGATGATTTCATAACATTTAACTGATGAGGAAGAGAAATCTTTGTGTTCTTAGGGGTTCATTACTTTGTGTTCTGT<br>TTTCAGTTTATTCTCTGTTTTCAAACTAAACTTGGTCTCATGTATCTGACAATGTGAGATACATTAGTATATAACTAAATAAGACAAGG<br>GAATCTATACTTTGTGTTCTGTTTTCAGTTTATACTCTGTTTTCAAACTAAACTTGGTCTCATGTATCTGACAATGTGAGATACATTAGT<br>ATATAACTAAATAAGACAAGGGAATCTATGGTTATTTGTTTCTACGAGTACTAAGCTTTTCTTCGTTCCGACCATAGAAAATTTGCTGT<br>TCGAAGGTCATGATGTTTGTTTGTTTAATTTAGTGTACAAATCAACAAACTTAAATTTAATTTATCTTAGTGTTTTACAGATTCTTGAAA<br>TTCTGTTATCTTGTTAAAGTTGAAAATTCATTTCATTGTTACTATATATTGTTACTAAATTATTTTTGCTATGCATTATTTTCTATATAAC<br>CATTTATTCCTCTAACTTGTATCCACTTTAAGTGCGGTAAATTTCTATGAAAATAAAAATTTCACTTCCGTGATGCGCCAATGACAGAC<br>AACACGAGCATGCTGCGGTCAGGCACAGTGTCTTCTAACTCGAAAAACGAATATCACTAGGTGGTTATCGAAGCTAATCAGCTTCTTT<br>TATAGTTTTCTCTTCTACACTACTGATATGCTTGTTATCTAGATTTTGGTATTGTTTTCTACCTAATCCTTTCTATTGTTACCAATTGTAC<br>ATAATTTATAAATCTTTTTATAAGAAATTTTGTATTGGACCAACAAGAGCACCAATAAAACCACACATAAACCAGTTGTAAGAAATTTT<br>GTATCACACACACCATGAGAGCGCGTAAACTATACGCGCTCACAATAATTATTTTTTCTTTTTCTCAATAAATAACATTAATTTCATAT<br>TAAAGTTAATAACTGTGTTTGGTCTTTTCTCTTTAATAAAAAAATCATTGTTCAAACATCATATAAAGAAATTTCTGCAAAAAATATAT<br>AGTTATGCATCGTGAGATAAAGCGTGAGCACACGTGTTTTCACATAATCTCTCTTATCATAAAACCATGCATTGCATGTTTGGTTTGCC<br>TTATATTGTCATTAAGCACTCAATGACCCTAGATTTTCTTCTGTATGTGGGATTTGACCAATTTAAGTATAGCTTCTTGATTCTTAAATT<br>TTTTCATATACAGACAAGAGTTAAAATTAAGAAAAAGCTTTGCATCTTTGACTTAGCTTAGCCTAAGGTTTTTATGGTGTGTAGATAGA<br>TCTTCAGATTTTATTAATACAAAATCTGTGACACGTTTTACTCATAATCTCACTCTCCTAAACCCCGAAACGTATCTCTTCTTAAATTTC<br>CACGCCCAAGAACCTATATAAGAAGACACCTTCACACCTTCCACAACACAAAACAGAGCATAGCTAGCAACAACAAGGTCTTATAGTT<br>TAAGACTTTTTTTCTTTTTCTTAAGAGAGAATCAAATAGTTCTTGAGAGAA
CsDof74	Csa12g042020.1	CAATGAAGTCCATGTAACAACGTTTCTGGTGGACAACTGATCAAATAATTTGTGACCATGAGAGAAACTGCTGCATTTGAAGTGCATA<br>TCAACAAGGGCACTATCAACTATAATGTTGGACTTGATATGGCACTTAATCATCACAGCGTGGGCTCTCATTCCGTGTTCCAGCGATG<br>CTAACGCAGAACAAGCTCTAAACACTGAAGCAAATGTATTAAGGTCCGGAAATATTCTGTTGTGTCTCATATCATAGTAAATATAAAG<br>CCCATCTTGTTCCATACCCTTTTGCACATACCCAGATATCATGGCATTCCAGGGAATCAAATCCCCCTTACATTGCAAACAACGAAAGA<br>GAATCGCAGCAGTTTGAAGATCCCTTGATAAGGCGTAAAGTATCAACAACTTGACTTTCAAATACTCATTGGGAGCAAATCCTACAAC<br>AACCATCTGAGCATTATCCTTTTCCCTTTACCATATTCCTTTCTTTCTTTGCATTCCTGCATAAGTCTCAGGCTGTACTTGCAACCACAC<br>GCTATGCCACAATAACCCAATAGCTTCTTTCAATCTCCCAGTAACACAAAGAACCTTTAGTGTCTTATCCAGCTTCTCTTTCTTTCTCTG<br>ATTCTCCACCTGAAAGCTAGAAACATGTTACAGGTGAAGAACCAAAATTTGATCTATCAATCAACGTAAAGCTTCAGACTTTATACAA<br>CCTCATAAGAAATGATCATAGCGTCATTGGACCAACTTAAGCATAAGTATATAACAGGGCTCAGCATTCATACCGACAAAAGAAGTAA<br>GAGTTGTAATTTTGGAAAAAAAACTGAAATTGTGGTATTTTGAGGAATTTCTATTTTATTATTTTCAGCAAATATGGTCTATAATTATG<br>CCATTTTAGCAAATAACCCTTTACCTATGTTCTTATATATTATTACCAGTGTTCAACAAAGCACTAGGCGGTATCTGGGTGATGACCCA<br>ACGTAGAGCGTCTAGAACGCCTAATTGGGGCCTAGACGGTTTTTAGGCGGTTTAGGCGTTTACAACATAAAACATGTTATATATGAAA<br>TTATGTACAAATATATGTTAGAAAAATAAAAAAATTATAAATTATAAACAAAAGTAAGAAAAATACATTTATTTAATTTATATTAACA<br>ACATATAAACATTTATAATTACGTATACAGATATAAACTTAATAATTTAACTATATGTGGTTAAAAATCAAAAATATATATTAAATTT<br>AAATTAATGTAATTTTAAGCGGTTCAGGCAGTCATCTAAGCTTCTGCTGAGCGCCTAGGCGCTGTCTAGTATTGATAATAACTTTTATA<br>ATAAATTTGTAAATCTCTAGAACAAACTTTTACAGTAATATTTTTTGATGCGCCAATAAGATCATGCGTGAACCGCACTCGCTTACACG<br>TATTATATTTTCTCTATAAAAAGATCACTATTAAATCTCTACTAAAATTAATTAAATGCTTTCTCATTTAGAAAATTAGGCAATTAAGA<br>GCTCTTTCTACCATGTGTAACTATTTTACATATTTCTCTATAAAAAAAAAATTATCTAACACATCATGAGATAGAGCATGAGCACACGCG<br>GCGCTTACAATTACTCTATCTCTTATAGTAAATGTATGCAATCTGTGTTTGATTTGCCTTAGATTGTCATTACACTCTCAATAGATTTTG<br>TATATGTTTATATGACTGAATCATTTAATTATATCTATCTTGATTCTCCATTTTTCATATACAAACAAGAGTTAGGAATTAATAAATCCT<br>TAGTTTTTTACTAAGCTTAGCCTAAGGTTTATGGTGTGTAGATAGATCTTCAGATTTTATTAATACACAATCTTTGACACGTTGCGCTC<br>TCAATCTAACTTTTTTAAACTCTCAACCCCTATCTCTTCTTATATAAGAAGGGACACCCTCCCAAAACCAAAGATCATAGCAACAACAA<br>AGTCATTATAGTCTAAACCCAAAAAATCAAGTGCTTCTTGAGAGAA
CsDof75	Csa12g081790.1	GGAAAAGGTATATATTTGTTATAGTTTTATGAAAAAATCTACTACACATAAAATTTTGTTGTATAACTTTTTGTTGTTATATTTTGTTTT<br>TGTAAATTATAAAAATCATTTATCTTTATTTATTTATTTATCAAAAATACATGAAAAAAATTCTTATTTTTAGGAAAAANNNNNNNNN<br>NNNNNNNNNNNNNNNNNNNNNNNNNNNNNNNNNNNNNNNNNNNNNNNNNNNNNNNNNNNNNNNNNNNNNNNNNNNNNNNNNNNNNNNNN<br>NNNNNNNNNNNNNNNNNNNNNNNNNNNNNNNNNNNNNNNNNNNNNNNNNNNNNNNNNNNNNNNNNNNNNNNNNNNNNNNNNNNNNNNNN<br>NNNNNNNATATTTACTTATTTATAAGATTATGTCATTTGAAATATGTCCAAGACATTGCCAACTATTTCTTTATTTTTAATAAGAAGAAG<br>AAAGGAAGAAAATAATGAAAATTGTATTTATTTACATGAAAGACATATATCTAGAAGATACTTAAAAAACAAAAGTTGATTTTGTAAA<br>ATCAAAGGTTGGTTTTGTAAAATCAAAAAAATAGTTGGCAGTGCCTTAGATATATTTTAAATGACATAATCTTATAAATAAGTAAAAA<br>GAAAGAAGATATATTTAAAATCTCCACCTTTGAAAATACAAAAAAAATGATTGTTAAAAGGAAAAGGTATATATTTGTTATAGTTTT<br>ATGAAAAAATCTACTACACATAAAATTTTGTTGTATAACTTTTTGTTGTTATATTTTGTTTTTGTAAATTATAAAAATCATTTATCTTTA<br>TTTATTTATTTATCAAAAATACATGAAAAAAATTCTTATTTTTAGGAAAAATAAACTTTTTTTTTGGAATATATTTTCTGGTCAACGCC<br>GGTCAACTCTGGTCAACGTCGGTTAAAACTGGTCAATATCGGTCAATACTGGTCAATGCCGGATTCCGGCGAACCAACCACCAGAACT<br>GAATATTTATGGTGTTGTACACATAACCTATATTTGTTTATGGATGTCCTTATGTGATGACAATAGTTGACGTAGTTAAGAAAACATTT<br>ATATTGTTTGTATGTGTGGCTATGTGTTAGAGTATACTTCAAGTAGCTGACAAACATTTTATATCTATTTTTTAGTTTGCCGGTTTATAT<br>AATTTCAACGCTGATTTTTTTTAGTTTTCCTAATAAAATTTATATTAAAAAATATAAAACTGTAAATGAAGAAAAGGTAAAATTCAGGC<br>TACACTAAAAAAAAAAATACTCCCTCCGTTTCAATATATAAGATGTTTTAGCCTCTTTTCTTGTTTCAAAATATAAGATGTCTTGTAACT<br>TTCCATACAAATTTTAATATATATTTAATAATCTATAATTATTATTTATGCAGTCTTCTTTATTATTGGTTGAATCTTTTTTTAATTTAAA<br>TATATATGATACTTTTTGAAAGAAAAGTAAACTATCTTAATATGTGTGCCTTACCTTAAACATCTTATATTTTGAAACGGAGGGAGTAC<br>AAAACTGTAAAATGGAGAAAAAGTAAAAGAAAAAAAAGTGCTGACCAGCTTGAAAAGGAAAACAAAAAGAAAGCCAAAACGAGACG<br>AAAAAAAAAAAAAAAAAAAAAAAAAGAGCAAACCTTTTAAATTGAAAAAACAAAAAAGTCCACAAAAACAAAAGTACAAAACTTGTGG<br>CTGAGAAGCCTCACGTGTAATGACGTGGCACTATCGGCGAATCAAAAGGTTGCACAATTCGAAAGATCACACAAAATAAAACAAAAA<br>AGTGTAATCTTTTTCTTTTCTATCTCTCTCTCTCTCGCAGAGACAAATAAACAAATCCGAGAAATACTCAGACAACCACCGAATACCAC<br>AAGACTGTCCTTTCTCTCTCTCTCTCTCTCTCTTTCCACAAATAAAAATTGTAACTTTTTTATTTTCTTCTCGAAAAGTTTCCGCTTTT<br>TTTTCGCTGGAGAAGAAGAAGAAGAAGAAGAAGAAGTGAGAAAAAAAAGAATCATTGAT

Name	Gene ID	Promoter sequence
CsDof76	Csa13g002360.1	TTCTGGAACCTCAGGCCCGGTTTGCTGGAACTCGGGATCCATTGATACTCGCTGACCAAGAAAACTGAGATTCTATTCCCAAAGTCTC<br>CAAGAAGAAGAAGAAGAGCAAGTGATGATCTAATTGGTATACTACAAAATTATACATCTGGTTTAATGTTCAACTGGGAGAGACTGTA<br>AAATGACAAATTCATAGGCCAACAAATGTTTGTTTATCCAATTTTCCTTTTTATTGGAACTTTCGTGTAATATCTCACCAGAAACCGAG<br>TCGGTTTACTTTTATCTGGGATTATTGAATTAAACCAAACGTAGCCGAATTAAACCTTGGCTATCTTGTTTACTGTACTGTATGTAACT<br>AAAACACTAAACAATTTAAGTTTCCGGTTAGGTCCGGTTCCCGAAATAGCGTTAGGCGATTTATATAGTTAAGTTAGGTGTTCAAATT<br>ACGCGCGTGTACTACGAAAAATCTAAAATTCCAATTCCCATGCAAAAGAAAAAAGACATCGCGGCGGTTCGAGGGAAAGAAAAGCAA<br>AACAAGTTGGGGACACTTCGGAAGAATGTTGGACTGTTTTAATTAATTTACTTTATATACATTTTTAAATTTTCCTGTATTTCAACTTAA<br>TCAACTCATCTCTAACAATTTTTAATCATTTTTTTAAATATATTACGAGTAATTAGTGTTGTTCCATTTAGTCCAACCTCTCCTTTATGT<br>TTAATTCGTATTTTGAAAGAAGTGGAACCTCATCTTGTTAAGATTCATCGTTAAGTATGTGAAATTGTGATCAATCATTTTCGTTGATA<br>TTTCTTTTCTAGCTTGGTCTCATATGTTTTCAAGTTTGTTGGCTGACGGTTAATTTCTTGATATGATTTACGTTTTTCTCTTTTTATTTTT<br>CATTATGCGTCAATATGTTTGACTTTATATTTCACATTTTGTTTATCCTTTGTTCAGTAAATCTACACATTTCGAAATCTACAAAATCGT<br>CCCCGACATCATCACATCACTAAAGAGAGCTCTTTCTTTACTGATATTACTGCCGATTAATATATTCAAGATAAGTGTTTAATTACTTA<br>ATTAATTAAAGCTGAGGTATATATACACGTAATATAACACAATTAAATGCATATATAACTTAGCTGCAGTGGCGGATCCAAAACATAA<br>AATAACATCATTTCTTTTACTTAAATGAAAATAATGAAAAATTTAGCTGATATAATCCATATATAAAAGCACTAGCTAGTGTCAGCTCT<br>AATTTTCTTATACAAGAACTAAGATATAACAACTGCAGTTTTTTTCTTGAATACTAACTTCACCTATACCCAACTCTTACGTACGTTTAT<br>CATCTTTTATTTTGTATTTGATATTTTGCATGGTAATAAAATGTTTCTAAAGTTCATGACAAACATCAATATTTATCATTCTTAGTGTGG<br>TATCTTTTTATGTATTTAAATTGTTATGAATTTTACCGACTTTGATTTTTGAAAAACACAACCAGTCAAGCTTAGTACTTTTTTAATATA<br>TATATATATACATATATATATATATTTATAATTAGCACACACAGTACAGATAGCAGTATTGACAGAAAGAGAGAATCAGAAAGAAACT<br>TATGAGTTAAGCAATTGAACATACTCACTCTCTCCTCCACCTTCCCAAAACCCTAAGCAAATTTGTATATAATATATAAAACAAAAAGA<br>GCCTTGCAATAACACTGTGGACACACTCAAATCTTATATTTTTTATTCATCTATAAAATAATCAATATAATACTCAAACATATTCCTAT<br>ATATATATACATATGTGGGTATGTATATATATGAATAAAGGTTCTTCCTTTACCTTTGCCTTCTCTCTCTATCTTTCTCTAAGCTCTCCT<br>CTCCTCTTCTTTTCTCTCATATAAGCCTTCACCAAAACAAAAAAGCTTTAATTCCTTTATTTGCTCAAAAACAAAACCATAAAAAAAAA<br>AACTAAAGAGAGAAATATCATCCAAGAAGAAAGATCAAAT
CsDof77	Csa13g056100.1	GTCCTTATAAAGAGGATGTAGAATATATACTAGCACTTAACTTTGTTTTCTGGTTGGATCAGACGAATGTGTATTCAGGACTAAAGCA<br>AGTGGTTCCATGGAAGAACAAGAAGACTACAGTTTGAGATGCTGCCCTGTTAAAGATTTATACAAGACTAAAAACAAAAACAAAAAA<br>CCAAGAAAGCTCCTGTGGTTTCTACATATATCTTCAAACTTTTGCTAATGATTTTTTTCTTTCTTTTTTCAGTATTTCCAAGAAAATGAC<br>ATGACCATTATTAAAATCATTACGAAGCCCAGTATCTTTCACACTTATTGTTGTTGTTTGTGTTTGTAATCTTTGAATATTTCTTGAAAC<br>TTATGTATAGAGAAACAGAGATGTATAATGGATATTATTCATCATGTTTGTAACAAAGAAAAAAAAAAAAACAAGAACCTTTGAAGGCT<br>CTTTCTTTACATGTATCATGTATGTTACCTTCTTGATAAAATCTGCTTCCTTTAGAAGCTCTTTCCGGTAAAAGAAATACACATCGAACT<br>TCGATTTTAGTGAAATAATTAAACCAAACAAGATAACATCGAATTTAATTGCGCAGTAGAACAACATGAGGAGAACGGAGGACCAAA<br>ATATTACATTTTTTTTATTAGTTCAAACTCCAAAGATTTCAAGAATCATTCAAGAAAACCGCAATGATCTGGATTTCATACACGTGTCA<br>ATTGGGAGTAGATCGATGGTGGAGAATGTGTGGACTCTCACTGTTATTTCATATCCCACTCAGACACGCAGTCTCGGTCATTCCTTTGA<br>AATTGAGTGAGAAGGCCTCAACCAAGCCAAGTTAACGTTCATATTTTGGGCTAAGTTAAACATTCGGCCTACTTTAAGCCCGTTGATA<br>TATGATGTAAGCCTGTTAACTAAGGAAAAACATGTAAAAGTATTAATTTCTCTAGTTATAGTTTATATAACGATCGTTATTGCTACATT<br>TTAAATCAAGGTTTAACGATTTTTAACGTTATTGCTACATTTTAAATCATGGTTTAACATAATTAACGATTTTTTTATTCTTTTCCTTAA<br>TCATGGTTTTAGACTTTAAGTGCTACATTTTAAATCAAGGTTTTAGCTTAATTTAGTTAGTTAGATGTATAGTTTGTATACTCTAAATCT<br>AATTTGTTTAGCCTCGAATTGAAAAGAAAAAAAAACAATATGTTATGCTAAATATCAACTACTTTATGATGATATAACCATCTATTAAA<br>AAATAGAGAGAAATAAATGATAGTCAAAACAAACACACCACCACTAAAATGCCCATAAAAGTGAAACCCAACACTTCTAAACTTTAGC<br>CTCAAAATATTCTCTTTCCCTCTCTCTCTCTAAGTCATCTCTTTGAACATCAGGTCAGTACATCTTCTTCCCTTTTTCTCTTATCTCCTTG<br>TTTCTTCTGCAAATTACTTTCTCTATTCTTTTTTTTTTTCCTTTTTAACTTTCTTTTGCTTTTTGGTTTGTGTTTATTAGGTTTTTTTTTTGT<br>TTTTTTTTGTTTTTTTTTGTTTTTTTTTTTGTTTTTTTTTTTGTTTTTTTTTTACTTTTCAAAGGTTTACATCTTTCCACCTTCTGATTTCTT<br>TGAAAATGTGTCCCTTATTTAAAAGATATGTCTGGCTTTTTTAGAGATCAGTTCCTAACACCTTTTTATTAGTGTCTTGCTCGATCCATA<br>CGTTTAGAAAGTGATTATTTGTTCCCACTCTCTCAGATCCTTTATTGATTAACTTTGGTCGTTCTCATTCATATACTAGACATAGCTCCA<br>TGTATATATATATATATATCTCGTTAGTTGGTTCATGGTCTTCGACTTTTTATGTTCTTTCAATAAGTTTTAGTATTTTATTTATTTGTTA<br>TGTGTCACTTTTTTATTTGTTCTACTTTTGTTATCATGTTTTTCATATGCTTCATTAGACGGGTTGATCATACATGATTATCACAGGGAT<br>TTATTATAAACTGTAATCGAAGCTTACATC
CsDof78	Csa14g009010.1	TCCCACCAAAAGTGTAATAGGGTATGATGATGATATGCTTTATTTATGGCATATGGGGCTTGCCATCTACATTATAAAGAATAATTGTA<br>GAAAATAATTTATTTTTGTAACTTAATTTGCCCCAGTATAATACATCCATCTTTTTCATTAATAACAAAAATGTCTCTATAGAGAGGCA<br>CGTACGTGAGAAAAAGAGAGTGTTCGGCGTTGGTACTGAGCCTCCATAATAATGAACAAAACGGCGCAGTTTGGGATTAAGATGATC<br>AGAGTTATATGAGTTTGATTTTTAAAGATTTCCGAGTTTTGATGAAATCTATGACAGGCCAGTGAATACTGTTTAGAATGTTGGAATG<br>AGTATAAATTTTTGTGATTTGCATGGTTTATGTAATTATGAAAAAATCGACTATGAGAAAAATGAGAAAACCAACAATATTATTGTAG<br>GATAAACACGTAAATGTTTCTAAAAGTTGTAAAAGTACCTCGAAAGATATTTAAAATTTATATTAGAAAAAAATTAGTTCAAAAATTG<br>TTATATTACATAACTATATTACAAAATATATATTTAACGTTTCTTCAGATTAAAAAAAAGTTATACAACTTCGTATAGTTTCGTCGAAC<br>GTTTCTTCTATATATATTATTCCATAGACGCCTAGGAGAGGAACCTTATAGGCGAAGGAAAACGTCCCCACTCAAACCCTAAAAATGG<br>GAATCAGAAATAAAAAATAACACACAAATGAAGTATAGAGACACGTATATGAGAGTGGAATTATAGAAATATATAGAAAGCATAAAA<br>AGAACCGAAATCAAATTCCTTTTCTTTTCTTTTTTTTTTTCTCATTAAAAAGCATTTTTCTTGCCTTTAATTAATTTGTCTTTGTTCTCCT<br>TCTCTCTTCTCACTTTCCTTTCTGATTTTGTGCTCTCTTAGCGTTTCTCTTTGGTCGTCTTGCTTCCTCTCTTTCGTTTTGCCTTTGTTTGT<br>TTCTTTTTTTTTGATTCCCCCACCAATTTTTCCCTTGGAGTTCTTTCCCCTTAAAGATCCATCAAACTTTATTAAAGAAGGTCGAAGAAA<br>GAGATAATAAAGGGTAGAAGAACAATGGCTTTCTCATCTAATTGGTCACAGGTTCACTTCTTCTTTGATCTTCCATTACTAAATGTTCT<br>CTCCTCTTTTTGTAATTTCTCTCTTAAANNNNNNNNNNNNNNNNNNNNNNNNNNNNNNNNNNNNNNNNNNNNNNNNNNNNNNNNNN<br>NNNNNNNNNNNNNNNNNNNNNNNNNNNNNNNNNNNNNNNNNNNNNNNNNNNNNNNNNNNNNNNNNNNNNNNNNNNNNNNNNNNNN<br>NNNNNNNNNNNNNNNNNNNNNNNNNNNNNNNNNNNNNNNNNNNNNNNNNNNNNNNNNNNNNNNNNNNNNNNNNNNNNNNNNNNNGAT<br>TTTTAAAATTTATATTAGAAAATTTAGTTAAAAAATTGTTATATTACAAAATATATATTTAACGTTTCTTCAGATTAAAAAAGTTATAC<br>AACTTCGTATAGTTTCGTCGAACGTTTCTTCTATATATATTATTCCATAGACGCCTAGGAGAGGAACCTTATAGGCGAAGGAAAACGT<br>CCCCACTCAAACCCTAAAAATGGGAATCAGAAATAAAAAATAACACACAAATGAAGTATAGAGACGCGTATACACATGAGGATATGA<br>GAGTGGAATTATAGAAATATATAGAAAGCATAAAAAGAACCGAAATCAAACTCCTTTTCTTTTTTTTTTTCTCATTAAAAAAATGCATT<br>TTCTTGCCTTTAATTCATTTGTCTTTGTTCTCCTTCTCTCTTCTCACTTTCCTTTCTGATTTTGTGCTCTCTTAGCGTTTCTCTTTGGTCGT<br>CTTGCTTCCTCTCTTTCGTTTTGCCTTTGTTTGTTTCTTTTTTTTTTGATTCCCCCACCAATTTTTCCTTGGAGTTCTTTCCCCTTAAAGA<br>TCCATCAAACTTTATTAAAGAAGGTAGAAGAAAGAGATAATAAAGGGTAGAAGAACA

Name	Gene ID	Promoter sequence
CsDof79	Csa14g026580.1	TTAAAAAAAAATTGATAATTCTAATTTCTAAGTGTTAGAATTTTACCTTGATATTATAAGTTGCATACTTGTAACAAATTATATATTTTT<br>GTTTATATCAAATAAATAGTACTACTAGTATATAATTAAAGTCAAAAGCAATTTCGAGTCAAACAGGAATTAATAATACAGCTGCGAA<br>TGAATAAATGCCAATCAGAAAAAGAAGAATTAAGGTTTGACTTTCATATTATTCACAATTTTGTAGGTATTTTTAATCATTAGACTACG<br>GATATCGTATCGTAAAACACAAAATAATATTGGGATCGGGGCTTACCATAGATCTAGTTTTAAGGCTGCCCAATCAAAACTAATCGGT<br>GGTGATCAACAATGATAAATTAACAATCAAGTGTTACAATAATGATAAAAGTGCTACAAAATAATACTCTCTCTATTTTAAAATTTTAA<br>AATATAAGATGTATTAACTAAAGCACGTAGATTAAGAAATTTTTATTTTTAACAAGTTTAACCAATTAGAAATAATATTGCATAATATA<br>AAATACTAAACTAATTTAAAAGTTGCATAGAAAATTGAAAATATCTATAAAATAAAAAATTTCTCTAAAACATCTTATATTTTGAAATG<br>GAGGGAGTAAATACATAAGAGAATTCAACTTGGGGGAATAAGAAAGAAAGAGAATTCAACTTCGTAGATGTGAAGGAATTGAGAGA<br>AATAAGAGAACCATAGGTCGACACGAGGGTAAAAGCACGTGTTCTCCCGTGCGTGTGGCCCTTAATCTTGGCGTTAATCTATCTTAAT<br>CTTACTATATTATCATGGTTACATAATATATGCTCTTAACGGTCACTAATCTACATTTGTACTTTAATTCAACAAATGAACATTTTGGCT<br>AGTAATGAAAATCATACTAATTGACCGAGTTAATGTTCCCCGCTATTCCAAGGATCATTATTACTCATCAAACCCTATATGGACAAGCC<br>CTATCAAACAAGTTTATATGTGAACTGTGAAAATATAGTTGAGTTGATCGGACATATATATTATATAGCTATATATGAAAGACTATATA<br>TATACAAGTAACTTGGTGAATGGATAAACAAAATGACAATAGTAAGAATAATGTACCGACGCTTGGAACTTTGGAAGTGTTAAAATGA<br>TACTAATGTAGAATGAGTGCAAGTGATCAAAATGTTACGATGCTTTGTTTAATAAATCCAGTAGTGTCTTGCGGTTCACGAGAACTAG<br>ATGCTGAGAATGTAAGTGACTAATCATCTACACACTAATATAATATGATGCCCCTACGTTAATATTTCAGAAGAGGGTAATTATAGTA<br>TTTTACTTACTTACATTCACGGTTAGGTCAAGCAGACAGAAAAAAGGGAATGCATCAACCAAAATTTTCTGATGTTTCCTCCAATAGAT<br>ATCTATCGAGTTGTGGCATTAAAAAAAACTCTTTAAAACCACTTGAACAACCTTGGTATCAACAATATTTTTATGTAGAGGTTTTTTGT<br>TTTACCACGACTGTTAACTGAAAGAAAAATGATGGCACAAATGCACATACGTTTATACTGTATTATTTTAATTTGCTCTTGGATGGTAT<br>GAATTATGAAACATAAATTTCAAGTTGCCTCAACAAAAACTTTCTGAAATTCATCATCCAATGTTTATCAAAACTTGGATTATTAAATG<br>GTCAAATAATTGATAAACATTTTATTGTCATTATATAAGTAAAAACTATTACGAAGGACCAAATTCGAAGGTAACTTGTTGAAGAAGA<br>AACGGAAAAGGAGACGCACGGCTCGGCAACCGCCCACGTTTACTTCGTAAGCCTTCACACATTAATCGATGCACCTCTGCTTCCACAC<br>ACACATGCTATTCCTCACCTCTACGTTCACACAATCCCATATATATTAGAGTTACATGTACTCGAATATACCACCAAGTGGCCACTTGT<br>TAGCCTTTCCAATCTTCTTTACACCAAAAATTCATAAAGAGTATATGAAAA
CsDof80	Csa14g031920.1	TAATACGACATGGACCTGCTTCAGTAATATACCAAATACATTCTTGGCAGATTCCAGATACACCTTTTGGATTATCATCTCTATCTGCT<br>CTAAAAATATCAAACGTAGCTGTTCTGTTGGGCCACTCGAAACTGCAAGTATATTTTGTTGTTTTACGGAGATTGACACGAAACCTGA<br>AATCAAAACTATCTTTCCAGTTGAGTCTTATTACTCCTAAATCATCCCCTTTGTTTCTACAATGCAGAATCAACATTGCATGTGAGACC<br>AATTTGTTAACAATAGTAACATGTTTAGCTGAAAATGGTAAAAGTCCATCAGTGGACGAATGGTTTTCTAATGATGCCGACGTTTTGA<br>GAACAAAAATAAATAAGACTAGCACGGAAATGAAAATTTGATTTGTTGAAGAAGCCATGGTTGTGGTTTAGTTTAAGAGATTGTGAG<br>AAAGCATGGATTTATAGATGAAACTACATTTATTTGGCTTAATGTTTTTACGAAAAAATATCTTCTAAGATAAATGTGAGAATCCTTAG<br>TTTGTAACTTATACAGCATACGTAAAAAAAGAAAGATATTGACTGGTTTCTAAATTATTATTATTCTTTGGATATAAAGATGGTCAAAT<br>TCATGGAGTTCCCTATTTTCTTCCTATAAACTCTGATTTTGTTATTTAATAAGCAATTTCTTCCATAGGTATGTATCTAATTTTAATAAT<br>GATCATAAAATTTTATATTTATAGCAGCAGATAATTATTTAAAGTTACTTATTTTGTTATTAATATCATAGTTTAATAGGGGAATATGC<br>TTAGTTTAAAGGTAAATAATTTATATATTACCTACTAAAACCATCGGATTAATTAGGTTCAATTCATAATTCATGAACCATGTAAGTTT<br>CAATTTATTAGATTATGGATTTTGTTGGGTTGGTCTATTTCACACTTTTAATTTAGAATAACATTTATCTAATTTGTATTATTTCGGAAA<br>ATTCAAAGGAAAAGGAACTCAAACTGTATTACTATTTGGTATAAACAACCCAATTAAATCCTGCCATTGAATTGACTCAAACATATAC<br>TATAACGTTATATCGGTACATTAAAACCTCTATAAATTAATAATGTTGGGACCATGAAAATCTATTAATTAACTTATAGAGGTTTTAAT<br>TTCTCGATAAATTAATTTATAGAAAGGTTTTCCTAATTTGGTAAAAATAGTAAGATTTAACCTTTATTACTAATATTTGTATAAAATCA<br>ATTACAAGGAAAATAACAATTATCATGTTTTGAATATAACACTCAAAGATTTTTATATAGTAAAAAAGTTAAAAATAAACTCTAATAA<br>AAATATGTATATATATATACATATATTTAGATAATTTTATATAATTATTAATTTATATTATTGATAGGACTATATATTTATAAAGGATTT<br>TCAAAAAAAAAAAATTATCTTATTATATTATTGAATAATGTCCAAAATTACACTAGTTCCATCTAGAAAATTATTAATTTATAGAAATTA<br>TTAATTTATCGAGTATTAATTTATAGAGATTCTACCTATATGATACCGATTTTTAAATTACGTATAGCAGCTGGCGTCTTTTGCATATGT<br>TTTCGCACTTTTGTATTTTTTAATACGAATATTCCAAGGAAAAAAAAGATTGTACTACATTTTAATGGCCAAACAAAATTTTGACGTGG<br>CAAAAAGTAAAGGGAGCCAAATCTTTTGAGTGGCACTGAAAGAGAGATCAAAAAGAGCAATGAAAAAGAGATAGCAAAAGCTCACTT<br>TTTTAGCAACAGCATGTGGCTATCTCTTCTCTACATGTCCCTGTCTCTGCTTACGTGTAATTTTCTCCTCTTTGTGTCTTGTGGCTCTGT<br>GCGTTCCATTATTAATTTAATACCAACCTCAGCCTCTTCTTATACATAGTCTTGTCTCTGACCTCAAATAAAGAATAAACACACATAAC<br>ATCAAATCTAATCTCCTCTGCTTCATCTTTCTTTTTTCTGTCTTT
CsDof81	Csa14g036700.1	TGAGAAAAAACATATAGTATAAAAAAAGCACGAACTTGAGAAAAAAATAAAGAGAAACATGAACATCGATTAGTGCTTTCCTTTTCCT<br>TTTTCTTTTCCTCTTCTTTTTTTTTCCCTTTCTCTCATCTTTAACATGCTTTAGAGTTTAATAACGTACGTGAATTATGTTAATTTAAATA<br>AAATATTTAAAGAAACATGCTTTGCTTAACTTTATTTGATTTTTTTTCCAAGGCAATATCCACTCAACCGTTCTACTAGATGATCAAGC<br>AAAATTGCACAAATTATTAGTCCAATTTTACCACTATCCACGGACCATGACATGCAAAAAGGGATAGATGTTCCATTTTAGTTTAACAT<br>AATCTTGTTAGTGCAGCTAGAACTATATTTGCATATACTATTATTATAAGTTAAACTCTTCCGGATTGAAAACCCAATTTCATTTATTC<br>ACAAGTTCACAATAAAAAAAAAAAGAAGAGAAATTCTCGATTAAATTTCAGTTTTATTATTTTATCCAAAGAAACTCTCTAATTATAATA<br>CTAAGCAAAAGAAGAAACTAAATTTTTCCTAATGAAATTTATTTTTCTATACAATACACAATGAAAGAAAGAAATAAATAAGTGAAGA<br>AAATGGAAGGATGACTTGGAAAAAAGGGAGGCCAAAAAAAGATGATGTGTGACTTGAATTGAAAGCTGATCCCCTCTAGTCATTCAG<br>GCTGCTATTCATCTCTTCTCCTTTTCTACACCAACACAACAACCGCTCTTTCTCTTTGGCGGCTTTGTCCAAATCCAAATCCTTCTCACT<br>TACATTATCTTTTCAATCGCATAAGGTTTTTTCCTAATTTCTTATATTCATTATATTTGTTGAGATTTTAATACAATTTTCTCGGTCTATA<br>TTCTAAACCTTACATTTTAATCTCATATTGAAGCATTGAGGTCGAACCCCTTACACCCGAGCGGTAAAGCAGAAGTGAGAAGTCGCTA<br>CTCAAGTTGCTATCTCGACTCATACTTCCAAATATTTATAATTCCAAAAATGTTAGTCAATGATGAAAATAAGAAGAAATAGATGATG<br>CTGTTGCGAATTTTAAAAGGATAAATAGTATATCATATAGATAACATAATTAGGTTTTGAATGTGATATTTGTTTTGTTTCTGTGACAC<br>AACCCCAATTCAGAAAATACCCCTTCAAATCACGTTGTCGCACAAAAGTATAAATTTTAAAATATTCAATAATTAATATCAAACCATAT<br>ACATTTTGTGAATCAGTTAGTTATATAAATAAATATTTAGGTAGCTTGTGAATTAATTATAGTAGTTTTTGGCTGTGTTTAGTTATTGTT<br>TTGTATTTCTATATTGCTTGTTCGCTGCTATTGTACCAAAGCTTTAACTCGTTTGATGCAACGAAATAAAGCTCATTCTCTGCATCTGTT<br>TTGCGAATTAAAGCTTCTAAAGATGTTTTGAACTTTTGCAAGTTTAACTAACAACTAAACAACATCATTTATTAGTAGTGATCACTTAG<br>AAATAGTATTAGCAAAGGGAGTAAATTCAAAACCAAATATAATTCACATAAATTTTGATAAAAATCATATTAATAAATAAAAGTGTTA<br>AAACATCACTATTCCAAAATACCTTTTATAAAATATACGGAAAGACTATGAAGACTGTACTGAAAAAGTTTGAAAATATACACATTAT<br>GGTGAAAAAGCAAAAGAATACAACACATTTTGTCAGCTGATATATGGATCGATATTTCCTTGAATGCAATAAGCAGTAAATGCGCCTA<br>ACATATTCTTTCATAATATTTTTATATTCTCCCTACTAACACTATTGCTTTTCTCTCCTTTCCTACGAAAAACATATAATAATGTTTTCAT<br>TTTCATACAAATCTTTTTACAAAAAAGATTGAAATCTTCTTCTTATAAACGTCTCTTCTTCTCTCTTTCATCACTGCAAGAAACTTAAAT<br>CACAACTCTCTCTTCTCTCTCCCTTACTCTCTCTCATTTTA

Name	Gene ID	Promoter sequence
CsDof82	Csa14g051530.1	ACTCATTGAGGGAAGTCTAGAAATGGTTGAAAGCTCCAATGGAATTTTGACATGGAAATTTCTCCTTCAGTAATCATAAAAGAATAGT<br>TTCTTTTCTATATACCAGACCACCATCAAATCATCGATTTCATGTGTATAGTTACATCCGAAGCTAACAATATTTGTGTAGTAGCAGAG<br>ATCCACTTACCAAGTCCAACAGCCTGAAACTTATGGTTTTGGGAGATGTTCCTATGTGTCAGTTGGGCCAGAGCATAATATAGACCAG<br>CAACATCAATGAAGCCAATCATTGCTTTCAATGCTTCCTGATACAAGGGAGATGGAACCCATACTTAGTCATCTAGATTGTAACATAA<br>CAAAACAAGCTAAGCAGTTACAGTAGAAGTTGATAGCAAAAAAGAAATTCAACAATGTGCATCTATATAATAAGAAAAGATAAGTCT<br>GGTTAACATTGTATTATTTGCATATCATTTTACTTAAAGAGTAAAGATGACAACTTTTCACAATCTTTTAACAGAGAAATAAACAACAG<br>ACTCATATAAAAAAAGTGTGAGTGCTACAGTGATCTTAGCTTAGCTACAAGTAAAAAAATACCTGGTATGGGTCAAATACTTCAGTCT<br>CAGATACTTGCAGGAAAGTTGCCAAACAAACAAGCTGTTATGAAAACAACCAATGAGCTGAGAATTAAGGTATAAGCTTAGCTAAAG<br>AGATTCTCCCAAAGATTGTTATAGTTGACTAAACCTTAACCAAAGCTGTAGCAAGGTAAACAACAGCAGCTTTCACGGAGGTTCCAAG<br>TGTGTCATATTCAGATCTGCAGATTCACAACTTCCCGGTTACAAACTATACCTTAACATATAATCTAATCCCAAATTCAATTTGGGGAT<br>TCTCAACACCTACGTTCTGCAGAAGCTCATACTAAGCCTTAAGATTCTAGATCACGAAGTTTAGAAGTAAGCGATTAAGTATATGCGC<br>AAGAGGATCTAGATAAAATCTTAGATTAGGCATGAATAAACTAAGAGATACTAAAATTTGAAACCACAGATCGAGAAAAAAGGAGAG<br>GATTTGTTCATACAAAGGAGTGGCAGAGTAGTAGACGGCATGAGGACCGAATGTGAGAATCGCACAGTTGAAGAAGTGAAAAAGCGT<br>CATCGTCTCTCTTTGTGACACCAAATCGGGAGCTACACAAGTCCGGCGACGCGAAAGACGACGACCACCACCACGACGTGAAGCTGT<br>GAAATTGAAGAAGAGGAGGCTTGGTTAGCTGAGAAAATCTGAAAATCTGATGGGATCAACAAGTCTTAATGGGTTCCTAATTGGGTCT<br>CTCTACTGGGCCGTATAATTTATAGTCTATGGCTACGAAAGTTATTATATGACGATGTTTTTTTTTAATATGATGATGTTTATAATTTGT<br>TAAATTCATATTTGATTTATTTGGGGGTCAAATTATCTTTGAATTTTTTTCTGAAATTATAGGTTCATATGAAATCTTATGCTTTTTCTC<br>ATATTTGATATTCCTCTTTTTTCACAAAAATATTTTTCTCAAAATTTTAAATATAGTTTGAACTTTTCGAATTTTTTTTCTTAGTTCAGAT<br>ACTATTTGACTATATGTTAACTTCTAATCTTATAAGCGCCTAAAGGCCTAAACCCGTTTTAGATTGTCTATATCCTGATTAATTGCAGG<br>TGCAAAATTTCAGACTATTTAAGTGTATTGCTATGAAGGAATTGACCTAAAACAAAAATCAAATTATAAATCTGTAACCAAAATCCAT<br>TACCAACCATTTTCAAATAAATCTATCAACTAAAACCTTCAAAATTAAAAGGAACATATAAAAATGAAGTATATCAAAACAGCAACTG<br>AAAGTAACGTCAAAGGCCTCTCTCTATATAAAAACCACAACACCACTCTCACAACATCCCCTTCTCTTTCGCTCTCTCTCTCTCTTCTTC<br>CCCTTCTCTCCTAATTATAACTTCTCTGTCGTTTTCTCATAAATCTCAACTTTCTA
CsDof83	Csa14g059960.1	AAATGATAAAATGTTATAAGGGAACAAAACTCTACGATAGTATTAGTTACGTCAACAGGTCAATGGTAAAGTTCAACGCGTCGAATCC<br>TGTCAAAGAGAACTATTGGAGGAAATTTCTAAGAAAGTTCGCTTAGTTTTATATTTTTTTTTTGTTATCCATAAAAAAAAAACATAACA<br>AAGTATTTAAATTATATTGAAAATTTTTAAAAGACAATTATTTTATATAAAACAAAATTACGCAAGAGAAAAAGAAAGGTTATTGAAA<br>ATCAACAAAACCATGACTTTTGAGAAACAAAGTAGAAAACTAATAGCAAACTTACAAAATAGAGAAAACAAAGGTCGGCTGCTGTCA<br>CAACAAAGAAGCAAAAAAAAAAAAAAAAAAAAAAAAAANACAACAAGAAATTTTTAAAAATCATGAAAATCTTCAACGAAAAATATTTC<br>ATCTATTTTTTATTTGGATGTTTAATATCTTTTTTAGTTAGAAGAAAAATACATCATTTAATTAAAATGTGCGATTTAATACCTAAACTA<br>ATAAATTTTATTAGTTTCGTACCTAAGATTTTTTGACAAAAATAAAATTTCGTTTTTACTCTTACTGTATCAATTAATAATTAATTTTAA<br>ATGATATGATATTAATTTATAAGTTTATTTGATTTTAAATTAATTTTATTTAATATTTATTTTTATTAAAATTTTGTTTTACAAAAAAAT<br>ATAGTCATTTTATCAAACCCTAATTCCTGTTTATTCTTTTCGCTCGCCGTCACGGACTCACGAACTCACGGCCACCACCTCCTTGGCAC<br>AACCACCACATGTTTCATCCTCAACATCGCTTAGTACAACAGCAACCTATTCTAAAAAGACATCTACACTGCAATCGGATGGATTGATC<br>GCCGCTGCCGAAACGATGAACACTATCTACGAAGTCTTCACCGTCTCCAAAGCTTCAACACTTATCGCTCTCTGCAGCATCGTTCCCGC<br>TAAGACAAGGCTGAGATCGACTTGTCATGGATATCTGCGGCGGCTGGTAAGGCAGGTGCGATTGTTCGAGCTTTCGGGCGCAATGCTA<br>GTTATCCTCCCGAGATTGGAGTTAGGAACTCGTTGTTGATGCTTGGTGCTATTACATTTTATGGGGATTGTTTTCACTGTGTTGGTTCC<br>TGAATCTAAAAGGAAGTTCTTGAAGAGATATTGGGAGAAGAAGATGAGGAACATATAGGCGGGGATGCGGCGGAGATGACTTTTCCG<br>ATTTGATTTTTTTTTTTTTAATAACTCAATATATTCATAAAATCTGTTGGTCTGTTGTTGATGGCATTGATCAATTGTACAAACTTATCTA<br>ATCATGAATTCATATATTCCTATGTTTTTGTTTAATGCACGATTCTGAAGAAAGAAAGCAGATTTGAAAGTGCTTCAGATCTTATAAAT<br>TAATTGTTAATTGTTTAAATAAAATTTGTATTTTGTAAAAACAAAATTGTAATAAAAATAAATATTAAATAAAATTAATTTTAAATCAA<br>ATAAACTTATATATTAATGTCATATTATTTTAAAATTAATTATTAATTGATATAGATATGAAACTAACAAAATTTTTTAGTTTAGATATT<br>AAATCATAAGTTTTAATTAAATGATGTATTTTTCTTCCAATTAAAAAAAGACCCAAAACTAGATGACACAATGCAGGATAGATAGATG<br>AAGTATTTTTAAGCTTTTTTCCCAAATCTTCAATGACACAAAACTCTCTACCCAAAAATAAAATAAAAAAATCAAGAAAGTGTTGAAA<br>AATTGTCATTTTGTTGGGTAAAATAAAAGAAGGGAAAACTCGTAGTTACCCCCACCACCAAATAGCTCCACTCTTTATCTCTCTTCTCT<br>CTATATACTTACAACTTCTTCCCCCTCACTCTCCCCCAAATTCAAGAATCCATTTTTTTTTAAAGGTCTCTCTCTCTCTCTGTACACAAC<br>CCAAAACAAAACATATTCTTGTAAATTAAGTCAGAGAAAAAAAAAAA
CsDof84	Csa15g031650.1	CCAGAAAAGGGAATCCGATTATAAGCAAAATCGTGATTGTAGCTTTCTCTCTTACATGACTTTTGAGAGTTGTTTATTTTGGAATATTC<br>TTGAAAACTATTTAAAGATTAGTACTATATCTCCATGTTTTATTTGTTAGTTTTGGGTAAGAACTCTTTTCTCATATACATGCTTTCACC<br>ATGTTTCACGTTAAAGAACGAGTAACAAAATATTTGCTTTGTTTTTGATAATTAAACAAGTGTCTCGTAGCTAAGCTTTTATGCTCCAA<br>TACGATTTGGTTGTGAAAAAAAAAAATTCTCAAGGGGCCACATGAAATCTAAGCCGTCCATGCGTCGAAGCGCAGCCACAAATCGTAGC<br>CGTAGAAATGACGCGATGATGGGAATGACAAAGGAAAGTGGAGTGTTAGTCCCCACGTGTTGGGAAAACAGAACACACGAACGCCAA<br>ATGGTCAGCTCTTACGGCAGCTTAACGCGACGACAATATCCTTAATTCTGTATCCATCGACACACGGACGGCTCCGATTCTATTCCAAA<br>TCTCGACGGCCACGATTCAAATATTCGAGTGTAATGATCTATCTCATCGCCATCGAGTGCACACATACATACTTGCTTCACAAGAACGC<br>TAACTACTATTTGTCTACACTTATTCTTGAGCCAGAGTGGGTTGTGGAAATGATGAACTCGATCGGTGCTTTTTTTTATATAATTATTTT<br>CAATCTTTTTAGCTAGCTAATTATGTAATAAACCACTAGATGATGCTTATTTAATGCAGATTTGATTATGTATAAAGAAGACTAGTTGA<br>TTACTTCTAATATTTCTTATTTGATTAAAAACAAAAATGGAAGTCTACGTGTAGGGCTTGTGCATGGATAAGAAAAACTCATGGGAAA<br>TGGGCGGGGCTTTGGGAGAGAACAACTCTTTTCTGACGTTTTTGGTTGTCTTTTGTTTGACGTCTTCTTTGGTTAGGTTTTTTGATTAA<br>GAATTTTTTTTTTTTTTGTCGGCATGTTTATATAATTAAAAATTAAAATGGGAATTCTCTTCTAAGCAAAATATTTGAACGTATGTAAA<br>AATGTTACTATTTGTTAGAATGTGGTCATTTGCTCCTTCCGTACGTGATTTAATTTCCCTTGCACAACTTTGTTGCTGATAGAAAACACT<br>TTGACAAACCAACATTTTGATCTCTTATGGATTTTATTTCATCAGTTTAATTACTATAGAAGTTAATTGAGAGTCACTAGCTAGTGAGA<br>CTTTTGTTCTTGAAAGAAAAAAGGTAATTGTGTTTGTATTCGGGTTAAAGTAAAGTACAAATAATAGAAAACAAAAAGATACGTAGAA<br>TCGATATTTGTCTAAACATAAGAAGCCAAACAAAACCATCTTGGCACAAAGACAGGCGTATCCAAATATGTTGGAAAAAGTTATTACC<br>CCTCGATTTGTTCAAAGTTTTTTTTTTTAACCATAATGCTCACAACTTTGATTTTTCTACATTTGTAAAAGCTAATCGATCTTACTAACT<br>CAAACAATTCTTCGAATTAATTAGACATATAAAAGTAAATGACATCAAACAAAACAACTCTATGATAATAGTAGTTAAAGAAGTCAAC<br>AAGTGAATTGAAAAAAAAAAATTAACACCATCCTCTTGTCTTCTAATTACAAAAATTAATAATCAATTAACATAGATAATAGTAATACAT<br>AAAACTTAAAAAAAAAAAGAAAGAAGGAAGATTACGTAGGATAGCATTAATCTTGTAATTTTGGGTTGCCCTATAAGACTGGTCACAC<br>ACACCACAGCTCACAAACTTGTCTTGTCGTTTAGACTTTAACCCAACACTCTCTTCCTCTTTCTTCCTTCTTCTTCTTCTTCTCTCTCTCT<br>CTCTCAAATTTTTTGTCTTATTTATGCGATAAGAACCACAATTAATTCCTCTATCAAAATCTTTTTTGTTTTCTTGATTATTTGATAAATT<br>TTTCAAGAAACACGAAAAGATCATCAAAGAAGAGAAGGAAAA

Name	Gene ID	Promoter sequence
CsDof85	Csa16g003010.1	GATTACCATTTATAAACGAAAAAGCTATATATATAACATAGTATAAGTCGTGCAGTATTGTTTGTTTGATGAAAAGTTTCTTTGGTTTC<br>TTATAATTGGCCTCGATTTTCAGTTTATGTGCTTTTACAAAGTTTTTTATTTTAGTTATCTGATTAACGCGACATTTGTTGTGTAGCTTT<br>GCCGATTTAAGAATAAAATCGTATTAACTTTATGAAGCCATGTGTGGTGTCATGTATAGTGGTCCTTTCACGAAGAAAAAAGCAGATC<br>ACAATTCAAATAAAGGGTGAAAATTTTGGTGTACCTTTTTCTTCGTCGAAACAAAATAATTAAAATAAGAGAAAGCATTATTTGCGTC<br>CGAAGAATACTCCATATTCCTGTAAATACAGTCAACATATATATTGTACTTTATATAAAGTTAGAAATAAAAAACGTTATATGTTTTAT<br>CGACCATATTAAGTCACAACTTTATCATAGATTAATCAGTAATTAGAATTTCAAGTTCACCACCGAATTTCGTAACCTAATCTACATAT<br>AAAAGATAAAATATCGAGTAATTATGATATCTATGTGGATCTTGAAATTGCCAAGGTATGACGGATCGTAACCCAACATATAATATTA<br>ATTACAAAATGGGTGGGCGGGAATAGTATACAACTCATAATTCCACTCACATTTTGTATTATTATGATATGAAAATAAATCAACATGC<br>ATAAGATAATATATTCATCTGTATATTATCGTATCAAAGATTTATTTGAAAATATATACAGTATCATCTTTGAAAACATAATTTACACA<br>CATGGTTTAGTCTTTAGTGACACATTTCAGTCGCAAATCATGAAATGACAAGTGATTCAAACAAATTTTAAACACCACTATGCGTATAA<br>CACTGTAATTGAGCCAACCAATTCGTTAAATAGTGTAATCAATATTTTAGCCAGATATTTTTTTCCTTTAAAAATATATAAAAGAGGGA<br>CCAGAAAAGAACCAGAGAGGGAGGCCATGAGACATTATTATCACTAGTCAAAAACAACAAACCCTCCTTTTGCTTTTTTCATGTAAAT<br>TATTATTATATTTCAATTTTGCAGGTTTCTTCTCTTCTTCTTCTTCTTCTTCATCTTGGCTGCTTTCTTTCATCATCCATAAAGTGAAAGC<br>TAACGCATAGAGCCATATCGTCCCAAAAAAAGCAAAAGTCCAAAAGAAAAACCATCTCCAAAAACATTCTCTCTTTGTTCTTTACTAG<br>CTCTTTAGTTTCTCTTTTCTCTCTCTCTCTGCCTTTCTCTTTGTCGAAGCTCATGGATGCTACGAAATGGACGCAGGTACGTAAAAAGG<br>ATCTCTCTTAATTGCTATATCTGTTTGTTTCTTCTCTCTAACTCTTACTTCTCTCTCTCTCTATCTCTCTCTTTATATCTCTACGCACATAT<br>AAATATATACATGTTTGTATATGCATGTATGTATGATGCAAGTTTTTGTTATGATGCGTCTTGGTTTATATATATATATATATACAGTTT<br>TATATCTAGATCATGTTTATATATGATGGGTTTTAATTTGGACTTTTATATATGAAAAATGCATGGGGAATTTTAATTTCCGTTTGAAA<br>TTTTCCCAAGTTAAGGGACTTTTGTTCTTTATAAGTTCACCTTTTCATGTGACTAATTTTATAAATTCTGATAACTTATGGATAAAAAGG<br>TATGATTTGTTAGTTACCTTTTGTTCTTGGTGCTCTGGTCATAACATTTTTTTCTTAAATTGTAATTTAATTAGTCTCCTTTTCTTGAATT<br>ATCTTTTCTTTTTTAACGTACCAATTATTTAACCTTTTGAAAACCTAGTATACATATATATATATACATGTATATTTATGTTCGTTTGAG<br>TATATTAGTGCTTCAATTTAATTGATCTCATGGCGAGAATCAAGACTCCGTTTTGCTTGTGGTCTATAGTTTTGGCATGGTTAGTGATT<br>AATTAAGGTCTATCTTAATTTTTAGGGATTTCAAGAA
CsDof86	Csa16g007040.1	AGTTCATCAACTGTAAAGATAGGAGGATAATTCAATCCAACGGTCTCAAAATTATAAAAATAATTAAAATATTAGTATATACAGTATG<br>AGACAAAGTATACTACTACATACACACAAGTTCATCCAGTTTTTGAAAAGAAAATATATATGGGGCAAGACTCCTAGGTGTCTAGAGA<br>GTTGCACATTTAATTTCATCTTCAAACCTAATAATAATAGATTATTGTTAAAGTGTTAGTAAAGTGGAACTCGTAACTCGTGCACTGTG<br>AATTCATTATCAAATCCGTAAATTATTAATGCAAAAGTCTTACCAACACATATATTTGCTTCGCTTTATAACACTTTTGTTATTTACTGC<br>CTTTTTTGTTTCTGATGAAAGCTTGTATATTGAAGGAAAAAATACATGTAGCTAACAAAAACTAGGATCAACTATATTTTCGTTAAGGC<br>AGAAATTCTAACTTAGTTGATAATCTATGAAAACTAGGTAATAACCCACACTACATACGGGATAAATAGACTCAATAAAATTATTACA<br>ATTAAAATTATTATTTGGAATTTAAGGTGCCATAATAATAATTTTACACATAATTTTTTTTATTCAAATTTACATTTAATTATTTTGTGT<br>AAAAATATATTTCACCCGTGAAATGTGGAATATAATGTTAATTATTGTGATAATTGTGATAATGTTATTTATTTTTAAGATTATTGTAA<br>TAATTAATTTTTAATATATACTCATTTTGTTTCATAAAGAGTGTCATTTTAGAATTTTATTTTTGTTTCACAAAAGTGTCATTTTACATTT<br>TCAATGTACTATTTTATATTTTCAATGCATATTTTTCATTAGATTTTCTAGTTTTATCCTTAATCTATGACTAAATTAACCCATTAAACA<br>TTTATTAAATAAGGGCATATATGTATATTTTAATATTTTTTTAATTTGTATGAAATGTGTCAAAATGACACTTATTGTGAAACAGATAG<br>AGTACTTGAATAGTCATCAAATTTTTAAAAATCGGAGAATAAGTTTTGTATTTCAAAAAGTTAAATAGATGAATAAGTTTTTTAATAAT<br>TTATGAAATATAATTTACTTTAAATTATTAAATATGTTATGAAAATGATAATTAAATTTTTAACTATATATATACTTTTTAATAGTTGAC<br>GAAGTCTTTTATTTTAAAAAACTAATATAAAGAAACATTACCTTATATATTCTAATAATACATTTATGTAAATAATAAGAAAATAAAGA<br>TTTCCTTATATATTTTAATGATACATTTCATAAATCAAATATTTTTGTAAATATATTTATCTTGAAATGGTTAATGGTAATTAAATCTTC<br>AAATCGACGATTTTTACAAAATGCTTTCCTTCAAATCAGTATTTTCACAAAATGCTTTTTTATTAATATATAATATTAATATATAAGGG<br>ATGTGAATAGATGGATCATGGATATAGAATCTCTTTCAAATAAACTGAGATTACTTTAAGTTGGATCACGTTCAAGCATCCACTAGTCT<br>AAACATTGTTACGTAATAATTGTAATTTTATATTCGTTTTAATAGGAGCAAAGTGGACAAAAAAGGGAAAACGTGTTGATAAGACTTC<br>TGCGCTCTATACTACTTTACGGACTCGATGATGGTTTTACAGTAAACTACTTTACTTACCAATCCCTCTATTTTTTTTTTCCAAATAAAT<br>ACTAGCTTTTTAAAGAAAGTTTTGTAGCTTGATCTGCACTGGTATTCGTGTTTTACGTGTAGATGTGAATTCTAGACCCAAAAGTAAAA<br>TAAAATAAAGCGATGAATGCGCCTAATACTATATGCTTTTCAACTTTATCCAGCAAAATATTTTATTTAATCTCCCACTACTTCAGTCG<br>CTTTTCATCTCCTTTTCTTTCTCCATATTTGCTAAAAAGTTATACTATCATCTTCCTATAAAAAGACGTCTTCTCCTATTTCTTACCTGCA<br>ACATACTTTTGATAAGCTAATCTTTCCACTCTCTTC
CsDof87	Csa16g016240.1	GTCTAGAAAAATATGAGGTATTTATTTGGGGGCACGGATATGTAATTTGCTACAACATCAAACTAAAGTATACAATTATATTCAATGT<br>TCTAAAATACGGACTATTCGGCCATATATACGGTGAGTATACGGTAAACGGTGACCCACTGTACCGTTTATAGTCTATTCGTTTTAATA<br>TATGCGGGTTGGTCTAATTCGCTTATTTATACGGCGTATATACGATCGATTATACGGTTAGCTCATAGACGTCCGTATTTTGTTTTTTTT<br>AAACCTTCATTCAAGATATAAAACGAAACCTATAAATTGAAGAAACCCTAAGCATTATTCGTTTTCTACTGCCGTGACACTTTTCTCAA<br>ACTTCATTCTATATTTAGAGAGTCTTCAGGTTTTCTTTACTCCTCTTCTCTTGTTTTGATTATATGATCTTTTTCTTTTTTGCTTTATCAT<br>GTCTATAGTATCGTTGTTGTCTCGTTGATTATAGAACTATAATACTTAACATCGTTAAAATAAGTTTGAGTTTTGAATTTTGATACTAC<br>AAGTCGACGGGGAAGAAGAACTTGAAGATTAAAATTGGTTTATTTTTCTGTTTACGTTTTGCTATTTCAATTATTGAGTTTTAGACTAC<br>TTGGTTTGACAATTTCATAAGTATTTTTGACAATTTAAGGTTGATATACTATTGGTTTGACAATTTCATGAGTATTTTTGCAGAGATTTT<br>AATATTTATTTTGTTCTTCTATTATATATATATATATATTTTTTATATAATATATTTATAATTATTCATATCTATATATATATTTTTGGTAAA<br>ATATATTTACCGTATATATCCCATATATACAGCCGCGTGTACAACTATACGTTAAACGGTGGTGCACCGTCCCACTAGCGCCTAGCGTA<br>TTTTAGAACATTGGTTATATTAAATAATAACCTGAAAATCTAGTAACAATAATAAGACTTTTCTTTAATTTTATTTTGTTATAAAGGCTT<br>ATAATTTTTTTTTCCTGCTAACTGAATTATTATTATTTTGTATAATTTTGTAAACATAAATTCTGACTGATACAAATATATAAGTGCAAC<br>CAAAAAGATAAGTACGAATAATATTTAAATATAGTGTGTGTTATTGATATAAAAGCAATACTATTTATGCAGTATTGACAAATTCAGA<br>ATAACCAGACTCCCAACATTTTAAAATTCTGAACTACATAAAGCACTAGACTATAGATCTAGTGACTGATATATCTAGTGACTGGATA<br>GACCAAAAAGATGATTTGCATATGGTGAAAAAATGTAATTAAAAATTGTTTGATTGTACAACTTTTCGCAAGTCCTTCTTATCACTAAA<br>CCAGTTTATCTGAACATTTCCCTAACTTATTTTACTTCGTGGTGAAGGTAAAACTGATATGATTGTAGTATTTTATTATTGATAAATCG<br>CTTAAAGCAAATCAAGGTCTTTTGGTTGTCTCTACTCTCTAGAACGTCTTTAATTTATTTATTAGACCTTGTAAATCATATGGAAAATG<br>TACATAAAATATATACACACATACAGTATATATAATATTTATATTTTAGATATAATTCGACAGAGAGAACCTTATAGGCGAAGAAATG<br>TCACTCCACTCAAACCCTAAAAAGAGAATCACAACAAAATCAATAATATATATATATATATATATATATATATATATACACACAAATAAA<br>CAATGCATGAGAGTGGGAAATATATATAAAAAGAAATAAAGCGTGAGAACATGACCCATGTTTAGAAAAGCATTATGTTCTTACTTTA<br>TCTTTGTGCTCATTCTCTATCATCATCTTCTCTCTAAACCTCTACTCTTCTCTCTTTCCCTTTTGGTTCTGTGCTCTCCCTCACATCTTCTT<br>TAGCCTTTGGTTCCCTTCTTTCGTTTTTATTTTGATTCTCCCACCATTTTTGTTCTTTTGAGTTTTAAAGATCCATCAACTTTATTAAAG<br>AAAGAAGAACAAAGAGATAAAAGGGAAGAAGAACA

Name	Gene ID	Promoter sequence
CsDof88	Csa16g016640.1	TTGATTTAATATTTTGTAATCTCCTGAGAAATTCCATGGATCTAGCTTTTTGATCTTCTGAATTTACAACAGTTTTATTTAATATNTGAT<br>CTGAACTCCTTCGAGAGAAGCATTCCTGAACTCCCTCATTTACTTTTTAATATCTATTGCATGTTATTCAGAATTATGTCTTGTTCATCA<br>TTAAACATGTCTGAGTAGTTTGTTTGTTAGGTTCAGGGTTTTTCACAGGGATTTTATTAATTATTAGATCTGTTTATTTAGGATTCTTTA<br>TCTTTCTAAATCTTCATCATAGGTTGTTCTTAATGCTAGATCTTTGATTAGCCATCTGGATTTAGATCTTAGGATTATTGATTGATATGA<br>GAATATAATTGATTTTCCTGATAAAACCTTTTGATGAGCAAAATTACTACTTGCAAAGACATTTGATTTAGTAATTTTGTGAACAATCT<br>AAACTTGTTTTTAAAGCTGATTTTTAACCTAGAATTTTGCAAAGAGATTTGGATTTTCTGGTTTTAAATTTAGATAATATTTGCAGTGA<br>AAACTGATTTATTTTACAAAAGTGTTTAGAGTTCTAGATCTGATTTTAATTGCTTAAATTTTATTTAATTATTGATTTAATATTTTGTAA<br>TCTCCTGAGAAATTCCATGGATCTAGCTTTTTGATCTTCTGAATTTACAACAGTTTTATTTAATATTTTGCATTGCCTTTTTAATTTGAA<br>TCAACTTGTTTAACTTAATTATAAAACTCTATAATTTATTGTGTAGACTTGAGTCCTTATGGAAATCGACCTCTAAGTATTACAATGAC<br>CTTTTAATTTGAGAGAGTAGCTCTATGATTTAATTTGAACATATCAACTTCACCAGATCCGAGCAAGAACCCAACAGTTCTGGTTCCTT<br>CTGTCAAAGATTGCAAGAGCAAGTCTTGGGGATTCAATTCATTTCCATTAATATTCTTTTTTAGATATTTGTAGCATCGCTATTGTCTA<br>CTTCCTTTCTTTTTATTTTTACGTATTCTAAGATCTTTTTTGGTTAAACAGATAATTTATCAAAATAAATGTCTTAGAAAACAAGAAAAA<br>GAAAATAAGAAGAAGACTCCTTACGGTTCTAGTTTAAATATATGTAACAAACAAAGTTAAAATTATAAGTTTGCGCTTTGCACAACAA<br>AAAGAAATGTTTATCGTCAAAAGTTTAAGTTAACATATAAGTTGAAGACTTTTGTATAACATTTCCCGACTTACATGTGAATATCAAAA<br>TAATTATTAAAAAAAAAAACAAGTAGCTACAATTAAGAAATAATTTAAATTATGAAAAAAACCATATATTTTAAATTAAAGGACAGACT<br>TTATCTGTTTTGCTTTATATATTAAGAACCATGCTCTTCTTCTCTATTCATCTCTTGCTCACCACTTTTCCCCAAACTCTCTTTCTTTCTC<br>TCTCTCTCTCTAGTTTCCTTGTTCCTTTCGAGTTTGAGCAAAAATATCAACCATAATTGCTTATTATTACTACATAACTACGTAATCGTT<br>GCTGACTTGCTTCTATCCTTTGTTGCATGGATGGATCCTCAACAGGTAATTCATTGTTCTATAATTTGTAAAAACTTTAAACACTTTATT<br>TGTTTACAATTTGAGCTCTCTTAGTTCTTAATTCCATCTACCGAAGTAAAAAACACTTAAAACCCTTTGAGAATTTTTTAAAACTTCTGC<br>TCAATCAAGAACTATCTATGTTACCAATTTGATGTTGTGTTAACTTGATTAGCTTACGCTTACCTCAAACCCCTTTTTTCAAGAAAAAT<br>AAAAACGTCTTCTTGTAGACAAGATGAGTTTACAAAGAATTATTGTCGGTAAAAGAATCGACGCCACGTACGGCTAATCCGTTATATA<br>AAGTAACCGTCATGATTTAACAATTTAATGAAATTTGTTTGATTGTGCAGGAAATCTCAAACGAGACTTTGGAAACCATATTGGTAAG<br>CTCATCAAAAGGAAACAACAATAACAATAAGAAA
CsDof89	Csa16g031000.1	GGCCAATGAACTAGATCGTTATAGATGATGATGCCCAAGTTTTATCCCATTAGTTAACAAAGGTTTTAGAGTCCACAATTCCTCGGTTG<br>TAATTGTAAAAGAACACGGTTTTAGCAAACAATTAATAGAGATTTTAAGATTTATATGATAATTATTATTTACTACATGCAATCGATTA<br>CGAGTGGTTATTGGTTCTATGATTTTAATAAATTTGGAAATCCAAACAAAAATCATGTGTTATTCAATCATTGATTTTAAAATACTTAT<br>CAAAATTAGGTGTTATTGGTTATATGACTTACAAATACTTGTTAAAATCTCATGTTATTCATTTAATGATTTGTTTTGGATTTCAAAATC<br>CACTATATGTTTTAAATGGATTTGAATGGATTTGAATGGATTTAAAAGTGTACAATAGAAGAGTTCAAATCCAATGATAAAACATGTT<br>ATTTCAAAATCCATGTATTTTGATTTCTAGAATTTACAATTTCATATGGATTTAGAAATCACATAGTTATCATAACCTCTCCAATAGTTA<br>TGATAGCTATATGATAATGAAATGTGTGTAGTATATGATAATTTTCAAAATATTTTCGTCACTCAGACATACGTCATAGGTTTTATAAT<br>TTCTAAAATATCATTTAATGTTTAAAAATAAAGTTGTATAGTACATACTCTTTTTCACAATAGGAACAATGAACCTTGGCATTCATAAA<br>ATGAGTTTAGGCTTTCATTATGTCAACCTTAGTCACAGCAACTATGCGAATATAGTGGACAGTAAGTAGTAAGTAGGATTATAATTGC<br>AAAATACGAAAACAAATGTTATAATAAGAAAAAGAGAAAAGAAAAAAAAAAACACGATCGGAAAAGACTTAATGGGATAAGAGAAA<br>AAGATATGGTCCAGCCTTATCTCATACCTGGAAATGCCACGTGTCATACACTACCAGAACATAGCCTCTCTTACCACCACAAACTTTTT<br>TTTTAATTCTTTTTGATTACTTTCTTTCCCATCATTAACTAAAGAACACAACGAACGATGCTAGCTAGCTATGTTTGGATTTGGATACGC<br>TCACACTACTATATATGAATAACCCTACAAGAGAATATAAATATGTTCCTCTATCCACGTGTCGGATAGGCATTGGTCACATGCATGAC<br>GAACAGCTAGATTCTGGCAGATGAACTTTTTTAAGAGTCCTGATGGACCAATCCACACGTGCTTTAATTACAGCTGACGTGGGGCTTT<br>TTATGTAGGATGTTTCGGAGACGAAGTGGGTTATGTGCCACGTGGTTACACAACCTGGCATTGACAGTTTATGCCTTGTCGGATTACT<br>TATTGTAACTTGTAAGATATGTTGTACCTAGAGAGAGAGAGAGAGAGAGAGAGAGAGAGAGAGAGAGAGATAGATGGATACCTAATTCT<br>GTATTAACACCCGATTAGTAACTTTTACCATGATTAAAAAATCGCAACCAGAGAAATTCGAGCACTTGTTTCTTAACTCTGTGTTAAAT<br>CTTTCAACCGTTGAACTATGAAGTTAGTGTTTCAAAATTTTATGTACATACGTCAACGAAAGTAATGTAACAAGAAGTTTCGATTGGTA<br>ATATGAACCGAACTTGAAAACCCTTCTTTTCCTCAAATGTTTTTGTTTATTAGTATGACTTTCATGTTACGTACATCTGACTTTTTTTTT<br>ATCATCCGTTTTGGGTACGTTACGTTAACACAATTTTGAACAAGCAGTAAGTACGTAACTAGATAATAATATTTTCATTCTTATAAAAA<br>TGCTTTTTGTAATAAACCAGAGGACACTACGAACCAAGTGTTTTTTTTTTCCCTCTAAATATTCCTTATAATATCCTATGTGGCAACAA<br>AGAGAAAGGTGAGAAAATCTTTTAGAACGCCACTAGAAGAGGGAATCAAAAAGAGCGTGAAAAAGATCTCAAAATGCCAACTTCCTT<br>AATAAAACATGTGGCTCTCACATTTTCACAAGTCTCTCTCTCTCTCT
CsDof90	Csa17g011020.1	NNNNNNNNNNNNNNNNNNNNNNNNNNNNNNNNNNNNNNNNNNNNNNNNNNNNNNNNNNNNNNNNNNNNNNNNNNNNNNNNNNNNN<br>NNNNNNNNNNNNNNNNNNNNNNNNNNNNNNNNNNNNNNNNNNNNNNNNNNNNNNNNNNNNNNNNNNNNNNNNNNNNNNNNNNNNN<br>NNNNNNNNNNNNNNNNNNNNNNNNNNNNNNNNNNNNNNNNNNNNNNNNNNNNNNNNNNNNNNNNNNNNNNNNNNNNNNNNNNNNN<br>NNNNNNNNNNNNNNNNNNNNNNNNNNNNNNNNNNNNNNNNNNNNNNNNNNNNNNNNNNNNNNNNNNNNNNNNNNNNNNNNNNNNN<br>NNNNNNNNNNNNNNNNNNNNNNNNNNNNNNNNNNNNNNNNNNNNNNNNNNNNNNNNNNNNNNNNNNNNNNNNNNNNNNNNNNNNN<br>NNNNNNNNNNNNNNNNNNNNNNNNNNNNNNNNNNNNNNNNNNNNNNNNNNNNNNNNNNNNNNNNNNNNNNNNNNNNNNNNNNNNN<br>NNNNNNNNNNNNNNNNNNNNNNNNNNNNNNNNNNNNNNNNNNNNNNNNNNNNNNNNNNNNNNNNNNNNNNNNNNNNNNNNNNNNN<br>NNNNNNNNNNNNNNNNNNNNNNNNNNATAAAAGTTGTAATTAATCGGAAACGAGCGACTCTTTCTTCTCTTTAAAAACCAAACTTCATT<br>ATCTACTAGAAGAAGTAAGACCGGCGGTCATGTTGGATTGTCTTCTAGTACTCTTGAGTTTTCATAAAATTATATAAATGGGGAAAAA<br>ATATTGAAATAATTACGGAAAACACATGTTATTTATCTAGCTTTTACGCTTATTTATGCTACTGAATGCAAATTTATTTAATTTGCATA<br>ATAGACAAGATCCCACCAAAAGTGTAATAGGGTATGATGATGATATACTTTATTTATGGCATATGGGGCTCGCCATCTACATTATAAA<br>GAATAATTGTAGAAAATAATTTATTTTCGTAACTTAATTCCCCCAGTATAATACATCCATCTTTTTCATTAGGCACTAATTCGTTTATAT<br>TTGGCTAATAACAAAAATGTCTCTATAGAGAGGCACGTACGTAAGTGAGAAAAAGAGAAAGATAGGGAAGAGAATAGTTGAGTGTTC<br>GGCGTTGGTGAGCTTCCATTATAATGAATAAAACGGCGCAGAATTGGGATTAAGATGATCAGAGTTATATGAGTTTAGTTTTTAAAGA<br>TTTCCGAGTTTTGATGAAATCTATGACAGGCCAGCGATTACTGTTGATGTTAGTATAAATTTTTCTGATTTGCATGGTTTATATATATA<br>TATAATTACGAAAAAAAATTCGACTATGAAAAAAATGAGAAAACCAACAATACTATTGTAGGATAAACACGTAAATGTTTCTAAAAGTT<br>GTAAAAGTACCTCGAAATATTTTTTAAAAAATTATATTAGAAAATTTAGTTAAAAAATTGTTATATTACATATTTATTTTACAAAATAT<br>ATATTTAACGTTTCTTCAGATTAAAAAAAGTTATACAACTTTGTATAGTTTCGTCGAACGTTTCTTCTATATATATTATTCGATAGACGC<br>CTAGGAGAGGAACCTTATAGGCGAAGGAAAACGTCCCCACTCAAACCCTAAAAATGGGAATCAGAAATTAAAATAACACATAAATGA<br>AGTATAGAGACGCGTATACACATGAGAGTATGAGAGTGGAATTATAGAAATATATAGAAAACATAAAAAGAACCGAAATCAAACTCC<br>TTTTCTTTTTTTCTCTCGTTAATTAAAAAAGCATTTTTCTTGCCTTTAATTTGTCTTTGTTCTCCTTCTCTCTTCTCACTTTCCTTTCTGAT<br>TTTGTGCTCTCTTAGCGTTTCTCTTTGGTCGTCTTGCTTCCTCTCTTTCGTTTTGCCTTTGTTTGTTTCTTTTTTTTGATTCCCCCACCAAT<br>TTTTTTCCTTGGAGTTCTTTCCCCTTAAAGATCCATCAAACTTTATTAAAGAAGGTAGAAGAAAGAGATAATAAAGGGTAGAAGAACA

Name	Gene ID	Promoter sequence
CsDof91	Csa17g027260.1	AGATAGCGAACCAACATGAAGATCAGTATTCATTGGTTGAATAAAATTCACAAAAGTGACATTTAATGATTAGTCAATGTAGCTAACA AGTAGCAGCGTATTAGGTTTAGTAACAATATCCTACACCCAAAACTCTTATGATAACAGAAGGTTTTCTTTTGTTTTTCGTGAATTTGA TTATTCTAAACTTTTTCTAAAGAAGTAGAGTAAATTATCTAAACCTTTTTCCTTGATATTGAGTTGCATACTTGTAAGTTGTAACAACTT ATATATACATCTTCTTGTTTATATTAACTAAAATATATAATTAAAGTCAAAAGCAATTTCGAGTCAAACAGGAATTAATAATACAGCTG GGAATGAATAAATGCCAATCCGAAAAAAAAAGAAGAATTAAGGTTTGACTTCATATTATTCACAATTTTATAGGTATTTTTAATCATTA GACTACGGATATCGTATCGTAAACACAAAATAATATTGGGATCGGAGCTTACCATAGATCTAGTTTTAAGGCTGCCCAATCAAAACTA ATCGGTGGTGATCAACAATGATAAATTAACAATCAAGTGTTATAATAATGATAAAAGTGCTAAAAATCTATATAAGAAAGAGAATTGG GGGAATAAGAAAGAAAGAGATTTCAACTTCGTAGATGTCAAGGAATTGAGAGAAATAAGAGAACCATAGGTCGACACGAGGGTAGA AGCACGTGTTCTCCCGTGCGTGTGGCCCTTAATCTTGGCGTTAATCTATCTTAATCTTACTATATTATCATGGTTATATAATCTATGCTC TTAACGGTCACTAATCTACATTTGTACTTTAATTCAACAAATAAACATTTTGGCTAGTAATGAAAACCATACTATAATTGACCGAGTAA ATGTTCCCCTCTATTCCAAGGATGATATTACATTATTACTCATCAAACCCTATATGTACAAAAGTAACAAAACCTATCAAACAAGTTTT ATATGTGAATTGTGAAAATATAGTTGAGTTGATCGGACATATATATATATATATATATATATATATATATATAGCTATGAAAGACTATA TATATGCAAGTAACTTGGTGAATGGATAAACAAAATGACAATAATAAGAATAATGTACTGACGCTTCGAACTTTGGAAGTGTTAAAAT GATACTAATGTAGAATGGGTGCAAGTGATCAAAATGTTACGATGCTTTGTTTAATAAATCCAGTAGTGTCTTGCGGTTCACGAGAACT AGATGCTGAGAATGTAAGTGACTAATCATCTACACACTAATATAATATGATGCCCCTACGTTAATATTTTAGAAGAGGGTAATTATAT AGTGTTTTACTTACTTACATTCACGATTAGGTCAAGCAGACAGAAAACAGGGAATGCATCAACCAAAATTTTCTGATGTTTCCTCCAAT AGATATCTATCGACTTGAACTAGTGTGGCATTAAAAAAACTCTTTCTACCCACTTGAACAACCTTGGTTTTAACAATATTTTTATGTAG AGGTTTTTACCACGACTGTTAACTGAGAGAATAGGATGGCACATATGCATATACTTTCATATTATTTTTAATTTGCTCTTTGATGGTAT GAAACATAAATTTCAAGTTGCCTCAACAAAAACTTTCTGAAATTCATCATCCAATCGTTTATCAAAACTTGGATTATTAAATCGTTAAA TAATTGATAAACATATTTAATTGTCATTATATATAGGTAAAAACTAATACGAAGGACCAAATTCGAAGGTAACTTGATGAAGAAGAAA CGGAAAAGGAGACGCACGGCTCGGCAACCGCCCACGTTTACTTCGTAAGCCTTCACACATTAATCGATGCACCTCTGCTTCCACACAC ACATGCTCTTCCTCACCTCTACGTTCACACAATCCCATATATATTAGAGATAGATGTACTCGAATATACCACCAAGTGCTCACTTGTTA GCTTTTCCAATCTTTACACACCAAAATTCGTGAAAAAAAAGTATATGAAAA
CsDof92	Csa17g034060.1	NNNNNNNNNNNNNNNNNNNNNNNNNNNNNNNNNNNNNNNNNNNNNNNNNNNNNNNNNNNNNNNNNNNNNNNNNNNNNNNNNNNNNNNNNNNNN NNNNNNNNNNNNNNNNNNNNNNNNNNNNNNNNNNNNNNNNNNNNNNNNNNNNNNNNNNNNNNNNNNNNNNNNNNNNNNNNNNNNNNNNNNNNN NNNNNNNNNNNNNNNNNNNNNNNNNNNNNNNNNNNNNNNNNNNNNNNNNNNNNNNNNNNNNNNNNNNNNNNNNNNNNNNNNNNNNNNNNNNNN NNNNNNNNNNNNNNNNNNNNNNNNNNNNNNNNNNNNNNNNNNNNNNNNNNNNNNNNNNNNNNNNNNNNNNNNNNNNNNNNNNNNNNNNNNNNN NNNNNNNNNNNNNNNNNNNNNNNNNNNNNNNNNNNNNNNNNNNNNNNNNNNNNNNNNNNNNNNNNNNNNNNNNNNNNNNNNNNNNNNNNNNNN NNNNNNNNNNNNNNNNNNNNNNNNNNNNNNNNNNNNNNNNNNNNNNNNNNNNNNNNNNNNNNNNNNNNNNNNNNNNNNNNNNNNNNNNNNNNN NNNNNNNNNNNNNNNNNNNNNNNNNNNNNNNNNNNNNNNNNNNNNNNNNNNNNNNNNNNNNNNNNNNNNNNNNNNNNNNNNNNNNNNNNNNNN NNNNNNNNNNNNNNNNNNNNNNNNNNNNNNNNNNNNNNNNNNNNNNNNNNNNNNNNNNNNNNNNNNNNNNNNNNNNNNNNNNNNNNNNNNNNN NNNNNNNNNNNNNNNNNNNNNNNNNNNNNNNNNNNNNNNNNNNNNNNNNNNNNNNNNNNNNNNNNNNNNNNNNNNNNNNNNNNNNNNNNNNNN NNNNNNNNNNNNNNNNNNNNNNNNNNNNNNNNNNNNNNNNNNNNNNNNNNNNNNNNNNNNNNNNNNNNNNNNNNNNNNNNNNNNNNNNNNNNN NNNNNNNNNNNNNNNNNNNNNNNNNNNNNNNNNNNNNNNNNNNNNNNNNNNNNNNNNNNNNNNNNNNNNNNNNNNNNNNNNNNNNNNNNNNNN NNNNNNNNNNNNNNNNNNNNNNNNNNNNNNNNNNNNNNNNNNNNNNNNNNNNNNNNNNNNNNNNNNNNNNNNNNNNNNNNNNNNNNNNNNNNN NNNNNNNNNNNNNNNNNNNNNNNNNNNNNNNNNNNNNNNNNNNNNNNNNNNNNNNNNNNNNNNNNNNNNNNNNNNNNNNNNNNNNNNNNNNNN NNNNNNNNNNNNNNNNNNNNNNNNNNNNNNNNNNNNNNNNNNNNNNNNNNNNNNNNNNNNNNNNNNNNNNNNNNNNNNNNNNNNNNNNNNNNN NNNNNNNNNNNNNNNNNNNNNNNNNNNNNNNNNNNNNNNNNNNNNNNNNNNNNNNNNNNNNNNNNNNNNNNNNNNNNNNNNNNNNNNNNNNNN NNNNNNNNNNNNNNNNNNNNNNNNNNNNNNNNNNNNNNNNNNNNNNNNNNNNNNNNNNNNNNNNNNNNNNNNNNNNNNNNNNNNNNNNNNNNN NNNNNNNNNNNNNNNNNNNNNNNNNNNNNNNNNNNNNNNNNNNNNNNNNNNNNNNNNNNNNNNNNNNNNNNNNNNNNNNNNNNNNNNNNNNNN NNNNNNNNNNNNNNNNNNNNNNNNNNNNNNNNNNNNNNNNNNNNNNNNNNNNNNNNNNNNNNNNNNNNNNNNNNNNNNNNNNNNNNNNNNNNN NNNNNNNNNNNNNNNNNNNNNNNNNNNNNNNNNNNNNNNNNNNNNNNNNNNNNNNNNNNNNNNNNNNNNNNNNNNNNNNNNNNNNNNNNNNNN NNNNNNNNNNNNNNNNNNNNNNNNNNNNNNNNNNNNNNNNNNNNNNNNNNNNNNNNNNNNNNNNNNNNNNNNNNNNNNNNNNNNNNNNNNNNN NNNNNNNNNNNNNNNNNNNNNNNNNNNNNNNNNNNNNNNNNNNNNNNNNNNNNNNNNNNNNNNNNNNNNNNNNNNNNNNNNNNNNNNNNNNNN NNNNNNNNNNNNNNNNNNNNNNNNNNNNNNNNNNNNNTTTTTAGCAACAGCATGTGGCTATCTCTTCTCTACATGTCCCTGTCTCTGCTTAC GTGTAATTTTCTCCTCTTTGTGTCTTGTGGCTCTGTGCGTTCCATTATTAATTTAATACCAACCTCAGCCTCTTCTTATACATAGTCTTG TATCTGACCTCAAATAAAGAATAAACACACATAACATCAAATCTAATCTCCTCTGCTTCATCTTTCTTTTTTCTGTCTTT
CsDof93	Csa17g041880.1	GAAAGATGAAATTAACAACAAACTAGGGTTTCTGTTAAACGCTTGTTTTGTTTTGCTTTAGTCAGAGATGGGTTATTTGTTTTGAACTT CTTTAATGGTTAACTTGTGATGTTTTGTAGATGGATGATGATGGTCACGAGGCTAACATGGAGGAGGAAGCTGGATGCTGATGAGCAA AGTCTCAATTTTTTTGGTTAGTTCGGTTTAGAAAAACCGAAATATATTGTATCTCGGTTTAATTCGGATTGACATTTTATTCAGAAAAC CGAACCGATTACCCCGACTACCGTTTAACCCGACCCGAAACTTTGTTCGGAATCATTCGGATCAGTTTTCAGATTACCGATTAAACCCG TAAACCGAATAAGCCGTTCCGAACAAACCGAAATTTCCGAAAATCCAGGGCTAATTATTATTATAAGTTCAACTCTTCCGGATTGAAA ACCCAATTCCATTTATTCACAAGTTCACAATAAAAAAAAAAAAAAAAAGAGAAATTCTCGATTAAATTTCAGTTTTATTATTTTATCCAA AGAAACTCTTATAATTCTAAGAAAAAGAAGAAACTAAAATTTTCCTAACGAAATTTATTTTTCTATACAATACACAATGAAAGAAAGA AAGAAATAAATGAAGAAAATGGAAGGATGACTTGGAAAAACAAGGGAGGCCAAAAAAGATGATGTGTGCCTTGAATTGAAAGCTGA TCCCCTCTAGTCATTCAGGCTGCTTATTTATCTCTTCTCCTTTTCTACGCCAACACACCAAACGTTCTTTCTCTTTGGCGGCTTTGTCCA AATCTCACTTACATAATCTTTGCAATCGCATAAAGTTTTTTTCCTAATTTCTTAAATTAGTTATACTTTGTTAAGATTTTAATATAATTT TCTTGGTCTATGATCTAAACCTTACACTCTAATCTCATATTGAAGAATTGAGGTCGAACCCCTTACACCCGAGCGGTAAAGCAGAAGT GAGAAGTCACCACTCAAGTTGCTCTCTCGACTCATACTTCCAAATATTATATAGATAATATAATTAGTCAACGATGAAAATAAGAAGA AATAGATGATGCTGTTGCGAATTTTAAAAGGATAAATAGTATATCATATAGATAACATAATTAGGTTTTGAATGTGATATTTGTTTTGT TTCTGTGACACAACCCCAATTCAGAAAATATTAAGAACGTACGTACCCCTTCCAAATCACGTTGTCGCACAAAAGTATCGATTTAAAT ATATTCAATAATTAATATCAAACCATACATTTTGTGAATTAGTTAGTTATATAAATAAATATTTAGGTAGCTTGTGAATATTAATTATA GTATAGTTTTTGGCAGTGTTTAGTTATTGTTTTGTATTTCTAAATTGCTTGTTCGCTGCTATTGTACCAAAGCTTTAACTCGTTTGATGC AACGAAATAAAGCTCATTCTCTGCATATGAAATAATGTTTTGAACTTTTGCAAGTTTAGATAACAACTAAAAACAACATCAATTATTAG TAGTGACTTAGAAATAGTATTACCAAAGGGAGTCAATTCAAAACCAAATATAATTCACATAAATTCTGATAAATAAAAGTGTTAAAAC ATCACTATTCCAAAATACCTTTTATAAAATATACGGAAAGACTATGAAGACTGTACTCAAAAAGTTTGAAGATACACACATTATATTAT GGTGAAAAAGCAAAAGAATACAACACATTTTGTCAGCTGATATATGGATCGATATTTCCTTGAATGCAATAAGCAGTAAATGCGCCTA ACATATTCTTTCATAATATTTTTATATTCTCCCTACTAACACTATTGCTTTTCTCTCCTTTCCTACGAAAAAGATATAATAATGTTTTCAT TTTCATACAAATCTTTTTTACAAAAAAGATTGAAATCTTCTTCCTATAATAAACGTCTCTTCTTCTCTCTTTCATCACTGCAAGAAACTT AAATCACAACTCTCTCTTCTCTCTCCCTTACTCTCTCTCTCATTTTA

Name	Gene ID	Promoter sequence
CsDof94	Csa17g073130.1	TCTTAGCTTAGCTACAAGTAAAAAATACCTGGTATGGGTCAAATACTTCAGTCTCAGATACTTGCAGAAAAGTTGCCAAACAAACAAG<br>CTGCCATGAAAAAACAACCCAATGAGCTTAGAATTAAGGTATAAGCTTAGCTAAAGAGATTCTCCCAAAGATTGTTATAGTTGACTAA<br>CCTTAACCAAAGCTGTAGCAAGGTAAACAACAGCAGCTTTCACGGAGGTTCCAAGTGTGTCATATTCAGATCTGCAGATTCACAACTT<br>TCCGATTACAAACTATACCAAATTCCAAATTTGGGGATTCTCAACACCTAAGTTCTGCAGAAGCTCATACTAAGCCTTAAGATTCTAGA<br>TCACGAAGTTTAGAAGTAAGCGATTAAGTATATGCGCAAGAGGATCTAGATAAAATCTTAGATTAGGCATGAATGAACTAAGAGATA<br>CTAAAATTTGAAACCACAGATCGAGAAAAAAAAAAAAGAGGATTTGTTCATACAAAGGAGTGGCGGAGTAGTAGACGGCGTGAGGAC<br>CGAATGTGAGAATCGCACAGTTGAAGAAGTGAAAAAGCGTCATCGTCTCTCTCTCGCTGTGACACCAAATCGGGAGCTACACAAGTCC<br>GGCGACGCAAAGACGACGACCACCACGACGTGAAGCTGTGAATTGAAGAAGAAGAGGTTTAGTTTAGCTGAGAAAATCTGAAAATCT<br>GATGGGATCAACAAAAGTCTTAATGGGTTCCGAATTGGGTCTCTCTATTGGGCCGTATAATTTATGAAAGTCATTATATAATTTTTTTT<br>TTTGTCAACAGCTATAGTGAGCTCAAACTGCTATAGTGTCATTATATAATGATTTTGTTTTTTTAGCTGAAAATTATATGATGTTTATA<br>AATTCGTATTTGATTTCTTTGGGGTTTAAATTCTCTTTGAATTTGTTTTCTTCTGAATTATAATTTCATACGAAAGCTTATGCATTTTCC<br>CATATTTGATATATTCCTCTATTTTTTTATTATAATATTTTCTCACAAATTTAACTATAGTTTGAGCTATAAAAAAAATATCTTAGTCCA<br>TGGTAGTATTGACTAGGTTATAATCTTATTAACGCCTAAACCCGTTTTAGGACCTGATTGGTGAAGGCTTTTAAGACTTTAAAAATATT<br>TAAAAGTCTTAAAAGCATTTTATTTACCAATCAGATTTTTATGACTTTTTTAATTTTTTAAGTTTCAACAGCCACTTTAATTTTTTCCTTT<br>TTTATTTCTTTTCGTTTTTTAAAGCTTAGAAATAACGCTTTAAAACCAAAAAAGTTAAAGCCTTTTTTTTTACTTCCTCCATCATAATTG<br>AAAACTTTTTATAAATTTATATATATTTTTGCACAAACTTTTTTTTATACATTAATTATCAGCATTTTTTTCCTTTATTTTGATATCCACT<br>TCTCTTCACAAATTTCTAATTTTTATCATCACCTTTGTATTTTCTTATAATTTTGTTATTAATAAAATTACAGCCACAGCTTTTAAAGTTT<br>TCTTTTTAACAATCAGATTTTAACAATTAAAGTATTTACAGCCAAAGTCTATACAGCCAAAATTTATACAGCCAAATTTTTACAGTCAA<br>AGTCTCTACAGCCACAAATAAAAGTCGTTACCAATCGGGGCCTTAGATTGAATATTGTAGGTTCAGAGGGTATTGTTATGAAGGAAGT<br>GACCCAAAAACAAAAATCAAATTATATAAATTTGTTATGTTCTTGATTTTTTCATTTATAACCAAAATACTAAAATCCACTACCAACCA<br>TTTTTCAAATAAATTTAATCTATCAACTAAAACCTTCAAAATTAAAAGTAACAAATAAAAATGATATATACCAAAACAGCAACTGAAA<br>GAACCGGAAAAAGCTTCTCTATATAAAAACCACAACACCACTCTCACAACATCCCCTTCTCTTTCGCTCTCTCTCTTCTTCCTTCTCTCC<br>TAATTAAAACTTCTCTGTCGCTTTCTCATAAACCTCAACTTATA
CsDof95	Csa17g090650.1	GAAAAAAAAAACATGAAAATCCGATTGGAAGAAATCGTGATTTTAGTAAAAGCTAATCCCATATGACTTTTGGATGGATATAGTTTAG<br>ATTTTATTATAATCTTTTCTGGCTCTTTTGTGTAATCATTGTTTTATTGATTCTTTTGTTAAACTTGTTTCGATTTTCTTGACTTATTAAA<br>ATAATACATTTCATTTTACATGTACATGTTCTTCTTGTTTAGTTATTTGGCCATTTTTCTTCTTACTTTATTATAATTTATTTGGCTTTCA<br>TGAAGGATATTTGTTTCACAAAACAAGGTAATTTAGATTTAACAGTAAGTGTTTGCTGCCTTGTTTATGCATATCTACTGAATTTCCTA<br>GGTAATTAGGACCTCCGATGTCGAAACGAATTATAAACAAAACGAATAAGGTCAATACGCGTCATTGTGAAAACTGTCTCTTGTGGCT<br>AAGACTTAAGGAACCTGAGCCATCCATTTGTCGTCCTAGAGAGCCACAAATGGTAGCCCGCACAAAAACGATCAGTGATAACGGAAG<br>TCGGCAGCGTAAGGCGATCGTCAAACGTGGTTTCGACACTTGTGTATGGTGTCTGTGGTTCAGTGTATTCACGATAGCATGGTACAAA<br>AAAAACTGACGGTGCTGATTCAATGTTCCATGGTGGGGTTCCAAGTGTAATGATGCCTTCTTTTTTTTTAATATATTATTCCTTTTTCAT<br>GATCCAACGAAATAATAAGACTTTGCTTGCTAAGTACGCTATCTTTTACATGTTTTTTTGTCTGCTAATTCGGAAGCCTTAGTGGGTCA<br>GAGAGCATCATAGCTCTTGAGTTTGTGCGTGCCCTAGTTGATTTTAAATTTGGGACAAATCAAAAAGTCTCTAAACTTATCTGTTTCTG<br>TCAAATTCAAAAACTTTATGTTTAATCCTATTAATAATTAACGTTTAACAAATCTATTTTAATCAGATGAATAACGTTTTGAACATAAC<br>TCGAAATCACTAATTTTAAAAATCAAAAGCGTCAAATTTGAGTCAGAATTTCATATTCCGTTACTTGCAAGTTGCAACATTTCGGAAGT<br>TATCTGGTGAACCTAACTTCGAAAGAGCAGTTCAGAATTTCATCTAAATAAGTTTCAAAATCAATGGGTCAAAATTTCATGTAAAAAA<br>GTTTCAAAATCTATGGGTTAAAGCTAAAAAGCATAATGACAGTTCGATTGCCTTCAACATATAGCATTTGAATTAGCAGACCGAGCTC<br>GACTATTAGGTTATTACTAGTATAAGTTTATTTTCTTGTGGACTATGAAATAAAATGATAAAAAGTTATAAGTTAAGTCAACAGGTCAA<br>TGGTAAAGTTCAACGCGTCGAATCTTGTCAAAGAAAACTATTGGAGGAAATTTCTAAGAAAGTTCGCTTAGTTTTTTATTTTATATTTT<br>TAAAGTCTATCCATAAATAGCATAACAAAATATATAAGTTATATTGAAATTTTTTAAAAAGTCATATATTTTAAAACATACTTAAATAT<br>AAAAACAGAATTACTTAAGAGGACAAAAAAAAGTATTAAATCAAGCTAACCATGACTTTGAAAAACATAGTAGAAAACTAATAATCC<br>TAGCAAAGTTACAAAATAGAGAAAACAAAGGTCGGCTGCTGTCACAACAATGAAGCAAAAAAAAAAAAAAAACACAGAGGTCAACAA<br>CAAGAAATAAATAAAATAAAAAAACCATGACAATCTTCAATAACACAAGTTTTGAAAAATTGTCATTTTGTGGGTAAAATAAAATAAA<br>GGGTGAAGAAGAAAACTCTTAAAACCCCACCACCAAAAAGCTCCACTCATTATCTCTCTTCTCTCATTCTCTCTATAACTTGTAACTTC<br>TTCTTCTTCCCCCCCACACTCCCAAATTAAAGAATCCATTTTTTTTTTGTTTTAAAGGTCTCTCTCTCTTTTTCTCTCTGTATGAACCAAA<br>ACAAACATTTTCTTGTATTCAGTCAGAGAAGAAGATAAAAAAAAAAAG
CsDof96	Csa18g033540.1	NNNNNNNNNNNNNNNNNNNNNNNNNNNNNNNNNNNNNNNNNNNNNNNNNNNNNNNNNNNNNNNNNNNNNNNNNNNNNNNNNNNNN<br>NNNNNNNNNNNNNNNNNNNNNNNNNNNNNNNNNNNNNNNNNNNNNNNNNNNNNNNNNNNNNNNNNNNNNNNNNNNNNNNNNNNNN<br>NNNNNNNNNNNNNNNNNNNNNNNNNNNNNNNNNNNNNNNNNNNNNNNNNNNNNNNNNNNNNNNNNNNNNNNNNNNNNNNNNNNNN<br>NNNNNNNNNNNNNNNNNNNNNNNNNNNNNNNNNNNNNNNNNNNNNNNNNNNNNNNNNNNNNNNNNNNNNNNNNNNNNNNNNNNNN<br>NNNNNNNNNNNNNNNNNNNNNNNNNNNNNNNNNNNNNNNNNNNNNNNNNNNNNNNNNNNNNNNNNNNNNNNNNNNNNNNNNNNNN<br>NNNNNNNNNNNNNNNNNNNNNNNNNNNNNNNNNNNNNNNNNNNNNNNNNNNNNNNNNNNNNNNNNNNNNNNNNNNNNACCTTTTA<br>GGCTGACACCTTTTTCGCCCATGCTCAGCATCTCCACGCTCGATGCACCGCACTGTCTTCAAGGTGTTGTGCTGGCAAAGAAACCTTGA<br>CACTTTCCCGCTTCGTCTGAAGTCCTTCAGGTACATTTTGCGATAGCTTGTAACTCGTACAGATCCTCTGTTTCTTTAAAGAGAGCCTC<br>GGTGGTTGCGCGAAAGAGAAGAGACGGCTAGGGCGAAGTCAGTTTTTTGCTCACGCAAAAAAATAGTATGGATGGTTATACCGACAA<br>TAGCTTTTATGCGTTCTACAATTCAATAAATTCCCATGCTAAGAAACCAACAAATACTAATATAAATTTGAGTATCTGTAATTGTTTTT<br>GCTTTAAATATGGAATTAGAATGAACGATCGAAATAGAAAATCACATATACTTTGTTGTACGAACTACGAAGGTCACTTTGAAACGGA<br>AGGGGTCCATTTAGGAAGAAACAAGACATTGCGATGAGTACGGGACCCGCGTGGTGACAACTCAACGGACCATTGGGACATAAAACA<br>CAGAAATGACAACGAAGTTGAGTGCGTGGGAATCCGACGTCGAATTCCAACCATCTCTTCTCTTATCCATCATCGTACGGTTACTATTC<br>ATCTTGAATCAAATAGATCCGATTTCACATTATTGATAACGACGACTGCAACTCATTCATTTATATATAAGTTGACAGCTTAACCGATA<br>CATTATCGGTGGAGTGGCTAAACGAGCTTAGTGATTATTTCGGACTATAGTTATAGACTTTAGGCTACAAGAGTTTTGTTTTATACTTC<br>CCAAGTTTTTTCTGTGGTTGTACAATTAATTACCTCAATATAAAACTACAATTTTGATTAGTAAAAGTTATTGTTTTTTTCGTAAGATAT<br>TATACAGTCAAAATATACAAATGTTAGATTATTTGGTAAATATGTTTAAATATGATTAGTAGTATTTTTATCAAAACCATCCAAAACAT<br>TAGTATTTTCGACCAATAAAAAATAACTCTTTTTTTTTGAAGTAGTACTCTCAACAAGTCTTGAAGAGAGATCGTAACACACTATCATC<br>AGTTTATGTACTTTCCCAATACCTTTATCAAACTACTAATTATGTAGAGAAAATTTTAATAATATGCGTACTGTATGTAGAATTTTTGTT<br>CACATTATAAAAATGAATTTAAACGGCGGCTTTATGTCTGTTCCAAATCACGTTCCGAATTGGTTTTCACATTATTCTTATTCATACCA<br>CCATTTTGTTTTAAAAATAATTAAATATTAAATAAAATAATATGAGATCGAGGTCACAACAAGAATTGTGTCTCTCTCTCTGCTCCCAC<br>ATTCCCATGTATATATACAGATGCTTGGCTTTTCCTTTTGCTTCACTTTTACTTTCTACTCGTATCCATCATTCTCTCTCTGATCTCAAGA<br>AACAATCTCAATCTCCCAACACATAAACACTACTAACACTCATTACTCTTCAAGCAGCTTCTCAGATTCATCAGCTCTT

Name	Gene ID	Promoter sequence
CsDof97	Csa18g034230.1	ATACAAAAAGTTTCTGAAAACAATGCGCAATCAAAAGCTCTACGCTTAACTTCAAATTCCAAAAGAAGCCTCTCGCGAAAATACCTAT<br>TTATGTTTTTGTTTCAATTTCACACGCAAAGTAAAATTCCATACTTATATANNNNNNNNNNNNNNNNNNNNNNNNNNNNNNNNNNNNNNN<br>NNNNNNNNNNNNNNNNNNNNNNNNNNNNNNNNNNNNNNNNNNNNNNNNNNNNNNNNNNNNNNNNNNNNNNNNNNNNNNNNNN<br>NNNNNNNNNNNNNNNNNNNNNNNNNNNNNNNNNNNNNNNNNNNNNNNNNNNNNNNNNNNNNNNNNNNNNNNNNNNNNNNNNN<br>NNNNNNNNNNNNNNNNNNNNNNNNNNNNNNNNNNNNNNNNNNNNNNNNNNNNNNNNNNNNNNNNNNNNNNNNNNNNNNNNNN<br>NNNNNNNNNNNNNNNNNNNNNNNNNNNNNNNNNNNNNNNNNNNNNNNNNNNNNNNNNNNNNNNNNNNNNNNNNNNNNNNNNN<br>NNNNNNNNNNNNNNNNNNNNNNNNNNNNNNNNNNNNNNNNNNNNNNNNNNNNNNNNNNNNNNNNNNNNNNNNNNNNNNNNNN<br>NNNNNNNNNNNNNNNNNNNNNNNNNNNNNNNNNNNNNNNNNNNNNNNNNNNNNNNNNNNNNNNNNNNNNNNNNNNNNNNNNN<br>NNNNNNNNNNNNNNNNNNNNNNNNNNNNNNNNNNNNNNNNNNNNNNNNNNNNNNNNNNNNNNNNNNNNNNNNNNNNNNNNNN<br>NNNNNNNNNNNNNNNNNNNNNNNNNNNNNNNNNNNNNNNNNNNNNNNNNNNNNNNNNNNNNNNNNNNNNNTACTATAATAAAT<br>AATTATTGAAAGATTGGCTTTGTAATTAAAATGTGGCAAGTGGCAACTATATTTCACTGTTCAAACCTTAAACTTACAACAAATGTTAG<br>ACGATATATACTAGATGTTAGTTTCCTTGTAGTTGAAGCAAGGGATTGGACCTTTTGACATTGCCTCCATTATATTTCCAATACAAAAA<br>GTTTCTGAAAACAATGCGCAATCAAAAGCTCTACGCTTAACTTCAAATTCCAAAAGAAGCCTCTCGCGAAAATACCTATTTATGTTTTT<br>GTTTCAATTTCACACGCAAAGTAAAATTCCATACTTATATATTTAATTACAAAAATATAAGTACATAAGAAATGATTCCTTTTTCAAGC<br>AACGTAGTTCAGTGAGATATTATATTATCTTGTGGACTATGTATGTGATATGTACTCAGTACGAGAATAATATCTACAGATTCAATACA<br>ATTTATACAGCATATATACGTAAACACTAGTCAACTCATTTCAATCATTTTTCATATAGTACGGGATTAGAAATTAGTAGGTTATTCAG<br>ACTTCTAATTCAGTATTGTTTTGCAGAAGAAAATCATTTTAAAAAGTTTCTTGGAAAATATTGATTTTAAATTCATATTTTATTAATTAC<br>GTGATCTCATGTATATTCGTTAGAAGATATTAACCTCTCACAGGAAGTAATTTGCATTACTTGTTTTAAGAAACAATGTACTAATAACA<br>TTTTTTTTTTAAATGTTTACTTAAAAAAATCATCGCATTAATATTTTTAAGCTTTTGGGATTCAATATAATTCGATGTGAAACTAAAAGA<br>TGTAAAAATCATATAAATATTTTTATCAATCATTGGATATTTTGATGCGACTCTTTGGCATACAAATAAATATATACAACCGAAATATA<br>AAAAATCGTTTAAATGCGAAGATATTAATATTTAATACATACATAATTTGATGGGGCCAAATTTTTGGACTCGAAGCTAACTTCTGAAT<br>ACAGCCATGCGAAGGGATTGTAAAATACAAAATATCGCATGGGCAGAACCGTCATTTGAAGATAAACTGCCTGTTCGGACATATGAA<br>AGATATGCTTTGTCAGAGAGTTGCAGAAGAAGTCTTTCCCATGCTTACCACTTCACCTCTCTCTCTCTCTCTTCTCTTTCTATTTCTCGT<br>ACCTTCTCCACTGTAATAACCTCCCCAATTCGAAAAACCCAAACTTGCTTAGATTGTATATTTTTCTTTATCATCTCTCTACGAA
CsDof98	Csa18g035890.1	TGTTTTAACTCAAATATCATATATTTTGAAACCGAGGCAGTATTTCATTGTCTTGCAATCAGTGCATATGAATGACTGGTTTTTGTTAC<br>TCACTTCTTTTATAAACTTCAGATCATATACTTAGAACGTATATATGTTAATTTAGGTTTGATGCTCTGAATTTGACAGTATAAGTACA<br>TTATTTCCAATTTAAAATCACTATAAACCGTGATACAAAAAAAAAATTCATCATAGAAATAGTATTTAGCTAAACTACGTTTTAGAAACA<br>AAACATGTTTATTGGGAAAATAAGAAAGAATTTTCAATTTGTCTGGTATCTAACAGAGAGTGTTGACAAAAAAAAAAACAAAAAAAA<br>AAACAGAGAGTAACAAGTGAAATAACCATTGTTTACGAGCCCTGCTAAGAGAGAGTCAAGGCTCAAAGCCTTAAACTAAAGCTGTCTT<br>AGGCCTTAGCCTCTTACTAAATAAATACTATATAGTATCAGGACTTGACGATGAACTATATTGTAAAAGAAGATTACATGGAGTGATA<br>CCACTGGTTGGACATAAACAAATCAATTGTTCAACTTCTTTTTATTTAAACGCACAATCTTGGATTGGAATAATGGTTTATTTCAAACA<br>ATTATGAATTATGAAGAAATGTTATCTGATATTCGGTATTCGGATGAATATTCTAAAATATCACAAACACAAACGCATTGTCTTTTGAA<br>GGTACAACACTTCTTTTCTATTTTGTTTTGTTAAGGAAGCTTCTTAAGTAGCTACGGGTCAGACTGCATGCAATCAGTTTGTGAAATCT<br>ATGATCTGACCAAGAGGTGCAGGTGAGTAACGGACCCACATGTAGAAGTTGACGTGGAAAGGCAGAGTCTGGTTAGATCCTCATGTA<br>AATCAATCCGAAAGACTCAAAGAGTTTGGTAAATCATACAATGTGGCAGAGACTTTAAAAGACCTTTTTGGTTATATCCAATGAGAGA<br>GCACCTCATATGTATTATCCTACCTTCTTCTGCAGACTTCTTATCTTAACGTACAACTACAACGGTTCACATAATGTAAATTCGTATAC<br>GGATGTTATATGGTTGTGATTTTTTTTTTAAATAAATCTGTCAAAAAAATTGAATTGAAAATGTTGAAATCCTTGTTGCATTTGTCTTCC<br>TACTTAAACAGAACAAAGATTCATATTATTAAATCTTCGGTAAAATATACCTTCTAAGTTCTAACAAAAGAAGGGTTCTTTAACGAAA<br>AAACGATTGGAAAAATAAATAAAAAGATGGTTCTTTCTTTCTAACCCAAAAGTGTGGATCGTTTTCAAATGAGGAATATTAATTCTCC<br>AACCAGCCAATAAAGAAATTGCACGTAACAAGACAGCTCTCGTGTGAGCTGCACCAAAGCCATCCCCTCGATGCTGACATTTACCTAC<br>CCAAAAGTTCGTAAACAGTTTACATTAGTTTATTTATTCCAATTCAAACGAACAAATAGAGTAGATCACTGTTTAAGATATGTTATAAT<br>TATAAAACCAATCTAATACCATATAATAAAGCGTGTTTATAGACCTTGACTAGTTGACTTCATGCTCTTAATTAGTAACACTTCGTTCT<br>TTCGTATGTTAGGCCTTTCATCCAAGTATTGTGAGTTTTTTGTTCAACGGAATTTTAGTGAAAGTCTTCAAACTAAGTATATATCAAAA<br>ATGATATTCTTACAAAGATTGGAATAATTTTAACAAAAGTTTAAAGAAAATAACTAAAAGATTATATGCTATATGGGTTTTGTGGAAG<br>AAAAGTAAAATATCAGAGGCCACATAAACTTGTGGTTGAGAATGAAACGAAAATGCTGACATGGAATCCACTTCACTTTTTAAAACGT<br>ATCAAATCCCAAATCTTACGATTTCCAAACCAAAAAAATTCCAATCTAAAACCCATACCTCAAAAAAAAAAAAAAGAGCTTCGAAGAG<br>AAACCTACGAGAAAAAAAAAAAAGAAGAAGAACAAATTCAAGGAAAGAAGAAATA
CsDof99	Csa18g036390.1	AGTTTGAGCTTACAAGGTTATTCTTTATCCCTCGAAGCTGGCCAAAAAAAAAAACCTCTTTTCTTCAATAATCAGTCAAGGAAAATACT<br>TTCCTAACAAGATTAATTAATGTGCATCGTCGTCAACCACATTTAACATTTTTCAAGATAGTATTTTATATTTTAATTAGTAAACTTTGA<br>AAAGAAAAACAAATTCATAACTTCAGACTATTTTTATAATCCTACCGTCGGGTAAAATCCCCTATTATTTTTAATTTACAAACGTAATG<br>CTTACTCCATCTAAATGAACATGGCTGCCAAAAGAATCAAAAATCAAAATCAACATGACGATTTAACACACACAAAATAAAAGATCGA<br>CATAATTGATATGTTTTCGTTTCTTTATTCGAATTTTGAATGTGAAATTATGTTTACAAGATTATCTATTTTCGTGTAATAGAACAGCCA<br>CATATATTATATTAGTTCCGATTTGTATCTGAAAGAAAAACAAAAAAAACACTTAAACTTAAAAAAAAGAGAAGATGATTACAGATAA<br>TAAACTATATATACACTAAAAATCTAAAAAGGAAAAATCTGTTTATCGAAAAGCACTGTTCTCAAACTTTTGTCTCTTTTTTATCATCG<br>GATTAAACTCTTTAACTTTTATTTCGAAATCCAATAATAAGAAAATATTCCCTTTAGTCTCATACAAATTGATGTTTTGAGATAATTTTT<br>ATCTCACAAAGATTGATGTTTTATAAACATCAAAAAATAAATATTAAAAATATTTTAAAAGTTTGAATTGTTATTGATTTAAAATAATA<br>ATATTTCTTTAATTAAAAAAAATATATTTAAATTAAATAATTATTGTTTTTTTAAAATATGTAAAAACTTTTAAATATAGACAGAATAT<br>CAAAAGTGGTTTTAACTCTTTGAATAATTTACTAATGATTCAGGGAAGTCAGAAAAAACCAAAAACTTTGAATCATATTATAAAACAA<br>CACATGTAAGAAAAGTGACATGTGGCAAGAGAATAGATACTTTGCTTGAGAGGAGCGAGCGAGTGGAACCCATCCCATTACTAGTCC<br>AGTAGTCCCAGTAGTGTCCCACCATAAGTTCTCAAACTCTCAATCCCTTCTTCTGTCCTTCTTTATCGTAAGTCTCAAAACACAGACAA<br>AACTTACGATAAAGATATTATTCTCTCTCACTTTTGTCTCTGTTGTGTGACGATCAAGCCGCTATTTCTAAACCCATCCGACGGCTCAC<br>CTTGTTTGTTTAGGTCCCATGGCTTTAATCTAAACCGTTGGATTTGATAAAGATGATTTGGTCAAGTTGACGTAGACCCACCCTAAGAG<br>TGTGGGTCCGAGGATTTTGGAACCACCGACGCTTTCTTTTTCCTCATCCTCCGCCACATCCGTATCTATACACTTATAGAGACATTTAT<br>TAATAAATACCAAATCACTCGAAATCAAAGTTTTTAACTTTTATCATGATCACACCAAAGTGAGGCCGTATTAACTTTTCAAAAATCTA<br>AATTTTGAATAAAAACAAATCTTTGTACCTCGAAACAGAACATATTGATTAAATAATATCTACATATCATGAAATTAAAGAAATAGTA<br>AAAATACACAGATACTGAGAAATTACTCACCGTACCCTATATTATTTCTAAAAATAGAAAATTCAAAGGAAGCTCCATAGTTCTCGTTT<br>TGGTGTCCATATATTAATATTAAACTTCTCAATAAATGTAAACCAAACTAATAATTATATCCAGAATTTATATATATTACATTGTCGCT<br>TTTAAACAAGATCACCTGAGTTATCCAAGATCAAAATCAATAATTAAATTTCAGAGAGATACATTAAGCATTTTTTTTTGAAATAATAT<br>TTCATTGATATGATACGAGTTATAGTAATATTTTAAAATAGAATTAAACTTGGAAAAAAAAATAAAGTAAAAAACAAATAAGTGTGAA<br>CTAATAATAACTTAAGTAAGCAGTAGAAGTAGCTTTGAGTGGAGAC

Name	Gene ID	Promoter sequence
CsDof100	Csa18g039010.1	CATGTTTCCCATTTTGTCTTCTCCGTTCTCTCGTGTTTCTTCACAGATGGAGACGTTTAATATATCAATCATCGAAACACAATCTCCTCT<br>CTTTTTCCATTTTTTTTTTATAATTTATACACACTTGTCTGTATTGGCCACATAACTAAAACCCAATTGAATCTGCATTTGATGGCAGTT<br>CACTTTGTACAAATGTCAATCACTTTTAGCTATAACTCAAGAGTATATATTAGCGATACAGAAACATGTCAGAGAGTTAATCTAACGTT<br>ACTTTCTTACTAAACCCTACTTCTTCTTCTTAGGGCCTCTAGATTACAAAACTCAAATTAATTATAAACTTCCTTATAATAACATAACTT<br>AGAAAAACTTCCTCAAATCTTTTATATTACTATATATATTGACGATTTGGACTCATATAAAATCCAAAACTTTCAGCTGGCTTGTATGC<br>ACCCTGAATCACGCCTACATAATACGTATTGATACTTATCCATTTACTCGATTTCCTTGGCTAACGCAAAATTTCTAGTTCTCATATATC<br>TTTTGTTCCAATAGTTTTGGTTTCCTTAATAGAGTTGCATACGCTTGACAGCTATATAAGTATNCTTTTTCCATTTTTTTTTTATAATTTA<br>TACACACTTGTCTGTATTGGCCACATAACTAAAACCCAATTGAATCTGCATTTGATGGCAGTTCACTTTGTACAAATGTCAATCACTTT<br>TAGCTATAACTCAAGAGTATATATTAGCGATACAGAAACATGTCAGAGAGTTAATCTAACGTTACTTTCTTACTAAACCCTACTTCTTC<br>TTCTTAGGGCCTCTAGATTACAAAACTCAAATTAATTATAAACTTCCTTATAATAACATAACTTAGAAAAACTTCCTCAAATCTTTTAT<br>ATTACTATATATATTGACGATTTGGACTCATATAAAATCCAAAACTTTCAGCTGGCTTGTATGCACCCTGAATCACGCCTACATAATAC<br>GTATTGATACTTATCCATTTACTCGATTTCCTTGGCTAACGCAAAATTTCTAGTTCTCATATATCTTTTGTTCCAATAGTTTTGGTTTCC<br>TTAATAGAGTTGCATACGCTTGACAGCTATATAAGTATAGCTGTATGTAAATAAAAGAAAATATTGTTTGTGATTGTATATAATTACAC<br>AAAATATGTAATGTAATATATAGTTTATACGGTATAAAGATGGTGCTGCAGTGCACACTTCACACATATAAAAAACGTTACCTCAACA<br>ATAGAAACAAGACACATATAACAAATTCTATCCGATTTGTTCCGATCAATTGCACAGTTTTATATGTTCTTCTGTCTTTTCTGCCACTTC<br>ATGTCTCTCTCTCATCTTCTCTTTTTGTCTCTATTATCTCTCACTCATGCCTCTCTCTCTCTCTCTATATATATATATCTATATAAGTA<br>CACACATTAACTACAAGGAGATTTTATGAAGAATTTAATCAAACAGTTTTGATAAAAGTGGCGGTTTACGATTATTCAAGTCTACAAC<br>TGTGTGAATGAACAAGAACACACCAAGACCAGAGTTTACAAGTACAACAGAAATAATATTTGTATTCTCAATATGAAAACTATGATAT<br>ACATGTTTTGACACATGAGATTTTATTATCCGTAAAGTTCATATCGAACACAATTATCACAGTTTTAATTAATATAACATTTTCAAGAC<br>ATAATATATACTAGAAATTATTTTGAATTAATTTAAATATATAAAAATGCCAAACAAACATAAACCCGACAGTATATATCACCCATATA<br>CATATATGCTCACATATATATAATTAATAATTTATATATAAATAAGTATAGAAGCATACGAAACCCTTTTACCGACTGTTGTTGTTGTT<br>GTTGTCTCTCTCCTCTCAATACTTTTACTTATTACATTCAAAGCAAGAAATCGAAAAAAGGAAGGAACCCCCAAAGCTTTCTTTCTTCC<br>TTCATTTTTGCTCTCTCCTCCCCATCATCATCATC
CsDof101	Csa18g040580.1	GGACACAGATCGTGGTTTAGATGACCATGCATCAATAAAGTTATATTCAGCTTTGAATATTTTTCATAATGGGATGTTTGGTGAAACAT<br>GCACCAACACAACATAAAAAGCCATATAATTAGGGCCTTAGCATAAAGAACGATTATTGCATGTTTTCTTTACTGTCATGTCAGATTGC<br>ATATCATTTTCATACAATTGTAGGGATCAGTTCTCAAACAAAAAAGAAAAAACACAAAGAGGATAATTAGCCCAACAATGTTATTTAC<br>GTTTTTTTCCTCGTATGTTTGCAATCGTTGCATAATGGGGTTTTGTTTTGGAGAGCGTCTCACATCCACATCTAAATACAAAAAAACAC<br>AAATCTCATTTGTAAGGCCCATGGTCTATAAAGTCGGATACATCTTTAAAAGAAATAGGCCCATTAGAAATACGGACGGAAATGACGA<br>AAACACCCTTTAGAAGGAAACAGAGGAGTAGAGTACTCTCAGACTAAGGGAGAAGAAAAGGACAAAAAGAATGGGATAAAGAAAGA<br>GATGAGTGTCAGAATTTGACCAGATTAGACCGGTCGGTACAACAGAGTAAGGACGGCTCTGAACCAGTGTAGACACGTCTTGGGCAC<br>ATACTTCTCACATGTCTCTCTCTCTCTCTCAAATGTTGGATGCTTTTTGGTGTGAAAGGGAGTGAAGATTTGCATGCTTCGTAATTCTT<br>TAGCTCTCCGTATCAATTCTTTTACCCGACAGCCTTCGCTTCTGTCTCTTCACAAAACGAATCTCTTCTGTCTCTTTTGACCGTTTTAGC<br>CTCCCACATTTACATTTACAACCCTCTTCTTCCGTATGGATTTGTCTCTCTTTCTCTCTCTCTCTCTCTCTACCTTCCTTAAAGATACATT<br>TGTCTTGTTTCCGCAACGCAAGATCATGTTTACTTGTTTAGTCAGAAGTCCTACGATTATTGTTACTCCTGTCTACTTGGTCTTGTTGTC<br>TAGATTTTTTTTACTTTTTATAGTCTCCTAAACAAGATAAGGTTTATATGACCAACCATAGCATAGAAAATAAAAGAAAACATGGATTT<br>GTGATTTTATATGCATAAGATTGACCATAACACGTAACATTGATCATTTAAGTTGGTCTAATACATACTGCTGTGATTTGATTTTAGTT<br>AGTGTTGTTTCTAAGGTCCTGAACGCATTGATTCTCGTATAGTAATTGTAAGAGCACATCTTCACACACCATAAATCTCTTAGGTCCAT<br>AGAGTGTACTAATTTTTTTTTTCTTTTGGGAAAAAGATGACACAACTTAATAACCATCGTAAATAAGGAAATTAGGCTAGTTATAGGA<br>GCCATAAATAAACCCTTTAACAGTATAATGGTTGAATTATTGGGAACTGCTTTACATCAATAAGCTCGCTCCCACAATTTGCCTATTGT<br>TGCCTACTCTCTTCACTCTTCATTTCCCGTAGACACGTACTTTTGACAGACGTTTTTTTTTTTCTTTTTAAATTAAAATCAATGTCATCAT<br>CTCTTAAATGGTGTTTGTTTCGACTCATAATAACTCGGAAGTACTACCTAACAACAAAATCAAAACTTAATTTACTTTAATTGGGTTTT<br>CTAGTCACTCACGAATCGCATTTCTGATAACCATGCAAATAATGTCTTAATATTTATTTTAAATCACTTATATGCCAAACAAAAACGTG<br>CGACAGATTATGGATGGAGGAGATTACATGTTTTGTTTGTTTTCCCATAATTTTCAAAACAGAGCCACCTACGTACAGTATAACGAAG<br>AGAGAGAGAGACAGGGCATCTCTTTCTATGTTCACAATAAACCCAACACACTCTGTCAAAAAGCTTCACTCTCTTCTCCTTCCCCATCT<br>CTTGTCTCTCTCTCTCTCTCTTCTCTCCATATAAGGAACCTCTTACTCTCTAAGAAAGTTCCATAACCTCTAACACTTCCAAGCTTCAAA<br>AAGTCTCAACTTTTTCAGCGAATTACCTACCTCTCTTCAAAAC
CsDof102	Csa19g029310.1	TTGTTTATTTTGGAATATTCTTGAAAACTATTTAAAGATTAGTACTATATCTCCATGTTTTATTTGTTAGTTTTTGGGTAAGAACTCTTT<br>TCTCATATACATGCTTTCACCATGTTTCACGTTAAAGAGCGAGTAACAAATTATTTGCTTTGTTTTTGATAACTAAACAAGTGTCTCGT<br>AGCTAAGCTTTATGCTCCAATACGATTTGGTTGTGAAATAAAAAAATATTCTCAAGGGGCCACATGAAATCTAAACCGTCCATGCGTC<br>GAAGCGCAGCCACAAATCGTAGCCGTAGAAATGACGCGATGATGGGAATGACAAACGAAAGTGGAGTGTTAGTCCCCACGTGTTGGG<br>AAAACAGAACACACGAACGCCAAATGGTCAGCTCTTACGGCAGCTTAACGCGACGACAATATCCTTAATTCTGTATCCATCGACACAC<br>GGACGGCTCCGATTCTATTCCAAATCTCGACGGCCACGATTCACATATTCGAGTGTAATGATCTATCTCTCATCGTCACCGAGTGCACC<br>ACATACATACTTGCTTCACAAGAACGCTAACTACTATTTGTCTACACTTATTCTTGAGCCAGAGTGGGTTGTGGAAATGATGAACTCGA<br>TCGGTGCCTTTTTTATATATAATTATATTCAATCTTTTTAGCTAAGCTAATTAGTATAATAAACACTAGATGATGCTTATTTAATGCAAA<br>TTTGATTATGTATAAAGAAGACTAGTTTGATAACTTCTAATATTTCTTATCTGATTAAAAACAAAAATGGAAGTCTACGTGTAGGGCTT<br>GTGCATGGATAAGAAAAACTCATGGGAAATGGGCGGGGCTTTGGGAGAGAACAACTCTTTTCTGACGTTTTGGTTGTCTTTTGTTTGA<br>CGTCTTCTTTGGTTAGGTTTTTTAATTAAGAATTCTTTTTTTTTTGTCGGCAAGTTTATATAATTAAGAATTAAAATGGGAAGGCTCTTC<br>TAAGCAAAATATTTGAACGTATGTAAAATGTTACTATTTGTTAGAATGTGGTAATTTGCTCCTTCCGTGATTTAATCCCCCTTGCTCAA<br>CTTTGTTGCTAATAGAAAACACTTTGACAAACCAAAAATTTTGATCTCTTATGGAATTTATTTCATCAGATTAATTACTCTAGAAGTTA<br>GTTGAGAGTCACTAGCTAGTGAGACTTTTGTTCTTGAAAGAAGAAAGGTAATTGTGTTTGTATTCGGGTTAAAGTAAAGTACAAATAA<br>TAGAAAACAAAAAGATACGTAGAATCGATATTTGTCTAAACATAAGAAGCCAAACAAAACCATCTTGGCACAAAGACAGGCGTATCC<br>AAATATGTTGGAAAAAGTTATTACCCCTCGATTTGTTCAAAGTTTTTTTTTTTAAACCATAATGCTCACAACTTTTATTTTTCTACATTT<br>GTAAAAGCTAATTGATCTTACTAACTCAAACAATTCTTCGAATTAATTAGACATATAAAAGTAAAAATTAGTACTTCCGTTTTATTTAC<br>TCTTTATACAATCGTTCCGACAAAATGACATCAAACAAAACAACTCTATGATAATATATATAGTACTATTAGTTAAAGAAGTCAACAAGT<br>GAAATGAAAAAAAAAAAAGTTCAACACCATCCTCTTGTCTTCTAATTACAAAAATTAATGATCAATTAACATAAATAATATAATCCCTAT<br>AAATTAAAAAAAAAAAGAAGAAGAAGGAAGATTACGTAGGATAGCATTAATCTTGTAATTTTGGGTTGCCCTATAAGACTGGTCACA<br>CACACCACAGCTCACAAACTTGTCTTGTCGTTTAGACTTTAAACCCAACACTCTCTTCCTCTTCCTTCCTTCTTCTCTTCTCTCTCTCT<br>CTCTCAAATTTTTTGTCTTATTTATGCGATAAGAACACAATTAATTCCTCAATCAAAATCTTTTTTTTTTGTTTTCTTGATTATTTGACAA<br>ATTTTTCAAGAAACACGAAAAGATCATAAAAGAAGAGAAGGAAAA

Name	Gene ID	Promoter sequence
CsDof103	Csa20g002520.1	CACACCTTTCTCGGTGCATGACGATGCTTACATTTTGAAACTGATGCAAGAACAAAAGCAACAACAAAACAGAGTTTCTTCTTCATCA<br>TCTACACAGCAGCAATCTCAACACAGCTCTGCTCATTCTTCCTCCAGTTCTTGCATTGCTTCTGGAACCTCAGGCCCGGTTTGCTGGAA<br>CTCGGGATCCATTGATACCCGCTGACCAAGAAAACTGAGATTCTATTCCCAAAGTCTCGAAGAAGAAGAAGAAGAAGAAGAGCAAGT<br>GATGATCTAATTGGTATACTAAAAAATTATACATCTGGTTTAATGTTCAACTGGGAGAGACTGTAAAATGACAAATTCATAGGCTTAT<br>CCAATTTTCCTTTTTATTGGAACTTTCGTGTAATATTTCACCAGAAACCGAGTCGGTTTACTTTTATCTGAGATTATTGAATTAAACCAA<br>ACCTGGCCGAATTAAACCGATTGGCTATCTGGTTTACTGTTCTGTATGTAACTAAAATACTGAACAATTTTAGTTTCCGGTTAGGTCCG<br>GTTCCCGAAATAGCGTTAGGCGATCTATAGTTAGTTAGGTCCGGTTCAATTTACGCGCGTGTACTACGAAAAATCTAAAATTCCAATTC<br>CCATGCAAAAGAAAAAAAGAGACATCGCGGCGGTTCGAGGGAAAGAAAAGCAAAACAAGTTGGGGACACTTCGGAAGAATGTTGGA<br>CTGTTTTAATTAAATTTACTTTATATATACATTTTTAAATTTTTAGTGTATCAACTTAATCTAATCATCTCTAACCATTTTTAATCATTTT<br>TATAATATATTAGTCCAACCTCTCTTTTATGTTTAATTCGTAATTGGAAAAAAGTTAGAAGTGGGAACCTTATTTTGTTAAGATTCATC<br>GTGAAGTATGTGAAATTGTATTTTCGTTTATATTTCTTTTCTAGCTTGGTATATCATTTGTTTTCAAGTTTGTTGGCTGACGGTTAATTT<br>CTTGATATGATTTACGTTTTTCTCTTTTTATTTTTCATTATGCGTCAATATGTTTGACTTTATATTTCACATTTTGTTTATCCTTTTGTTC<br>AGTAAATCTACACATTTCGAAATCTACAAAATCGTCCCCGACATCACTAAAGAGAGCTCTTTCTTTAGTGATATTACTGCCGATTAATA<br>TATTCAAGATAAGTGTTTAATTACTTAATTAATTAAAGCTGAGGTATATATACACGTAATATAACACAATTAAATGCATAACTTAGCTG<br>CAGTGGCGGATCCAAAACATAAAATAACATCATTTCTTTTACTGAAATGAAAAAATAATGAAAAATTTAGCTGATATAATCCATATAT<br>AAAAGCACTAGCTAGTGTCAGCTCTAATTTTCATATACAAGAACTAAGATATAACAATTGCAATTTTTTCTTGAATACTAACTTCACCT<br>AGACCCAACTCTTACGTACGTTTATCATCTTTTATTTTGTATTTGATATTTTGCATGGTAATAAAATGTTTCTAAAGTTCATGACAAACA<br>TCAATATATTATCATTTTTAGTGGTCTCTTTTTATGTATTTAAAATTAAATTAAAAAAATTGTTATGAATTTTACCGACTTTGATTTTTG<br>AAAAACACAACCAGTCAAGCTTAGTAACTTTTTTGAATATATATAGATTGACAGATATATATATATATATATATATATATATATATATA<br>TATATNAGAGCCTTGCAATAACACTGTGGACACACTCAAATCTTATTTTTTTTATTCACTATAAATAATTGATATAATATTCAAACATA<br>TTCCTCTATATATATGTGGGTATGTATATATATGAATAAAGGTTCTTCCTTTACCTTTGCCTTCTCTCTCTATCTTTCTCTAAGCTCTCCT<br>CTCCTCTTCTTTTCTCTCATATAAGCCTTCACCAAAACAAAAAAGCTTTAATTCCTTTATTTGCTCAAAAACAAAACCATTAAAAAAAA<br>CACTAAAGAGAGAAATATCATCCAAGAAGAAAGATCAAAG
