# Supplementary material for: Genome-wide identification and functional analysis of Dof transcription factor family in Camelina sativa
Source: BMC Genomics. 2022 Dec 8;23:812. doi: 10.1186/s12864-022-09056-9 (PMC9730592; doi:10.1186/s12864-022-09056-9)
Supplement: Supplementary file 12 — Additional file 12: Table S10. The 2000 bp promoter region upstream of the CsDof`s target genes. [file 12864_2022_9056_MOESM12_ESM.pdf]

Table S10. The 2000 bp promoter region upstream of the *CsDof*`s target genes.

Name	Gene ID	Promoter sequence
ROD1	Csa01g018440.1	ATTCACAGGGCGCTAGTTTCTAGTTATCCTAACTTTGTAAAAGATGTTTAACTACTATAATCTTACGTACATGTAGTGAG<br>ATTTCATGTTAAATTATAAAAGTAAGAAAAAGGGAAAATTGGAAATTGATGGTGTGGCTGAGGATATTTGTGAATTAAA<br>ATATATATCACATCGTAAGATCTATTTTGTACGTCGAGTTTTATTAAAGTTAGGAAAAAGTGAATCATCAAACATCAACA<br>TTGTCCCAAATCTTTAGCCTCCTCCACCGGTAGACAACAAACACGTCTAATTGGGTGAAGATTAGACATACACGTCCAAT<br>TAGCTGAAAAACAAACAAAATCACCACCTCTTGATTTACATTTACATTTACACCACGTGGTAATTAAATATGTATATGGC<br>TATGTAAAACTTACAAAATCTTTGGCAGAACTACAATTCAGATTTTTCCTATAATCTTCTAAACGTCTATGTAATTCTACA<br>GAACAAACGATAATATTATGGTTGTTAACATATTGAAAACATACAATACAACAATCAAAACGAAATACGATCGATAAGC<br>TTAACTACACTGAATACATGGCCAATAATTAAACTAACTACTTCATCCTCGTCTTTGGTATCCAGACCAGTTCAATCAAC<br>CGCGGGACTCACAAACTCGAGAAACTTCATGATTGACACACTGAGAATATTCCACGTGGGTGTTGAGATAAATTAAAGA<br>GAGTTTTTATTTTGTCTGAAAACAACTCAGTCAGTCACCATGAGTTAGATATCCTACCTTCACTCGAGAAATTGTTTTTTT<br>TTTAAGATACAAAATTATGATAGAATATAGAATGGTTCAATTATTTGTTAGATACTTTAAACAAAATTTTACTTTCGTGC<br>AAGGATCATAAAATTGTAACACATGAATTACGGTCAAGTACTATAGACAAATACAGTACAACTACAATAATATAAACAG<br>ATGCTTTGGGTTATAGTATAATTAACACTTCACATACAAAAATAAACAAATCGTGTTAACAAGAAACTATAATCCAAAAA<br>ATCAGAAATCAAAACCGTGATGGAGTGAACAATGTCACTAATATTCCAAAAAGAAGAAATTAATTTTGTTCGTTTCATTC<br>ATATTTCTTGGTAAATGTATCCATCAGTTTGTTTCTTCCTGTTTTTGTTTTCTTATTGTTCACTACGTGCACCAATGCTTCT<br>CATGTTTGGAGCACGAGATAGTGTTTTGTTTTTTACTCCACCAACATATGAGAAAGTCTTCCAATATTAGTTACAGTAAC<br>AATTTAGCATCAGGAGATTAGCAGCCCTTTCAAATATAAATAATTTTACCACGATTTGTGAAACATGTAAGTATGCGGTA<br>CTTAATAAAATATATTTTCGAATGTTTATTGTTAGTAGTATCATACGAATAACAATGATTTTTTTTTTTGAACAACAACAA<br>TGATCTAATCTAAGTCTCATACTCATGTGTTTTTTCTTCTTCTAACCATACGTGACACATGGATAATATTAGCTAAAATCA<br>CTAATAAACAACATGTAACCAACTGTCATTTATGTTTTTCAATGATATTGGAGATTTAGGTAGTTAAGAATTTAAGAGTA<br>ATCCAATAGCCAATAATAGTTGTATCAAAAAGTGTGAAAGAAGATATTTGCGCGAAATGGAGAACCAATGCAGTAAATT<br>TAGTTTTTTATATTCAACGAAAAGCACCAAATCCTTTGATATATTCTCCATAATCTTGAATTAGGATTGTTAATACATTGC<br>ATACACCAATAATGCATTTTATGTGATAAACTATAGTTTCTATAGGTTTTGGGGAATCTCATATTCTCTTCTCTCTCTTTT<br>TGTCGTCGAAATGTATTATACTTATTAAAACATAAACATTAGTATAAAAAGAACAACATGTCGGTACACAAACAAAAAA<br>GTAAAGCCCACCAAAGAGTTAGAGTGTATTTAACTAACAACCATAAAATTAAAATGTCGTTCTCAAATTCACTTTCAT
FAD3	Csa05g033930.1	AAGAGTTATGATTGTACCAGAATAATAACGGACACTCAGATATTTCTATAACTATAATTAACAAGAAACACATAATATAT<br>AGTCAGTCTATCAACTAACCGGCCGGCCTTATAAGTAATTACCACAAAGAGTTATAGGTGAGGTGAGCCTTGGCTATGA<br>TATGACTGACTAAAGTCTTTCCTATGTTTCTGATTACTATAAACGTGTTACGTTTAGGTTGCCAACTCCAGTCATACAGA<br>AGGTCGATATTCATGTGAATATGTTTTTTTCCAAATAAAGAAAAGTTGTTGCAAGTAATTAATTGCATATATAGTGAATT<br>GTTGTAGTCAAAATATGTATAAAATAATTAAGGCGTAAATCGTAAATGTGTGCTTGAAAAACAGTTAAGAGAAAAGAAG<br>AGGTAGAGGATTTGCTGTACAAATTCAATGTCTCTCCTCTACTAACCATTTTTTTTGTCAAACCTCCTCTCTACTAACCAT<br>AAATGTAATGATTCAAGCTTTGAATTATGAGGACGAAGGAAATTGATAACAGTGTATTTTGTTATGAAAACAAGTTCAC<br>TATTTTTATTTTTCTGGTCTCTTTTTCGTTTTAATAAATCCATGGGCTGATCCATCGTTAGAAACACACCGCAATATTACA<br>ACGTGGATTTCTTTCAGAAGAGTCACCGAACTGTATATTTGAGTTTAGAATGTTTTCCGACTAATGTCCGAGGATGTAAA<br>CCATATAATTTGGTTTATACTATCCAAAATATTTTTAAGTCTTTATAAAAAAATTTCATATTTTCTAATACTCTCTATATTT<br>CATAAATATAATTCTTTTTAGTTTTCCAAAAAAAAAATATCAGAAAATATTTTATACTATTTTCTAGGCATTAACATTTTTA<br>TTTTTATCCTTGTTATAATTAGAGATAAAACAACTATATTTAATTTTAGAATATATAAGAGTACATTATGTTTTTTTTTTT<br>TTGAAAAAGAGAGTACATTATGTTTTGCAACGTAACTTTGACCACCATGTGTGTATAAGCTTAAAAATCTATCTTCATGA<br>AACATAGGAAAATGTTGTGATATCTTCATGAAAGATGAAACCTAGGAAAATCTATCTTCATTGACTACACAATTTGTTCG<br>AGTTGTGTTGTTTTTAATCCGCATCAAGTGCTCTGCCTACAAATAATAATTAGGGACTCATCATGTTGTTGTAATTAATG<br>TGATGAACCAAAAAAAAAAAAAAAATCCACCACTTAATTGTATAAATCGGAAACGGTAAAAATCGGTGATGTTGAACTAT<br>TGAAGTGATTTAAAACTAATTGGAAGTTGAAACAGCTACAGACCTCTTTATTTTTTGGTCAAATAAATCAATGTACTGAT<br>GTGTCAATTACATTTTAATTATTCCTCGAATTAATCACCGACCTAATTTTCACTACTATGTATTAATACTAGCCACTAATT<br>TTAAGATATTTTCCTTTTTTTTTTTTAAACTATTTGTTGTCCATTTTACATTACAGCATATAGTGTTTTTTTTTTTTTTTGT<br>TTATAAATTAGTTTTCTCCTATATAGTGTTTCAGTATAAGAAATATTATACTCGTTGTCTTGTCAGTTTCGGTGCATAAAA<br>ATGGTCAAGAAATGAATATAATTTACTTGGTATACTTTGGTACGTGCAAGGAAAAATATTCACCTTCCTTTCAGAATTAC<br>CACTTATACAAAGAAATAAATCATGAGAAATATATATTTTCAAACTTACACGAATTTTGAAGAAATGGTAAAGTTTTGTT<br>CTTGTTAAAAAACCAAAAAACAGCAAAATTACCCTTTTTATCCTTTTAAATAAAATAAATAAATAAACATTAAAAAAAAA<br>ATTGGAGTTATTCTCTCATTTTCTCCCTTTATATAAACAAATCACACAAACCCCAAAAAAATTCATCAAACCCTTTCTTCT<br>TCTCACCACATTTTTTCTTTCTTTCACTGAGAGCCCATTTCTCTTTCTCTCTCTTTCTCTCTTTCTCCGGAG
LACS3	Csa16g057140.1	CTTCTATTTCAGCAATAATAGACCTATTGGCAACCCTTTGCATTGCATCCCTTCTTATAAGTTCTAACATTTGGACAAATG<br>GCTTCCTCCTTGCAATCTTTAAGGTTCTATTGTATGACTCAGTGTGATTGTTGTGTGTATCAGCACAACAACAACCAACC<br>CTGAAAAACGCCCTACACCATGAACTTGGATCCTTTCGTAGTAGAGTCTTACAAGCCTGGGGATCAAATTCCTTGAATAT<br>GCTCAAATTTCTTTATAATCCGCCTTGTTGTAACTGCTTGCAATTCGNNNNNNNNNNNNNNNNNNNNNNNNNNNNNNN<br>NNNNNNNNNNNNNNNNNNNNNNNNNNNNNNNNNNNNNNNNNNNNNNNNNNNNNNNNNNNNNNNNNNNNNNNNNNNNNNN<br>NNNNNNNNNNNNNNNNNNNNNNNNNNNNNNNNNNNNNNNNNNNNNNNNNNNNNNNNNNNNNNNNNNNNNNNNNNNNNNN<br>NNNNNNNNNNNNNNNNNNNNNNNNNNNNNNNNNNNNNNNNNNNNNNNNNNNNNNNNNNNNNNNNNNNNNNNNNNNNNNN<br>NNNNNNNNNNNNNNNNNNNNNNNNNNNNNNNNNNNNNNNNNNNNNNNNNNNNNNNNNNNNNNNNNNNNNNNNNNNNNNN<br>NNNNNNNNNNNNNNNNNNNNNNNNNNNNNNNNNNNNNNNNNNNNNNNNNNNNNNNNNNNNNNNNNNNNNNNNNNNNNNN<br>NNNNNNNNNNNNNNNNNNNNNNNNNNNNNNNNNNNNNNNNNNNNNNNNNNNNNNNNNNNNNNNNNNNNNNNNNNNNNNN<br>NNNNNNNNNNNNNNNNNNNNNNNNNNNNNNNNNNNNNNNNNNNNNNNNNNNNNNNNNNNNNNNNNNNNNNNNNNNNNNN<br>NNNNNNNNNNNNNNNNNNNNNTAAGGCTACACAACAAGACTAGACAATAAACTAAGTAACATATAAAGAAGAATTGA<br>ATCTTGCCGACAACAACTCCACTTTCCCACTTGTTTAAAACGACAAAATAAAAGTTAAAAATCAACAGCAAAATGCCAG<br>CTTTACCTCATTGGGACGAGAATCAACAGGCAATGTCTTCTTTATTCCGATTTCCAGCAAAGATTTCACTGCATTATCTCT<br>GTTAAAAAACCTAATTGAAAAGATGCTCTCACAGTTCTTCGATATCTTCGATAAAACGATAAAACTTTTTCAAACAACTT<br>TTTCACTTTTCTTGTTTTGATCGATGAATAATTAGAAAATGGGCCTGTGTTTTTTTTAATTTAGATAAAAAGCCCATTAAA<br>ATTGGGTCCGGGTCGGGTATTTTATTTTGGTTGCAGGTGCTCGGGTCGGGTTTTAGTTTTCCCCGATTATGGTTCTGATG<br>AGTCAACGCCGGTCGTTGCCGGGAGAAATGACCCGGTGAATCGAGATTTTATGTCCATGGTGTAAAACACATGACGTCA<br>AGTACTTGGTGCATGTGGCCATGCCAAACATATAATTGATGTAGTTGAGCATACATTATTAAATTTGAAATGTGTGGCCA<br>TGTATTGCGTCATACCTCGTGTAGCTGGACATCTTTTTTCCTTTTTCTTATATATAGTACTTAATTACTTAAGAGCTCTGG<br>ATTATCGTAAATATGTGAGTAACATGTACACCATTAACTAGATCCAGTATATAGGAAAGTGCCCACATTTTTTATTTTTA<br>TTTTAATCAATTAATTTTTCTTGATTTTTTTTTTTTGCTAAAATCAAAATGTTATAAGGACTTTATTATTTCTTCCTTTTGG<br>TTAATTGATTAAAATATAAATAGATAAAATCTAATAATAATACTATGAGCTTTTATTGAGGGGAAAAAGTAAAACAAGA<br>TATAAATTACTTATAAATACTATCACCCAATATGGAGGAGTCAGCAACCCTCGTGTCTATCAAATCATTTTTGGTTTTCCT<br>CTTTACAACATTCACACGCCATTTTAAAATTTGTCTCTCTAGTTGCTGCACCTTTACAAATCAACACGAACGATCAAAAC<br>ACATAAAATACACAGATAATTAAGGACAATAAACGCAAAAGAA

Name	Gene ID	Promoter sequence
SAD6	Csa17g070600.1	TGTTTGAATGTCATTGTTAAGGTCTAAGAAATAAGCTCTTTTATTGATGTTTCAGTAATTATCTCTTCGTCACTGGTTACA<br>AGTATATCTATTGTCTACTCTACAGGAGTATTGCATGTCTTGTAGAGGATCCGATGTTGTGGAAGCTACCAAGACAGTTG<br>AACTTGTGATCCCTAGGGGTTGGCAATGTTTTTGACATGGTTTTTGATTCTAAAACATATCAGTTCTGCTATGAAATGGG<br>TTGCAAAATTTCATTCACACTGTTTACGTTCCTTTCTCTGAGGATTTGTGATTAAAATGATATTGGTCACATTTATTGTAT<br>GGCTGTCTTGTCCTTTTATTATGGTTGGCAATGTGCATTTCCCGGTTAATTTTCTTTTGTCTCCTGATATTTGCGGCAAAT<br>ACTAAACAAAGAATTTAGAATTGTGATTTCCCGAGTATAATACATGATTTTGCAGAACTAGATAATGATAATTTGGTTTT<br>CGATGGCATAATTCTGTCAATCTCTAATTTGCTTTCTTGTCTCTGTACTTGACCATAGGGGTGGAGTCTGATGCTACAAT<br>CCCAATTCCAGATGCCGGCCATGTAAGATCAAGAACAAGTCAACCTGGGAACTTGTATATCAAACTAAAGGTTTGTTAA<br>AAACTATCTTTATTTTCATCAAAGTTTGCTTGTATACATTTTCTGAGATGTGCTTAGAGAAGTTTGAATCGAACCCACCTT<br>GACTTTGATCAATTCAGCTTAAGAGATAGACAATCGATGCAAGCATCAAAGTCATGTTATAAATTATATATGAACTTTGA<br>ATGATTCATTCATTCCAAATATAATTTACACCGTTGTTCACAGATTTACACCGTTGTTGTCTCCAGTGGGTCACTATATAA<br>TCAAGATTTATTTTACCCTGTAAATAGATTGTTTATGCTTCTTAAGCTTCATAGTAGTCTTATATGTTATCATCGCTCTTG<br>CCCAGGTAGGGTCTTACTACATGTGGCATTTTCCTTAAATTAATTATTACCCAAAGCTTACAAACCAATAAAGGCCTATG<br>AAAAGTCTAAGAAAAAAAGGATGAATTTCAAGTCGAAATTGTTTCTCTTTTGCGTATCTTTTACTGAGGTTCCGACGATG<br>ACACGATGTTCCGAGAAGAATTAAAAATAAATTAGCTAAGAATTTATTTAAATCTTTATAATGATAAATATGTATTAATT<br>CAGTTATTTACAATATTATTAACTATTAACTAAACCAAAAATAAAAATAAAAATAAGTAATTTGTGACGGAACTTAGTTG<br>ATTGCATGATATTTTGGTTTTCTTGATGTGTCAGACTTAAAACATTCTTTAGAATAATAAAATAAAACTTTTGTTATTTTC<br>TTAAAAGTAAAATAAATTTTTACTAAAAAAGACAAAAGAAATAAATTTCATGATCTTGTGTTTTTGCTAAGAGAATGTCA<br>TTCAAGTGGTTACCTTATAAATATTAAAATGTATAAAGTATACAATAAAATTTGAGCAAAATCAGAATACTAATTATTAC<br>AAGAATTTTAAATGCTCAATTTTATTTATTTTAAACTATAATTAATTATTAAAAATAGTCCACATACCAACAAGTTCCAA<br>ACAAATTAAGAAAAAATTACAAACAACATAAAAAACACAACAAAATAGTAATAAAAAAAAGAAAAATCCTAGTATTACC<br>TAAAAATTGAAGTTTAAACGATTTTAAGAAAAAAATTGAAGAAAAAAAAGAAGTTACTCTCAGTTAAGACTTAAATGGG<br>ACAAAATTAGAAAAATATAAACAAAACCCATAGAAACTCAAACCAGTTAGCATGAGCACCCCTTGTTTCACCCCACGGT<br>TTGTCTAACAAAAATACCTGCCAACCACCAATTACTACTATACCCTCAGCACTCTTTGACTATTTAGTCCACCACAACTCC<br>ACAGTAGTCGTCATCACTCGCAAACAAAAATACCTCCAACCAAAGTTTAGAAGTCTTGTCTCCTTTACCATACAAA
CsPDAT1-A	Csa13g016300.1	TCTGTTTTTCTTGCGGAGGAAAGAATGTTTGGTACTGTTAGAGAGCGAAGAGAGATGCTCTGCTTTAGAAGTGGATAGA<br>GTGAATGGAGAATACACAAGCGAAGCCATTTTTTTGGCTGCCGGATTCTCCAAGAGTTGTAATCAAAGAGAAGAGGATA<br>AGAGAAGAGATTGAGAATTGAGAATTGAGAGAAGAGATCTCTCTATCTTGTTTAGTCTGGTTGTGTCTTACACACTTGTG<br>TTCTTCTGCTCCAAAAGCAACGCTTCGGAAGAAGGAACTAAAAAAGCAGCGTTGGTTTTGAAGGTAAGTGGTGGGAGGG<br>AAGGACACGTGGACACAAGAGGGGTTCTCTACTTGTAAGGTCGAGTTTGGTTGGATGTATATCCGCTTAGGACACAATG<br>AGATGGACTTTACTGCCTTCTGATTGGCTCATTTAGATTTCTATTCATATTTTCATGGTTTTGGGAGTGTAGTAGAGATAT<br>TGGAGTGTTTTATTATCTTTGTTTTTTAGTTGATAAGTTTATGTTGCTGTCTCGAGATGATCGTGCTTGCGGTTTCAGCCA<br>TGTAAAAGATTTTTTTCCGATGACGACACTTTTTTATTTTAGGCATACTAATTTGGGGTAGCCGGTCCAATCCGGCCAGC<br>ACAAAATATGTATAGTTTTTTTGGTTTACCTTGGTCCAGTCTACTATTTGTCCACTAGTAATTTTTTTTTTTGTATTCAGG<br>AAAAAACACATATAGAGACCAAATAATAAATGCAGAAAAATAAATAATAACTATAAGACTCGTAATAAATATATTTTAA<br>AAATCAAAATTTTAGTTGTAAAATTCTAGAATTGCATTGATGCATTTAAATTTCATCATCATCATCAAAGTTTGTTTATAA<br>AAGATGGAAGTGAAATCTTATCAATTTCACCATATAGAAGATTTAACTGAAAGTATATATTAAAGATTATAAGTAAAGT<br>GTAGATGGGCATGTAGCATTGCAGTTTGAACTAGATAGTCGTTGCATCACGTTTGCATATTCTTATCTTTATCTTACAAG<br>ATCCATTCCCCCCCCCCCCCCCCCCNTCCTTTATGAAACCTAAGTCTAGGGTTTGAATTTTACATTAAAACATTTCTTAA<br>GTTGACTATAAGAGCTACTAAGAGTTGAGATTACTCAAATCATAAAACTCTTTGATTCACGATTTGGTTTGACGGAAGTG<br>AGATATGATCTCTTTGTCCCTTCAAGATAAGAGTTTCTCCTTAGGCGTTTCAAGTTTCTTCCTCTGCCTGTCTTTTCCCTC<br>TTTTTTTCCTTAGTTCATTTTTTTGTCATTAAACATGAGTTCTAAGTTCGATATCATAAAGGGGGTAGAAAAGCGTAGAT<br>AAATTAGGTTCTTGTACTAGGCTCATCAACCTAACTTAACCCAACACCAATTTGGTGATATACGTACATGTAACATGATG<br>TTTGCAATAATTTTTTGGTGCAGTTTTGTAGTATTTTAGTGTGTCCAACCTAGGCTGGTTCGCATAAGCCATACCCACACT<br>TCACAAGCACACACACAAAAAAAGGTCAACCTTGTGAGTGTGGGCTAACGTCATGGGCTTGTGGAGTGTGGACTTGTGT<br>CAAGTGTTCTAAACATAATTATGGTCCAAATACTTAAAATTGATTATAACGGAAGCGGCGTGTTAAAATAGGTGATTAA<br>AAAAAAATTGTTACGGACTAAACTAAACAATGAAATCAACGAAACGGATCGGACGGGTCTTAGGTTGACCCTTGAACCG<br>CAACCAGGAGCAAGTGGTCAAGTTTTTGCCCATACGGATACGGTCCGAGTCCCTTGGAAATTGGAATTATCAGAACGAA<br>TAAAAAAAAAAAAAAAAAAAAAAAAGTCGAACAAAAAAAACAAAATTTGATTTTTGATTGGTATCAGAAATTCGCAGCG<br>AAGGAGAGCTCTTTGTCTAATCTGGGTTCTTCCAAGAGATTGACAAAGTCCATAGCTTCGTCTAAGTGGACAAGGTCAC<br>AGGGGG
CsPDAT1-C	Csa20g019000.1	TAGATAGATTTTTCTACAGAGTTGTAACCAAAGAGAAGAGATTGAGAATTGAGAGAATTGAGAGAAGAGATCTCTCTAT<br>CTCTTCTGGTTTAGTTTGGTTTGTGTCTTACACACTTTGAGTTCTTCTGTTCCAAAAGCAACGCTTCGGAAGAAGGGAAC<br>TAAAAAAGCAGCGATGGTTTTGAAGGTAAGTGGTGGGAGGGAAGGACACGTGGACACAAGAGGGGGACTCTACTTGTG<br>AGGTCGAGTTTGGTTGGATGTATATCCGCTTAGGACACAATGAGATGGACTTTGCTGCCTTGTGATTGGCTCATTTAGAT<br>TTCTATTCATATTTCATGGTTTTTAGGAGTGTAGTAGAGATATTGGAGTGATTTATTATCTTTGTTTTTTAGTTGATAAGT<br>TTTATGTTGCTGTCTCGAGATGATCGTGCTTGCGGTTTCAGCCATGTAAAAGATTTTTTTCCTATGACGATACTTTTTTTT<br>TTTATTAGGCATACTAATTTGGGGTAGCCGGTCCAATCCGGCCCGCACAAAATATGTATAGTTTTTTTGGTTTAGCTTGG<br>TCCACTCTACTATTTGCCCACTAGTAATTTTTTTTTTTTTATTGTATTCAGAAAAAAAAACACATACAAAGACCAACTAATA<br>AATGCGGAAAAACAAATAATAACTATAAGACTCATAAGAAATATATTTAAAAAATCAAAATTTTAGTTGTAAAATTCTA<br>GAAATGCATTGATGCATTTAAATTTCATCATCATCATCATCATCATCATCCAAGTGTGTTCATAAAAGATGGAAGTGAAA<br>TCTTATCAATTTCACCATATCGAAGATTTAGTTTTTCTTTTGACAACCATATAGAAAATTTATCTGAAAGTATATATTAAA<br>TTAGATTTTTAAGATTATAAGTAAATGTAGATGGCCATGTAGCATTGCAGTTTGTACTAGATAGTCGTTGCATCACATTT<br>GAACATCCTTTTCTTACTCTGGTTCAATTCTCTTATGAATCTTAAGCTTAAGGTTTCAATTTCACACGATAACATATCTTA<br>AGTTGAATAGTAGCTAGGGTGAAGATAAACGAATGACAATTTCTTTAATTCACGATTTAATTTGACGATAATGAAATAT<br>GAATTTTTTTCCTTCAAGGTTGAGTTTCTCCTTAAGGGTTTCATGTTTCTTTCCCTGTCTTTTCCCTCTTAATCTTTATTTC<br>AATTTTCATCATCAAAAATGAGTTCTAGGTTCCATATTATATTATAAAGGGTAGAAAGGTTTAGTTGATTTAGGTTCTTG<br>TAGTAGGCTCATCAACCTAATTTATCCCAAGGTAGGATAGCAAAAATTCTTCTTGCAATTCGAACCAACACCAGTTTGGT<br>GATAAATGATGTTTGGCTGCAATAGGCTTTTGGTGCAGTTTGTAGTATTTAACTAGTGTGTCCAACCTAGTCTGGTTCGA<br>ATAAGCCACCCATCGTACATCCCACAGTTAACAAGCACACACAAAAAAAAGGTCAACCTTGGGAGTGTGGGCTAACGTC<br>ATGGGCTTGTGGACTTGTGTCATGTGTTCTAAACATAATTAAGGTCCAAATACTTAAAACTGATTATTATAGTTGTAAAA<br>AAAATACAATTAAAACGGAAGCGGCGTGTTAAATAGGTGATTAAAAAAATTGTTACGGACTAAACTAAACTAAACAATG<br>AAATCAAACGAAACGGATCGGACGGGTCTTAGGTTGACCCTTGAACCGCAACCAGAACCAAGTGGTCAAGTTTTTGCCC<br>ATACGGATACGGTCCGAGTCCCTTGGAAATTGGAATTATCAGAACGAATAGAAAAAAAAAAAACAGAAAAGTCGAACAA<br>AAAAAAAACAAATAGAAACAAAATTTGATTTTTGATTGGTATCAGAAATTCGCAGCGAAGCAGAGCTCTCTTTGTCTCTC<br>TCGATTGATCTAAAAAATATCCCTAATCTCGGTTCGTCCAAGAGATTGACAAAGTCCATAGCTTCGTCTAAGTGACAAGG<br>GGG

Name	Gene ID	Promoter sequence
CsDGAT1-C	Csa15g084220.1	ATCTTATAGTTTACATTAGCGCATCTAGCTAGCGAGTAGTACTTGGTAAGTTTTTAATAACGACCTCTATGACTTGGCCT<br>TGGCCTAGGAGCTTGGTTTACAGTTTACATCATGTATCGGGCTATCAGCTGACTCGACTAAATGGTTGGTGGAACGGAA<br>TACTATTTTTATAAGCTTGAAGTTATTAAAATAGTCTATAGTTATCATTAAAATAAAAATATCACGAGTAAAATATTTTCT<br>GTTTTAAATATAGTTTCTTTTGTTTGTTATGGAACTAATTTCTTTATTTATGTTTATTTTTTGGTATTTGAACTCTTTTCTT<br>TAAAATAGACACATCAACTTGGTGTTCTTATTATCAGAAAACAGCATTTCTTCTTTTCTTTAAAAATTATTATTACAAGAA<br>AATTTTTGCTTTGTTTCTATAACTTACAGTAAATCCCCAGGTTTATGGAGGGACCCGAGACTAATCCCTTAAGGAGGTGC<br>AGCTCATGGATGGACCCTCTTTCCGGGTATTCAAATGGGCCGTAAGCAAGGACCCATATCCATATTGTCACATAGTAAAT<br>GAGTAGCAATTCCAAATCTGGGGAGAGGTCCGAGACTAATCCCTGAGAGAAGTGCAGCCCACGGATAGACCTTCTCTCC<br>GGGTATTCAAATGGGCCGTAAGCAAAGGCCTATATCCATGTTGCCACATAGTAAATGAGTAGCAATTCCAAATCTGGAG<br>AGAATTGATCCTGGCCTGGACCAACAAGACAACTCTTCCAACCAGATGGGAACCACTAGCCCACCACCATGTGGTTAAA<br>AAATCTTGCTTTGAAAAAATGCATTCAATCTTTATCATTTTCAGATGATTAATATTTTTTATAATTATTTAATTGATAAGA<br>CCTAATCTATAGTTTAATCCAGGAGGATCGGTTACAGAAGTAGTATTTTAACCACTTGACTACGAATACTTCCTATACGA<br>TTATCTTTTTACAATAATAAAAGAAAATAAACCATTAAATACATTGGATTAAGGAGTATTAGCAAAAATACTATCTATTG<br>CAAAAATATAGAAACACAAATAGAAAAATATTATTTTTCTCAAACAGGATTTAAACGGTATTTCAAAACATTAAAATAAT<br>TTCCTAATTTCAACATATTTAATATTATTTAAACACCAAATCATAAACTAGAAAACAATAGGAAAAAAACACTTCTGCAA<br>CCACTTGACTACAAACACTTTCCATATAATTATCTTTTTACTATAATAGAAAAATAACAAATCCATTAAATACATTGGAT<br>TAAGAAACATTTGCAAAAATACATTTTTACAAAAATACTATCTATCGCAAAAATATAGAAACACAAATAGAAAAATTTT<br>ATTTTTCTCAAATCAGATTTAAACAGTATTTCAAAAAATTAAAATAATTTCCTAATTTCAACATATTTAATATTATTTAAA<br>CACCAAATCATTAATTAGAAAACAATAGGGAAATTTTTTTATGTTTTTAAAAAAGTTGGGGTACATATTTTCGTGGCGAT<br>GTTATGAATTCTCTCACCCGAGGATCTTATTATGCAAAAATTAAAAAAGAAAAAGAAAAAAAGAATAAAACCATAGAAA<br>AATATACTCGCAAACAAAAAAATATCGCAAACACCGCTTTTCAATTAAATCATTCGTCACCATTGTTGTCATCTTCCTCCTT<br>CTCTCTCTCTCTCTCGTGAATCTTTTTTCTTCTCTTCAAAGTTCTCTTTTAAACTTTTGCTTCTCTCTTTATAGGGAAACCA<br>GACACGATTCCACCGATTCCTTCTCAGCTTCTTCTTCTTCCTCCCCCCCTTCTAAACAAAACAAACCTTCTCTCAATATTC<br>TCTCTTCATTGGATTCTGATTCTCTTCAACNNNNNNNNNNNNNNNNNNNNNCCTTTTTTTTTTTTTTTTCAATTTCATCATC<br>TGCATGTTTCTCGATTCTCTCTGACACCTTCCTTCTCTAACGTTTCTTCGGAAGCTGTTTCCGTCAATTCGATAA
