# Supplementary material for: Genome-wide identification and functional analysis of Dof transcription factor family in Camelina sativa
Source: BMC Genomics. 2022 Dec 8;23:812. doi: 10.1186/s12864-022-09056-9 (PMC9730592; doi:10.1186/s12864-022-09056-9)
Supplement: Supplementary file 13 — Additional file 13: Table S11. The primer sequences of 6 CsDof genes used for qRT-PCR. [file 12864_2022_9056_MOESM13_ESM.pdf]

Table S11. The primer sequences of 6 CsDof genes used for qRT-PCR.

Name	Forward primer (5'-3')	Reverse primer (5'-3')
CsActin	ACAATTTCCCGCTCTGCTGTTGTG	AGGGTTTCTCTCTTCCACATGCCA
CsDof27	TAGGAAAACGGTCAAGGGAT	AAGGTTCAGCGACGACAAAG
CsDof54	CAAGAGTCCTGCTTCTCATT	CTTCATTTGGCAATGGGTTT
CsDof60	ATCCGGCGATTAAGCTGTT	AGAATCACCCATCTCATCTTCA
CsDof63	CAGAGATATTGGACCGCAG	CCTAGACACTAGCTTTGGAC
CsDof83	CCTCAAGAATCATCAAACCG	CCACTAGTAGTAGTACAATTCTGA
CsDof95	AAGAGAGGGTTGTTACTACG	TCATCGTTACTCCTCCGTAA
