# Supplementary material for: Genome-wide identification and functional analysis of Dof transcription factor family in Camelina sativa
Source: BMC Genomics. 2022 Dec 8;23:812. doi: 10.1186/s12864-022-09056-9 (PMC9730592; doi:10.1186/s12864-022-09056-9)
Supplement: Supplementary file 14 — Additional file 14: Fig. S3. The total lipid content in leaves of wild type (WT) and CsDof13-transgenic lines (T1 tobacco plants). Fig. S4. The Malondialdehyde (MDA) content of wild type (WT) and CsDof27-transgenic lines (T1 tobacco plants) before and after salt stress. [file 12864_2022_9056_MOESM14_ESM.pdf]

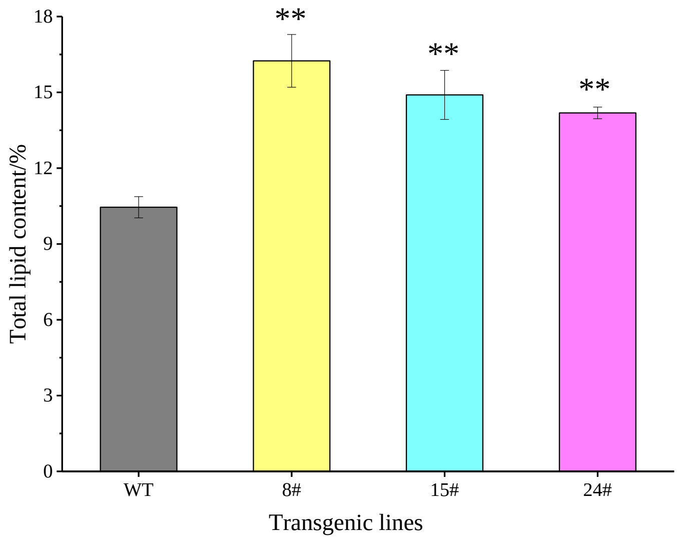

Figure S3. The total lipid content in leaves of wild type (WT) and *CsDof13*-transgenic lines (T1 tobacco plants). Statistical significance was determined by one-way ANOVA and multiple comparison by the Duncan method, $^{**}P < 0.01$.

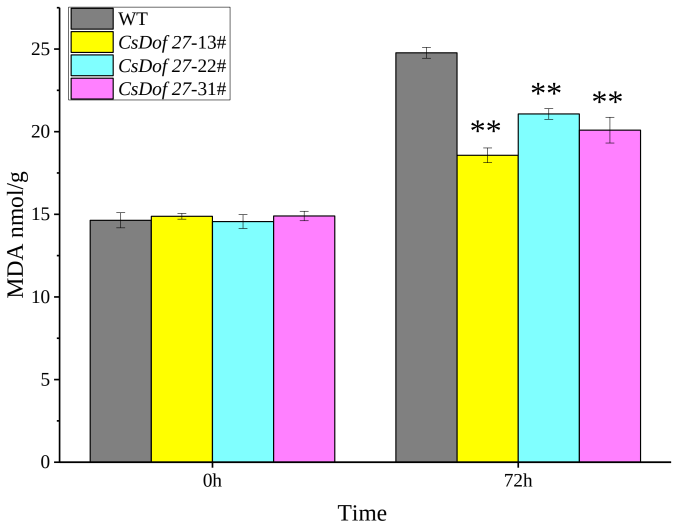

Figure S4. The Malondialdehyde (MDA) content of wild type (WT) and *CsDof27*-transgenic lines (T1 tobacco plants) before and after salt stress. Statistical significance was determined by one-way ANOVA and multiple comparison by the Duncan method, $^{**}P < 0.01$.
