# Supplementary material for: Genome-wide identification and functional analysis of Dof transcription factor family in Camelina sativa
Source: BMC Genomics. 2022 Dec 8;23:812. doi: 10.1186/s12864-022-09056-9 (PMC9730592; doi:10.1186/s12864-022-09056-9)
Supplement: Supplementary file 3 — Additional file 3: Fig. S2. Chromosomal distribution of 103 CsDof genes. [file 12864_2022_9056_MOESM3_ESM.pdf]

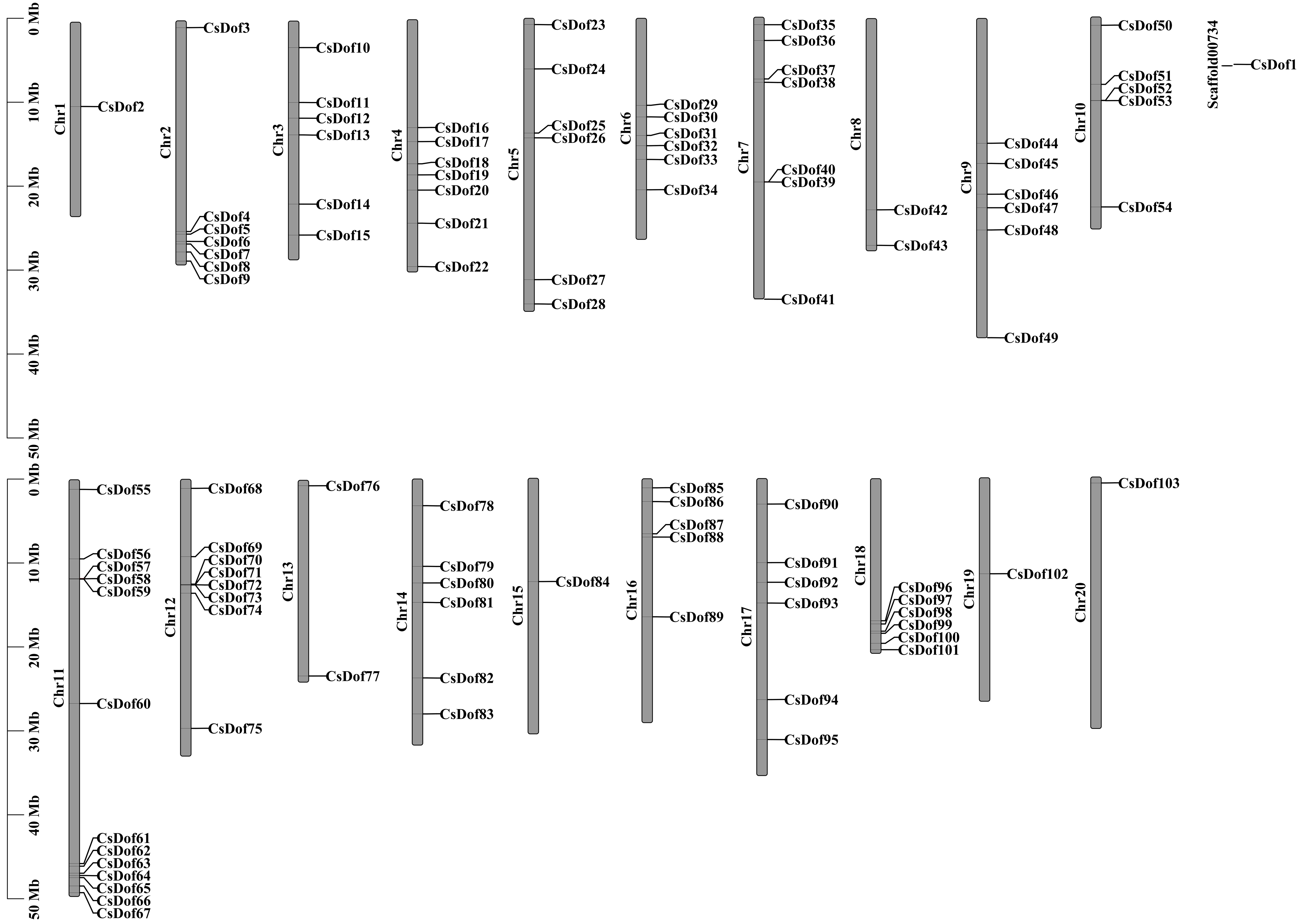

0 Mb
10 Mb
20 Mb
30 Mb
40 Mb
50 Mb
Chr1
CsDof2
Chr2
CsDof3
CsDof4
CsDof5
CsDof6
CsDof7
CsDof8
CsDof9
Chr3
CsDof10
CsDof11
CsDof12
CsDof13
CsDof14
CsDof15
Chr4
CsDof16
CsDof17
CsDof18
CsDof19
CsDof20
CsDof21
CsDof22
Chr5
CsDof23
CsDof24
CsDof25
CsDof26
CsDof27
CsDof28
Chr6
CsDof29
CsDof30
CsDof31
CsDof32
CsDof33
CsDof34
Chr7
CsDof35
CsDof36
CsDof37
CsDof38
CsDof40
CsDof39
CsDof41
Chr8
CsDof42
CsDof43
Chr9
CsDof44
CsDof45
CsDof46
CsDof47
CsDof48
CsDof49
Chr10
CsDof50
CsDof51
CsDof52
CsDof53
CsDof54
Scaffold00734
CsDof1
0 Mb
10 Mb
20 Mb
30 Mb
40 Mb
50 Mb
Chr11
CsDof55
CsDof56
CsDof57
CsDof58
CsDof59
CsDof60
CsDof61
CsDof62
CsDof63
CsDof64
CsDof65
CsDof66
CsDof67
Chr12
CsDof68
CsDof69
CsDof70
CsDof71
CsDof72
CsDof73
CsDof74
CsDof75
Chr13
CsDof76
CsDof77
Chr14
CsDof78
CsDof79
CsDof80
CsDof81
CsDof82
CsDof83
Chr15
CsDof84
Chr16
CsDof85
CsDof86
CsDof87
CsDof88
CsDof89
Chr17
CsDof90
CsDof91
CsDof92
CsDof93
CsDof94
CsDof95
Chr18
CsDof96
CsDof97
CsDof98
CsDof99
CsDof100
CsDof101
Chr19
CsDof102
Chr20
CsDof103
